# Supplementary material for: The Diversity of Methylation Patterns in Serous Borderline Ovarian Tumors and Serous Ovarian Carcinomas
Source: Cancers (Basel). 2024 Oct 18;16(20):3524. doi: 10.3390/cancers16203524 (PMC11505613; doi:10.3390/cancers16203524)
Supplement: Supplementary file 1 [file cancers-16-03524-s001.zip › GeneRegions.pdf]

Comparison of beta values distribution, gene: POLR2D(m) , region: promoters(m)

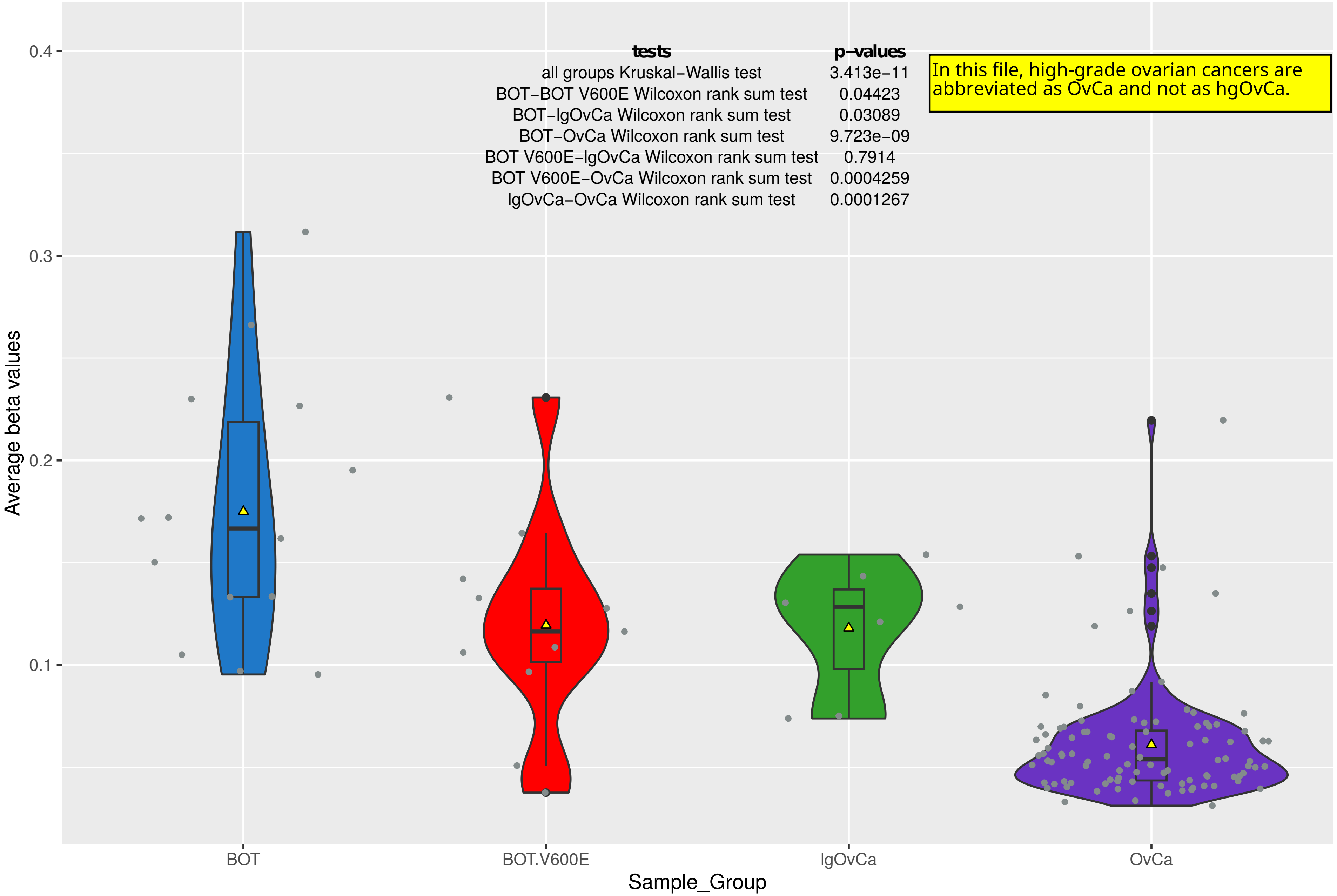

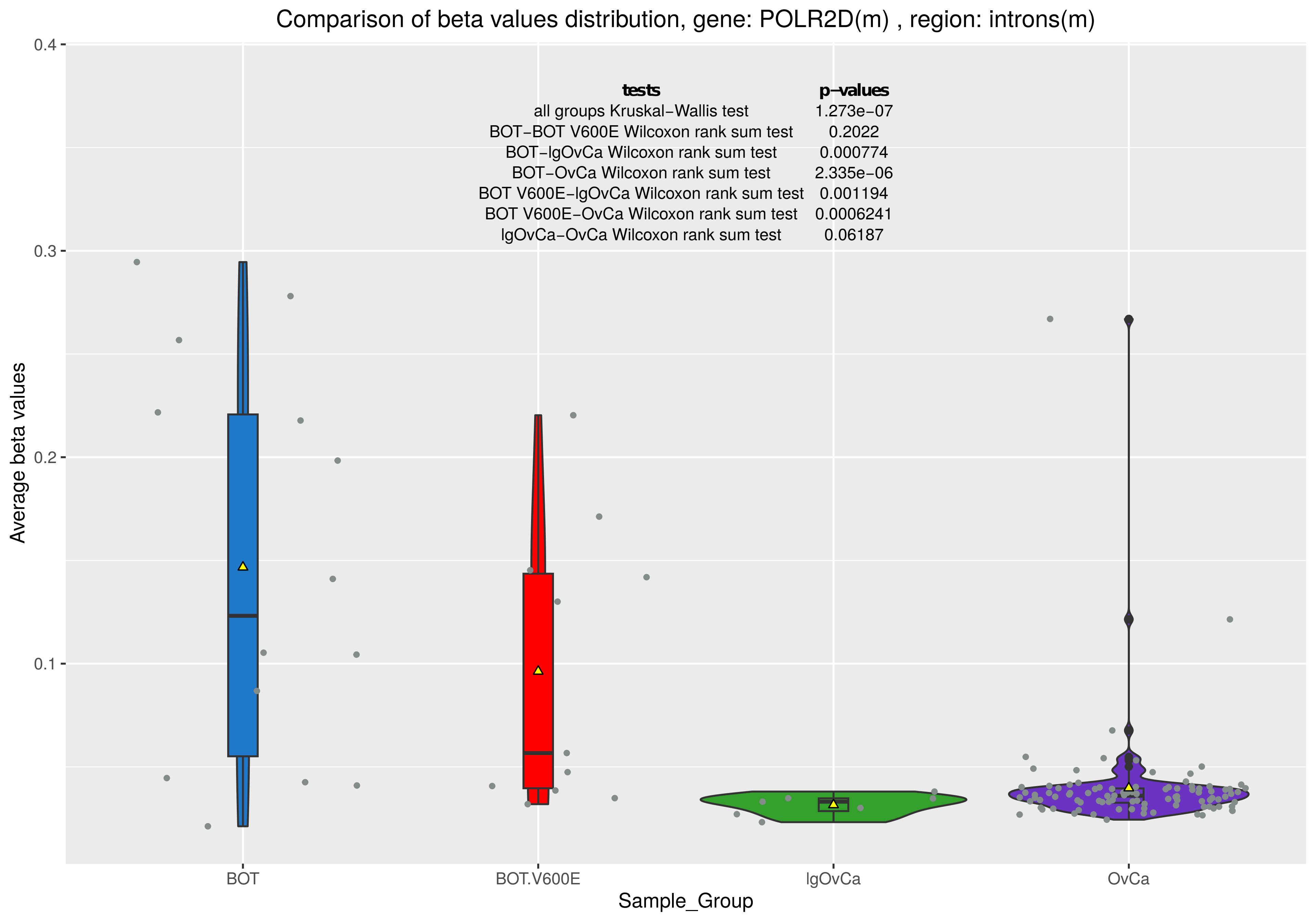

Comparison of beta values distribution, gene: POLR2D(m) , region: introns(m)
tests
p−values
all groups Kruskal−Wallis test
1.273e−07
BOT−BOT V600E Wilcoxon rank sum test
0.2022
BOT−lgOvCa Wilcoxon rank sum test
0.000774
BOT−OvCa Wilcoxon rank sum test
2.335e−06
BOT V600E−lgOvCa Wilcoxon rank sum test
0.001194
BOT V600E−OvCa Wilcoxon rank sum test
0.0006241
lgOvCa−OvCa Wilcoxon rank sum test
0.06187
Average beta values
0.4
0.3
0.2
0.1
BOT
BOT.V600E
lgOvCa
OvCa
Sample_Group

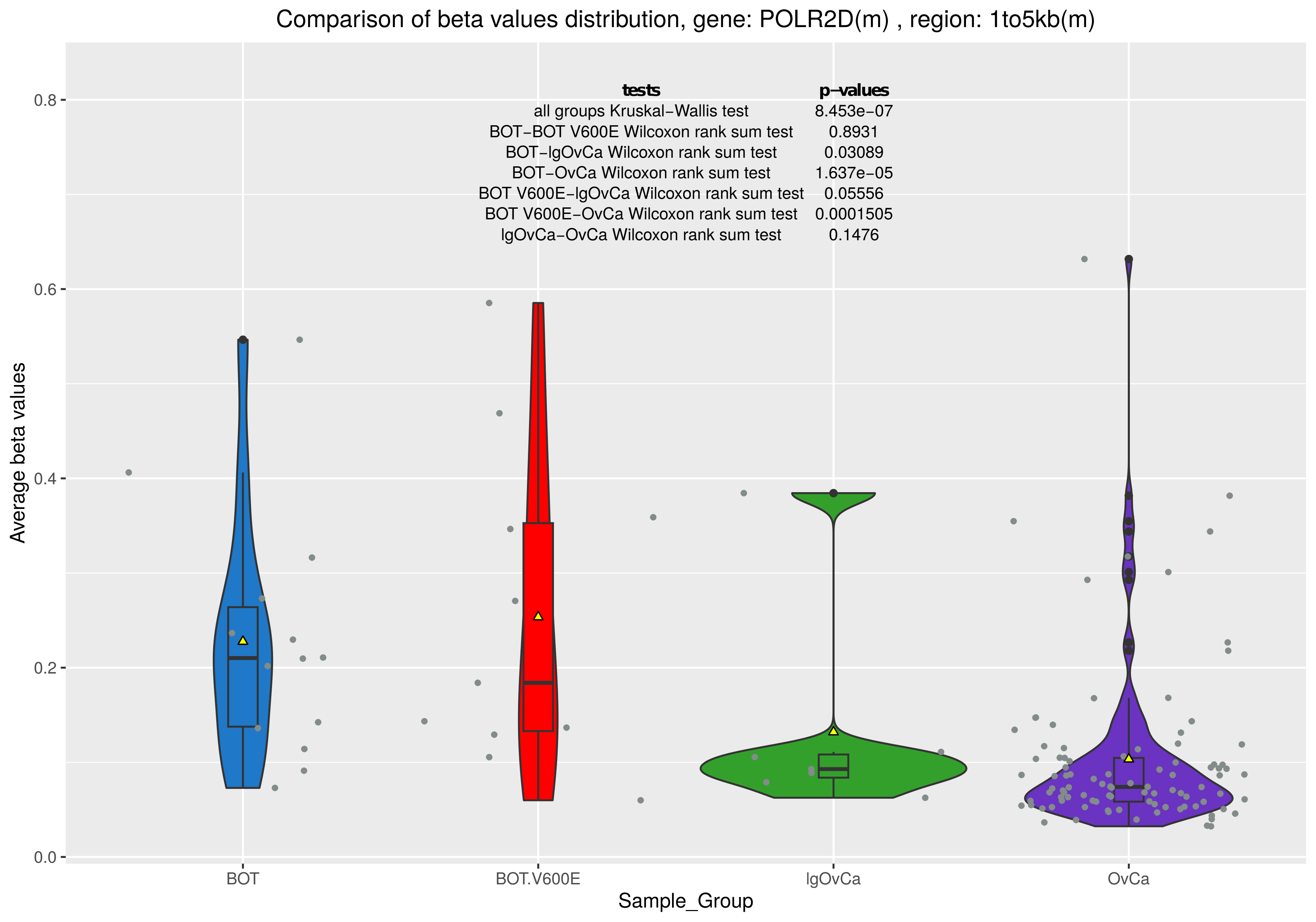
Comparison of beta values distribution, gene: POLR2D(m) , region: 1to5kb(m)
tests
p−values
all groups Kruskal−Wallis test
8.453e−07
BOT−BOT V600E Wilcoxon rank sum test
0.8931
BOT−lgOvCa Wilcoxon rank sum test
0.03089
BOT−OvCa Wilcoxon rank sum test
1.637e−05
BOT V600E−lgOvCa Wilcoxon rank sum test
0.05556
BOT V600E−OvCa Wilcoxon rank sum test
0.0001505
lgOvCa−OvCa Wilcoxon rank sum test
0.1476
Average beta values
0.0
0.2
0.4
0.6
0.8
BOT
BOT.V600E
lgOvCa
OvCa
Sample_Group

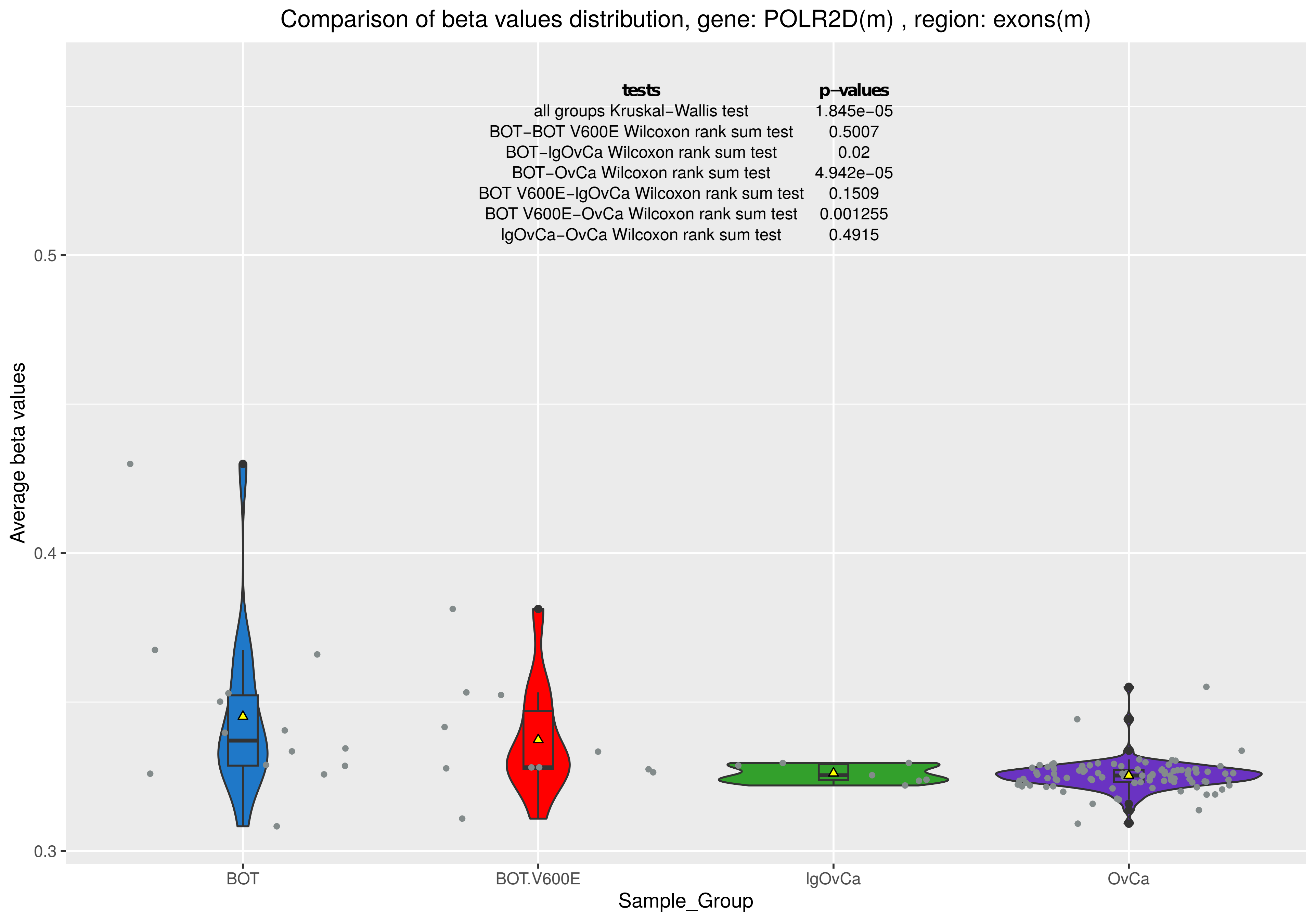
Comparison of beta values distribution, gene: POLR2D(m) , region: exons(m)
tests
p−values
all groups Kruskal−Wallis test 1.845e−05
BOT−BOT V600E Wilcoxon rank sum test 0.5007
BOT−lgOvCa Wilcoxon rank sum test 0.02
BOT−OvCa Wilcoxon rank sum test 4.942e−05
BOT V600E−lgOvCa Wilcoxon rank sum test 0.1509
BOT V600E−OvCa Wilcoxon rank sum test 0.001255
lgOvCa−OvCa Wilcoxon rank sum test 0.4915
Average beta values
0.5
0.4
0.3
BOT
BOT.V600E
lgOvCa
OvCa
Sample_Group

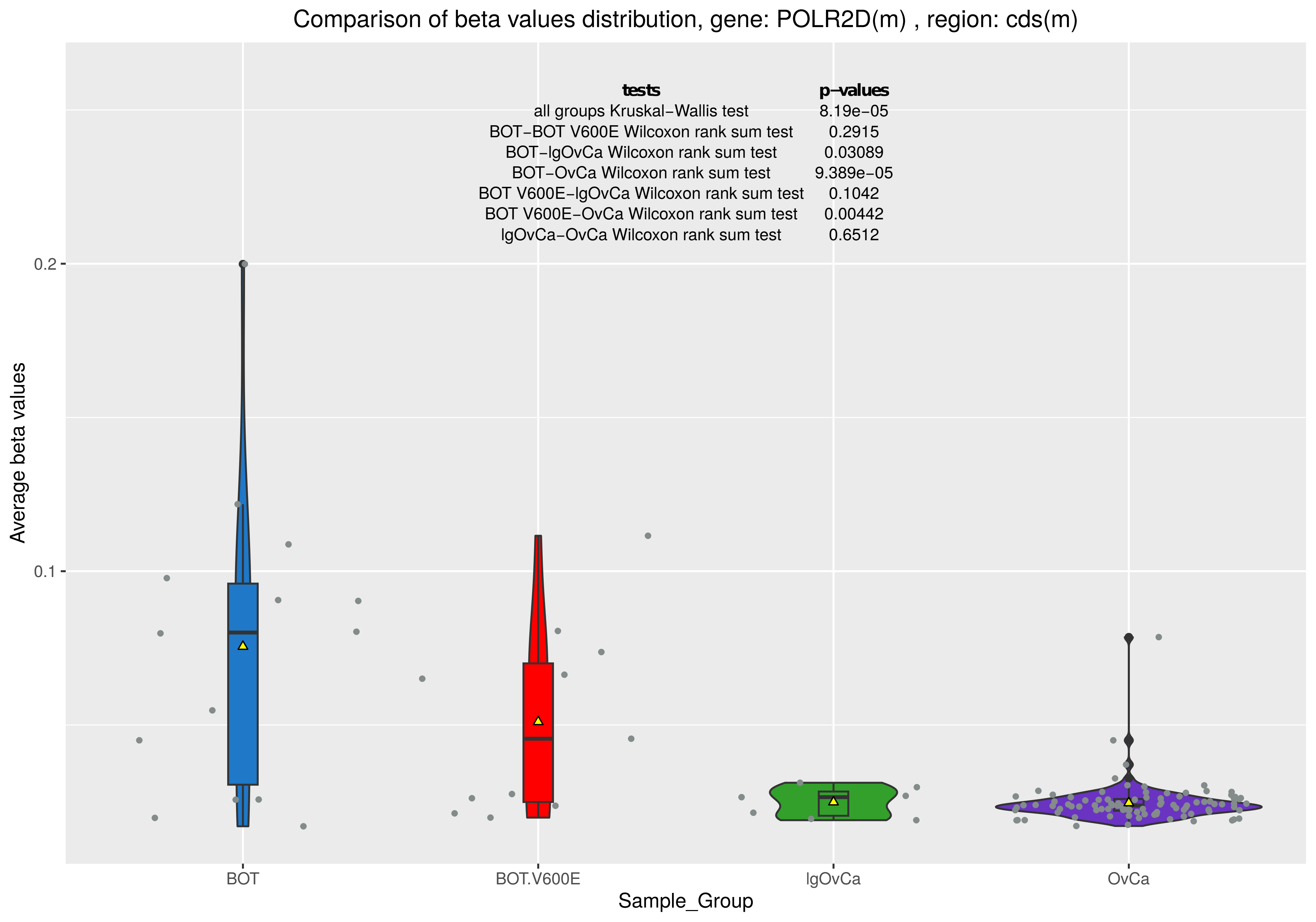

Comparison of beta values distribution, gene: POLR2D(m) , region: cds(m)
tests
p−values
all groups Kruskal−Wallis test
8.19e−05
BOT−BOT V600E Wilcoxon rank sum test
0.2915
BOT−lgOvCa Wilcoxon rank sum test
0.03089
BOT−OvCa Wilcoxon rank sum test
9.389e−05
BOT V600E−lgOvCa Wilcoxon rank sum test
0.1042
BOT V600E−OvCa Wilcoxon rank sum test
0.00442
lgOvCa−OvCa Wilcoxon rank sum test
0.6512
Average beta values
0.2
0.1
BOT
BOT.V600E
lgOvCa
OvCa
Sample_Group

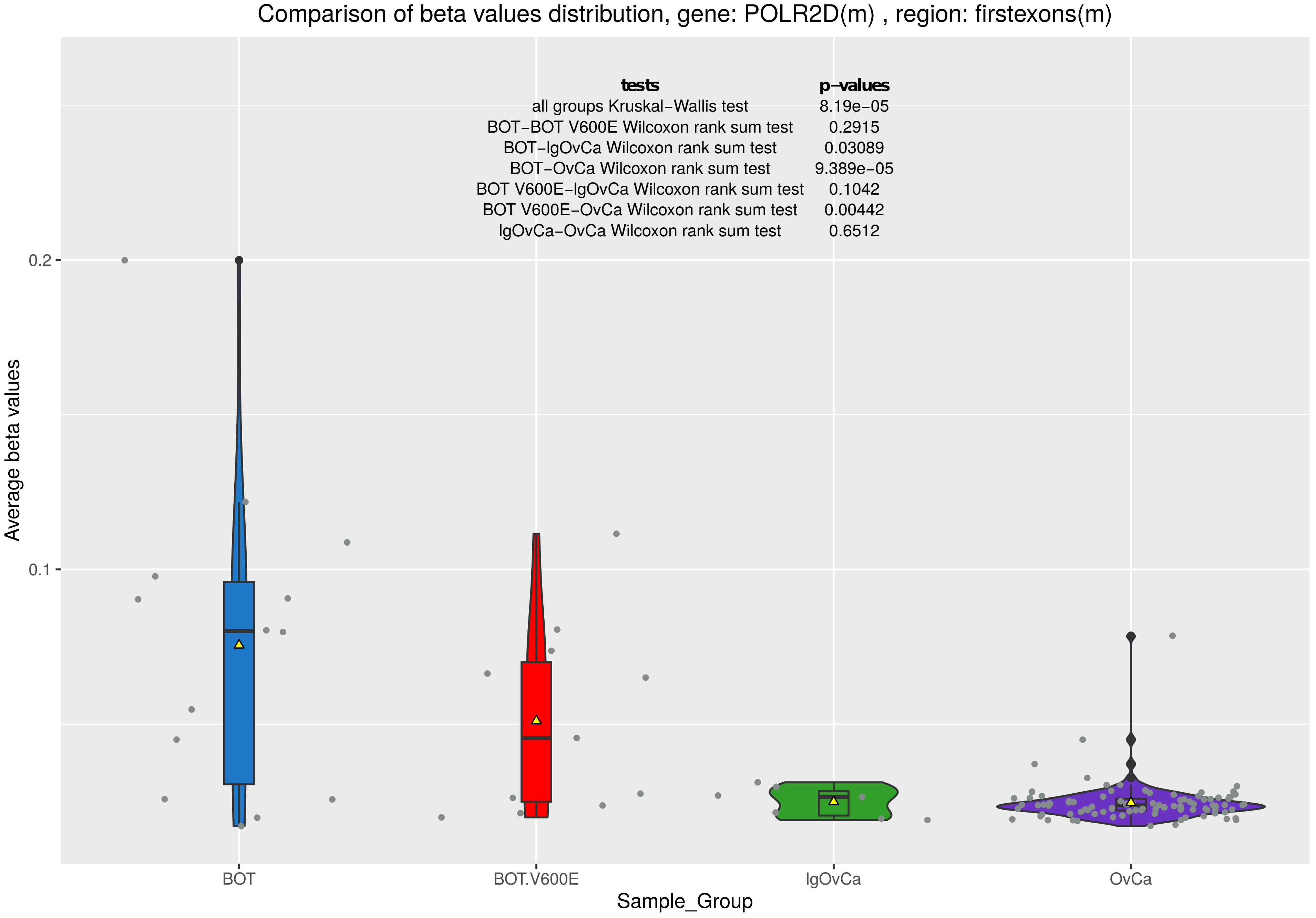

Comparison of beta values distribution, gene: POLR2D(m) , region: firstexons(m)
tests
p−values
all groups Kruskal−Wallis test
8.19e−05
BOT−BOT V600E Wilcoxon rank sum test
0.2915
BOT−lgOvCa Wilcoxon rank sum test
0.03089
BOT−OvCa Wilcoxon rank sum test
9.389e−05
BOT V600E−lgOvCa Wilcoxon rank sum test
0.1042
BOT V600E−OvCa Wilcoxon rank sum test
0.00442
lgOvCa−OvCa Wilcoxon rank sum test
0.6512
Average beta values
0.2
0.1
BOT
BOT.V600E
lgOvCa
OvCa
Sample_Group

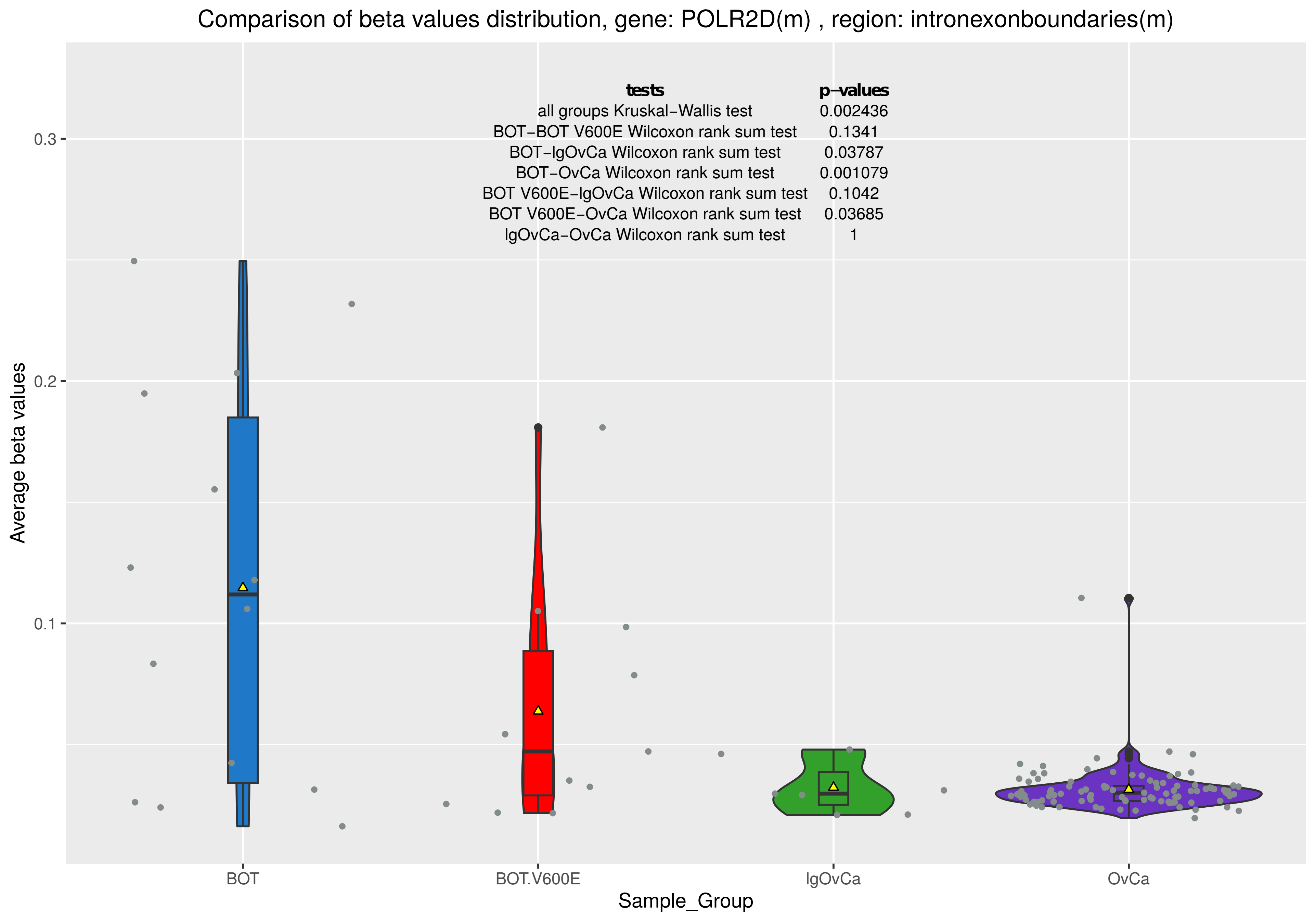

Comparison of beta values distribution, gene: POLR2D(m) , region: intronexonboundaries(m)
tests
p−values
all groups Kruskal−Wallis test
0.002436
BOT−BOT V600E Wilcoxon rank sum test
0.1341
BOT−lgOvCa Wilcoxon rank sum test
0.03787
BOT−OvCa Wilcoxon rank sum test
0.001079
BOT V600E−lgOvCa Wilcoxon rank sum test
0.1042
BOT V600E−OvCa Wilcoxon rank sum test
0.03685
lgOvCa−OvCa Wilcoxon rank sum test
1
Average beta values
0.3
0.2
0.1
BOT
BOT.V600E
lgOvCa
OvCa
Sample_Group

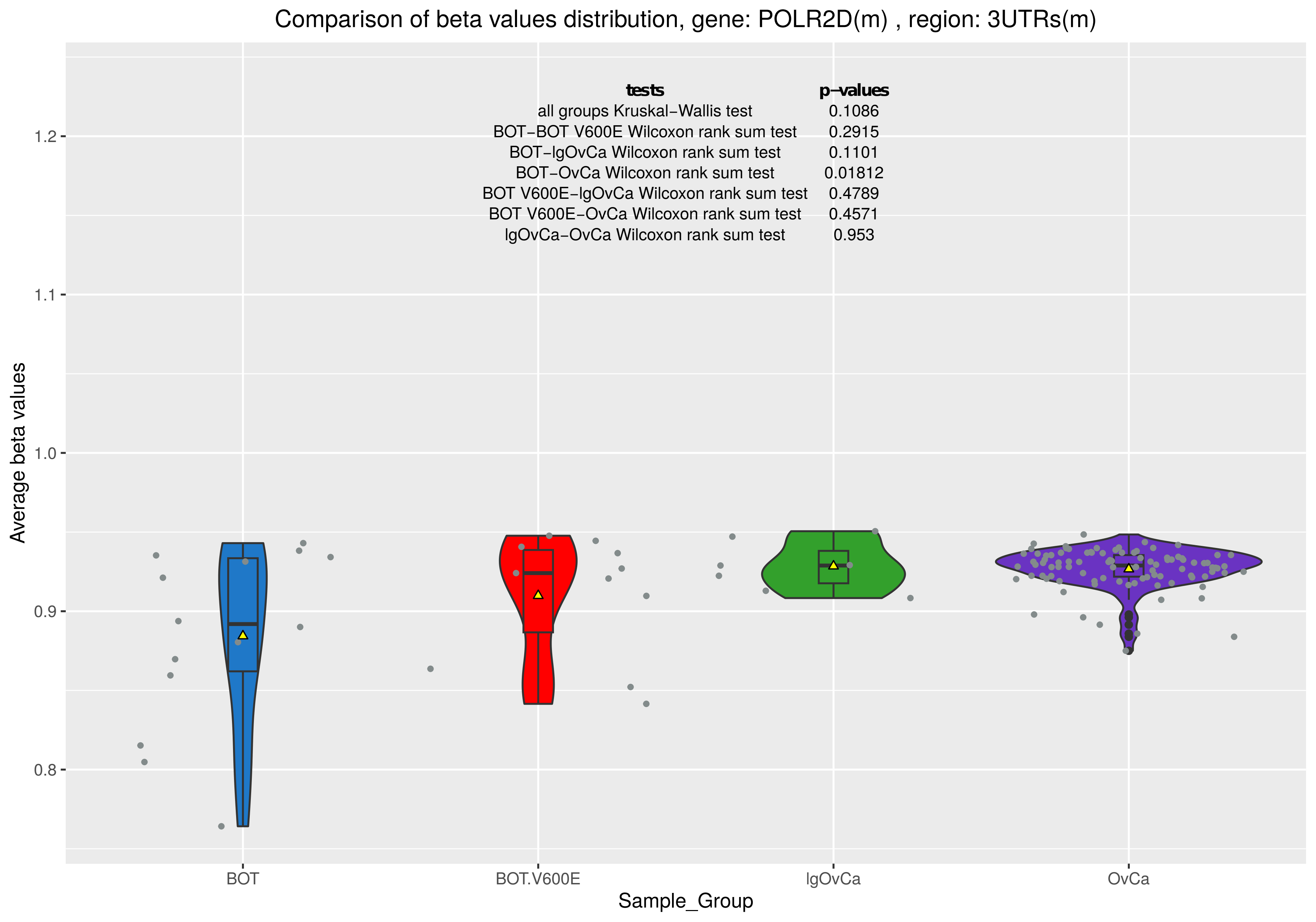

Comparison of beta values distribution, gene: POLR2D(m) , region: 3UTRs(m)
tests
p−values
all groups Kruskal−Wallis test
0.1086
BOT−BOT V600E Wilcoxon rank sum test
0.2915
BOT−lgOvCa Wilcoxon rank sum test
0.1101
BOT−OvCa Wilcoxon rank sum test
0.01812
BOT V600E−lgOvCa Wilcoxon rank sum test
0.4789
BOT V600E−OvCa Wilcoxon rank sum test
0.4571
lgOvCa−OvCa Wilcoxon rank sum test
0.953
Average beta values
1.2
1.1
1.0
0.9
0.8
BOT
BOT.V600E
lgOvCa
OvCa
Sample_Group

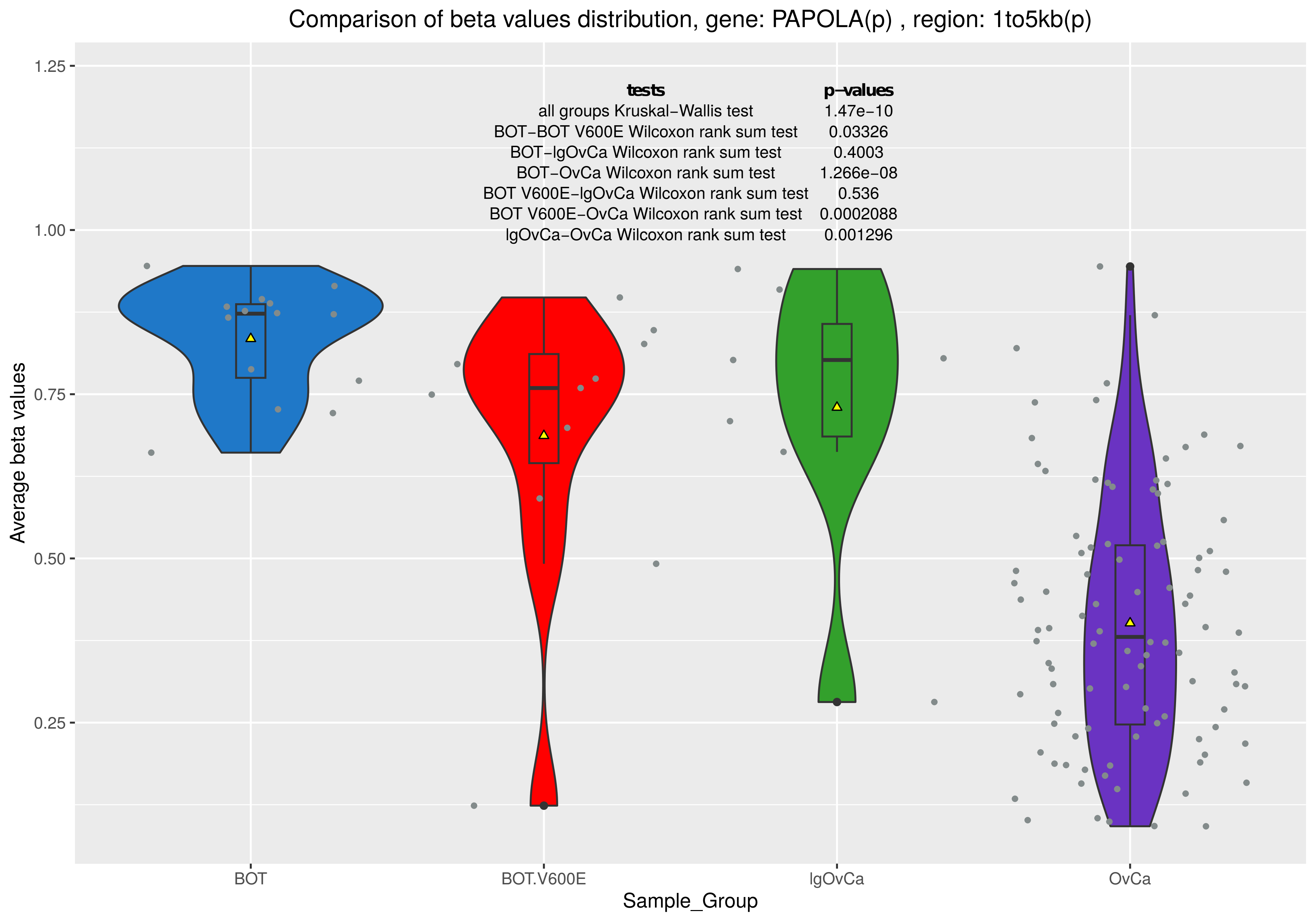
Comparison of beta values distribution, gene: PAPOLA(p) , region: 1to5kb(p)
tests
p−values
all groups Kruskal−Wallis test
1.47e−10
BOT−BOT V600E Wilcoxon rank sum test
0.03326
BOT−lgOvCa Wilcoxon rank sum test
0.4003
BOT−OvCa Wilcoxon rank sum test
1.266e−08
BOT V600E−lgOvCa Wilcoxon rank sum test
0.536
BOT V600E−OvCa Wilcoxon rank sum test
0.0002088
lgOvCa−OvCa Wilcoxon rank sum test
0.001296
Average beta values
1.25
1.00
0.75
0.50
0.25
BOT
BOT.V600E
lgOvCa
OvCa
Sample_Group

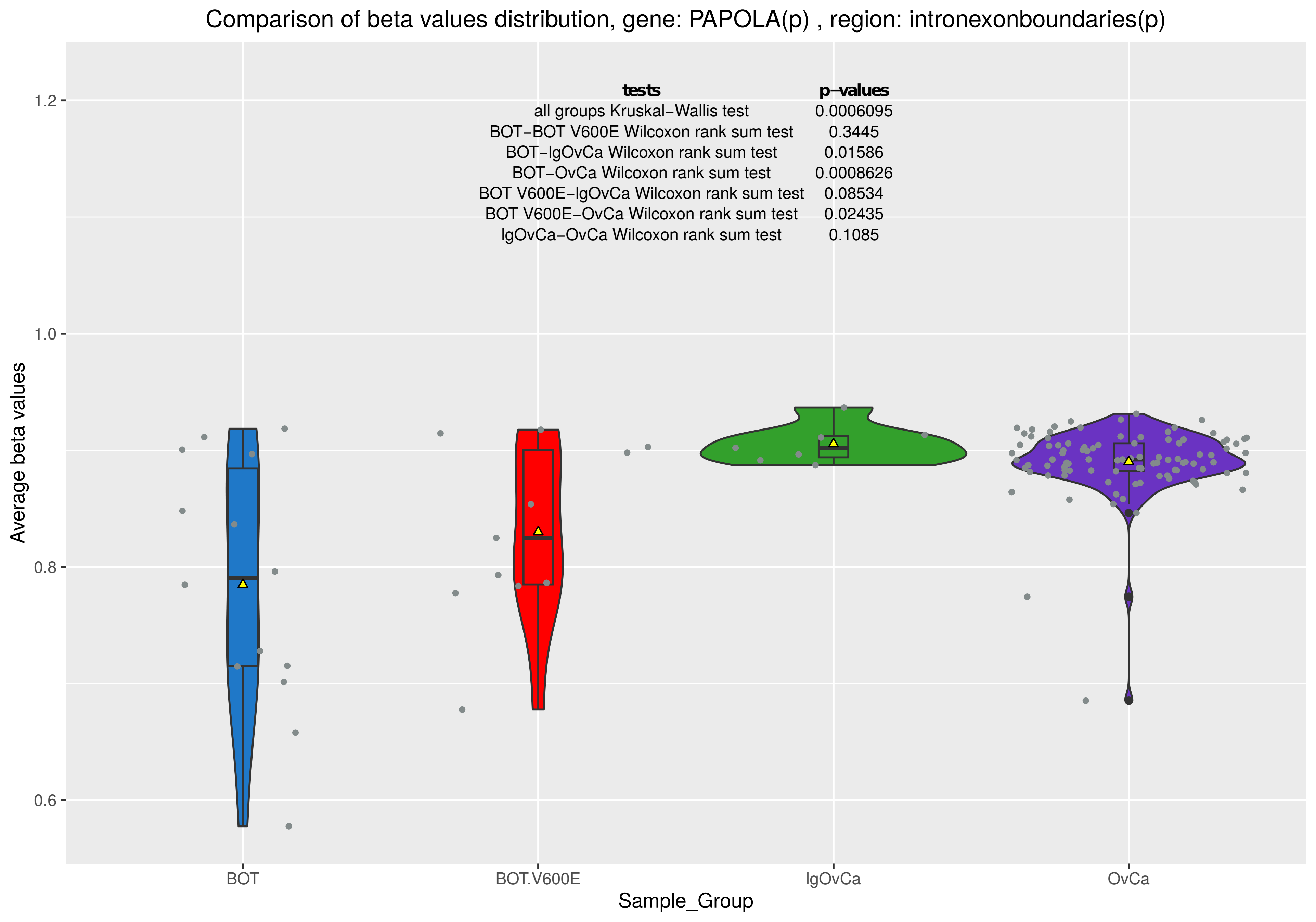

Comparison of beta values distribution, gene: PAPOLA(p) , region: intronexonboundaries(p)
tests
p−values
all groups Kruskal−Wallis test 0.0006095
BOT−BOT V600E Wilcoxon rank sum test 0.3445
BOT−lgOvCa Wilcoxon rank sum test 0.01586
BOT−OvCa Wilcoxon rank sum test 0.0008626
BOT V600E−lgOvCa Wilcoxon rank sum test 0.08534
BOT V600E−OvCa Wilcoxon rank sum test 0.02435
lgOvCa−OvCa Wilcoxon rank sum test 0.1085
Average beta values
1.2
1.0
0.8
0.6
BOT
BOT.V600E
lgOvCa
OvCa
Sample_Group

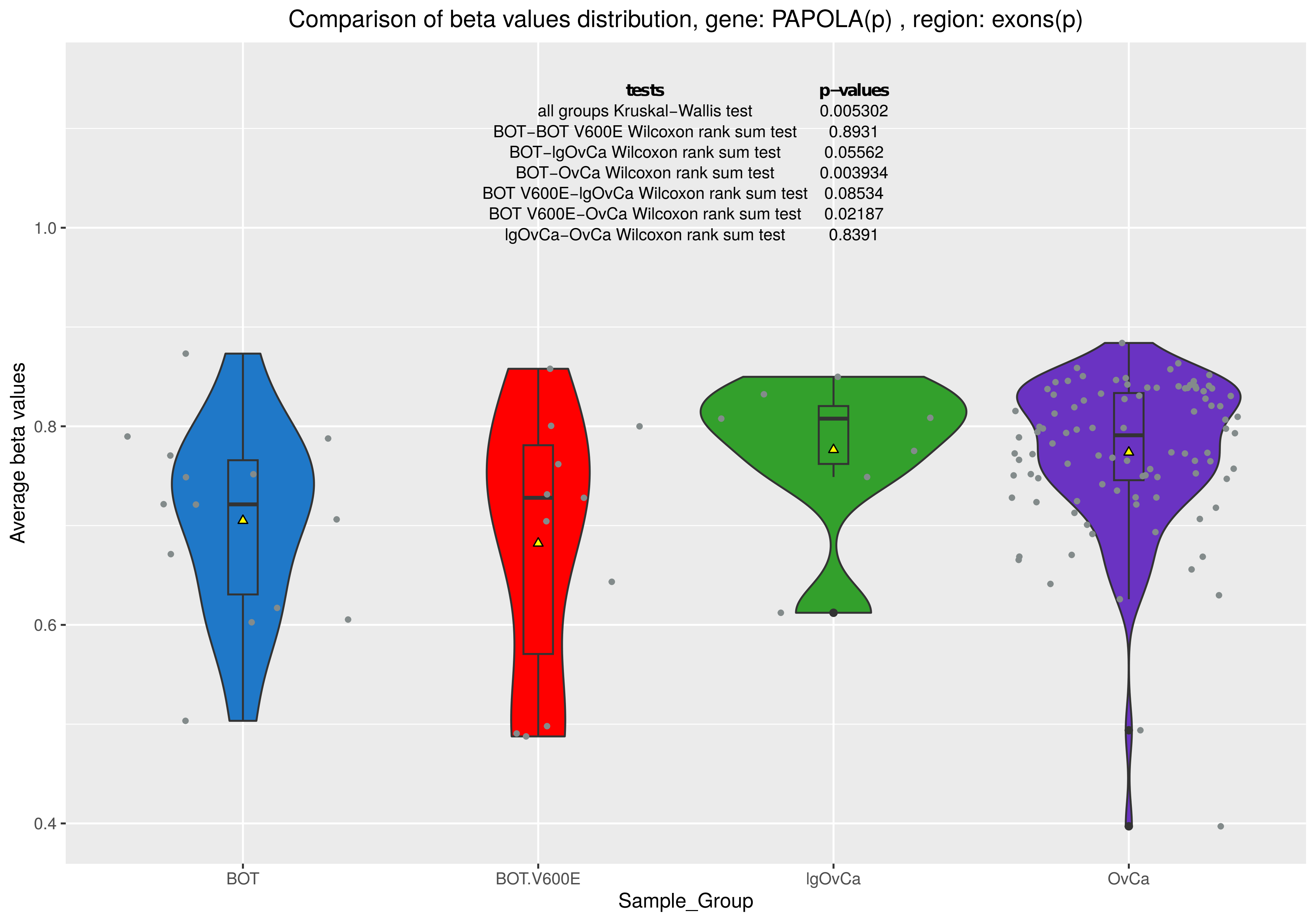

Comparison of beta values distribution, gene: PAPOLA(p) , region: exons(p)
tests
p−values
all groups Kruskal−Wallis test
0.005302
BOT−BOT V600E Wilcoxon rank sum test
0.8931
BOT−lgOvCa Wilcoxon rank sum test
0.05562
BOT−OvCa Wilcoxon rank sum test
0.003934
BOT V600E−lgOvCa Wilcoxon rank sum test
0.08534
BOT V600E−OvCa Wilcoxon rank sum test
0.02187
lgOvCa−OvCa Wilcoxon rank sum test
0.8391
Average beta values
1.0
0.8
0.6
0.4
BOT
BOT.V600E
lgOvCa
OvCa
Sample_Group

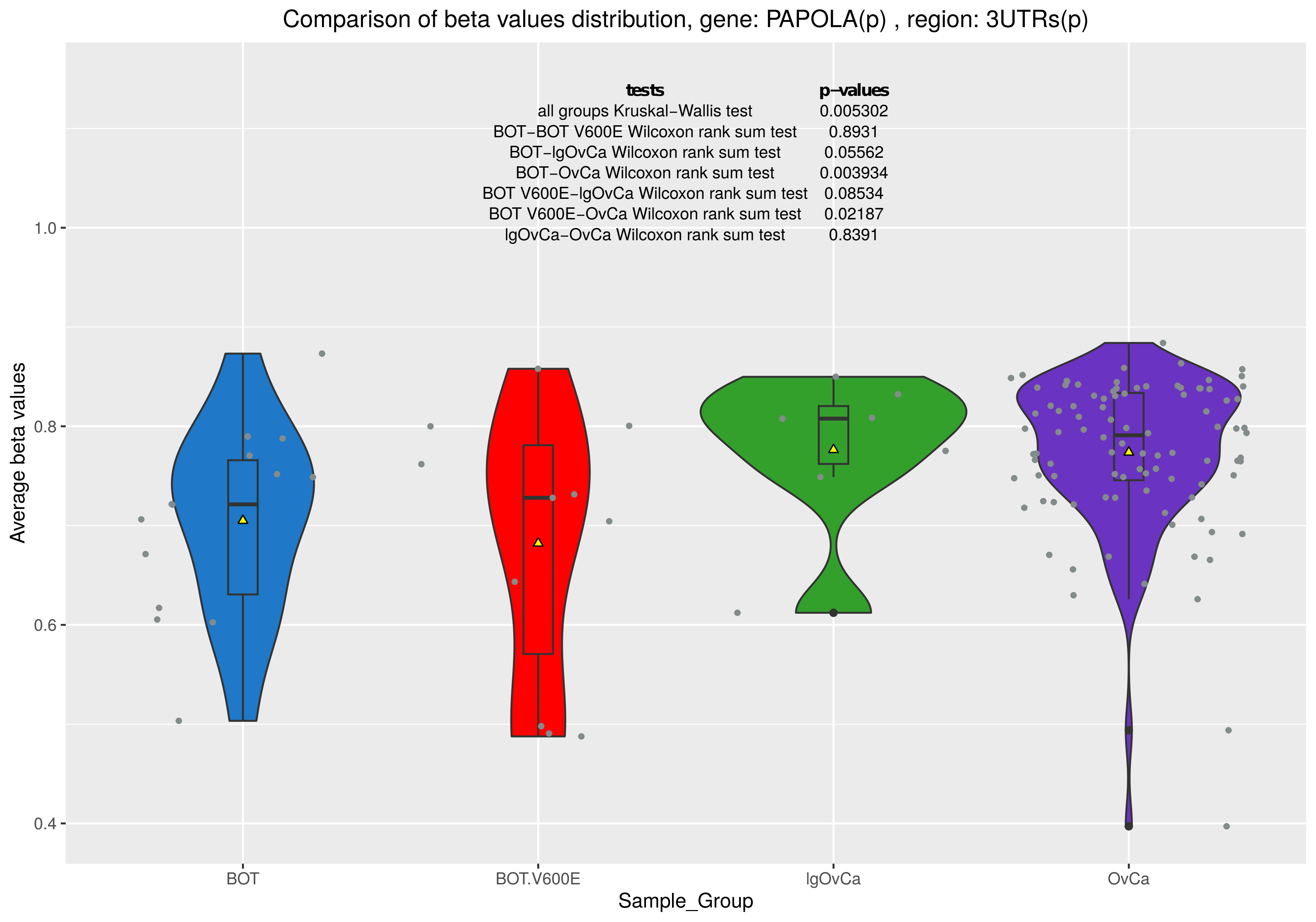

Comparison of beta values distribution, gene: PAPOLA(p) , region: 3UTRs(p)
tests
p−values
all groups Kruskal−Wallis test
0.005302
BOT−BOT V600E Wilcoxon rank sum test
0.8931
BOT−lgOvCa Wilcoxon rank sum test
0.05562
BOT−OvCa Wilcoxon rank sum test
0.003934
BOT V600E−lgOvCa Wilcoxon rank sum test
0.08534
BOT V600E−OvCa Wilcoxon rank sum test
0.02187
lgOvCa−OvCa Wilcoxon rank sum test
0.8391
Average beta values
1.0
0.8
0.6
0.4
BOT
BOT.V600E
lgOvCa
OvCa
Sample_Group

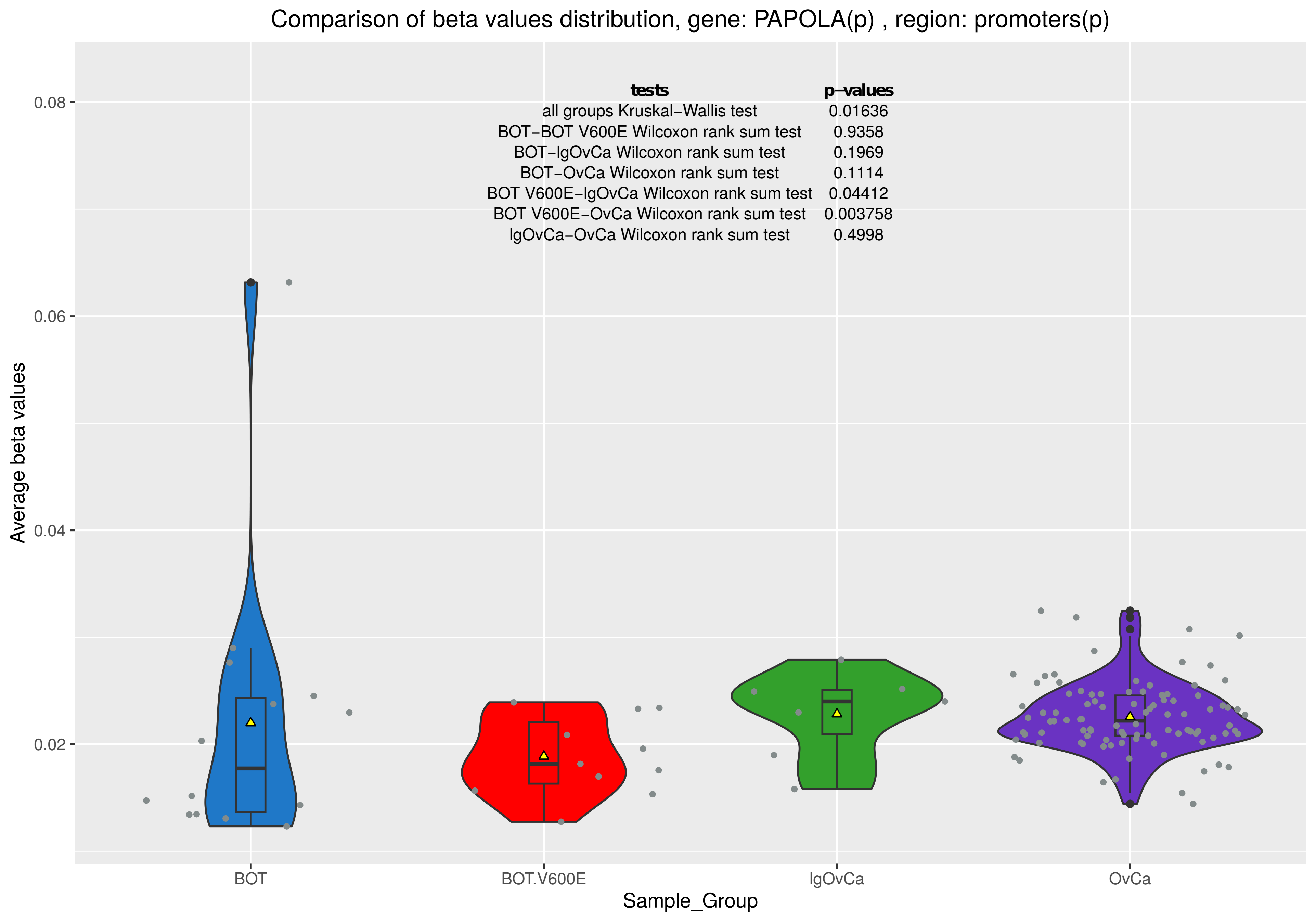
Comparison of beta values distribution, gene: PAPOLA(p) , region: promoters(p)
tests
p−values
all groups Kruskal−Wallis test
0.01636
BOT−BOT V600E Wilcoxon rank sum test
0.9358
BOT−lgOvCa Wilcoxon rank sum test
0.1969
BOT−OvCa Wilcoxon rank sum test
0.1114
BOT V600E−lgOvCa Wilcoxon rank sum test
0.04412
BOT V600E−OvCa Wilcoxon rank sum test
0.003758
lgOvCa−OvCa Wilcoxon rank sum test
0.4998
Average beta values
0.08
0.06
0.04
0.02
BOT
BOT.V600E
lgOvCa
OvCa
Sample_Group

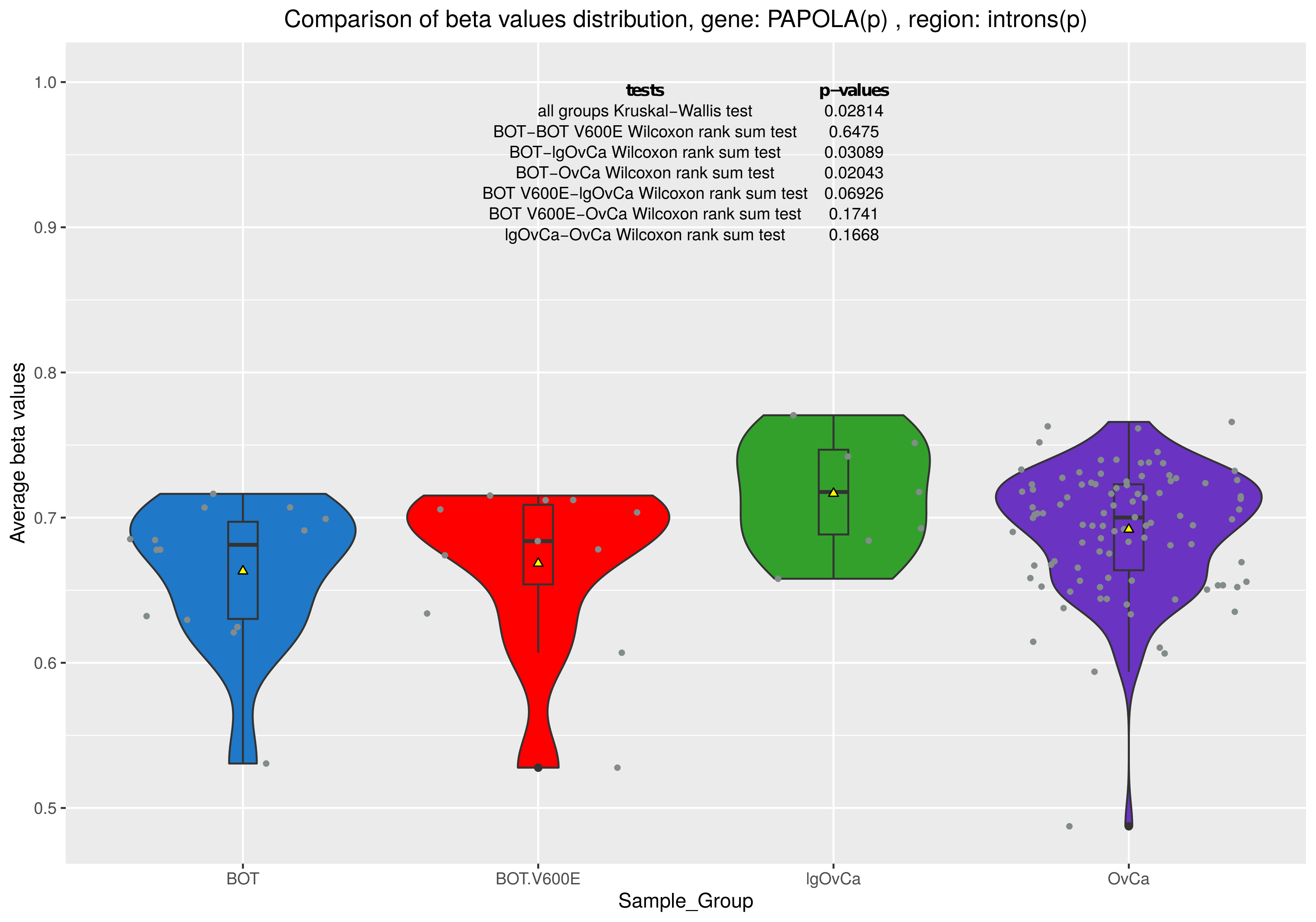
Comparison of beta values distribution, gene: PAPOLA(p) , region: introns(p)
tests
p−values
all groups Kruskal−Wallis test
0.02814
BOT−BOT V600E Wilcoxon rank sum test
0.6475
BOT−lgOvCa Wilcoxon rank sum test
0.03089
BOT−OvCa Wilcoxon rank sum test
0.02043
BOT V600E−lgOvCa Wilcoxon rank sum test
0.06926
BOT V600E−OvCa Wilcoxon rank sum test
0.1741
lgOvCa−OvCa Wilcoxon rank sum test
0.1668
Average beta values
1.0
0.9
0.8
0.7
0.6
0.5
BOT
BOT.V600E
lgOvCa
OvCa
Sample_Group

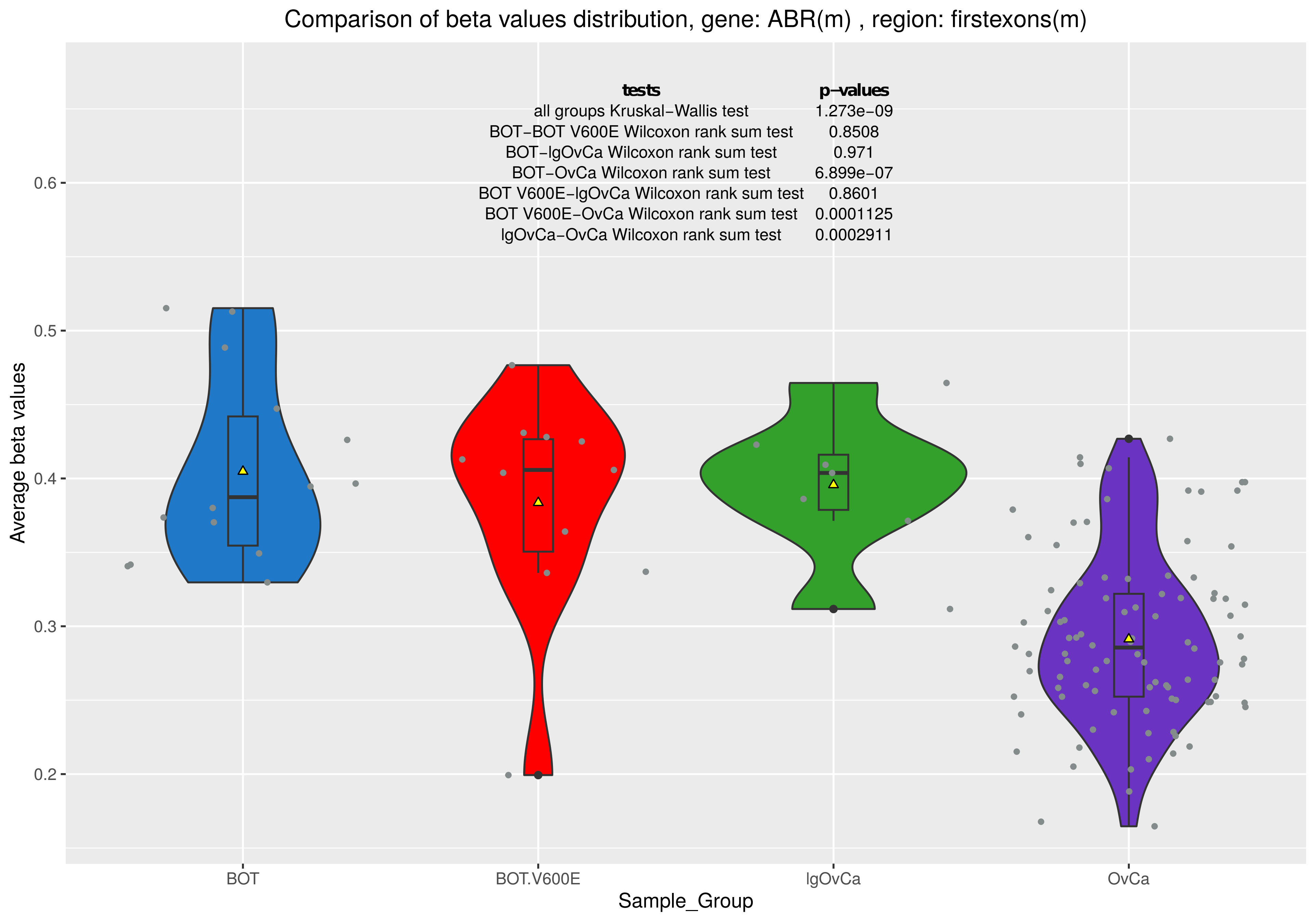

Comparison of beta values distribution, gene: ABR(m) , region: firstexons(m)
tests
p−values
all groups Kruskal−Wallis test
1.273e−09
BOT−BOT V600E Wilcoxon rank sum test
0.8508
BOT−lgOvCa Wilcoxon rank sum test
0.971
BOT−OvCa Wilcoxon rank sum test
6.899e−07
BOT V600E−lgOvCa Wilcoxon rank sum test
0.8601
BOT V600E−OvCa Wilcoxon rank sum test
0.0001125
lgOvCa−OvCa Wilcoxon rank sum test
0.0002911
Average beta values
0.6
0.5
0.4
0.3
0.2
BOT
BOT.V600E
lgOvCa
OvCa
Sample_Group

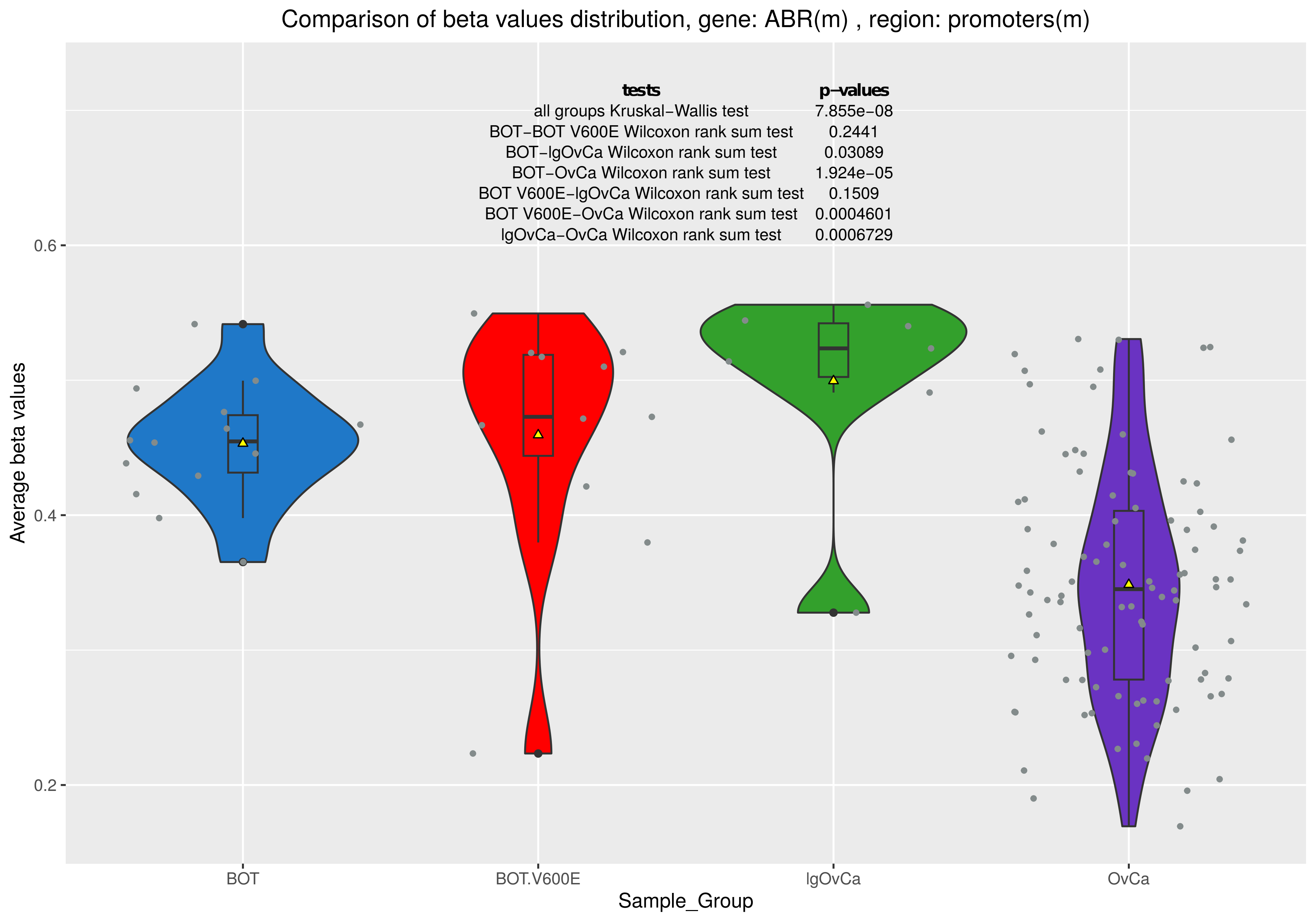

Comparison of beta values distribution, gene: ABR(m) , region: promoters(m)
tests
p−values
all groups Kruskal−Wallis test
7.855e−08
BOT−BOT V600E Wilcoxon rank sum test
0.2441
BOT−lgOvCa Wilcoxon rank sum test
0.03089
BOT−OvCa Wilcoxon rank sum test
1.924e−05
BOT V600E−lgOvCa Wilcoxon rank sum test
0.1509
BOT V600E−OvCa Wilcoxon rank sum test
0.0004601
lgOvCa−OvCa Wilcoxon rank sum test
0.0006729
Average beta values
0.6
0.4
0.2
BOT
BOT.V600E
lgOvCa
OvCa
Sample_Group

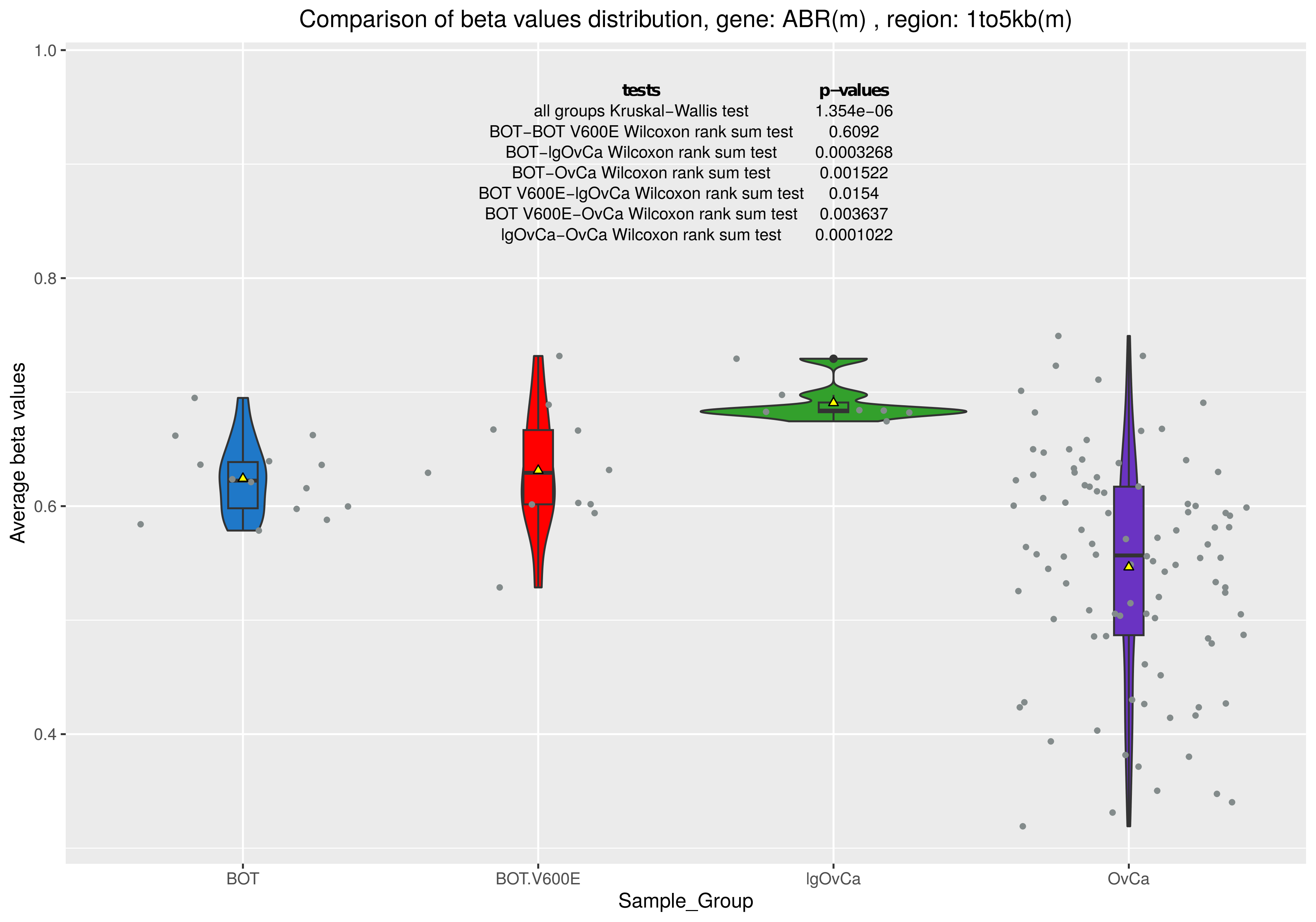

Comparison of beta values distribution, gene: ABR(m) , region: 1to5kb(m)
tests
p−values
all groups Kruskal−Wallis test
1.354e−06
BOT−BOT V600E Wilcoxon rank sum test
0.6092
BOT−IgOvCa Wilcoxon rank sum test
0.0003268
BOT−OvCa Wilcoxon rank sum test
0.001522
BOT V600E−IgOvCa Wilcoxon rank sum test
0.0154
BOT V600E−OvCa Wilcoxon rank sum test
0.003637
IgOvCa−OvCa Wilcoxon rank sum test
0.0001022
Average beta values
1.0
0.8
0.6
0.4
BOT
BOT.V600E
IgOvCa
OvCa
Sample_Group

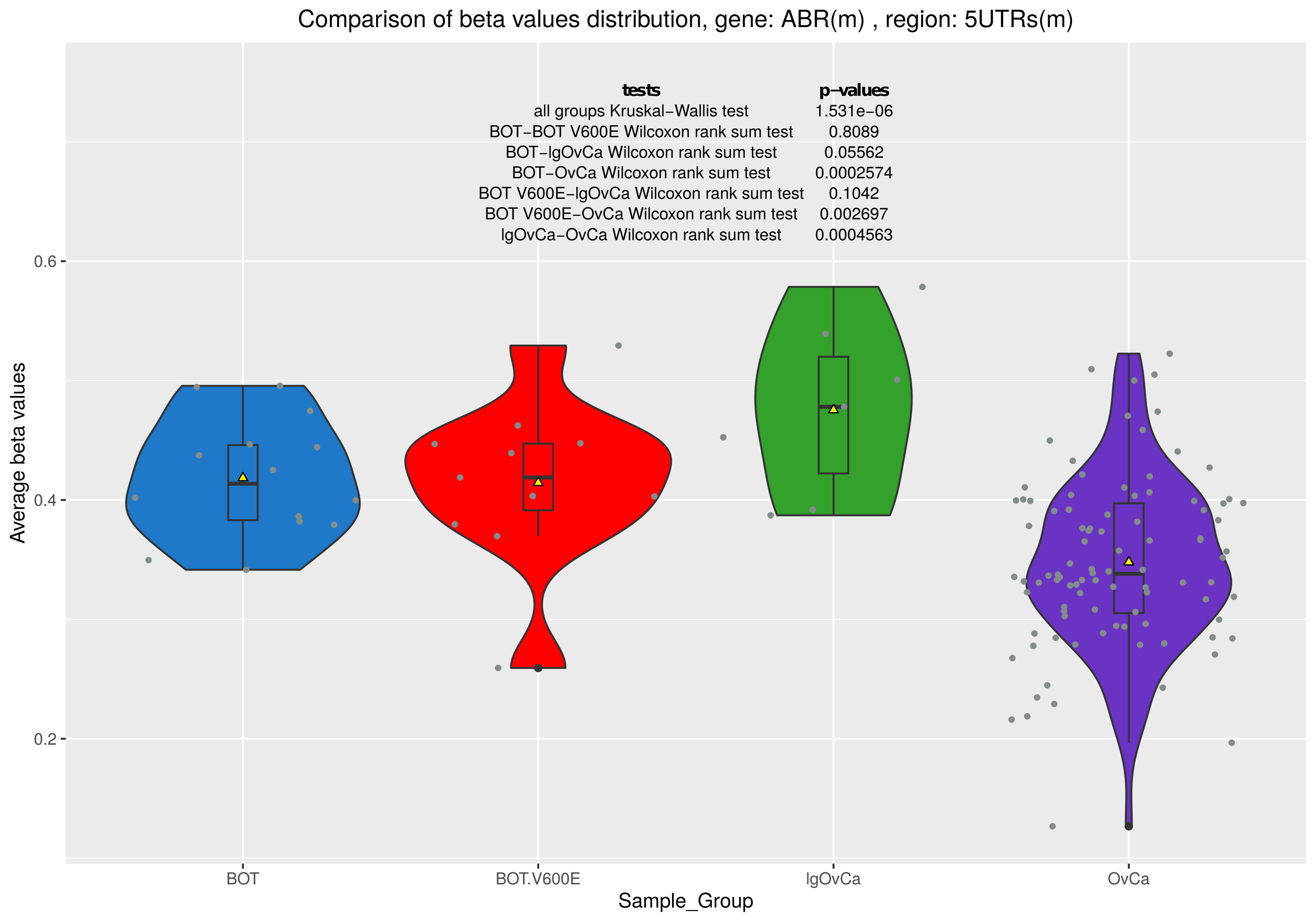

Comparison of beta values distribution, gene: ABR(m) , region: 5UTRs(m)
tests
p−values
all groups Kruskal−Wallis test
1.531e−06
BOT−BOT V600E Wilcoxon rank sum test
0.8089
BOT−lgOvCa Wilcoxon rank sum test
0.05562
BOT−OvCa Wilcoxon rank sum test
0.0002574
BOT V600E−lgOvCa Wilcoxon rank sum test
0.1042
BOT V600E−OvCa Wilcoxon rank sum test
0.002697
lgOvCa−OvCa Wilcoxon rank sum test
0.0004563
Average beta values
0.6
0.4
0.2
BOT
BOT.V600E
lgOvCa
OvCa
Sample_Group

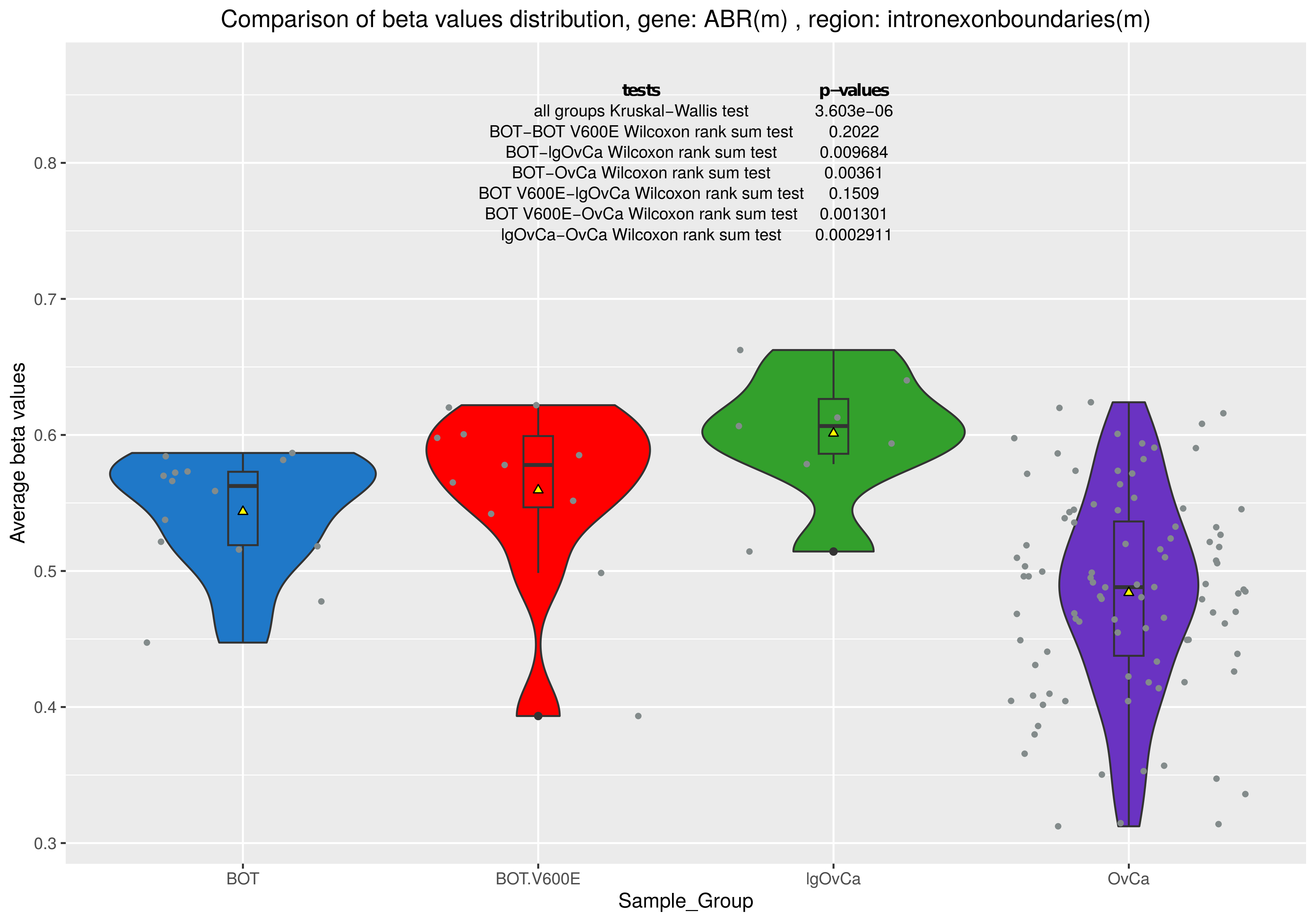

Comparison of beta values distribution, gene: ABR(m) , region: intronexonboundaries(m)
tests
p-values
all groups Kruskal-Wallis test
3.603e-06
BOT-BOT V600E Wilcoxon rank sum test
0.2022
BOT-lgOvCa Wilcoxon rank sum test
0.009684
BOT-OvCa Wilcoxon rank sum test
0.00361
BOT V600E-lgOvCa Wilcoxon rank sum test
0.1509
BOT V600E-OvCa Wilcoxon rank sum test
0.001301
lgOvCa-OvCa Wilcoxon rank sum test
0.0002911
Average beta values
0.8
0.7
0.6
0.5
0.4
0.3
BOT
BOT.V600E
lgOvCa
OvCa
Sample_Group

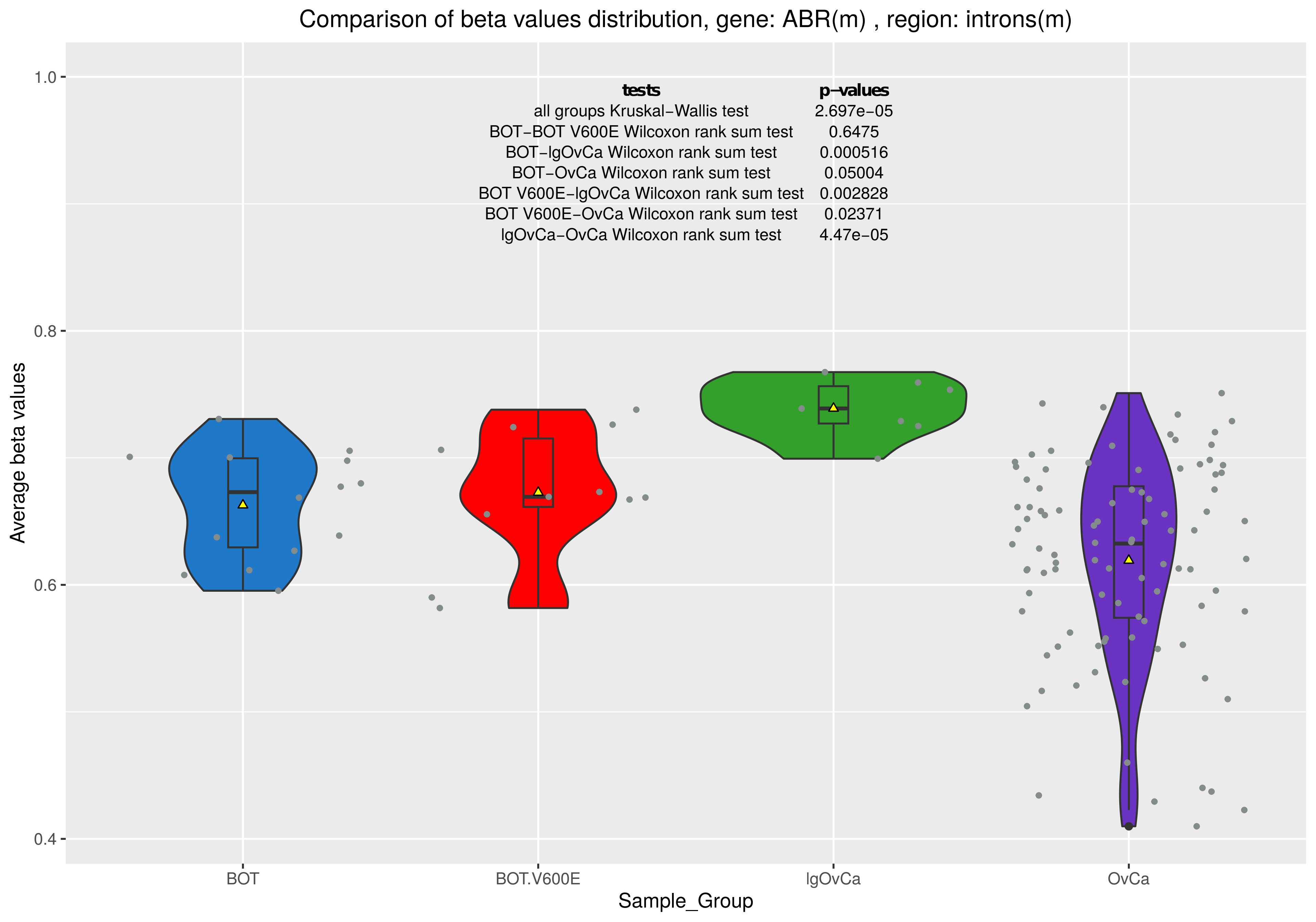
Comparison of beta values distribution, gene: ABR(m) , region: introns(m)
tests
p−values
all groups Kruskal−Wallis test
2.697e−05
BOT−BOT V600E Wilcoxon rank sum test
0.6475
BOT−IgOvCa Wilcoxon rank sum test
0.000516
BOT−OvCa Wilcoxon rank sum test
0.05004
BOT V600E−IgOvCa Wilcoxon rank sum test
0.002828
BOT V600E−OvCa Wilcoxon rank sum test
0.02371
IgOvCa−OvCa Wilcoxon rank sum test
4.47e−05
Average beta values
1.0
0.8
0.6
0.4
BOT
BOT.V600E
IgOvCa
OvCa
Sample_Group

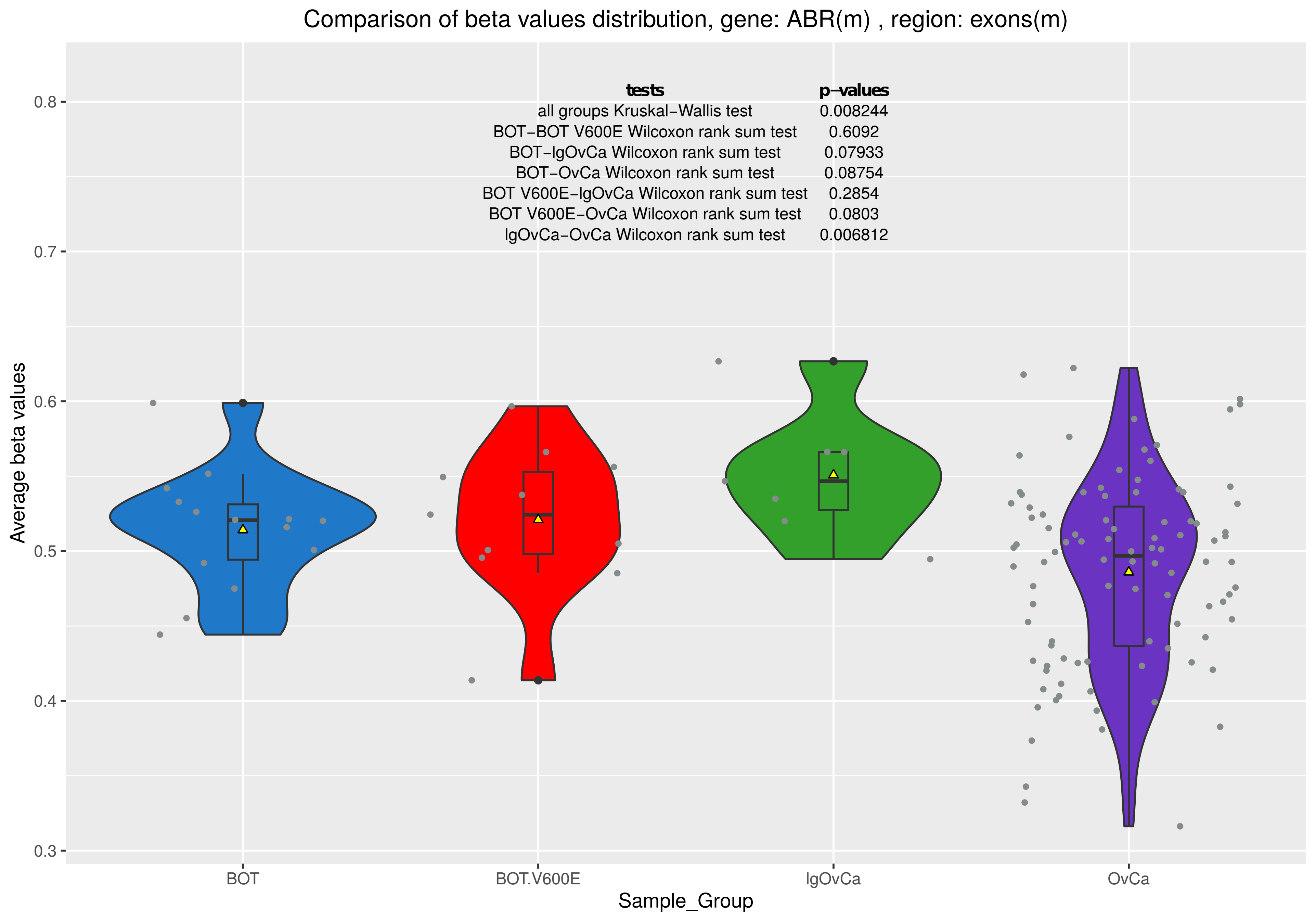
Comparison of beta values distribution, gene: ABR(m) , region: exons(m)
tests
p−values
all groups Kruskal−Wallis test
0.008244
BOT−BOT V600E Wilcoxon rank sum test
0.6092
BOT−lgOvCa Wilcoxon rank sum test
0.07933
BOT−OvCa Wilcoxon rank sum test
0.08754
BOT V600E−lgOvCa Wilcoxon rank sum test
0.2854
BOT V600E−OvCa Wilcoxon rank sum test
0.0803
lgOvCa−OvCa Wilcoxon rank sum test
0.006812
Average beta values
0.8
0.7
0.6
0.5
0.4
0.3
BOT
BOT.V600E
lgOvCa
OvCa
Sample_Group

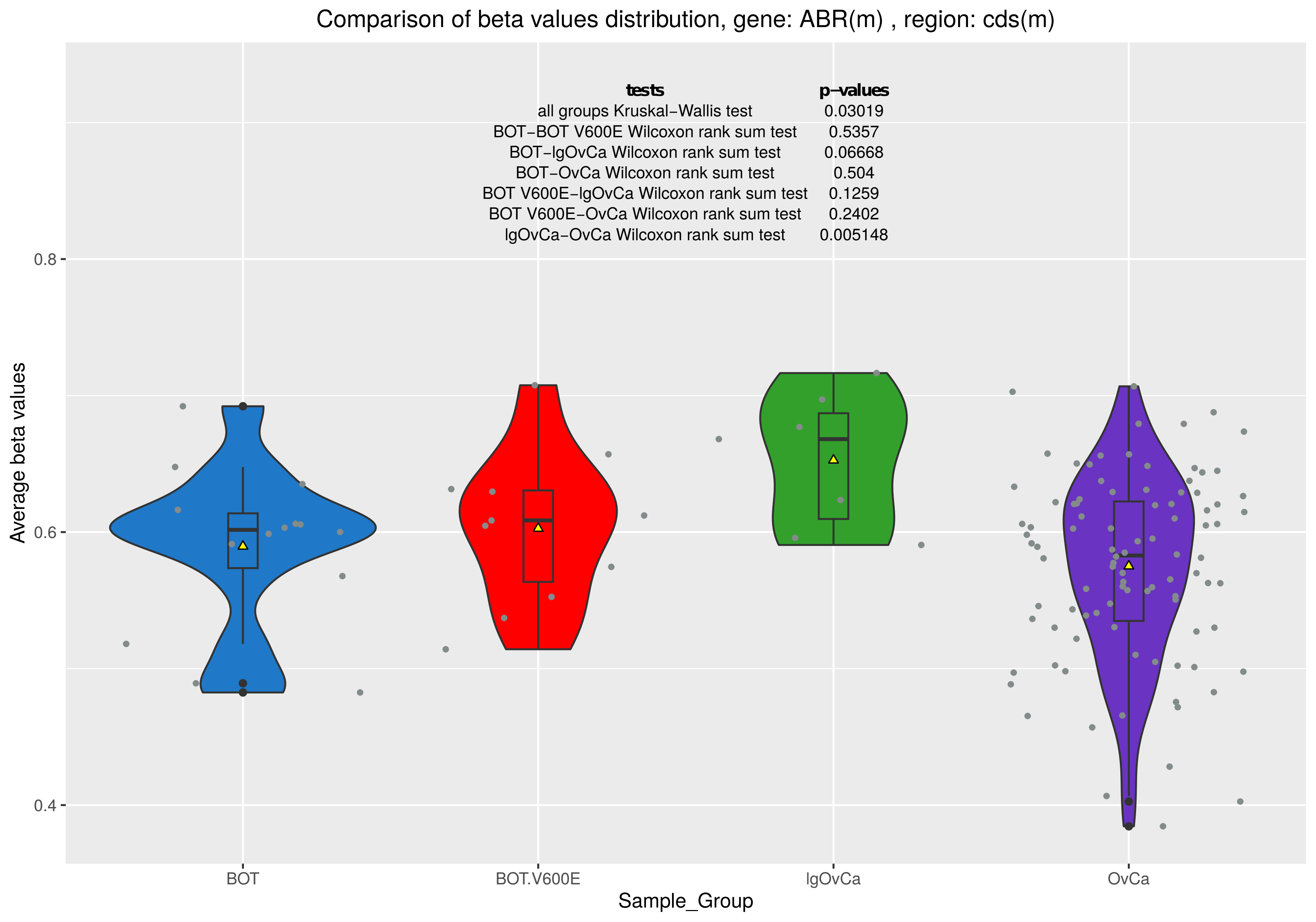
Comparison of beta values distribution, gene: ABR(m) , region: cds(m)
tests
p−values
all groups Kruskal−Wallis test
0.03019
BOT−BOT V600E Wilcoxon rank sum test
0.5357
BOT−lgOvCa Wilcoxon rank sum test
0.06668
BOT−OvCa Wilcoxon rank sum test
0.504
BOT V600E−lgOvCa Wilcoxon rank sum test
0.1259
BOT V600E−OvCa Wilcoxon rank sum test
0.2402
lgOvCa−OvCa Wilcoxon rank sum test
0.005148
Average beta values
0.8
0.6
0.4
BOT
BOT.V600E
lgOvCa
OvCa
Sample_Group

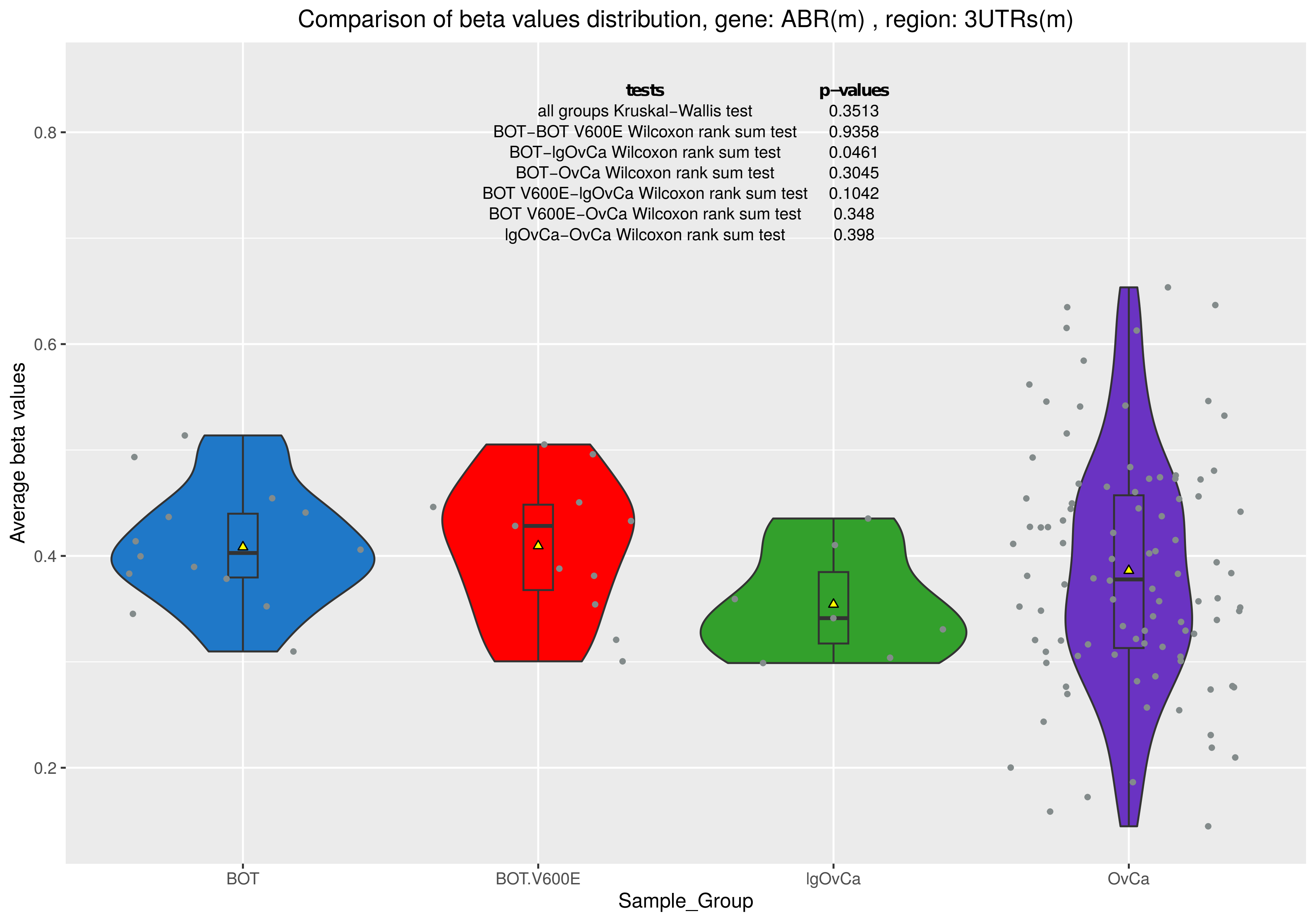

Comparison of beta values distribution, gene: ABR(m) , region: 3UTRs(m)
tests
p−values
all groups Kruskal−Wallis test
0.3513
BOT−BOT V600E Wilcoxon rank sum test
0.9358
BOT−lgOvCa Wilcoxon rank sum test
0.0461
BOT−OvCa Wilcoxon rank sum test
0.3045
BOT V600E−lgOvCa Wilcoxon rank sum test
0.1042
BOT V600E−OvCa Wilcoxon rank sum test
0.348
lgOvCa−OvCa Wilcoxon rank sum test
0.398
Average beta values
0.8
0.6
0.4
0.2
BOT
BOT.V600E
lgOvCa
OvCa
Sample_Group

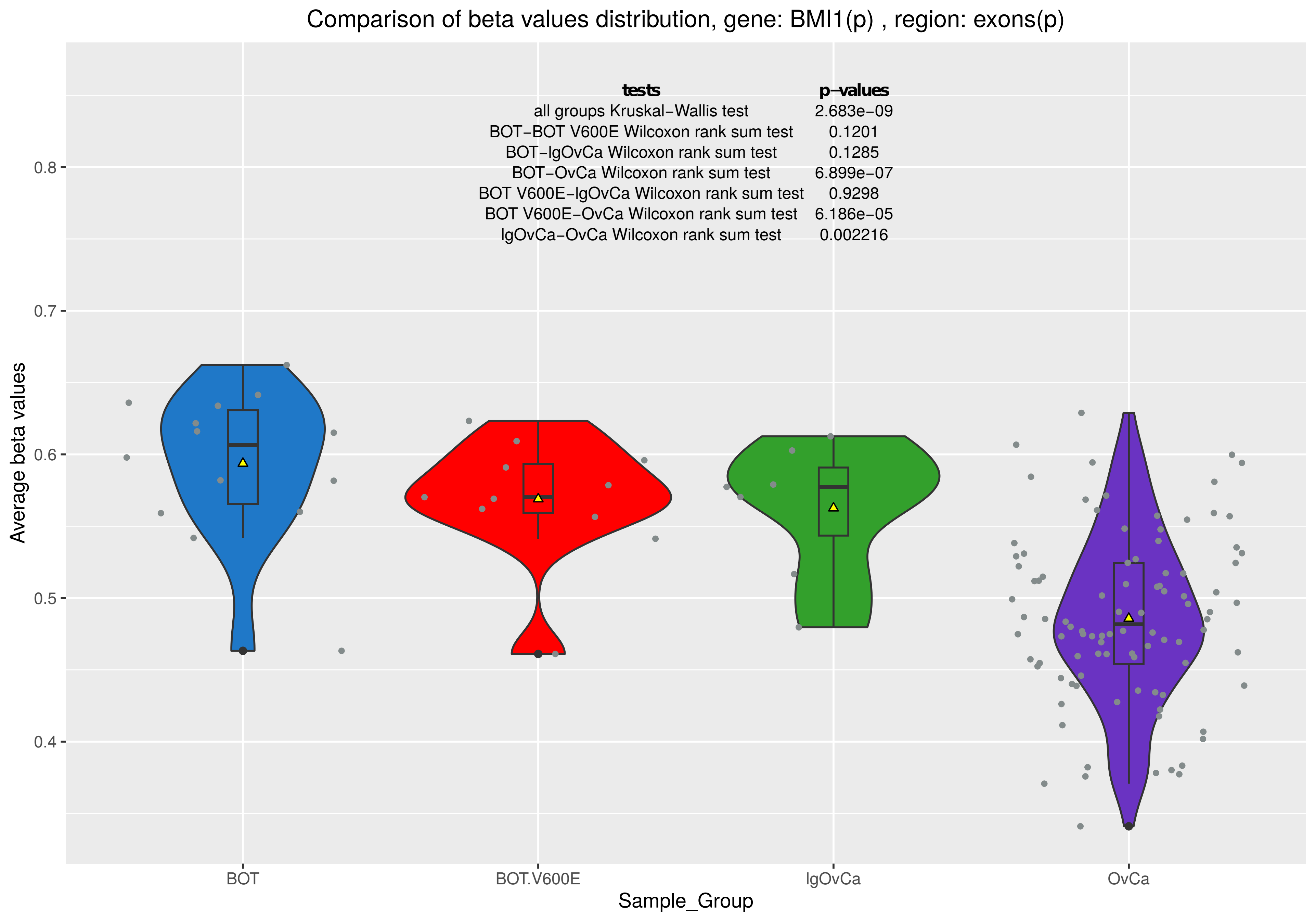

Comparison of beta values distribution, gene: BMI1(p) , region: exons(p)
tests
p−values
all groups Kruskal−Wallis test
2.683e−09
BOT−BOT V600E Wilcoxon rank sum test
0.1201
BOT−lgOvCa Wilcoxon rank sum test
0.1285
BOT−OvCa Wilcoxon rank sum test
6.899e−07
BOT V600E−lgOvCa Wilcoxon rank sum test
0.9298
BOT V600E−OvCa Wilcoxon rank sum test
6.186e−05
lgOvCa−OvCa Wilcoxon rank sum test
0.002216
Average beta values
0.8
0.7
0.6
0.5
0.4
BOT
BOT.V600E
lgOvCa
OvCa
Sample_Group

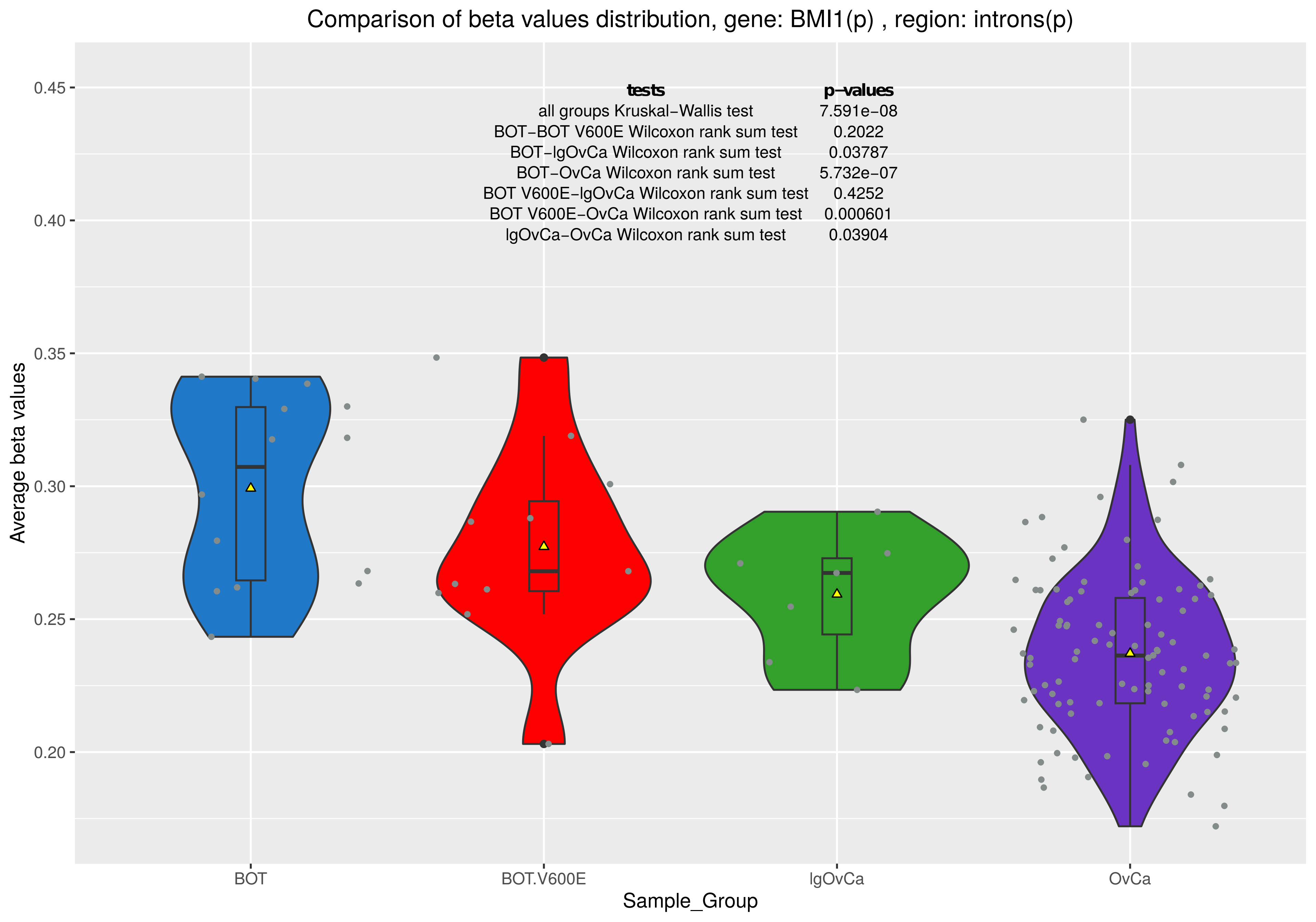
Comparison of beta values distribution, gene: BMI1(p) , region: introns(p)
tests
p−values
all groups Kruskal−Wallis test
7.591e−08
BOT−BOT V600E Wilcoxon rank sum test
0.2022
BOT−lgOvCa Wilcoxon rank sum test
0.03787
BOT−OvCa Wilcoxon rank sum test
5.732e−07
BOT V600E−lgOvCa Wilcoxon rank sum test
0.4252
BOT V600E−OvCa Wilcoxon rank sum test
0.000601
lgOvCa−OvCa Wilcoxon rank sum test
0.03904
Average beta values
0.45
0.40
0.35
0.30
0.25
0.20
BOT
BOT.V600E
lgOvCa
OvCa
Sample_Group

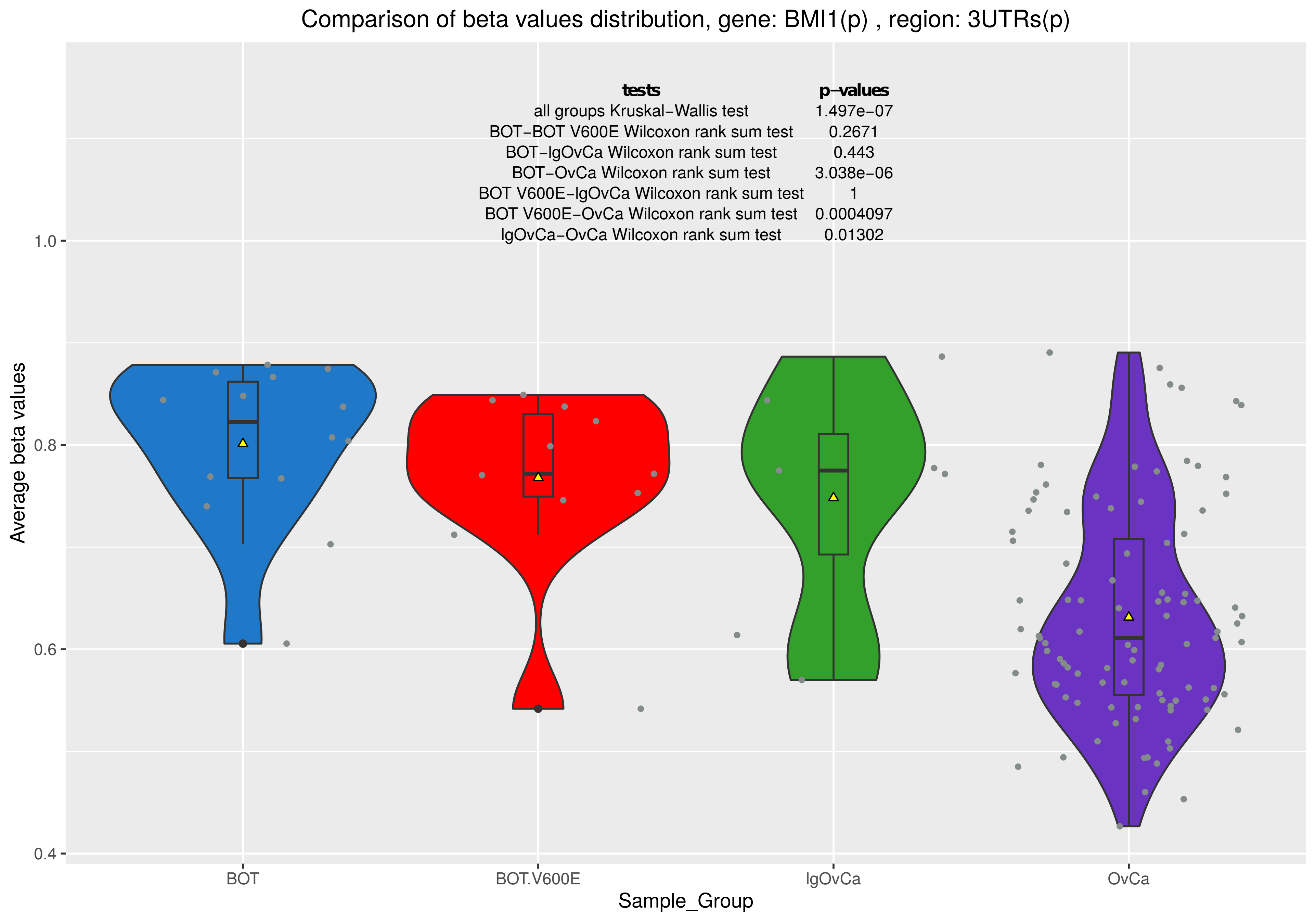
Comparison of beta values distribution, gene: BMI1(p) , region: 3UTRs(p)
tests
p−values
all groups Kruskal−Wallis test
1.497e−07
BOT−BOT V600E Wilcoxon rank sum test
0.2671
BOT−lgOvCa Wilcoxon rank sum test
0.443
BOT−OvCa Wilcoxon rank sum test
3.038e−06
BOT V600E−lgOvCa Wilcoxon rank sum test
1
BOT V600E−OvCa Wilcoxon rank sum test
0.0004097
lgOvCa−OvCa Wilcoxon rank sum test
0.01302
Average beta values
1.0
0.8
0.6
0.4
BOT
BOT.V600E
lgOvCa
OvCa
Sample_Group

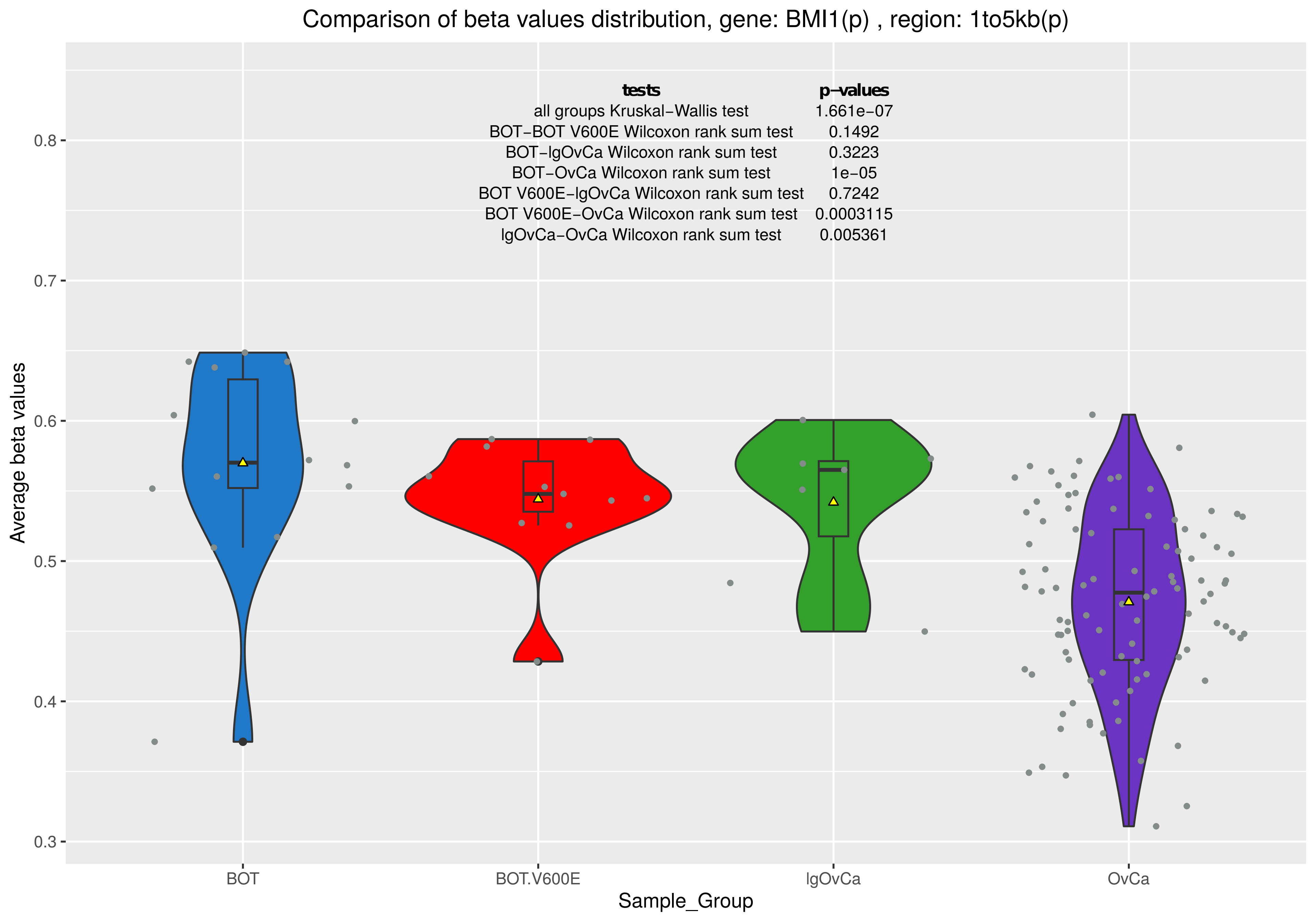

Comparison of beta values distribution, gene: BMI1(p) , region: 1to5kb(p)
tests
p−values
all groups Kruskal−Wallis test
1.661e−07
BOT−BOT V600E Wilcoxon rank sum test
0.1492
BOT−IgOvCa Wilcoxon rank sum test
0.3223
BOT−OvCa Wilcoxon rank sum test
1e−05
BOT V600E−IgOvCa Wilcoxon rank sum test
0.7242
BOT V600E−OvCa Wilcoxon rank sum test
0.0003115
IgOvCa−OvCa Wilcoxon rank sum test
0.005361
Average beta values
0.8
0.7
0.6
0.5
0.4
0.3
BOT
BOT.V600E
IgOvCa
OvCa
Sample_Group

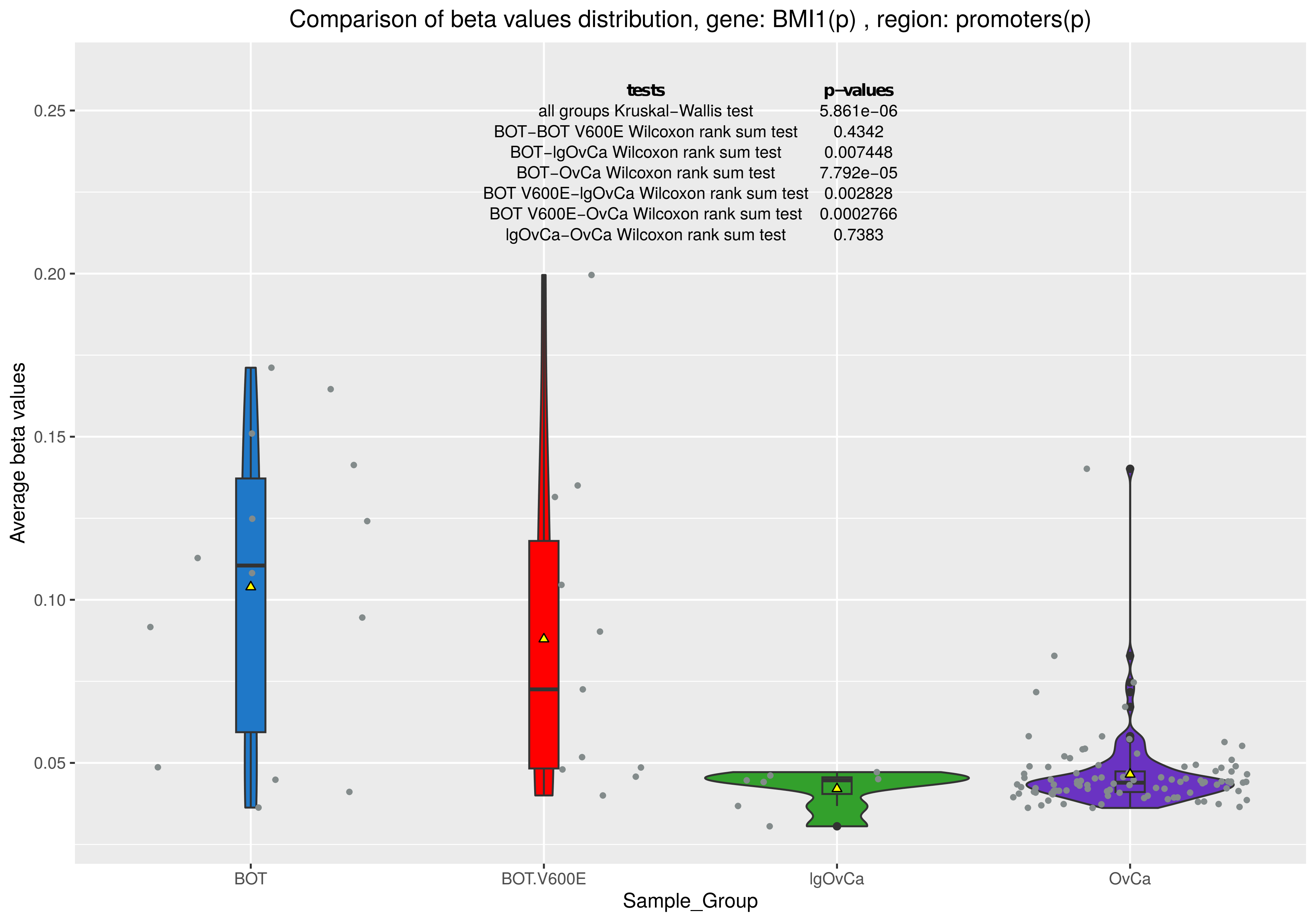
Comparison of beta values distribution, gene: BMI1(p) , region: promoters(p)
tests
p−values
all groups Kruskal−Wallis test 5.861e−06
BOT−BOT V600E Wilcoxon rank sum test 0.4342
BOT−lgOvCa Wilcoxon rank sum test 0.007448
BOT−OvCa Wilcoxon rank sum test 7.792e−05
BOT V600E−lgOvCa Wilcoxon rank sum test 0.002828
BOT V600E−OvCa Wilcoxon rank sum test 0.0002766
lgOvCa−OvCa Wilcoxon rank sum test 0.7383
Average beta values
0.25
0.20
0.15
0.10
0.05
BOT
BOT.V600E
lgOvCa
OvCa
Sample_Group

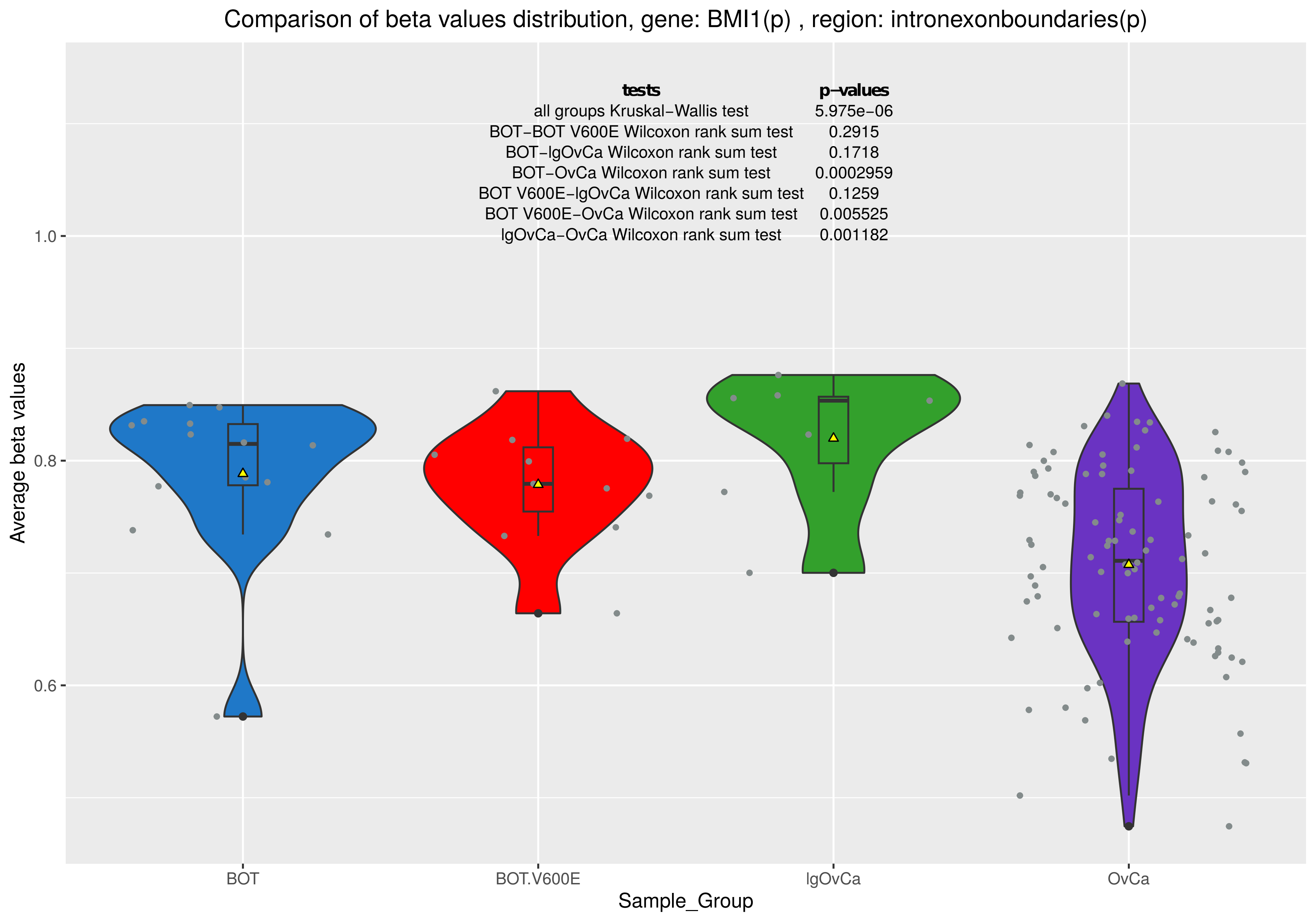

Comparison of beta values distribution, gene: BMI1(p) , region: intronexonboundaries(p)
tests
p−values
all groups Kruskal−Wallis test
5.975e−06
BOT−BOT V600E Wilcoxon rank sum test
0.2915
BOT−lgOvCa Wilcoxon rank sum test
0.1718
BOT−OvCa Wilcoxon rank sum test
0.0002959
BOT V600E−lgOvCa Wilcoxon rank sum test
0.1259
BOT V600E−OvCa Wilcoxon rank sum test
0.005525
lgOvCa−OvCa Wilcoxon rank sum test
0.001182
Average beta values
1.0
0.8
0.6
BOT
BOT.V600E
lgOvCa
OvCa
Sample_Group

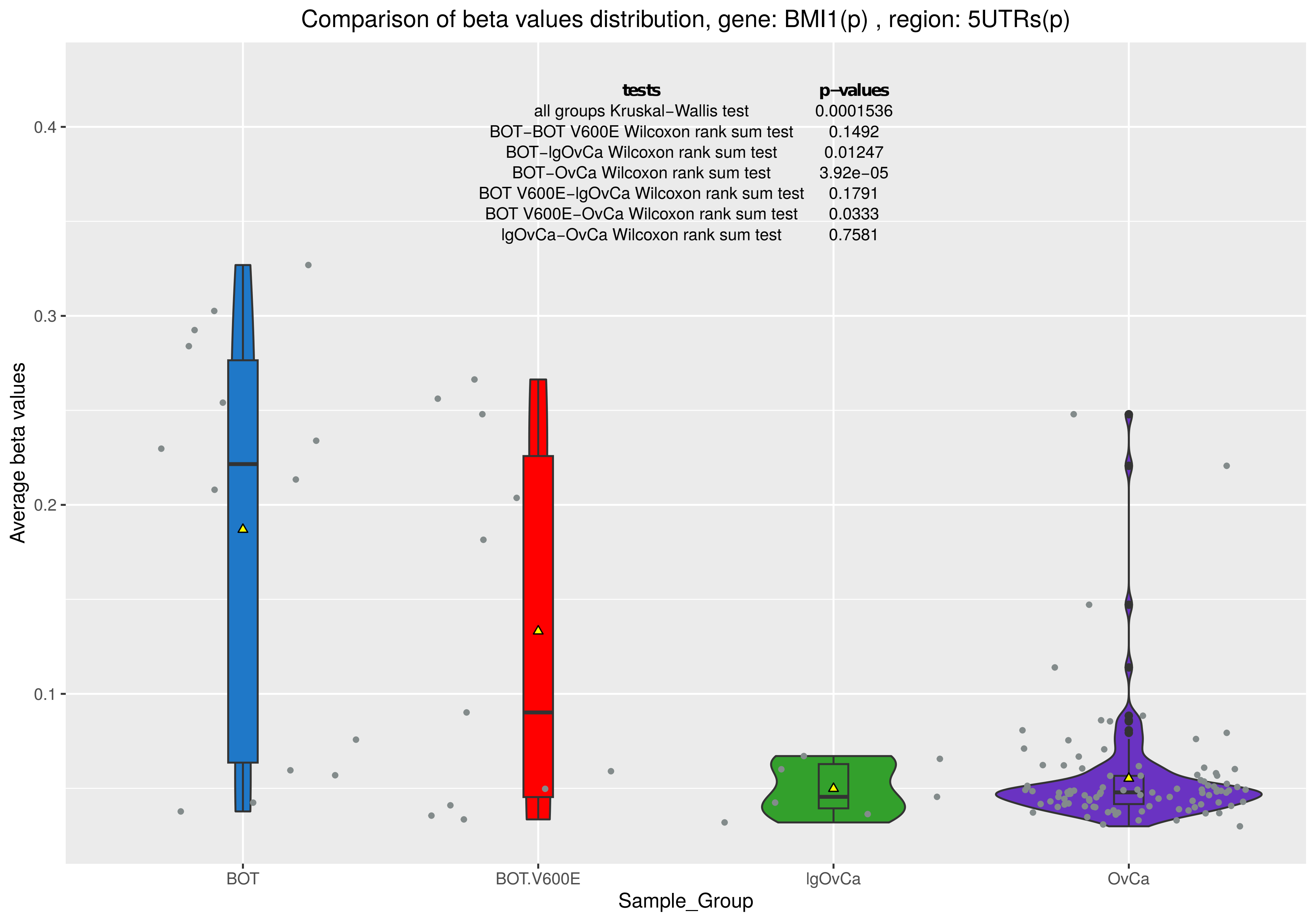

Comparison of beta values distribution, gene: BMI1(p) , region: 5UTRs(p)
tests
p−values
all groups Kruskal−Wallis test
0.0001536
BOT−BOT V600E Wilcoxon rank sum test
0.1492
BOT−IgOvCa Wilcoxon rank sum test
0.01247
BOT−OvCa Wilcoxon rank sum test
3.92e−05
BOT V600E−IgOvCa Wilcoxon rank sum test
0.1791
BOT V600E−OvCa Wilcoxon rank sum test
0.0333
IgOvCa−OvCa Wilcoxon rank sum test
0.7581
Average beta values
0.4
0.3
0.2
0.1
BOT
BOT.V600E
IgOvCa
OvCa
Sample_Group

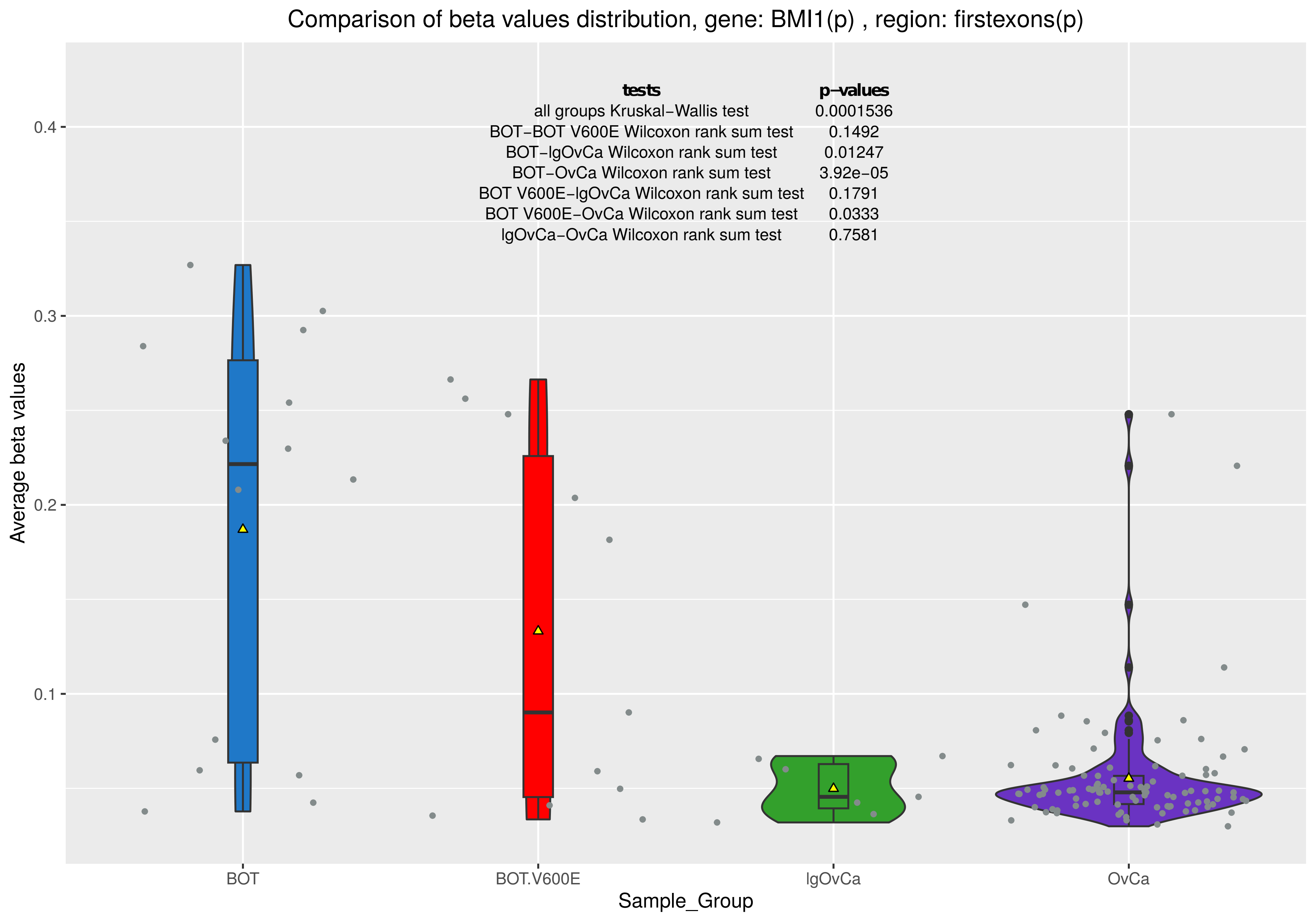
Comparison of beta values distribution, gene: BMI1(p) , region: firstexons(p)
tests
p−values
all groups Kruskal−Wallis test
0.0001536
BOT−BOT V600E Wilcoxon rank sum test
0.1492
BOT−lgOvCa Wilcoxon rank sum test
0.01247
BOT−OvCa Wilcoxon rank sum test
3.92e−05
BOT V600E−lgOvCa Wilcoxon rank sum test
0.1791
BOT V600E−OvCa Wilcoxon rank sum test
0.0333
lgOvCa−OvCa Wilcoxon rank sum test
0.7581
Average beta values
0.4
0.3
0.2
0.1
BOT
BOT.V600E
lgOvCa
OvCa
Sample_Group

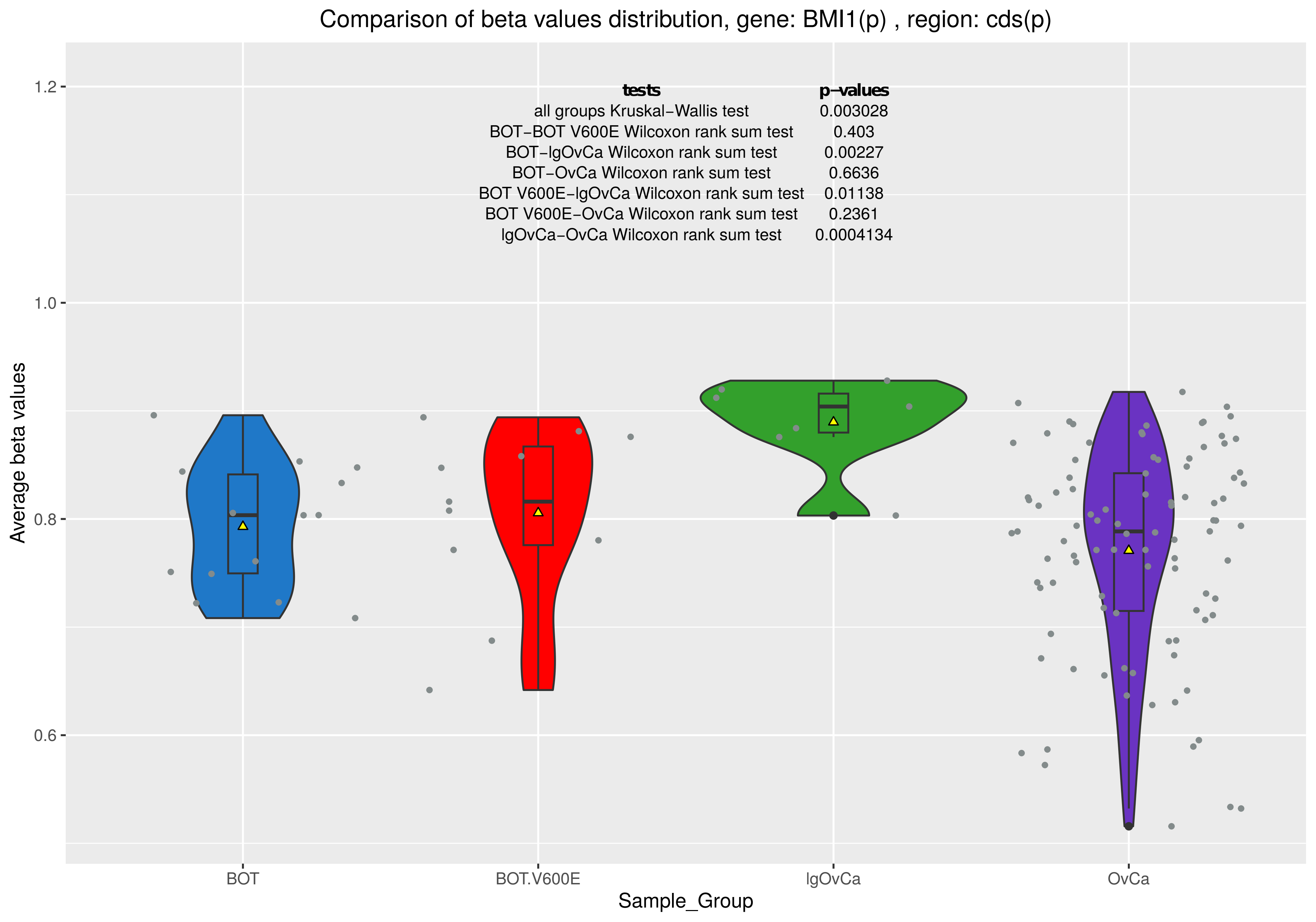

Comparison of beta values distribution, gene: BMI1(p) , region: cds(p)
tests
p−values
all groups Kruskal−Wallis test
0.003028
BOT−BOT V600E Wilcoxon rank sum test
0.403
BOT−lgOvCa Wilcoxon rank sum test
0.00227
BOT−OvCa Wilcoxon rank sum test
0.6636
BOT V600E−lgOvCa Wilcoxon rank sum test
0.01138
BOT V600E−OvCa Wilcoxon rank sum test
0.2361
lgOvCa−OvCa Wilcoxon rank sum test
0.0004134
Average beta values
1.2
1.0
0.8
0.6
BOT
BOT.V600E
lgOvCa
OvCa
Sample_Group

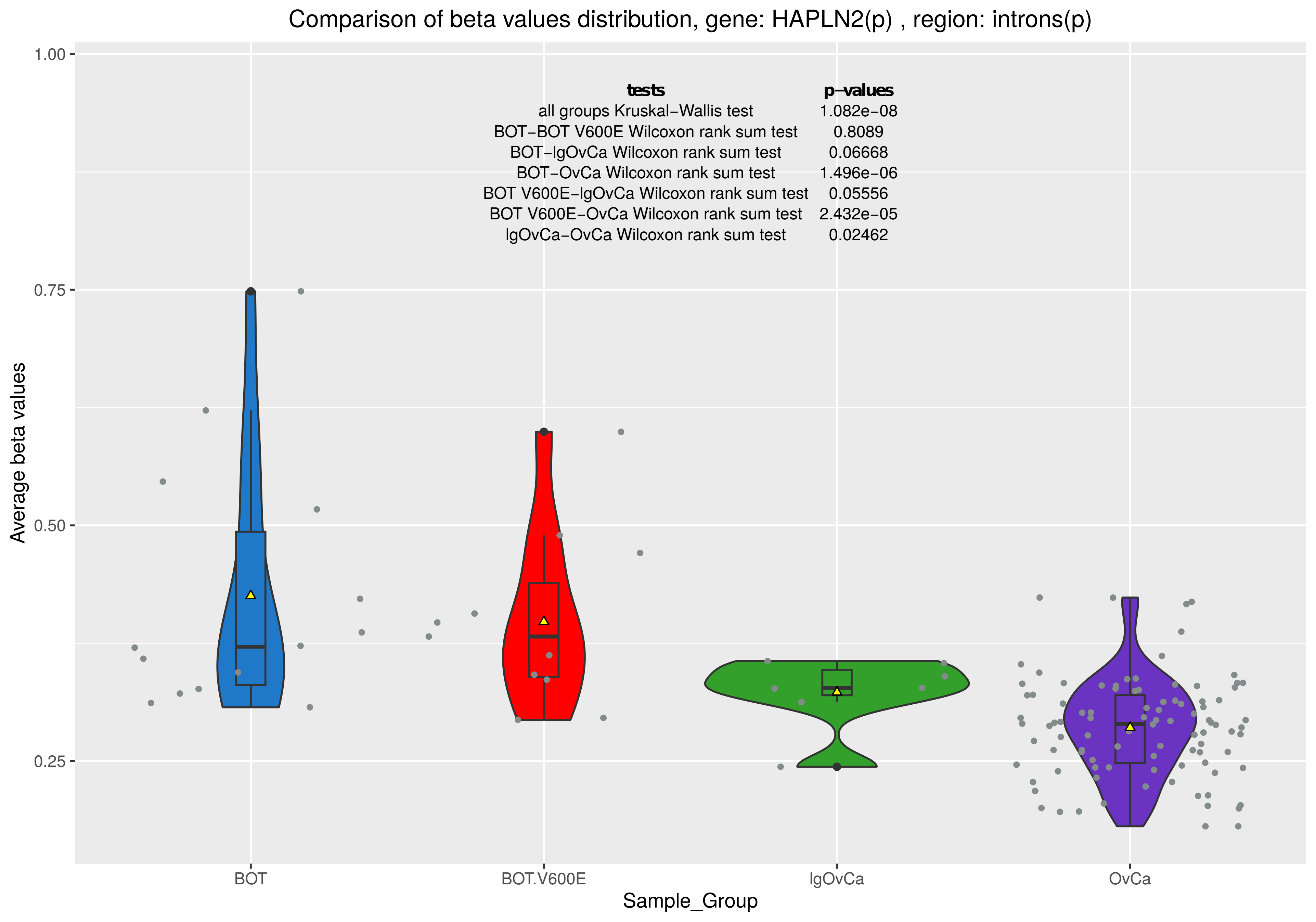
Comparison of beta values distribution, gene: HAPLN2(p) , region: introns(p)
tests
p−values
all groups Kruskal−Wallis test
1.082e−08
BOT−BOT V600E Wilcoxon rank sum test
0.8089
BOT−lgOvCa Wilcoxon rank sum test
0.06668
BOT−OvCa Wilcoxon rank sum test
1.496e−06
BOT V600E−lgOvCa Wilcoxon rank sum test
0.05556
BOT V600E−OvCa Wilcoxon rank sum test
2.432e−05
lgOvCa−OvCa Wilcoxon rank sum test
0.02462
Average beta values
1.00
0.75
0.50
0.25
BOT
BOT.V600E
lgOvCa
OvCa
Sample_Group

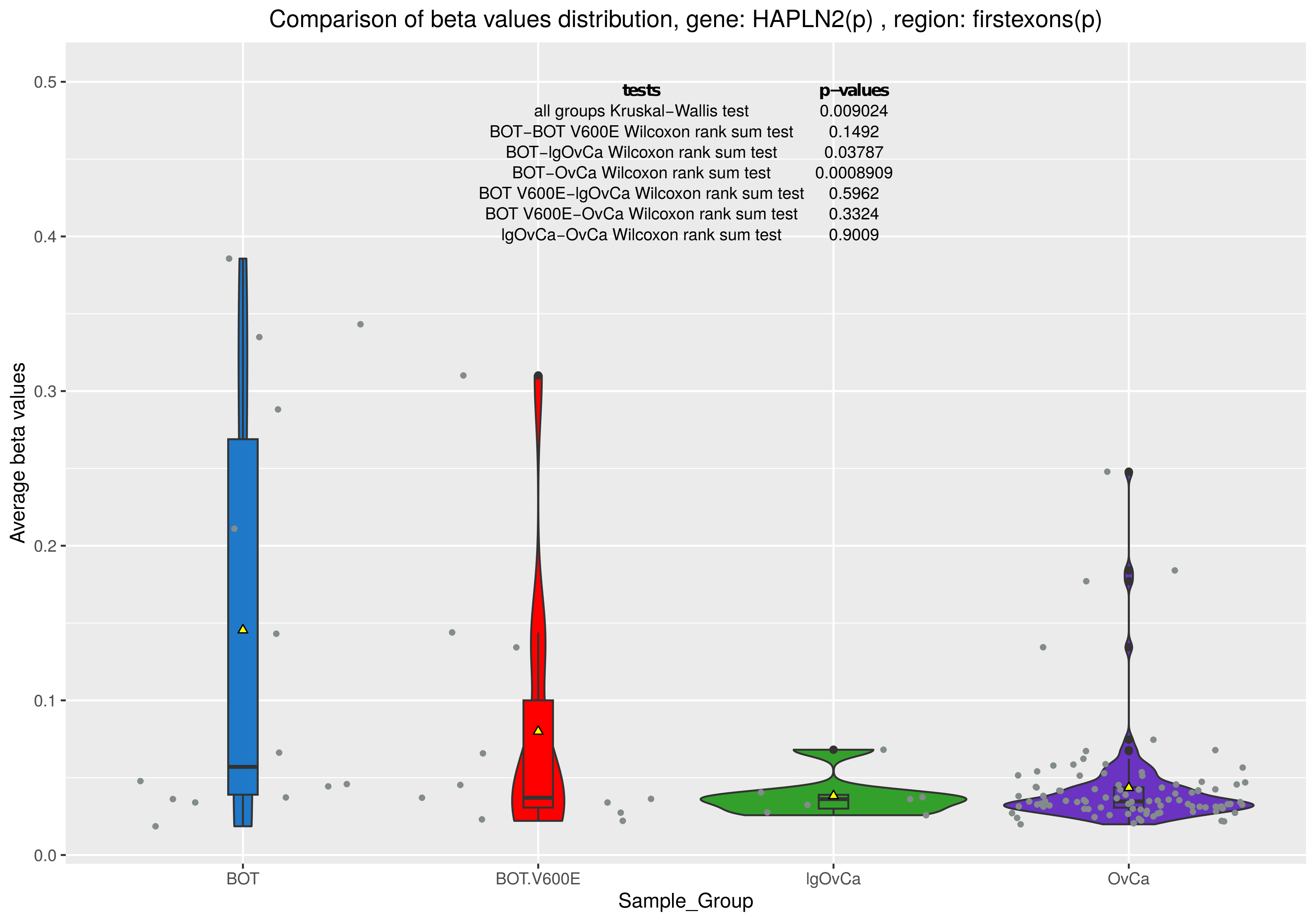
Comparison of beta values distribution, gene: HAPLN2(p) , region: firstexons(p)
tests
p−values
all groups Kruskal−Wallis test
0.009024
BOT−BOT V600E Wilcoxon rank sum test
0.1492
BOT−lgOvCa Wilcoxon rank sum test
0.03787
BOT−OvCa Wilcoxon rank sum test
0.0008909
BOT V600E−lgOvCa Wilcoxon rank sum test
0.5962
BOT V600E−OvCa Wilcoxon rank sum test
0.3324
lgOvCa−OvCa Wilcoxon rank sum test
0.9009
Average beta values
0.0
0.1
0.2
0.3
0.4
0.5
BOT
BOT.V600E
lgOvCa
OvCa
Sample_Group

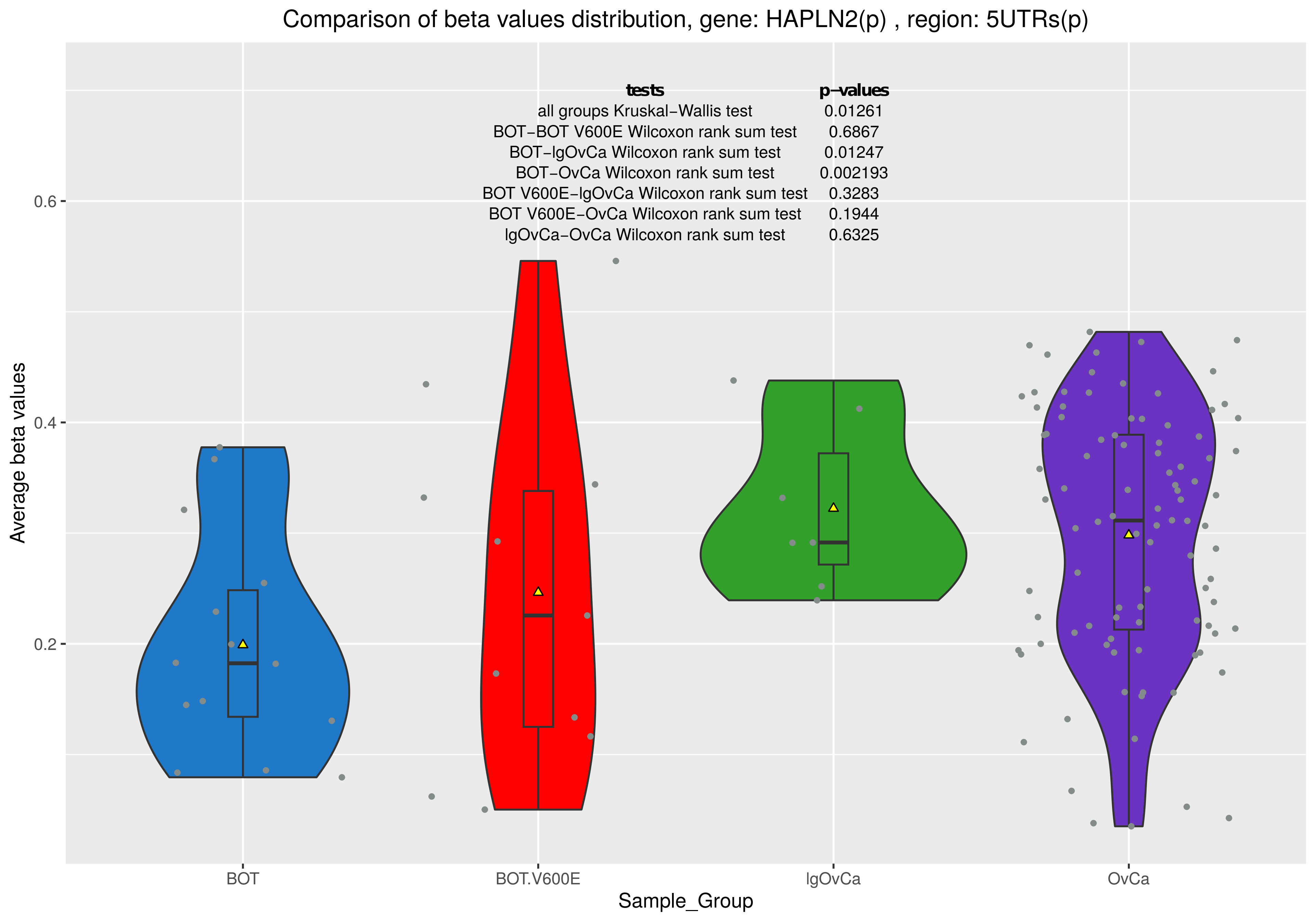
Comparison of beta values distribution, gene: HAPLN2(p) , region: 5UTRs(p)
tests
p−values
all groups Kruskal−Wallis test
0.01261
BOT−BOT V600E Wilcoxon rank sum test
0.6867
BOT−lgOvCa Wilcoxon rank sum test
0.01247
BOT−OvCa Wilcoxon rank sum test
0.002193
BOT V600E−lgOvCa Wilcoxon rank sum test
0.3283
BOT V600E−OvCa Wilcoxon rank sum test
0.1944
lgOvCa−OvCa Wilcoxon rank sum test
0.6325
Average beta values
0.6
0.4
0.2
BOT
BOT.V600E
lgOvCa
OvCa
Sample_Group

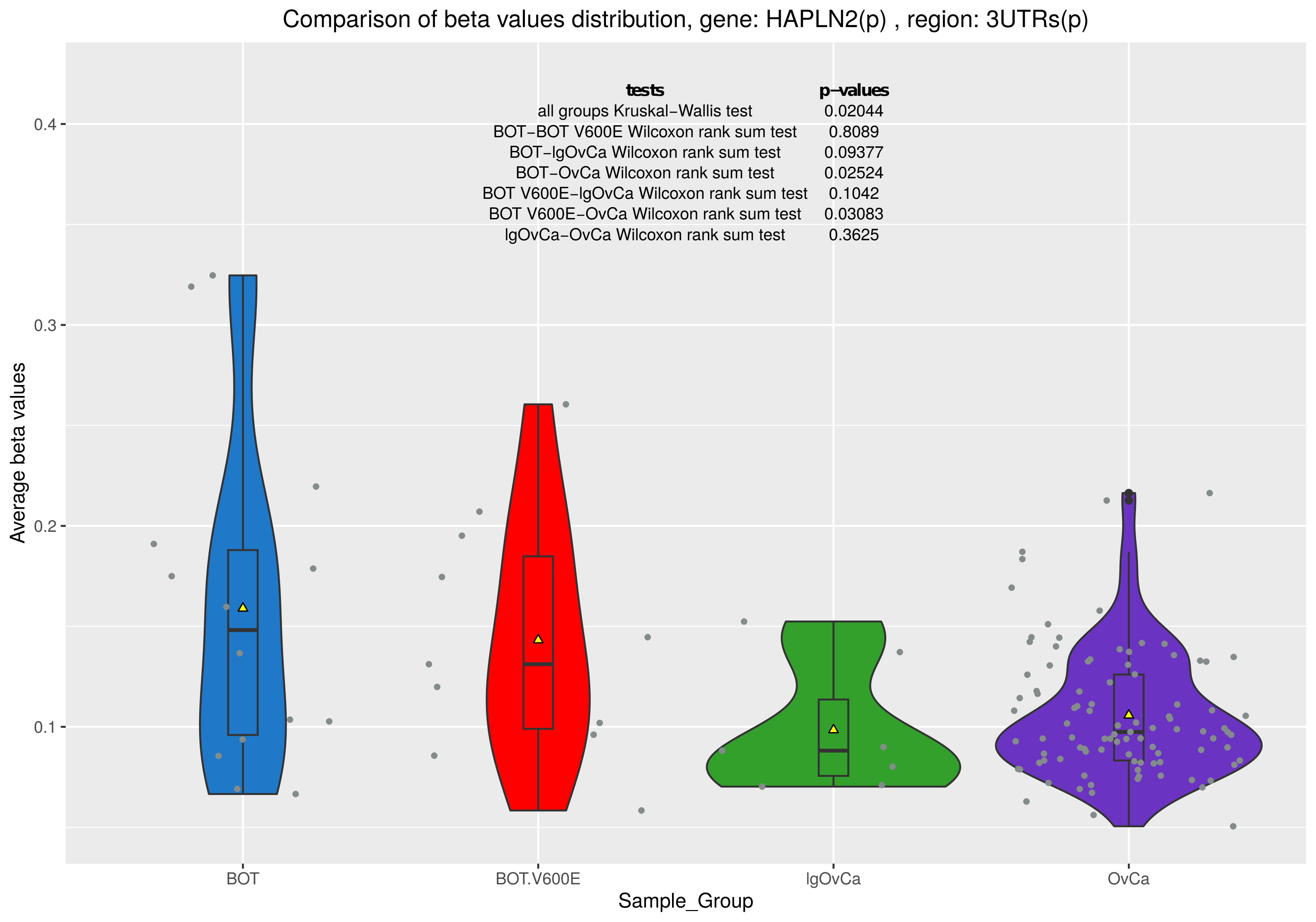

Comparison of beta values distribution, gene: HAPLN2(p) , region: 3UTRs(p)
tests
p−values
all groups Kruskal−Wallis test
0.02044
BOT−BOT V600E Wilcoxon rank sum test
0.8089
BOT−lgOvCa Wilcoxon rank sum test
0.09377
BOT−OvCa Wilcoxon rank sum test
0.02524
BOT V600E−lgOvCa Wilcoxon rank sum test
0.1042
BOT V600E−OvCa Wilcoxon rank sum test
0.03083
lgOvCa−OvCa Wilcoxon rank sum test
0.3625
Average beta values
0.4
0.3
0.2
0.1
BOT
BOT.V600E
lgOvCa
OvCa
Sample_Group

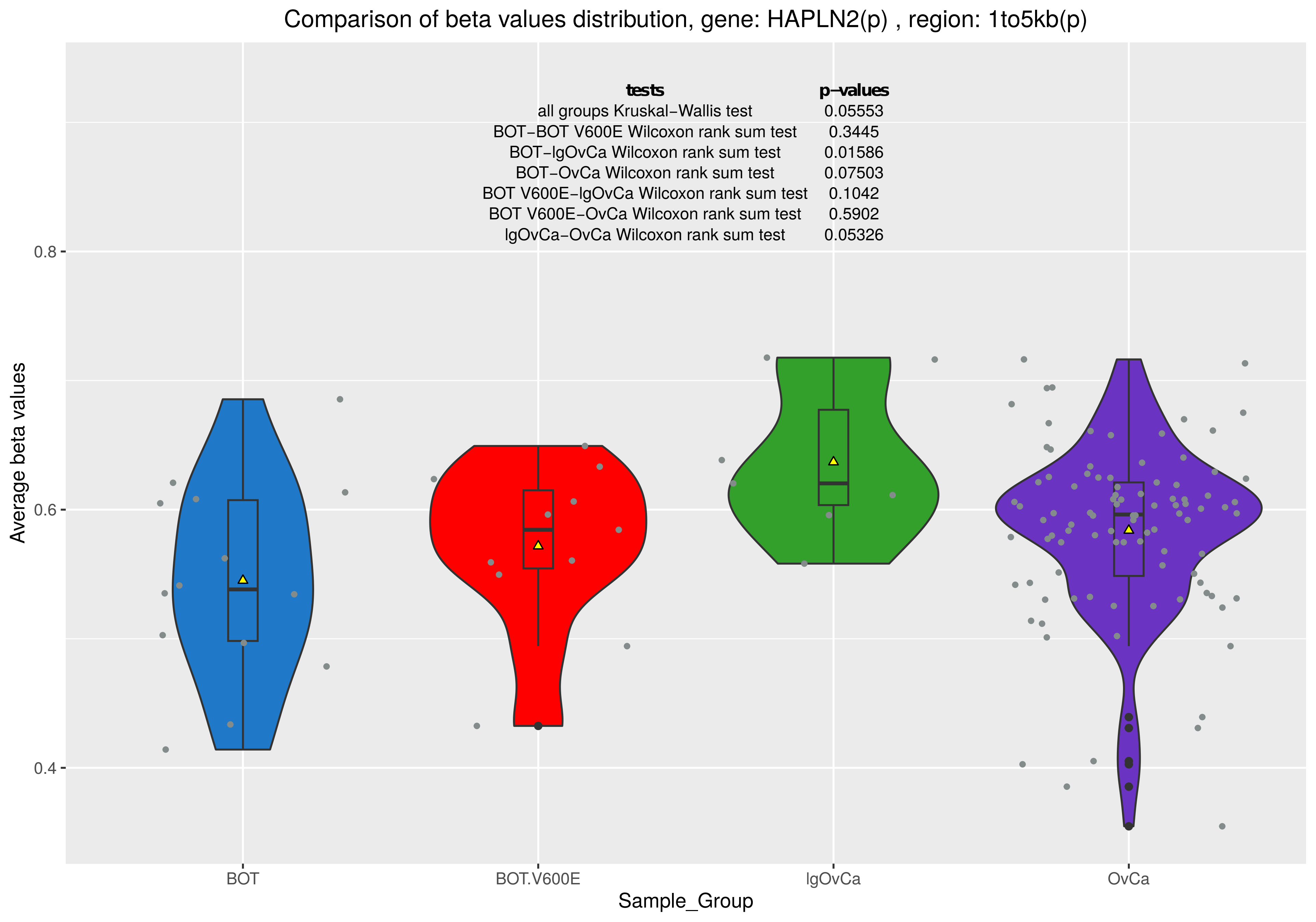
Comparison of beta values distribution, gene: HAPLN2(p) , region: 1to5kb(p)
tests
p−values
all groups Kruskal−Wallis test
0.05553
BOT−BOT V600E Wilcoxon rank sum test
0.3445
BOT−lgOvCa Wilcoxon rank sum test
0.01586
BOT−OvCa Wilcoxon rank sum test
0.07503
BOT V600E−lgOvCa Wilcoxon rank sum test
0.1042
BOT V600E−OvCa Wilcoxon rank sum test
0.5902
lgOvCa−OvCa Wilcoxon rank sum test
0.05326
Average beta values
0.8
0.6
0.4
BOT
BOT.V600E
lgOvCa
OvCa
Sample_Group

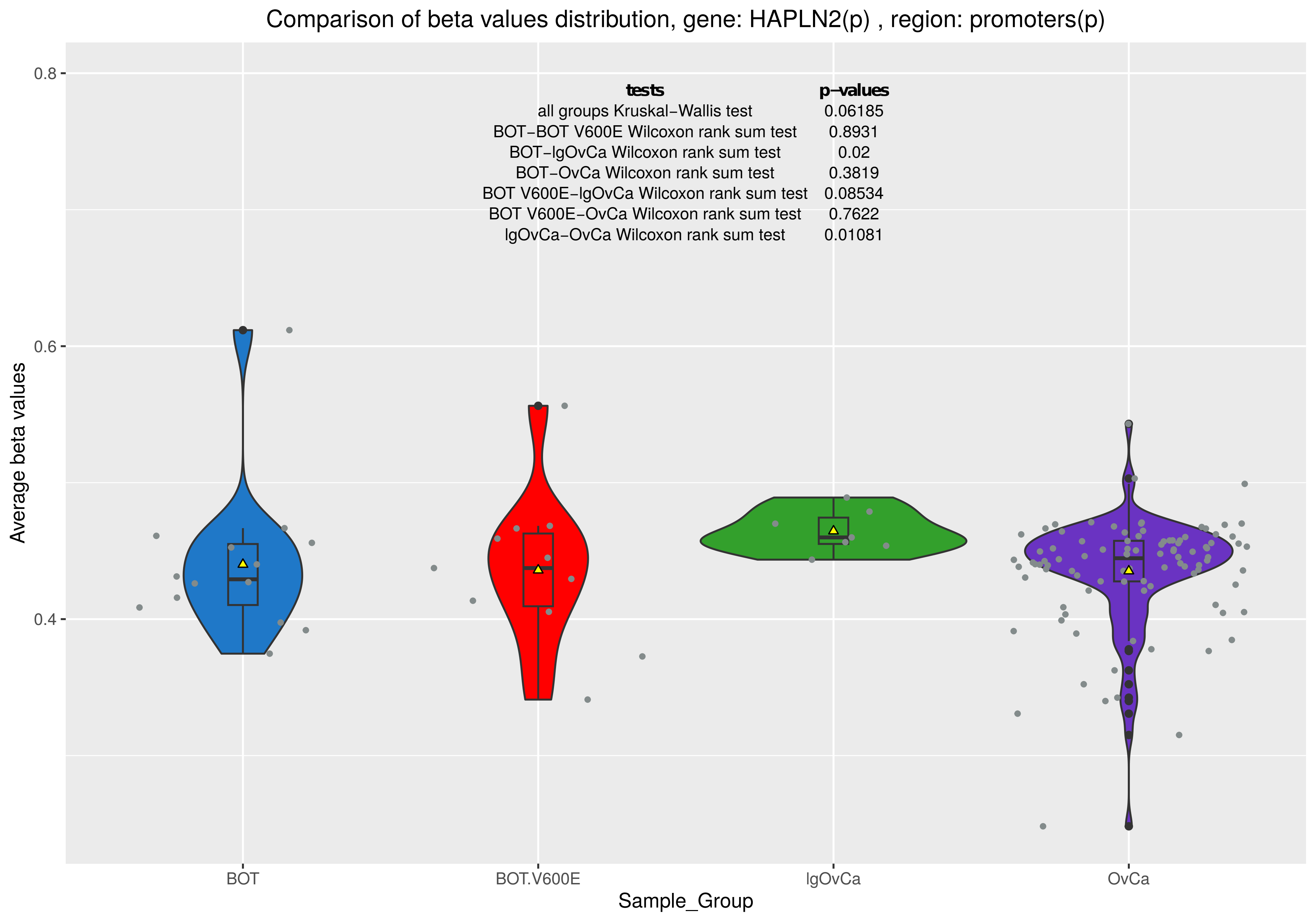

Comparison of beta values distribution, gene: HAPLN2(p) , region: promoters(p)
tests
p−values
all groups Kruskal−Wallis test
0.06185
BOT−BOT V600E Wilcoxon rank sum test
0.8931
BOT−lgOvCa Wilcoxon rank sum test
0.02
BOT−OvCa Wilcoxon rank sum test
0.3819
BOT V600E−lgOvCa Wilcoxon rank sum test
0.08534
BOT V600E−OvCa Wilcoxon rank sum test
0.7622
lgOvCa−OvCa Wilcoxon rank sum test
0.01081
Average beta values
0.8
0.6
0.4
BOT
BOT.V600E
lgOvCa
OvCa
Sample_Group

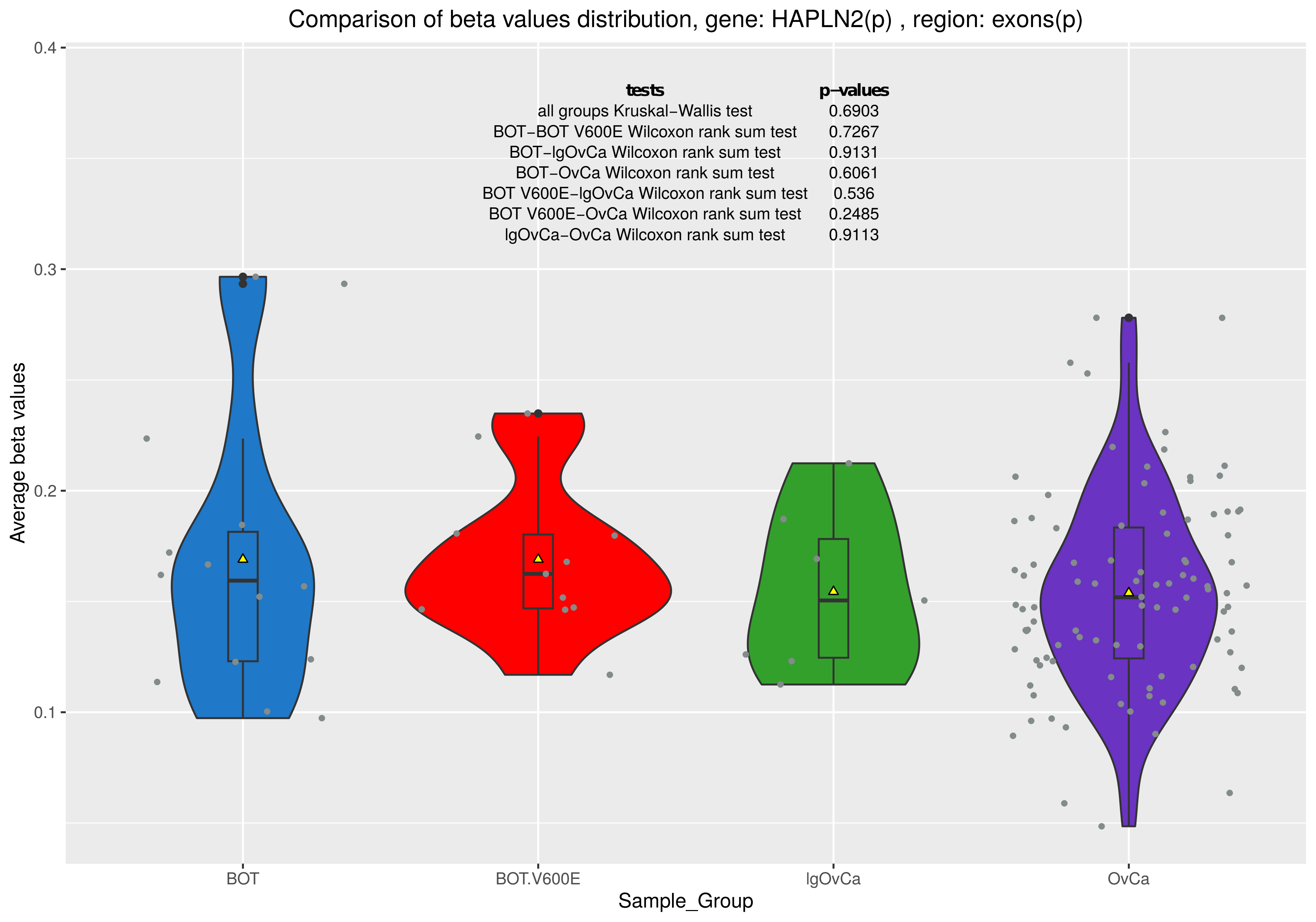

Comparison of beta values distribution, gene: HAPLN2(p) , region: exons(p)
tests
p−values
all groups Kruskal−Wallis test 0.6903
BOT−BOT V600E Wilcoxon rank sum test 0.7267
BOT−IgOvCa Wilcoxon rank sum test 0.9131
BOT−OvCa Wilcoxon rank sum test 0.6061
BOT V600E−IgOvCa Wilcoxon rank sum test 0.536
BOT V600E−OvCa Wilcoxon rank sum test 0.2485
IgOvCa−OvCa Wilcoxon rank sum test 0.9113
Average beta values
0.4
0.3
0.2
0.1
BOT
BOT.V600E
IgOvCa
OvCa
Sample_Group

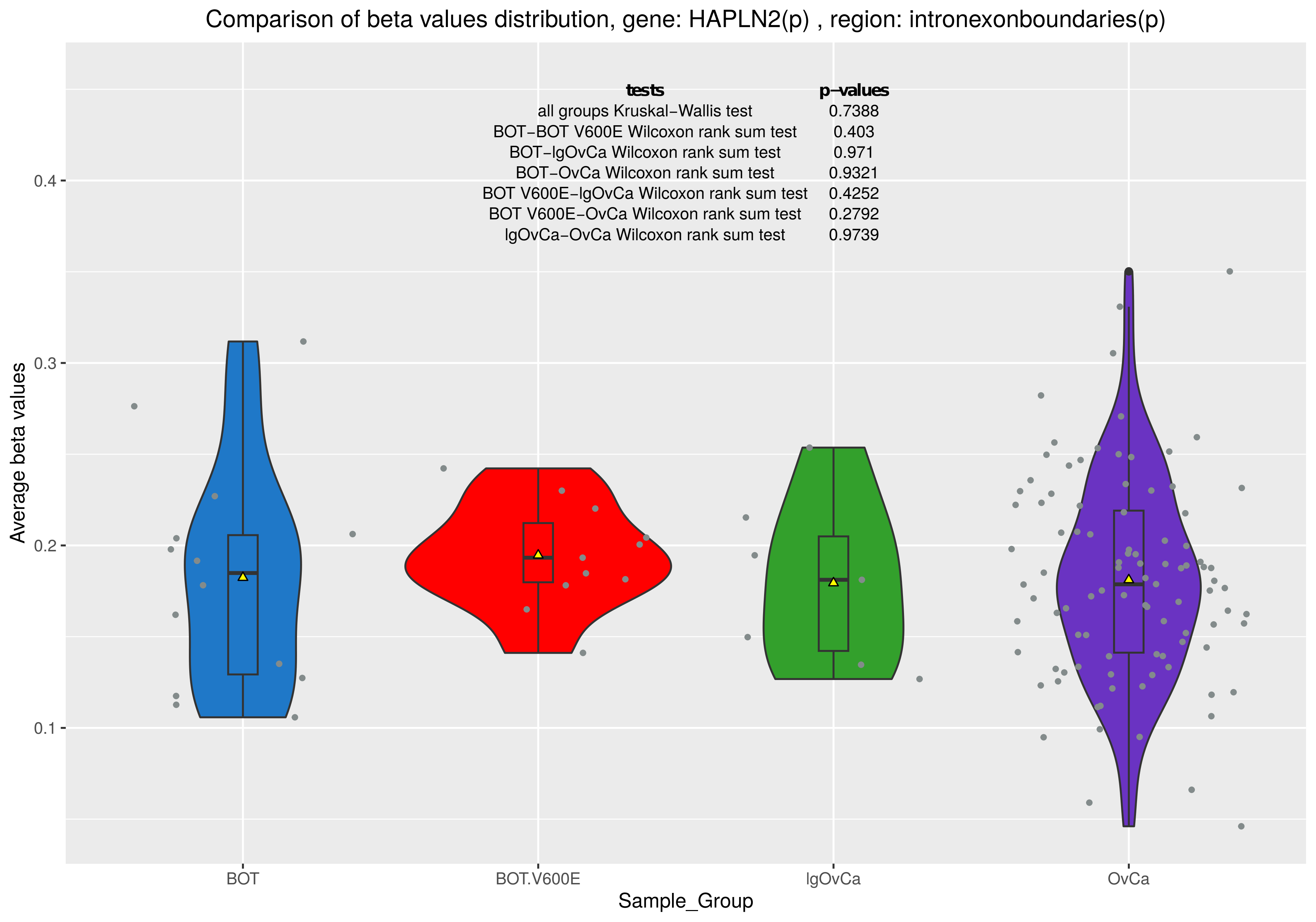

Comparison of beta values distribution, gene: HAPLN2(p) , region: intronexonboundaries(p)
tests
p−values
all groups Kruskal−Wallis test
0.7388
BOT−BOT V600E Wilcoxon rank sum test
0.403
BOT−lgOvCa Wilcoxon rank sum test
0.971
BOT−OvCa Wilcoxon rank sum test
0.9321
BOT V600E−lgOvCa Wilcoxon rank sum test
0.4252
BOT V600E−OvCa Wilcoxon rank sum test
0.2792
lgOvCa−OvCa Wilcoxon rank sum test
0.9739
Average beta values
0.4
0.3
0.2
0.1
BOT
BOT.V600E
lgOvCa
OvCa
Sample_Group

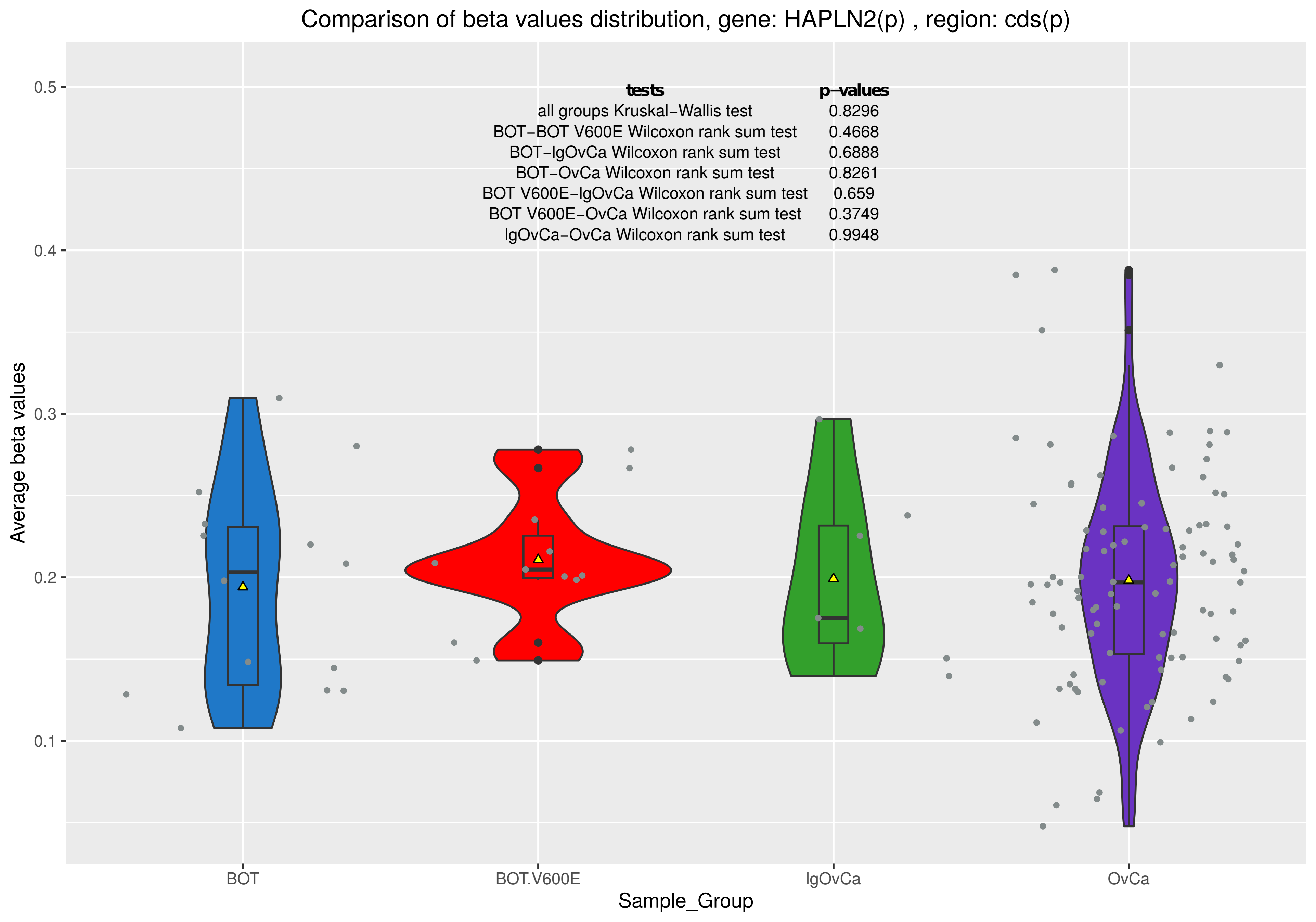

Comparison of beta values distribution, gene: HAPLN2(p) , region: cds(p)
tests
p−values
all groups Kruskal−Wallis test
0.8296
BOT−BOT V600E Wilcoxon rank sum test
0.4668
BOT−IgOvCa Wilcoxon rank sum test
0.6888
BOT−OvCa Wilcoxon rank sum test
0.8261
BOT V600E−IgOvCa Wilcoxon rank sum test
0.659
BOT V600E−OvCa Wilcoxon rank sum test
0.3749
IgOvCa−OvCa Wilcoxon rank sum test
0.9948
Average beta values
0.5
0.4
0.3
0.2
0.1
BOT
BOT.V600E
IgOvCa
OvCa
Sample_Group

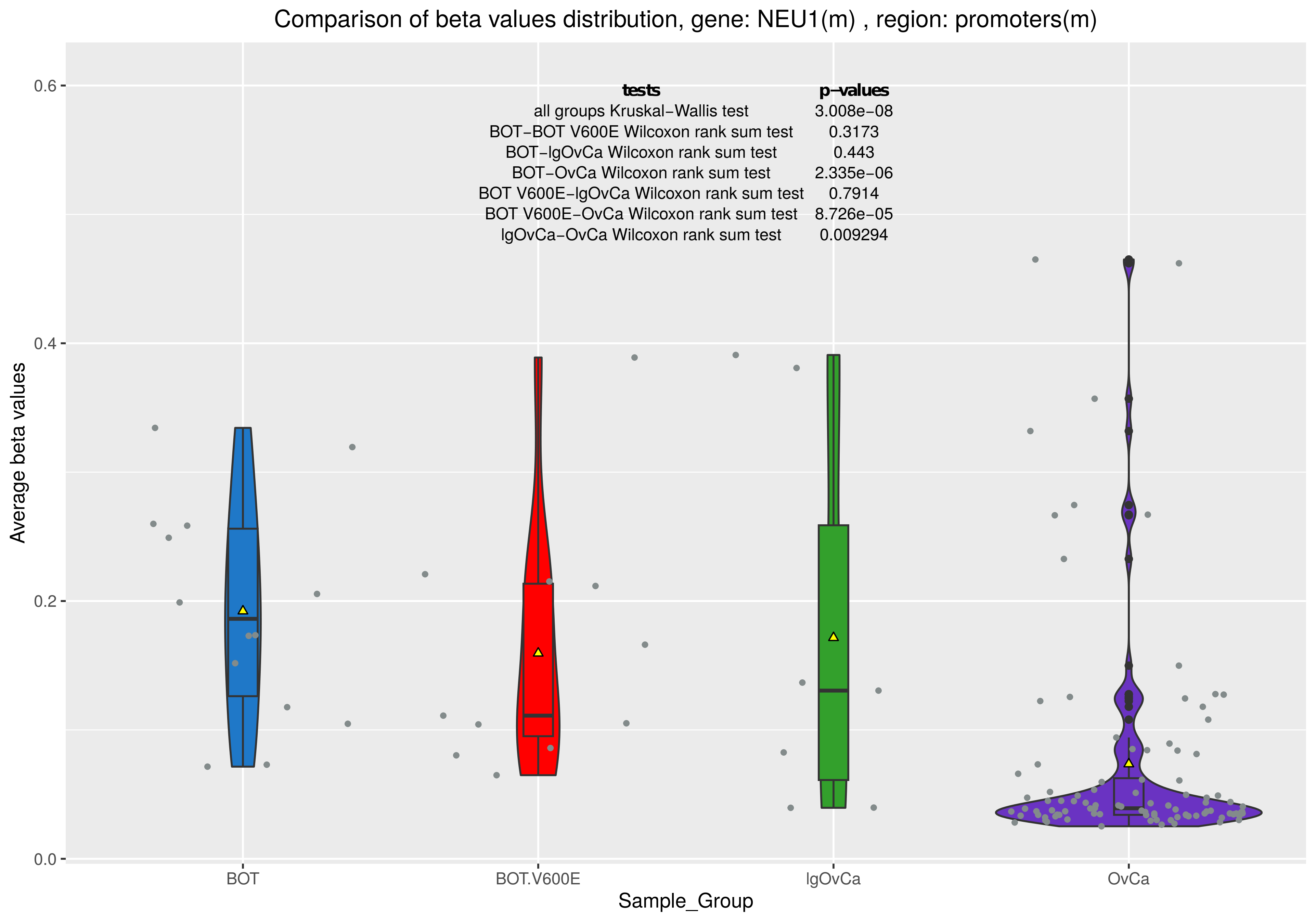

Comparison of beta values distribution, gene: NEU1(m) , region: promoters(m)
tests
p−values
all groups Kruskal−Wallis test
3.008e−08
BOT−BOT V600E Wilcoxon rank sum test
0.3173
BOT−lgOvCa Wilcoxon rank sum test
0.443
BOT−OvCa Wilcoxon rank sum test
2.335e−06
BOT V600E−lgOvCa Wilcoxon rank sum test
0.7914
BOT V600E−OvCa Wilcoxon rank sum test
8.726e−05
lgOvCa−OvCa Wilcoxon rank sum test
0.009294
Average beta values
0.0
0.2
0.4
0.6
BOT
BOT.V600E
lgOvCa
OvCa
Sample_Group

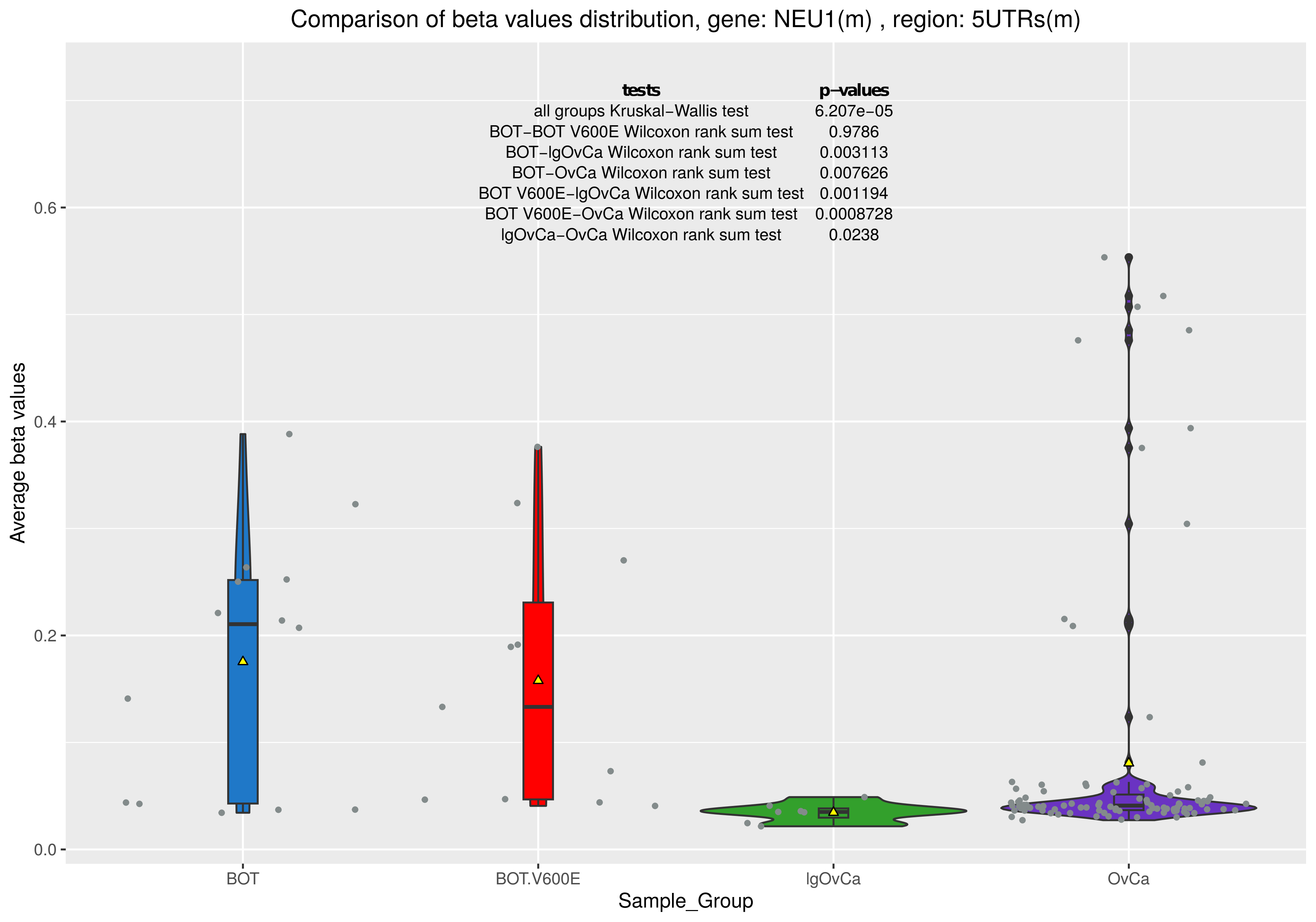
Comparison of beta values distribution, gene: NEU1(m) , region: 5UTRs(m)
tests
p−values
all groups Kruskal−Wallis test
6.207e−05
BOT−BOT V600E Wilcoxon rank sum test
0.9786
BOT−lgOvCa Wilcoxon rank sum test
0.003113
BOT−OvCa Wilcoxon rank sum test
0.007626
BOT V600E−lgOvCa Wilcoxon rank sum test
0.001194
BOT V600E−OvCa Wilcoxon rank sum test
0.0008728
lgOvCa−OvCa Wilcoxon rank sum test
0.0238
Average beta values
0.6
0.4
0.2
0.0
BOT
BOT.V600E
lgOvCa
OvCa
Sample_Group

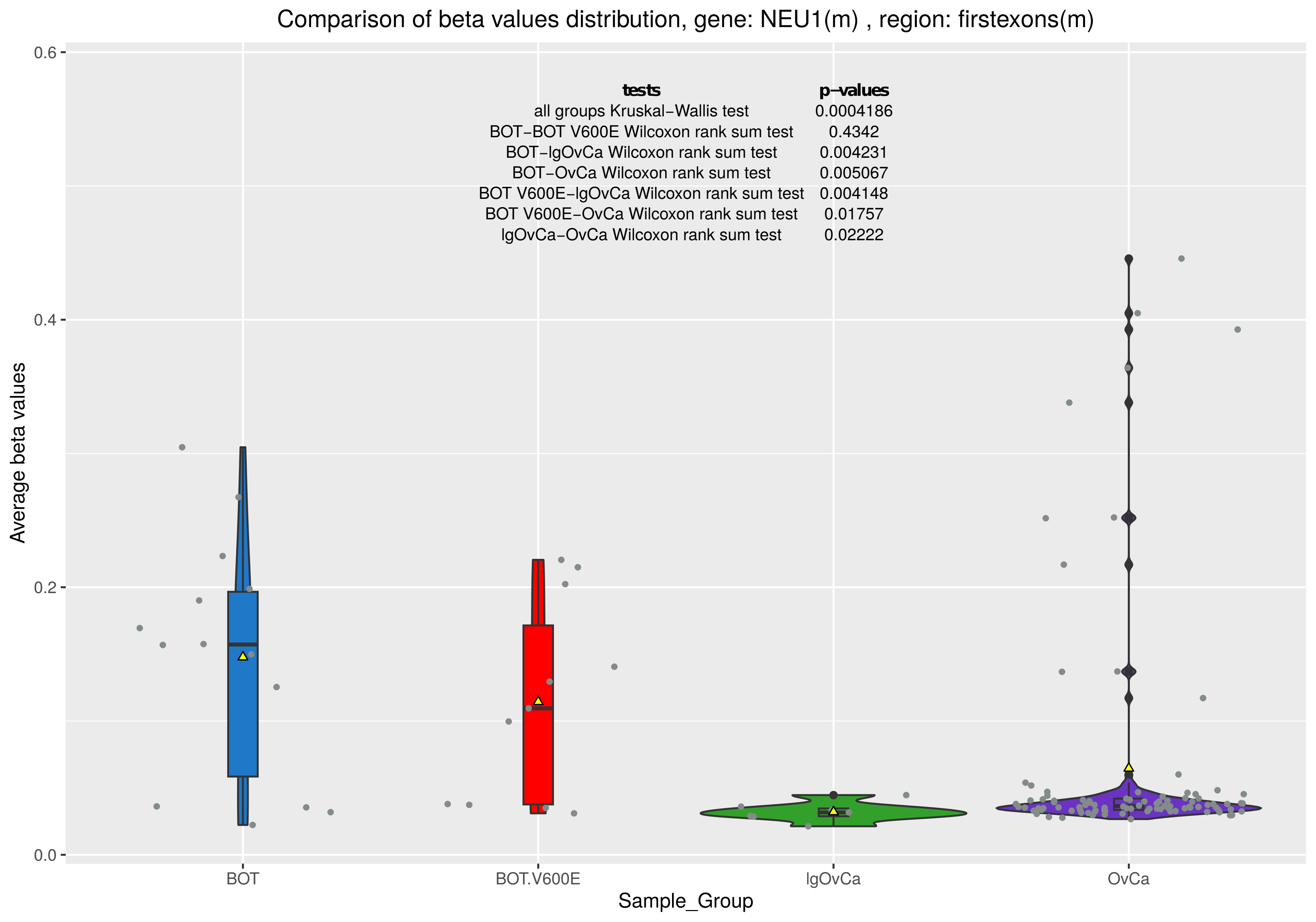
Comparison of beta values distribution, gene: NEU1(m) , region: firstexons(m)
tests
p−values
all groups Kruskal−Wallis test
0.0004186
BOT−BOT V600E Wilcoxon rank sum test
0.4342
BOT−lgOvCa Wilcoxon rank sum test
0.004231
BOT−OvCa Wilcoxon rank sum test
0.005067
BOT V600E−lgOvCa Wilcoxon rank sum test
0.004148
BOT V600E−OvCa Wilcoxon rank sum test
0.01757
lgOvCa−OvCa Wilcoxon rank sum test
0.02222
Average beta values
0.6
0.4
0.2
0.0
BOT
BOT.V600E
lgOvCa
OvCa
Sample_Group

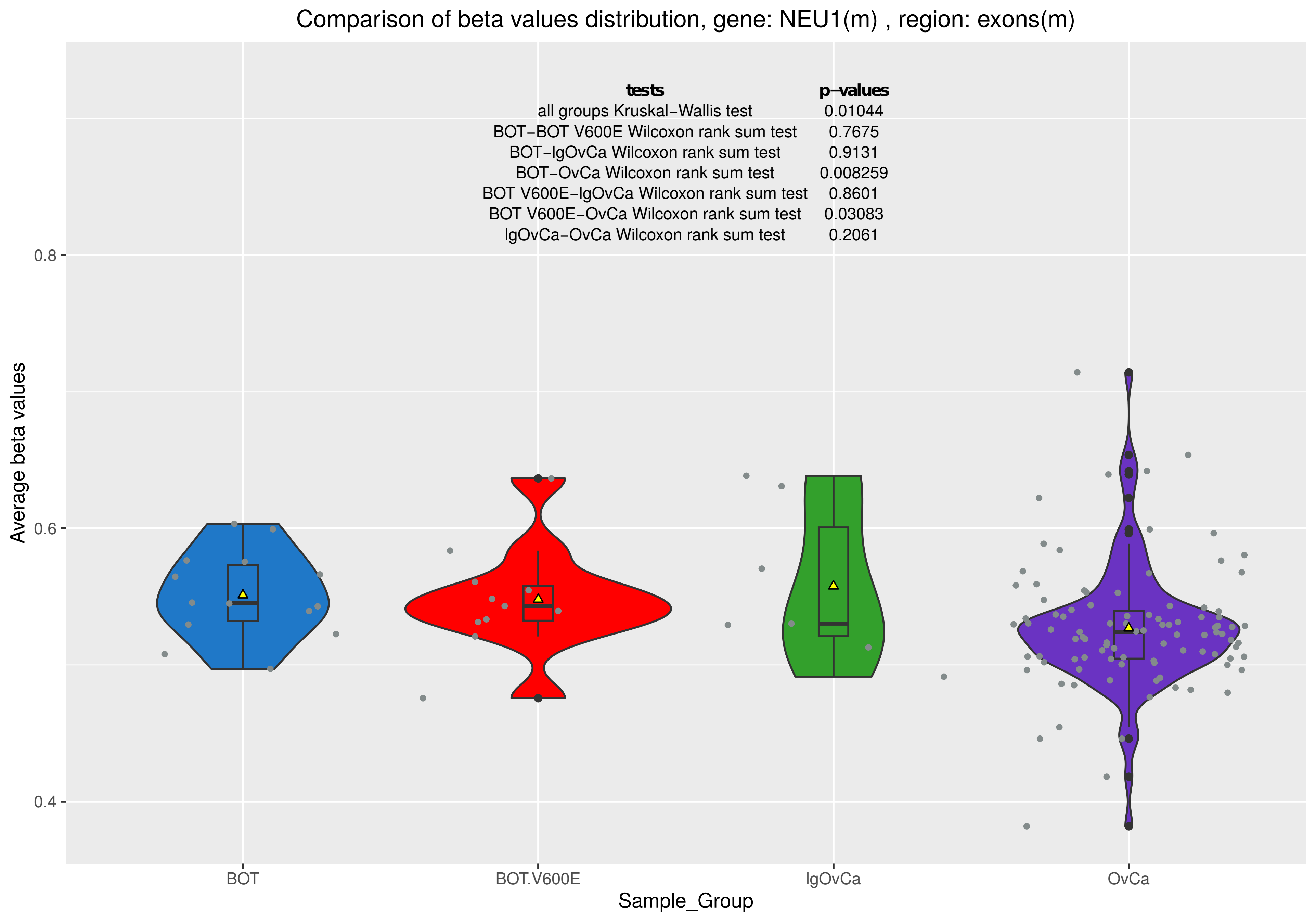

Comparison of beta values distribution, gene: NEU1(m) , region: exons(m)
tests
p-values
all groups Kruskal-Wallis test
0.01044
BOT-BOT V600E Wilcoxon rank sum test
0.7675
BOT-lgOvCa Wilcoxon rank sum test
0.9131
BOT-OvCa Wilcoxon rank sum test
0.008259
BOT V600E-lgOvCa Wilcoxon rank sum test
0.8601
BOT V600E-OvCa Wilcoxon rank sum test
0.03083
lgOvCa-OvCa Wilcoxon rank sum test
0.2061
Average beta values
0.8
0.6
0.4
BOT
BOT.V600E
lgOvCa
OvCa
Sample_Group

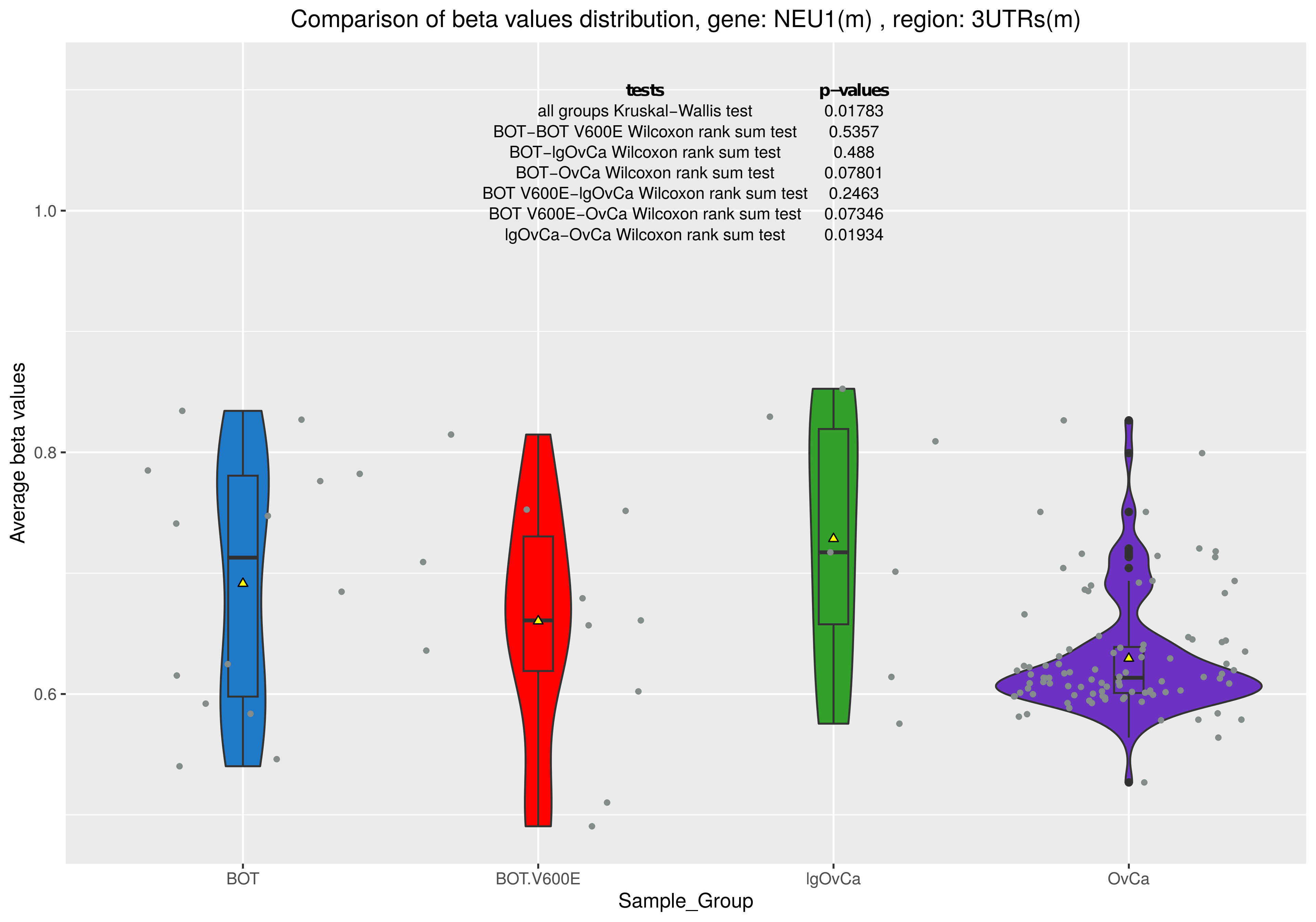

Comparison of beta values distribution, gene: NEU1(m) , region: 3UTRs(m)
tests
p−values
all groups Kruskal−Wallis test
0.01783
BOT−BOT V600E Wilcoxon rank sum test
0.5357
BOT−lgOvCa Wilcoxon rank sum test
0.488
BOT−OvCa Wilcoxon rank sum test
0.07801
BOT V600E−lgOvCa Wilcoxon rank sum test
0.2463
BOT V600E−OvCa Wilcoxon rank sum test
0.07346
lgOvCa−OvCa Wilcoxon rank sum test
0.01934
Average beta values
1.0
0.8
0.6
BOT
BOT.V600E
lgOvCa
OvCa
Sample_Group

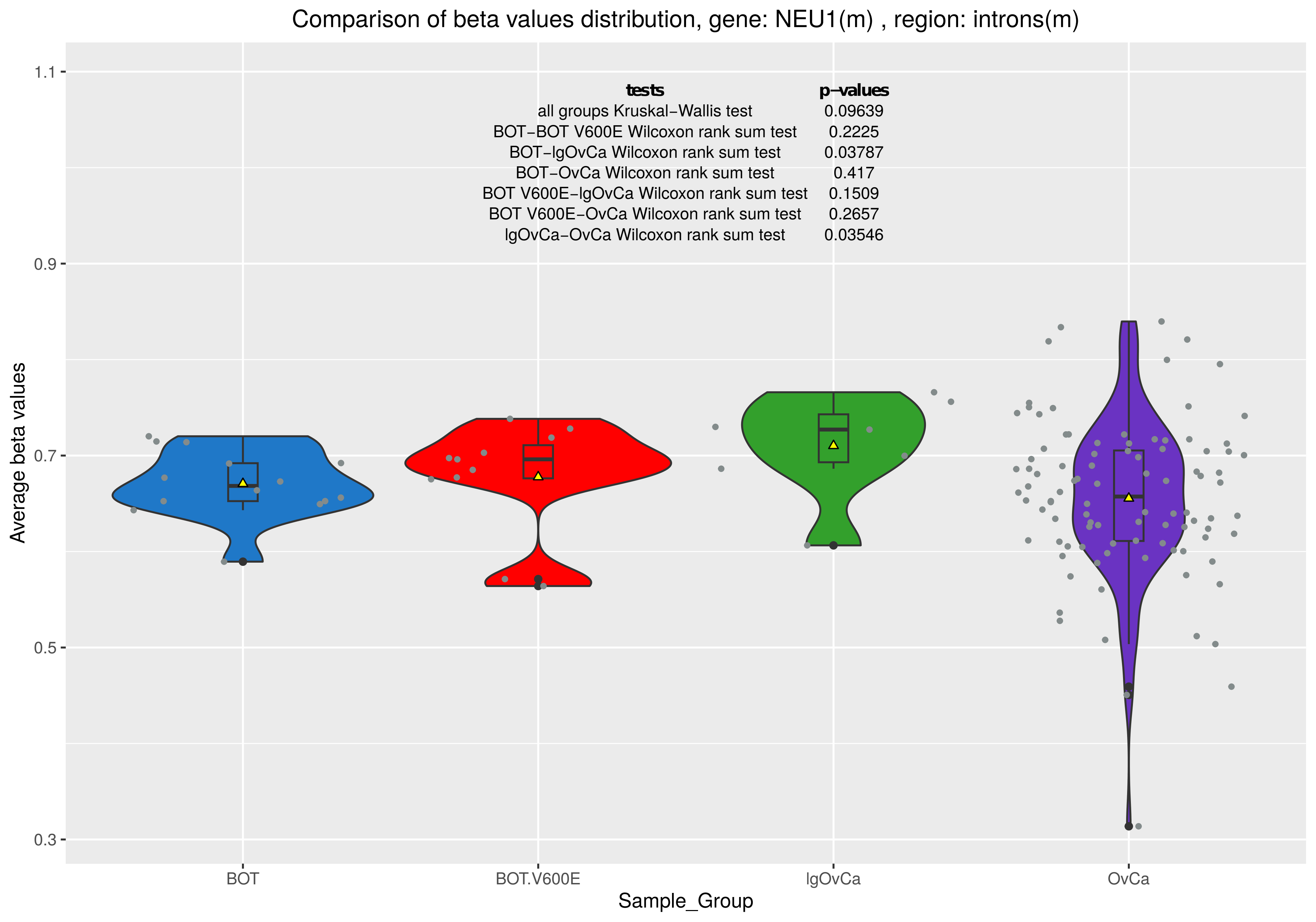

Comparison of beta values distribution, gene: NEU1(m) , region: introns(m)
tests
p−values
all groups Kruskal−Wallis test
0.09639
BOT−BOT V600E Wilcoxon rank sum test
0.2225
BOT−IgOvCa Wilcoxon rank sum test
0.03787
BOT−OvCa Wilcoxon rank sum test
0.417
BOT V600E−IgOvCa Wilcoxon rank sum test
0.1509
BOT V600E−OvCa Wilcoxon rank sum test
0.2657
IgOvCa−OvCa Wilcoxon rank sum test
0.03546
Average beta values
1.1
0.9
0.7
0.5
0.3
BOT
BOT.V600E
IgOvCa
OvCa
Sample_Group

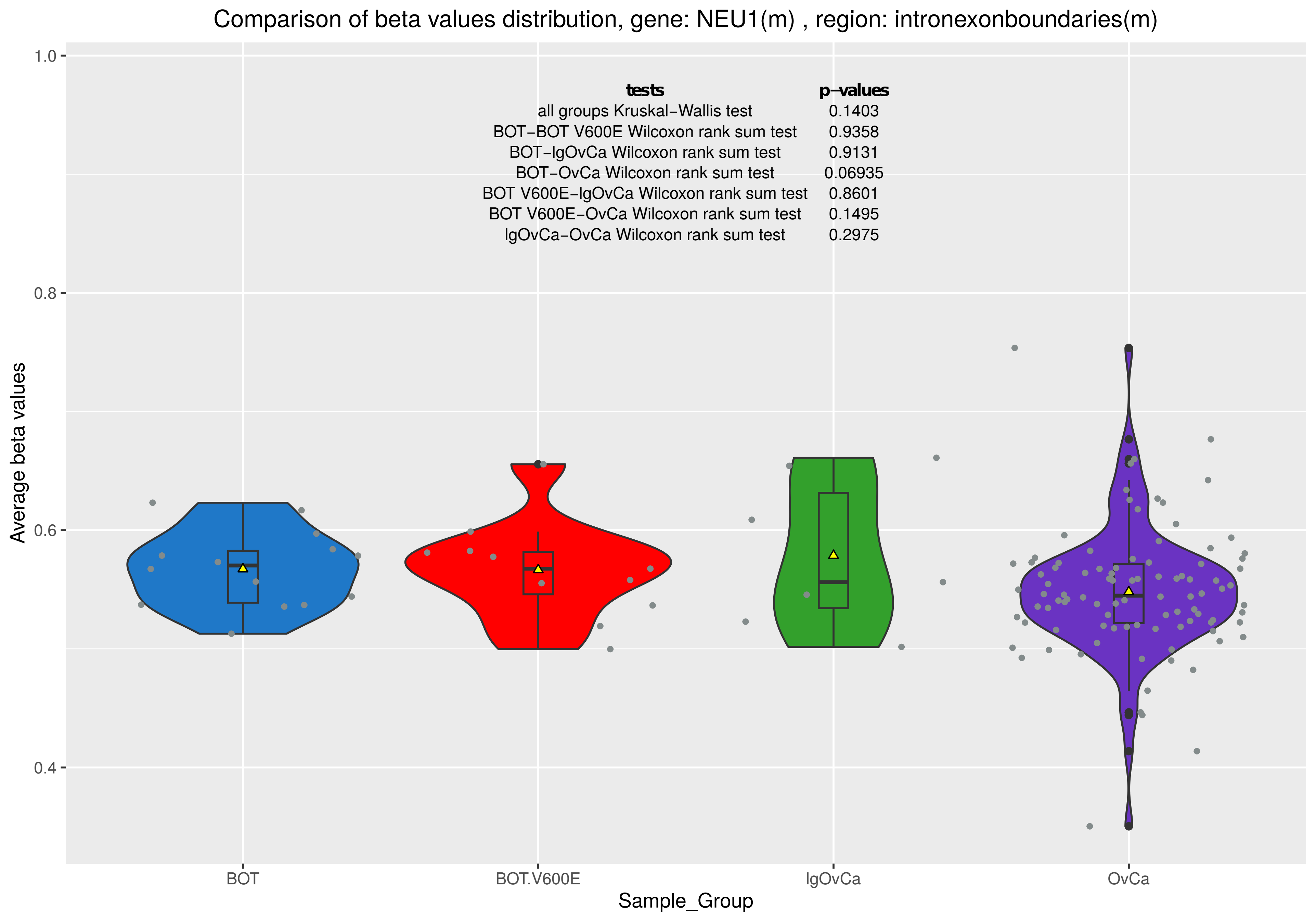

Comparison of beta values distribution, gene: NEU1(m) , region: intronexonboundaries(m)
tests
p−values
all groups Kruskal−Wallis test
0.1403
BOT−BOT V600E Wilcoxon rank sum test
0.9358
BOT−lgOvCa Wilcoxon rank sum test
0.9131
BOT−OvCa Wilcoxon rank sum test
0.06935
BOT V600E−lgOvCa Wilcoxon rank sum test
0.8601
BOT V600E−OvCa Wilcoxon rank sum test
0.1495
lgOvCa−OvCa Wilcoxon rank sum test
0.2975
Average beta values
1.0
0.8
0.6
0.4
BOT
BOT.V600E
lgOvCa
OvCa
Sample_Group

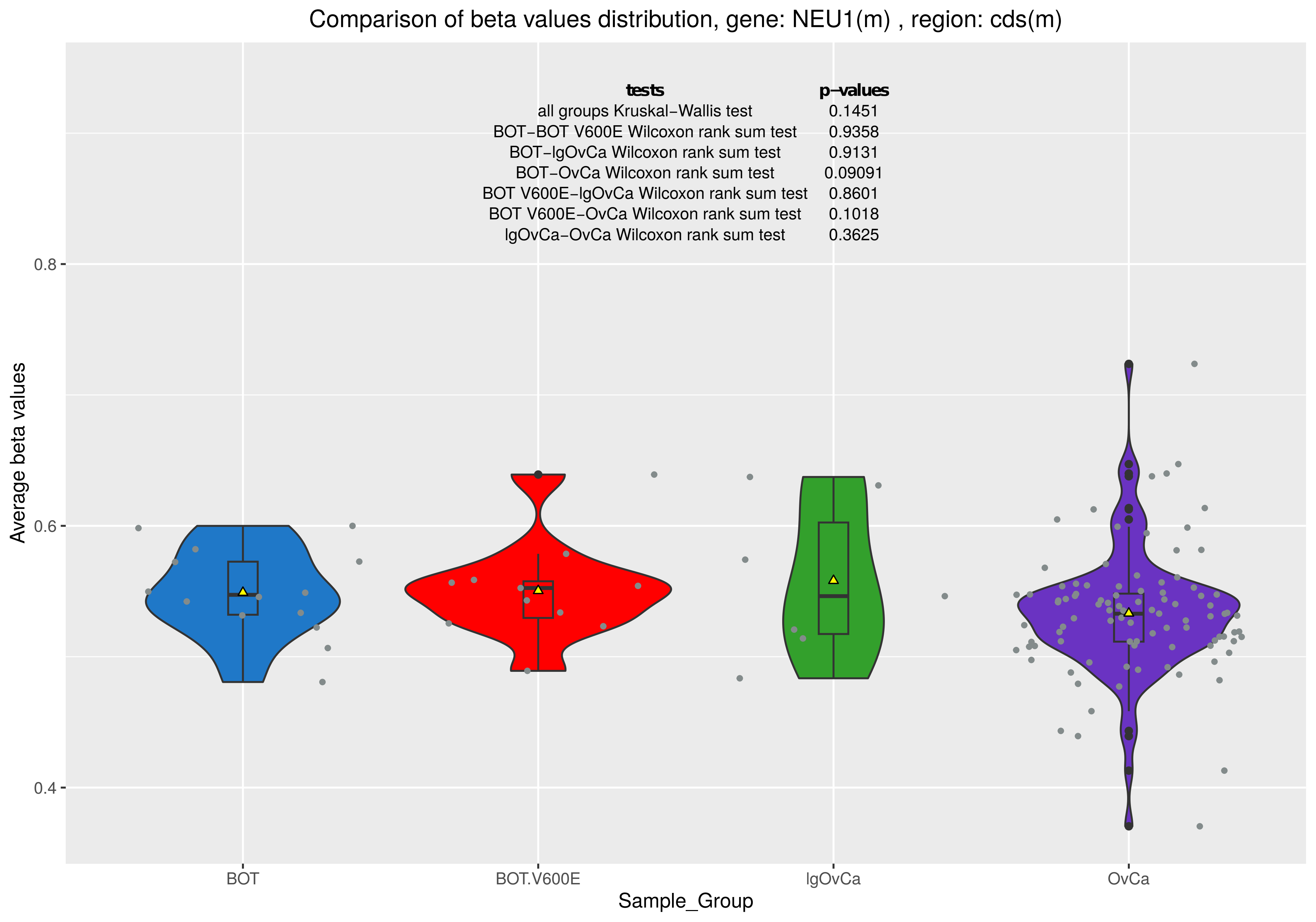
Comparison of beta values distribution, gene: NEU1(m) , region: cds(m)
tests
p−values
all groups Kruskal−Wallis test 0.1451
BOT−BOT V600E Wilcoxon rank sum test 0.9358
BOT−lgOvCa Wilcoxon rank sum test 0.9131
BOT−OvCa Wilcoxon rank sum test 0.09091
BOT V600E−lgOvCa Wilcoxon rank sum test 0.8601
BOT V600E−OvCa Wilcoxon rank sum test 0.1018
lgOvCa−OvCa Wilcoxon rank sum test 0.3625
Average beta values
0.8
0.6
0.4
BOT
BOT.V600E
lgOvCa
OvCa
Sample_Group

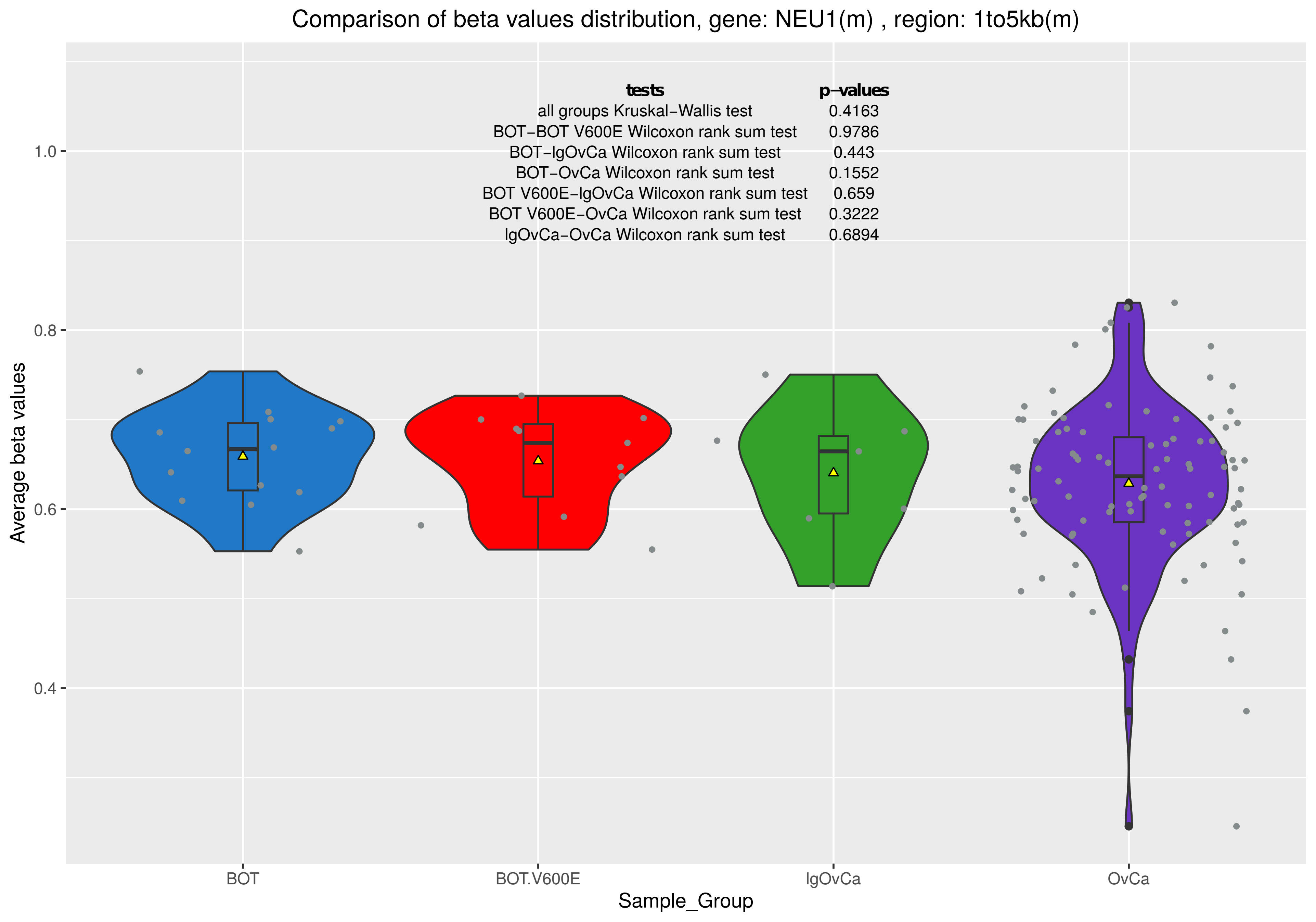
Comparison of beta values distribution, gene: NEU1(m) , region: 1to5kb(m)
tests
p−values
all groups Kruskal−Wallis test
0.4163
BOT−BOT V600E Wilcoxon rank sum test
0.9786
BOT−lgOvCa Wilcoxon rank sum test
0.443
BOT−OvCa Wilcoxon rank sum test
0.1552
BOT V600E−lgOvCa Wilcoxon rank sum test
0.659
BOT V600E−OvCa Wilcoxon rank sum test
0.3222
lgOvCa−OvCa Wilcoxon rank sum test
0.6894
Average beta values
1.0
0.8
0.6
0.4
BOT
BOT.V600E
lgOvCa
OvCa
Sample_Group

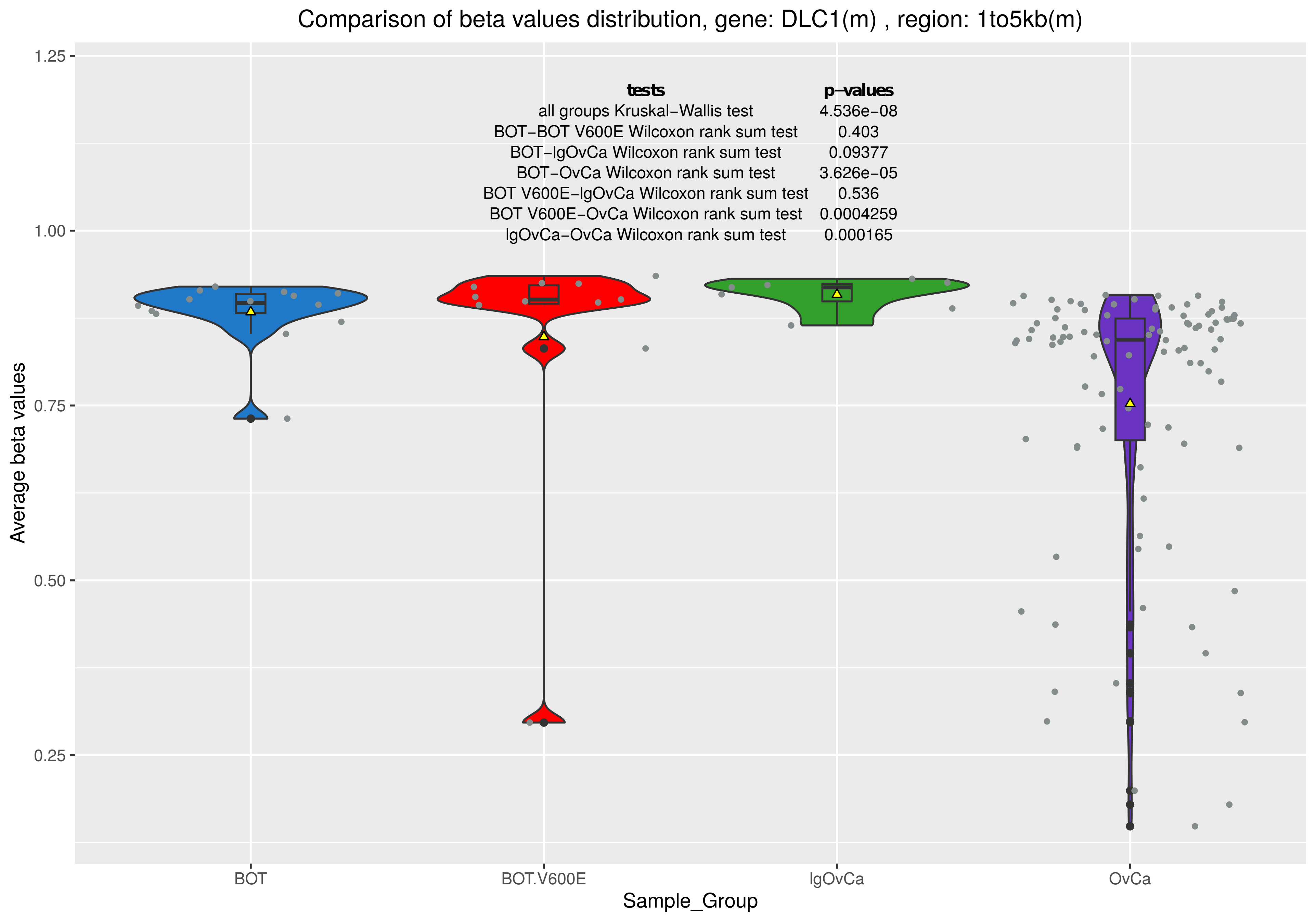

Comparison of beta values distribution, gene: DLC1(m) , region: 1to5kb(m)
tests
p−values
all groups Kruskal−Wallis test
4.536e−08
BOT−BOT V600E Wilcoxon rank sum test
0.403
BOT−lgOvCa Wilcoxon rank sum test
0.09377
BOT−OvCa Wilcoxon rank sum test
3.626e−05
BOT V600E−lgOvCa Wilcoxon rank sum test
0.536
BOT V600E−OvCa Wilcoxon rank sum test
0.0004259
lgOvCa−OvCa Wilcoxon rank sum test
0.000165
Average beta values
1.25
1.00
0.75
0.50
0.25
BOT
BOT.V600E
lgOvCa
OvCa
Sample_Group

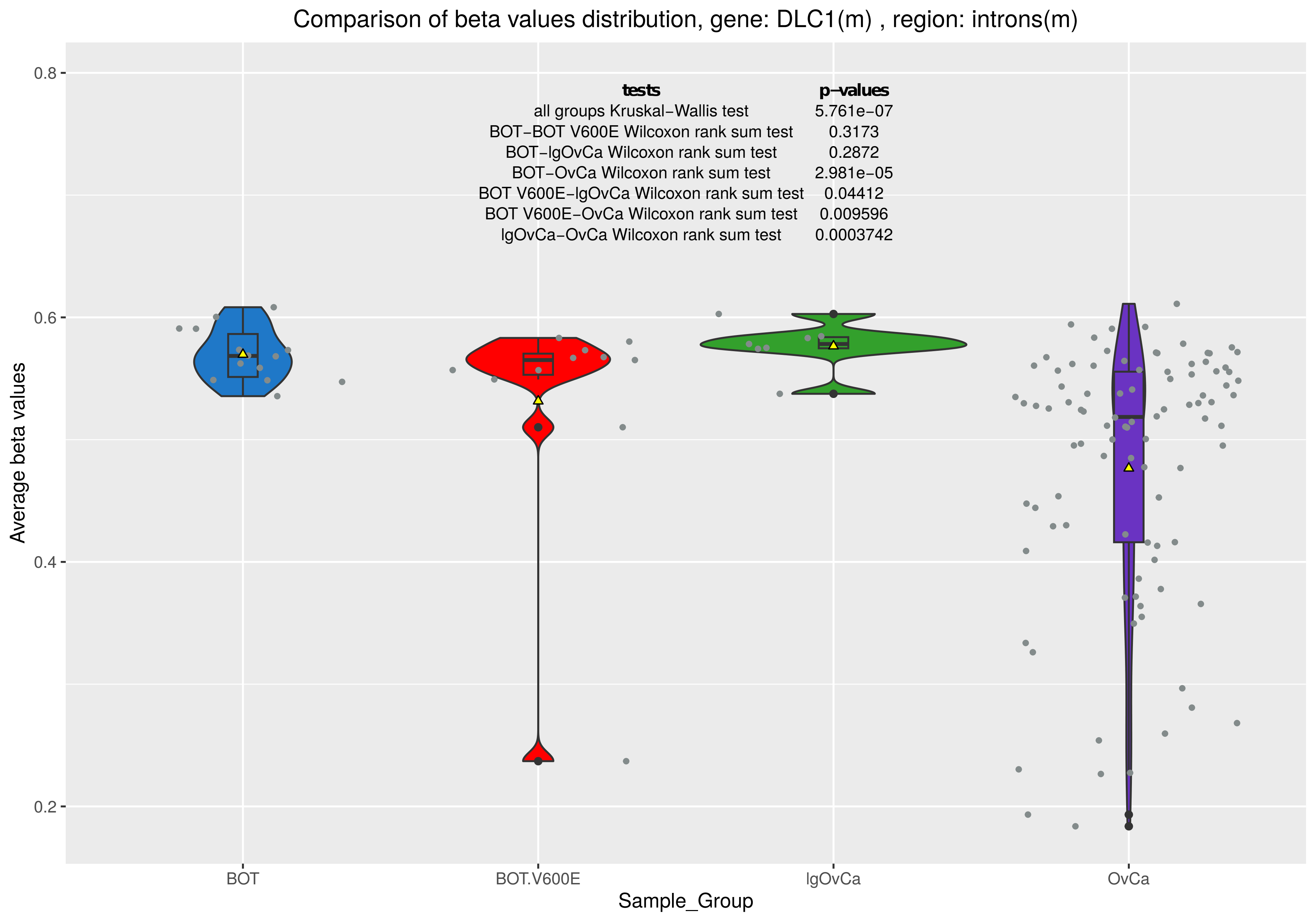

Comparison of beta values distribution, gene: DLC1(m) , region: introns(m)
tests
p−values
all groups Kruskal−Wallis test
5.761e−07
BOT−BOT V600E Wilcoxon rank sum test
0.3173
BOT−lgOvCa Wilcoxon rank sum test
0.2872
BOT−OvCa Wilcoxon rank sum test
2.981e−05
BOT V600E−lgOvCa Wilcoxon rank sum test
0.04412
BOT V600E−OvCa Wilcoxon rank sum test
0.009596
lgOvCa−OvCa Wilcoxon rank sum test
0.0003742
Average beta values
0.8
0.6
0.4
0.2
BOT
BOT.V600E
lgOvCa
OvCa
Sample_Group

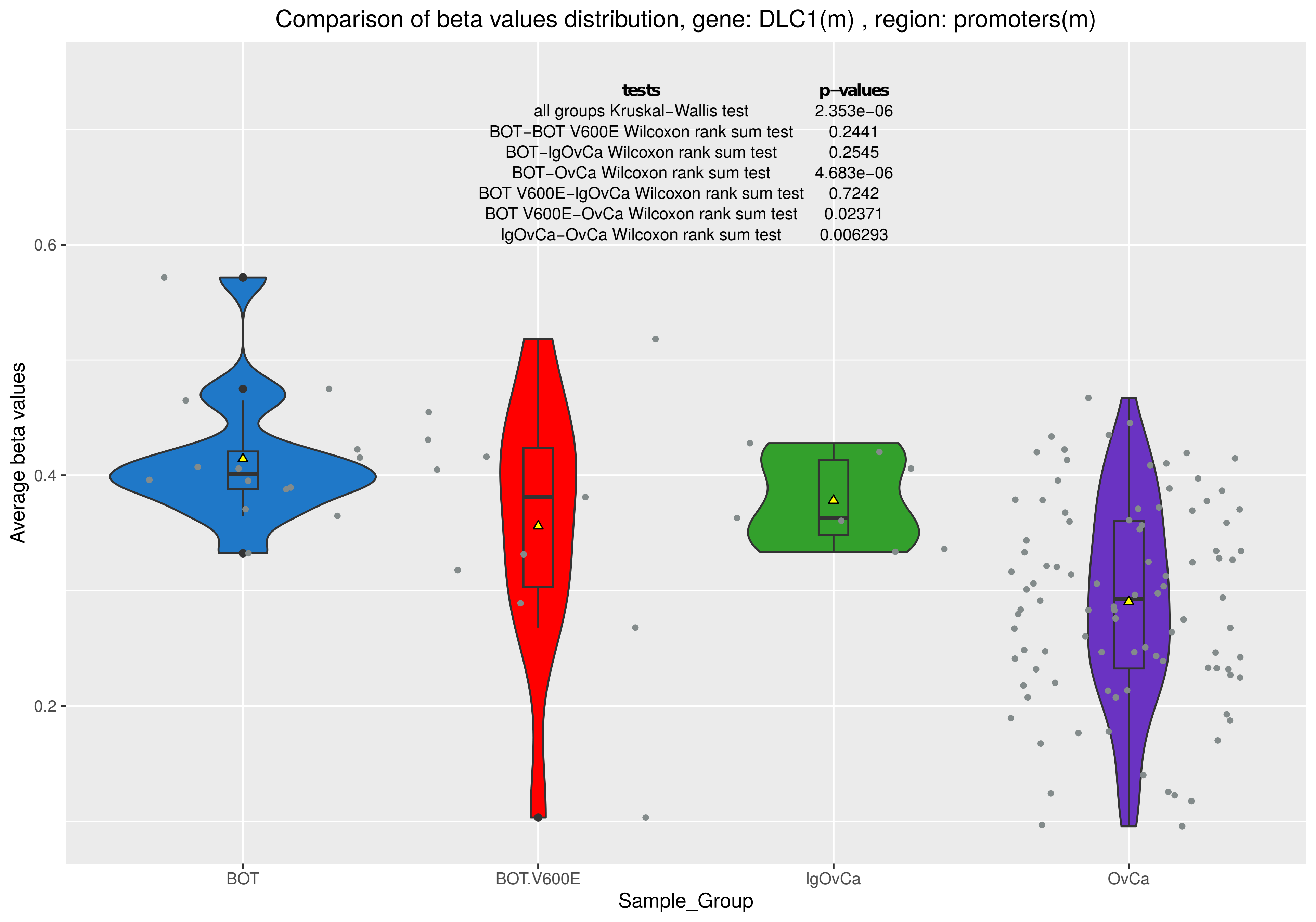
Comparison of beta values distribution, gene: DLC1(m) , region: promoters(m)
tests
p−values
all groups Kruskal−Wallis test
2.353e−06
BOT−BOT V600E Wilcoxon rank sum test
0.2441
BOT−lgOvCa Wilcoxon rank sum test
0.2545
BOT−OvCa Wilcoxon rank sum test
4.683e−06
BOT V600E−lgOvCa Wilcoxon rank sum test
0.7242
BOT V600E−OvCa Wilcoxon rank sum test
0.02371
lgOvCa−OvCa Wilcoxon rank sum test
0.006293
Average beta values
0.6
0.4
0.2
BOT
BOT.V600E
lgOvCa
OvCa
Sample_Group

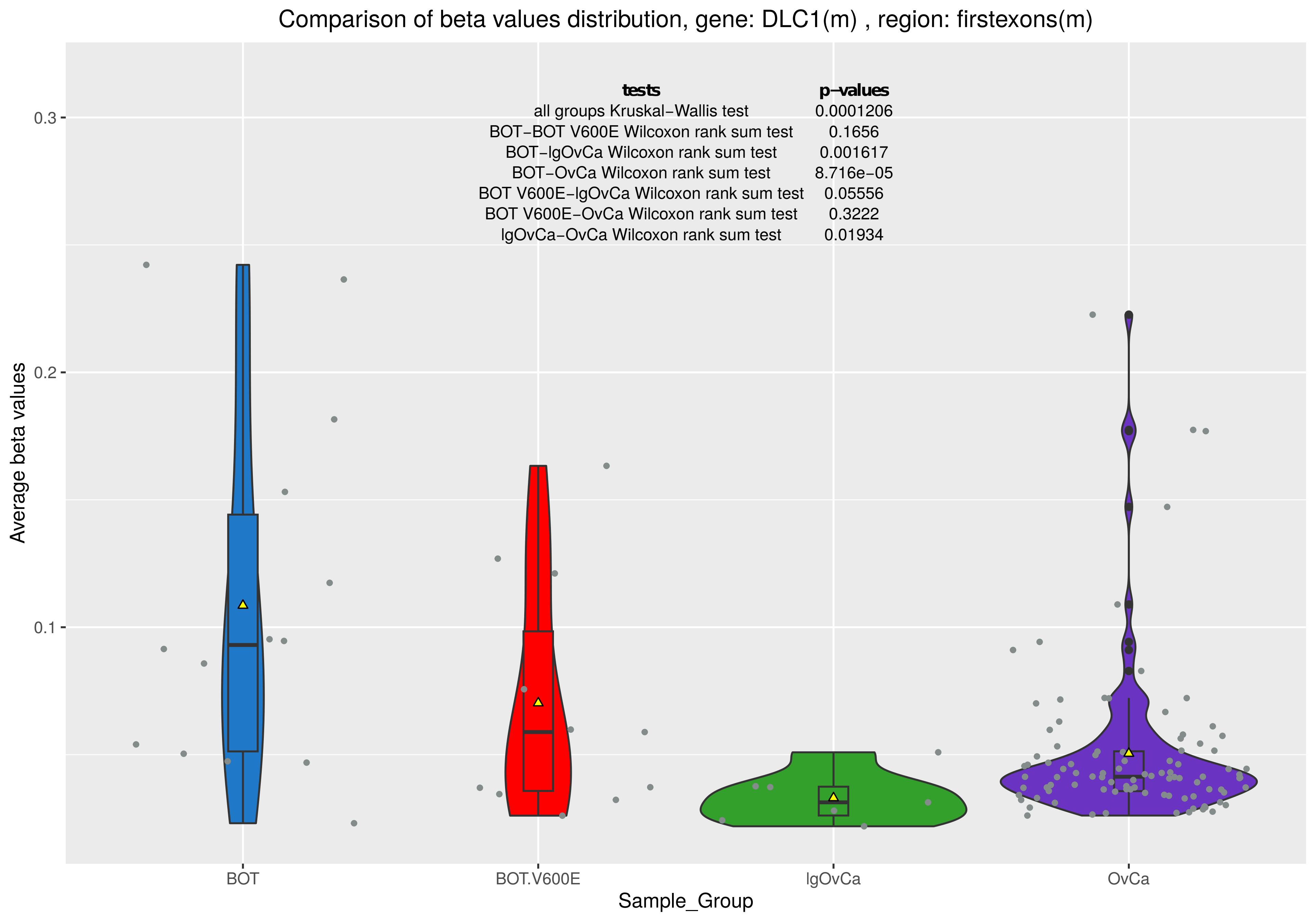

Comparison of beta values distribution, gene: DLC1(m) , region: firstexons(m)
tests
p−values
all groups Kruskal−Wallis test
0.0001206
BOT−BOT V600E Wilcoxon rank sum test
0.1656
BOT−lgOvCa Wilcoxon rank sum test
0.001617
BOT−OvCa Wilcoxon rank sum test
8.716e−05
BOT V600E−lgOvCa Wilcoxon rank sum test
0.05556
BOT V600E−OvCa Wilcoxon rank sum test
0.3222
lgOvCa−OvCa Wilcoxon rank sum test
0.01934
Average beta values
0.3
0.2
0.1
BOT
BOT.V600E
lgOvCa
OvCa
Sample_Group

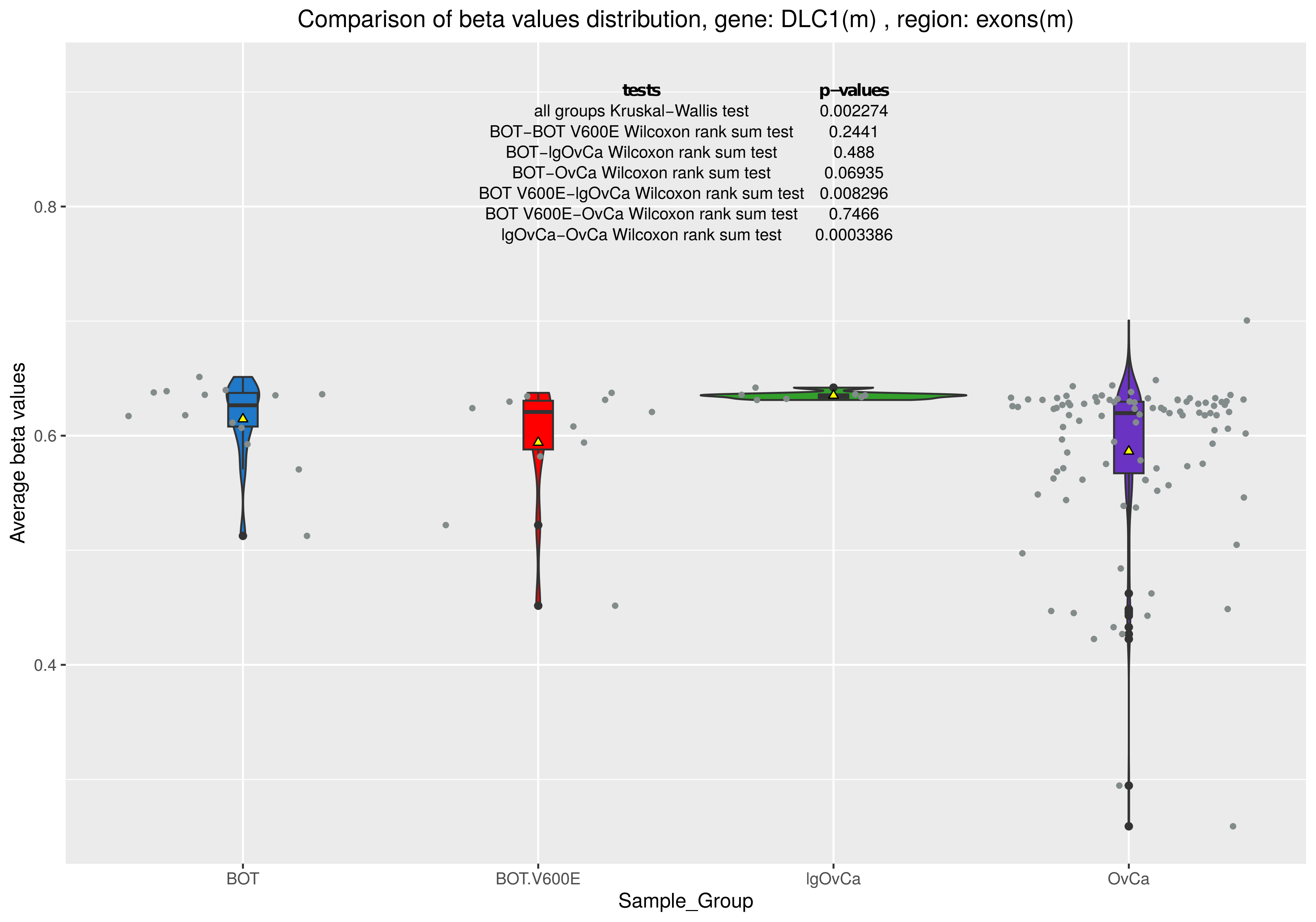

Comparison of beta values distribution, gene: DLC1(m) , region: exons(m)
tests
p−values
all groups Kruskal−Wallis test
0.002274
BOT−BOT V600E Wilcoxon rank sum test
0.2441
BOT−lgOvCa Wilcoxon rank sum test
0.488
BOT−OvCa Wilcoxon rank sum test
0.06935
BOT V600E−lgOvCa Wilcoxon rank sum test
0.008296
BOT V600E−OvCa Wilcoxon rank sum test
0.7466
lgOvCa−OvCa Wilcoxon rank sum test
0.0003386
Average beta values
0.8
0.6
0.4
BOT
BOT.V600E
lgOvCa
OvCa
Sample_Group

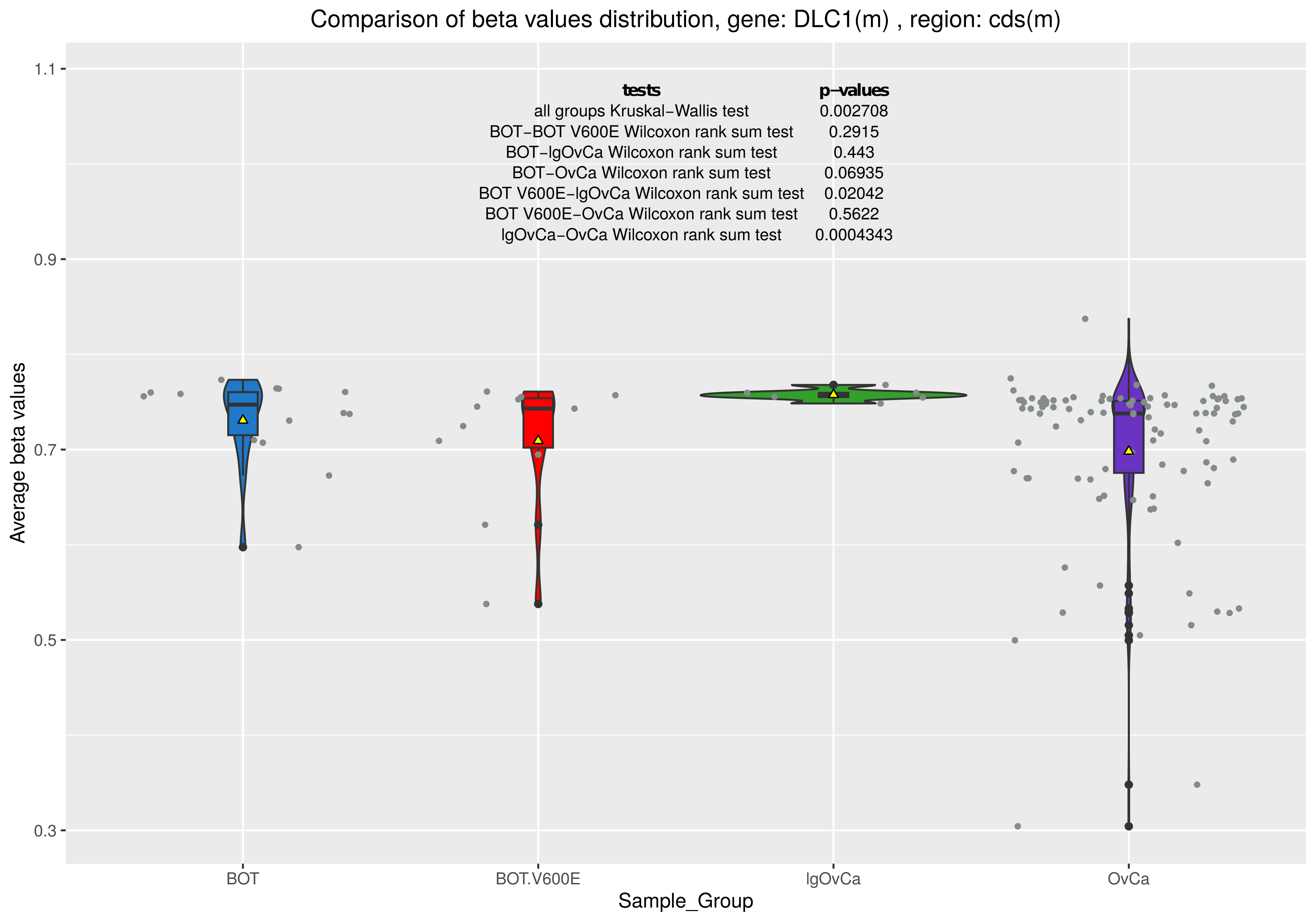
Comparison of beta values distribution, gene: DLC1(m) , region: cds(m)
tests
p−values
all groups Kruskal−Wallis test
0.002708
BOT−BOT V600E Wilcoxon rank sum test
0.2915
BOT−lgOvCa Wilcoxon rank sum test
0.443
BOT−OvCa Wilcoxon rank sum test
0.06935
BOT V600E−lgOvCa Wilcoxon rank sum test
0.02042
BOT V600E−OvCa Wilcoxon rank sum test
0.5622
lgOvCa−OvCa Wilcoxon rank sum test
0.0004343
Average beta values
1.1
0.9
0.7
0.5
0.3
BOT
BOT.V600E
lgOvCa
OvCa
Sample_Group

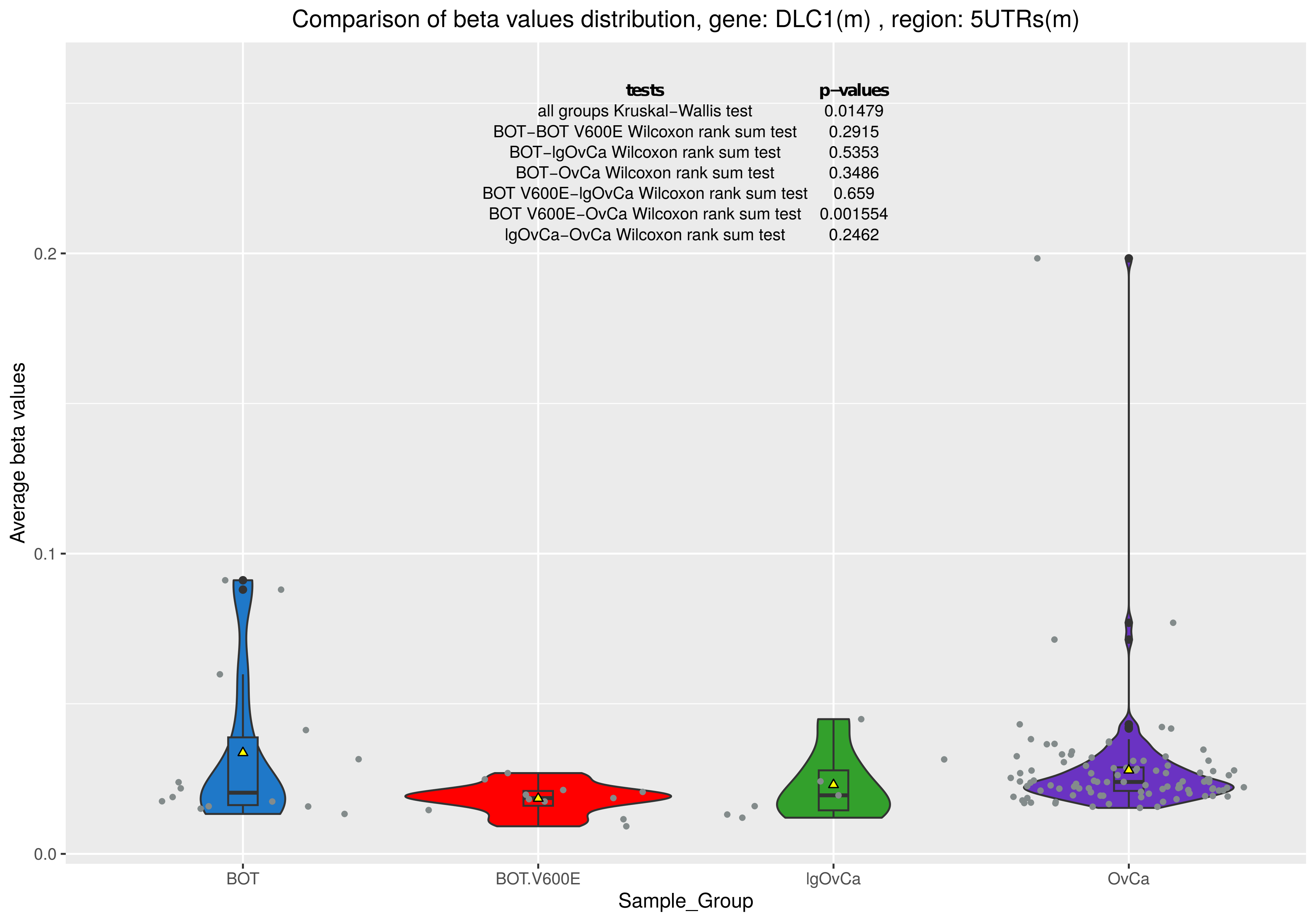
Comparison of beta values distribution, gene: DLC1(m) , region: 5UTRs(m)
tests
p−values
all groups Kruskal−Wallis test
0.01479
BOT−BOT V600E Wilcoxon rank sum test
0.2915
BOT−lgOvCa Wilcoxon rank sum test
0.5353
BOT−OvCa Wilcoxon rank sum test
0.3486
BOT V600E−lgOvCa Wilcoxon rank sum test
0.659
BOT V600E−OvCa Wilcoxon rank sum test
0.001554
lgOvCa−OvCa Wilcoxon rank sum test
0.2462
Average beta values
0.2
0.1
0.0
BOT
BOT.V600E
lgOvCa
OvCa
Sample_Group

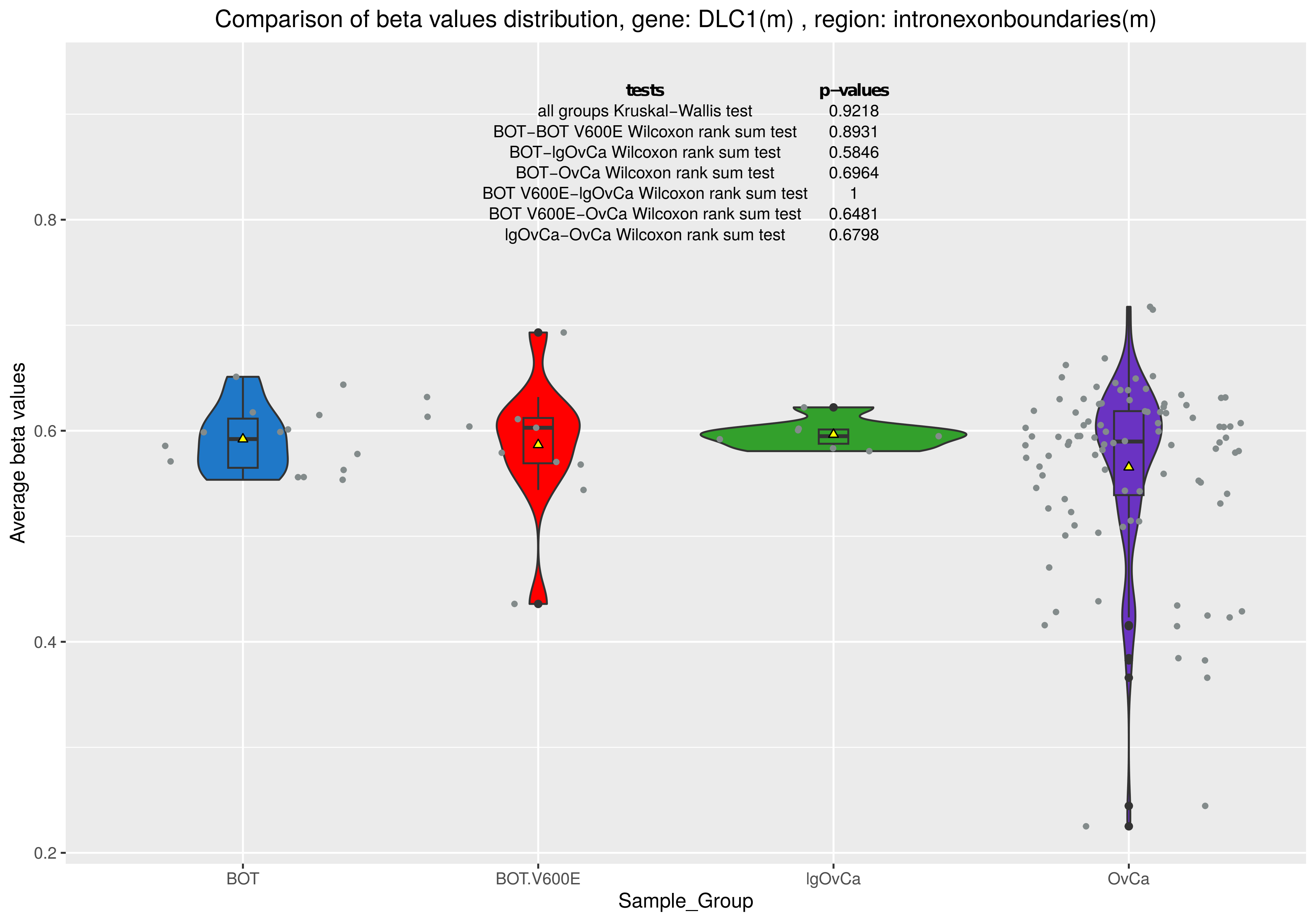
Comparison of beta values distribution, gene: DLC1(m) , region: intronexonboundaries(m)
tests
p−values
all groups Kruskal−Wallis test
0.9218
BOT−BOT V600E Wilcoxon rank sum test
0.8931
BOT−lgOvCa Wilcoxon rank sum test
0.5846
BOT−OvCa Wilcoxon rank sum test
0.6964
BOT V600E−lgOvCa Wilcoxon rank sum test
1
BOT V600E−OvCa Wilcoxon rank sum test
0.6481
lgOvCa−OvCa Wilcoxon rank sum test
0.6798
Average beta values
0.8
0.6
0.4
0.2
BOT
BOT.V600E
lgOvCa
OvCa
Sample_Group

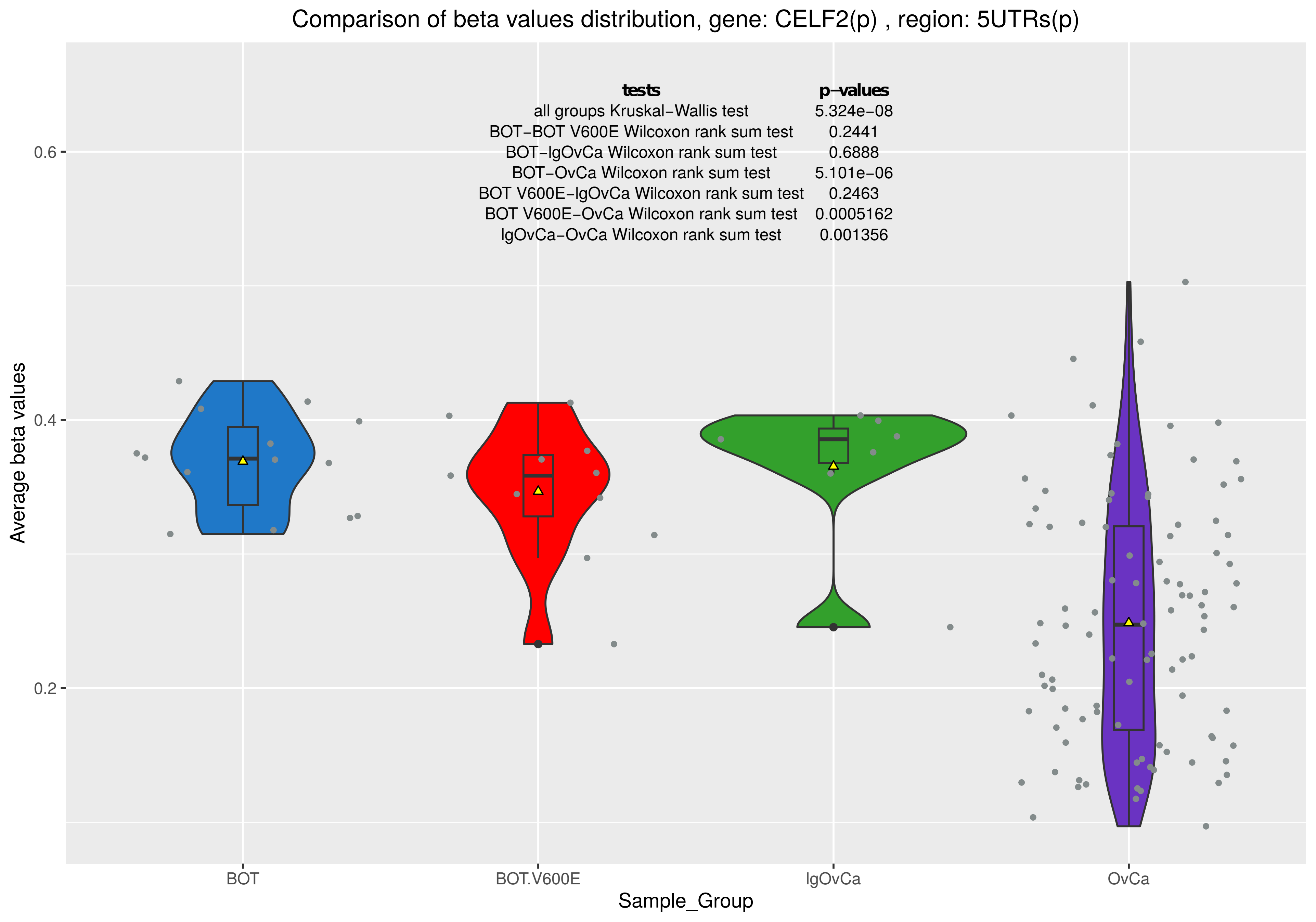
Comparison of beta values distribution, gene: CELF2(p) , region: 5UTRs(p)
tests
p−values
all groups Kruskal−Wallis test
5.324e−08
BOT−BOT V600E Wilcoxon rank sum test
0.2441
BOT−lgOvCa Wilcoxon rank sum test
0.6888
BOT−OvCa Wilcoxon rank sum test
5.101e−06
BOT V600E−lgOvCa Wilcoxon rank sum test
0.2463
BOT V600E−OvCa Wilcoxon rank sum test
0.0005162
lgOvCa−OvCa Wilcoxon rank sum test
0.001356
Average beta values
0.6
0.4
0.2
BOT
BOT.V600E
lgOvCa
OvCa
Sample_Group

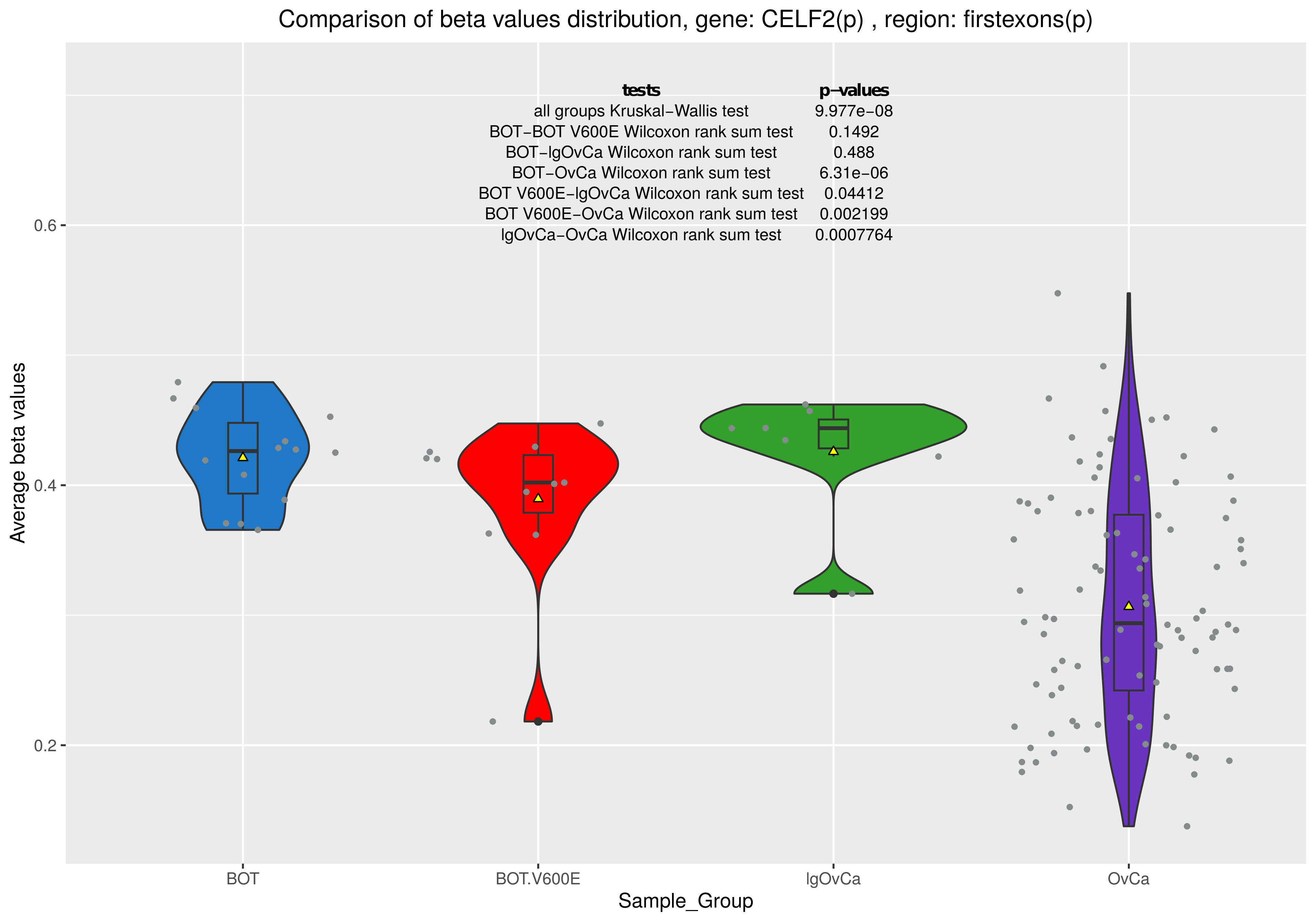
Comparison of beta values distribution, gene: CELF2(p) , region: firstexons(p)
tests
p−values
all groups Kruskal−Wallis test
9.977e−08
BOT−BOT V600E Wilcoxon rank sum test
0.1492
BOT−lgOvCa Wilcoxon rank sum test
0.488
BOT−OvCa Wilcoxon rank sum test
6.31e−06
BOT V600E−lgOvCa Wilcoxon rank sum test
0.04412
BOT V600E−OvCa Wilcoxon rank sum test
0.002199
lgOvCa−OvCa Wilcoxon rank sum test
0.0007764
Average beta values
0.6
0.4
0.2
BOT
BOT.V600E
lgOvCa
OvCa
Sample_Group

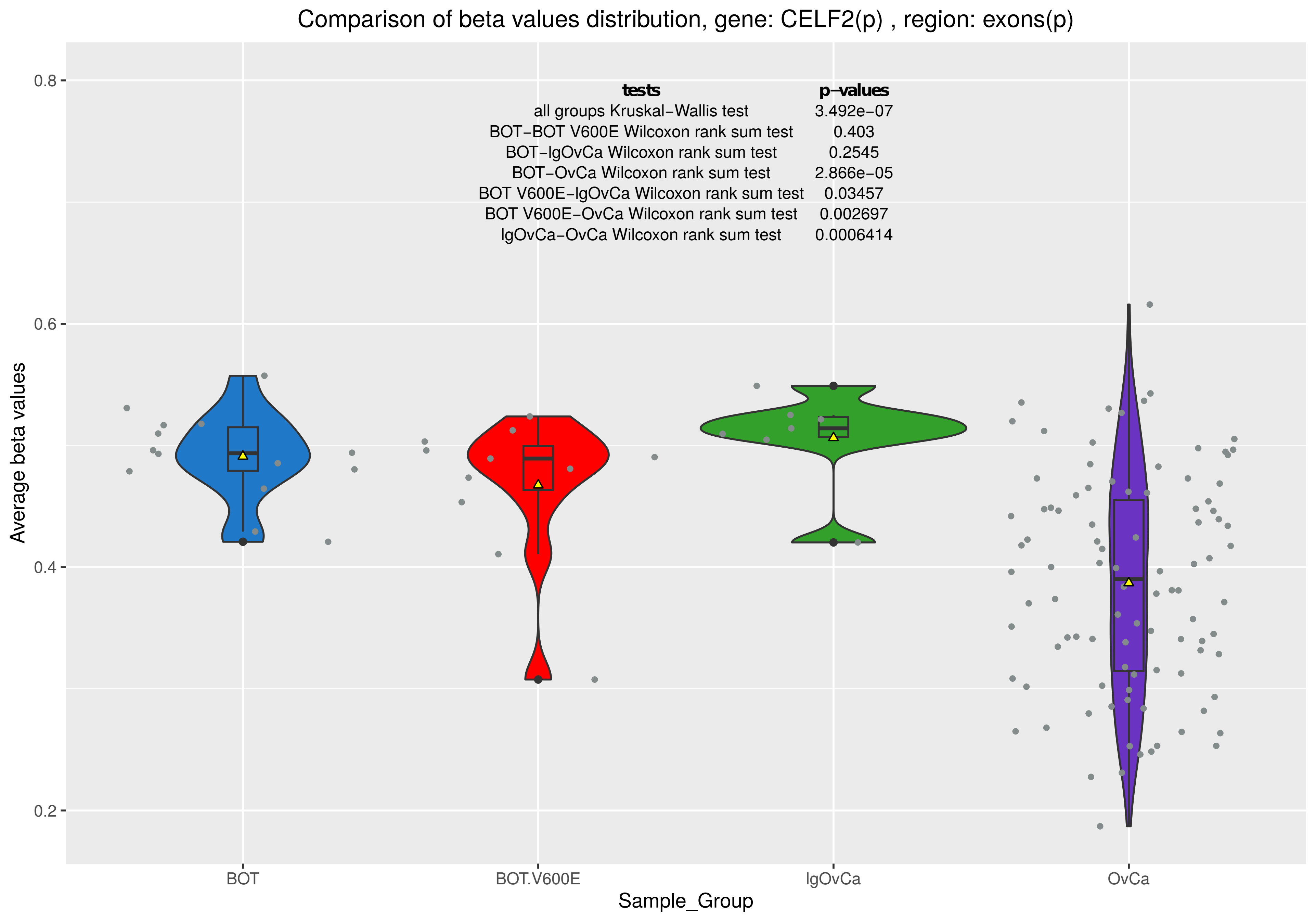
Comparison of beta values distribution, gene: CELF2(p) , region: exons(p)
tests
p−values
all groups Kruskal−Wallis test
3.492e−07
BOT−BOT V600E Wilcoxon rank sum test
0.403
BOT−lgOvCa Wilcoxon rank sum test
0.2545
BOT−OvCa Wilcoxon rank sum test
2.866e−05
BOT V600E−lgOvCa Wilcoxon rank sum test
0.03457
BOT V600E−OvCa Wilcoxon rank sum test
0.002697
lgOvCa−OvCa Wilcoxon rank sum test
0.0006414
Average beta values
0.8
0.6
0.4
0.2
BOT
BOT.V600E
lgOvCa
OvCa
Sample_Group

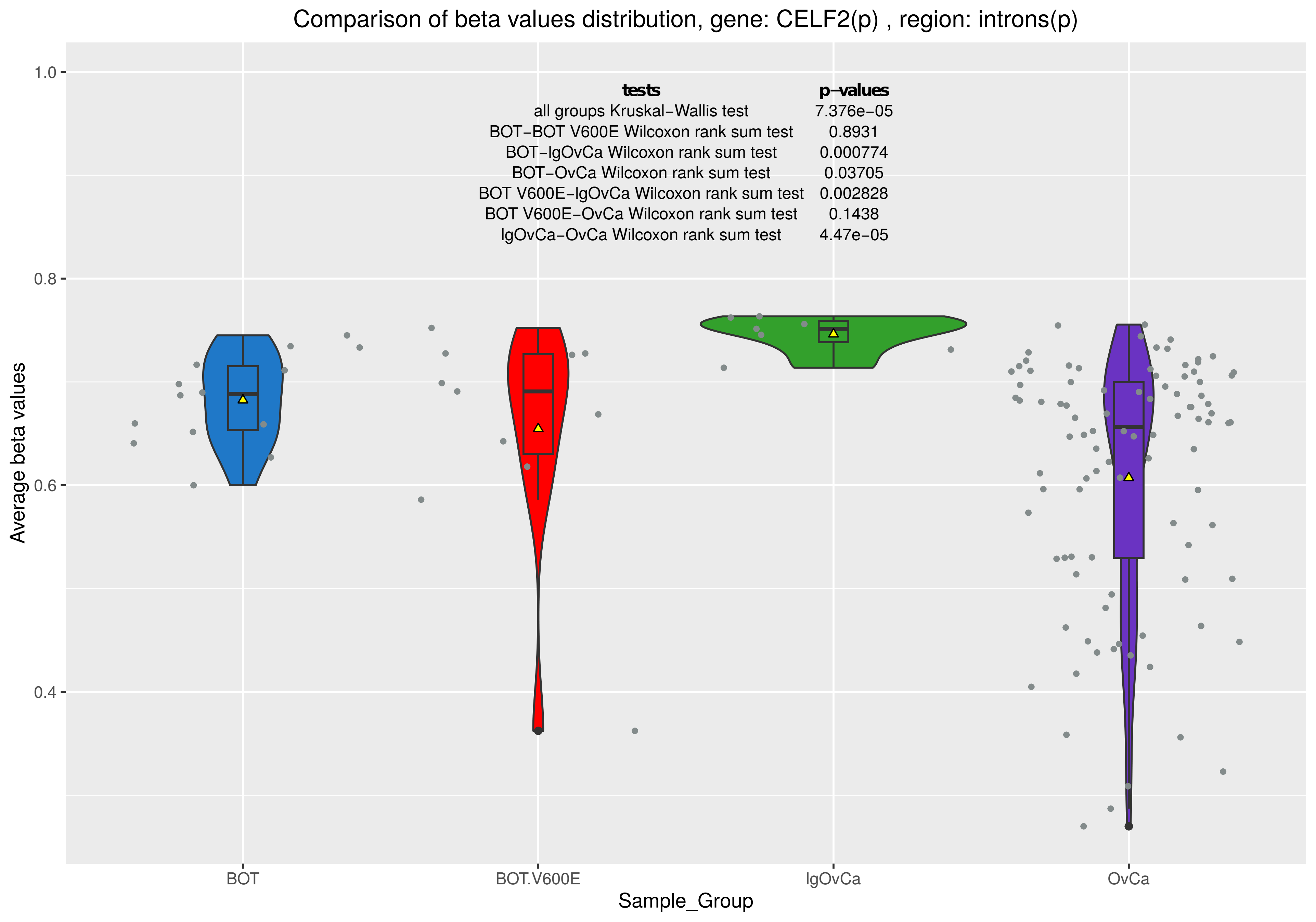

Comparison of beta values distribution, gene: CELF2(p) , region: introns(p)
tests
p−values
all groups Kruskal−Wallis test
7.376e−05
BOT−BOT V600E Wilcoxon rank sum test
0.8931
BOT−lgOvCa Wilcoxon rank sum test
0.000774
BOT−OvCa Wilcoxon rank sum test
0.03705
BOT V600E−lgOvCa Wilcoxon rank sum test
0.002828
BOT V600E−OvCa Wilcoxon rank sum test
0.1438
lgOvCa−OvCa Wilcoxon rank sum test
4.47e−05
Average beta values
1.0
0.8
0.6
0.4
BOT
BOT.V600E
lgOvCa
OvCa
Sample_Group

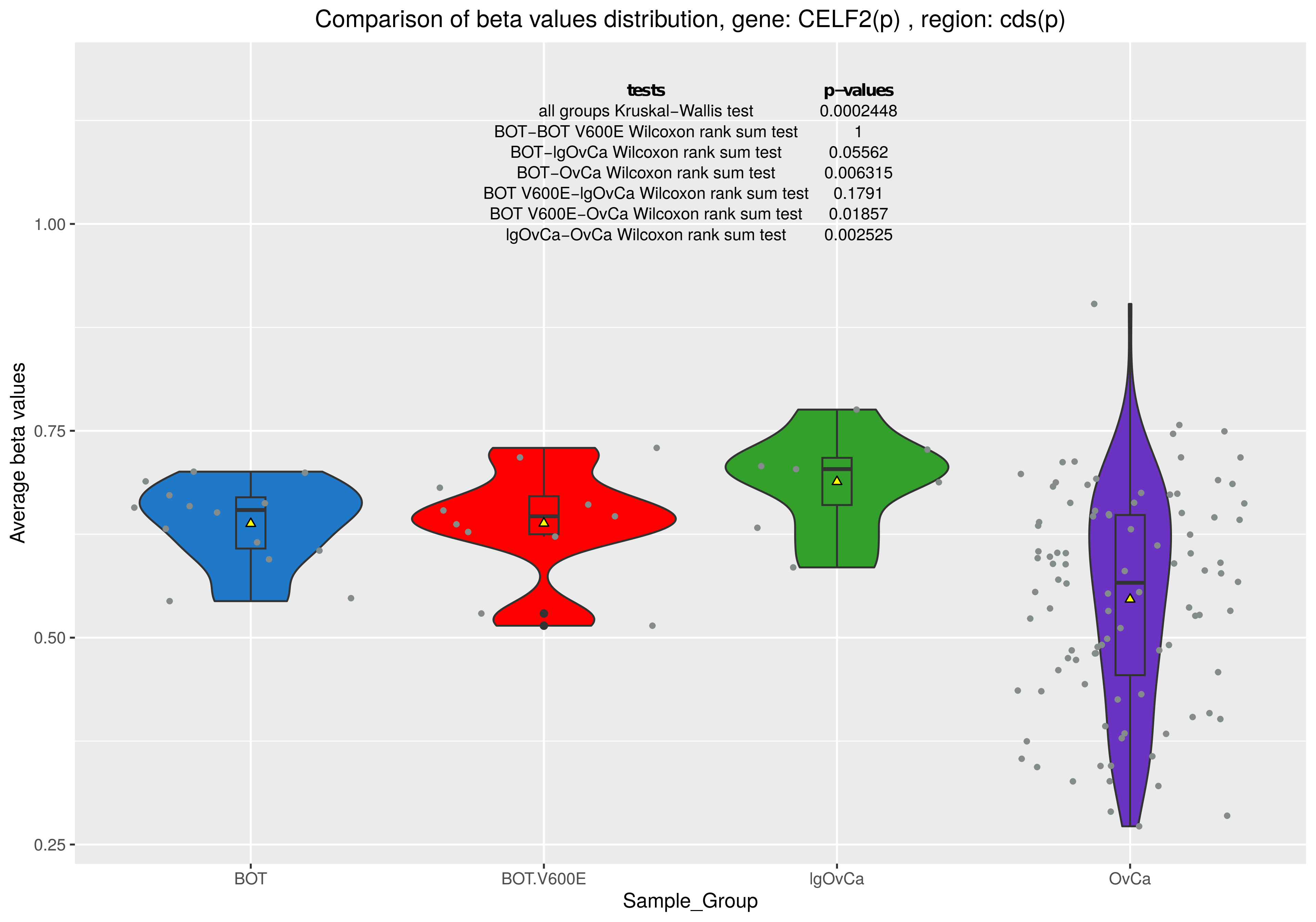

Comparison of beta values distribution, gene: CELF2(p) , region: cds(p)
tests
p−values
all groups Kruskal−Wallis test
0.0002448
BOT−BOT V600E Wilcoxon rank sum test
1
BOT−IgOvCa Wilcoxon rank sum test
0.05562
BOT−OvCa Wilcoxon rank sum test
0.006315
BOT V600E−IgOvCa Wilcoxon rank sum test
0.1791
BOT V600E−OvCa Wilcoxon rank sum test
0.01857
IgOvCa−OvCa Wilcoxon rank sum test
0.002525
Average beta values
1.00
0.75
0.50
0.25
BOT
BOT.V600E
IgOvCa
OvCa
Sample_Group

Comparison of beta values distribution, gene: CELF2(p) , region: 1to5kb(p)

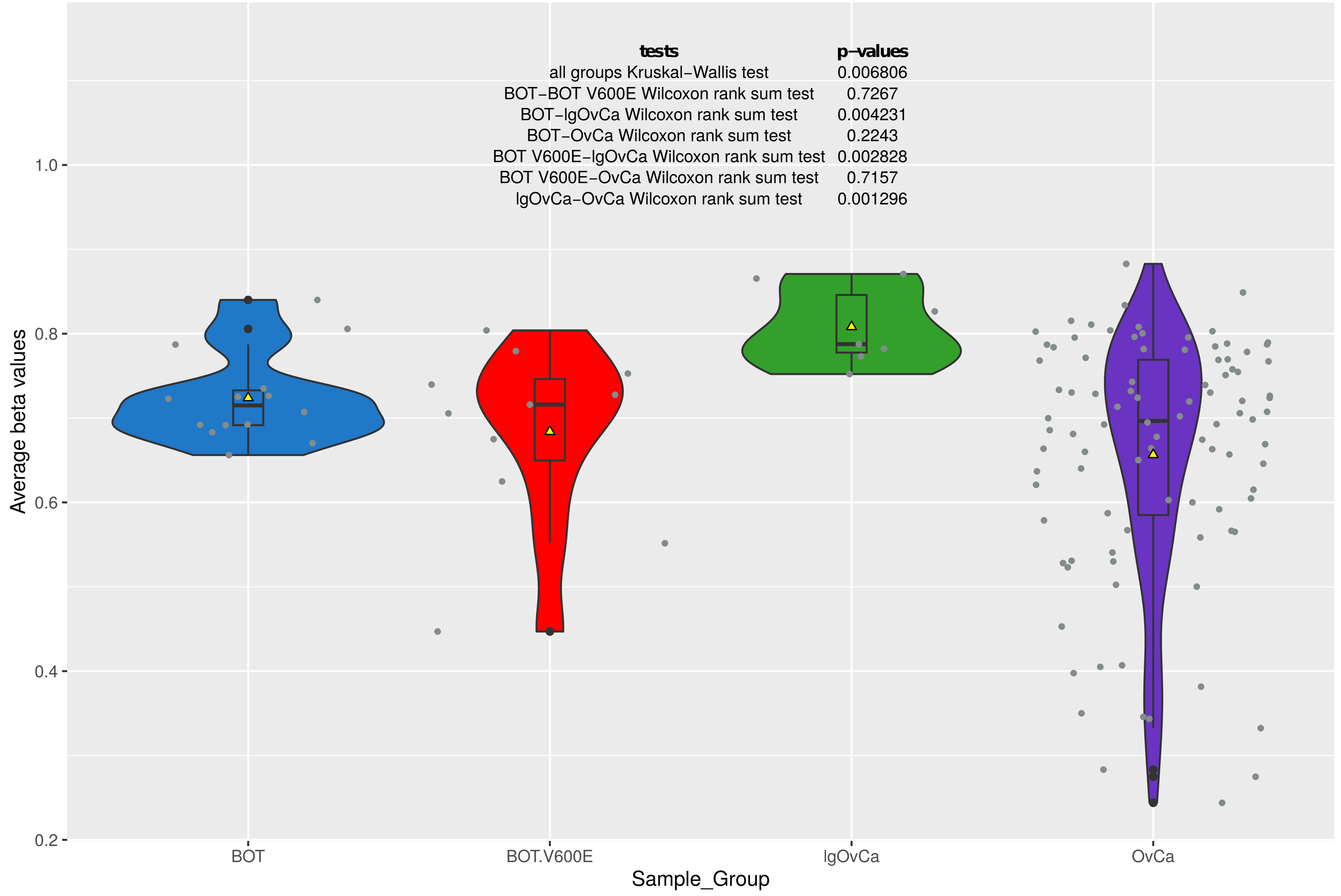

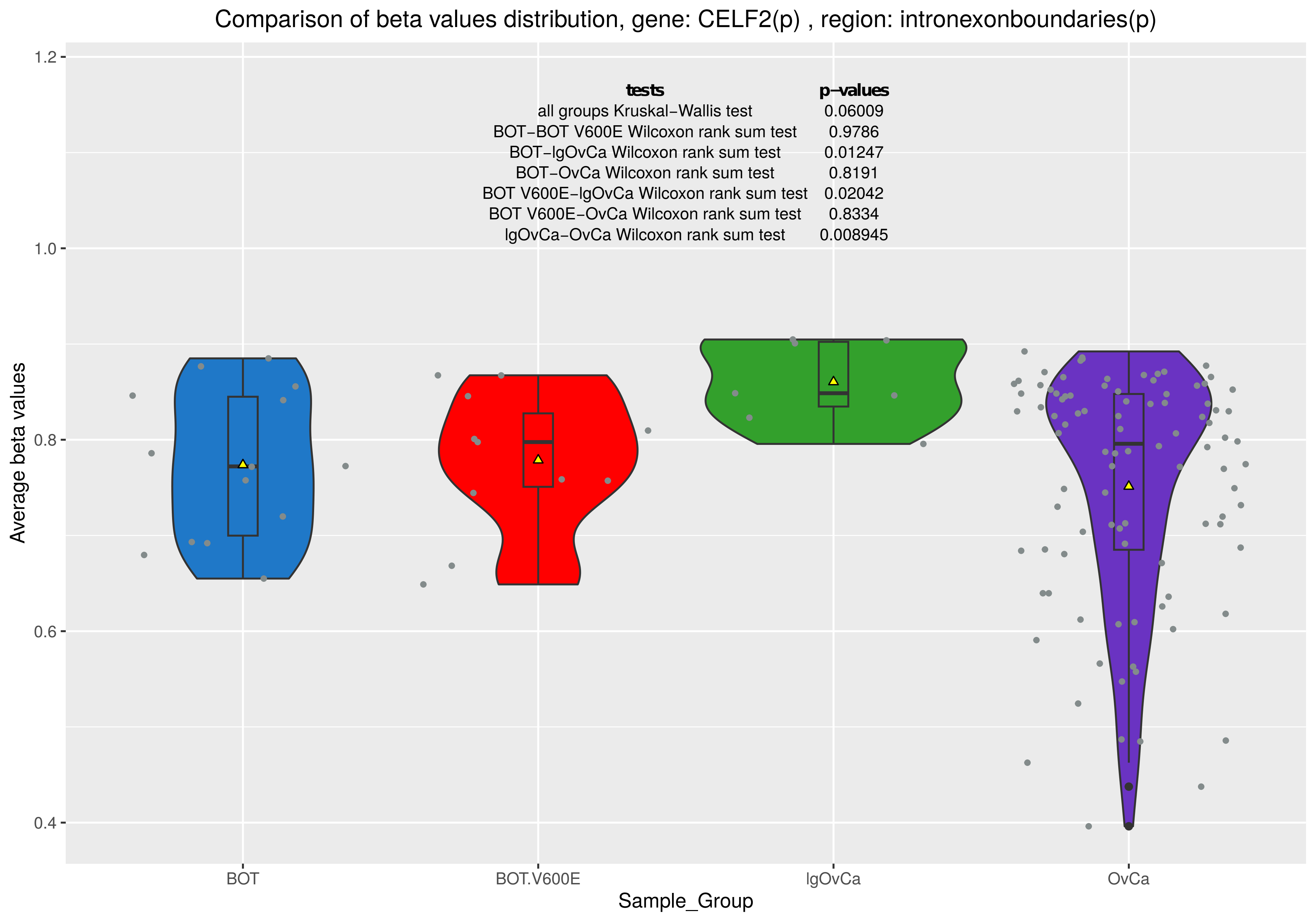

Comparison of beta values distribution, gene: CELF2(p) , region: intronexonboundaries(p)
tests
p−values
all groups Kruskal−Wallis test
0.06009
BOT−BOT V600E Wilcoxon rank sum test
0.9786
BOT−lgOvCa Wilcoxon rank sum test
0.01247
BOT−OvCa Wilcoxon rank sum test
0.8191
BOT V600E−lgOvCa Wilcoxon rank sum test
0.02042
BOT V600E−OvCa Wilcoxon rank sum test
0.8334
lgOvCa−OvCa Wilcoxon rank sum test
0.008945
Average beta values
1.2
1.0
0.8
0.6
0.4
BOT
BOT.V600E
lgOvCa
OvCa
Sample_Group

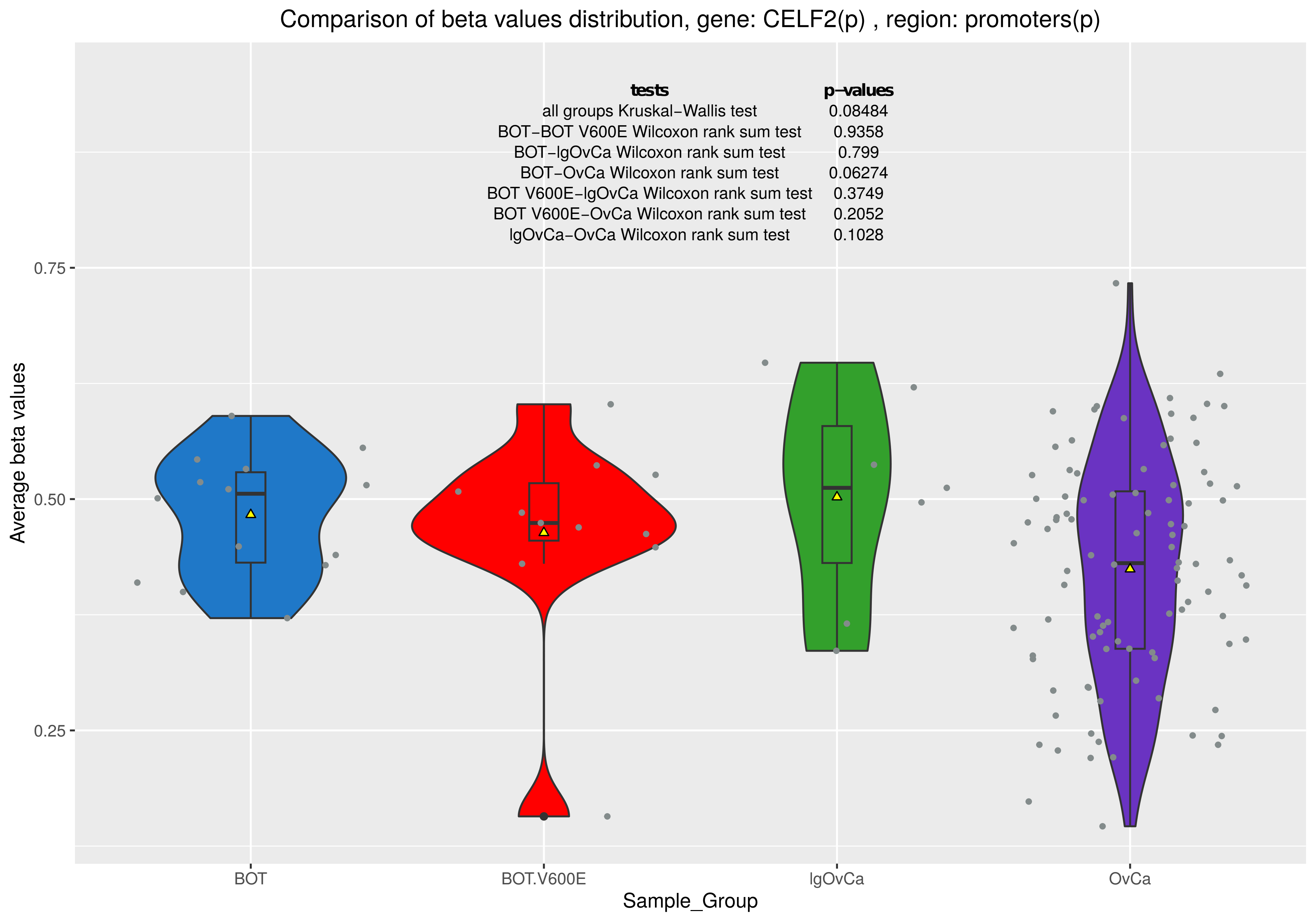

Comparison of beta values distribution, gene: CELF2(p) , region: promoters(p)
tests
p−values
all groups Kruskal−Wallis test
0.08484
BOT−BOT V600E Wilcoxon rank sum test
0.9358
BOT−lgOvCa Wilcoxon rank sum test
0.799
BOT−OvCa Wilcoxon rank sum test
0.06274
BOT V600E−lgOvCa Wilcoxon rank sum test
0.3749
BOT V600E−OvCa Wilcoxon rank sum test
0.2052
lgOvCa−OvCa Wilcoxon rank sum test
0.1028
Average beta values
0.75
0.50
0.25
BOT
BOT.V600E
lgOvCa
OvCa
Sample_Group

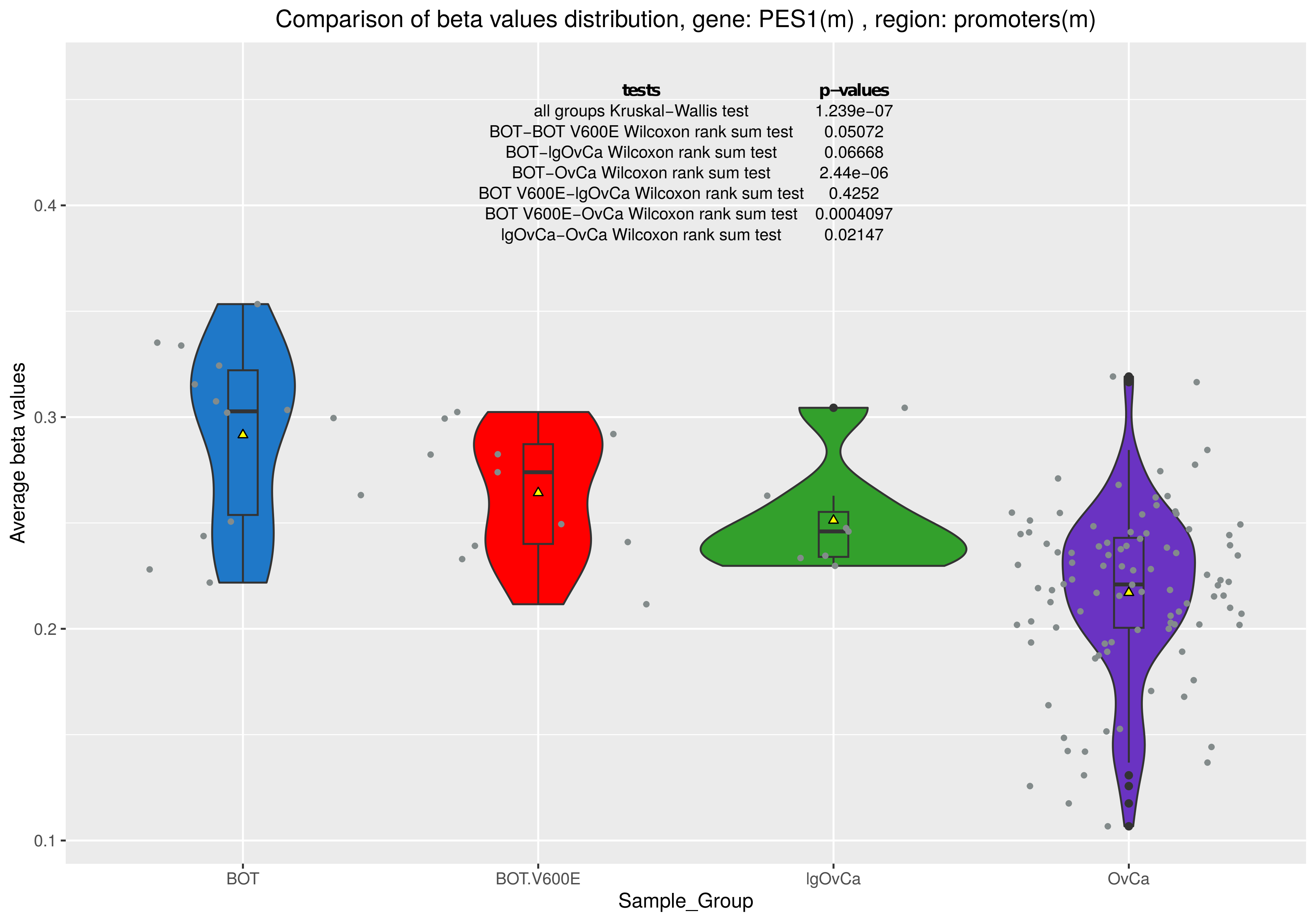

Comparison of beta values distribution, gene: PES1(m) , region: promoters(m)
tests
p−values
all groups Kruskal−Wallis test
1.239e−07
BOT−BOT V600E Wilcoxon rank sum test
0.05072
BOT−lgOvCa Wilcoxon rank sum test
0.06668
BOT−OvCa Wilcoxon rank sum test
2.44e−06
BOT V600E−lgOvCa Wilcoxon rank sum test
0.4252
BOT V600E−OvCa Wilcoxon rank sum test
0.0004097
lgOvCa−OvCa Wilcoxon rank sum test
0.02147
Average beta values
0.4
0.3
0.2
0.1
BOT
BOT.V600E
lgOvCa
OvCa
Sample_Group

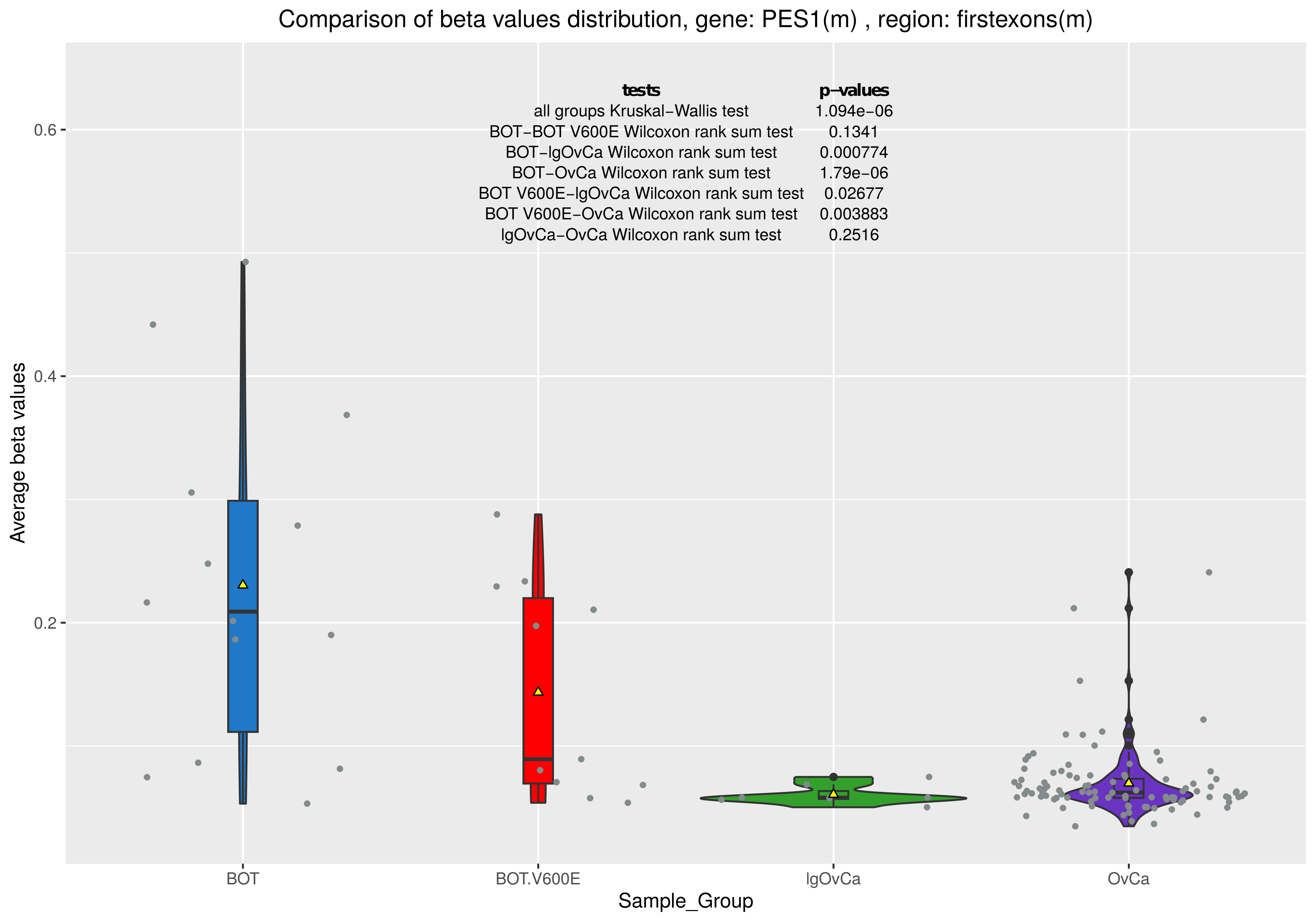
Comparison of beta values distribution, gene: PES1(m) , region: firstexons(m)
tests
p−values
all groups Kruskal−Wallis test
1.094e−06
BOT−BOT V600E Wilcoxon rank sum test
0.1341
BOT−lgOvCa Wilcoxon rank sum test
0.000774
BOT−OvCa Wilcoxon rank sum test
1.79e−06
BOT V600E−lgOvCa Wilcoxon rank sum test
0.02677
BOT V600E−OvCa Wilcoxon rank sum test
0.003883
lgOvCa−OvCa Wilcoxon rank sum test
0.2516
Average beta values
0.6
0.4
0.2
BOT
BOT.V600E
lgOvCa
OvCa
Sample_Group

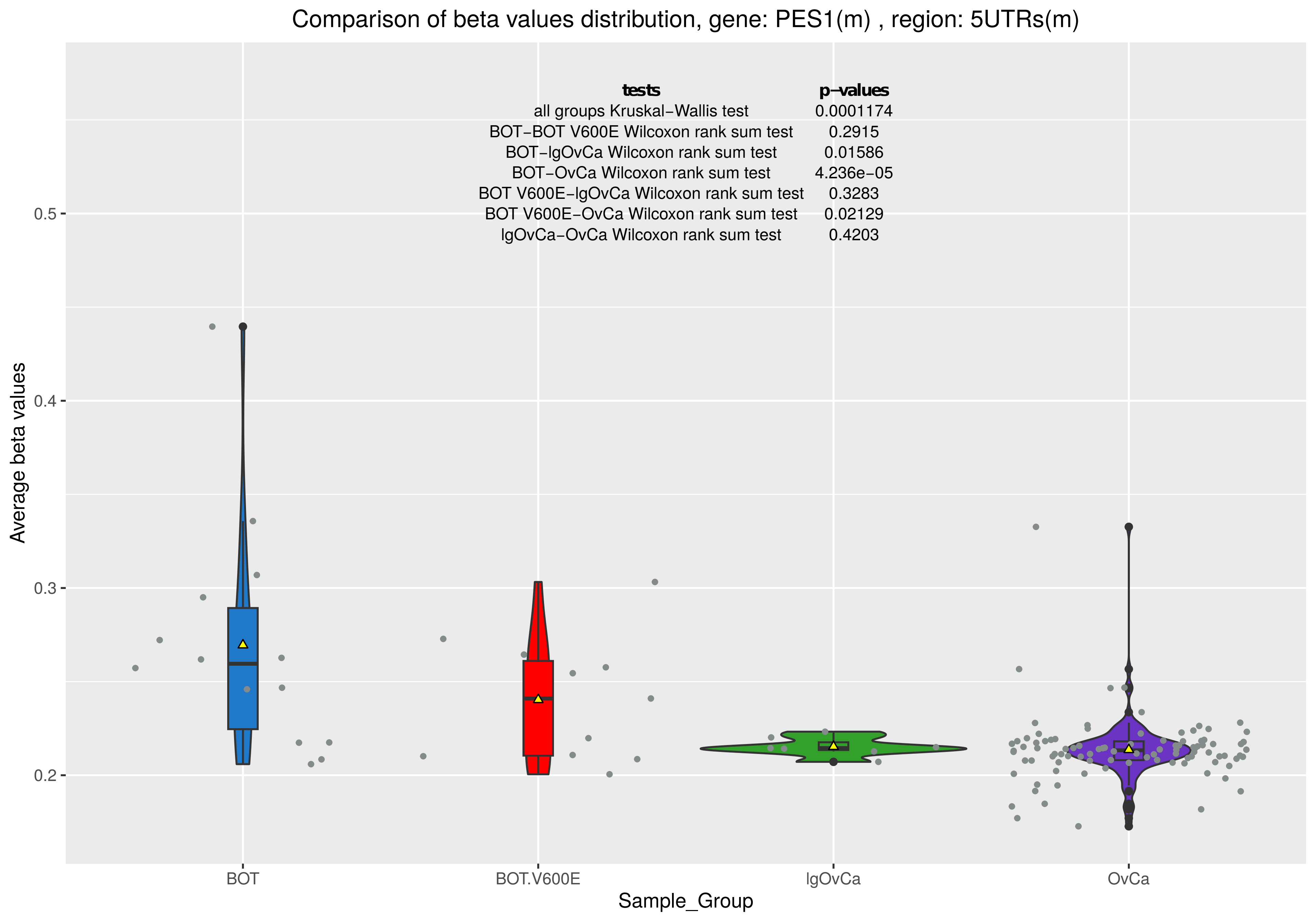

Comparison of beta values distribution, gene: PES1(m) , region: 5UTRs(m)
tests
p−values
all groups Kruskal−Wallis test
0.0001174
BOT−BOT V600E Wilcoxon rank sum test
0.2915
BOT−lgOvCa Wilcoxon rank sum test
0.01586
BOT−OvCa Wilcoxon rank sum test
4.236e−05
BOT V600E−lgOvCa Wilcoxon rank sum test
0.3283
BOT V600E−OvCa Wilcoxon rank sum test
0.02129
lgOvCa−OvCa Wilcoxon rank sum test
0.4203
Average beta values
0.5
0.4
0.3
0.2
BOT
BOT.V600E
lgOvCa
OvCa
Sample_Group

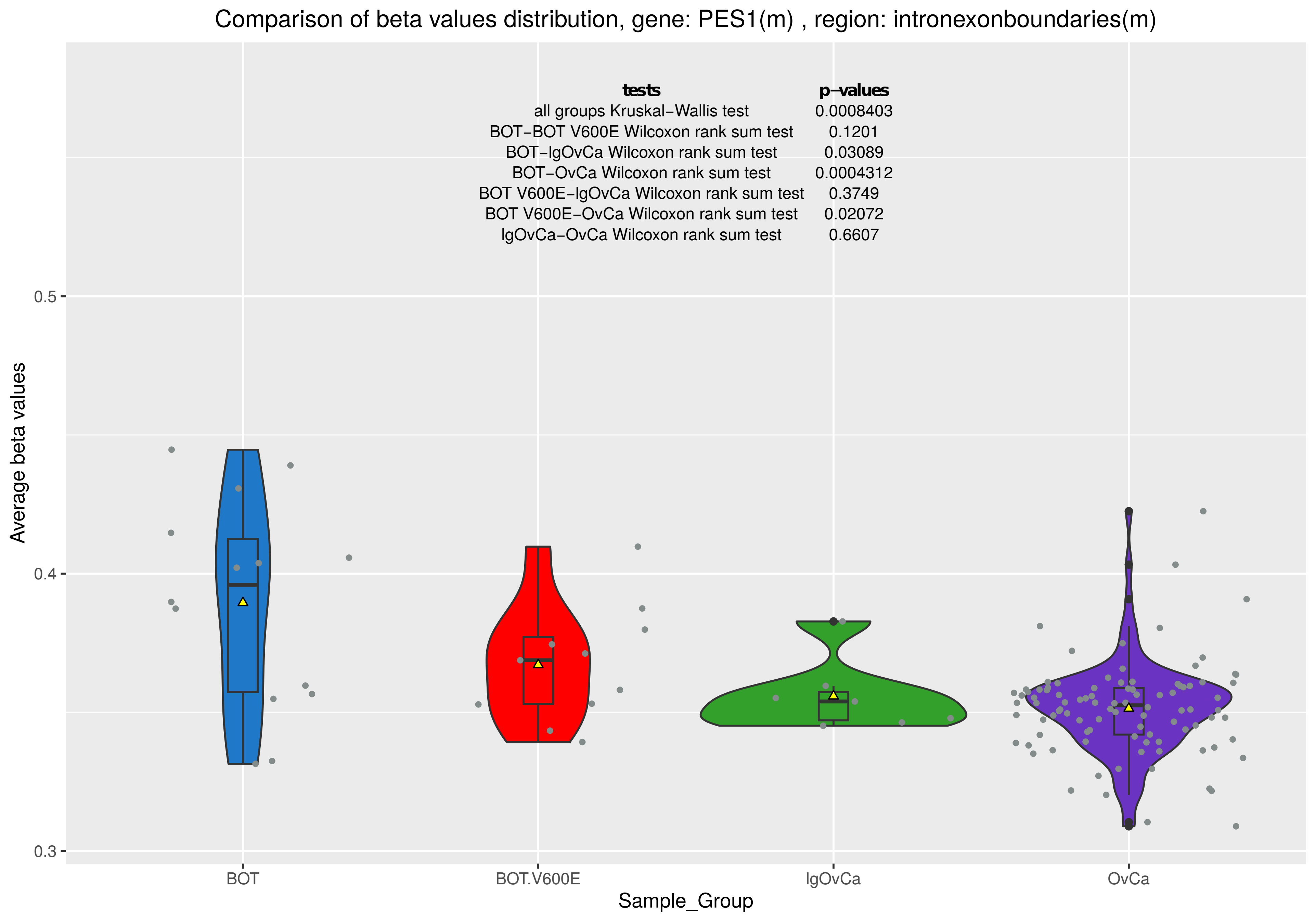

Comparison of beta values distribution, gene: PES1(m) , region: intronexonboundaries(m)
tests
p−values
all groups Kruskal−Wallis test
0.0008403
BOT−BOT V600E Wilcoxon rank sum test
0.1201
BOT−IgOvCa Wilcoxon rank sum test
0.03089
BOT−OvCa Wilcoxon rank sum test
0.0004312
BOT V600E−IgOvCa Wilcoxon rank sum test
0.3749
BOT V600E−OvCa Wilcoxon rank sum test
0.02072
IgOvCa−OvCa Wilcoxon rank sum test
0.6607
Average beta values
0.5
0.4
0.3
BOT
BOT.V600E
IgOvCa
OvCa
Sample_Group

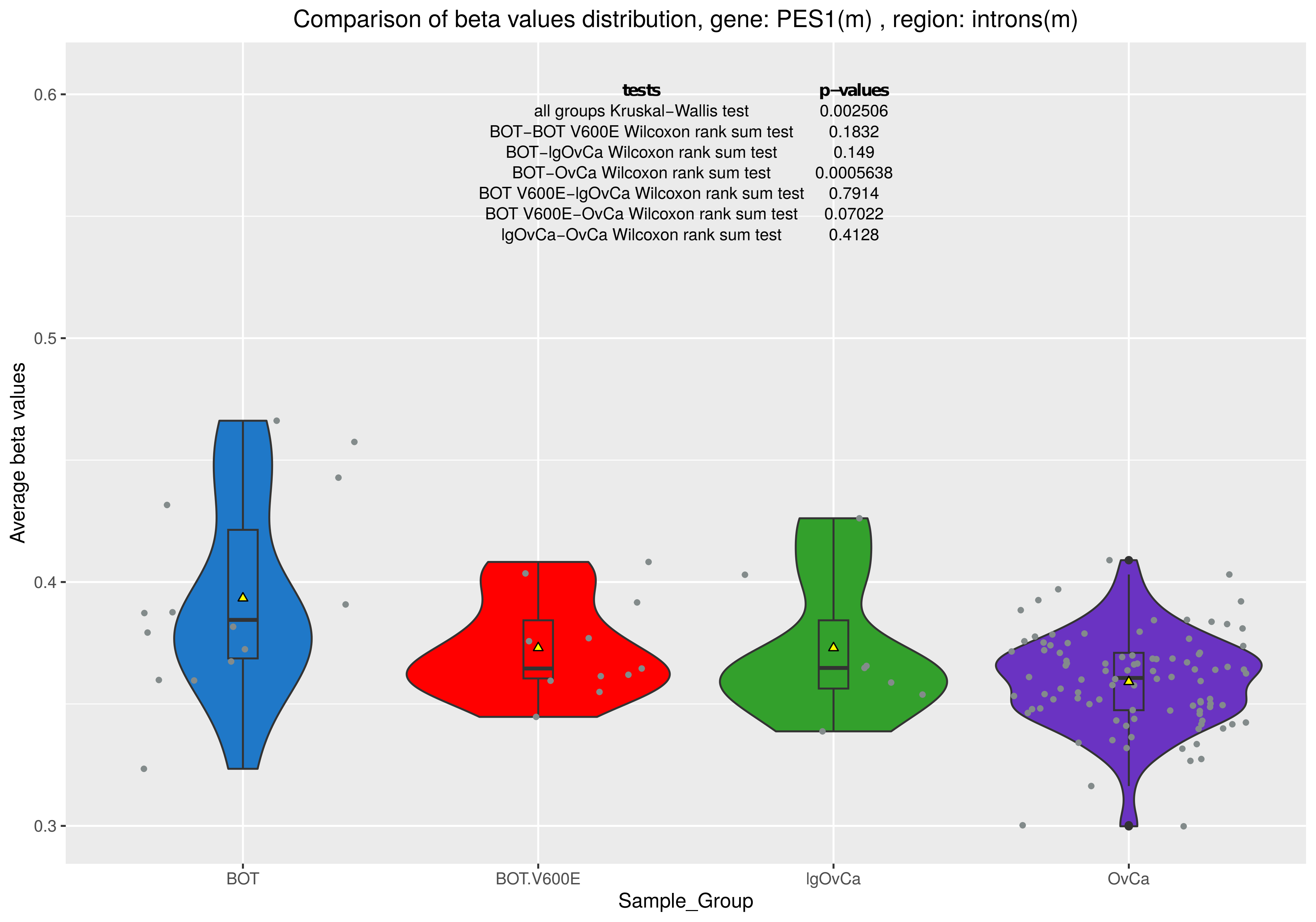
Comparison of beta values distribution, gene: PES1(m) , region: introns(m)
tests
p−values
all groups Kruskal−Wallis test
0.002506
BOT−BOT V600E Wilcoxon rank sum test
0.1832
BOT−IgOvCa Wilcoxon rank sum test
0.149
BOT−OvCa Wilcoxon rank sum test
0.0005638
BOT V600E−IgOvCa Wilcoxon rank sum test
0.7914
BOT V600E−OvCa Wilcoxon rank sum test
0.07022
IgOvCa−OvCa Wilcoxon rank sum test
0.4128
Average beta values
0.6
0.5
0.4
0.3
BOT
BOT.V600E
IgOvCa
OvCa
Sample_Group

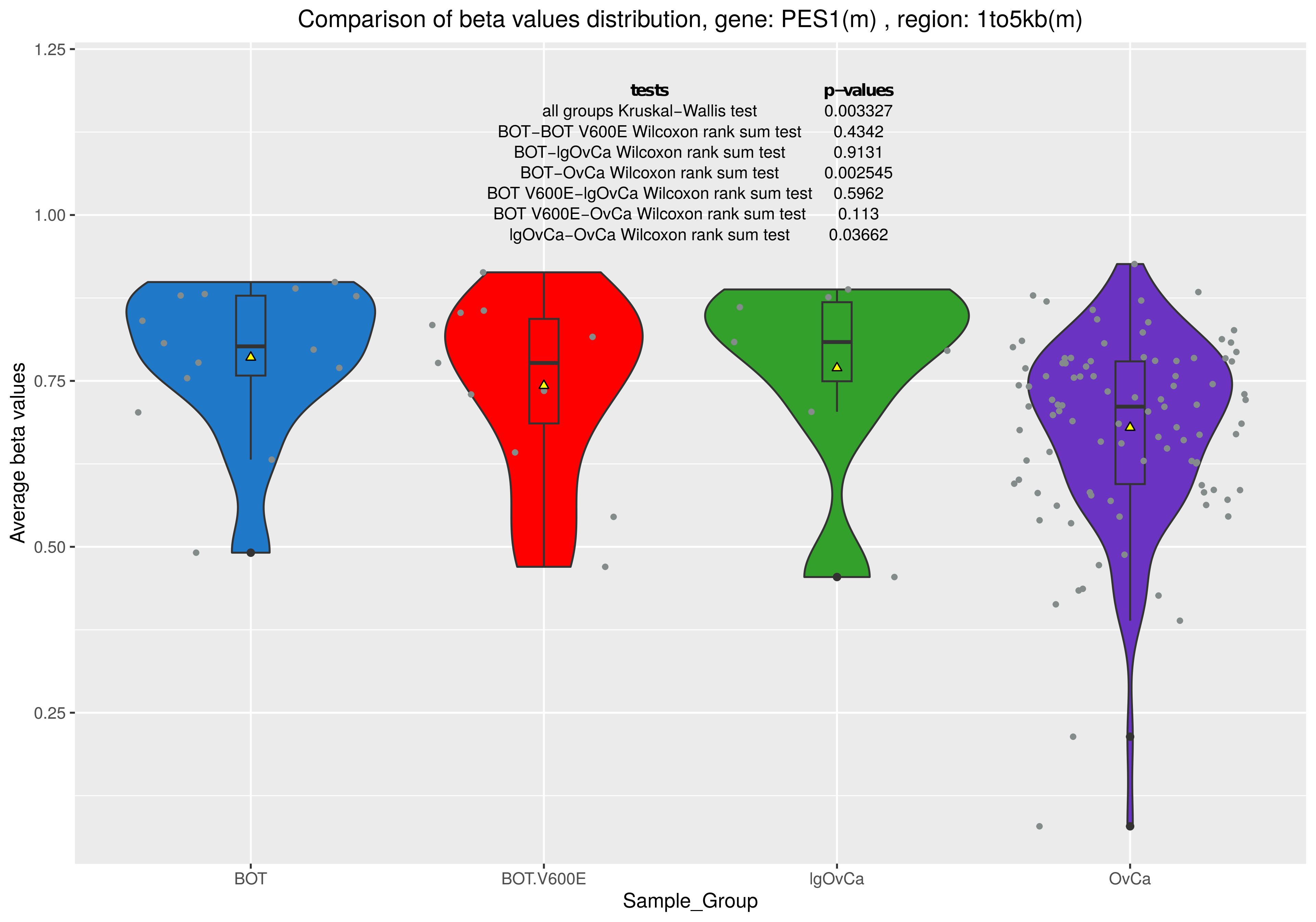

Comparison of beta values distribution, gene: PES1(m) , region: 1to5kb(m)
tests
p−values
all groups Kruskal−Wallis test
0.003327
BOT−BOT V600E Wilcoxon rank sum test
0.4342
BOT−lgOvCa Wilcoxon rank sum test
0.9131
BOT−OvCa Wilcoxon rank sum test
0.002545
BOT V600E−lgOvCa Wilcoxon rank sum test
0.5962
BOT V600E−OvCa Wilcoxon rank sum test
0.113
lgOvCa−OvCa Wilcoxon rank sum test
0.03662
Average beta values
1.25
1.00
0.75
0.50
0.25
BOT
BOT.V600E
lgOvCa
OvCa
Sample_Group

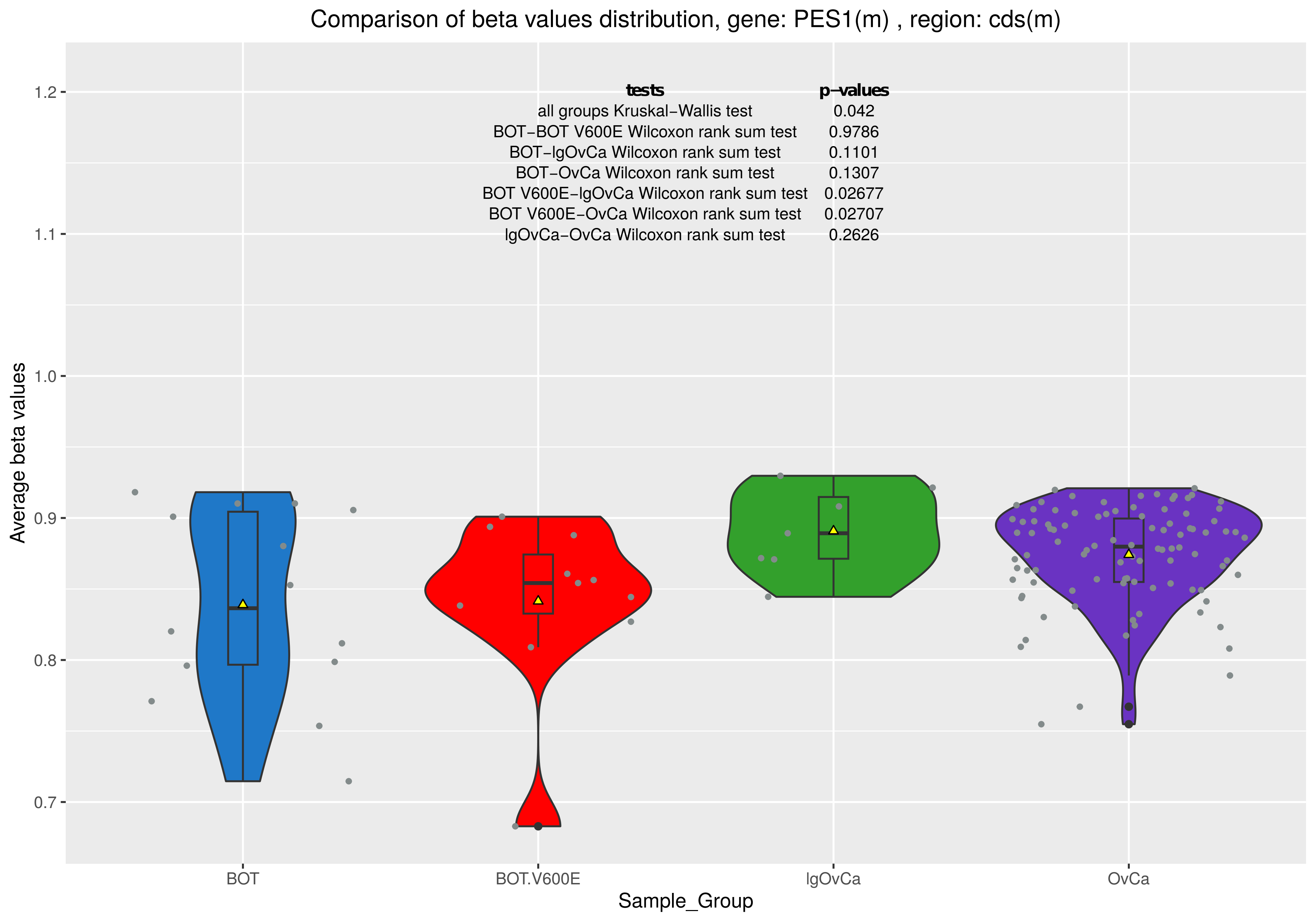

Comparison of beta values distribution, gene: PES1(m) , region: cds(m)
tests
p−values
all groups Kruskal−Wallis test
0.042
BOT−BOT V600E Wilcoxon rank sum test
0.9786
BOT−lgOvCa Wilcoxon rank sum test
0.1101
BOT−OvCa Wilcoxon rank sum test
0.1307
BOT V600E−lgOvCa Wilcoxon rank sum test
0.02677
BOT V600E−OvCa Wilcoxon rank sum test
0.02707
lgOvCa−OvCa Wilcoxon rank sum test
0.2626
Average beta values
1.2
1.1
1.0
0.9
0.8
0.7
BOT
BOT.V600E
lgOvCa
OvCa
Sample_Group

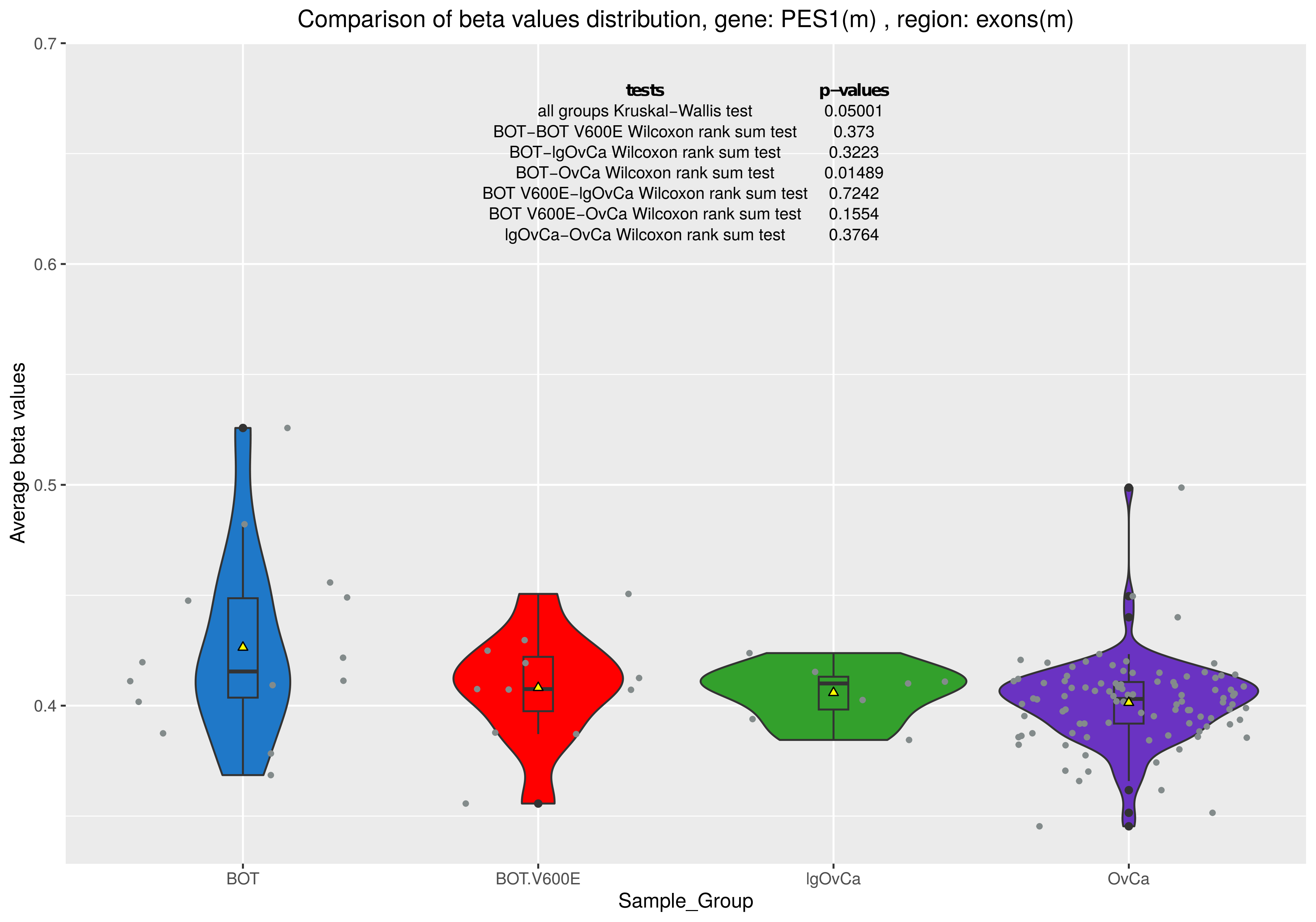

Comparison of beta values distribution, gene: PES1(m) , region: exons(m)
tests
p−values
all groups Kruskal−Wallis test
0.05001
BOT−BOT V600E Wilcoxon rank sum test
0.373
BOT−lgOvCa Wilcoxon rank sum test
0.3223
BOT−OvCa Wilcoxon rank sum test
0.01489
BOT V600E−lgOvCa Wilcoxon rank sum test
0.7242
BOT V600E−OvCa Wilcoxon rank sum test
0.1554
lgOvCa−OvCa Wilcoxon rank sum test
0.3764
Average beta values
0.7
0.6
0.5
0.4
BOT
BOT.V600E
lgOvCa
OvCa
Sample_Group

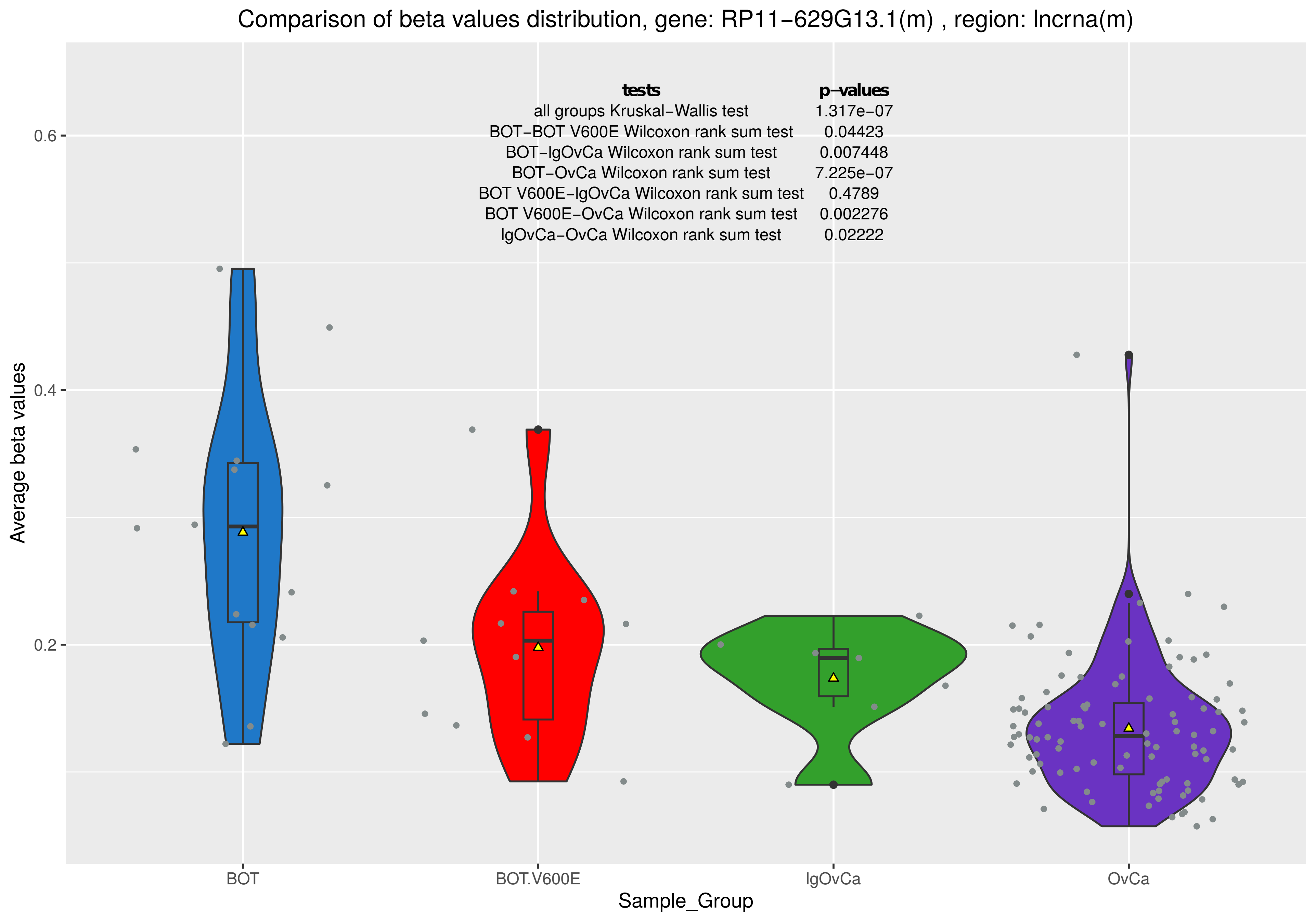

Comparison of beta values distribution, gene: RP11−629G13.1(m) , region: lncrna(m)
tests
p−values
all groups Kruskal−Wallis test
1.317e−07
BOT−BOT V600E Wilcoxon rank sum test
0.04423
BOT−lgOvCa Wilcoxon rank sum test
0.007448
BOT−OvCa Wilcoxon rank sum test
7.225e−07
BOT V600E−lgOvCa Wilcoxon rank sum test
0.4789
BOT V600E−OvCa Wilcoxon rank sum test
0.002276
lgOvCa−OvCa Wilcoxon rank sum test
0.02222
Average beta values
0.6
0.4
0.2
BOT
BOT.V600E
lgOvCa
OvCa
Sample_Group

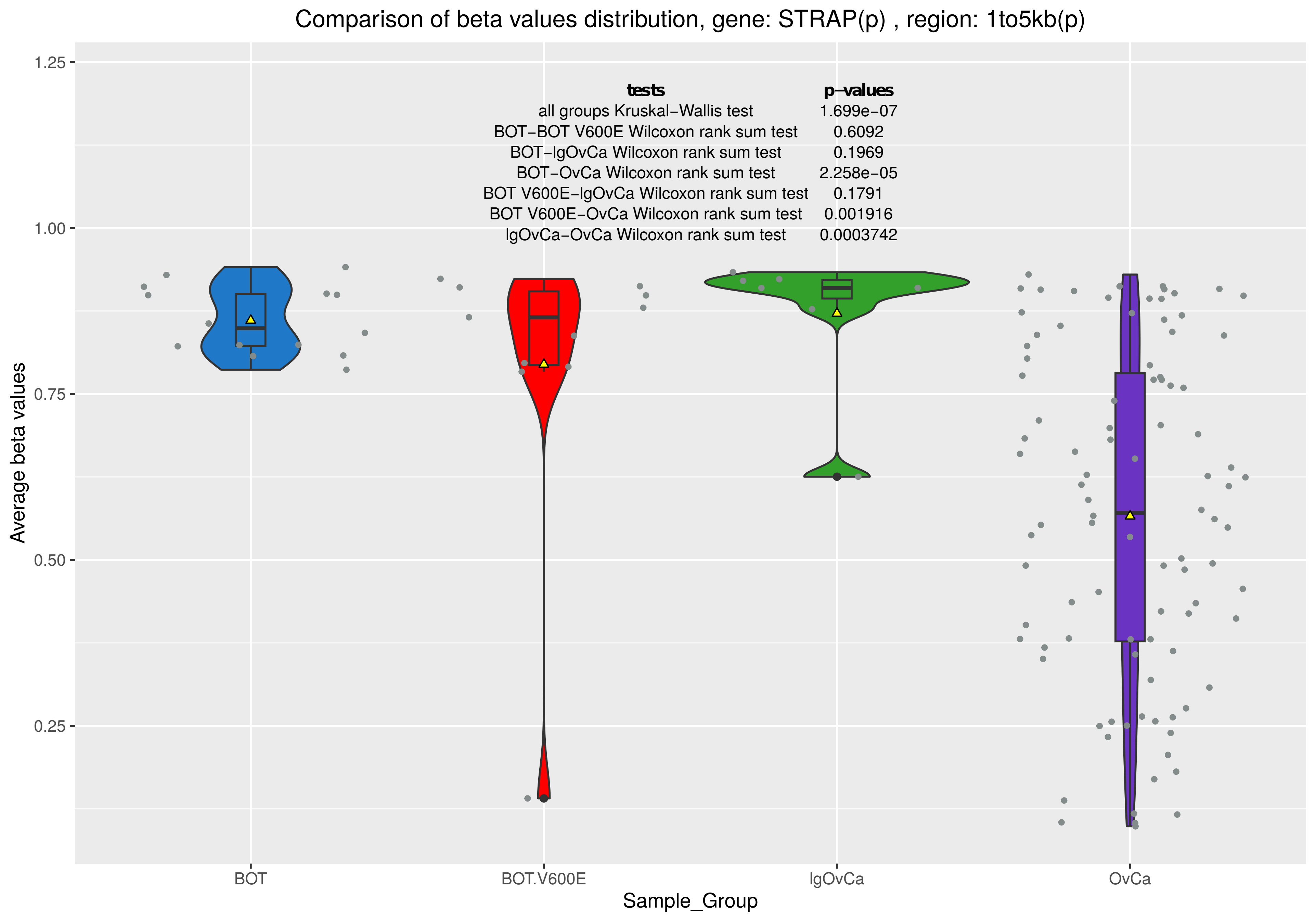

Comparison of beta values distribution, gene: STRAP(p) , region: 1to5kb(p)
tests
p−values
all groups Kruskal−Wallis test 1.699e−07
BOT−BOT V600E Wilcoxon rank sum test 0.6092
BOT−IgOvCa Wilcoxon rank sum test 0.1969
BOT−OvCa Wilcoxon rank sum test 2.258e−05
BOT V600E−IgOvCa Wilcoxon rank sum test 0.1791
BOT V600E−OvCa Wilcoxon rank sum test 0.001916
IgOvCa−OvCa Wilcoxon rank sum test 0.0003742
Average beta values
1.25
1.00
0.75
0.50
0.25
BOT
BOT.V600E
IgOvCa
OvCa
Sample_Group

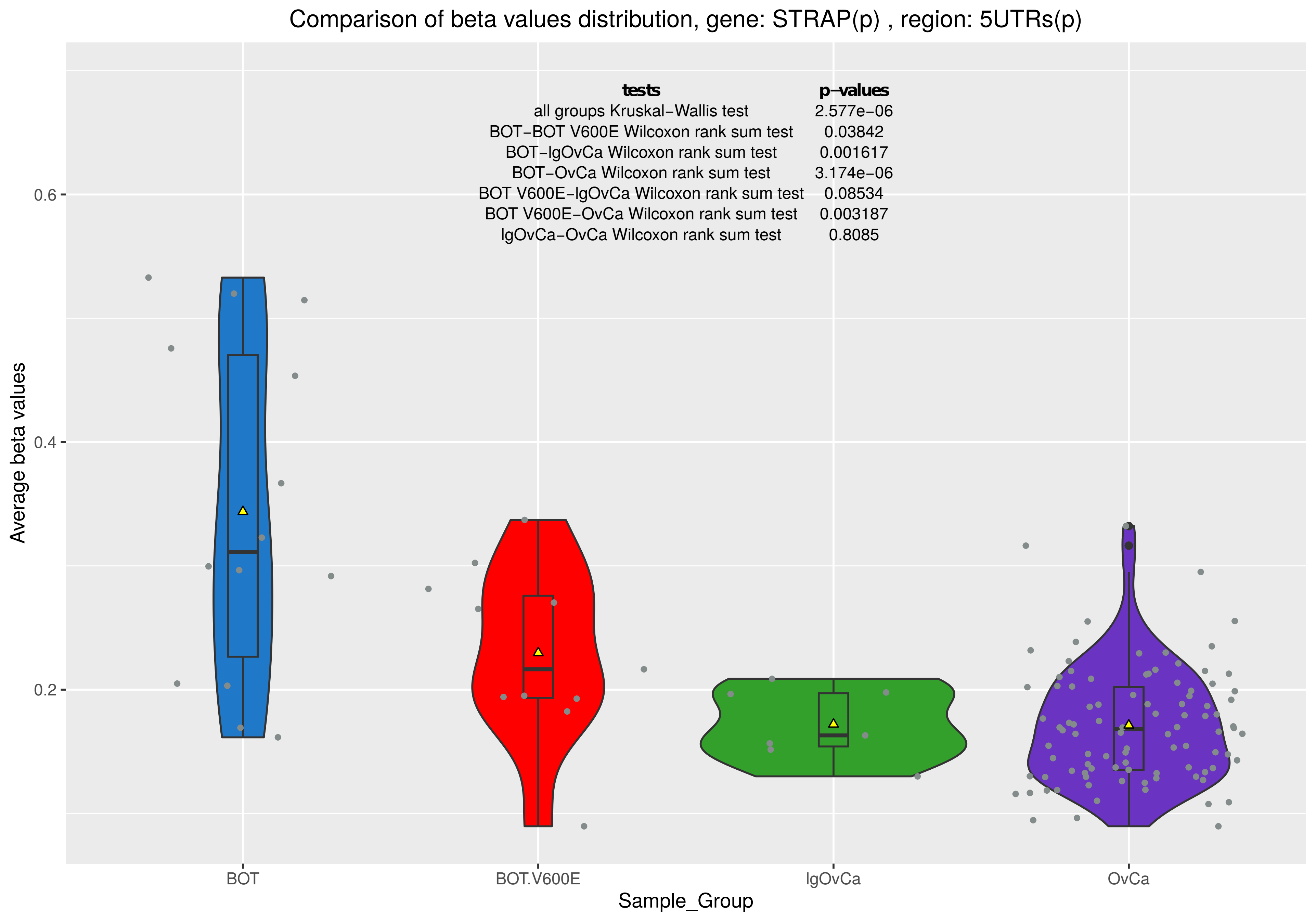
Comparison of beta values distribution, gene: STRAP(p) , region: 5UTRs(p)
tests
p−values
all groups Kruskal−Wallis test
2.577e−06
BOT−BOT V600E Wilcoxon rank sum test
0.03842
BOT−lgOvCa Wilcoxon rank sum test
0.001617
BOT−OvCa Wilcoxon rank sum test
3.174e−06
BOT V600E−lgOvCa Wilcoxon rank sum test
0.08534
BOT V600E−OvCa Wilcoxon rank sum test
0.003187
lgOvCa−OvCa Wilcoxon rank sum test
0.8085
Average beta values
0.6
0.4
0.2
BOT
BOT.V600E
lgOvCa
OvCa
Sample_Group

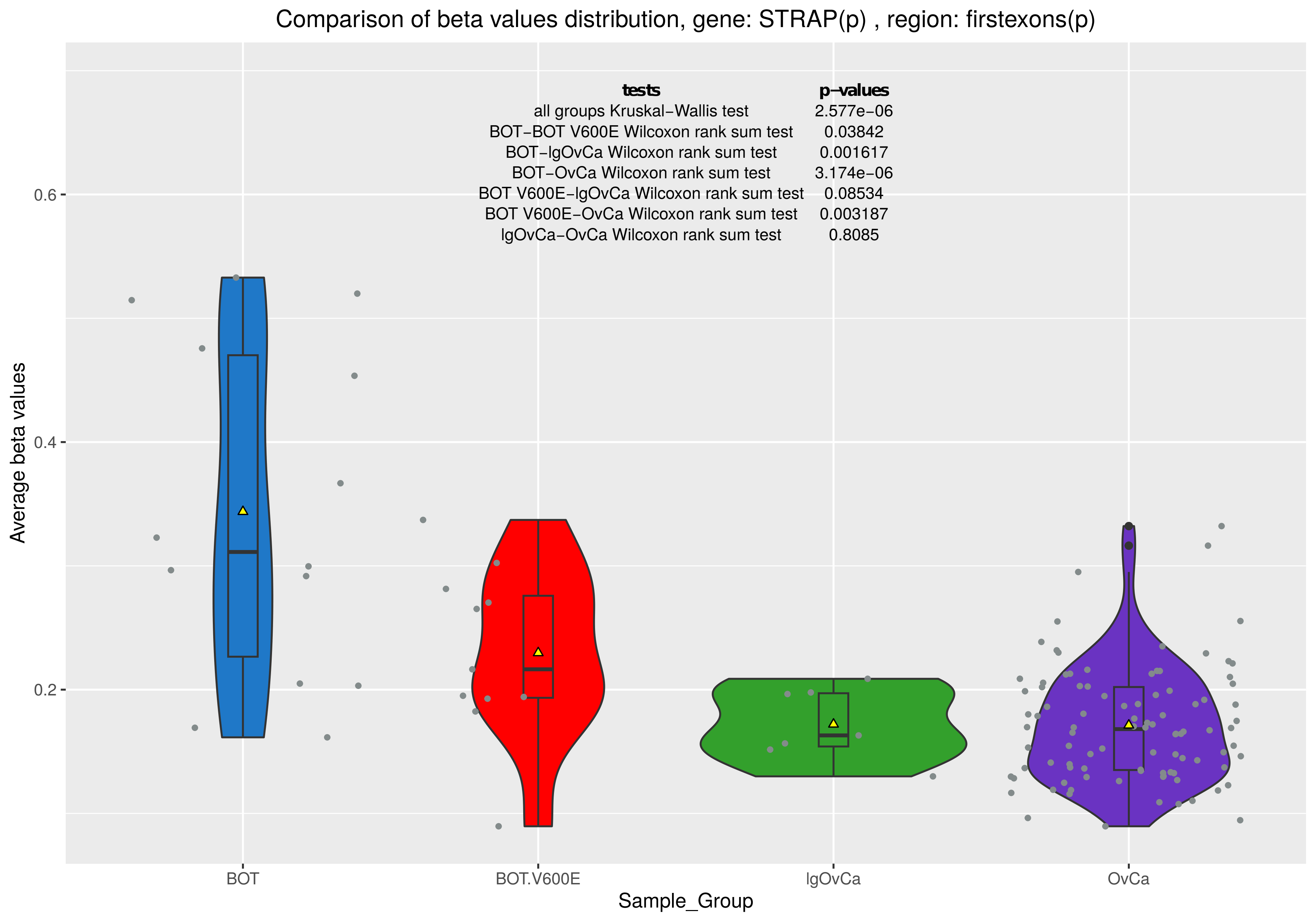

Comparison of beta values distribution, gene: STRAP(p) , region: firstexons(p)
tests
p−values
all groups Kruskal−Wallis test
2.577e−06
BOT−BOT V600E Wilcoxon rank sum test
0.03842
BOT−lgOvCa Wilcoxon rank sum test
0.001617
BOT−OvCa Wilcoxon rank sum test
3.174e−06
BOT V600E−lgOvCa Wilcoxon rank sum test
0.08534
BOT V600E−OvCa Wilcoxon rank sum test
0.003187
lgOvCa−OvCa Wilcoxon rank sum test
0.8085
Average beta values
0.6
0.4
0.2
BOT
BOT.V600E
lgOvCa
OvCa
Sample_Group

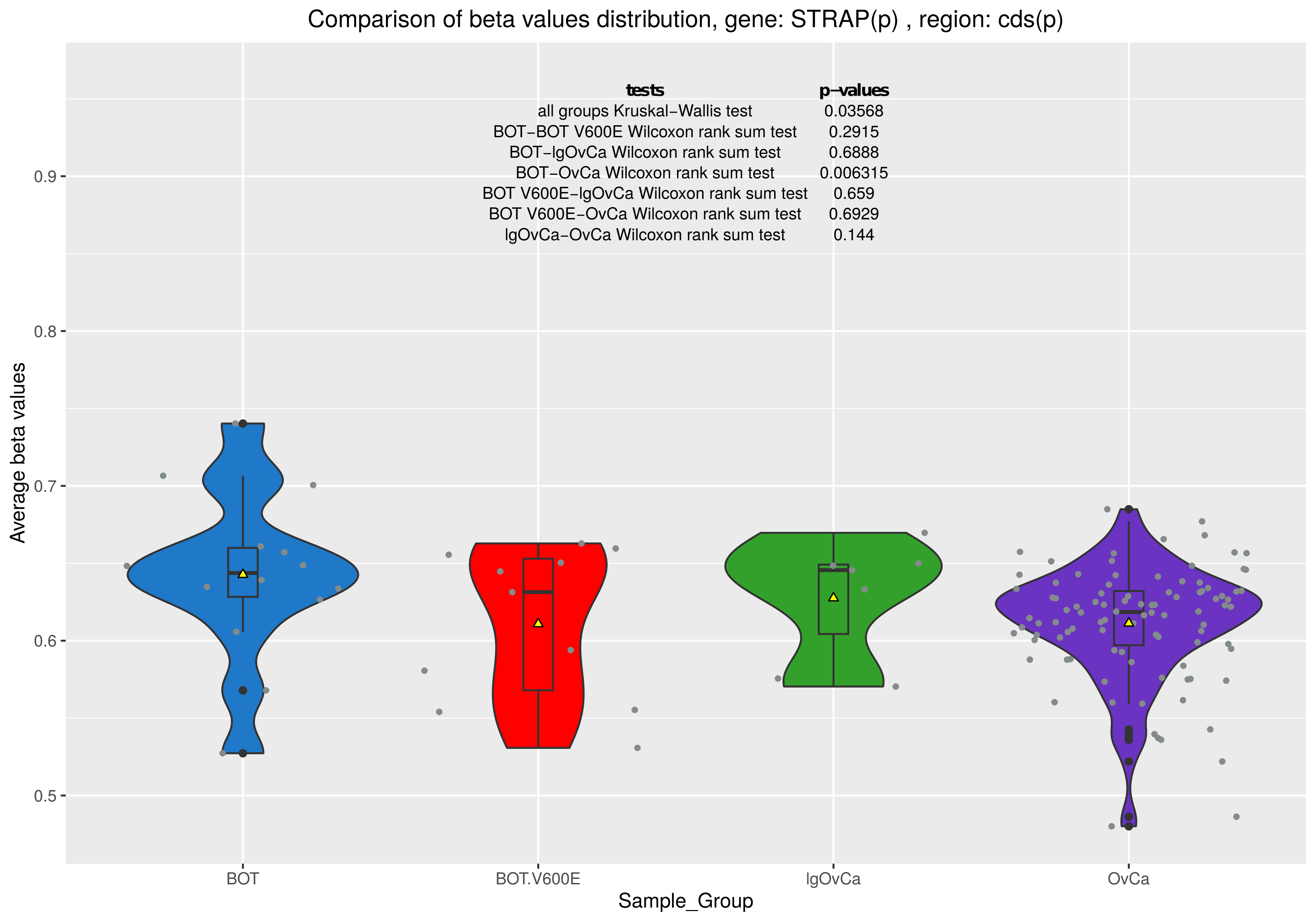

Comparison of beta values distribution, gene: STRAP(p) , region: cds(p)
tests
p−values
all groups Kruskal−Wallis test
0.03568
BOT−BOT V600E Wilcoxon rank sum test
0.2915
BOT−lgOvCa Wilcoxon rank sum test
0.6888
BOT−OvCa Wilcoxon rank sum test
0.006315
BOT V600E−lgOvCa Wilcoxon rank sum test
0.659
BOT V600E−OvCa Wilcoxon rank sum test
0.6929
lgOvCa−OvCa Wilcoxon rank sum test
0.144
Average beta values
0.9
0.8
0.7
0.6
0.5
BOT
BOT.V600E
lgOvCa
OvCa
Sample_Group

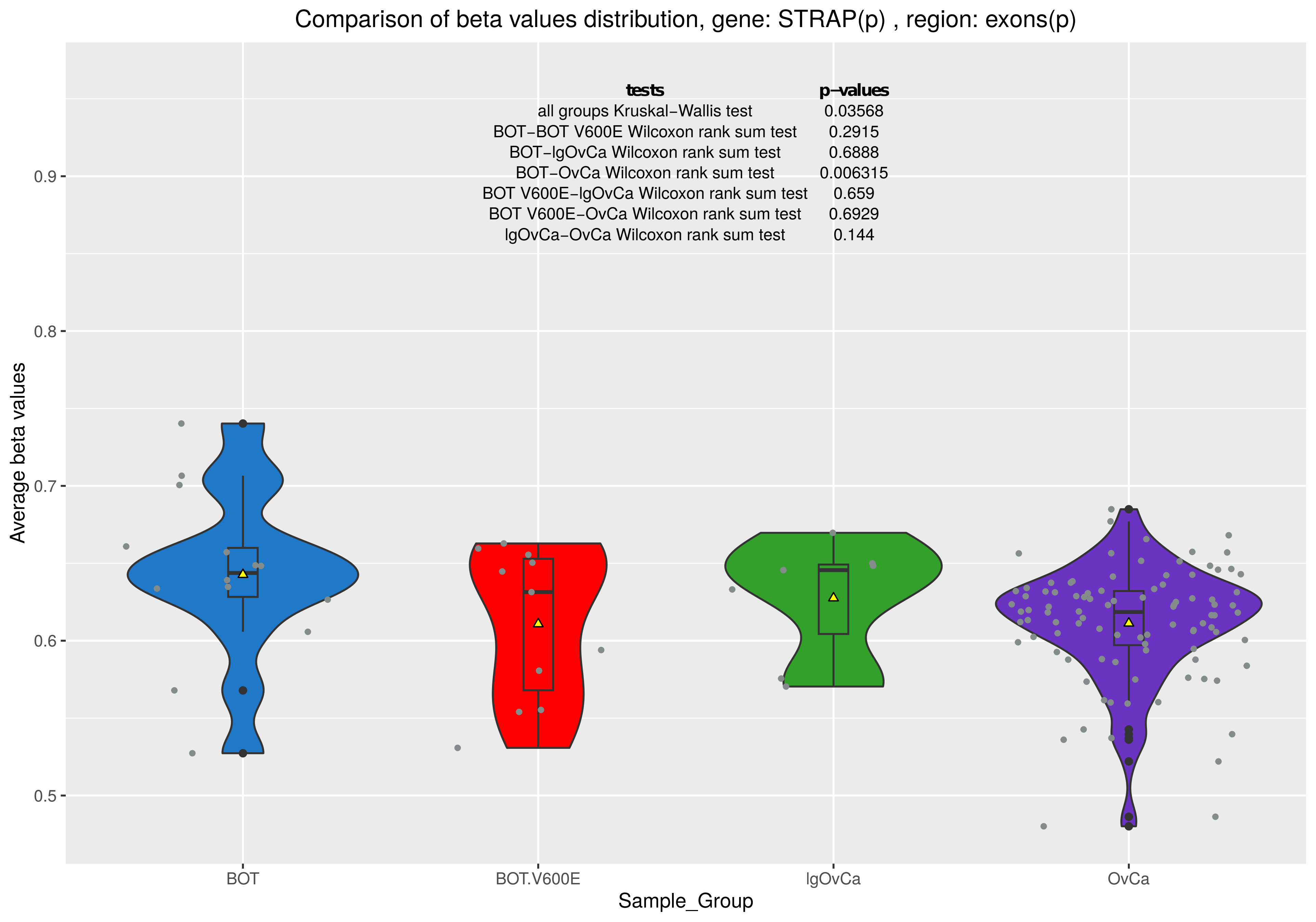
Comparison of beta values distribution, gene: STRAP(p) , region: exons(p)
tests
p−values
all groups Kruskal−Wallis test
0.03568
BOT−BOT V600E Wilcoxon rank sum test
0.2915
BOT−lgOvCa Wilcoxon rank sum test
0.6888
BOT−OvCa Wilcoxon rank sum test
0.006315
BOT V600E−lgOvCa Wilcoxon rank sum test
0.659
BOT V600E−OvCa Wilcoxon rank sum test
0.6929
lgOvCa−OvCa Wilcoxon rank sum test
0.144
Average beta values
0.5
0.6
0.7
0.8
0.9
BOT
BOT.V600E
lgOvCa
OvCa
Sample_Group

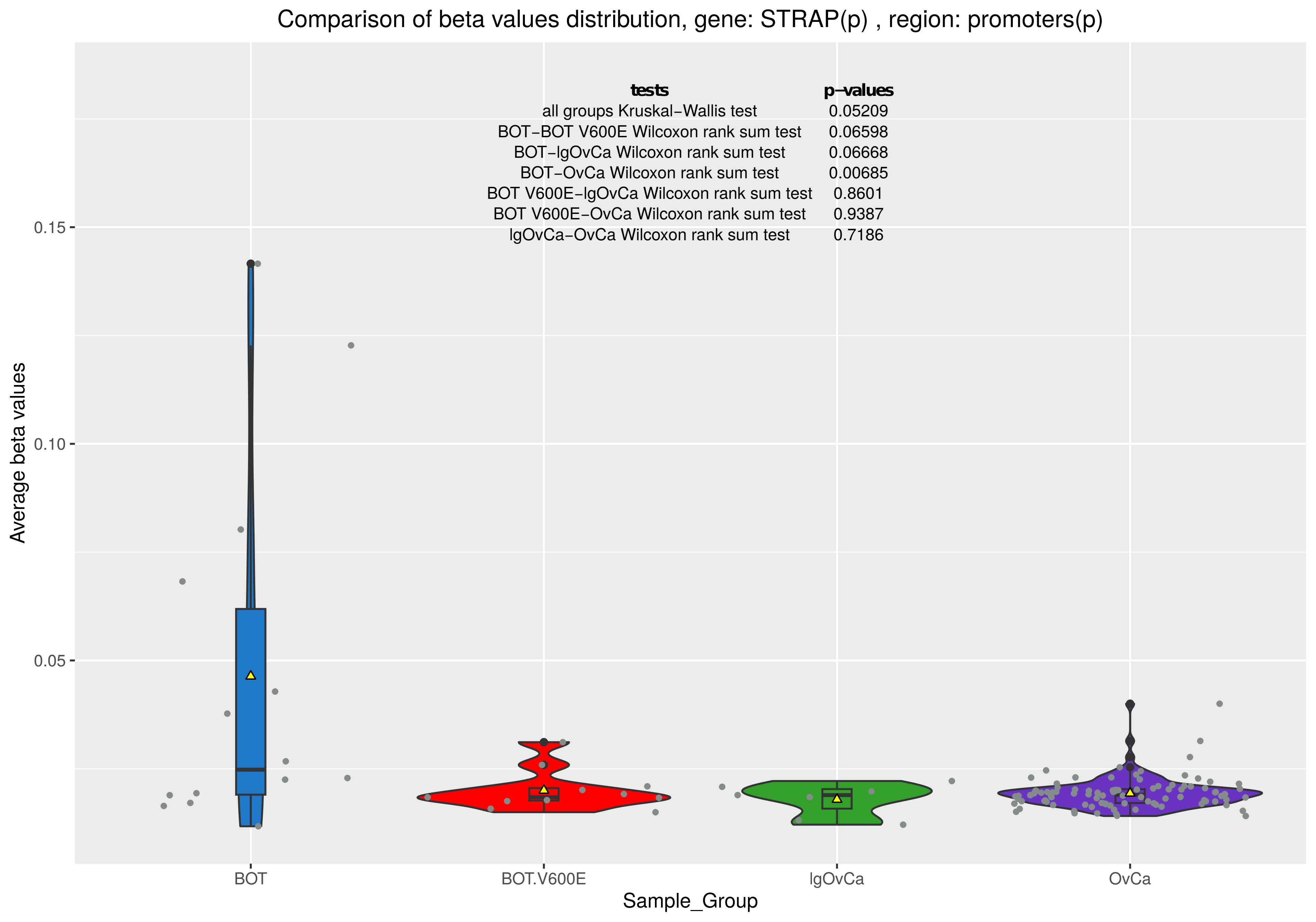
Comparison of beta values distribution, gene: STRAP(p) , region: promoters(p)
tests
p−values
all groups Kruskal−Wallis test
0.05209
BOT−BOT V600E Wilcoxon rank sum test
0.06598
BOT−lgOvCa Wilcoxon rank sum test
0.06668
BOT−OvCa Wilcoxon rank sum test
0.00685
BOT V600E−lgOvCa Wilcoxon rank sum test
0.8601
BOT V600E−OvCa Wilcoxon rank sum test
0.9387
lgOvCa−OvCa Wilcoxon rank sum test
0.7186
Average beta values
0.15
0.10
0.05
BOT
BOT.V600E
lgOvCa
OvCa
Sample_Group

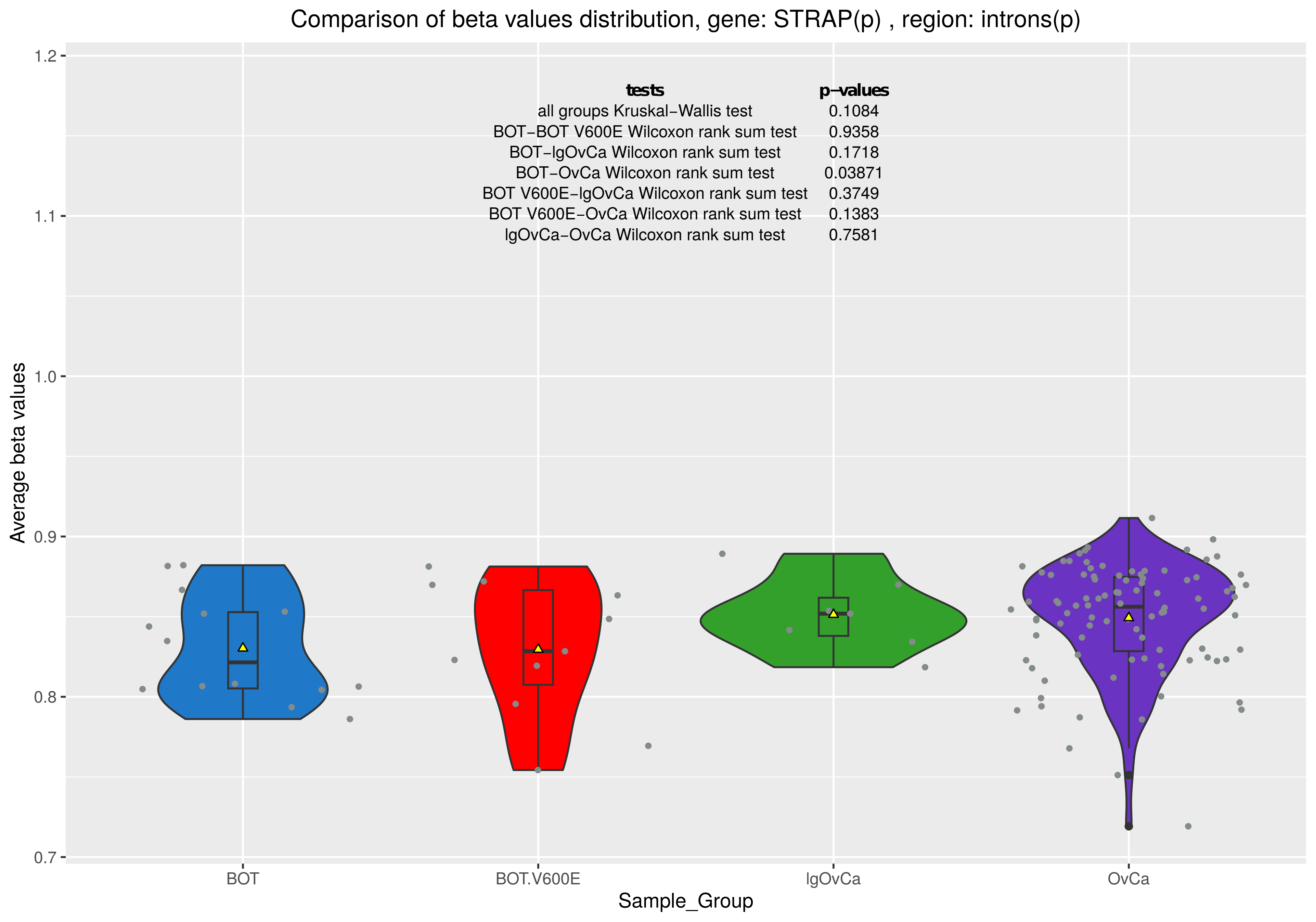

Comparison of beta values distribution, gene: STRAP(p) , region: introns(p)
tests
p−values
all groups Kruskal−Wallis test
0.1084
BOT−BOT V600E Wilcoxon rank sum test
0.9358
BOT−lgOvCa Wilcoxon rank sum test
0.1718
BOT−OvCa Wilcoxon rank sum test
0.03871
BOT V600E−lgOvCa Wilcoxon rank sum test
0.3749
BOT V600E−OvCa Wilcoxon rank sum test
0.1383
lgOvCa−OvCa Wilcoxon rank sum test
0.7581
Average beta values
1.2
1.1
1.0
0.9
0.8
0.7
BOT
BOT.V600E
lgOvCa
OvCa
Sample_Group

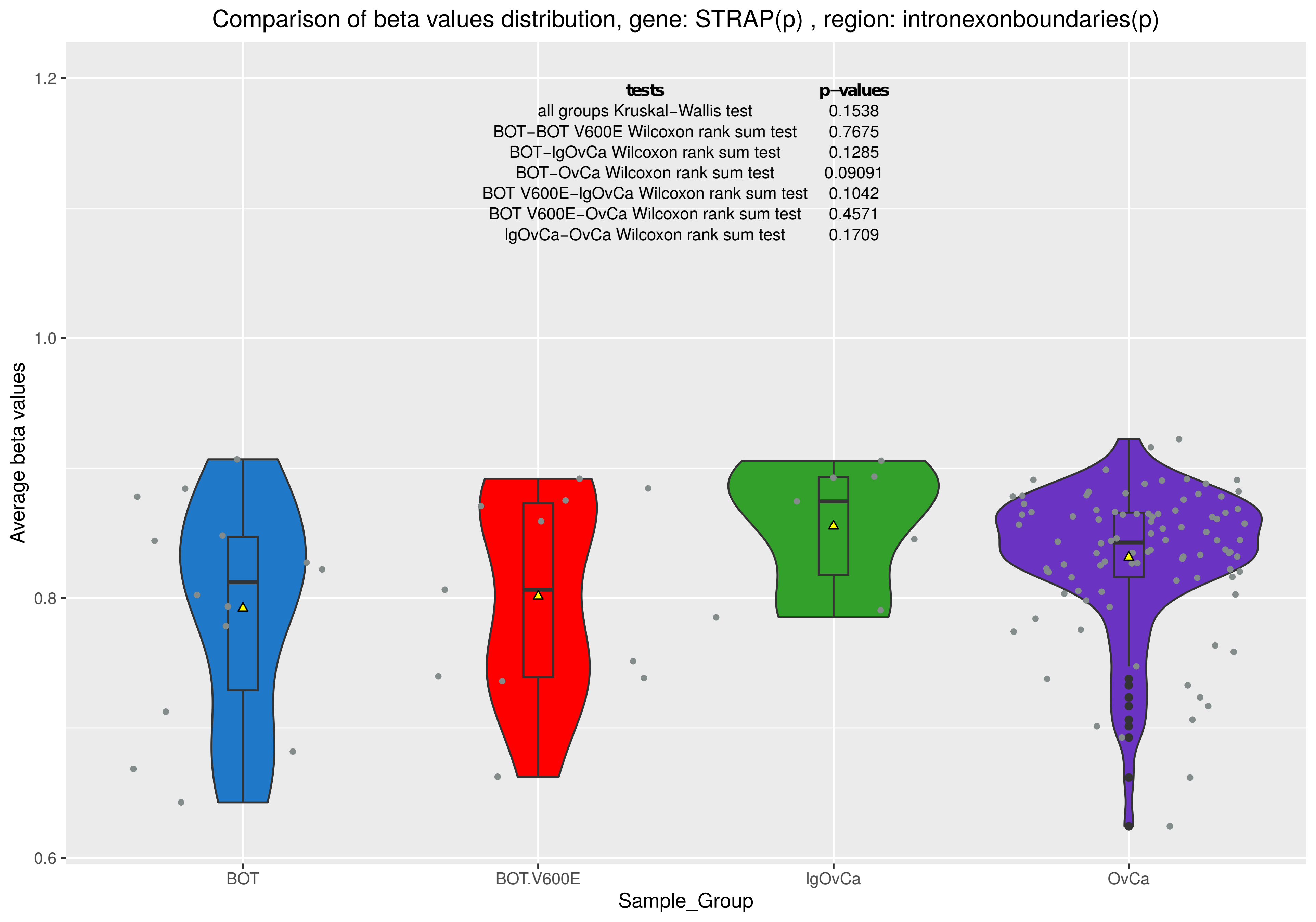

Comparison of beta values distribution, gene: STRAP(p) , region: intronexonboundaries(p)
tests
p−values
all groups Kruskal−Wallis test 0.1538
BOT−BOT V600E Wilcoxon rank sum test 0.7675
BOT−lgOvCa Wilcoxon rank sum test 0.1285
BOT−OvCa Wilcoxon rank sum test 0.09091
BOT V600E−lgOvCa Wilcoxon rank sum test 0.1042
BOT V600E−OvCa Wilcoxon rank sum test 0.4571
lgOvCa−OvCa Wilcoxon rank sum test 0.1709
Average beta values
1.2
1.0
0.8
0.6
BOT
BOT.V600E
lgOvCa
OvCa
Sample_Group

Comparison of beta values distribution, gene: CDKN2B(m) , region: promoters(m)

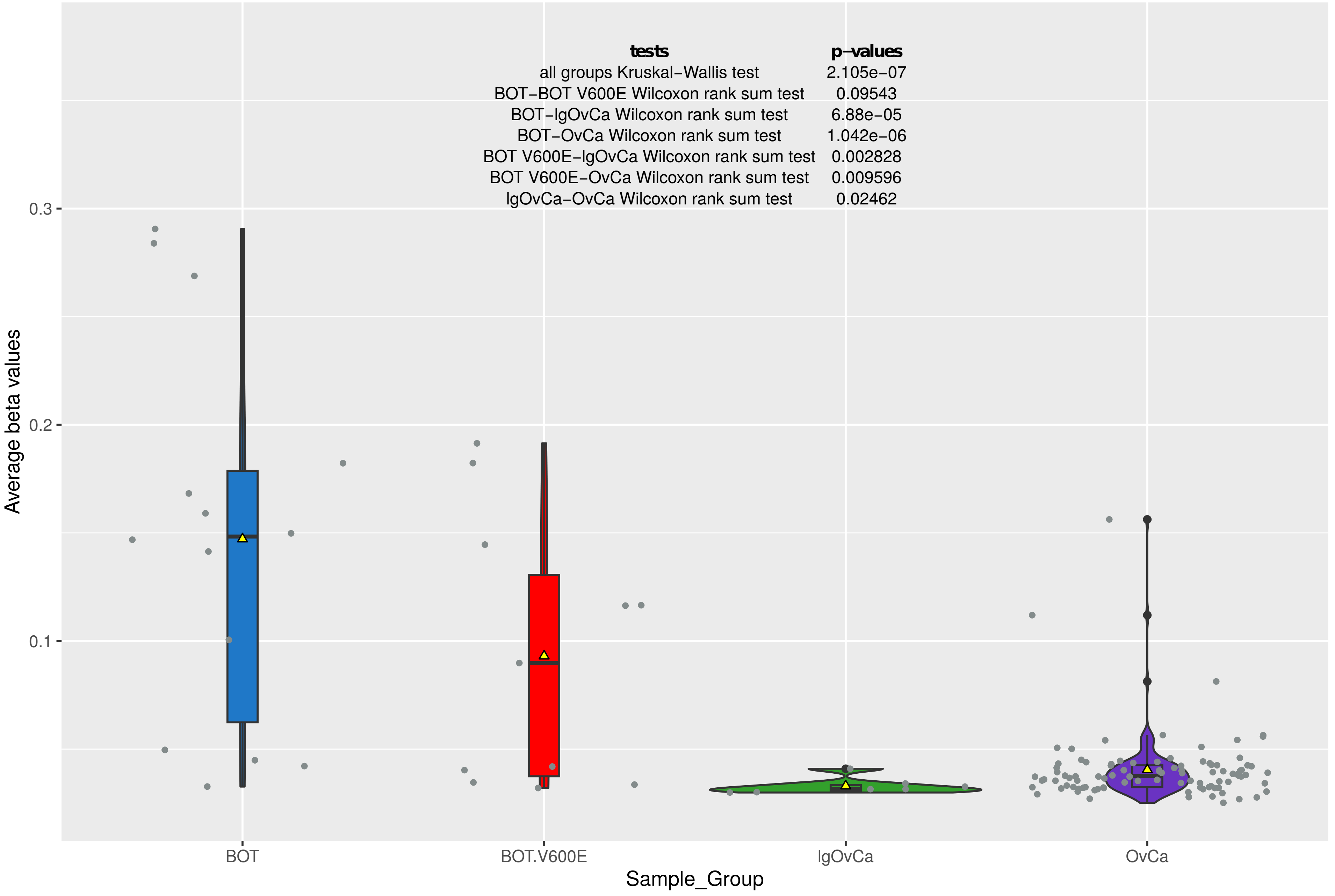

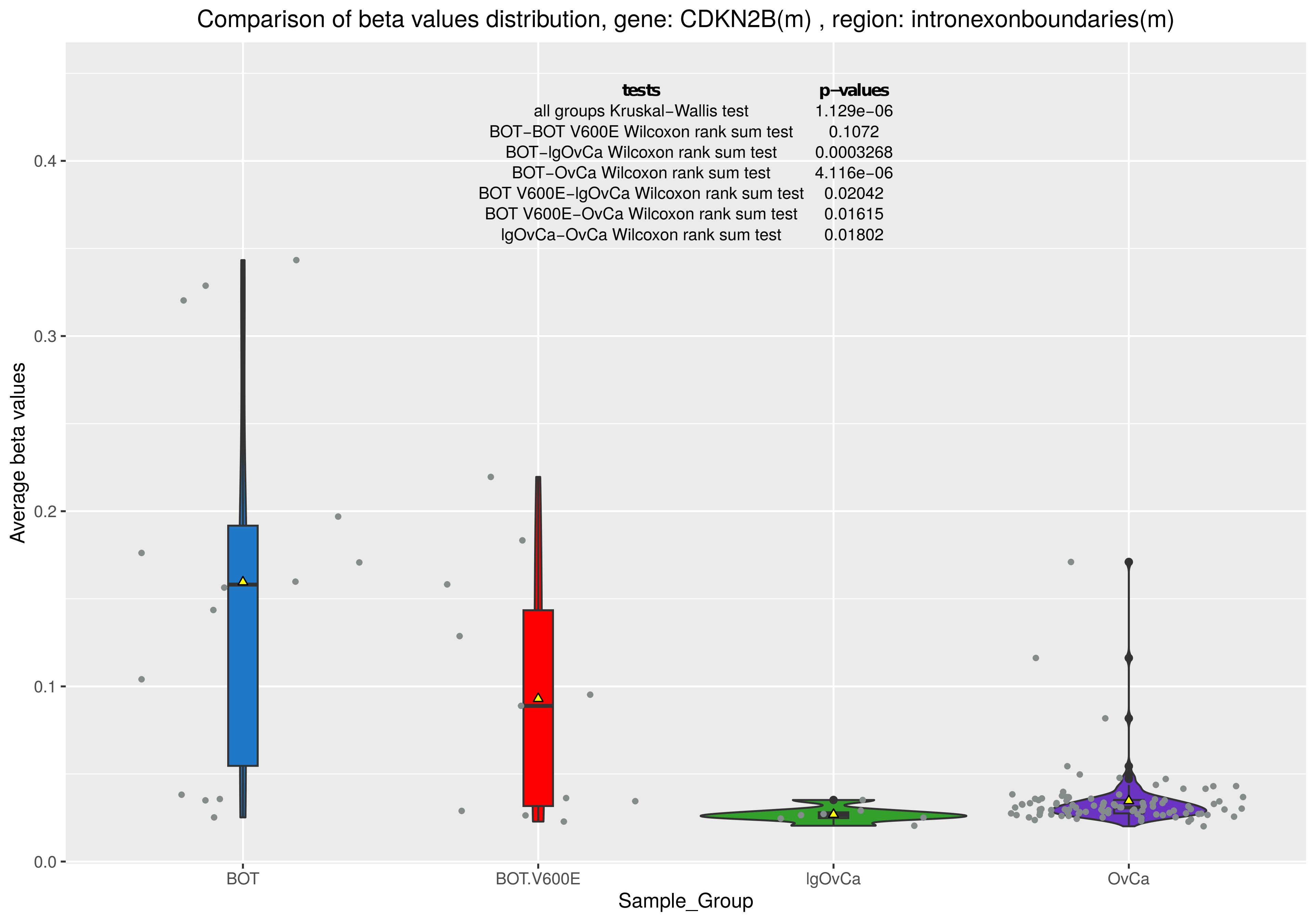
Comparison of beta values distribution, gene: CDKN2B(m) , region: intronexonboundaries(m)
tests
p−values
all groups Kruskal−Wallis test
1.129e−06
BOT−BOT V600E Wilcoxon rank sum test
0.1072
BOT−lgOvCa Wilcoxon rank sum test
0.0003268
BOT−OvCa Wilcoxon rank sum test
4.116e−06
BOT V600E−lgOvCa Wilcoxon rank sum test
0.02042
BOT V600E−OvCa Wilcoxon rank sum test
0.01615
lgOvCa−OvCa Wilcoxon rank sum test
0.01802
Average beta values
0.0
0.1
0.2
0.3
0.4
BOT
BOT.V600E
lgOvCa
OvCa
Sample_Group

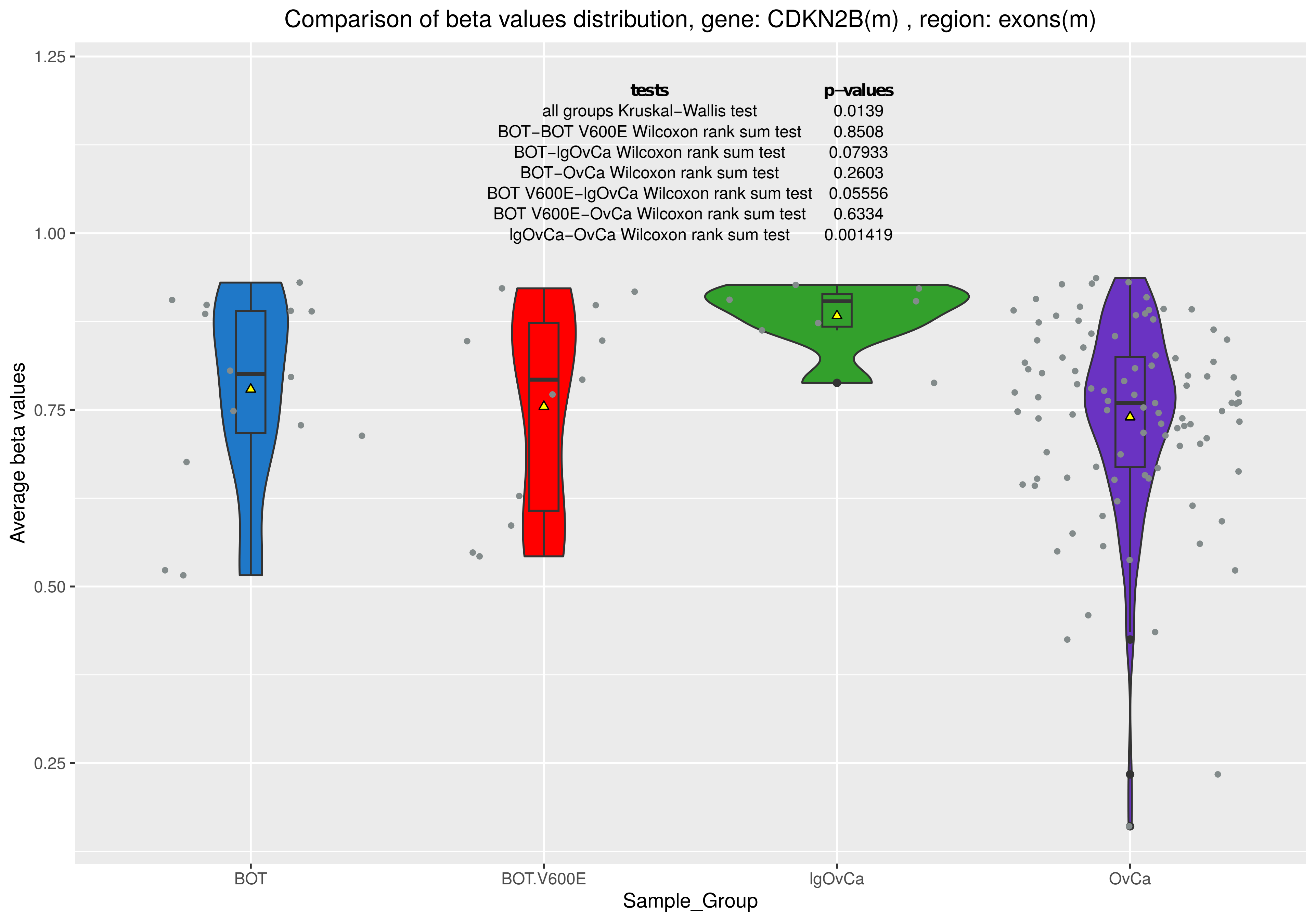
Comparison of beta values distribution, gene: CDKN2B(m) , region: exons(m)
tests
p−values
all groups Kruskal−Wallis test
0.0139
BOT−BOT V600E Wilcoxon rank sum test
0.8508
BOT−lgOvCa Wilcoxon rank sum test
0.07933
BOT−OvCa Wilcoxon rank sum test
0.2603
BOT V600E−lgOvCa Wilcoxon rank sum test
0.05556
BOT V600E−OvCa Wilcoxon rank sum test
0.6334
lgOvCa−OvCa Wilcoxon rank sum test
0.001419
Average beta values
1.25
1.00
0.75
0.50
0.25
BOT
BOT.V600E
lgOvCa
OvCa
Sample_Group

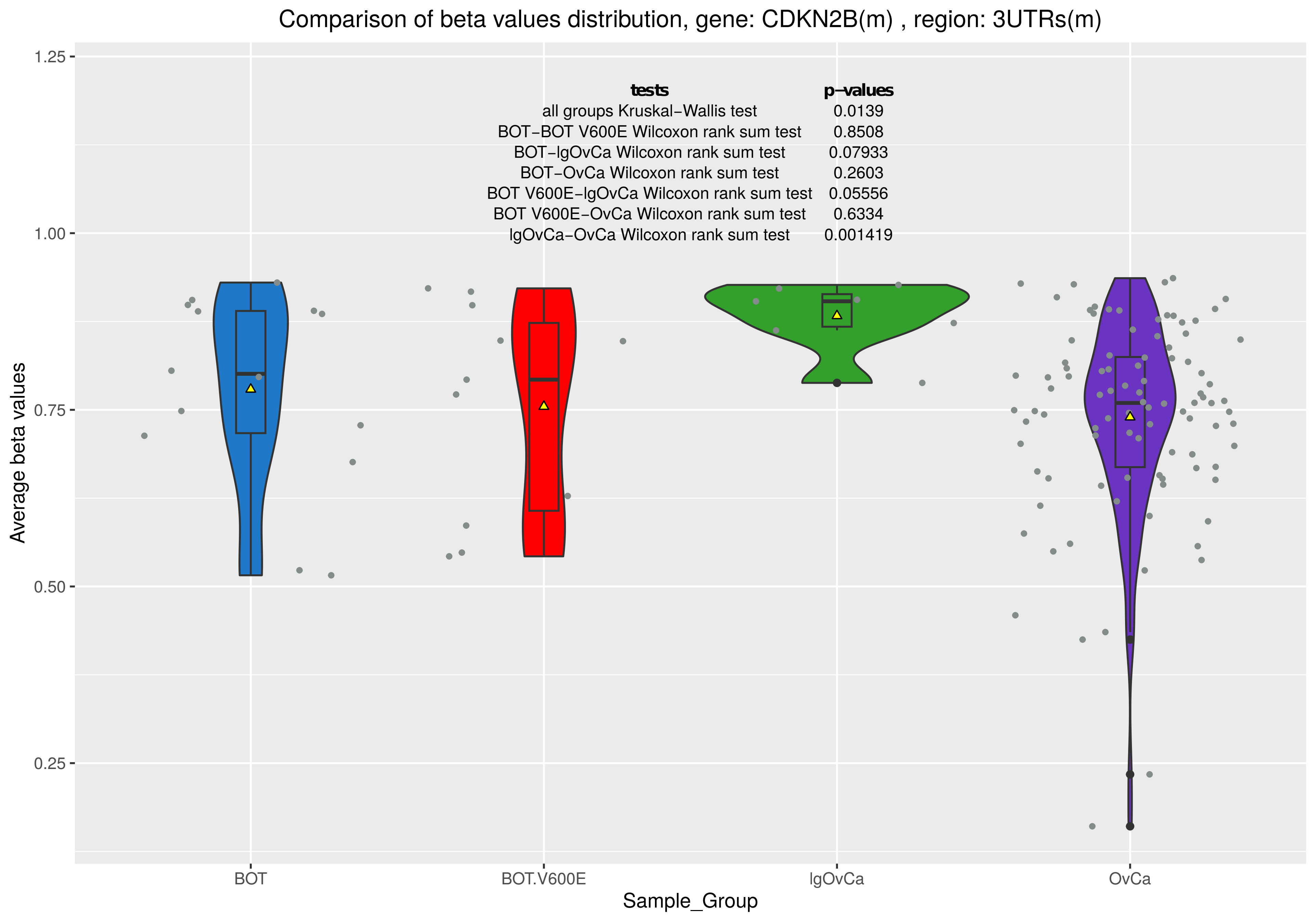

Comparison of beta values distribution, gene: CDKN2B(m) , region: 3UTRs(m)
tests
p−values
all groups Kruskal−Wallis test
0.0139
BOT−BOT V600E Wilcoxon rank sum test
0.8508
BOT−lgOvCa Wilcoxon rank sum test
0.07933
BOT−OvCa Wilcoxon rank sum test
0.2603
BOT V600E−lgOvCa Wilcoxon rank sum test
0.05556
BOT V600E−OvCa Wilcoxon rank sum test
0.6334
lgOvCa−OvCa Wilcoxon rank sum test
0.001419
1.25
1.00
0.75
0.50
0.25
Average beta values
BOT
BOT.V600E
lgOvCa
OvCa
Sample_Group

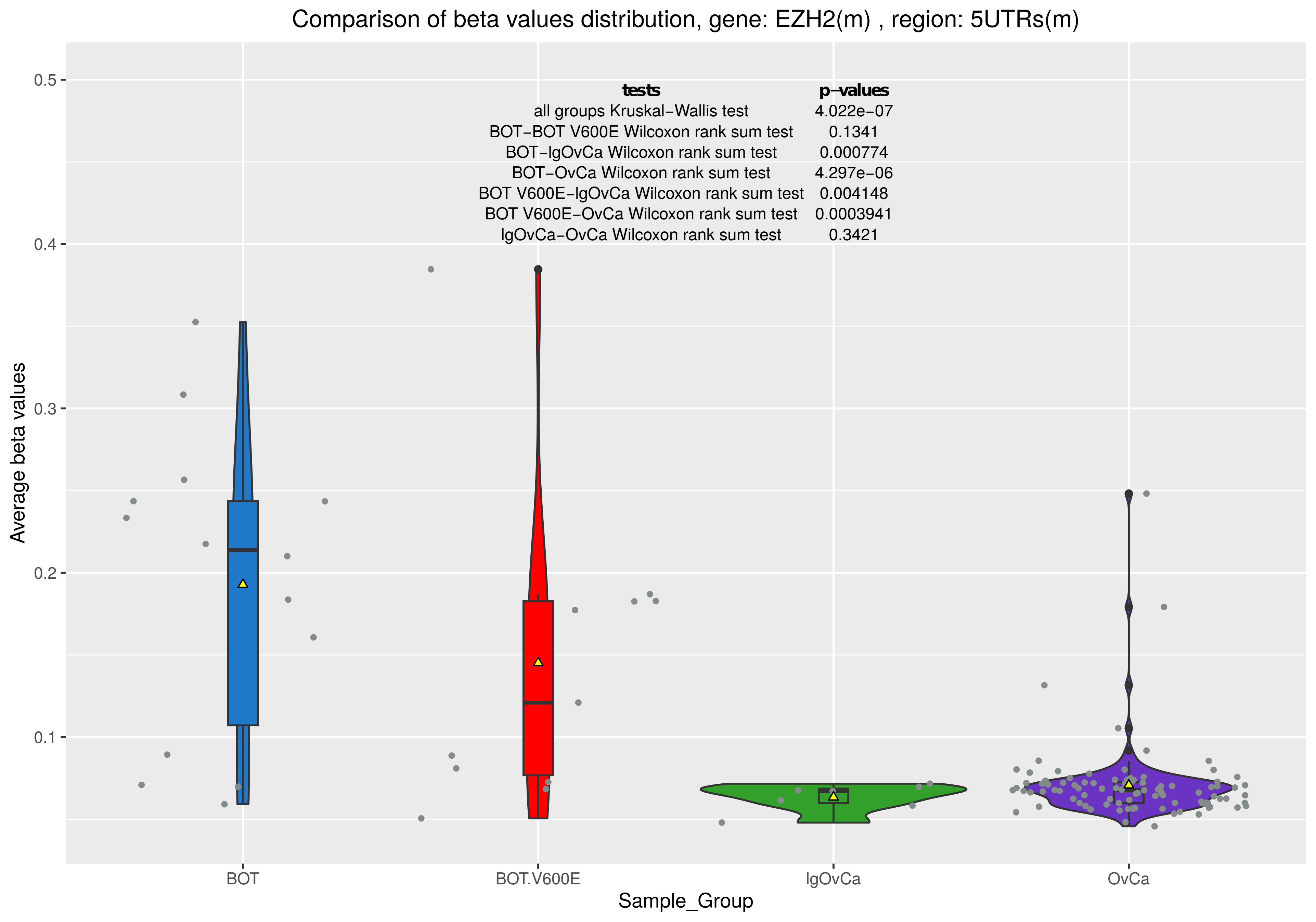
Comparison of beta values distribution, gene: EZH2(m) , region: 5UTRs(m)
tests
p−values
all groups Kruskal−Wallis test
4.022e−07
BOT−BOT V600E Wilcoxon rank sum test
0.1341
BOT−lgOvCa Wilcoxon rank sum test
0.000774
BOT−OvCa Wilcoxon rank sum test
4.297e−06
BOT V600E−lgOvCa Wilcoxon rank sum test
0.004148
BOT V600E−OvCa Wilcoxon rank sum test
0.0003941
lgOvCa−OvCa Wilcoxon rank sum test
0.3421
Average beta values
0.5
0.4
0.3
0.2
0.1
BOT
BOT.V600E
lgOvCa
OvCa
Sample_Group

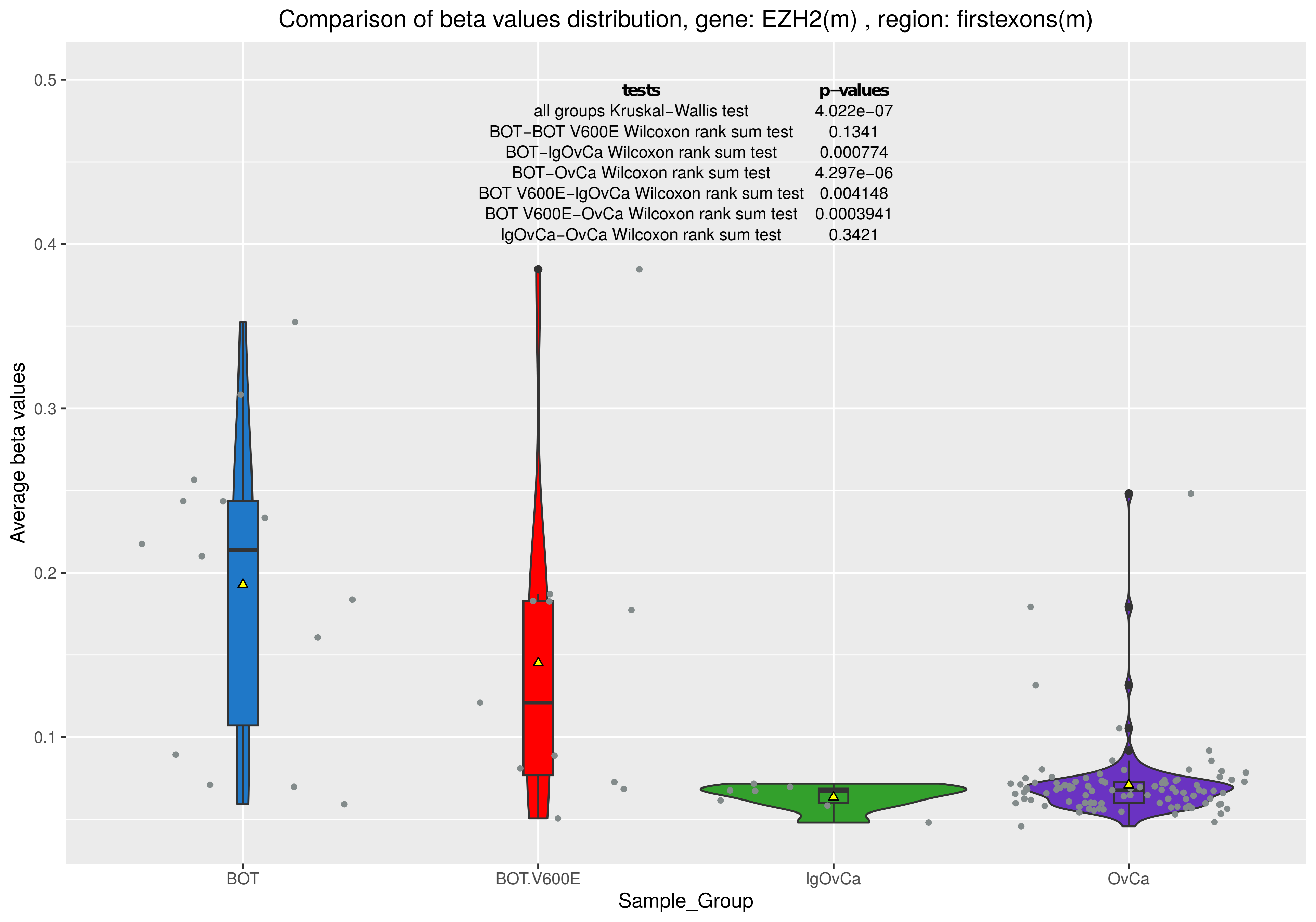
Comparison of beta values distribution, gene: EZH2(m) , region: firstexons(m)
tests
p−values
all groups Kruskal−Wallis test
4.022e−07
BOT−BOT V600E Wilcoxon rank sum test
0.1341
BOT−lgOvCa Wilcoxon rank sum test
0.000774
BOT−OvCa Wilcoxon rank sum test
4.297e−06
BOT V600E−lgOvCa Wilcoxon rank sum test
0.004148
BOT V600E−OvCa Wilcoxon rank sum test
0.0003941
lgOvCa−OvCa Wilcoxon rank sum test
0.3421
Average beta values
0.5
0.4
0.3
0.2
0.1
BOT
BOT.V600E
lgOvCa
OvCa
Sample_Group

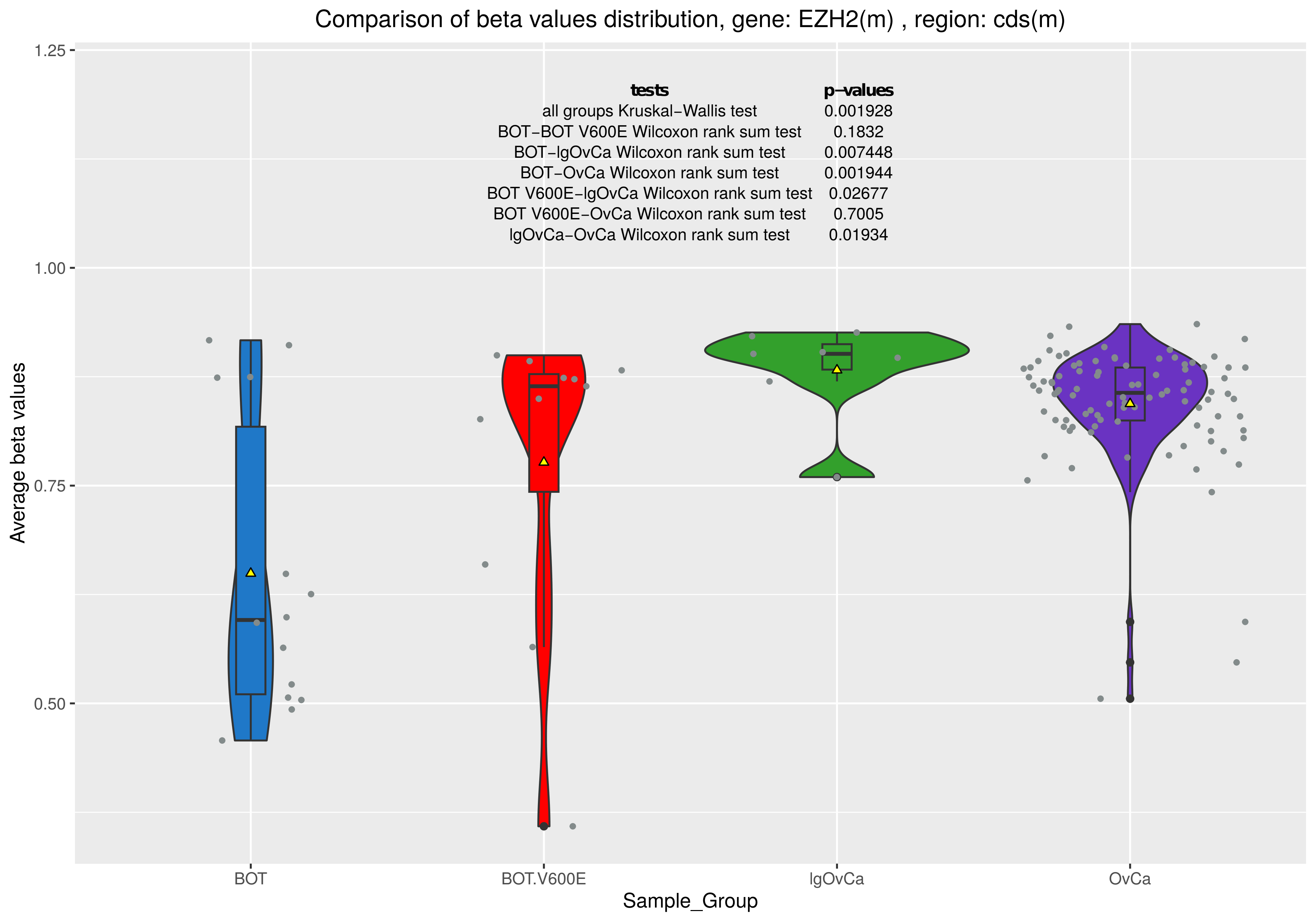

Comparison of beta values distribution, gene: EZH2(m) , region: cds(m)
tests
p−values
all groups Kruskal−Wallis test
0.001928
BOT−BOT V600E Wilcoxon rank sum test
0.1832
BOT−lgOvCa Wilcoxon rank sum test
0.007448
BOT−OvCa Wilcoxon rank sum test
0.001944
BOT V600E−lgOvCa Wilcoxon rank sum test
0.02677
BOT V600E−OvCa Wilcoxon rank sum test
0.7005
lgOvCa−OvCa Wilcoxon rank sum test
0.01934
Average beta values
1.25
1.00
0.75
0.50
BOT
BOT.V600E
lgOvCa
OvCa
Sample_Group

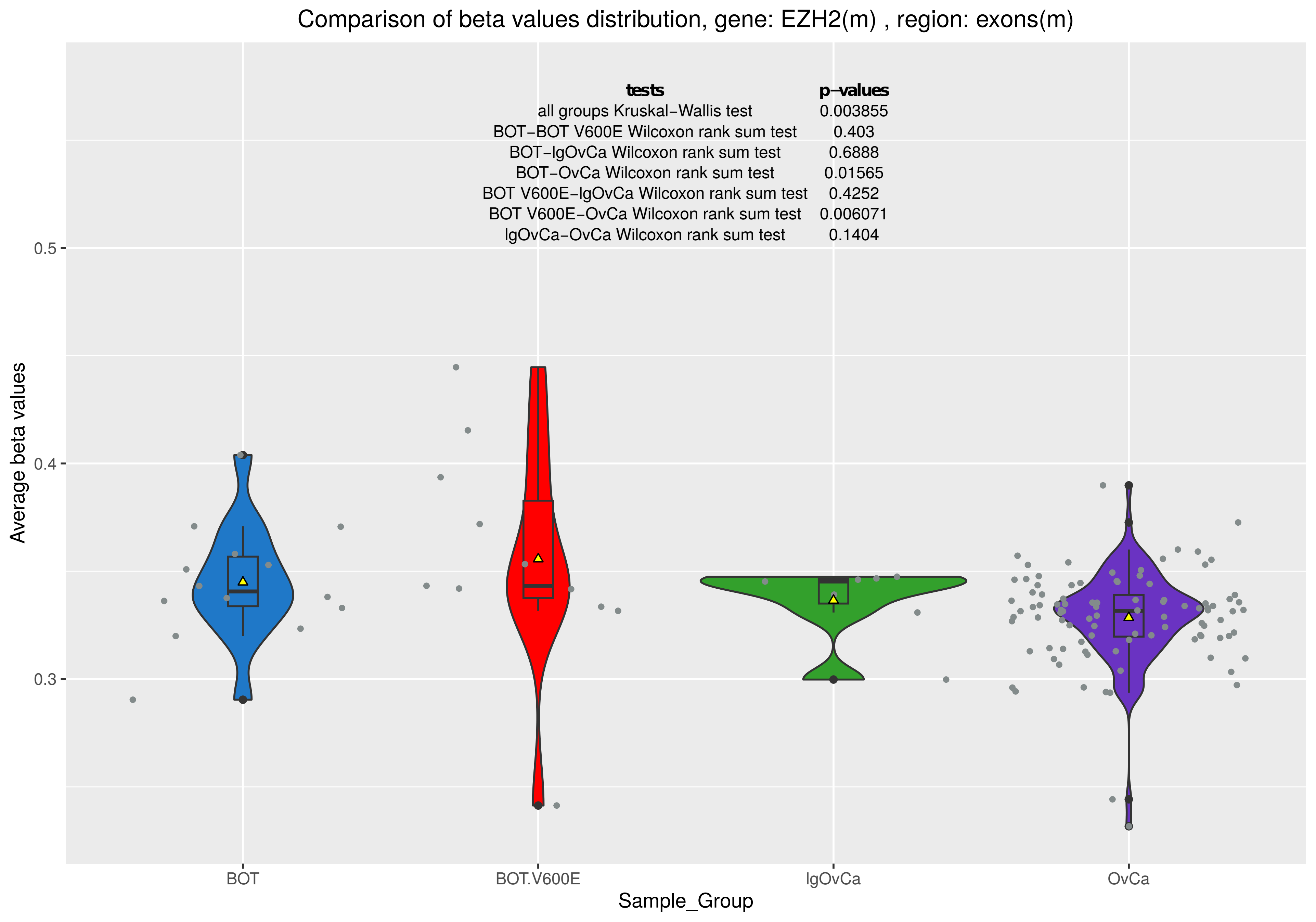

Comparison of beta values distribution, gene: EZH2(m) , region: exons(m)
tests
p−values
all groups Kruskal−Wallis test
0.003855
BOT−BOT V600E Wilcoxon rank sum test
0.403
BOT−lgOvCa Wilcoxon rank sum test
0.6888
BOT−OvCa Wilcoxon rank sum test
0.01565
BOT V600E−lgOvCa Wilcoxon rank sum test
0.4252
BOT V600E−OvCa Wilcoxon rank sum test
0.006071
lgOvCa−OvCa Wilcoxon rank sum test
0.1404
Average beta values
0.5
0.4
0.3
BOT
BOT.V600E
lgOvCa
OvCa
Sample_Group

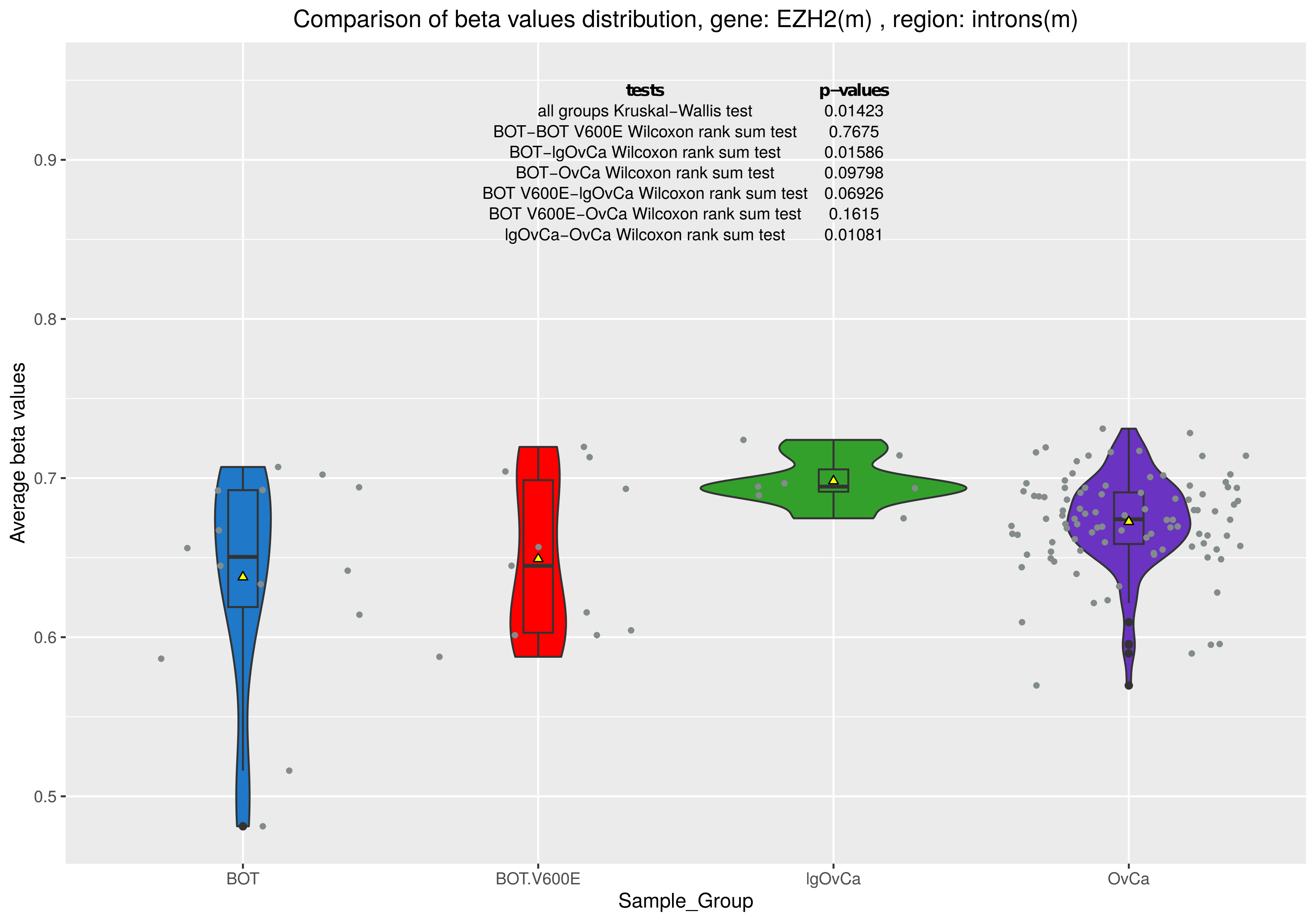
Comparison of beta values distribution, gene: EZH2(m) , region: introns(m)
tests
p−values
all groups Kruskal−Wallis test
0.01423
BOT−BOT V600E Wilcoxon rank sum test
0.7675
BOT−lgOvCa Wilcoxon rank sum test
0.01586
BOT−OvCa Wilcoxon rank sum test
0.09798
BOT V600E−lgOvCa Wilcoxon rank sum test
0.06926
BOT V600E−OvCa Wilcoxon rank sum test
0.1615
lgOvCa−OvCa Wilcoxon rank sum test
0.01081
Average beta values
0.9
0.8
0.7
0.6
0.5
BOT
BOT.V600E
lgOvCa
OvCa
Sample_Group

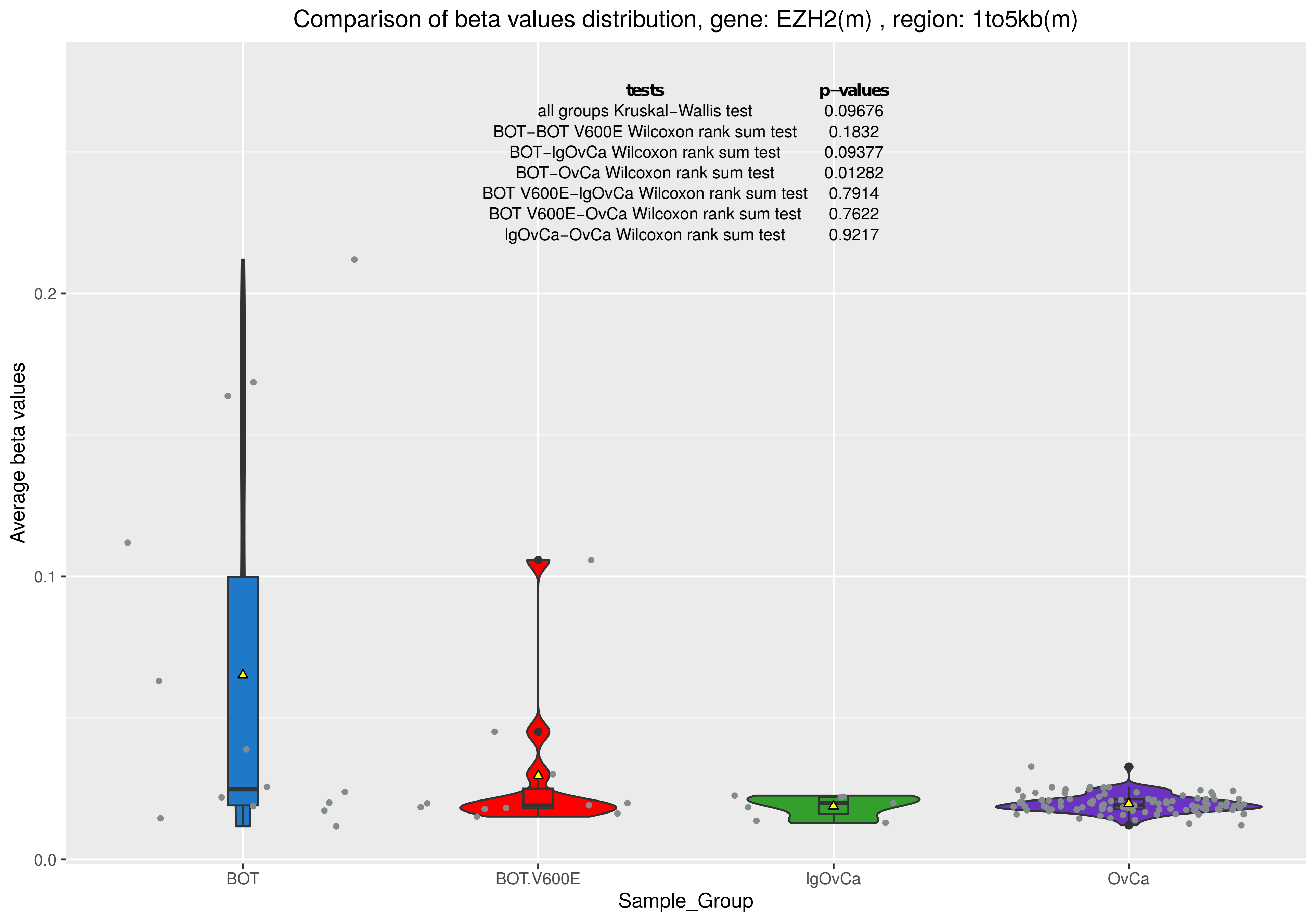
Comparison of beta values distribution, gene: EZH2(m) , region: 1to5kb(m)
tests
p−values
all groups Kruskal−Wallis test
0.09676
BOT−BOT V600E Wilcoxon rank sum test
0.1832
BOT−lgOvCa Wilcoxon rank sum test
0.09377
BOT−OvCa Wilcoxon rank sum test
0.01282
BOT V600E−lgOvCa Wilcoxon rank sum test
0.7914
BOT V600E−OvCa Wilcoxon rank sum test
0.7622
lgOvCa−OvCa Wilcoxon rank sum test
0.9217
Average beta values
0.2
0.1
0.0
BOT
BOT.V600E
lgOvCa
OvCa
Sample_Group

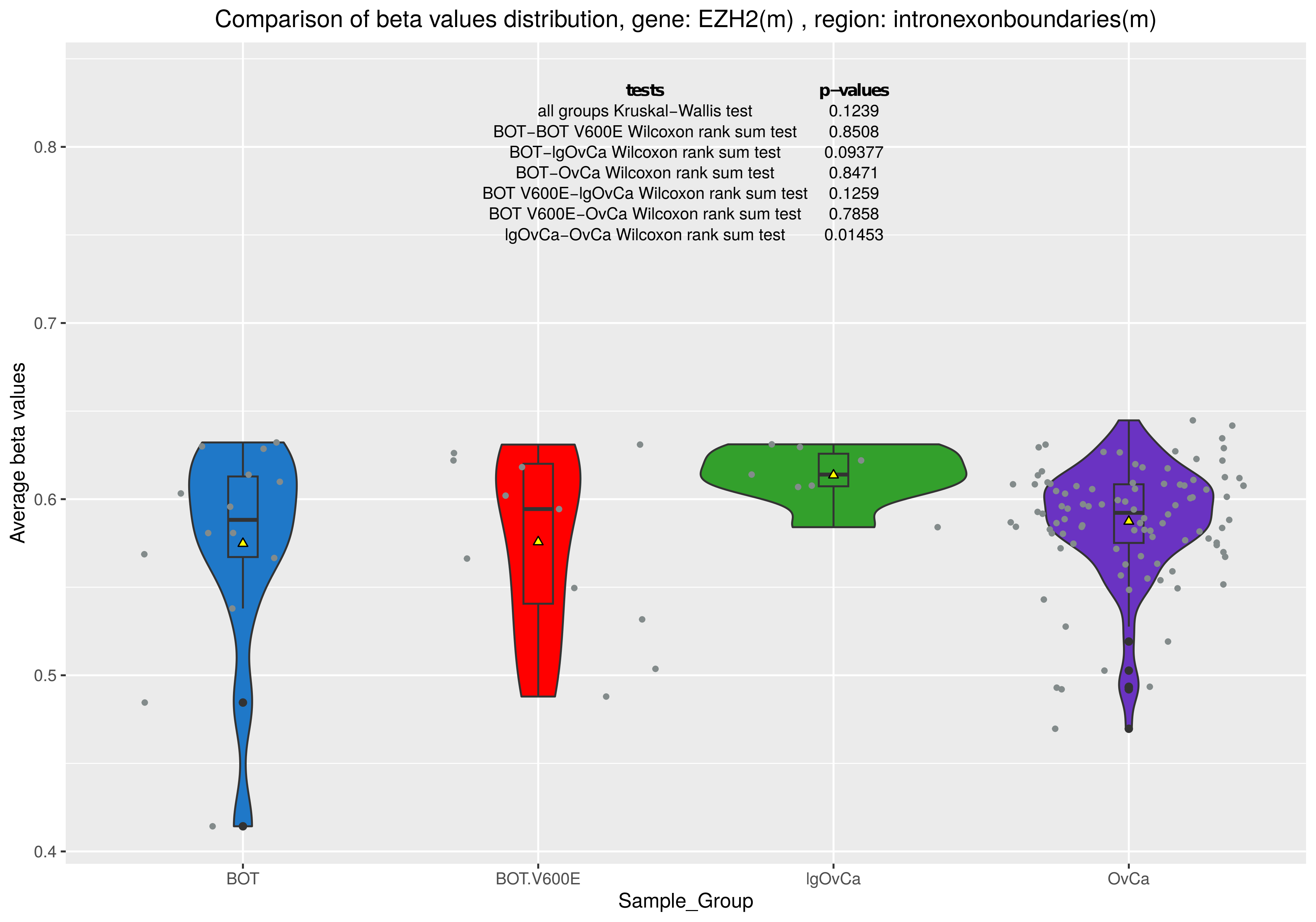

Comparison of beta values distribution, gene: EZH2(m) , region: intronexonboundaries(m)
tests
p−values
all groups Kruskal−Wallis test
0.1239
BOT−BOT V600E Wilcoxon rank sum test
0.8508
BOT−IgOvCa Wilcoxon rank sum test
0.09377
BOT−OvCa Wilcoxon rank sum test
0.8471
BOT V600E−IgOvCa Wilcoxon rank sum test
0.1259
BOT V600E−OvCa Wilcoxon rank sum test
0.7858
IgOvCa−OvCa Wilcoxon rank sum test
0.01453
Average beta values
0.8
0.7
0.6
0.5
0.4
BOT
BOT.V600E
IgOvCa
OvCa
Sample_Group

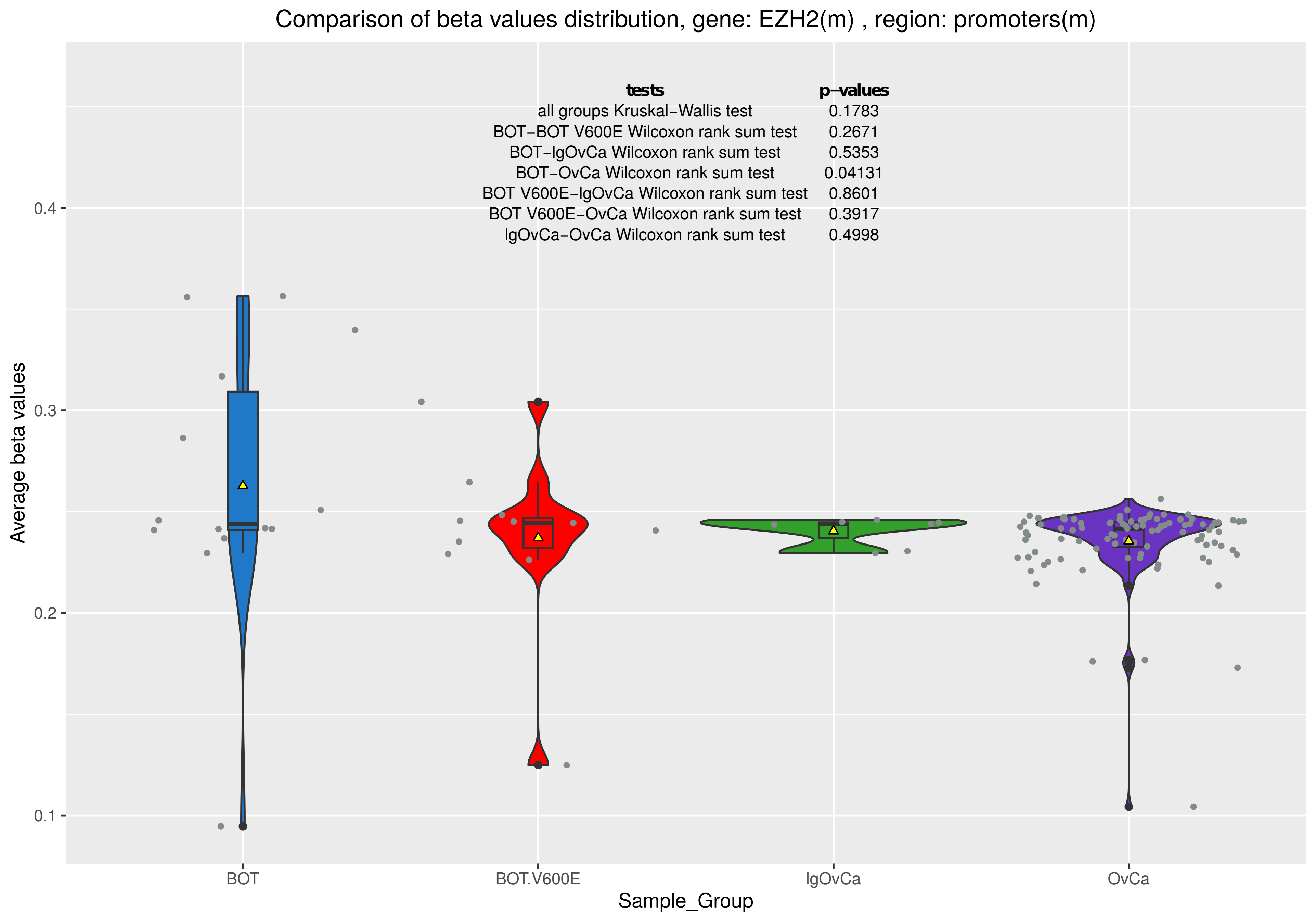

Comparison of beta values distribution, gene: EZH2(m) , region: promoters(m)
tests
p−values
all groups Kruskal−Wallis test
0.1783
BOT−BOT V600E Wilcoxon rank sum test
0.2671
BOT−lgOvCa Wilcoxon rank sum test
0.5353
BOT−OvCa Wilcoxon rank sum test
0.04131
BOT V600E−lgOvCa Wilcoxon rank sum test
0.8601
BOT V600E−OvCa Wilcoxon rank sum test
0.3917
lgOvCa−OvCa Wilcoxon rank sum test
0.4998
Average beta values
0.4
0.3
0.2
0.1
BOT
BOT.V600E
lgOvCa
OvCa
Sample_Group

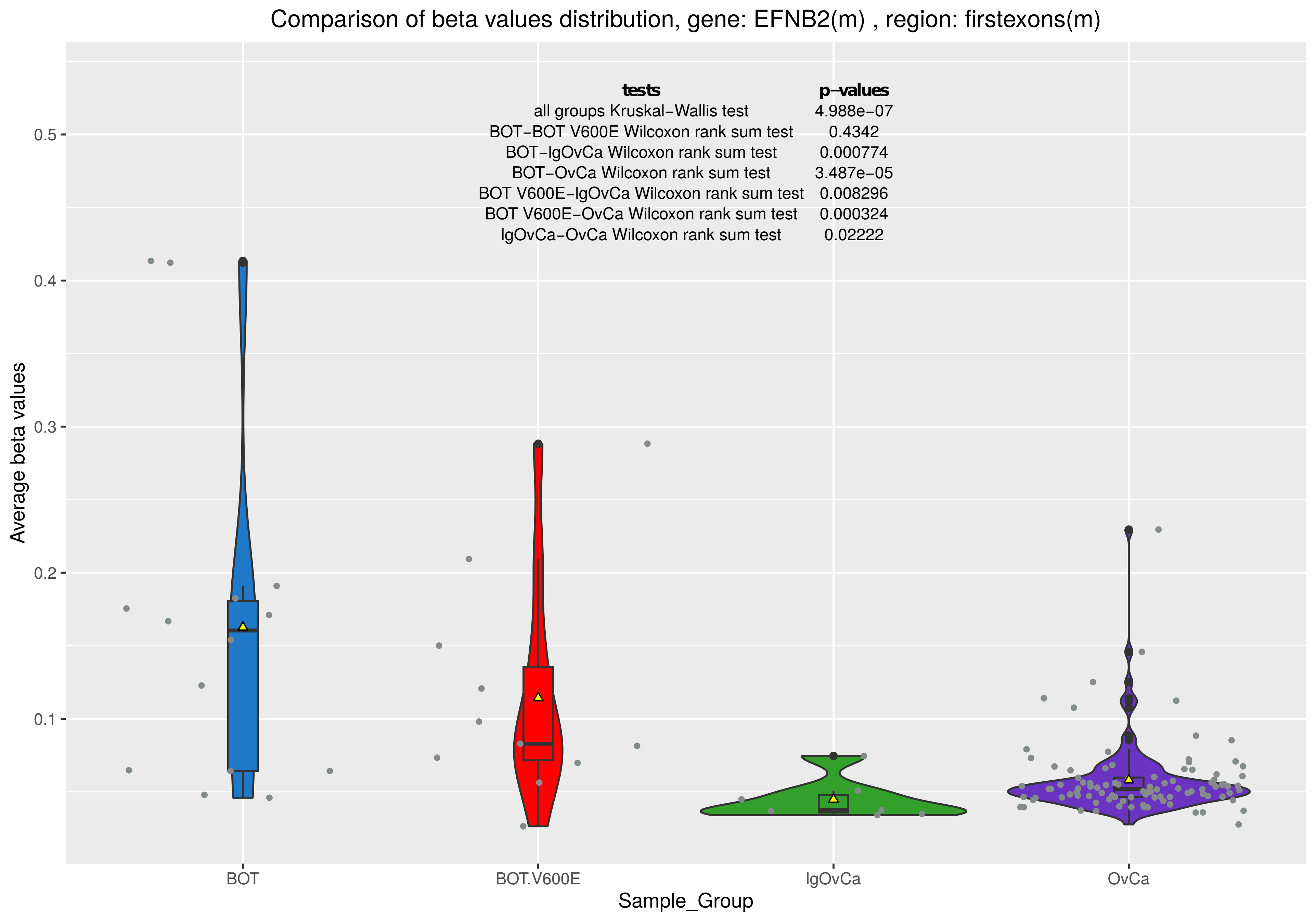
Comparison of beta values distribution, gene: EFNB2(m) , region: firstexons(m)
tests
p−values
all groups Kruskal−Wallis test
4.988e−07
BOT−BOT V600E Wilcoxon rank sum test
0.4342
BOT−lgOvCa Wilcoxon rank sum test
0.000774
BOT−OvCa Wilcoxon rank sum test
3.487e−05
BOT V600E−lgOvCa Wilcoxon rank sum test
0.008296
BOT V600E−OvCa Wilcoxon rank sum test
0.000324
lgOvCa−OvCa Wilcoxon rank sum test
0.02222
Average beta values
0.5
0.4
0.3
0.2
0.1
BOT
BOT.V600E
lgOvCa
OvCa
Sample_Group

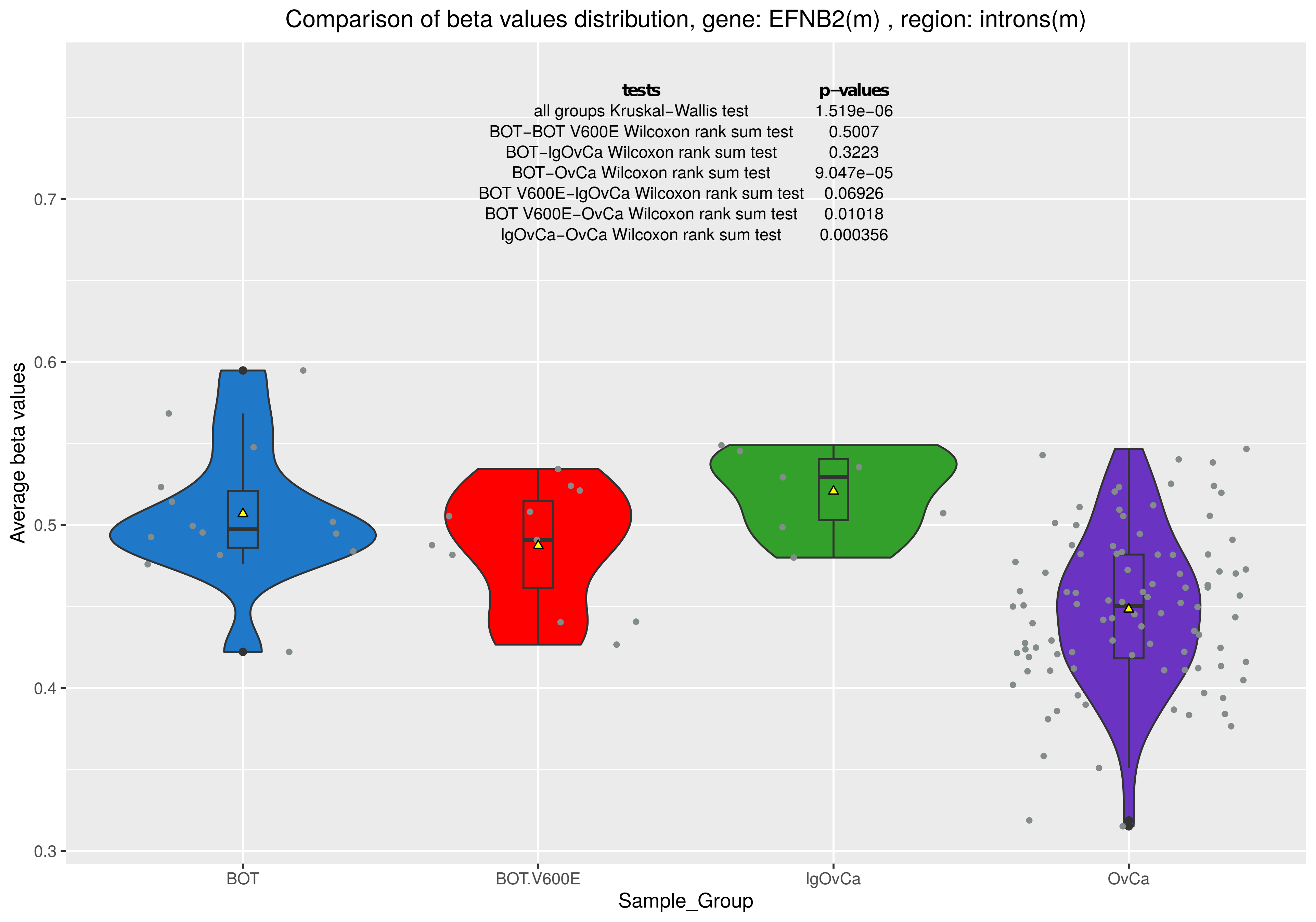

Comparison of beta values distribution, gene: EFNB2(m) , region: introns(m)
tests
p−values
all groups Kruskal−Wallis test
1.519e−06
BOT−BOT V600E Wilcoxon rank sum test
0.5007
BOT−lgOvCa Wilcoxon rank sum test
0.3223
BOT−OvCa Wilcoxon rank sum test
9.047e−05
BOT V600E−lgOvCa Wilcoxon rank sum test
0.06926
BOT V600E−OvCa Wilcoxon rank sum test
0.01018
lgOvCa−OvCa Wilcoxon rank sum test
0.000356
Average beta values
0.7
0.6
0.5
0.4
0.3
BOT
BOT.V600E
lgOvCa
OvCa
Sample_Group

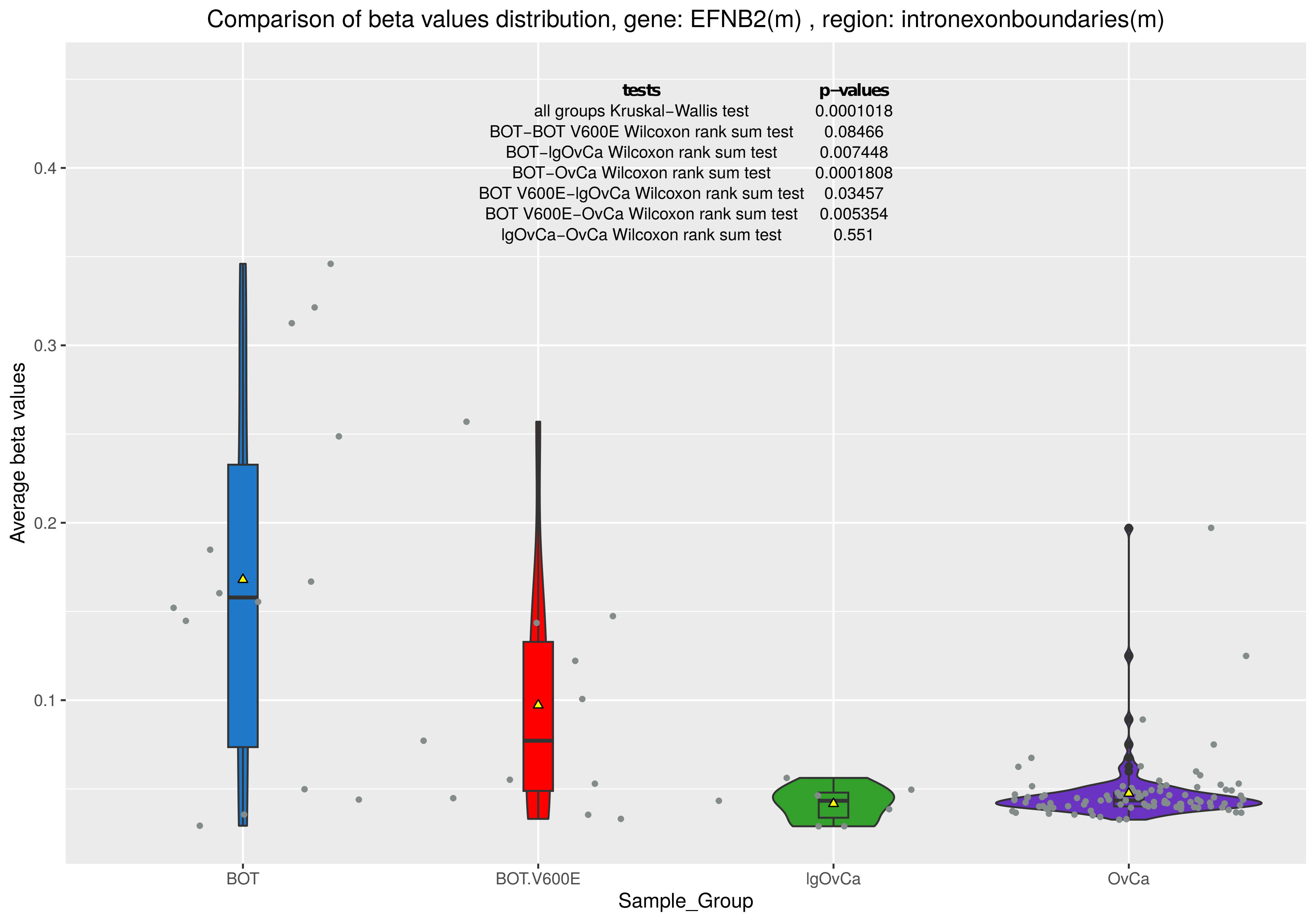
Comparison of beta values distribution, gene: EFNB2(m) , region: intronexonboundaries(m)
tests
p−values
all groups Kruskal−Wallis test
0.0001018
BOT−BOT V600E Wilcoxon rank sum test
0.08466
BOT−lgOvCa Wilcoxon rank sum test
0.007448
BOT−OvCa Wilcoxon rank sum test
0.0001808
BOT V600E−lgOvCa Wilcoxon rank sum test
0.03457
BOT V600E−OvCa Wilcoxon rank sum test
0.005354
lgOvCa−OvCa Wilcoxon rank sum test
0.551
Average beta values
0.4
0.3
0.2
0.1
BOT
BOT.V600E
lgOvCa
OvCa
Sample_Group

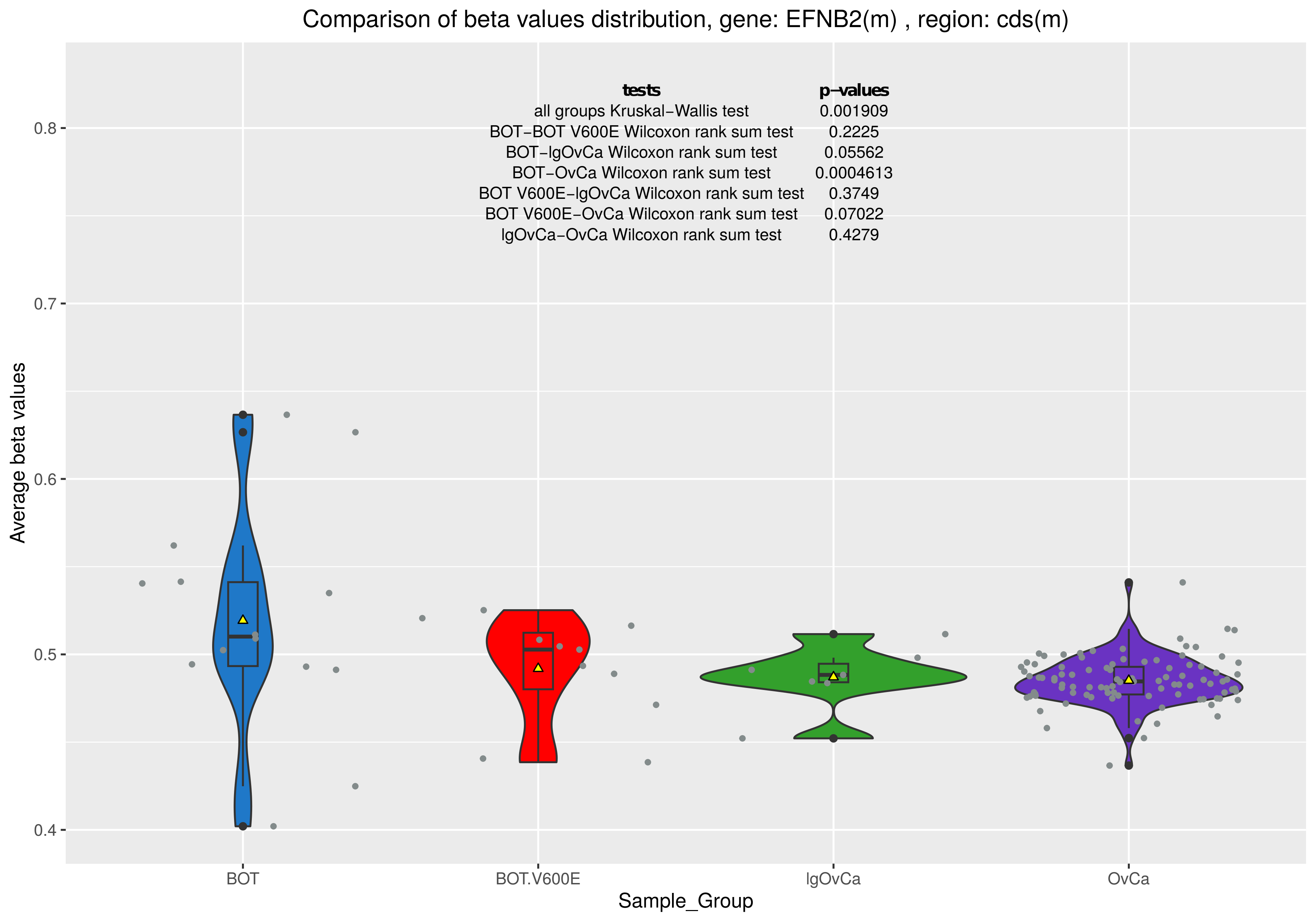
Comparison of beta values distribution, gene: EFNB2(m) , region: cds(m)
tests
p−values
all groups Kruskal−Wallis test
0.001909
BOT−BOT V600E Wilcoxon rank sum test
0.2225
BOT−lgOvCa Wilcoxon rank sum test
0.05562
BOT−OvCa Wilcoxon rank sum test
0.0004613
BOT V600E−lgOvCa Wilcoxon rank sum test
0.3749
BOT V600E−OvCa Wilcoxon rank sum test
0.07022
lgOvCa−OvCa Wilcoxon rank sum test
0.4279
Average beta values
0.8
0.7
0.6
0.5
0.4
BOT
BOT.V600E
lgOvCa
OvCa
Sample_Group

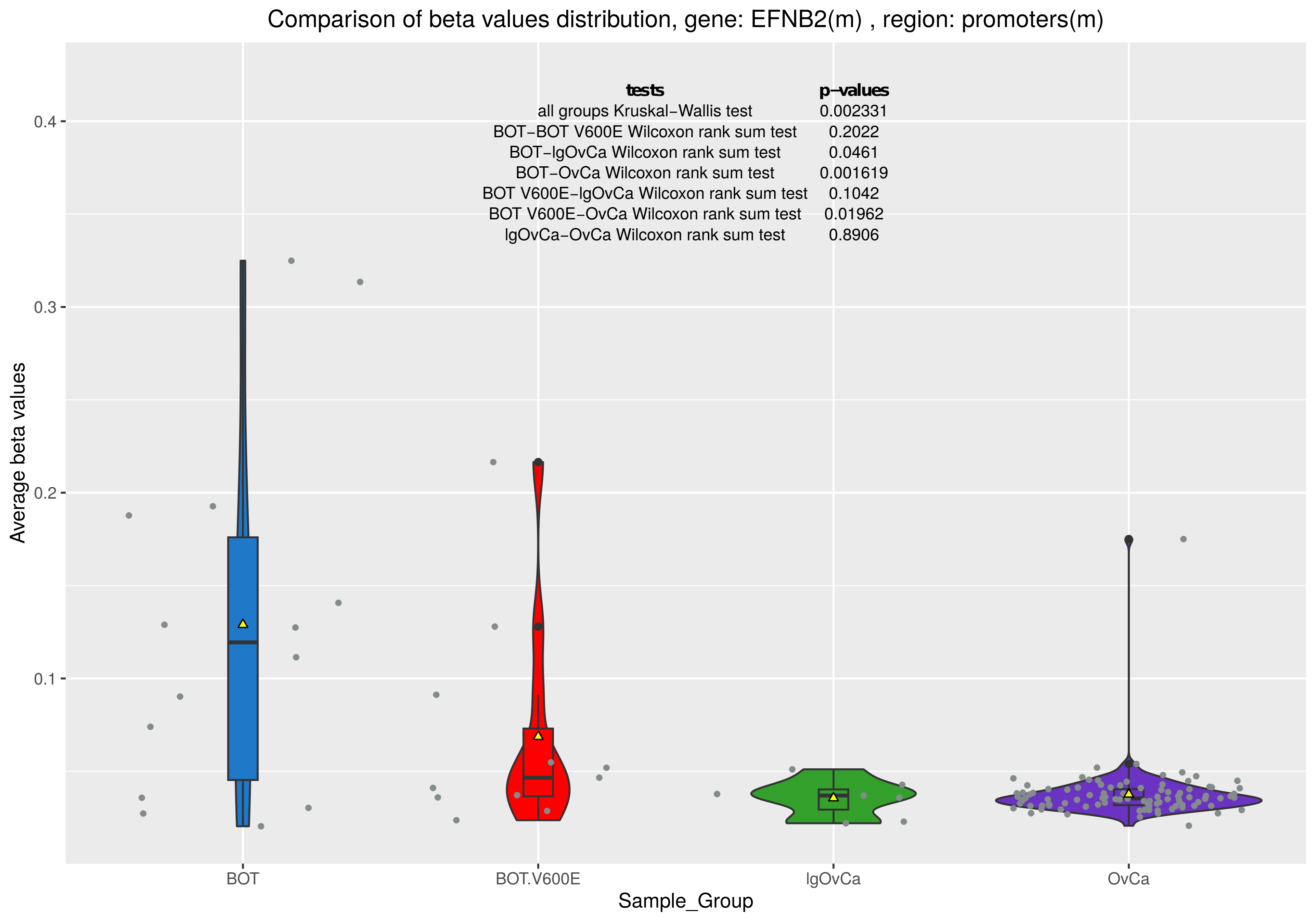

Comparison of beta values distribution, gene: EFNB2(m) , region: promoters(m)
tests
p−values
all groups Kruskal−Wallis test 0.002331
BOT−BOT V600E Wilcoxon rank sum test 0.2022
BOT−lgOvCa Wilcoxon rank sum test 0.0461
BOT−OvCa Wilcoxon rank sum test 0.001619
BOT V600E−lgOvCa Wilcoxon rank sum test 0.1042
BOT V600E−OvCa Wilcoxon rank sum test 0.01962
lgOvCa−OvCa Wilcoxon rank sum test 0.8906
Average beta values
0.4
0.3
0.2
0.1
BOT
BOT.V600E
lgOvCa
OvCa
Sample_Group

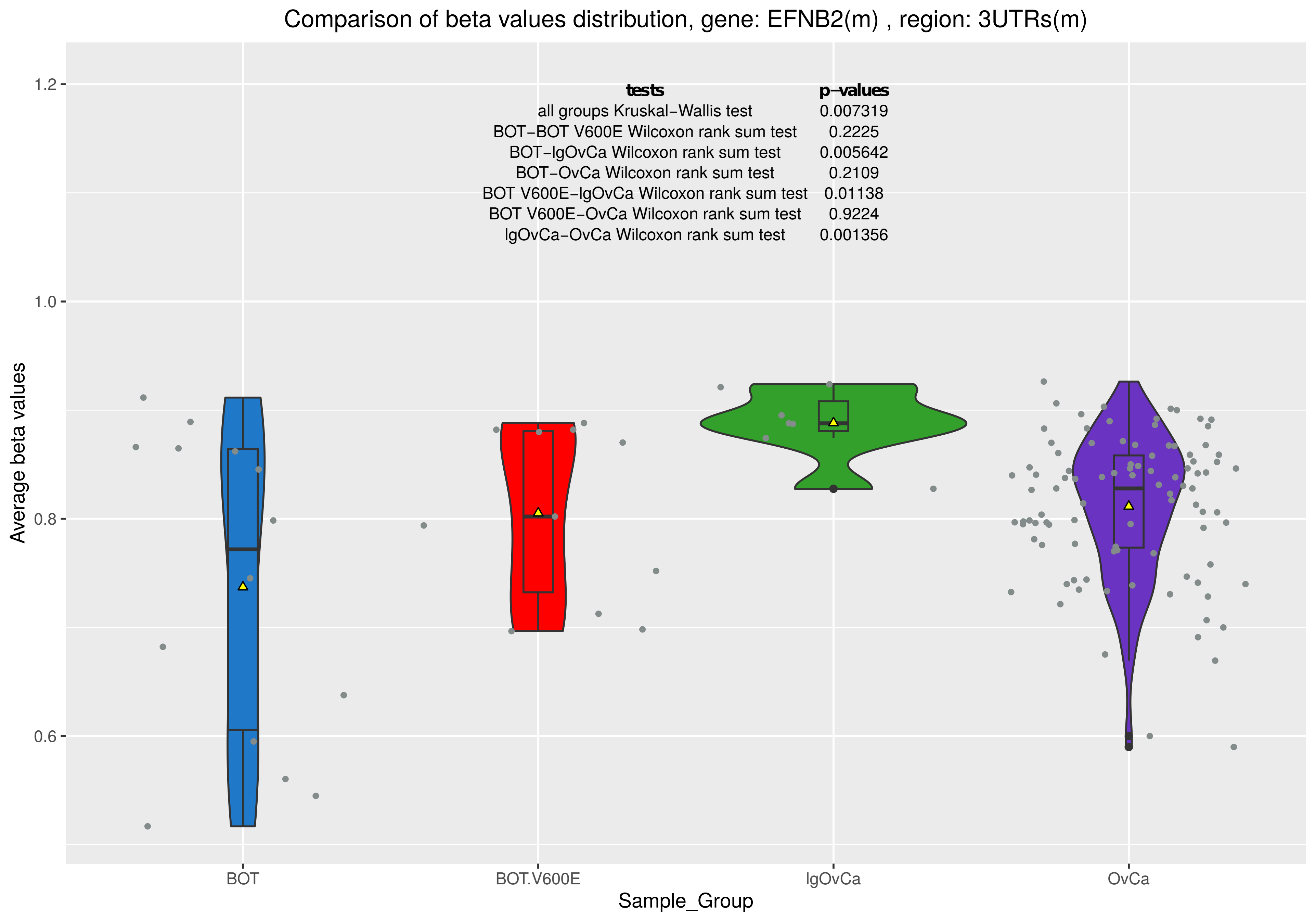

Comparison of beta values distribution, gene: EFNB2(m) , region: 3UTRs(m)
tests
p−values
all groups Kruskal−Wallis test
0.007319
BOT−BOT V600E Wilcoxon rank sum test
0.2225
BOT−lgOvCa Wilcoxon rank sum test
0.005642
BOT−OvCa Wilcoxon rank sum test
0.2109
BOT V600E−lgOvCa Wilcoxon rank sum test
0.01138
BOT V600E−OvCa Wilcoxon rank sum test
0.9224
lgOvCa−OvCa Wilcoxon rank sum test
0.001356
Average beta values
1.2
1.0
0.8
0.6
BOT
BOT.V600E
lgOvCa
OvCa
Sample_Group

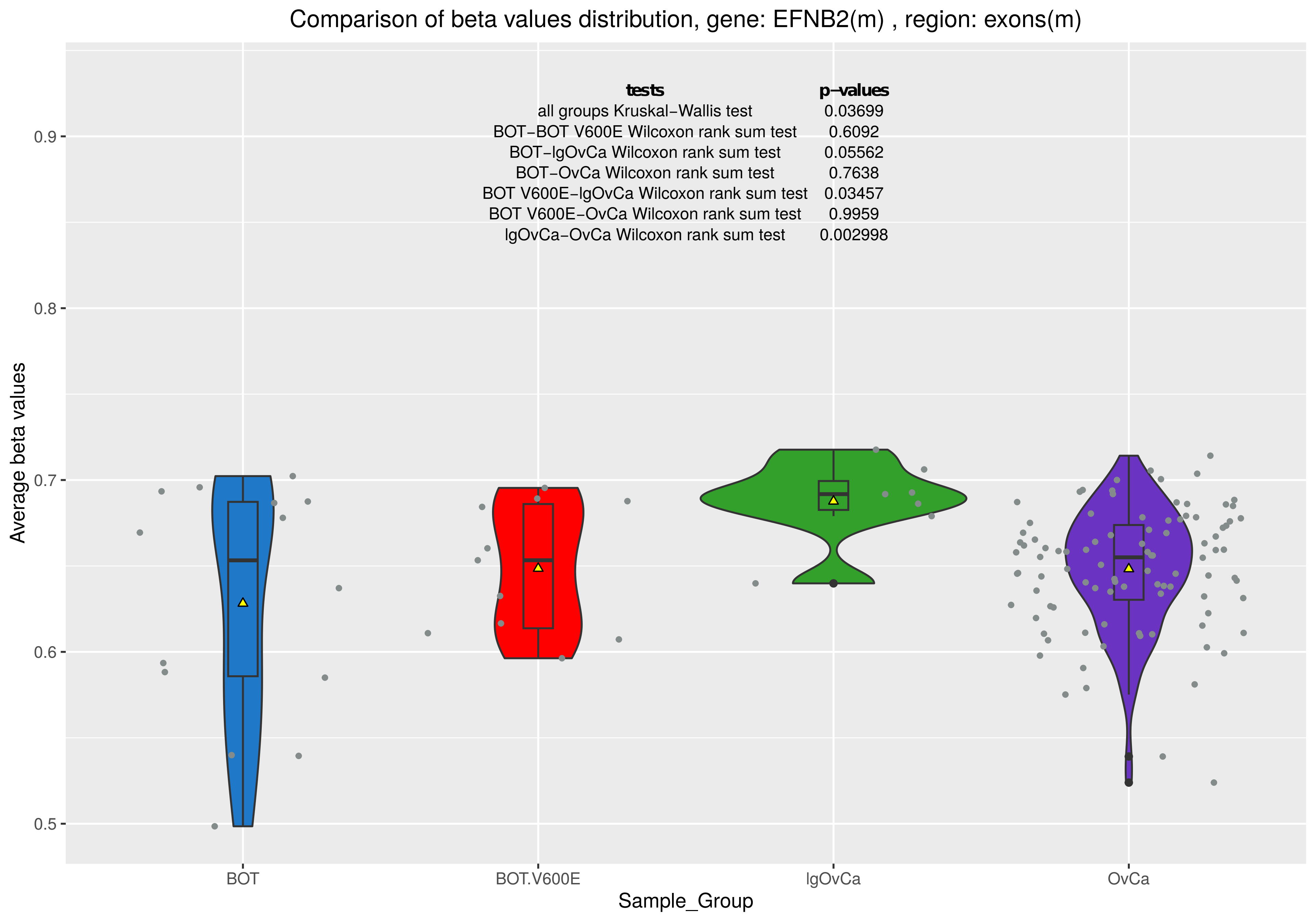
Comparison of beta values distribution, gene: EFNB2(m) , region: exons(m)
tests
p−values
all groups Kruskal−Wallis test
0.03699
BOT−BOT V600E Wilcoxon rank sum test
0.6092
BOT−lgOvCa Wilcoxon rank sum test
0.05562
BOT−OvCa Wilcoxon rank sum test
0.7638
BOT V600E−lgOvCa Wilcoxon rank sum test
0.03457
BOT V600E−OvCa Wilcoxon rank sum test
0.9959
lgOvCa−OvCa Wilcoxon rank sum test
0.002998
Average beta values
0.9
0.8
0.7
0.6
0.5
BOT
BOT.V600E
lgOvCa
OvCa
Sample_Group

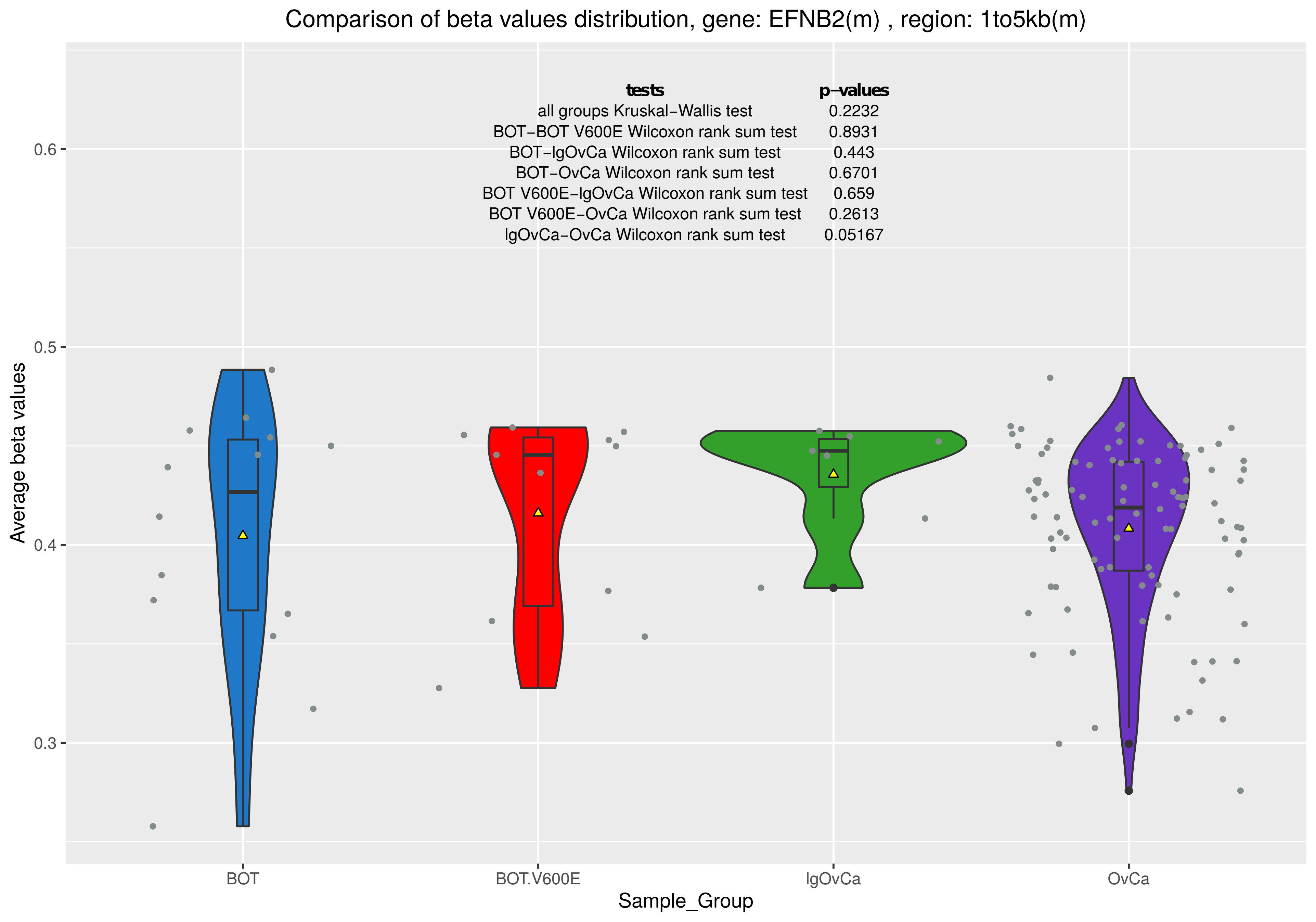
Comparison of beta values distribution, gene: EFNB2(m) , region: 1to5kb(m)
tests
p−values
all groups Kruskal−Wallis test
0.2232
BOT−BOT V600E Wilcoxon rank sum test
0.8931
BOT−lgOvCa Wilcoxon rank sum test
0.443
BOT−OvCa Wilcoxon rank sum test
0.6701
BOT V600E−lgOvCa Wilcoxon rank sum test
0.659
BOT V600E−OvCa Wilcoxon rank sum test
0.2613
lgOvCa−OvCa Wilcoxon rank sum test
0.05167
Average beta values
0.6
0.5
0.4
0.3
BOT
BOT.V600E
lgOvCa
OvCa
Sample_Group

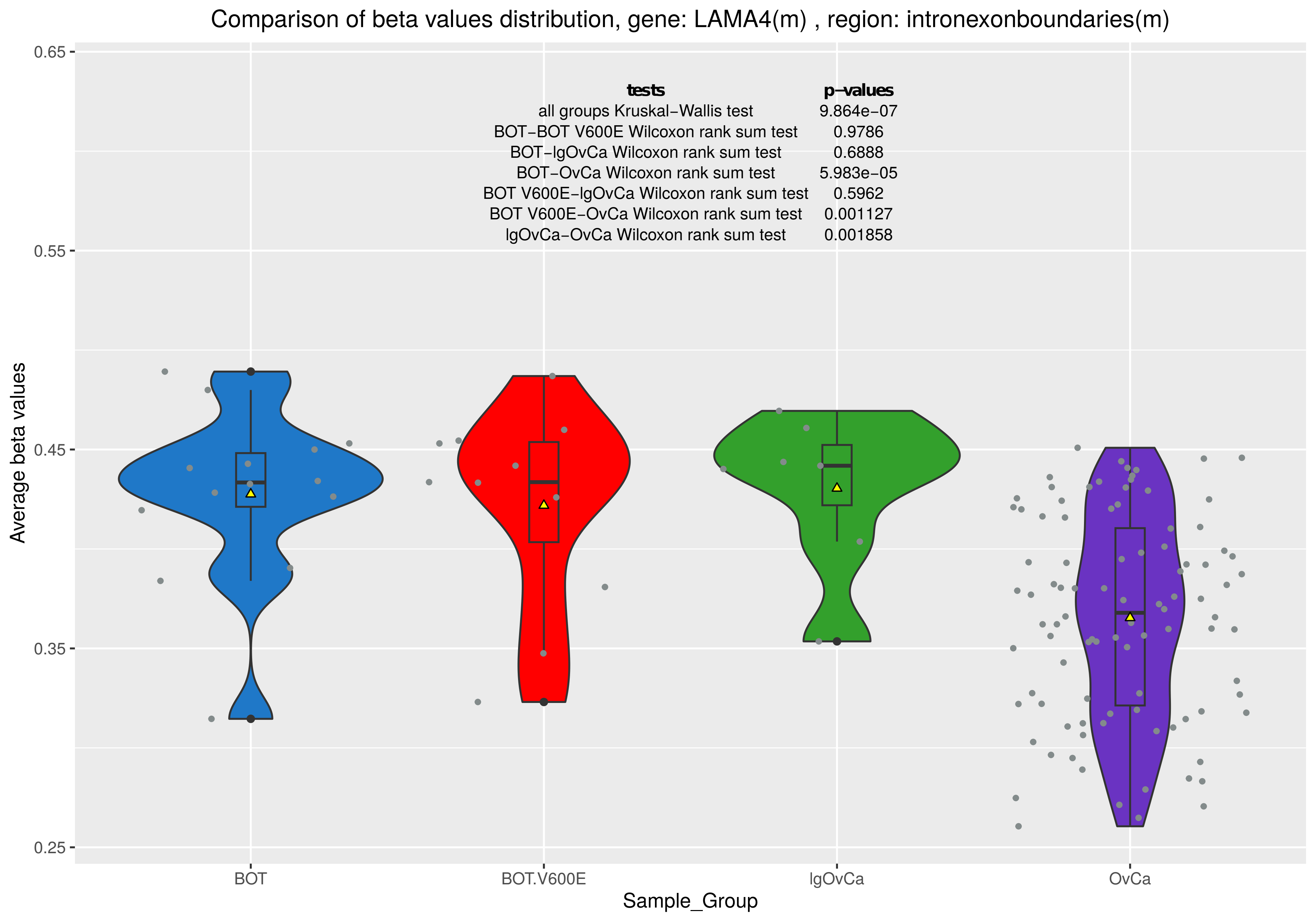
Comparison of beta values distribution, gene: LAMA4(m) , region: intronexonboundaries(m)
tests
p−values
all groups Kruskal−Wallis test
9.864e−07
BOT−BOT V600E Wilcoxon rank sum test
0.9786
BOT−lgOvCa Wilcoxon rank sum test
0.6888
BOT−OvCa Wilcoxon rank sum test
5.983e−05
BOT V600E−lgOvCa Wilcoxon rank sum test
0.5962
BOT V600E−OvCa Wilcoxon rank sum test
0.001127
lgOvCa−OvCa Wilcoxon rank sum test
0.001858
Average beta values
0.65
0.55
0.45
0.35
0.25
BOT
BOT.V600E
lgOvCa
OvCa
Sample_Group

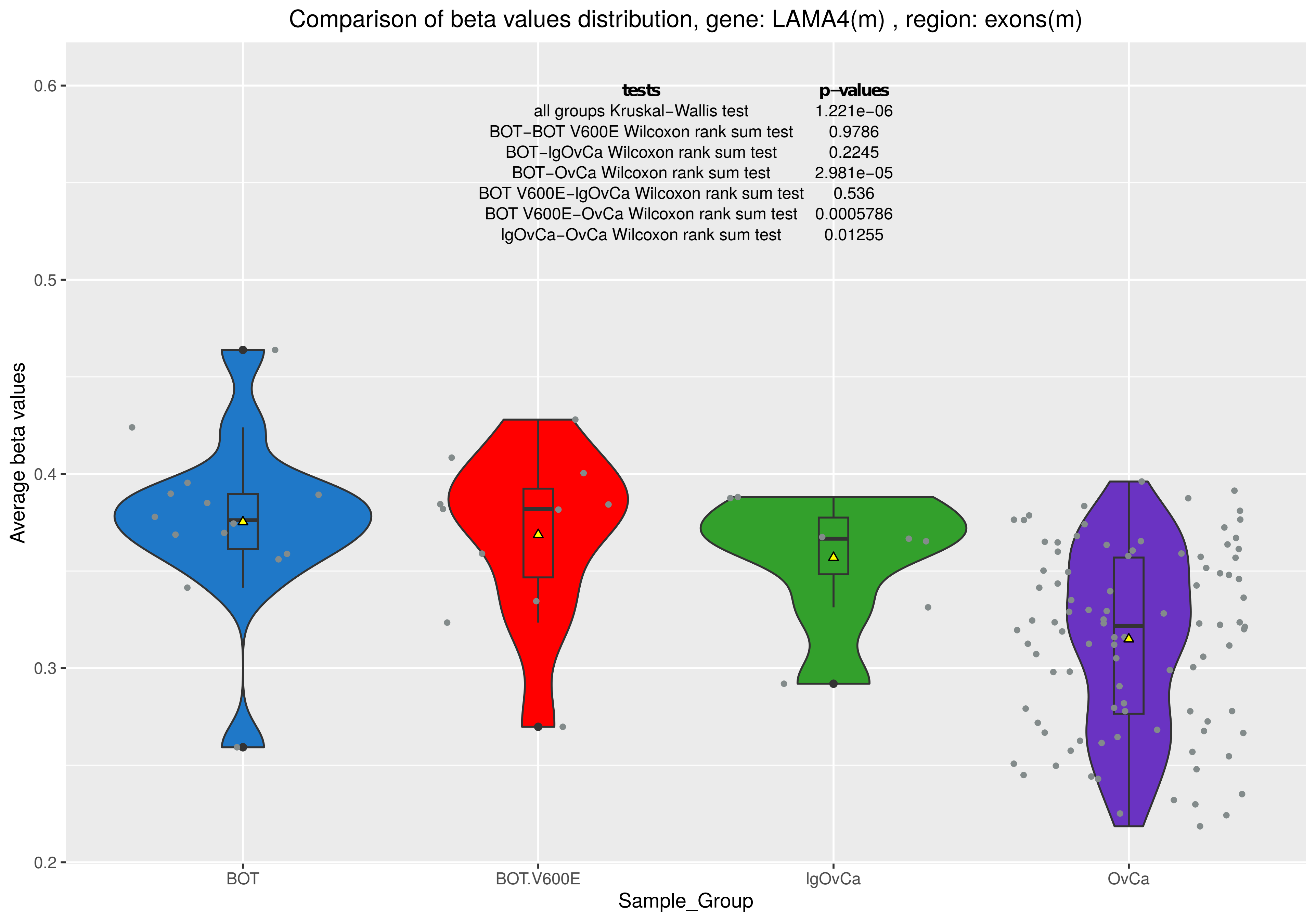
Comparison of beta values distribution, gene: LAMA4(m) , region: exons(m)
tests
p−values
all groups Kruskal−Wallis test
1.221e−06
BOT−BOT V600E Wilcoxon rank sum test
0.9786
BOT−lgOvCa Wilcoxon rank sum test
0.2245
BOT−OvCa Wilcoxon rank sum test
2.981e−05
BOT V600E−lgOvCa Wilcoxon rank sum test
0.536
BOT V600E−OvCa Wilcoxon rank sum test
0.0005786
lgOvCa−OvCa Wilcoxon rank sum test
0.01255
Average beta values
0.6
0.5
0.4
0.3
0.2
BOT
BOT.V600E
lgOvCa
OvCa
Sample_Group

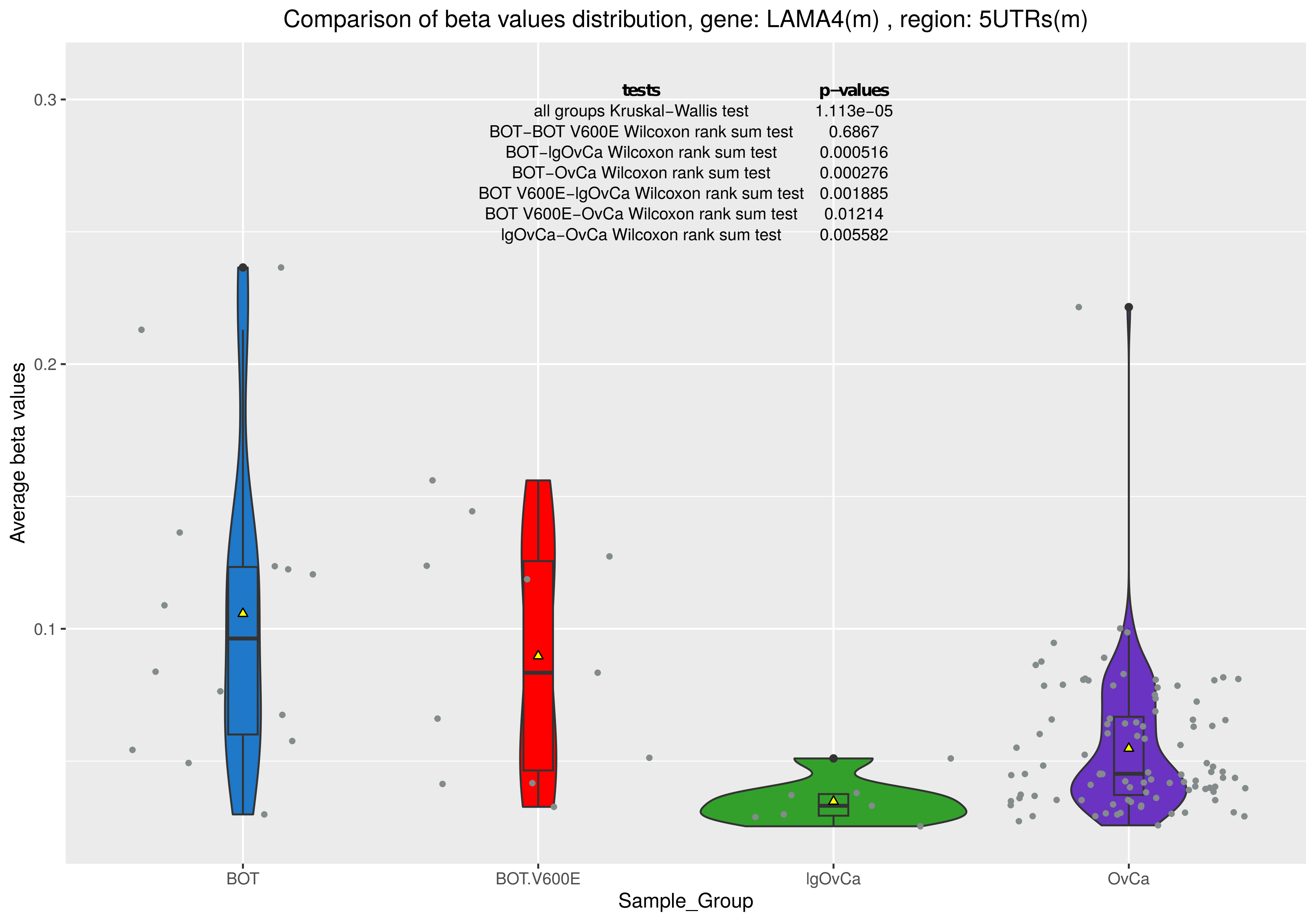
Comparison of beta values distribution, gene: LAMA4(m) , region: 5UTRs(m)
tests
p−values
all groups Kruskal−Wallis test
1.113e−05
BOT−BOT V600E Wilcoxon rank sum test
0.6867
BOT−lgOvCa Wilcoxon rank sum test
0.000516
BOT−OvCa Wilcoxon rank sum test
0.000276
BOT V600E−lgOvCa Wilcoxon rank sum test
0.001885
BOT V600E−OvCa Wilcoxon rank sum test
0.01214
lgOvCa−OvCa Wilcoxon rank sum test
0.005582
Average beta values
0.3
0.2
0.1
BOT
BOT.V600E
lgOvCa
OvCa
Sample_Group

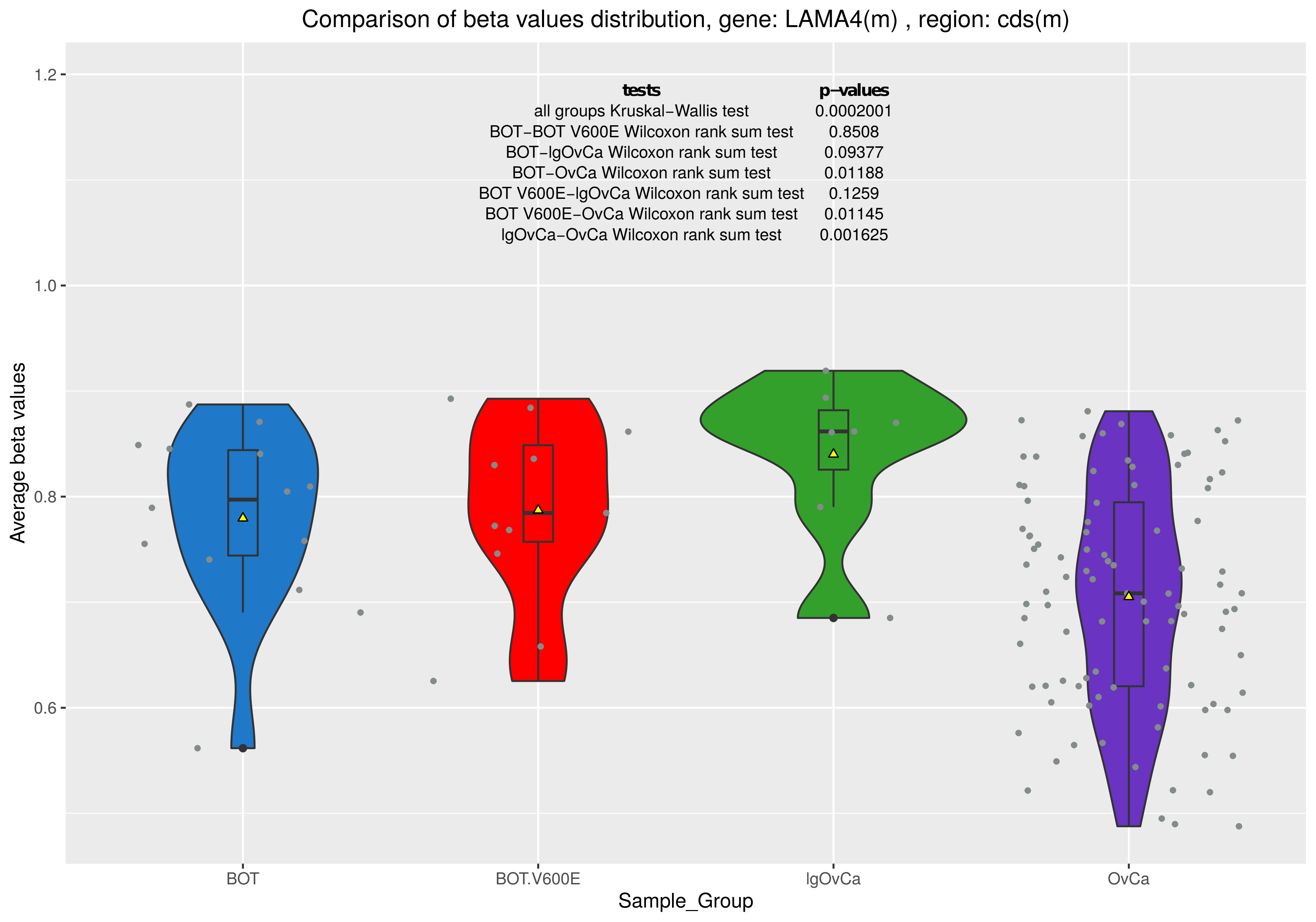

Comparison of beta values distribution, gene: LAMA4(m) , region: cds(m)
tests
p−values
all groups Kruskal−Wallis test
0.0002001
BOT−BOT V600E Wilcoxon rank sum test
0.8508
BOT−lgOvCa Wilcoxon rank sum test
0.09377
BOT−OvCa Wilcoxon rank sum test
0.01188
BOT V600E−lgOvCa Wilcoxon rank sum test
0.1259
BOT V600E−OvCa Wilcoxon rank sum test
0.01145
lgOvCa−OvCa Wilcoxon rank sum test
0.001625
Average beta values
1.2
1.0
0.8
0.6
BOT
BOT.V600E
lgOvCa
OvCa
Sample_Group

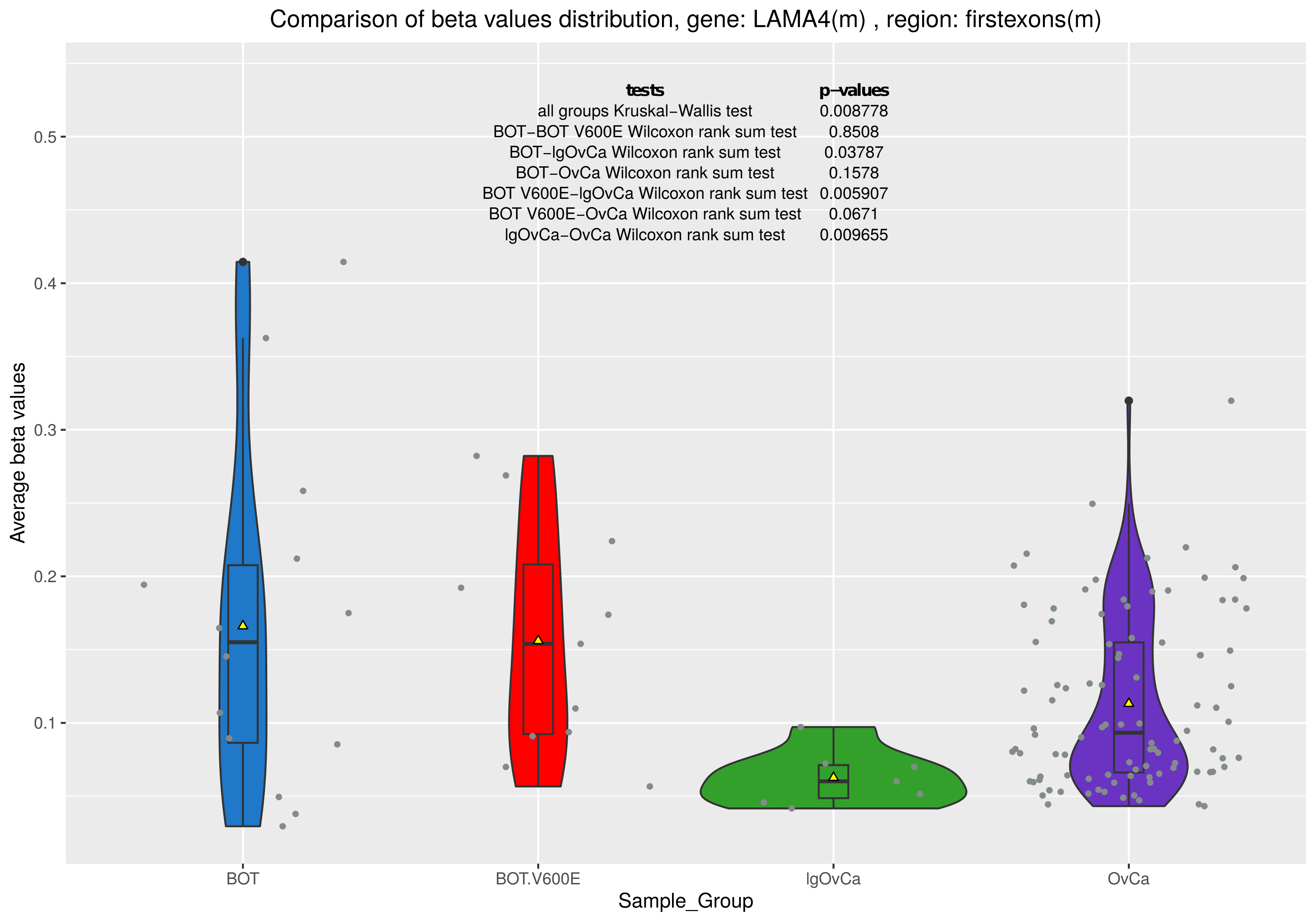

Comparison of beta values distribution, gene: LAMA4(m) , region: firstexons(m)
tests
p−values
all groups Kruskal−Wallis test 0.008778
BOT−BOT V600E Wilcoxon rank sum test 0.8508
BOT−IgOvCa Wilcoxon rank sum test 0.03787
BOT−OvCa Wilcoxon rank sum test 0.1578
BOT V600E−IgOvCa Wilcoxon rank sum test 0.005907
BOT V600E−OvCa Wilcoxon rank sum test 0.0671
IgOvCa−OvCa Wilcoxon rank sum test 0.009655
Average beta values
0.5
0.4
0.3
0.2
0.1
BOT
BOT.V600E
IgOvCa
OvCa
Sample_Group

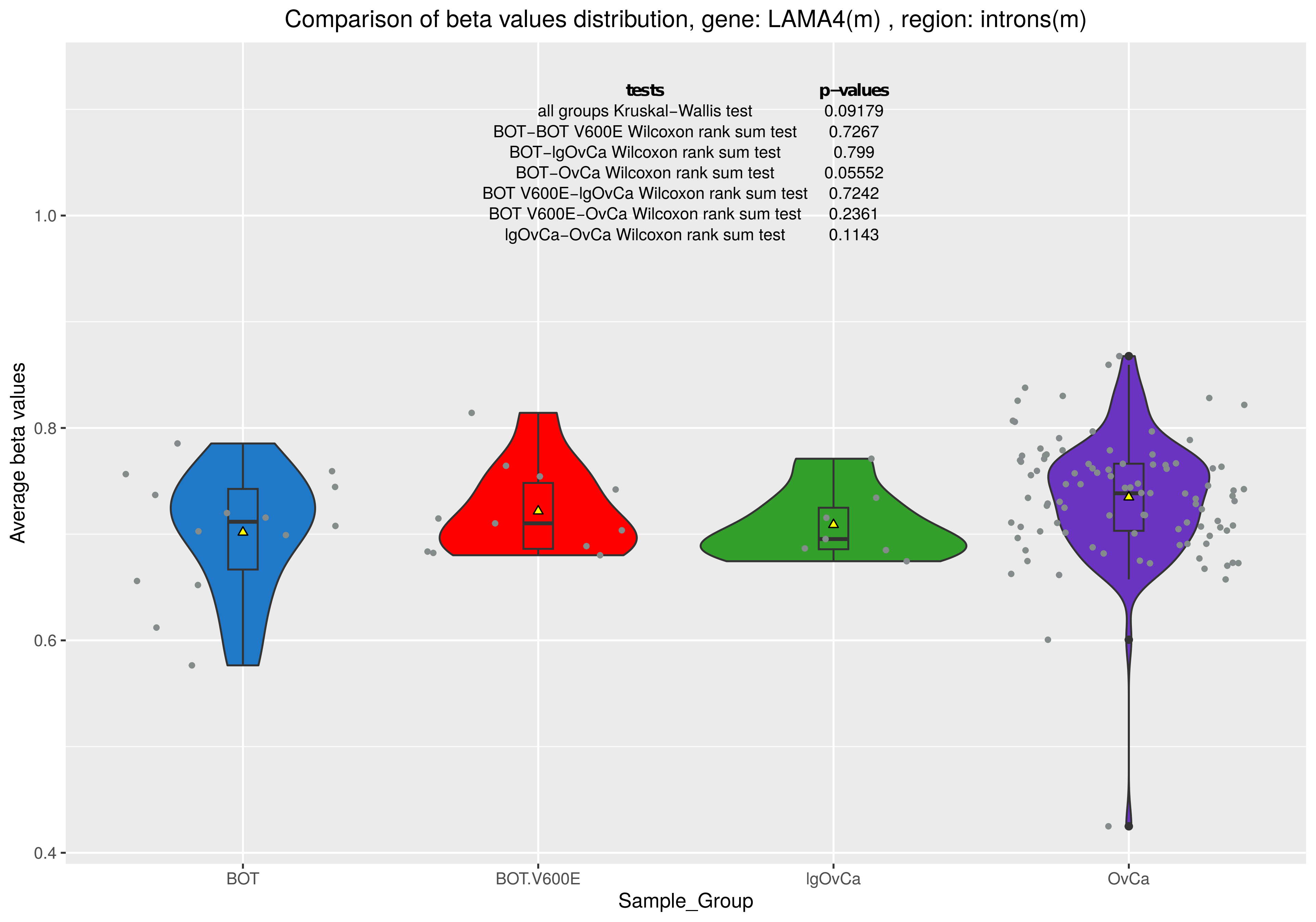
Comparison of beta values distribution, gene: LAMA4(m) , region: introns(m)
tests
p−values
all groups Kruskal−Wallis test
0.09179
BOT−BOT V600E Wilcoxon rank sum test
0.7267
BOT−lgOvCa Wilcoxon rank sum test
0.799
BOT−OvCa Wilcoxon rank sum test
0.05552
BOT V600E−lgOvCa Wilcoxon rank sum test
0.7242
BOT V600E−OvCa Wilcoxon rank sum test
0.2361
lgOvCa−OvCa Wilcoxon rank sum test
0.1143
Average beta values
1.0
0.8
0.6
0.4
BOT
BOT.V600E
lgOvCa
OvCa
Sample_Group

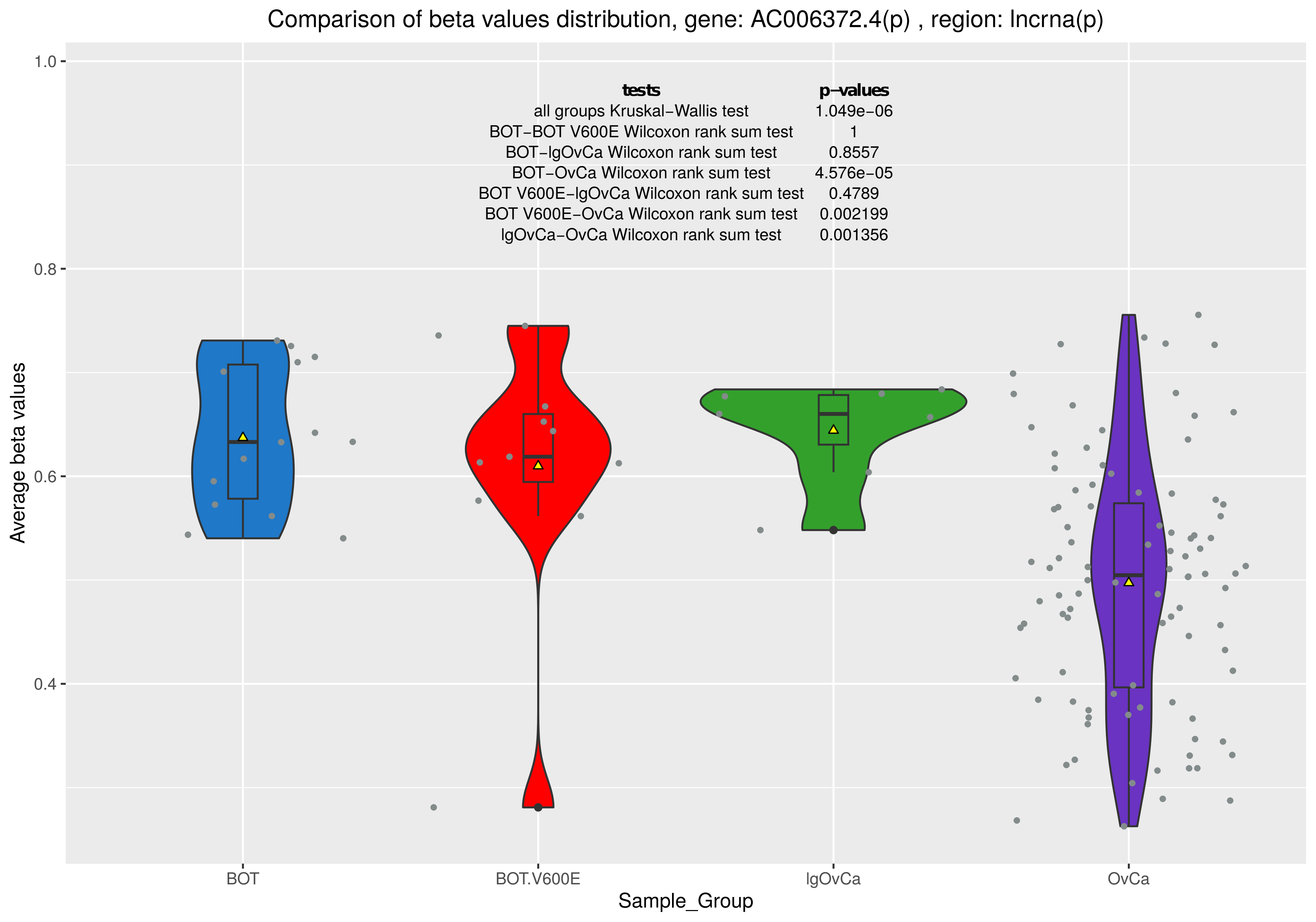
Comparison of beta values distribution, gene: AC006372.4(p) , region: lncrna(p)
tests
p−values
all groups Kruskal−Wallis test
1.049e−06
BOT−BOT V600E Wilcoxon rank sum test
1
BOT−lgOvCa Wilcoxon rank sum test
0.8557
BOT−OvCa Wilcoxon rank sum test
4.576e−05
BOT V600E−lgOvCa Wilcoxon rank sum test
0.4789
BOT V600E−OvCa Wilcoxon rank sum test
0.002199
lgOvCa−OvCa Wilcoxon rank sum test
0.001356
Average beta values
1.0
0.8
0.6
0.4
BOT
BOT.V600E
lgOvCa
OvCa
Sample_Group

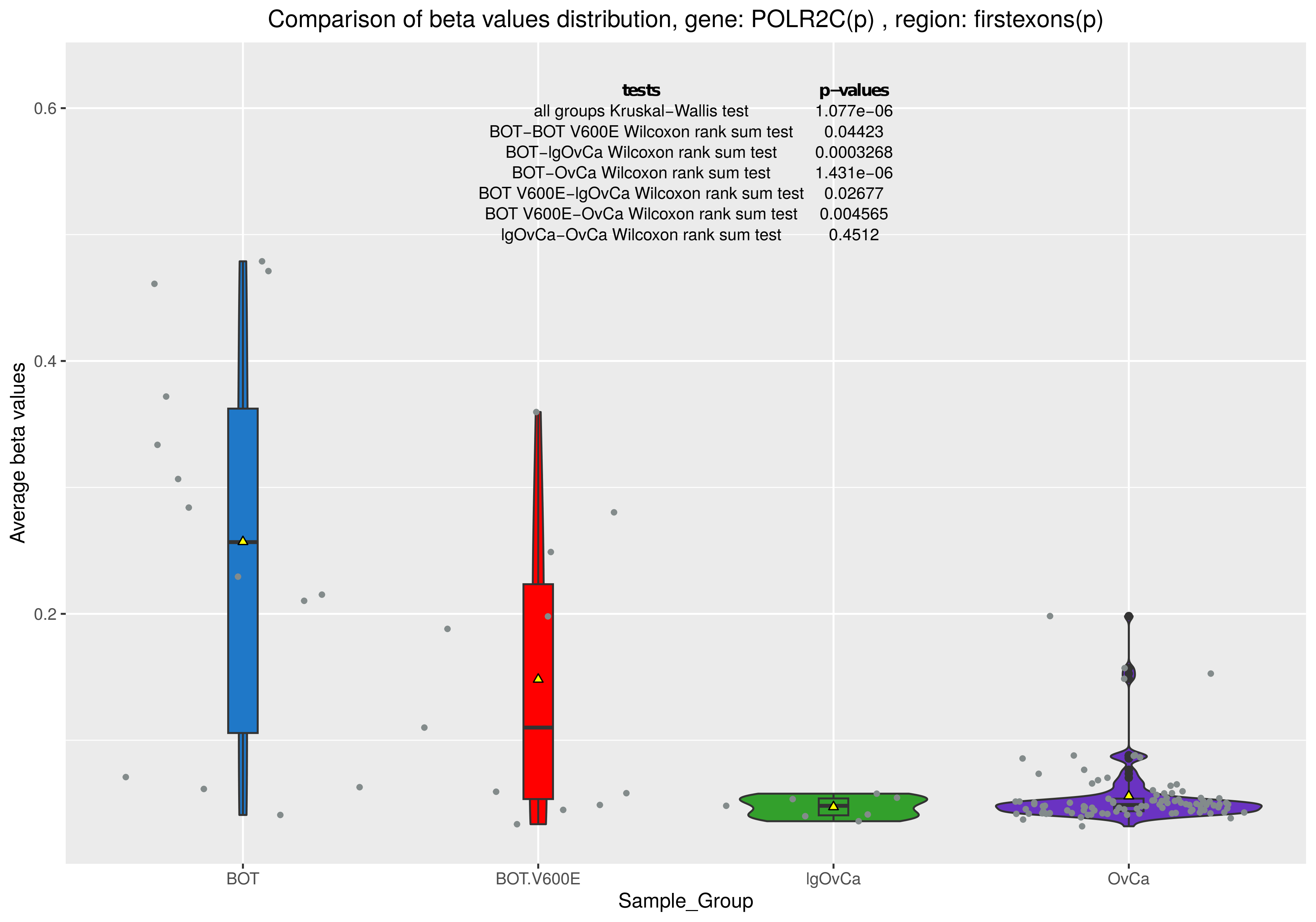
Comparison of beta values distribution, gene: POLR2C(p) , region: firstexons(p)
tests
p−values
all groups Kruskal−Wallis test
1.077e−06
BOT−BOT V600E Wilcoxon rank sum test
0.04423
BOT−lgOvCa Wilcoxon rank sum test
0.0003268
BOT−OvCa Wilcoxon rank sum test
1.431e−06
BOT V600E−lgOvCa Wilcoxon rank sum test
0.02677
BOT V600E−OvCa Wilcoxon rank sum test
0.004565
lgOvCa−OvCa Wilcoxon rank sum test
0.4512
Average beta values
0.6
0.4
0.2
BOT
BOT.V600E
lgOvCa
OvCa
Sample_Group

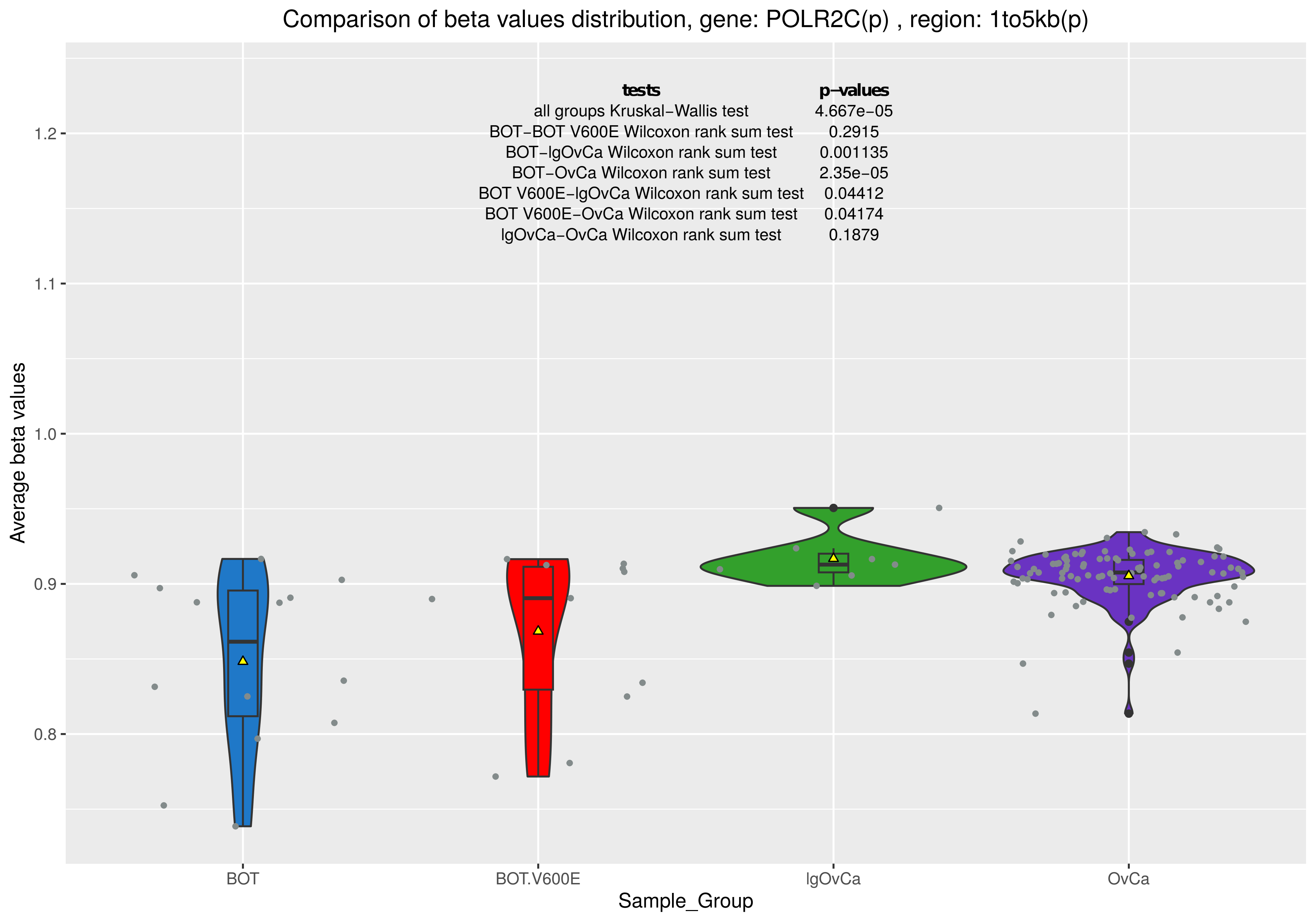
Comparison of beta values distribution, gene: POLR2C(p) , region: 1to5kb(p)
tests
p−values
all groups Kruskal−Wallis test
4.667e−05
BOT−BOT V600E Wilcoxon rank sum test
0.2915
BOT−lgOvCa Wilcoxon rank sum test
0.001135
BOT−OvCa Wilcoxon rank sum test
2.35e−05
BOT V600E−lgOvCa Wilcoxon rank sum test
0.04412
BOT V600E−OvCa Wilcoxon rank sum test
0.04174
lgOvCa−OvCa Wilcoxon rank sum test
0.1879
Average beta values
1.2
1.1
1.0
0.9
0.8
BOT
BOT.V600E
lgOvCa
OvCa
Sample_Group

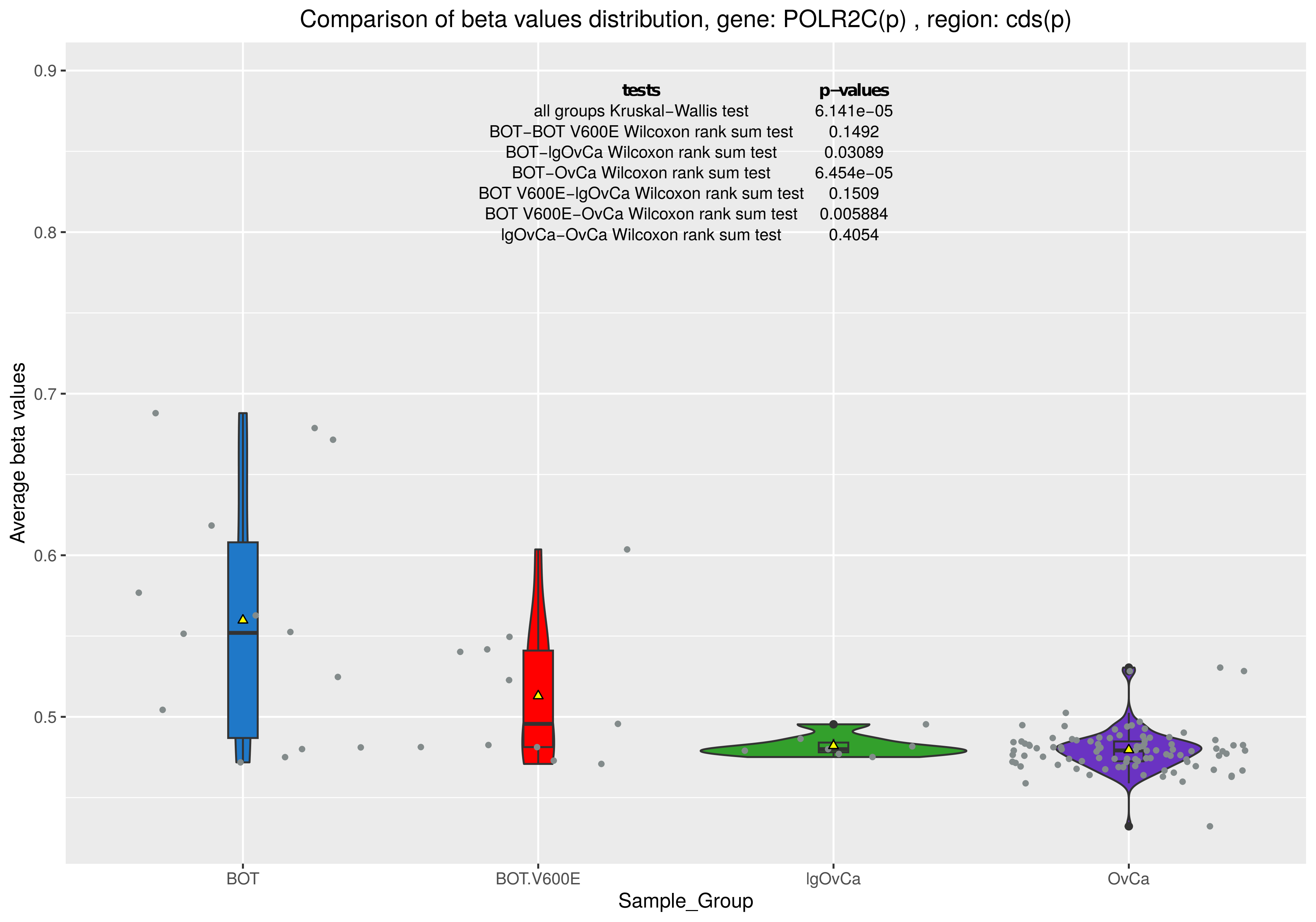

Comparison of beta values distribution, gene: POLR2C(p) , region: cds(p)
tests
p−values
all groups Kruskal−Wallis test
6.141e−05
BOT−BOT V600E Wilcoxon rank sum test
0.1492
BOT−lgOvCa Wilcoxon rank sum test
0.03089
BOT−OvCa Wilcoxon rank sum test
6.454e−05
BOT V600E−lgOvCa Wilcoxon rank sum test
0.1509
BOT V600E−OvCa Wilcoxon rank sum test
0.005884
lgOvCa−OvCa Wilcoxon rank sum test
0.4054
Average beta values
0.9
0.8
0.7
0.6
0.5
BOT
BOT.V600E
lgOvCa
OvCa
Sample_Group

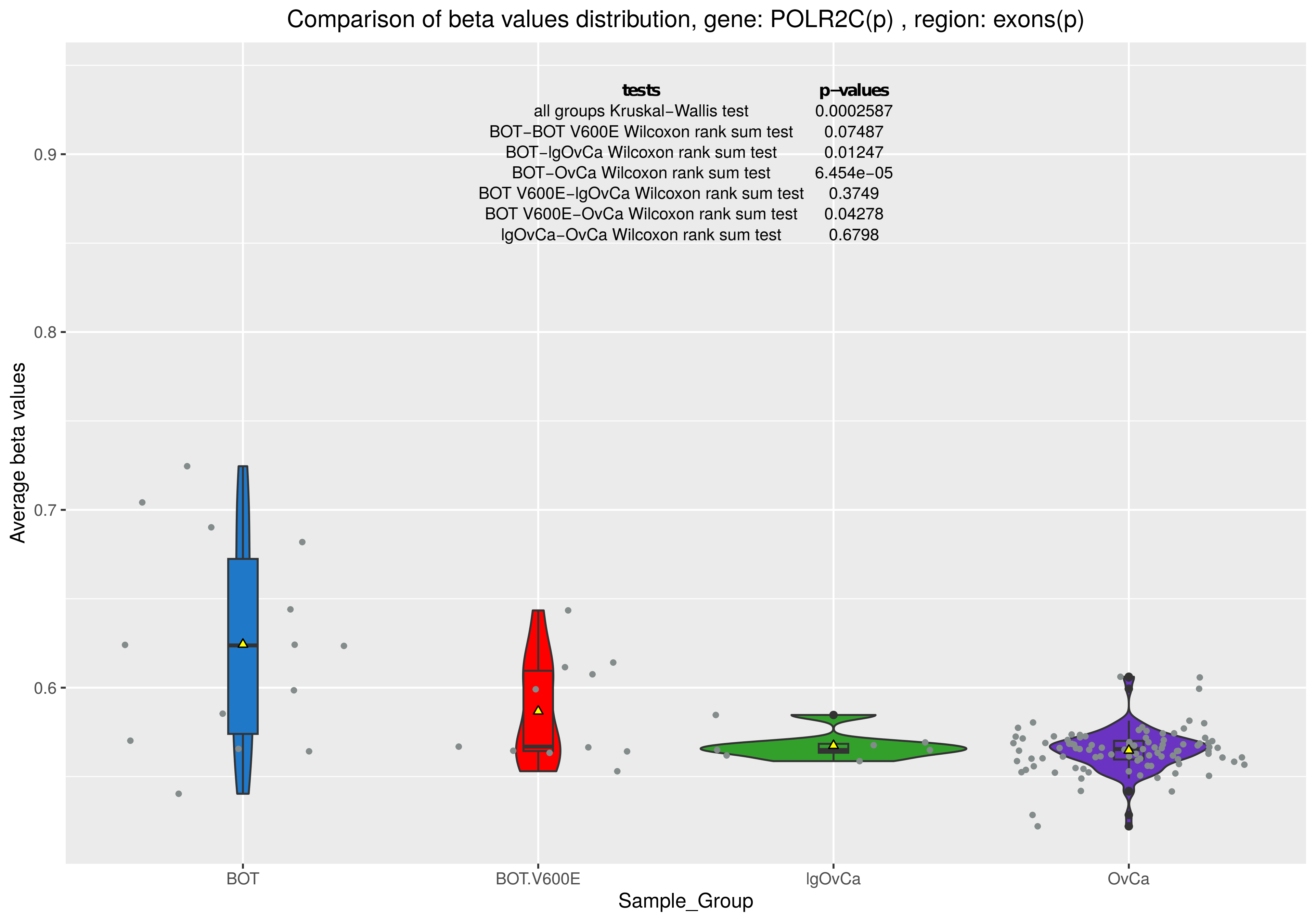
Comparison of beta values distribution, gene: POLR2C(p) , region: exons(p)
tests
p−values
all groups Kruskal−Wallis test
0.0002587
BOT−BOT V600E Wilcoxon rank sum test
0.07487
BOT−lgOvCa Wilcoxon rank sum test
0.01247
BOT−OvCa Wilcoxon rank sum test
6.454e−05
BOT V600E−lgOvCa Wilcoxon rank sum test
0.3749
BOT V600E−OvCa Wilcoxon rank sum test
0.04278
lgOvCa−OvCa Wilcoxon rank sum test
0.6798
Average beta values
0.9
0.8
0.7
0.6
BOT
BOT.V600E
lgOvCa
OvCa
Sample_Group

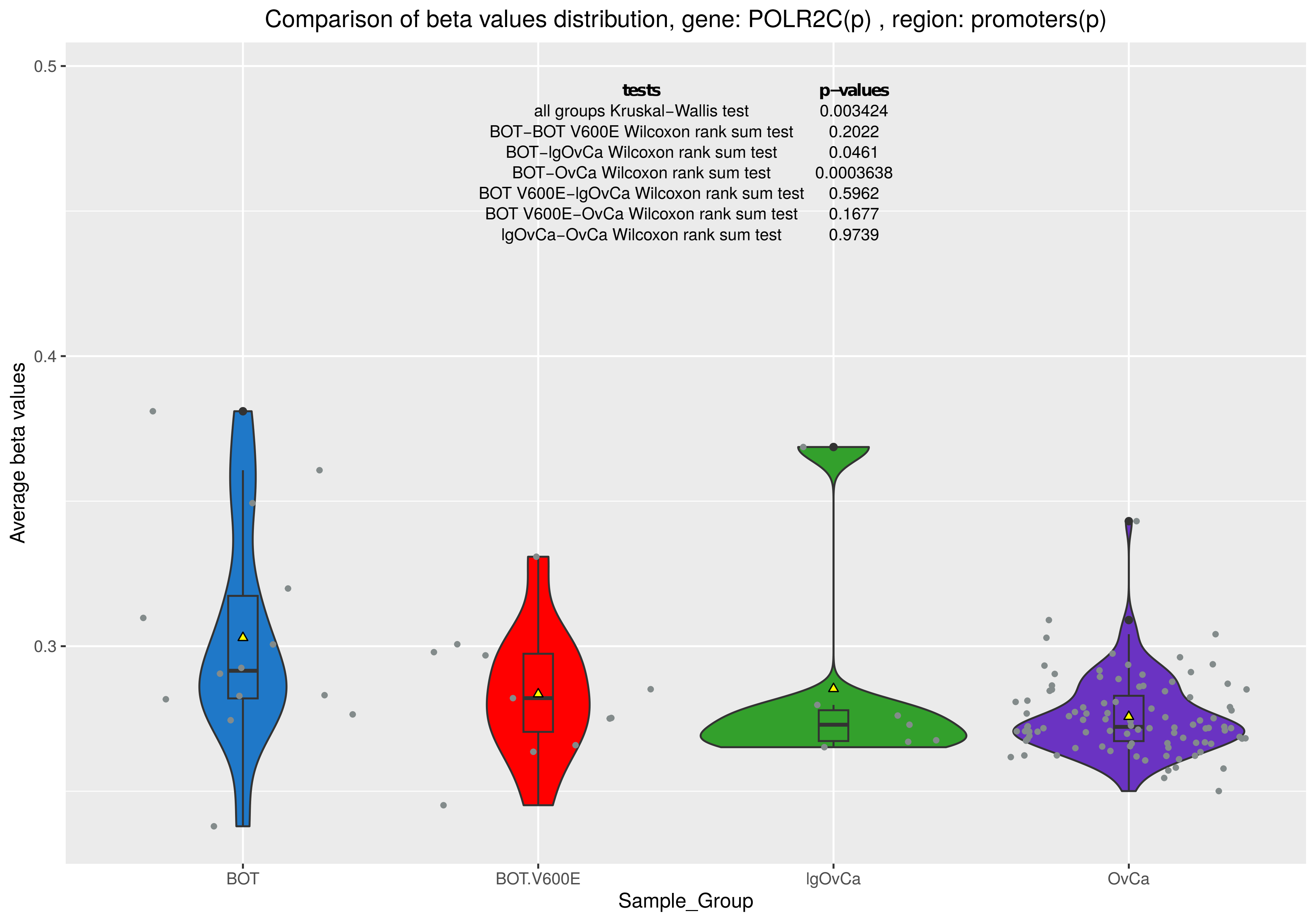

Comparison of beta values distribution, gene: POLR2C(p) , region: promoters(p)
tests
p−values
all groups Kruskal−Wallis test
0.003424
BOT−BOT V600E Wilcoxon rank sum test
0.2022
BOT−lgOvCa Wilcoxon rank sum test
0.0461
BOT−OvCa Wilcoxon rank sum test
0.0003638
BOT V600E−lgOvCa Wilcoxon rank sum test
0.5962
BOT V600E−OvCa Wilcoxon rank sum test
0.1677
lgOvCa−OvCa Wilcoxon rank sum test
0.9739
Average beta values
0.5
0.4
0.3
BOT
BOT.V600E
lgOvCa
OvCa
Sample_Group

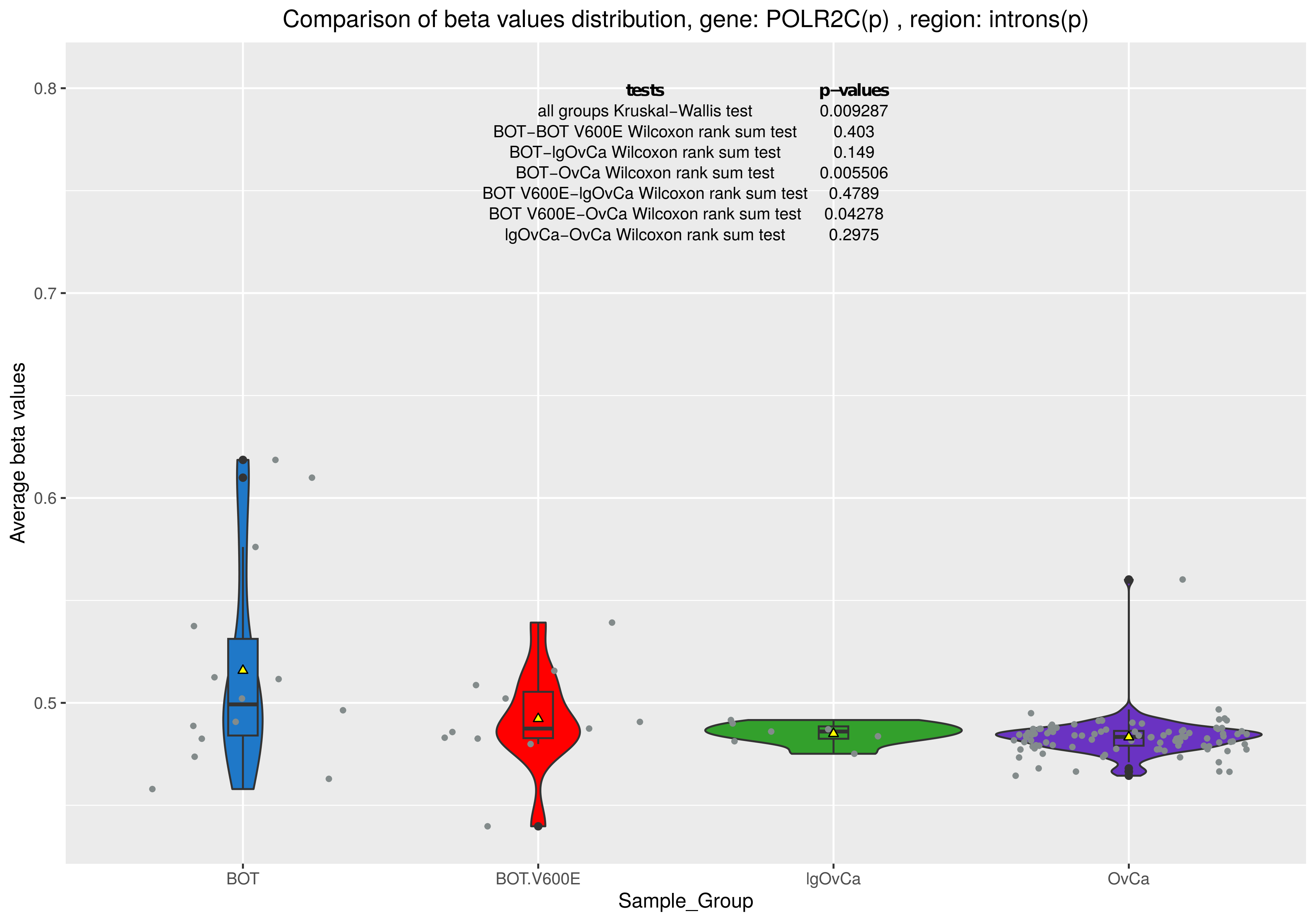

Comparison of beta values distribution, gene: POLR2C(p) , region: introns(p)
tests
p−values
all groups Kruskal−Wallis test 0.009287
BOT−BOT V600E Wilcoxon rank sum test 0.403
BOT−IgOvCa Wilcoxon rank sum test 0.149
BOT−OvCa Wilcoxon rank sum test 0.005506
BOT V600E−IgOvCa Wilcoxon rank sum test 0.4789
BOT V600E−OvCa Wilcoxon rank sum test 0.04278
IgOvCa−OvCa Wilcoxon rank sum test 0.2975
Average beta values
0.8
0.7
0.6
0.5
BOT
BOT.V600E
IgOvCa
OvCa
Sample_Group

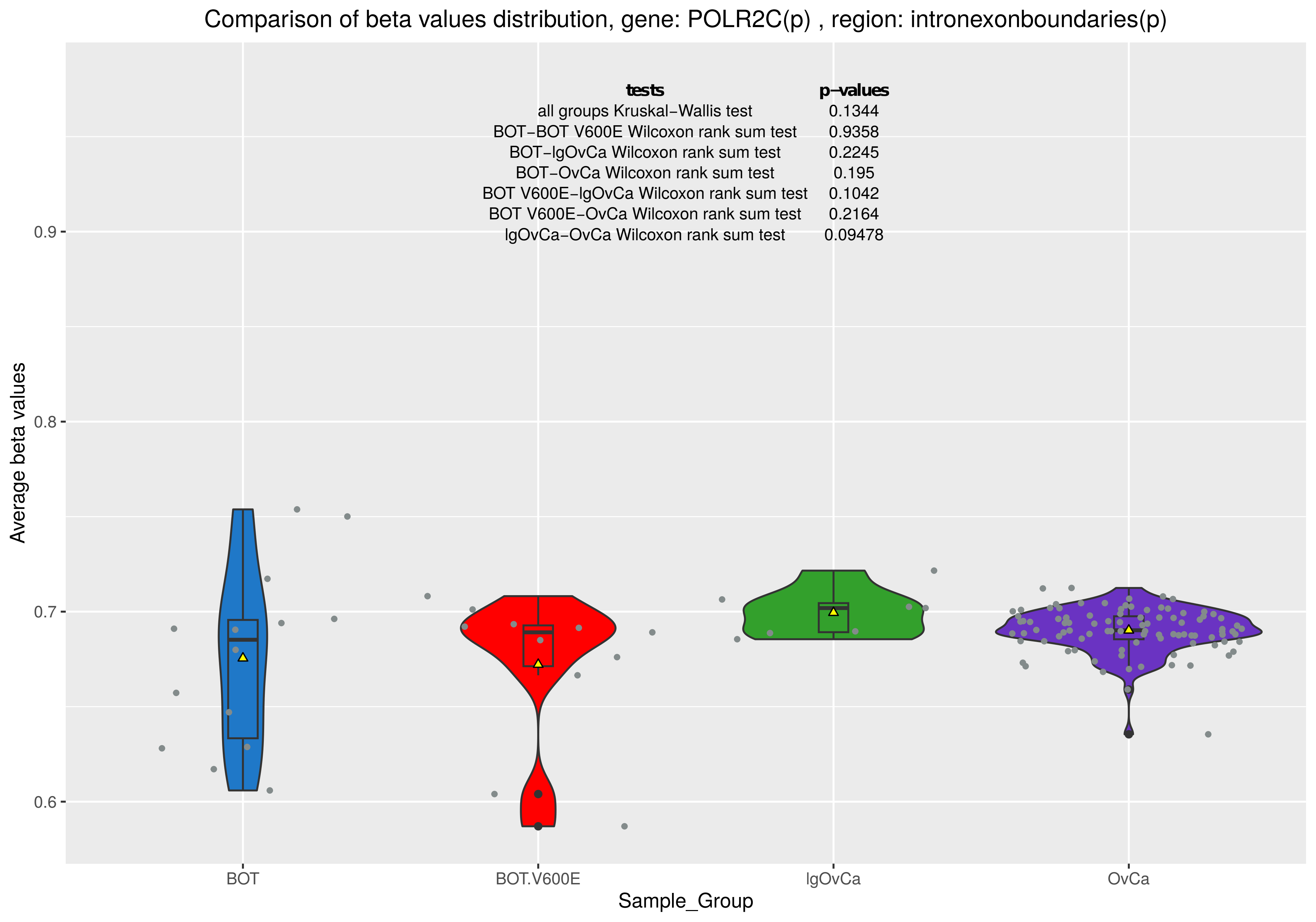
Comparison of beta values distribution, gene: POLR2C(p) , region: intronexonboundaries(p)
tests
p−values
all groups Kruskal−Wallis test
0.1344
BOT−BOT V600E Wilcoxon rank sum test
0.9358
BOT−lgOvCa Wilcoxon rank sum test
0.2245
BOT−OvCa Wilcoxon rank sum test
0.195
BOT V600E−lgOvCa Wilcoxon rank sum test
0.1042
BOT V600E−OvCa Wilcoxon rank sum test
0.2164
lgOvCa−OvCa Wilcoxon rank sum test
0.09478
Average beta values
0.9
0.8
0.7
0.6
BOT
BOT.V600E
lgOvCa
OvCa
Sample_Group

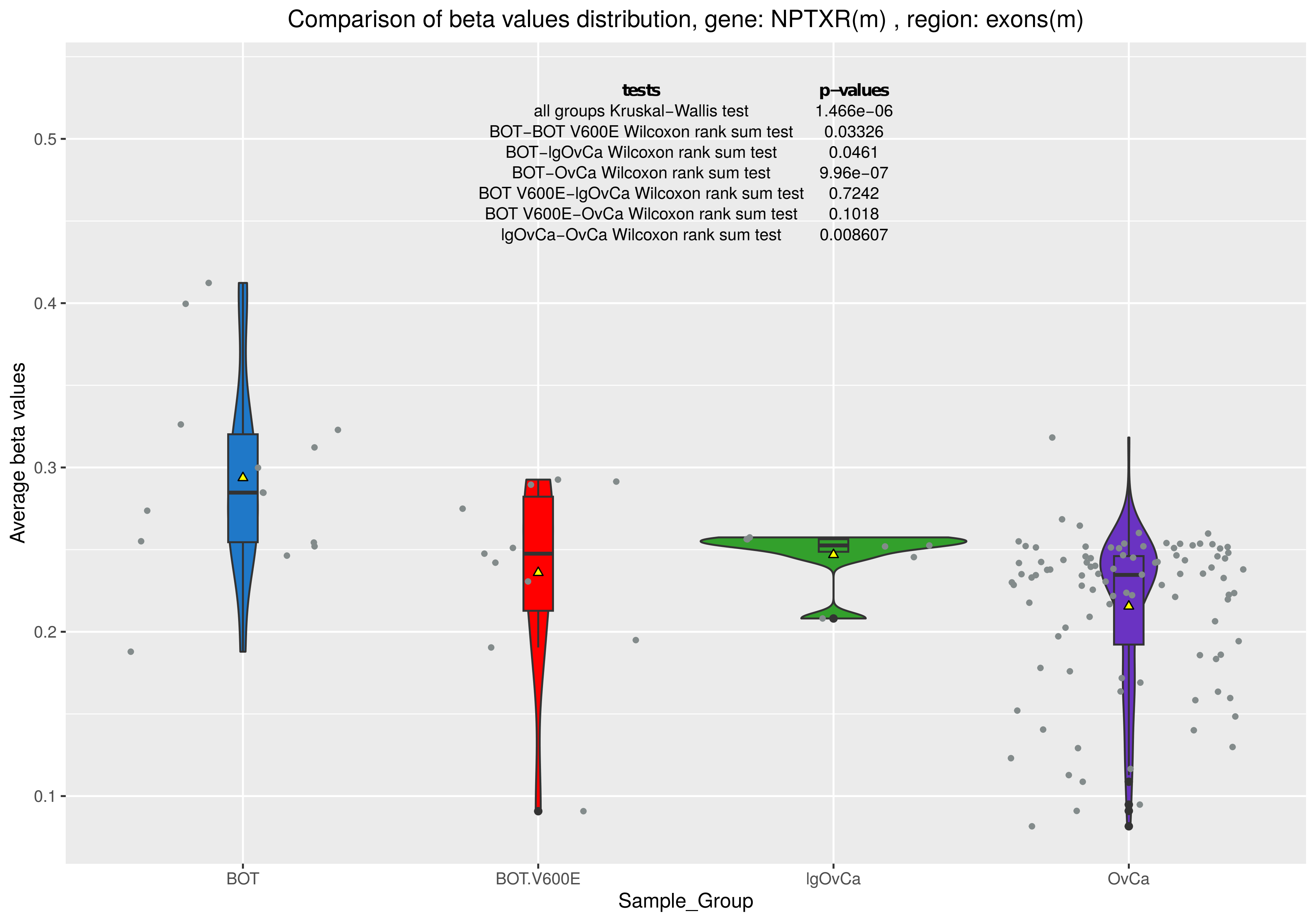
Comparison of beta values distribution, gene: NPTXR(m) , region: exons(m)
tests p−values
all groups Kruskal−Wallis test 1.466e−06
BOT−BOT V600E Wilcoxon rank sum test 0.03326
BOT−lgOvCa Wilcoxon rank sum test 0.0461
BOT−OvCa Wilcoxon rank sum test 9.96e−07
BOT V600E−lgOvCa Wilcoxon rank sum test 0.7242
BOT V600E−OvCa Wilcoxon rank sum test 0.1018
lgOvCa−OvCa Wilcoxon rank sum test 0.008607
Average beta values
0.5
0.4
0.3
0.2
0.1
BOT
BOT.V600E
lgOvCa
OvCa
Sample_Group

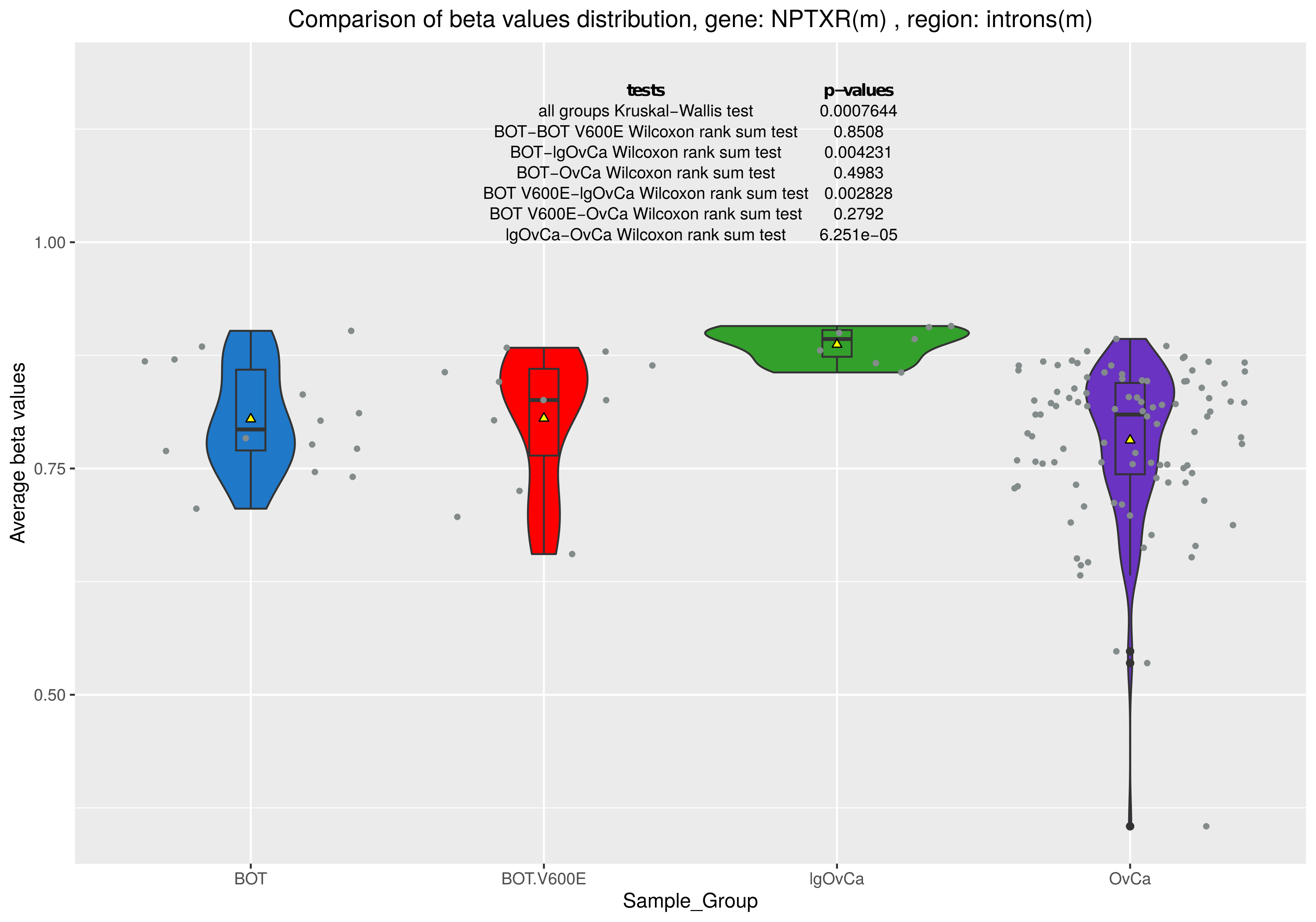
Comparison of beta values distribution, gene: NPTXR(m) , region: introns(m)
tests
p−values
all groups Kruskal−Wallis test
0.0007644
BOT−BOT V600E Wilcoxon rank sum test
0.8508
BOT−lgOvCa Wilcoxon rank sum test
0.004231
BOT−OvCa Wilcoxon rank sum test
0.4983
BOT V600E−lgOvCa Wilcoxon rank sum test
0.002828
BOT V600E−OvCa Wilcoxon rank sum test
0.2792
lgOvCa−OvCa Wilcoxon rank sum test
6.251e−05
Average beta values
1.00
0.75
0.50
BOT
BOT.V600E
lgOvCa
OvCa
Sample_Group

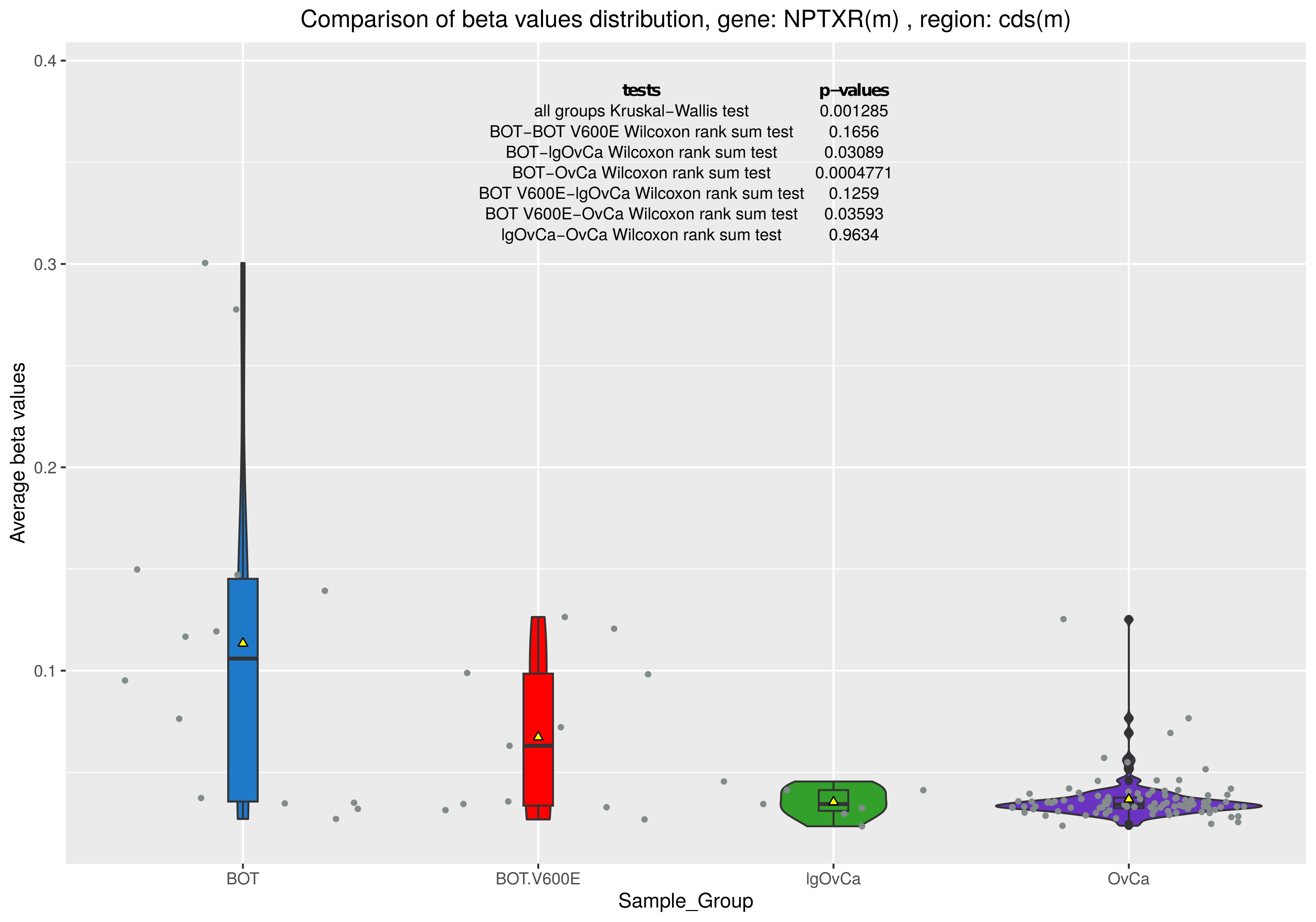

Comparison of beta values distribution, gene: NPTXR(m) , region: cds(m)
tests
p−values
all groups Kruskal−Wallis test
0.001285
BOT−BOT V600E Wilcoxon rank sum test
0.1656
BOT−lgOvCa Wilcoxon rank sum test
0.03089
BOT−OvCa Wilcoxon rank sum test
0.0004771
BOT V600E−lgOvCa Wilcoxon rank sum test
0.1259
BOT V600E−OvCa Wilcoxon rank sum test
0.03593
lgOvCa−OvCa Wilcoxon rank sum test
0.9634
Average beta values
0.4
0.3
0.2
0.1
BOT
BOT.V600E
lgOvCa
OvCa
Sample_Group

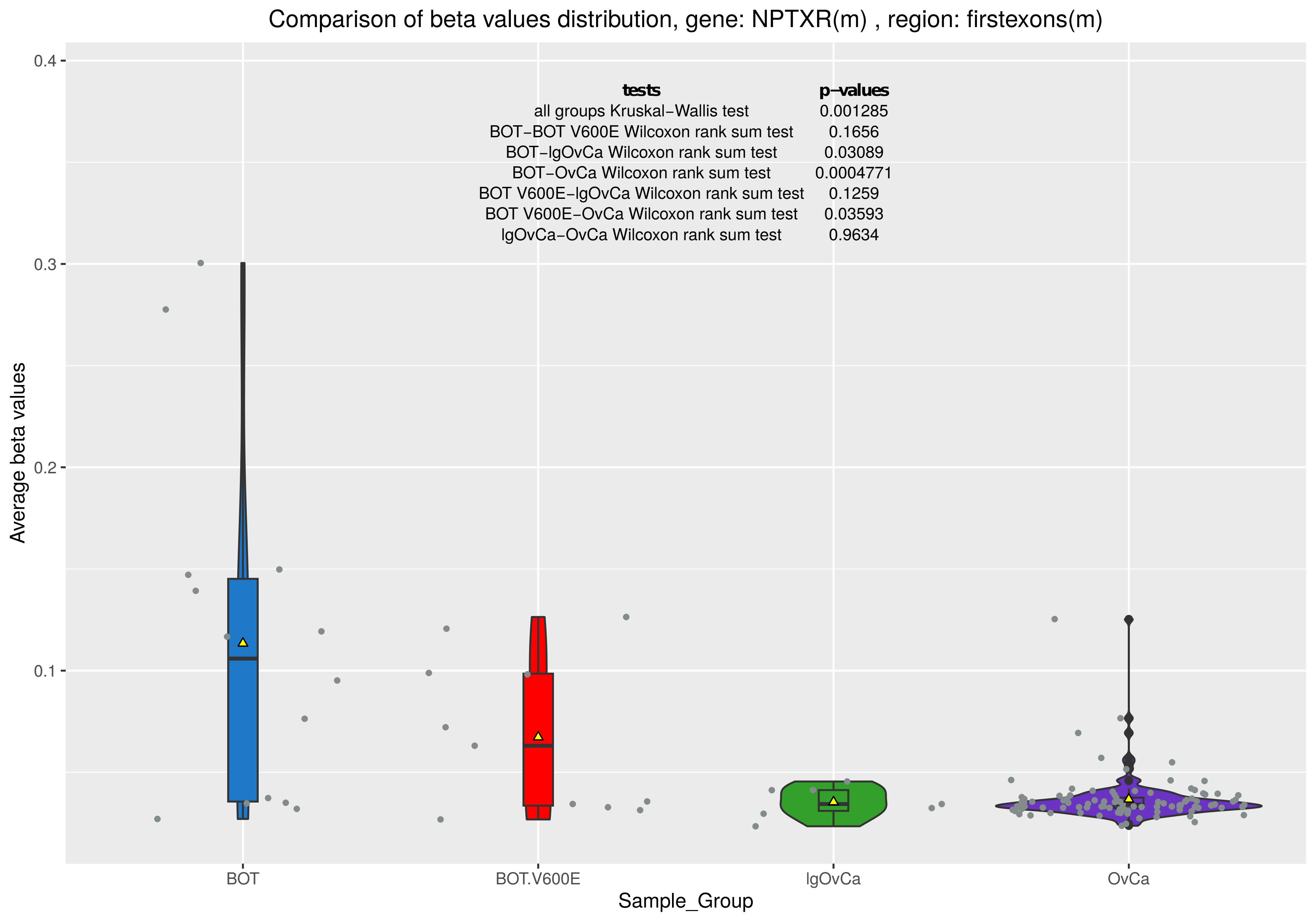
Comparison of beta values distribution, gene: NPTXR(m) , region: firstexons(m)
tests
p−values
all groups Kruskal−Wallis test
0.001285
BOT−BOT V600E Wilcoxon rank sum test
0.1656
BOT−IgOvCa Wilcoxon rank sum test
0.03089
BOT−OvCa Wilcoxon rank sum test
0.0004771
BOT V600E−IgOvCa Wilcoxon rank sum test
0.1259
BOT V600E−OvCa Wilcoxon rank sum test
0.03593
IgOvCa−OvCa Wilcoxon rank sum test
0.9634
Average beta values
0.4
0.3
0.2
0.1
BOT
BOT.V600E
IgOvCa
OvCa
Sample_Group

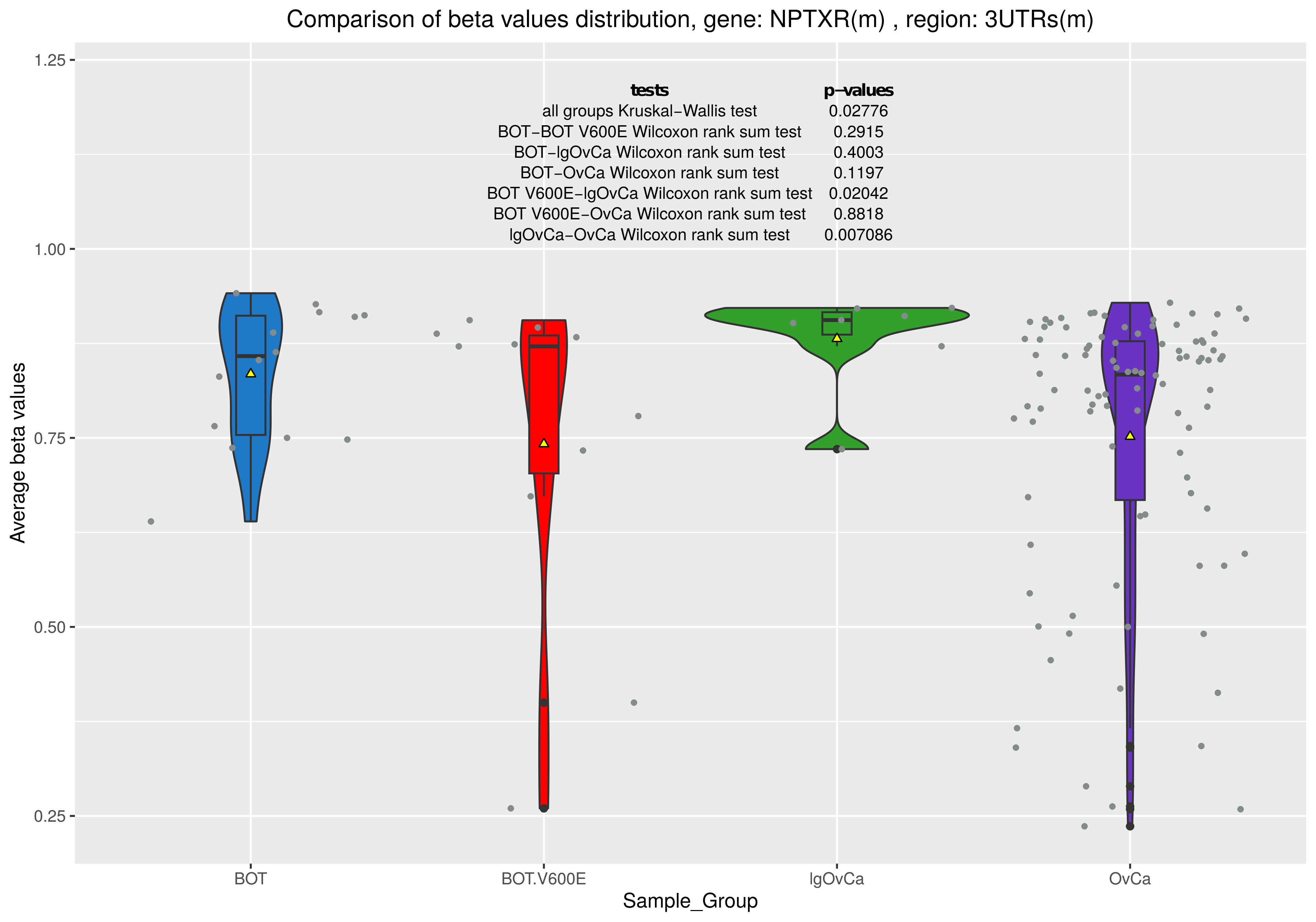

Comparison of beta values distribution, gene: NPTXR(m) , region: 3UTRs(m)
tests
p−values
all groups Kruskal−Wallis test 0.02776
BOT−BOT V600E Wilcoxon rank sum test 0.2915
BOT−lgOvCa Wilcoxon rank sum test 0.4003
BOT−OvCa Wilcoxon rank sum test 0.1197
BOT V600E−lgOvCa Wilcoxon rank sum test 0.02042
BOT V600E−OvCa Wilcoxon rank sum test 0.8818
lgOvCa−OvCa Wilcoxon rank sum test 0.007086
Average beta values
1.25
1.00
0.75
0.50
0.25
BOT
BOT.V600E
lgOvCa
OvCa
Sample_Group

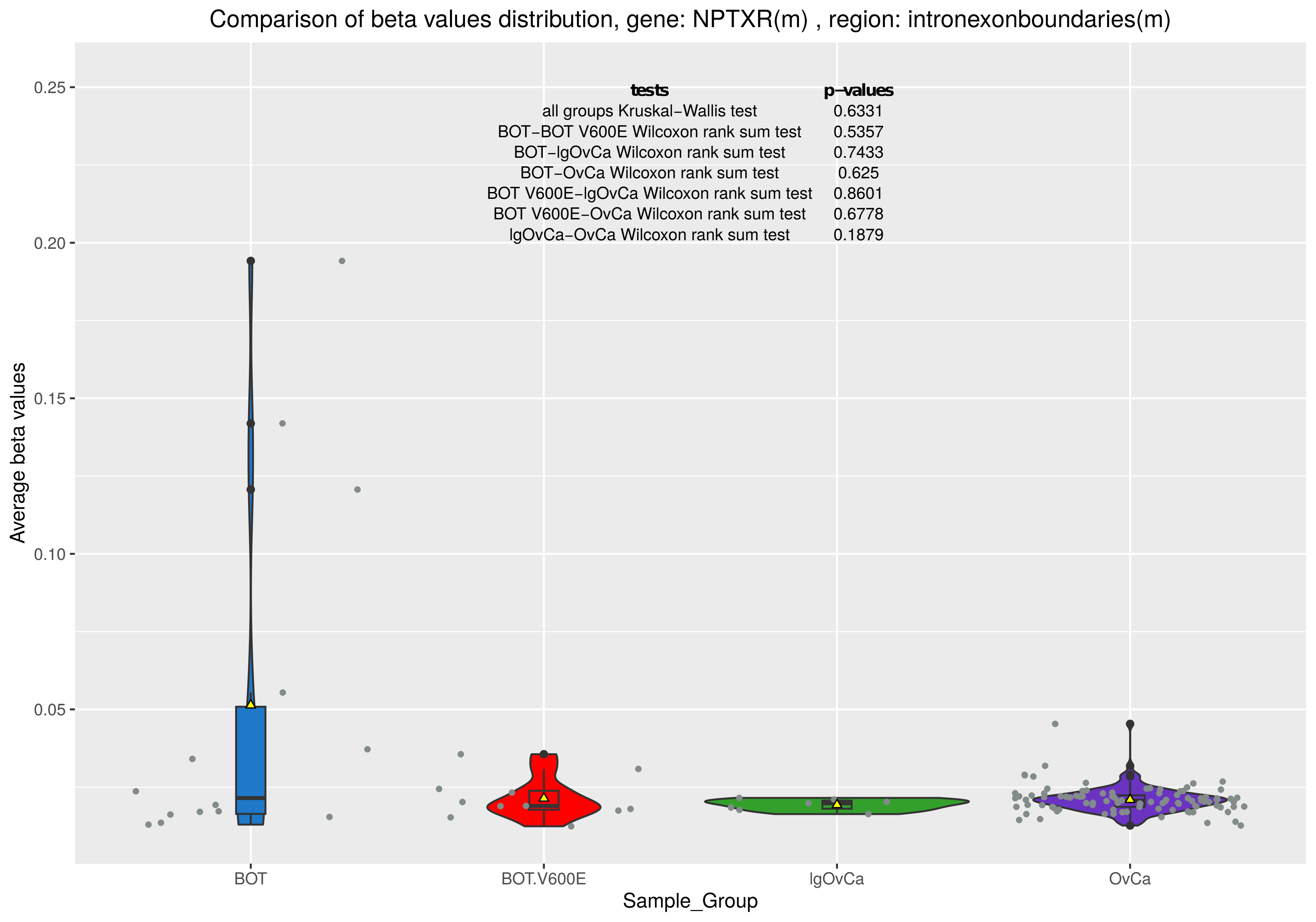

Comparison of beta values distribution, gene: NPTXR(m) , region: intronexonboundaries(m)
tests
p−values
all groups Kruskal−Wallis test 0.6331
BOT−BOT V600E Wilcoxon rank sum test 0.5357
BOT−lgOvCa Wilcoxon rank sum test 0.7433
BOT−OvCa Wilcoxon rank sum test 0.625
BOT V600E−lgOvCa Wilcoxon rank sum test 0.8601
BOT V600E−OvCa Wilcoxon rank sum test 0.6778
lgOvCa−OvCa Wilcoxon rank sum test 0.1879
Average beta values
0.25
0.20
0.15
0.10
0.05
BOT
BOT.V600E
lgOvCa
OvCa
Sample_Group

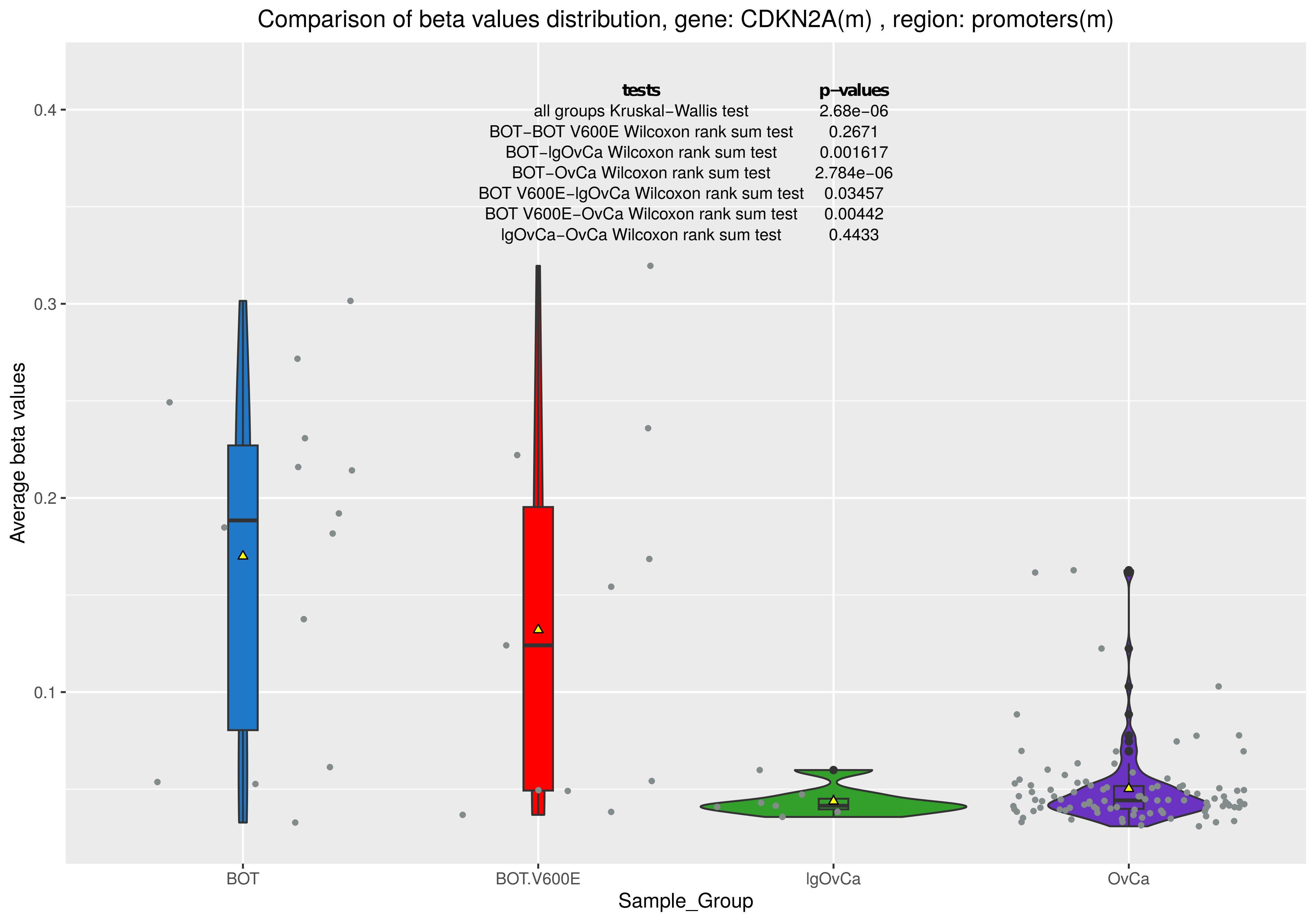

Comparison of beta values distribution, gene: CDKN2A(m) , region: promoters(m)
tests
p−values
all groups Kruskal−Wallis test
2.68e−06
BOT−BOT V600E Wilcoxon rank sum test
0.2671
BOT−lgOvCa Wilcoxon rank sum test
0.001617
BOT−OvCa Wilcoxon rank sum test
2.784e−06
BOT V600E−lgOvCa Wilcoxon rank sum test
0.03457
BOT V600E−OvCa Wilcoxon rank sum test
0.00442
lgOvCa−OvCa Wilcoxon rank sum test
0.4433
Average beta values
0.4
0.3
0.2
0.1
BOT
BOT.V600E
lgOvCa
OvCa
Sample_Group

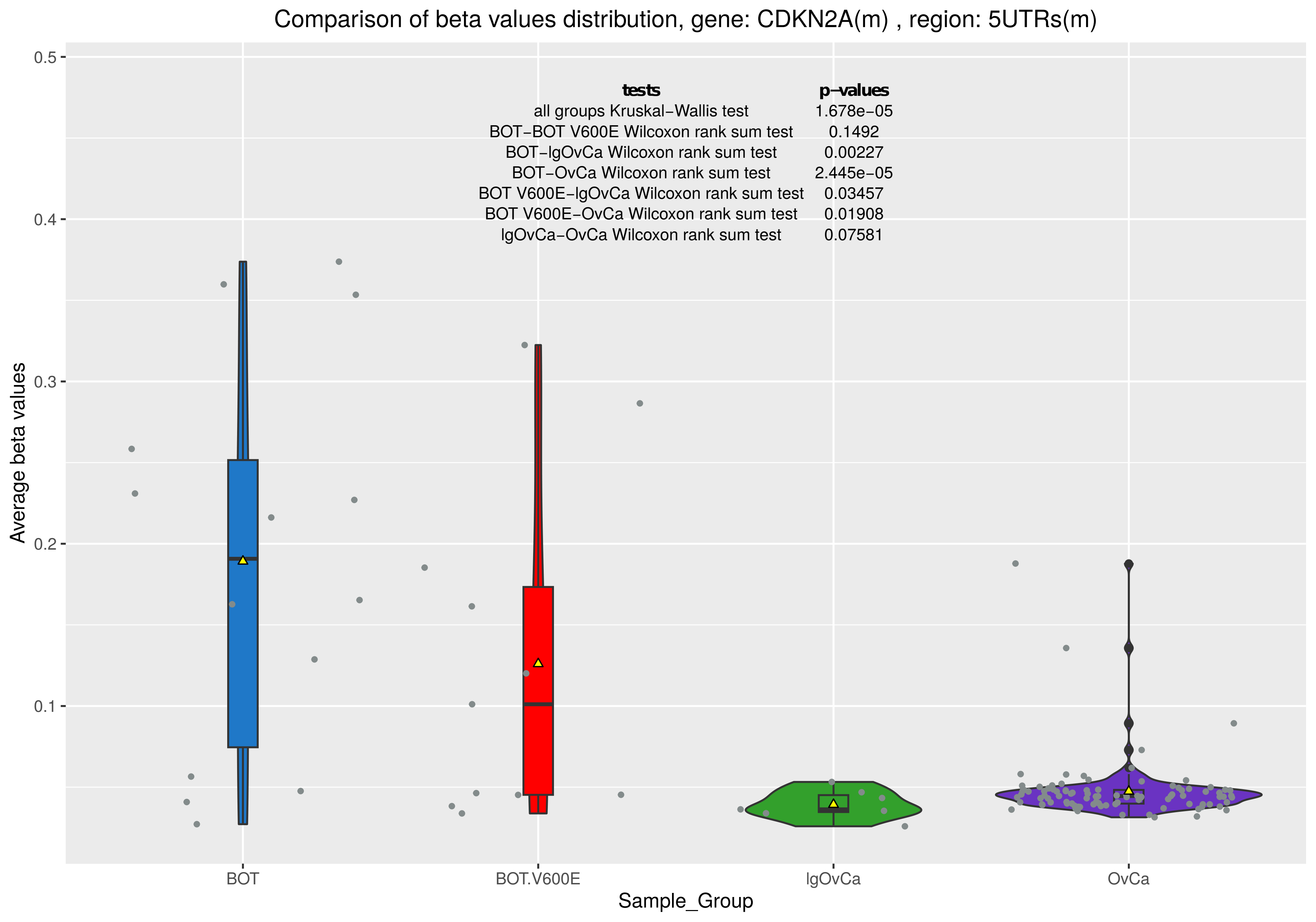
Comparison of beta values distribution, gene: CDKN2A(m) , region: 5UTRs(m)
tests
p−values
all groups Kruskal−Wallis test 1.678e−05
BOT−BOT V600E Wilcoxon rank sum test 0.1492
BOT−lgOvCa Wilcoxon rank sum test 0.00227
BOT−OvCa Wilcoxon rank sum test 2.445e−05
BOT V600E−lgOvCa Wilcoxon rank sum test 0.03457
BOT V600E−OvCa Wilcoxon rank sum test 0.01908
lgOvCa−OvCa Wilcoxon rank sum test 0.07581
Average beta values
0.5
0.4
0.3
0.2
0.1
BOT
BOT.V600E
lgOvCa
OvCa
Sample_Group

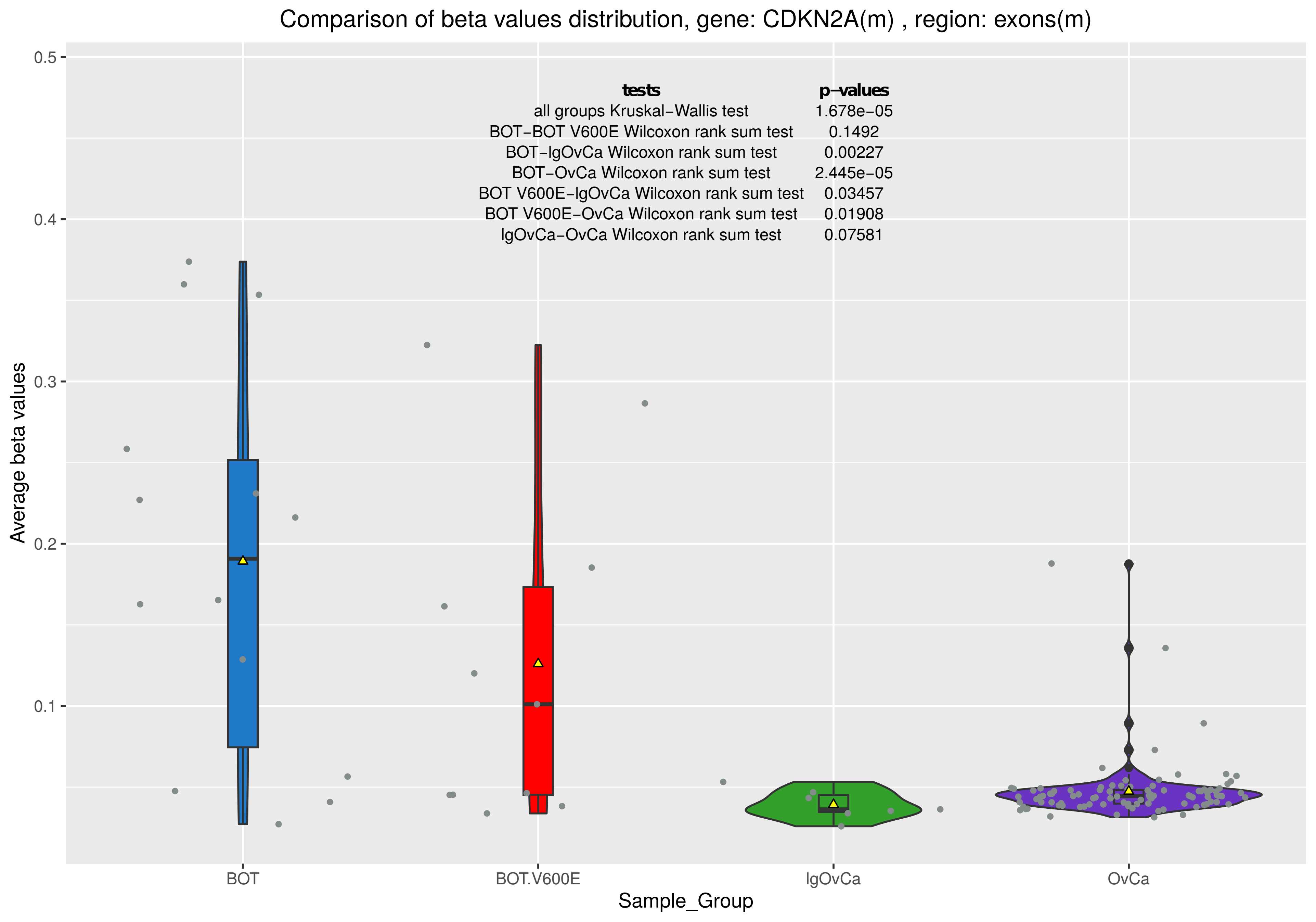

Comparison of beta values distribution, gene: CDKN2A(m) , region: exons(m)
tests
p−values
all groups Kruskal−Wallis test 1.678e−05
BOT−BOT V600E Wilcoxon rank sum test 0.1492
BOT−lgOvCa Wilcoxon rank sum test 0.00227
BOT−OvCa Wilcoxon rank sum test 2.445e−05
BOT V600E−lgOvCa Wilcoxon rank sum test 0.03457
BOT V600E−OvCa Wilcoxon rank sum test 0.01908
lgOvCa−OvCa Wilcoxon rank sum test 0.07581
Average beta values
0.5
0.4
0.3
0.2
0.1
BOT
BOT.V600E
lgOvCa
OvCa
Sample_Group

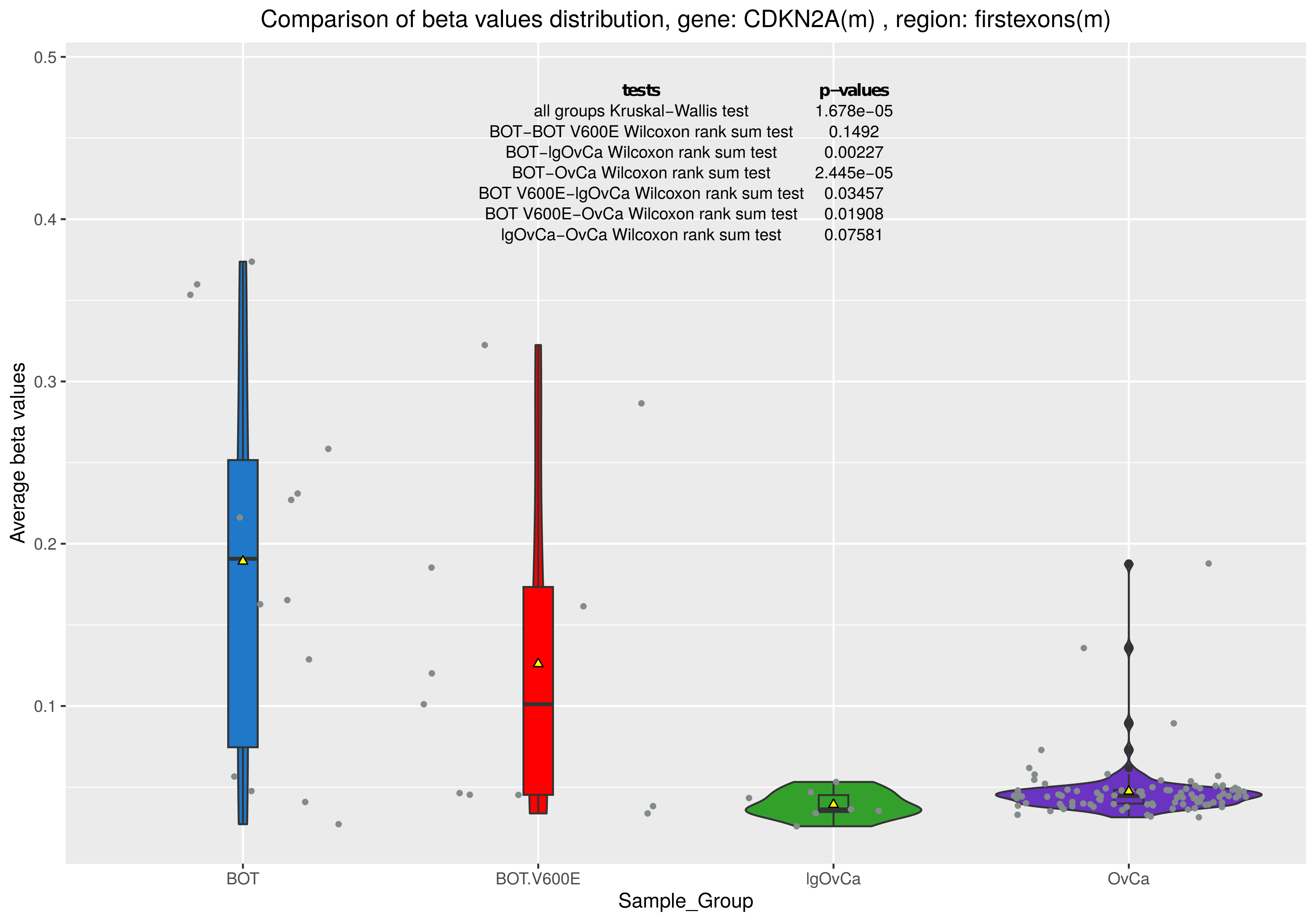
Comparison of beta values distribution, gene: CDKN2A(m) , region: firstexons(m)
tests
p−values
all groups Kruskal−Wallis test
1.678e−05
BOT−BOT V600E Wilcoxon rank sum test
0.1492
BOT−IgOvCa Wilcoxon rank sum test
0.00227
BOT−OvCa Wilcoxon rank sum test
2.445e−05
BOT V600E−IgOvCa Wilcoxon rank sum test
0.03457
BOT V600E−OvCa Wilcoxon rank sum test
0.01908
IgOvCa−OvCa Wilcoxon rank sum test
0.07581
Average beta values
0.5
0.4
0.3
0.2
0.1
BOT
BOT.V600E
IgOvCa
OvCa
Sample_Group

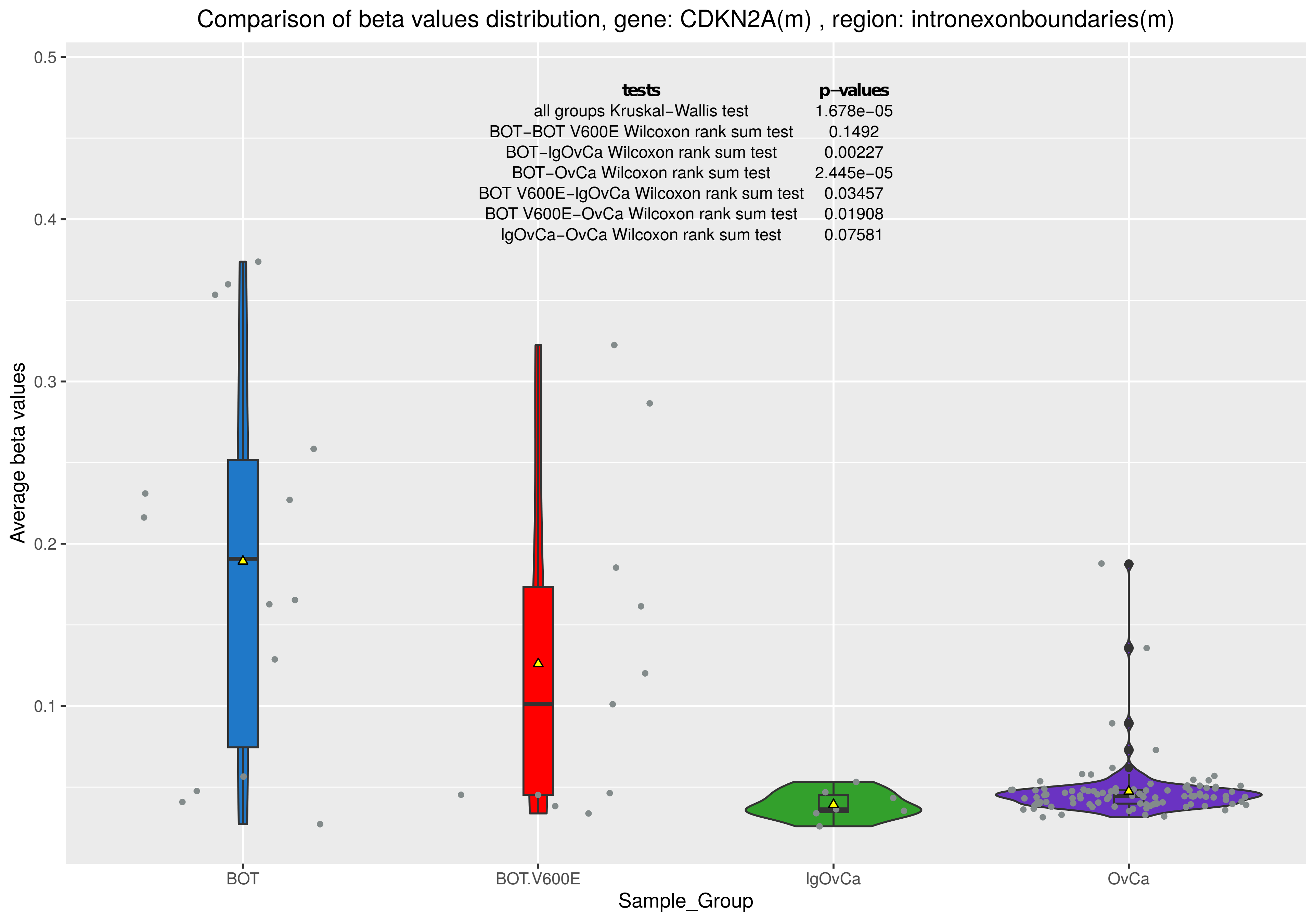
Comparison of beta values distribution, gene: CDKN2A(m) , region: intronexonboundaries(m)
tests
p−values
all groups Kruskal−Wallis test
1.678e−05
BOT−BOT V600E Wilcoxon rank sum test
0.1492
BOT−lgOvCa Wilcoxon rank sum test
0.00227
BOT−OvCa Wilcoxon rank sum test
2.445e−05
BOT V600E−lgOvCa Wilcoxon rank sum test
0.03457
BOT V600E−OvCa Wilcoxon rank sum test
0.01908
lgOvCa−OvCa Wilcoxon rank sum test
0.07581
Average beta values
0.5
0.4
0.3
0.2
0.1
BOT
BOT.V600E
lgOvCa
OvCa
Sample_Group

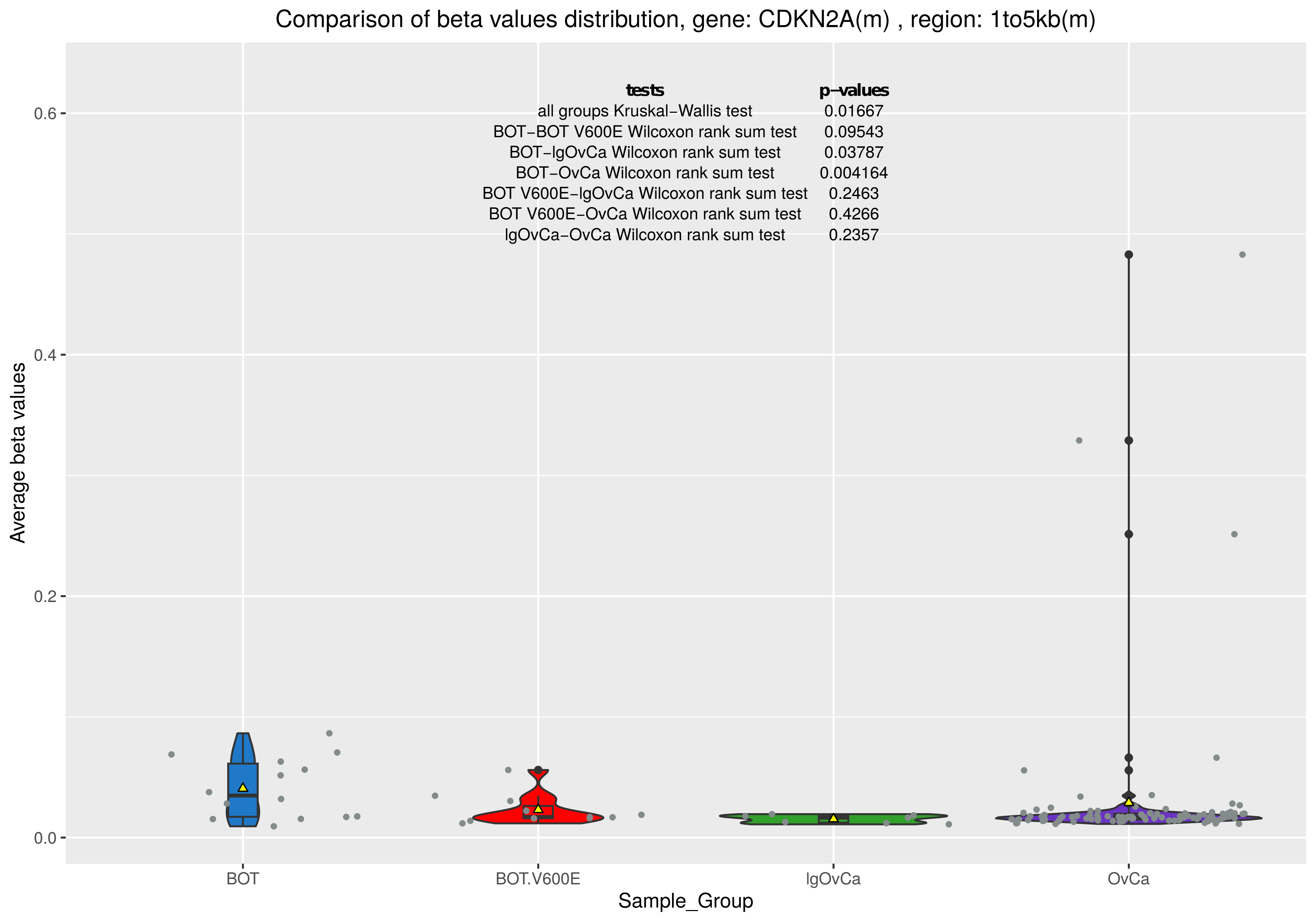

Comparison of beta values distribution, gene: CDKN2A(m) , region: 1to5kb(m)
tests
p−values
all groups Kruskal−Wallis test
0.01667
BOT−BOT V600E Wilcoxon rank sum test
0.09543
BOT−lgOvCa Wilcoxon rank sum test
0.03787
BOT−OvCa Wilcoxon rank sum test
0.004164
BOT V600E−lgOvCa Wilcoxon rank sum test
0.2463
BOT V600E−OvCa Wilcoxon rank sum test
0.4266
lgOvCa−OvCa Wilcoxon rank sum test
0.2357
Average beta values
0.6
0.4
0.2
0.0
BOT
BOT.V600E
lgOvCa
OvCa
Sample_Group

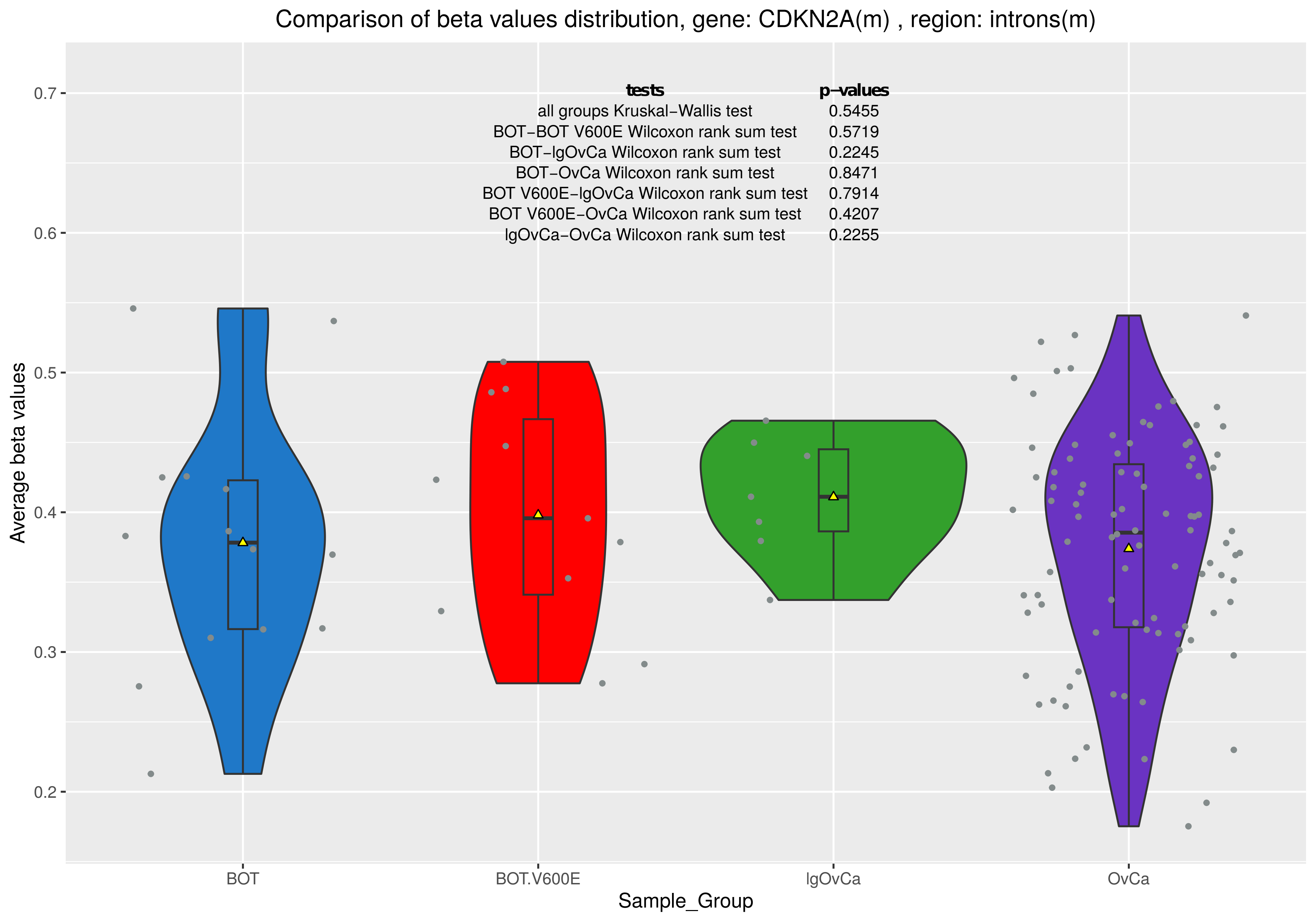
Comparison of beta values distribution, gene: CDKN2A(m) , region: introns(m)
tests
p−values
all groups Kruskal−Wallis test 0.5455
BOT−BOT V600E Wilcoxon rank sum test 0.5719
BOT−lgOvCa Wilcoxon rank sum test 0.2245
BOT−OvCa Wilcoxon rank sum test 0.8471
BOT V600E−lgOvCa Wilcoxon rank sum test 0.7914
BOT V600E−OvCa Wilcoxon rank sum test 0.4207
lgOvCa−OvCa Wilcoxon rank sum test 0.2255
Average beta values
0.7
0.6
0.5
0.4
0.3
0.2
BOT
BOT.V600E
lgOvCa
OvCa
Sample_Group

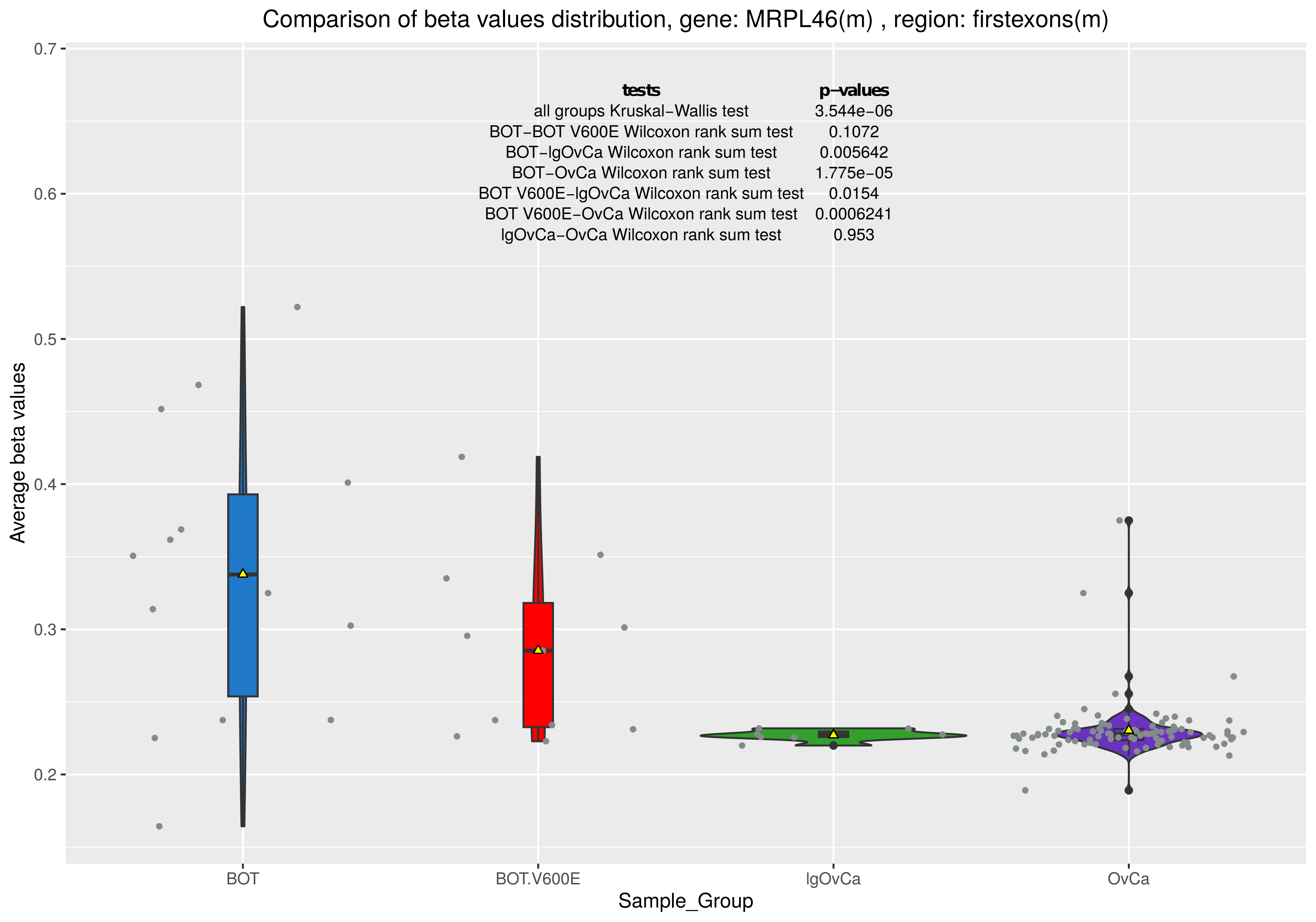

Comparison of beta values distribution, gene: MRPL46(m) , region: firstexons(m)
tests
p−values
all groups Kruskal−Wallis test
3.544e−06
BOT−BOT V600E Wilcoxon rank sum test
0.1072
BOT−lgOvCa Wilcoxon rank sum test
0.005642
BOT−OvCa Wilcoxon rank sum test
1.775e−05
BOT V600E−lgOvCa Wilcoxon rank sum test
0.0154
BOT V600E−OvCa Wilcoxon rank sum test
0.0006241
lgOvCa−OvCa Wilcoxon rank sum test
0.953
Average beta values
0.7
0.6
0.5
0.4
0.3
0.2
BOT
BOT.V600E
lgOvCa
OvCa
Sample_Group

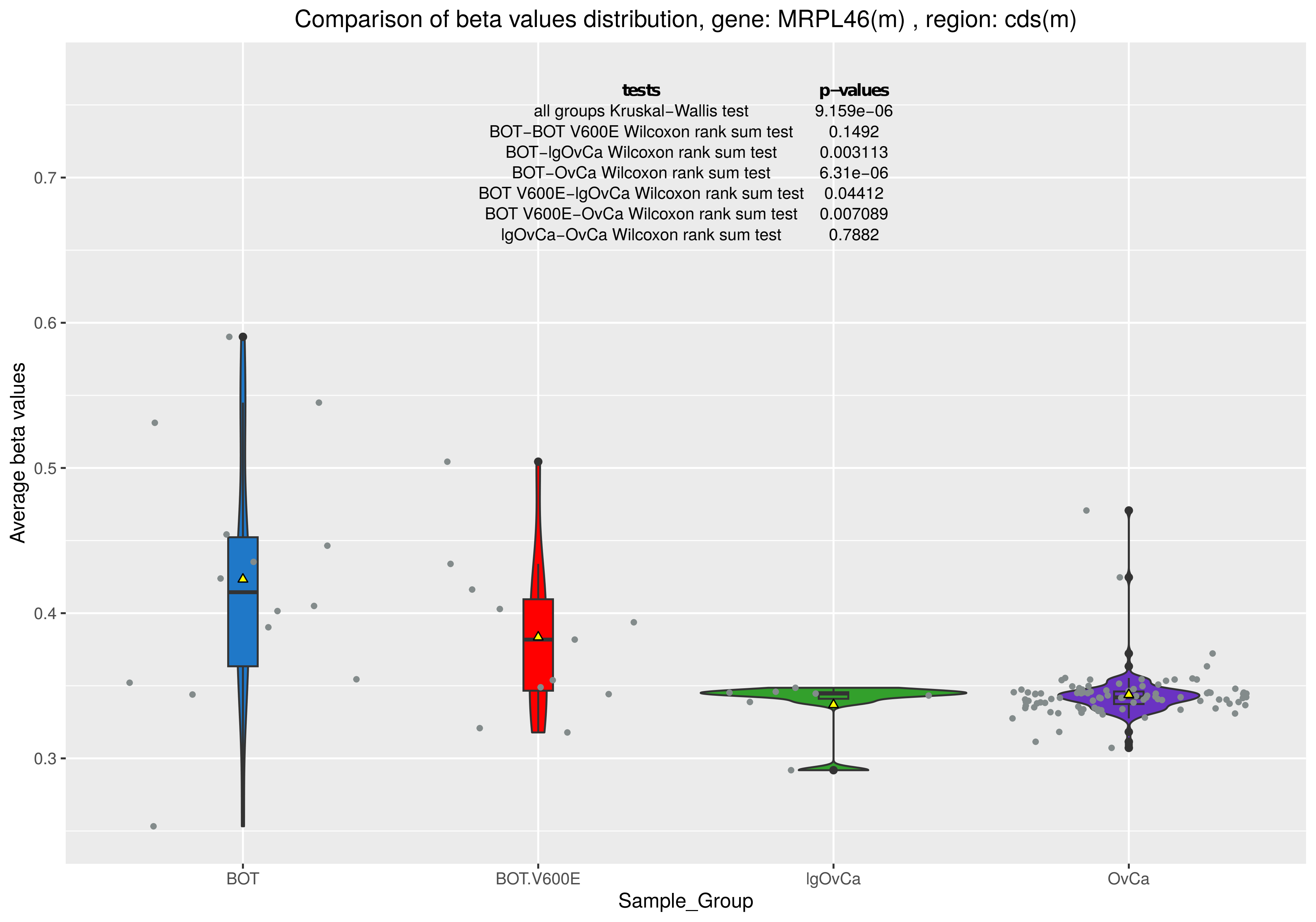

Comparison of beta values distribution, gene: MRPL46(m) , region: cds(m)
tests
p−values
all groups Kruskal−Wallis test
9.159e−06
BOT−BOT V600E Wilcoxon rank sum test
0.1492
BOT−lgOvCa Wilcoxon rank sum test
0.003113
BOT−OvCa Wilcoxon rank sum test
6.31e−06
BOT V600E−lgOvCa Wilcoxon rank sum test
0.04412
BOT V600E−OvCa Wilcoxon rank sum test
0.007089
lgOvCa−OvCa Wilcoxon rank sum test
0.7882
Average beta values
0.7
0.6
0.5
0.4
0.3
BOT
BOT.V600E
lgOvCa
OvCa
Sample_Group

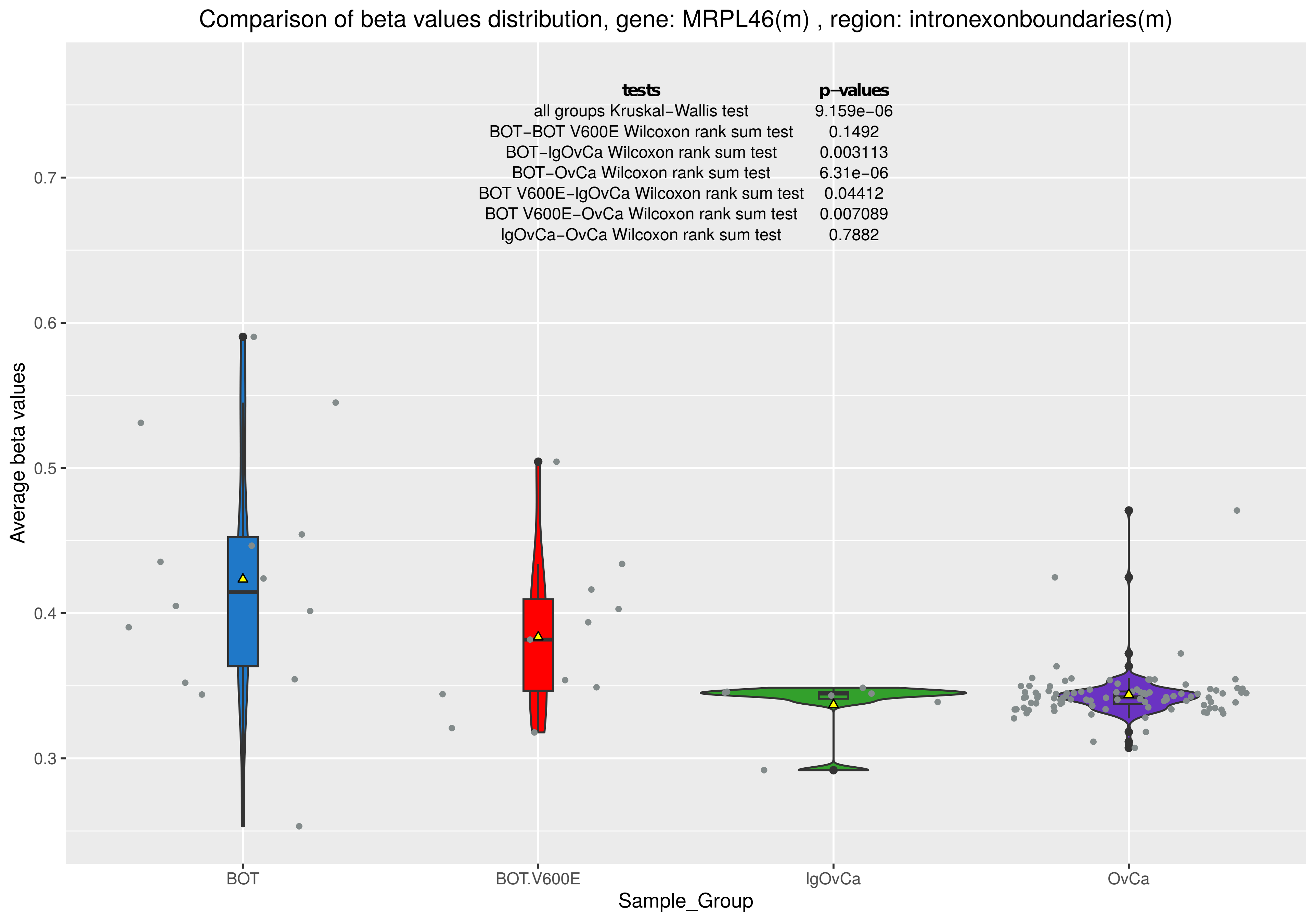

Comparison of beta values distribution, gene: MRPL46(m) , region: intronexonboundaries(m)
tests
p−values
all groups Kruskal−Wallis test
9.159e−06
BOT−BOT V600E Wilcoxon rank sum test
0.1492
BOT−lgOvCa Wilcoxon rank sum test
0.003113
BOT−OvCa Wilcoxon rank sum test
6.31e−06
BOT V600E−lgOvCa Wilcoxon rank sum test
0.04412
BOT V600E−OvCa Wilcoxon rank sum test
0.007089
lgOvCa−OvCa Wilcoxon rank sum test
0.7882
Average beta values
0.7
0.6
0.5
0.4
0.3
BOT
BOT.V600E
lgOvCa
OvCa
Sample_Group

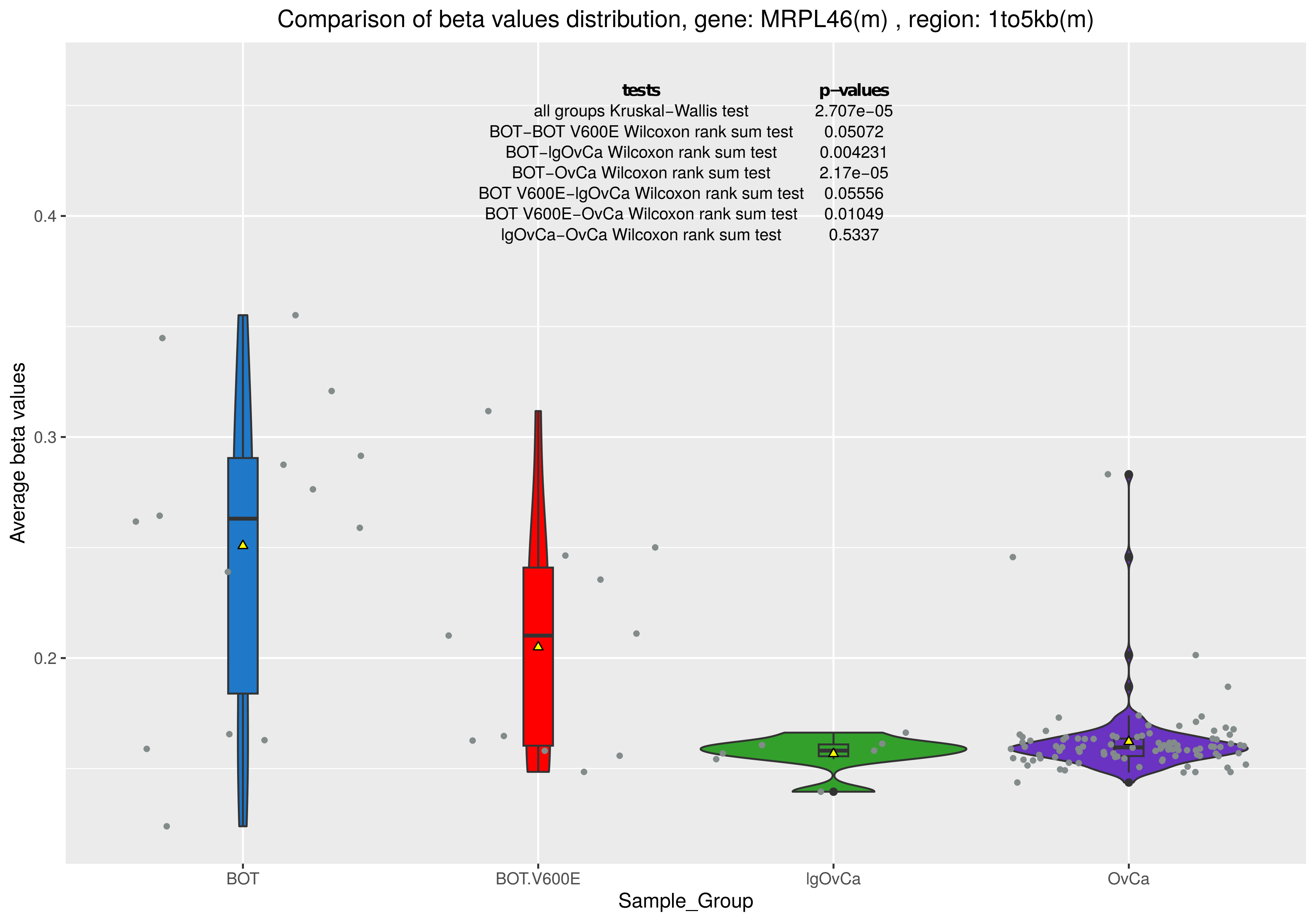

Comparison of beta values distribution, gene: MRPL46(m) , region: 1to5kb(m)
tests
p−values
all groups Kruskal−Wallis test
2.707e−05
BOT−BOT V600E Wilcoxon rank sum test
0.05072
BOT−lgOvCa Wilcoxon rank sum test
0.004231
BOT−OvCa Wilcoxon rank sum test
2.17e−05
BOT V600E−lgOvCa Wilcoxon rank sum test
0.05556
BOT V600E−OvCa Wilcoxon rank sum test
0.01049
lgOvCa−OvCa Wilcoxon rank sum test
0.5337
Average beta values
0.4
0.3
0.2
BOT
BOT.V600E
lgOvCa
OvCa
Sample_Group

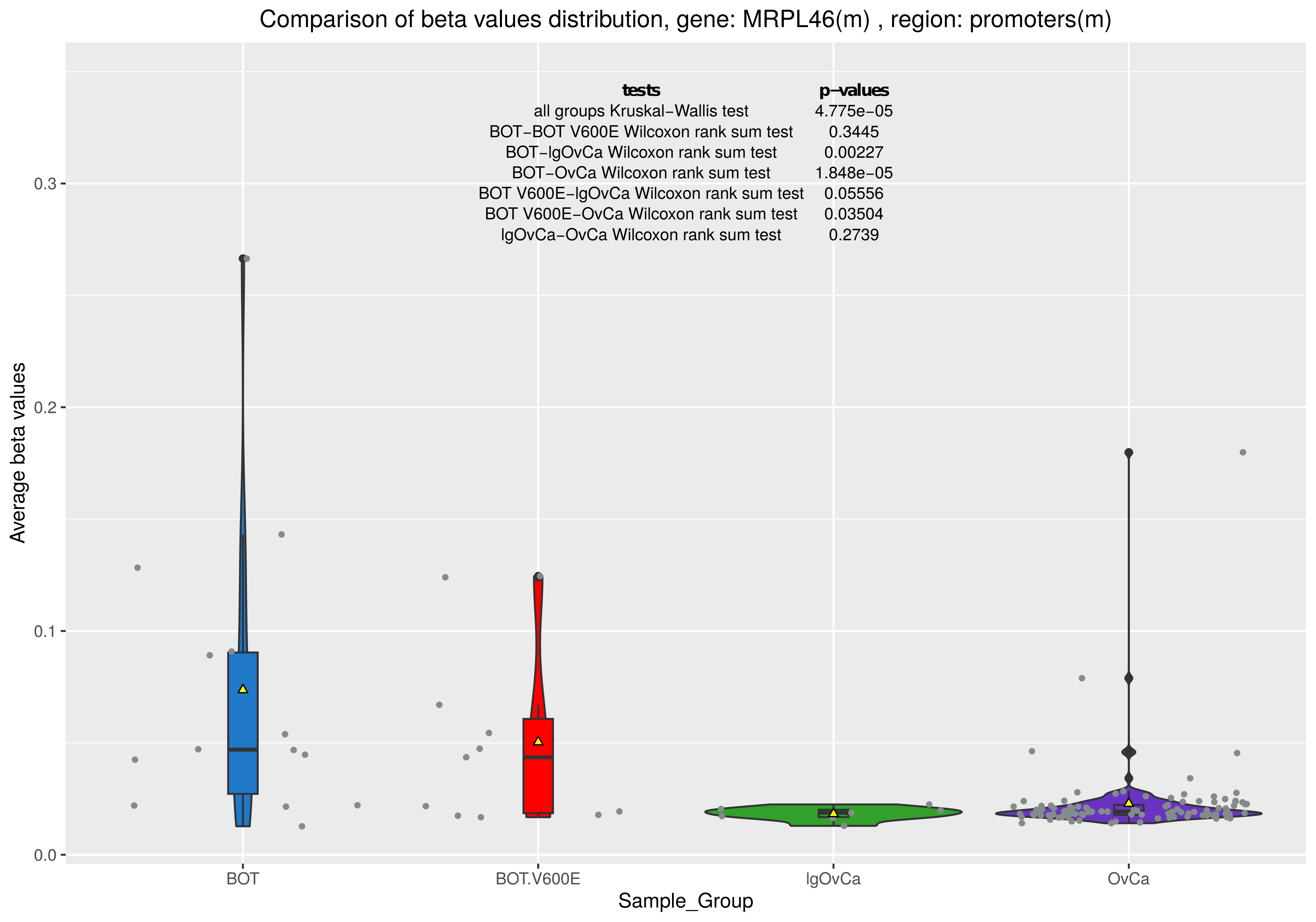

Comparison of beta values distribution, gene: MRPL46(m) , region: promoters(m)
tests
p−values
all groups Kruskal−Wallis test
4.775e−05
BOT−BOT V600E Wilcoxon rank sum test
0.3445
BOT−IgOvCa Wilcoxon rank sum test
0.00227
BOT−OvCa Wilcoxon rank sum test
1.848e−05
BOT V600E−IgOvCa Wilcoxon rank sum test
0.05556
BOT V600E−OvCa Wilcoxon rank sum test
0.03504
IgOvCa−OvCa Wilcoxon rank sum test
0.2739
Average beta values
0.0
0.1
0.2
0.3
BOT
BOT.V600E
IgOvCa
OvCa
Sample_Group

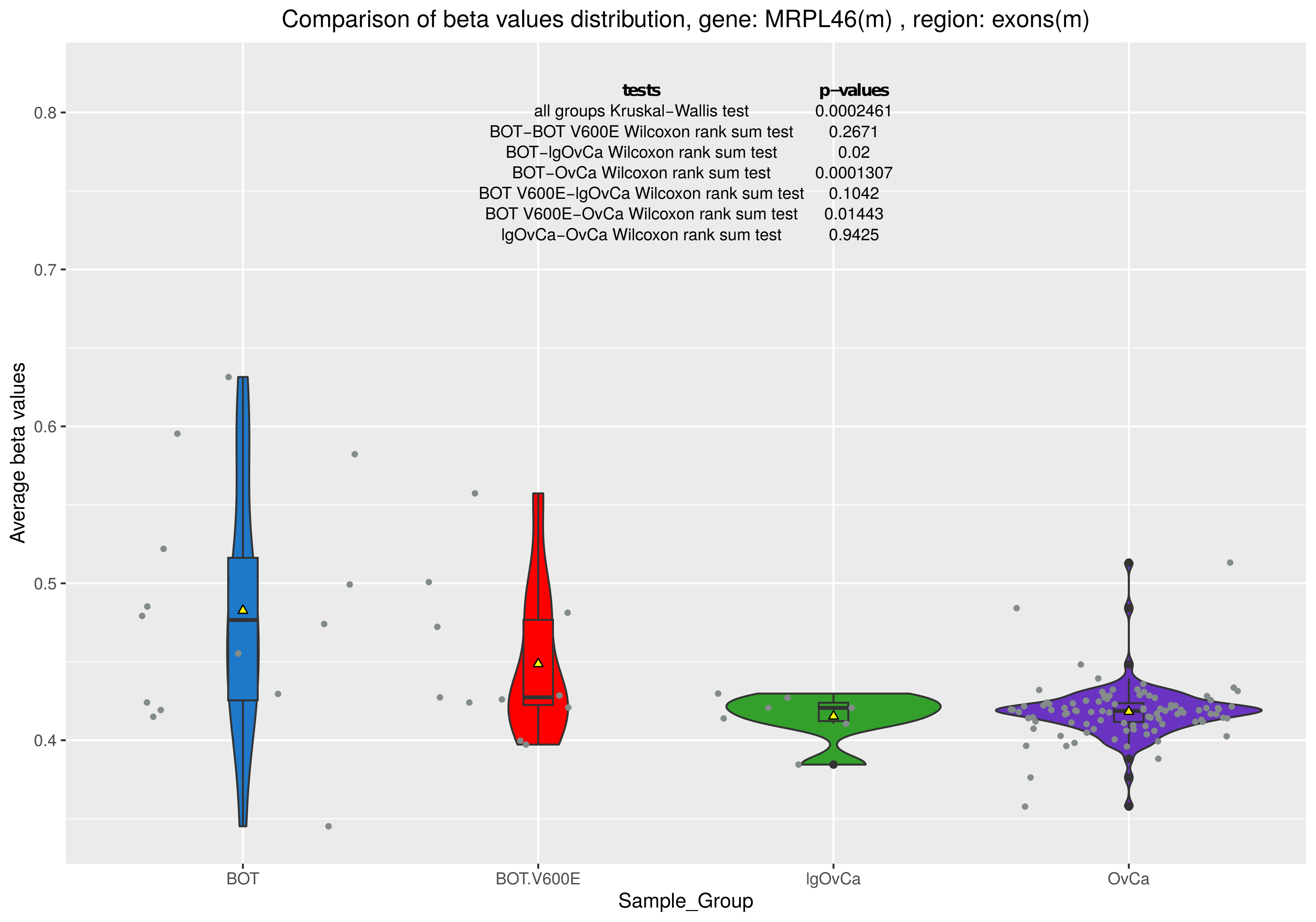

Comparison of beta values distribution, gene: MRPL46(m) , region: exons(m)
tests
p−values
all groups Kruskal−Wallis test
0.0002461
BOT−BOT V600E Wilcoxon rank sum test
0.2671
BOT−lgOvCa Wilcoxon rank sum test
0.02
BOT−OvCa Wilcoxon rank sum test
0.0001307
BOT V600E−lgOvCa Wilcoxon rank sum test
0.1042
BOT V600E−OvCa Wilcoxon rank sum test
0.01443
lgOvCa−OvCa Wilcoxon rank sum test
0.9425
Average beta values
0.8
0.7
0.6
0.5
0.4
BOT
BOT.V600E
lgOvCa
OvCa
Sample_Group

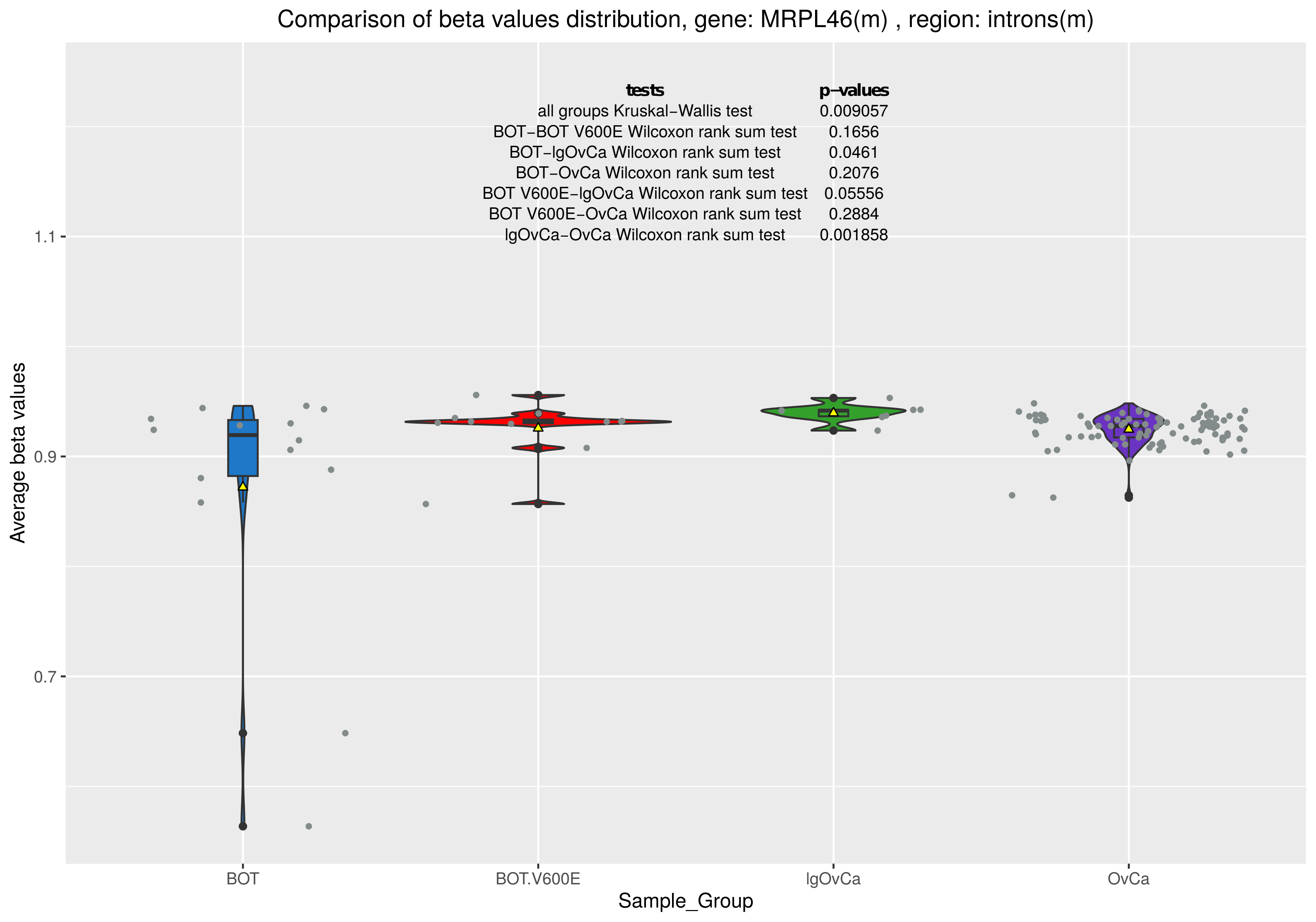

Comparison of beta values distribution, gene: MRPL46(m) , region: introns(m)
tests
p−values
all groups Kruskal−Wallis test
0.009057
BOT−BOT V600E Wilcoxon rank sum test
0.1656
BOT−lgOvCa Wilcoxon rank sum test
0.0461
BOT−OvCa Wilcoxon rank sum test
0.2076
BOT V600E−lgOvCa Wilcoxon rank sum test
0.05556
BOT V600E−OvCa Wilcoxon rank sum test
0.2884
lgOvCa−OvCa Wilcoxon rank sum test
0.001858
Average beta values
1.1
0.9
0.7
BOT
BOT.V600E
lgOvCa
OvCa
Sample_Group

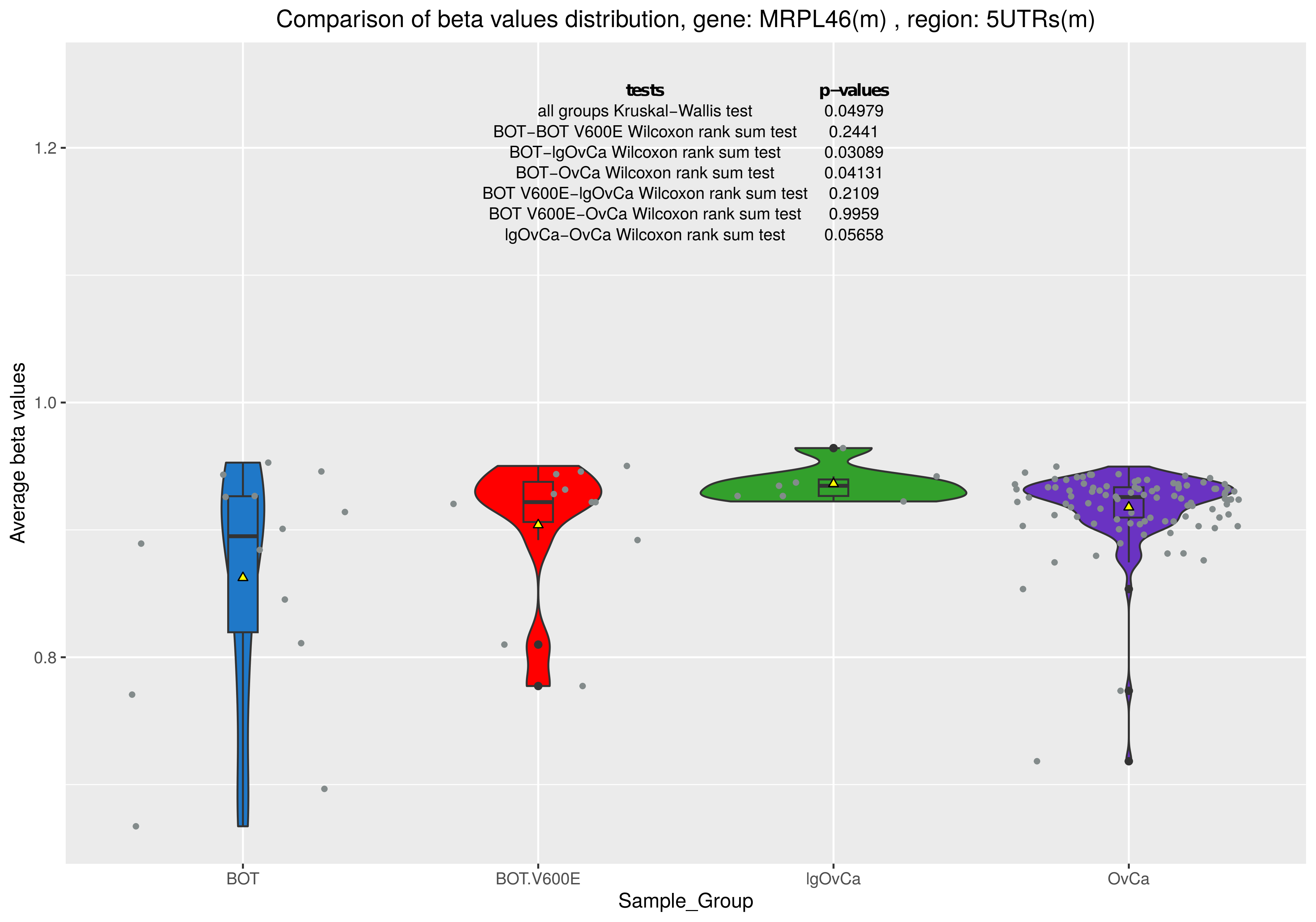
Comparison of beta values distribution, gene: MRPL46(m) , region: 5UTRs(m)
tests
p−values
all groups Kruskal−Wallis test
0.04979
BOT−BOT V600E Wilcoxon rank sum test
0.2441
BOT−lgOvCa Wilcoxon rank sum test
0.03089
BOT−OvCa Wilcoxon rank sum test
0.04131
BOT V600E−lgOvCa Wilcoxon rank sum test
0.2109
BOT V600E−OvCa Wilcoxon rank sum test
0.9959
lgOvCa−OvCa Wilcoxon rank sum test
0.05658
Average beta values
1.2
1.0
0.8
BOT
BOT.V600E
lgOvCa
OvCa
Sample_Group

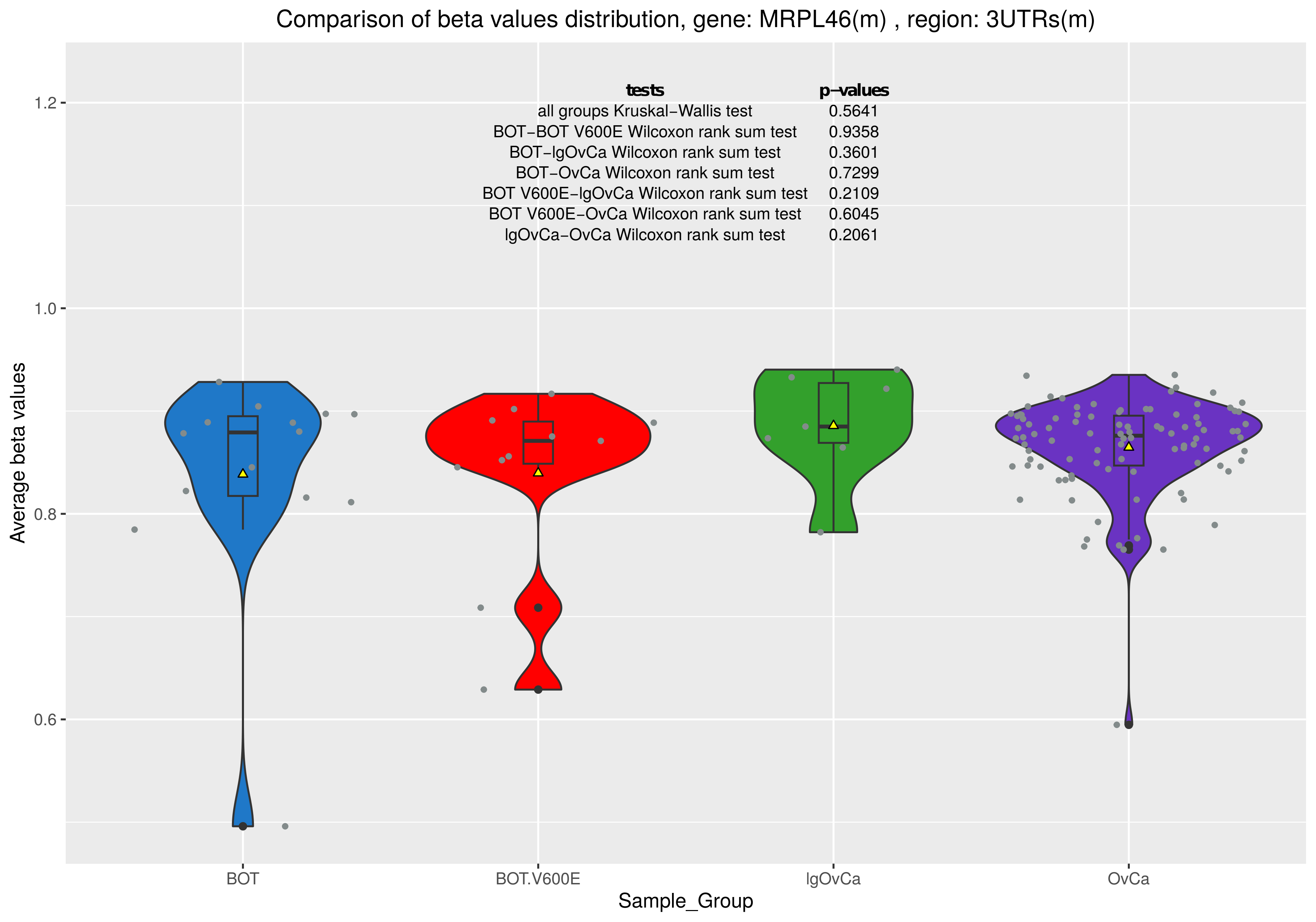

Comparison of beta values distribution, gene: MRPL46(m) , region: 3UTRs(m)
tests
p−values
all groups Kruskal−Wallis test
0.5641
BOT−BOT V600E Wilcoxon rank sum test
0.9358
BOT−lgOvCa Wilcoxon rank sum test
0.3601
BOT−OvCa Wilcoxon rank sum test
0.7299
BOT V600E−lgOvCa Wilcoxon rank sum test
0.2109
BOT V600E−OvCa Wilcoxon rank sum test
0.6045
lgOvCa−OvCa Wilcoxon rank sum test
0.2061
Average beta values
1.2
1.0
0.8
0.6
BOT
BOT.V600E
lgOvCa
OvCa
Sample_Group

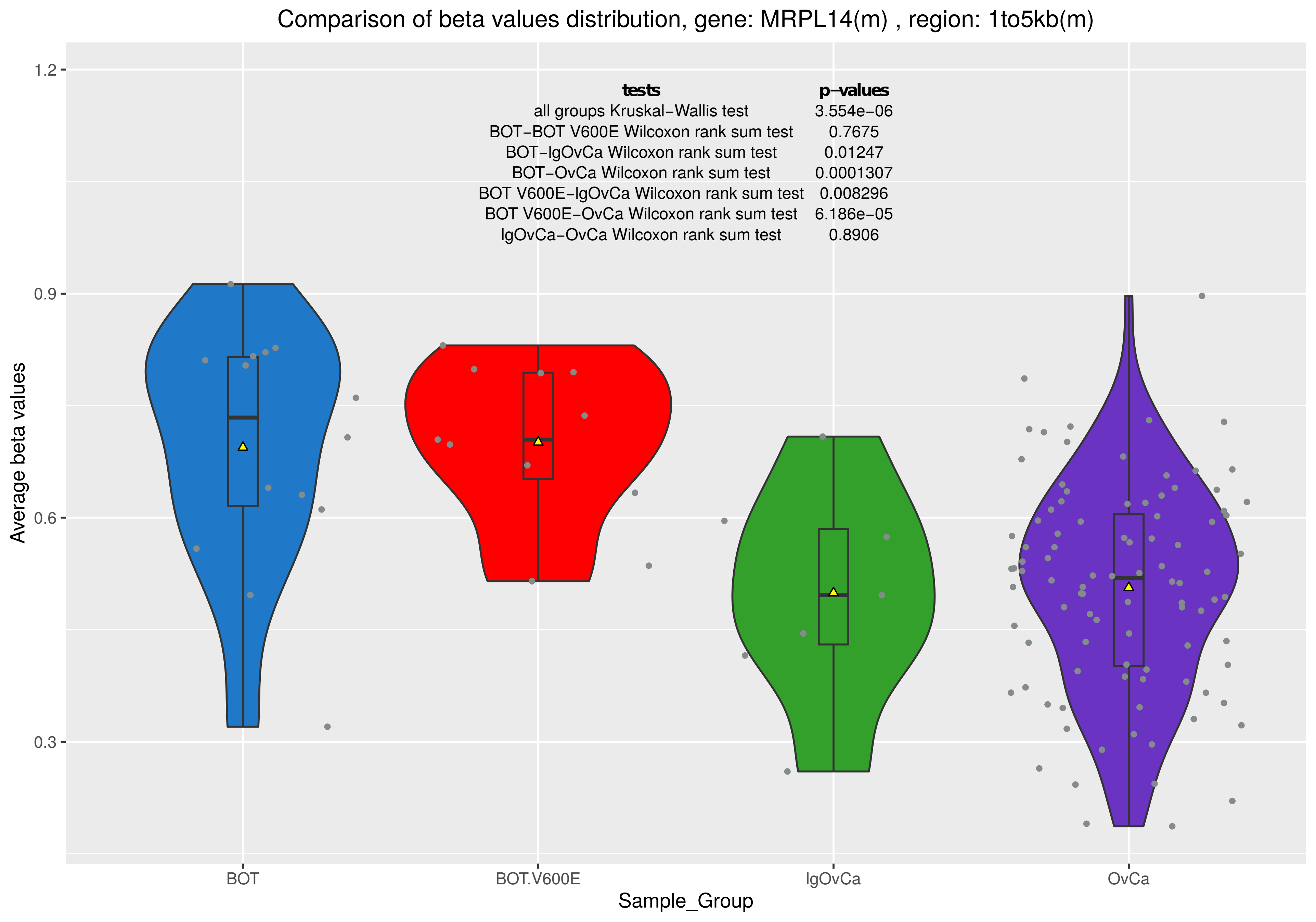
Comparison of beta values distribution, gene: MRPL14(m) , region: 1to5kb(m)
tests
p−values
all groups Kruskal−Wallis test
3.554e−06
BOT−BOT V600E Wilcoxon rank sum test
0.7675
BOT−IgOvCa Wilcoxon rank sum test
0.01247
BOT−OvCa Wilcoxon rank sum test
0.0001307
BOT V600E−IgOvCa Wilcoxon rank sum test
0.008296
BOT V600E−OvCa Wilcoxon rank sum test
6.186e−05
IgOvCa−OvCa Wilcoxon rank sum test
0.8906
Average beta values
1.2
0.9
0.6
0.3
BOT
BOT.V600E
IgOvCa
OvCa
Sample_Group

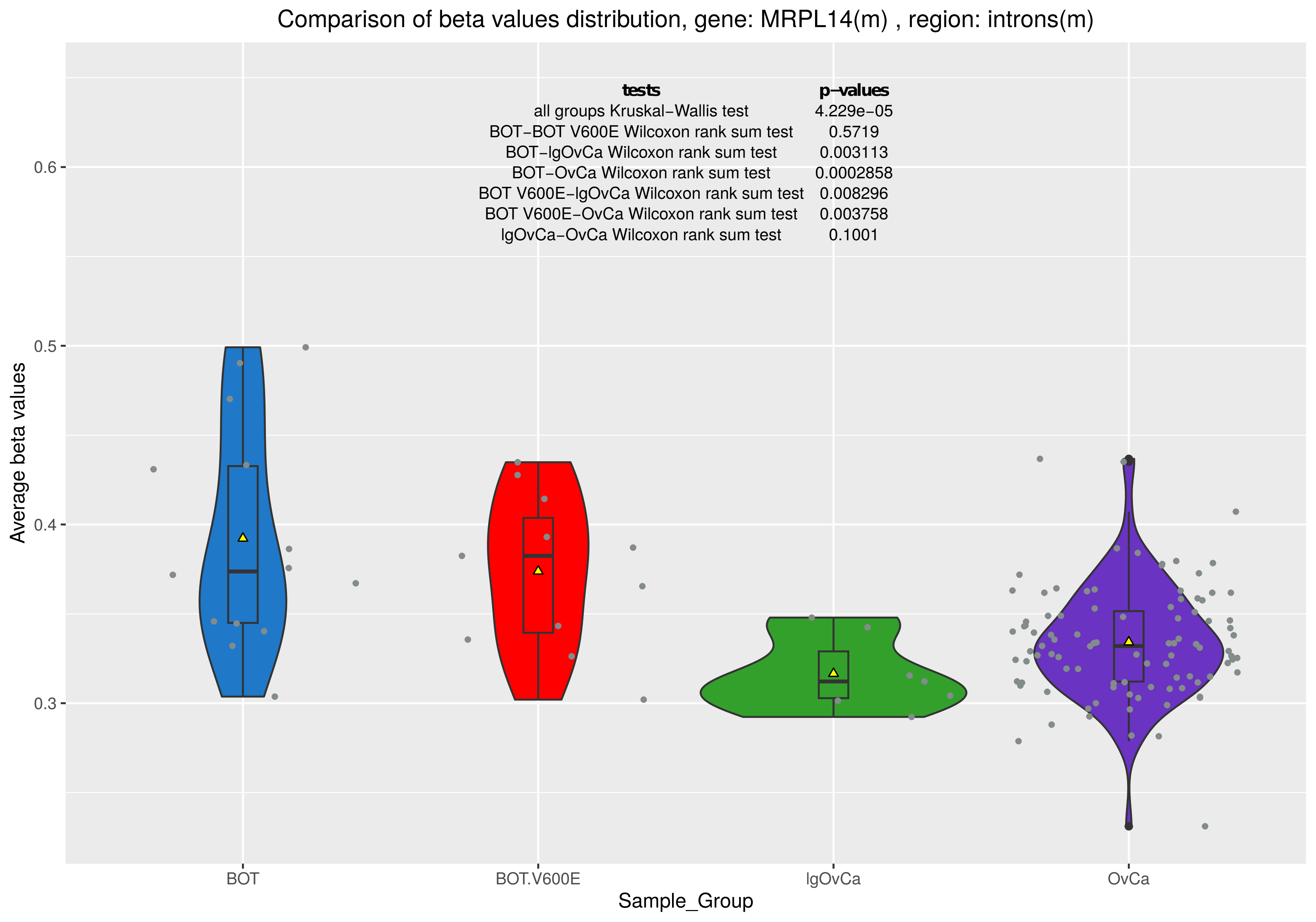

Comparison of beta values distribution, gene: MRPL14(m) , region: introns(m)
tests
p−values
all groups Kruskal−Wallis test
4.229e−05
BOT−BOT V600E Wilcoxon rank sum test
0.5719
BOT−IgOvCa Wilcoxon rank sum test
0.003113
BOT−OvCa Wilcoxon rank sum test
0.0002858
BOT V600E−IgOvCa Wilcoxon rank sum test
0.008296
BOT V600E−OvCa Wilcoxon rank sum test
0.003758
IgOvCa−OvCa Wilcoxon rank sum test
0.1001
Average beta values
0.6
0.5
0.4
0.3
BOT
BOT.V600E
IgOvCa
OvCa
Sample_Group

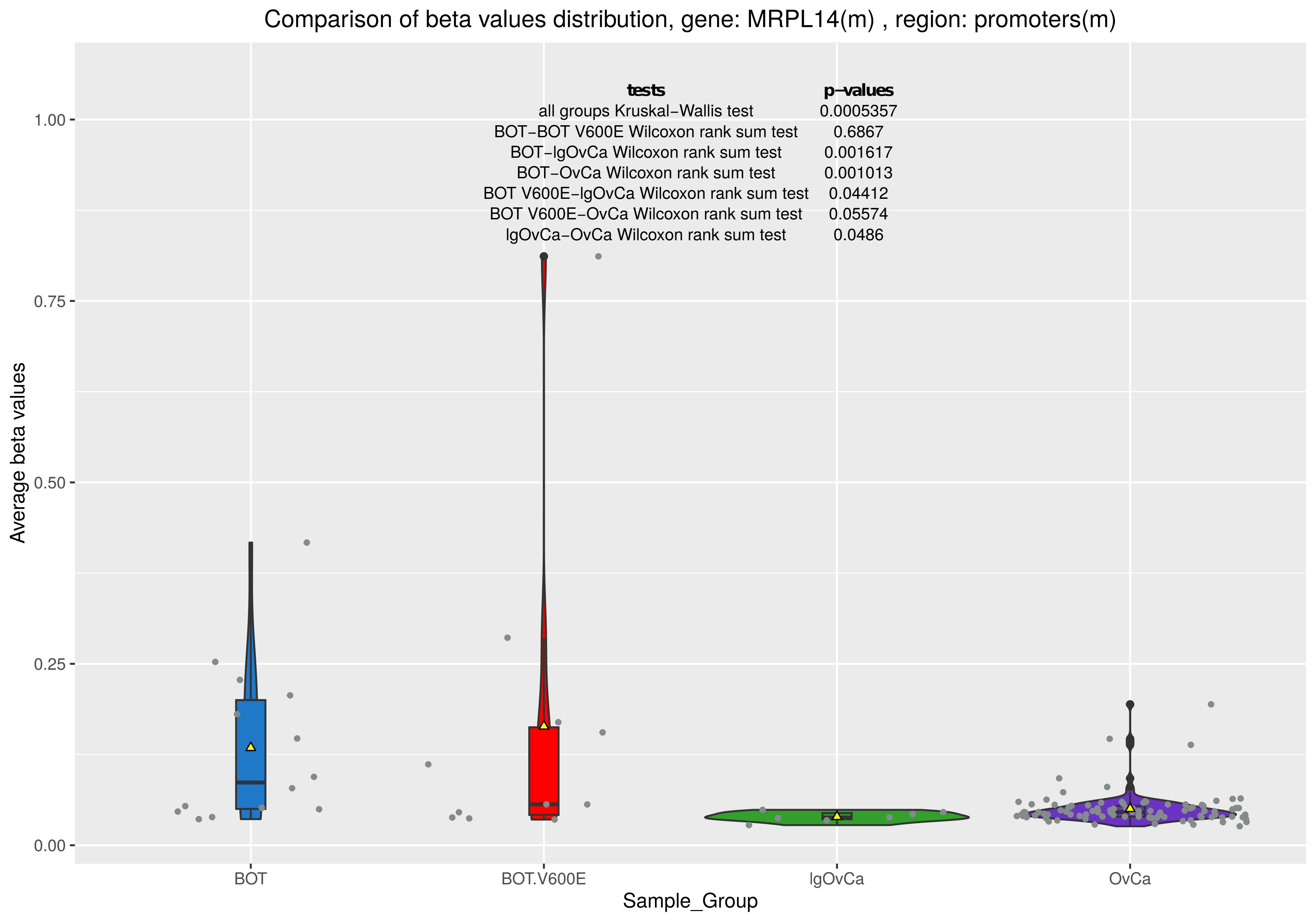
Comparison of beta values distribution, gene: MRPL14(m) , region: promoters(m)
tests
p−values
all groups Kruskal−Wallis test
0.0005357
BOT−BOT V600E Wilcoxon rank sum test
0.6867
BOT−IgOvCa Wilcoxon rank sum test
0.001617
BOT−OvCa Wilcoxon rank sum test
0.001013
BOT V600E−IgOvCa Wilcoxon rank sum test
0.04412
BOT V600E−OvCa Wilcoxon rank sum test
0.05574
IgOvCa−OvCa Wilcoxon rank sum test
0.0486
Average beta values
1.00
0.75
0.50
0.25
0.00
BOT
BOT.V600E
IgOvCa
OvCa
Sample_Group

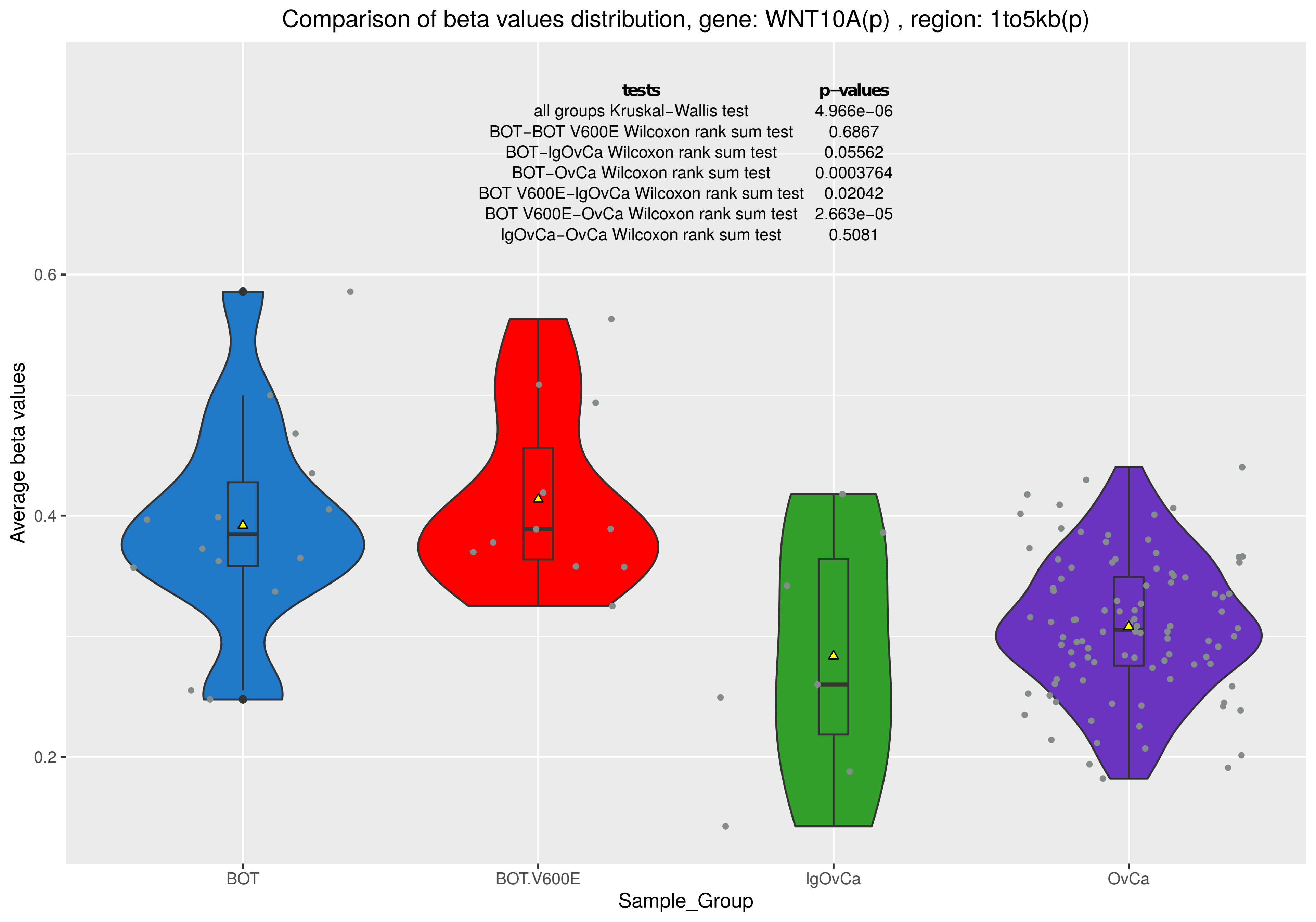
Comparison of beta values distribution, gene: WNT10A(p) , region: 1to5kb(p)
tests
p−values
all groups Kruskal−Wallis test
4.966e−06
BOT−BOT V600E Wilcoxon rank sum test
0.6867
BOT−IgOvCa Wilcoxon rank sum test
0.05562
BOT−OvCa Wilcoxon rank sum test
0.0003764
BOT V600E−IgOvCa Wilcoxon rank sum test
0.02042
BOT V600E−OvCa Wilcoxon rank sum test
2.663e−05
IgOvCa−OvCa Wilcoxon rank sum test
0.5081
Average beta values
0.6
0.4
0.2
BOT
BOT.V600E
IgOvCa
OvCa
Sample_Group

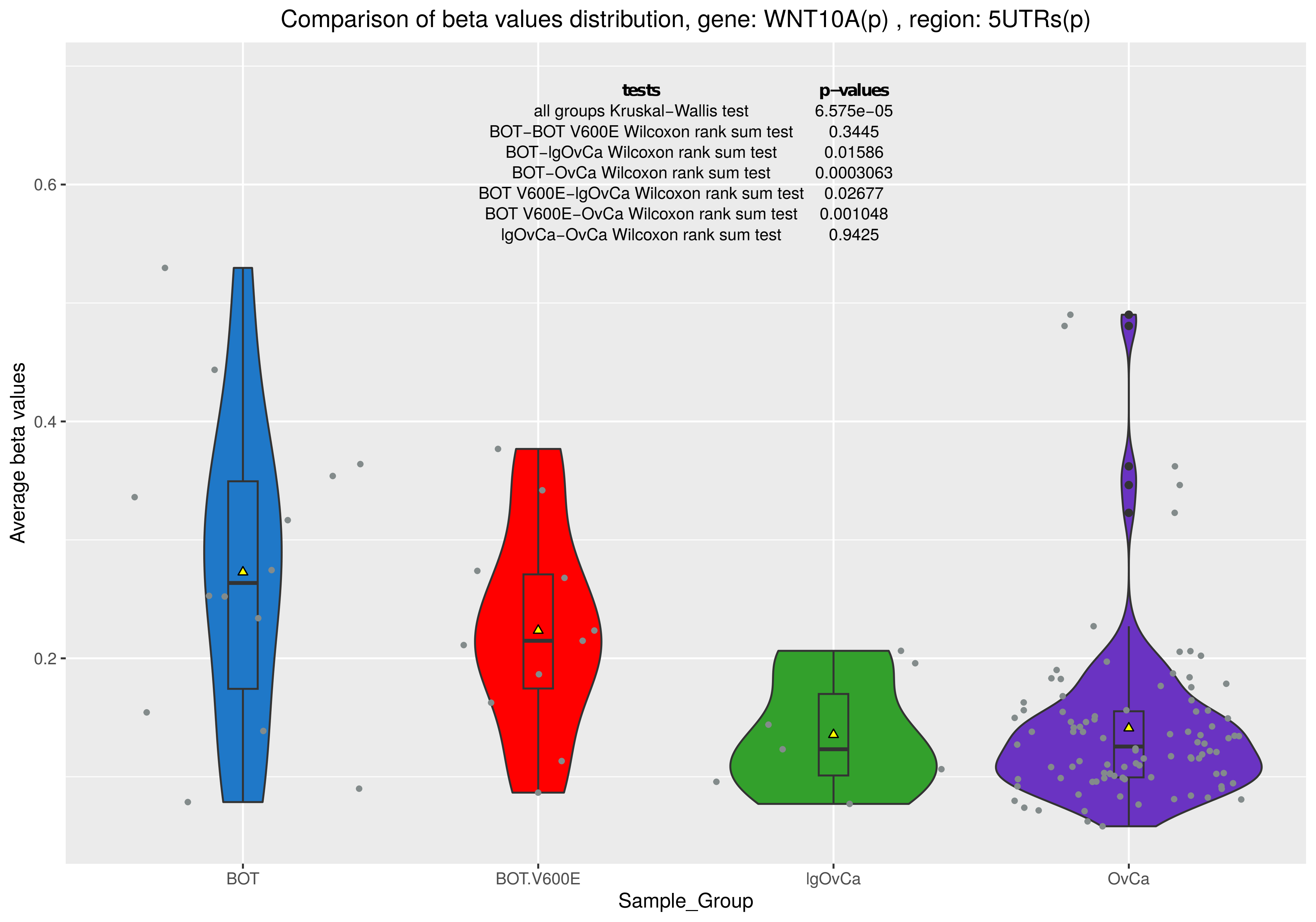
Comparison of beta values distribution, gene: WNT10A(p) , region: 5UTRs(p)
tests
p−values
all groups Kruskal−Wallis test
6.575e−05
BOT−BOT V600E Wilcoxon rank sum test
0.3445
BOT−lgOvCa Wilcoxon rank sum test
0.01586
BOT−OvCa Wilcoxon rank sum test
0.0003063
BOT V600E−lgOvCa Wilcoxon rank sum test
0.02677
BOT V600E−OvCa Wilcoxon rank sum test
0.001048
lgOvCa−OvCa Wilcoxon rank sum test
0.9425
Average beta values
0.6
0.4
0.2
BOT
BOT.V600E
lgOvCa
OvCa
Sample_Group

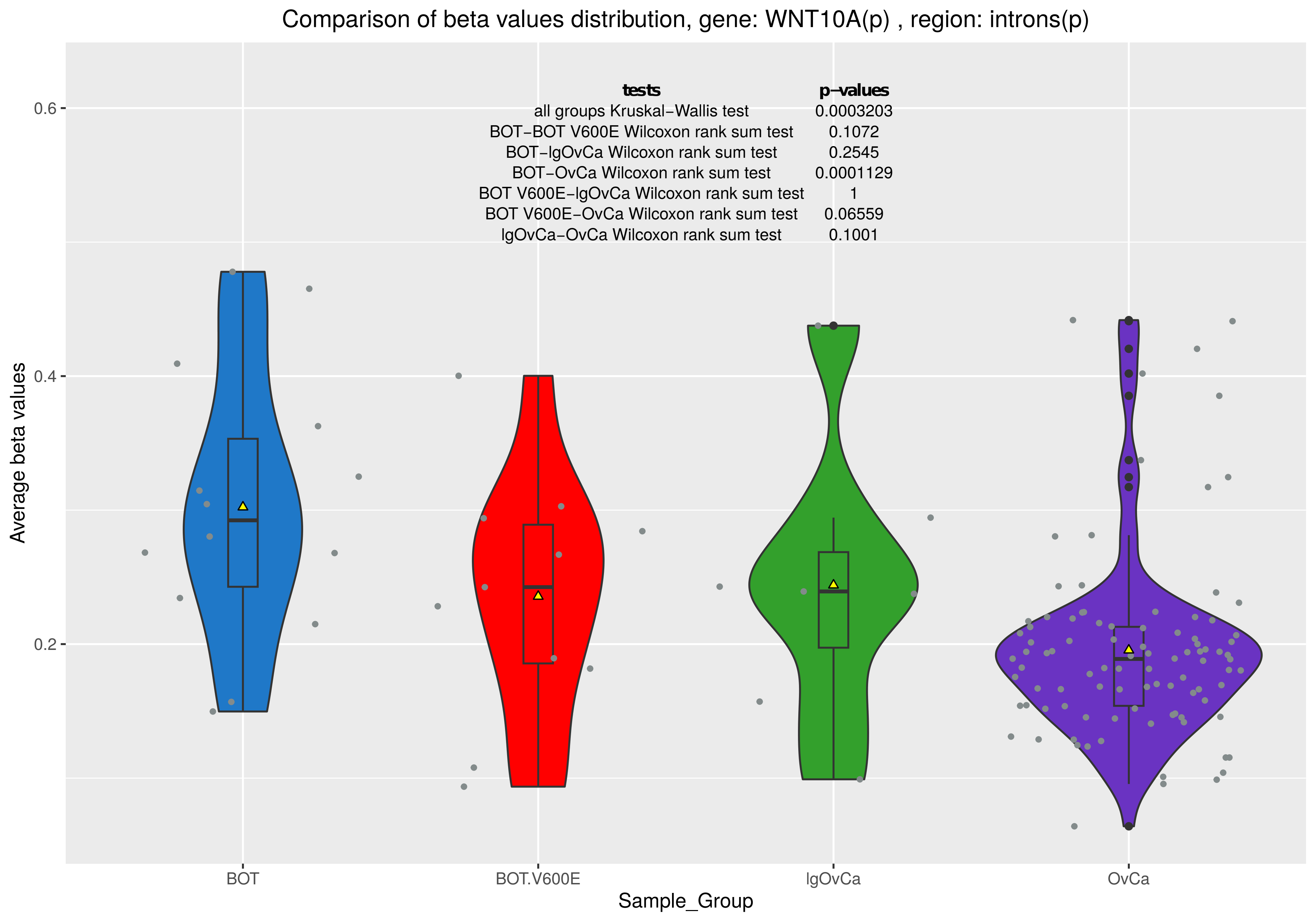
Comparison of beta values distribution, gene: WNT10A(p) , region: introns(p)
tests
p−values
all groups Kruskal−Wallis test
0.0003203
BOT−BOT V600E Wilcoxon rank sum test
0.1072
BOT−lgOvCa Wilcoxon rank sum test
0.2545
BOT−OvCa Wilcoxon rank sum test
0.0001129
BOT V600E−lgOvCa Wilcoxon rank sum test
1
BOT V600E−OvCa Wilcoxon rank sum test
0.06559
lgOvCa−OvCa Wilcoxon rank sum test
0.1001
0.6
0.4
0.2
Average beta values
BOT
BOT.V600E
lgOvCa
OvCa
Sample_Group

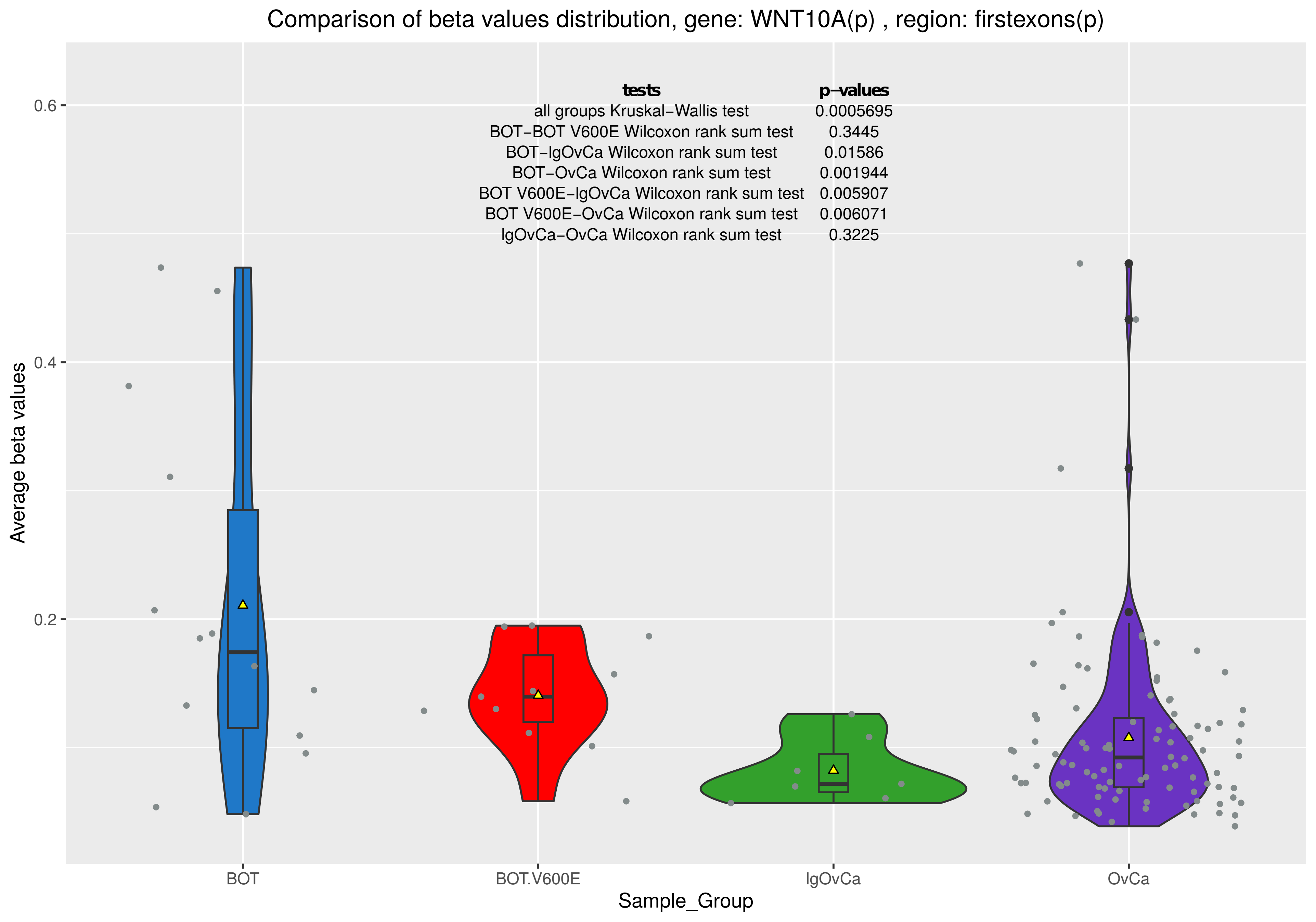

Comparison of beta values distribution, gene: WNT10A(p) , region: firstexons(p)
tests
p−values
all groups Kruskal−Wallis test
0.0005695
BOT−BOT V600E Wilcoxon rank sum test
0.3445
BOT−lgOvCa Wilcoxon rank sum test
0.01586
BOT−OvCa Wilcoxon rank sum test
0.001944
BOT V600E−lgOvCa Wilcoxon rank sum test
0.005907
BOT V600E−OvCa Wilcoxon rank sum test
0.006071
lgOvCa−OvCa Wilcoxon rank sum test
0.3225
0.6
0.4
0.2
Average beta values
BOT
BOT.V600E
lgOvCa
OvCa
Sample_Group

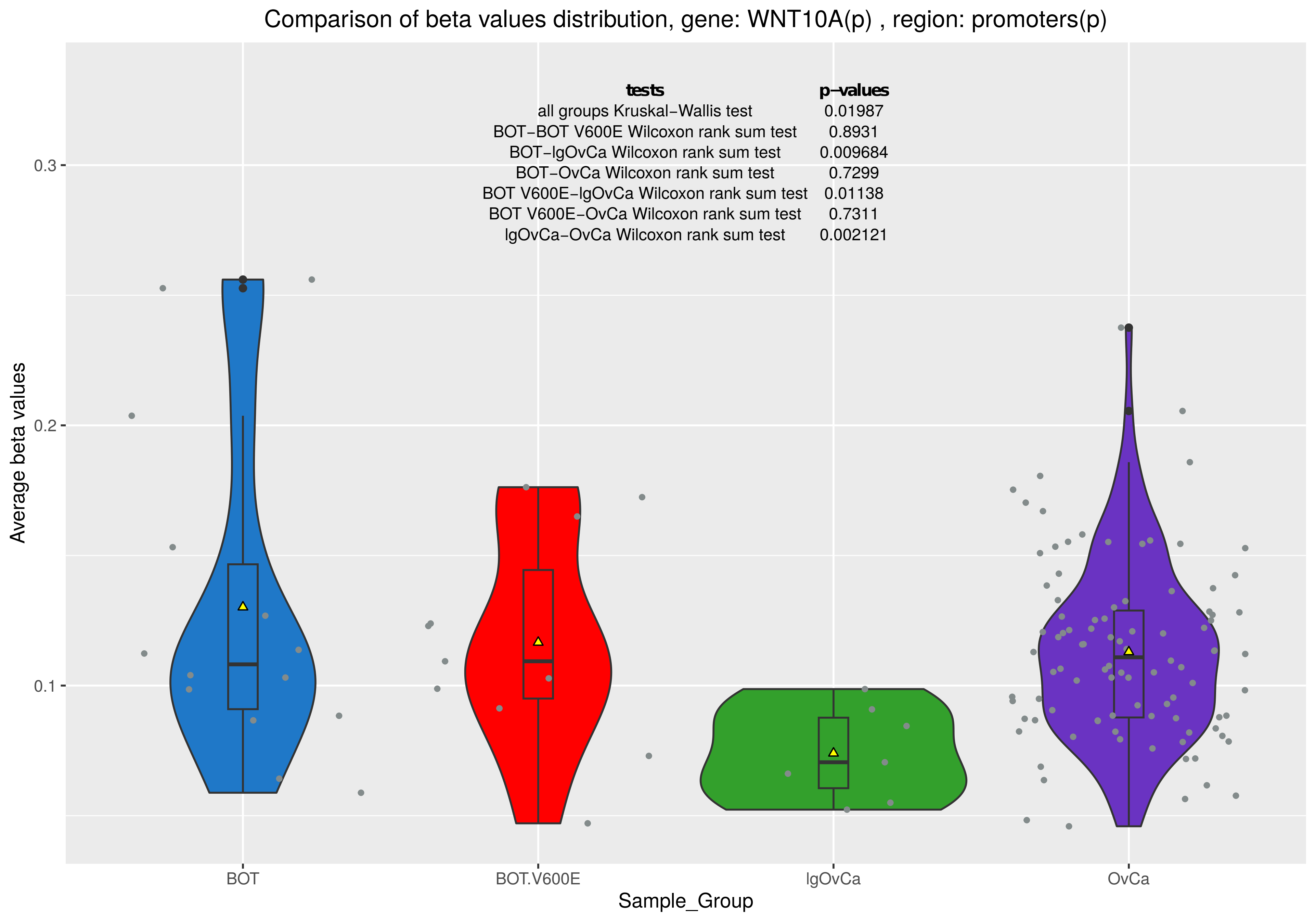
Comparison of beta values distribution, gene: WNT10A(p) , region: promoters(p)
tests
p−values
all groups Kruskal−Wallis test
0.01987
BOT−BOT V600E Wilcoxon rank sum test
0.8931
BOT−IgOvCa Wilcoxon rank sum test
0.009684
BOT−OvCa Wilcoxon rank sum test
0.7299
BOT V600E−IgOvCa Wilcoxon rank sum test
0.01138
BOT V600E−OvCa Wilcoxon rank sum test
0.7311
IgOvCa−OvCa Wilcoxon rank sum test
0.002121
Average beta values
0.3
0.2
0.1
BOT
BOT.V600E
IgOvCa
OvCa
Sample_Group

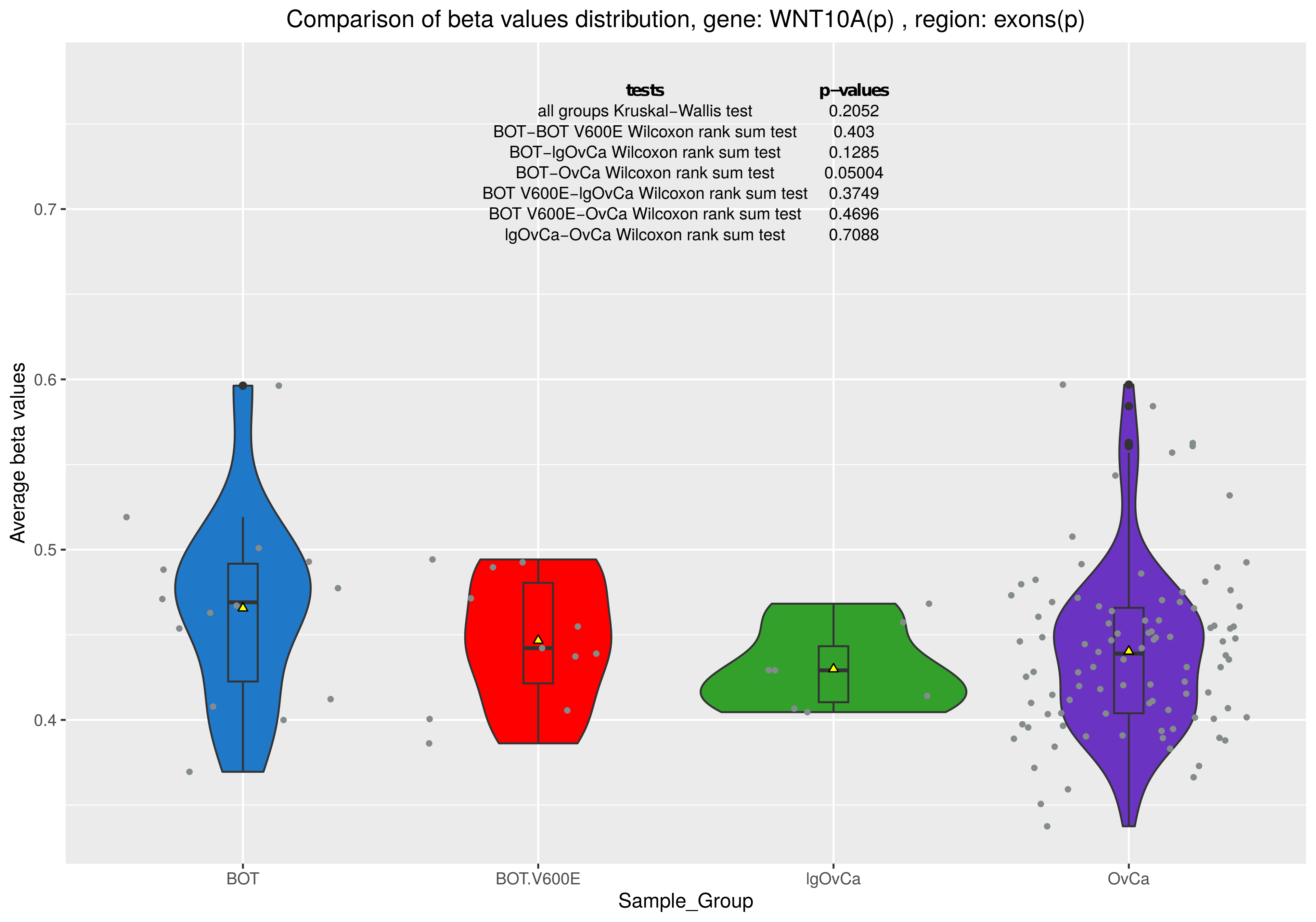
Comparison of beta values distribution, gene: WNT10A(p) , region: exons(p)
tests
p−values
all groups Kruskal−Wallis test
0.2052
BOT−BOT V600E Wilcoxon rank sum test
0.403
BOT−lgOvCa Wilcoxon rank sum test
0.1285
BOT−OvCa Wilcoxon rank sum test
0.05004
BOT V600E−lgOvCa Wilcoxon rank sum test
0.3749
BOT V600E−OvCa Wilcoxon rank sum test
0.4696
lgOvCa−OvCa Wilcoxon rank sum test
0.7088
Average beta values
0.7
0.6
0.5
0.4
BOT
BOT.V600E
lgOvCa
OvCa
Sample_Group

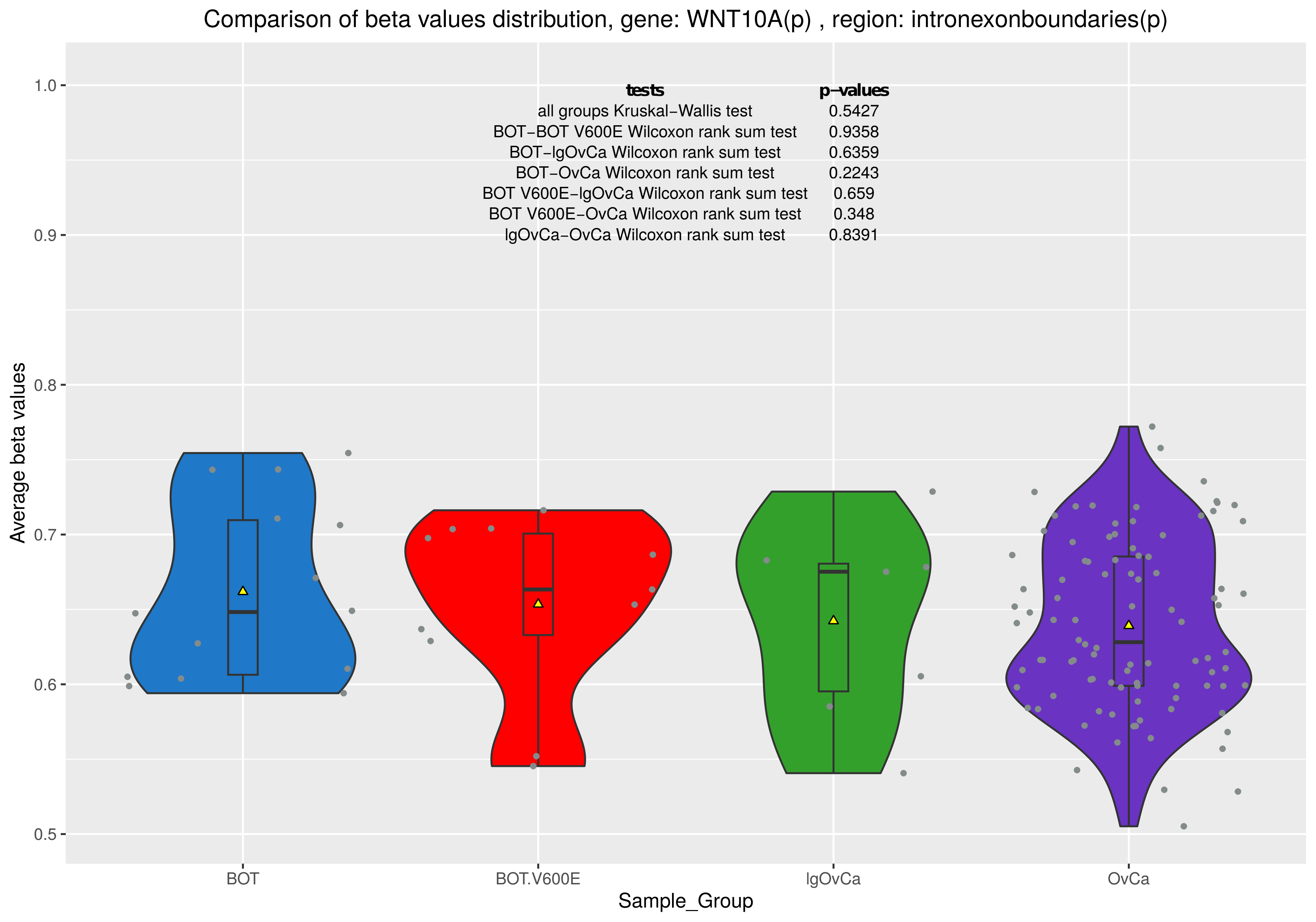

Comparison of beta values distribution, gene: WNT10A(p) , region: intronexonboundaries(p)
tests
p−values
all groups Kruskal−Wallis test 0.5427
BOT−BOT V600E Wilcoxon rank sum test 0.9358
BOT−lgOvCa Wilcoxon rank sum test 0.6359
BOT−OvCa Wilcoxon rank sum test 0.2243
BOT V600E−lgOvCa Wilcoxon rank sum test 0.659
BOT V600E−OvCa Wilcoxon rank sum test 0.348
lgOvCa−OvCa Wilcoxon rank sum test 0.8391
Average beta values
1.0
0.9
0.8
0.7
0.6
0.5
BOT
BOT.V600E
lgOvCa
OvCa
Sample_Group

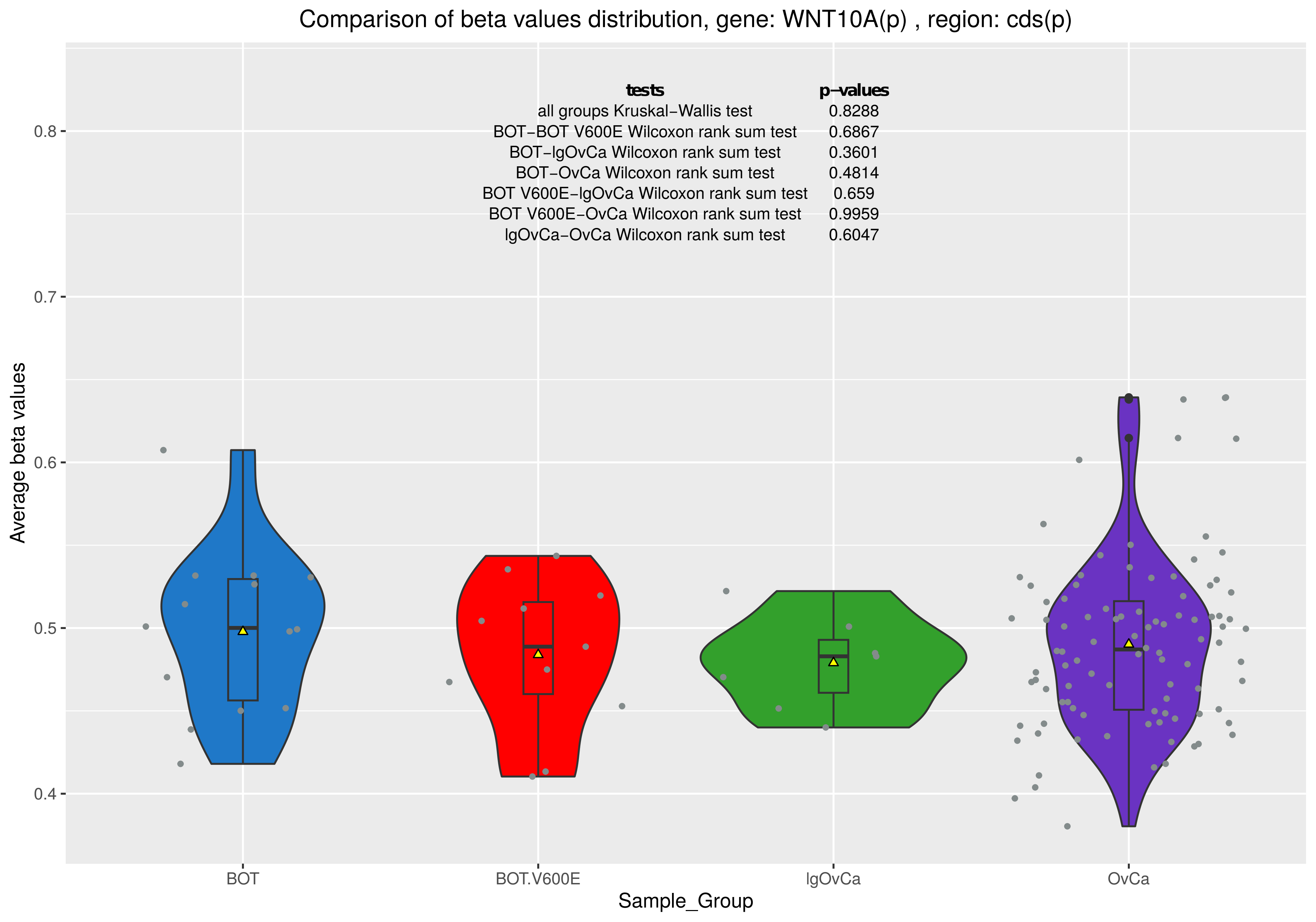

Comparison of beta values distribution, gene: WNT10A(p) , region: cds(p)
tests
p−values
all groups Kruskal−Wallis test 0.8288
BOT−BOT V600E Wilcoxon rank sum test 0.6867
BOT−lgOvCa Wilcoxon rank sum test 0.3601
BOT−OvCa Wilcoxon rank sum test 0.4814
BOT V600E−lgOvCa Wilcoxon rank sum test 0.659
BOT V600E−OvCa Wilcoxon rank sum test 0.9959
lgOvCa−OvCa Wilcoxon rank sum test 0.6047
Average beta values
0.8
0.7
0.6
0.5
0.4
BOT
BOT.V600E
lgOvCa
OvCa
Sample_Group

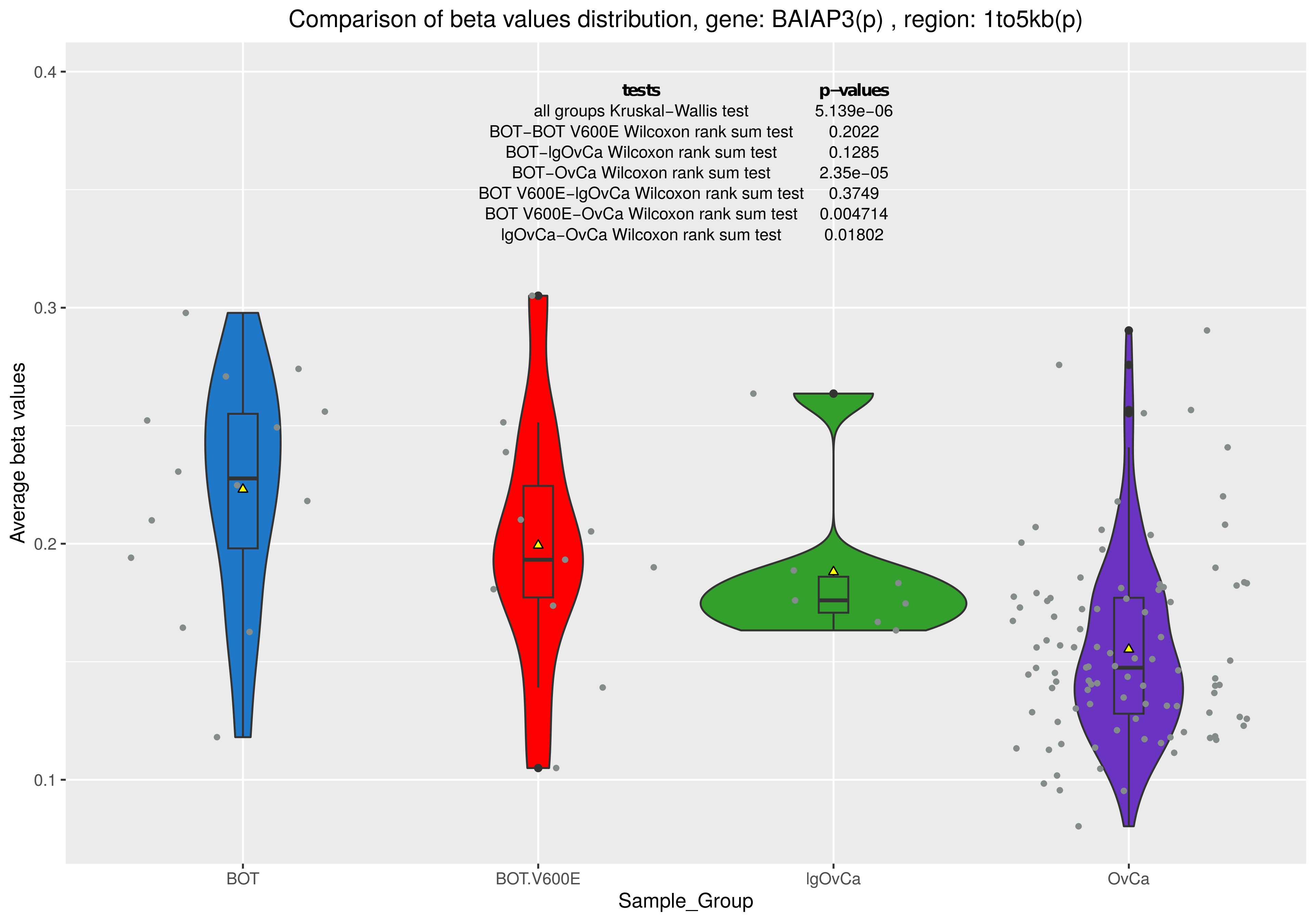

Comparison of beta values distribution, gene: BAIAP3(p) , region: 1to5kb(p)
tests
p−values
all groups Kruskal−Wallis test
5.139e−06
BOT−BOT V600E Wilcoxon rank sum test
0.2022
BOT−IgOvCa Wilcoxon rank sum test
0.1285
BOT−OvCa Wilcoxon rank sum test
2.35e−05
BOT V600E−IgOvCa Wilcoxon rank sum test
0.3749
BOT V600E−OvCa Wilcoxon rank sum test
0.004714
IgOvCa−OvCa Wilcoxon rank sum test
0.01802
Average beta values
0.4
0.3
0.2
0.1
BOT
BOT.V600E
IgOvCa
OvCa
Sample_Group

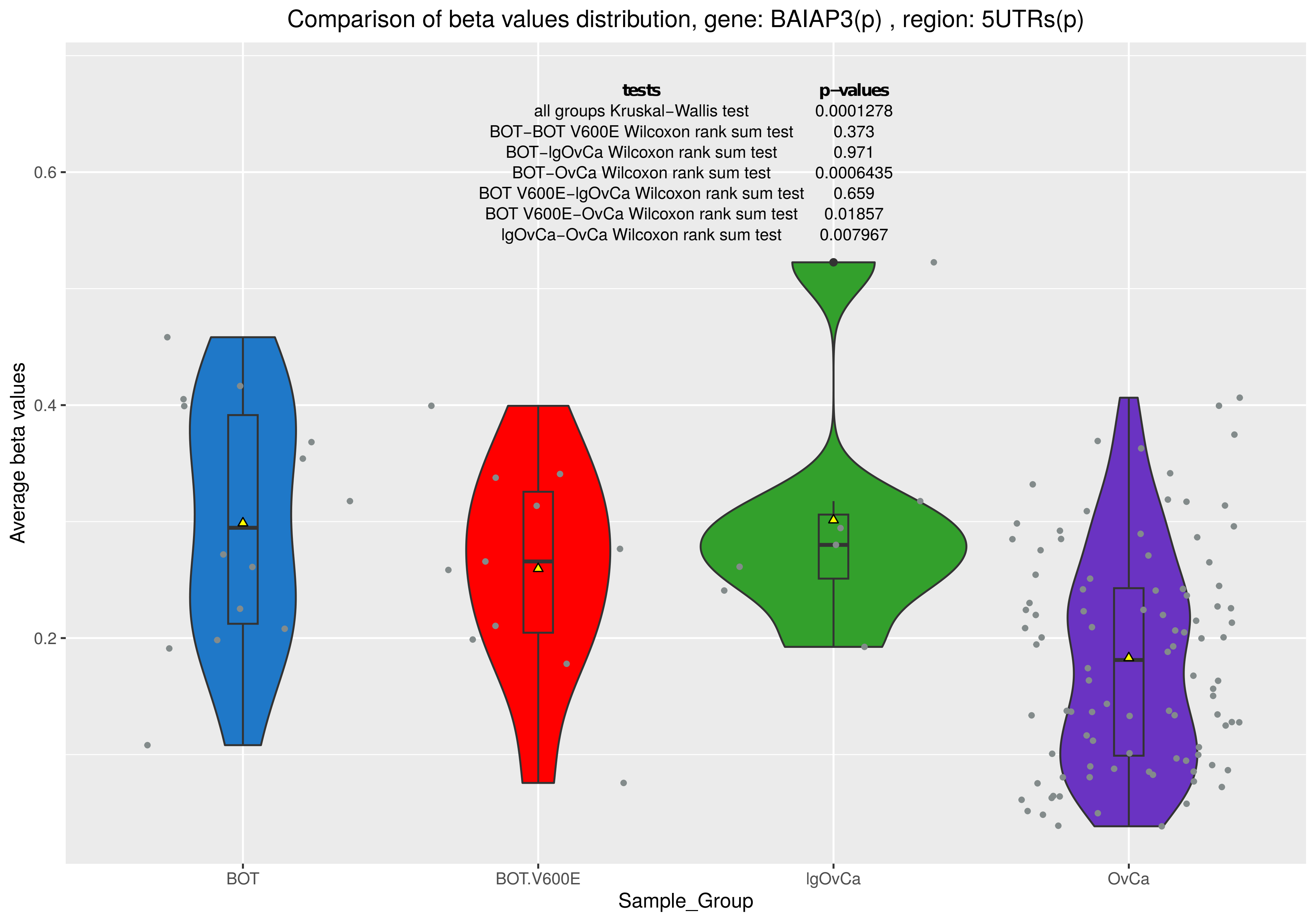
Comparison of beta values distribution, gene: BAIAP3(p) , region: 5UTRs(p)
tests
p−values
all groups Kruskal−Wallis test
0.0001278
BOT−BOT V600E Wilcoxon rank sum test
0.373
BOT−IgOvCa Wilcoxon rank sum test
0.971
BOT−OvCa Wilcoxon rank sum test
0.0006435
BOT V600E−IgOvCa Wilcoxon rank sum test
0.659
BOT V600E−OvCa Wilcoxon rank sum test
0.01857
IgOvCa−OvCa Wilcoxon rank sum test
0.007967
Average beta values
0.6
0.4
0.2
BOT
BOT.V600E
IgOvCa
OvCa
Sample_Group

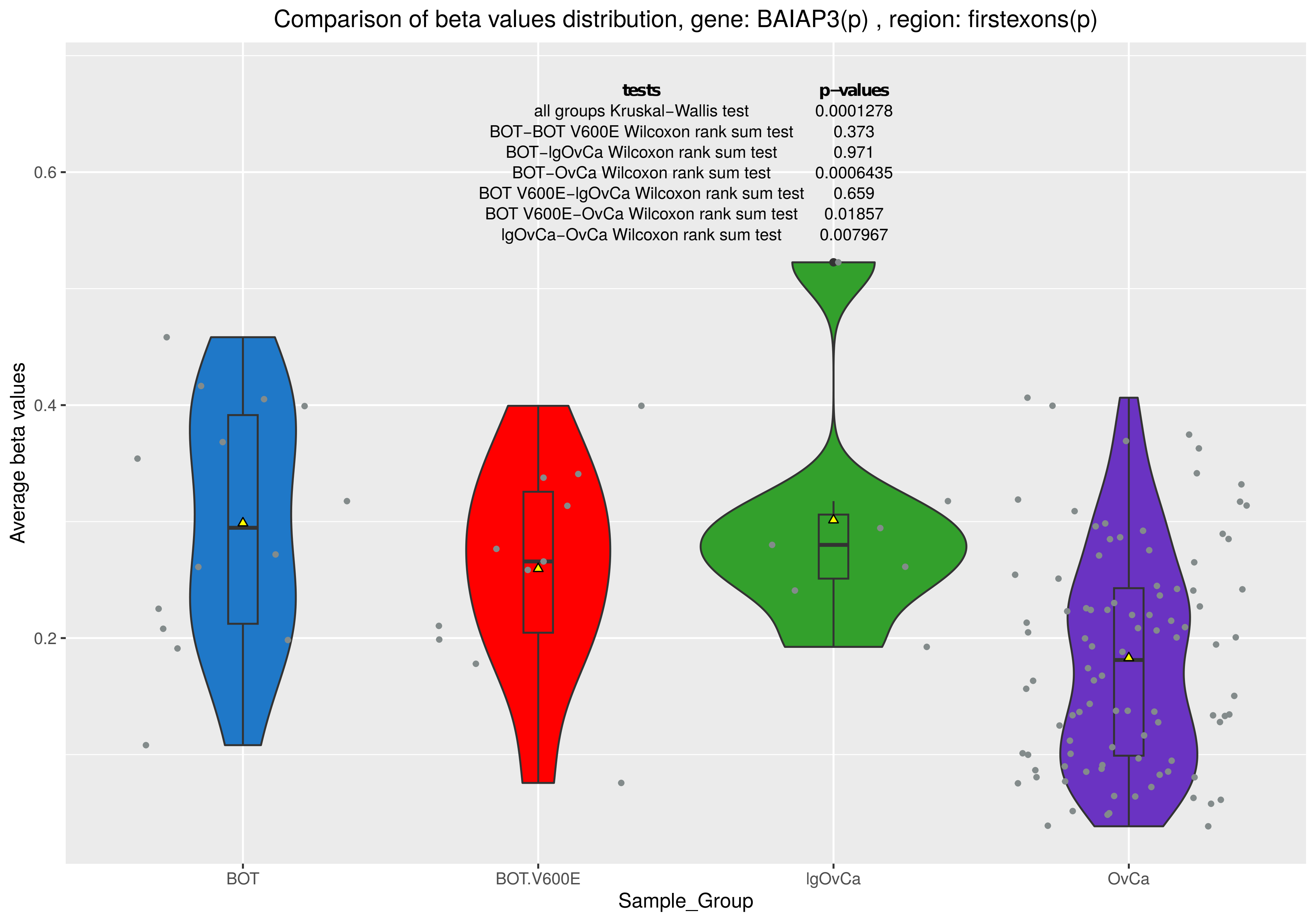

Comparison of beta values distribution, gene: BAIAP3(p) , region: firstexons(p)
tests
p−values
all groups Kruskal−Wallis test
0.0001278
BOT−BOT V600E Wilcoxon rank sum test
0.373
BOT−lgOvCa Wilcoxon rank sum test
0.971
BOT−OvCa Wilcoxon rank sum test
0.0006435
BOT V600E−lgOvCa Wilcoxon rank sum test
0.659
BOT V600E−OvCa Wilcoxon rank sum test
0.01857
lgOvCa−OvCa Wilcoxon rank sum test
0.007967
Average beta values
0.6
0.4
0.2
BOT
BOT.V600E
lgOvCa
OvCa
Sample_Group

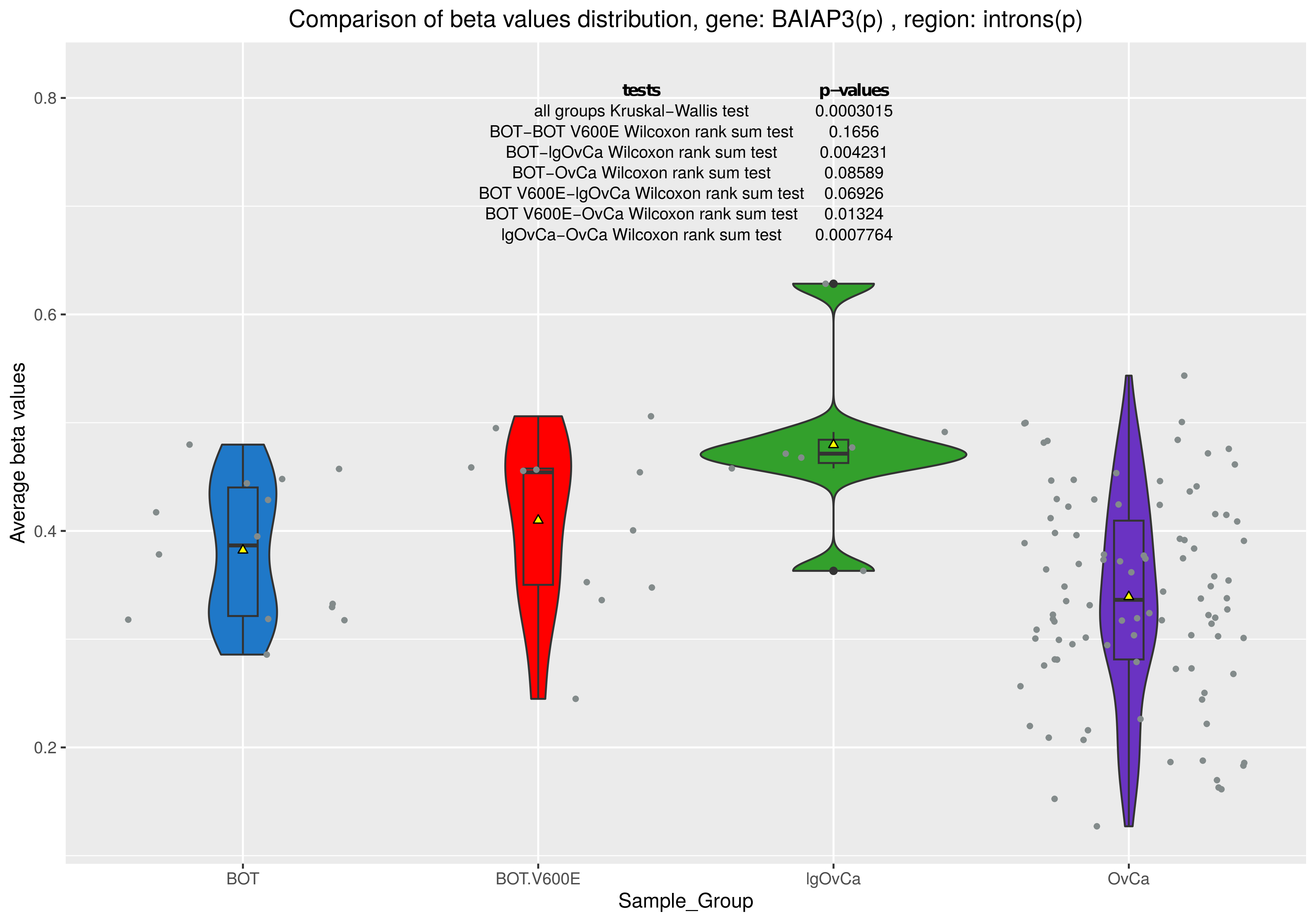
Comparison of beta values distribution, gene: BAIAP3(p) , region: introns(p)
tests
p−values
all groups Kruskal−Wallis test
0.0003015
BOT−BOT V600E Wilcoxon rank sum test
0.1656
BOT−lgOvCa Wilcoxon rank sum test
0.004231
BOT−OvCa Wilcoxon rank sum test
0.08589
BOT V600E−lgOvCa Wilcoxon rank sum test
0.06926
BOT V600E−OvCa Wilcoxon rank sum test
0.01324
lgOvCa−OvCa Wilcoxon rank sum test
0.0007764
Average beta values
0.8
0.6
0.4
0.2
BOT
BOT.V600E
lgOvCa
OvCa
Sample_Group

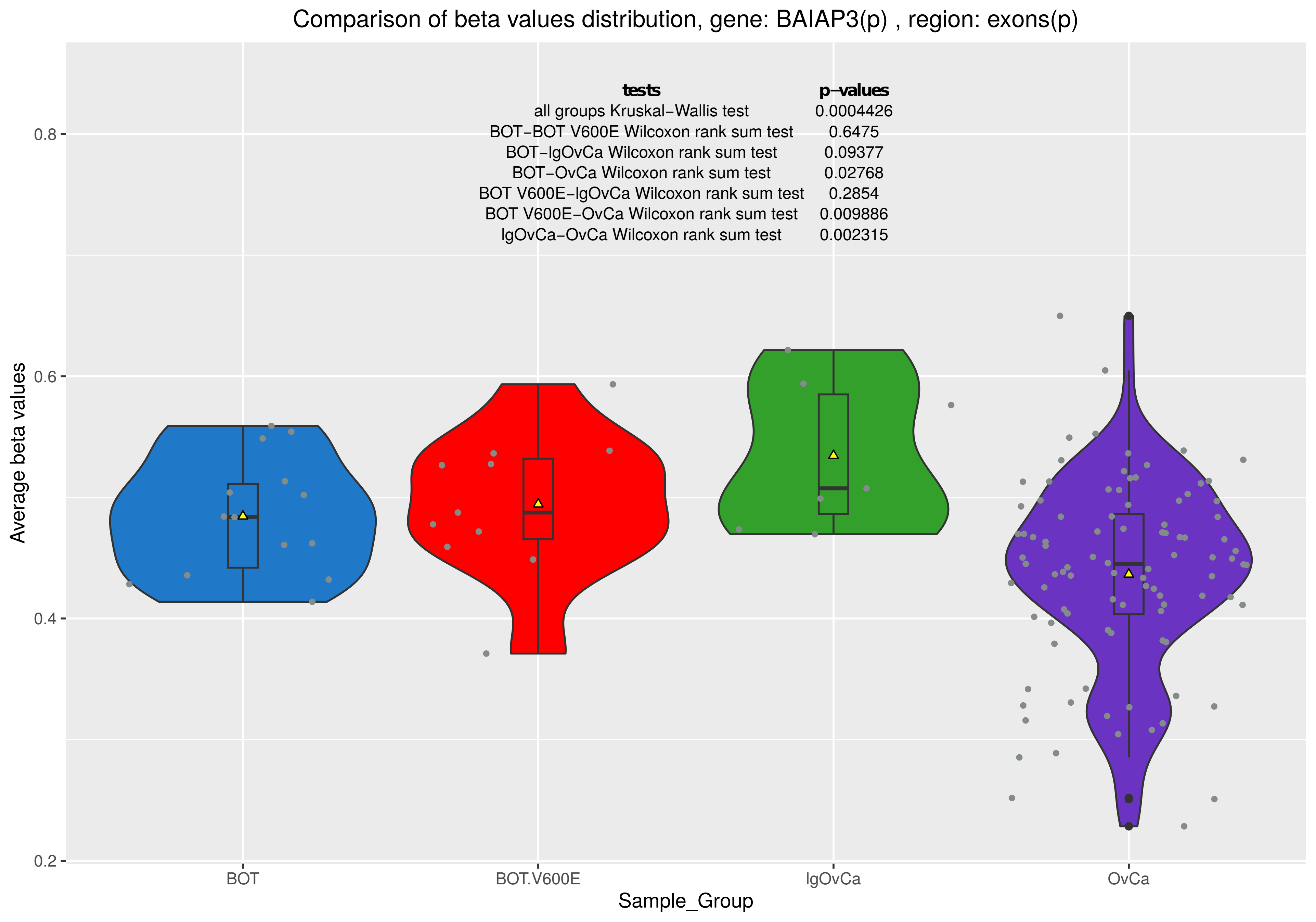

Comparison of beta values distribution, gene: BAIAP3(p) , region: exons(p)
tests
p−values
all groups Kruskal−Wallis test
0.0004426
BOT−BOT V600E Wilcoxon rank sum test
0.6475
BOT−lgOvCa Wilcoxon rank sum test
0.09377
BOT−OvCa Wilcoxon rank sum test
0.02768
BOT V600E−lgOvCa Wilcoxon rank sum test
0.2854
BOT V600E−OvCa Wilcoxon rank sum test
0.009886
lgOvCa−OvCa Wilcoxon rank sum test
0.002315
Average beta values
0.2
0.4
0.6
0.8
BOT
BOT.V600E
lgOvCa
OvCa
Sample_Group

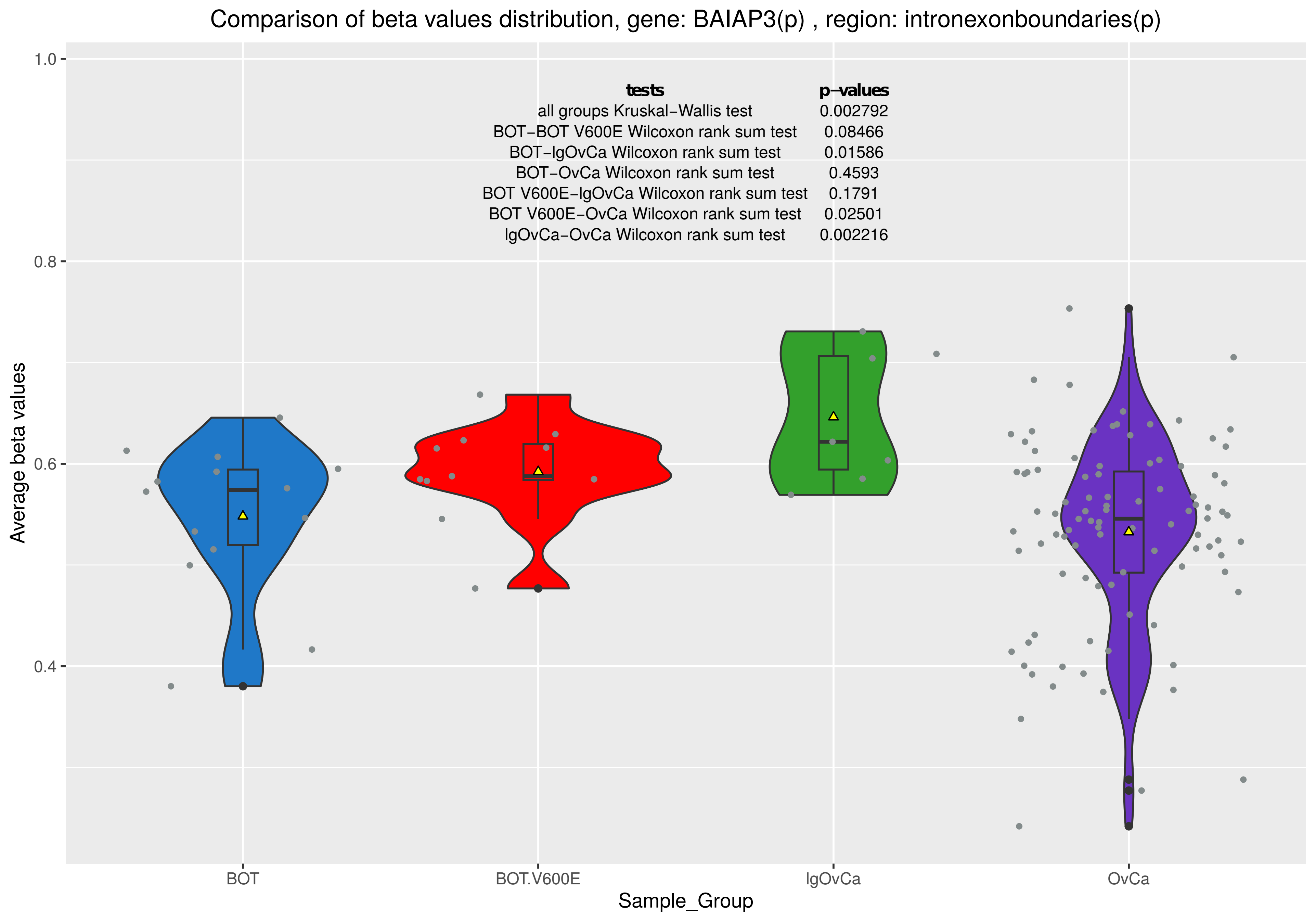

Comparison of beta values distribution, gene: BAIAP3(p) , region: intronexonboundaries(p)
tests
p−values
all groups Kruskal−Wallis test
0.002792
BOT−BOT V600E Wilcoxon rank sum test
0.08466
BOT−IgOvCa Wilcoxon rank sum test
0.01586
BOT−OvCa Wilcoxon rank sum test
0.4593
BOT V600E−IgOvCa Wilcoxon rank sum test
0.1791
BOT V600E−OvCa Wilcoxon rank sum test
0.02501
IgOvCa−OvCa Wilcoxon rank sum test
0.002216
Average beta values
1.0
0.8
0.6
0.4
BOT
BOT.V600E
IgOvCa
OvCa
Sample_Group

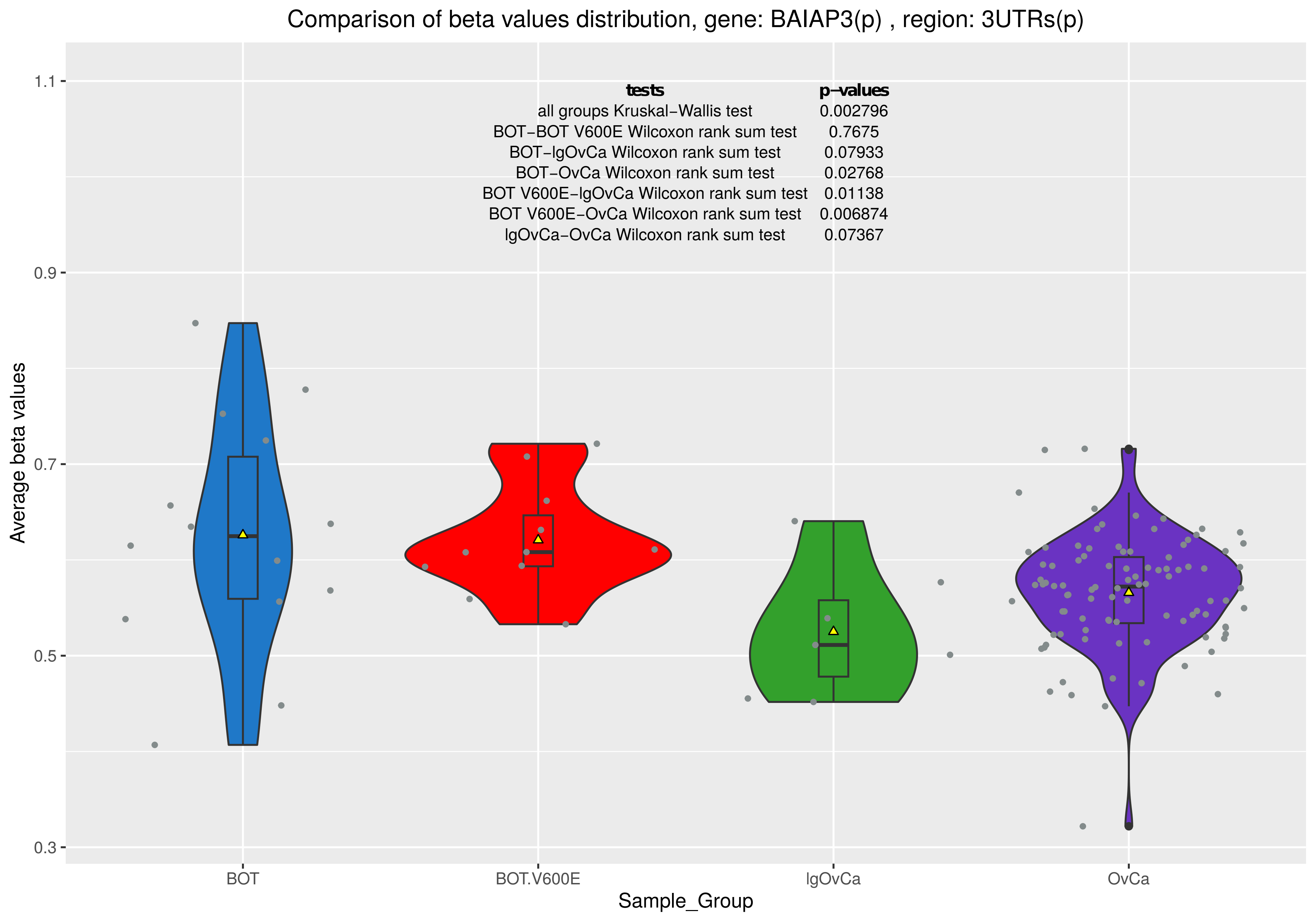

Comparison of beta values distribution, gene: BAIAP3(p) , region: 3UTRs(p)
tests
p−values
all groups Kruskal−Wallis test
0.002796
BOT−BOT V600E Wilcoxon rank sum test
0.7675
BOT−lgOvCa Wilcoxon rank sum test
0.07933
BOT−OvCa Wilcoxon rank sum test
0.02768
BOT V600E−lgOvCa Wilcoxon rank sum test
0.01138
BOT V600E−OvCa Wilcoxon rank sum test
0.006874
lgOvCa−OvCa Wilcoxon rank sum test
0.07367
Average beta values
1.1
0.9
0.7
0.5
0.3
BOT
BOT.V600E
lgOvCa
OvCa
Sample_Group

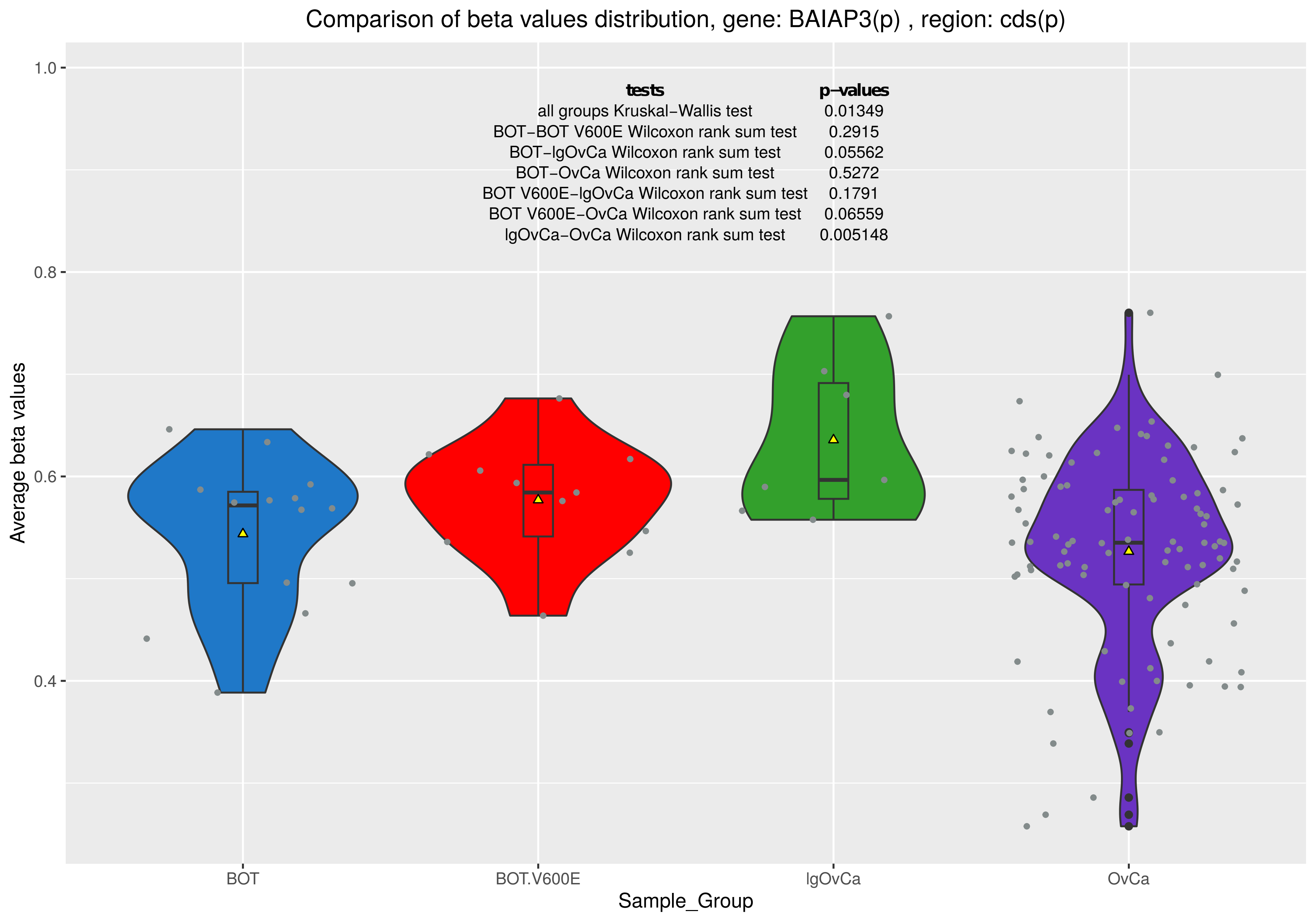
Comparison of beta values distribution, gene: BAIAP3(p) , region: cds(p)
tests
p−values
all groups Kruskal−Wallis test
0.01349
BOT−BOT V600E Wilcoxon rank sum test
0.2915
BOT−IgOvCa Wilcoxon rank sum test
0.05562
BOT−OvCa Wilcoxon rank sum test
0.5272
BOT V600E−IgOvCa Wilcoxon rank sum test
0.1791
BOT V600E−OvCa Wilcoxon rank sum test
0.06559
IgOvCa−OvCa Wilcoxon rank sum test
0.005148
Average beta values
1.0
0.8
0.6
0.4
BOT
BOT.V600E
IgOvCa
OvCa
Sample_Group

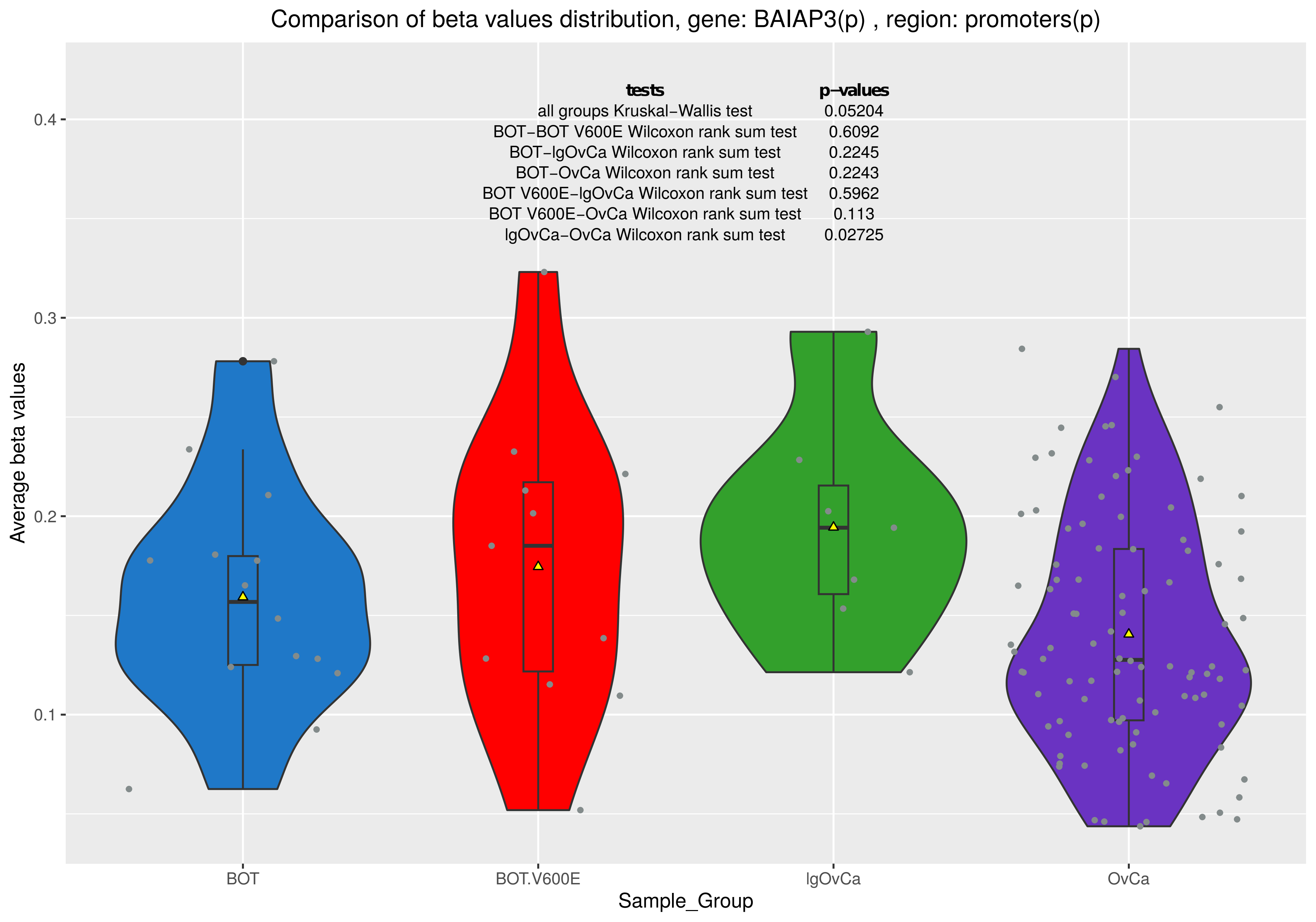
Comparison of beta values distribution, gene: BAIAP3(p) , region: promoters(p)
tests
p−values
all groups Kruskal−Wallis test
0.05204
BOT−BOT V600E Wilcoxon rank sum test
0.6092
BOT−IgOvCa Wilcoxon rank sum test
0.2245
BOT−OvCa Wilcoxon rank sum test
0.2243
BOT V600E−IgOvCa Wilcoxon rank sum test
0.5962
BOT V600E−OvCa Wilcoxon rank sum test
0.113
IgOvCa−OvCa Wilcoxon rank sum test
0.02725
Average beta values
0.4
0.3
0.2
0.1
BOT
BOT.V600E
IgOvCa
OvCa
Sample_Group

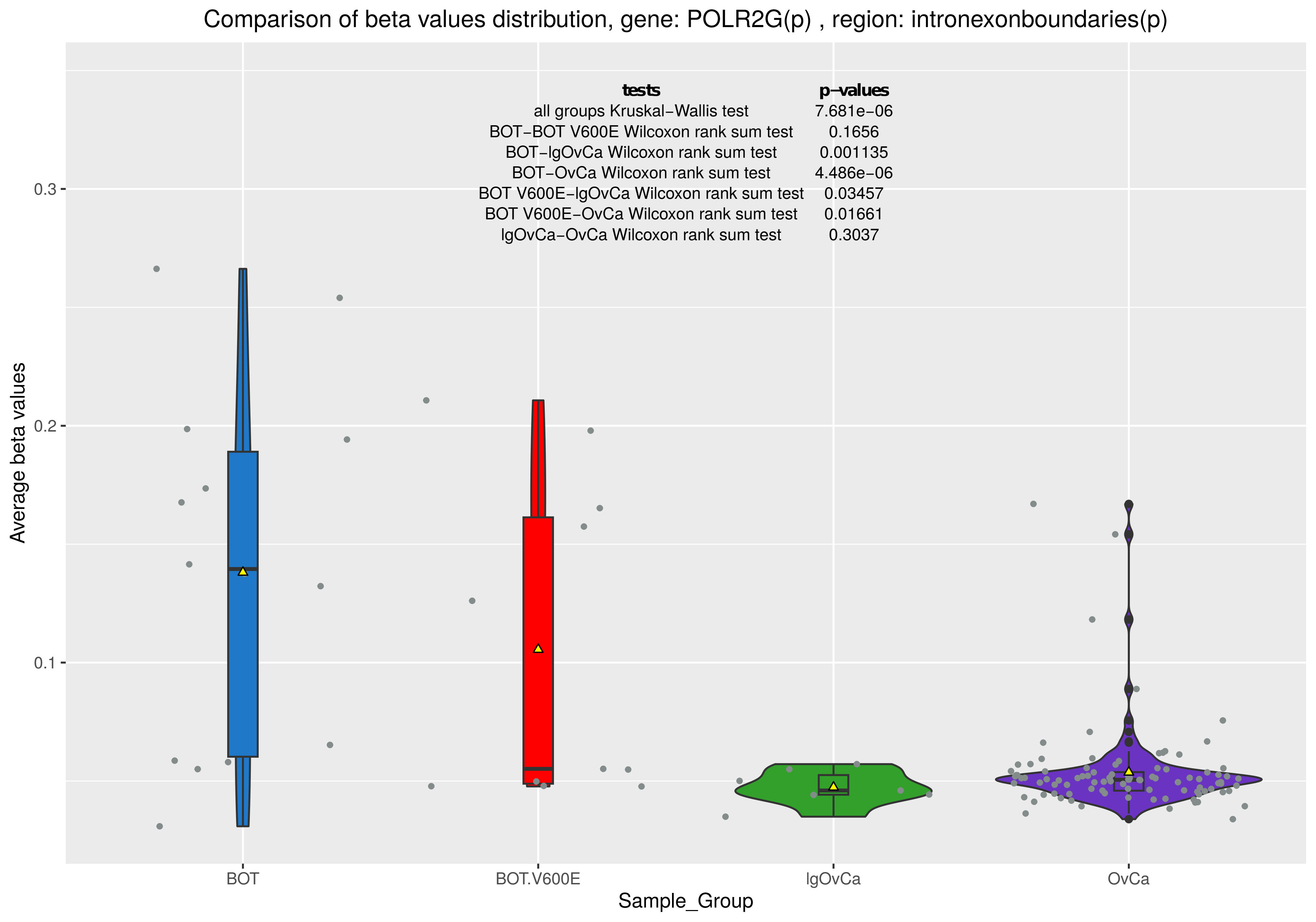

Comparison of beta values distribution, gene: POLR2G(p) , region: intronexonboundaries(p)
tests
p-values
all groups Kruskal-Wallis test
7.681e-06
BOT-BOT V600E Wilcoxon rank sum test
0.1656
BOT-lgOvCa Wilcoxon rank sum test
0.001135
BOT-OvCa Wilcoxon rank sum test
4.486e-06
BOT V600E-lgOvCa Wilcoxon rank sum test
0.03457
BOT V600E-OvCa Wilcoxon rank sum test
0.01661
lgOvCa-OvCa Wilcoxon rank sum test
0.3037
Average beta values
0.3
0.2
0.1
BOT
BOT.V600E
lgOvCa
OvCa
Sample_Group

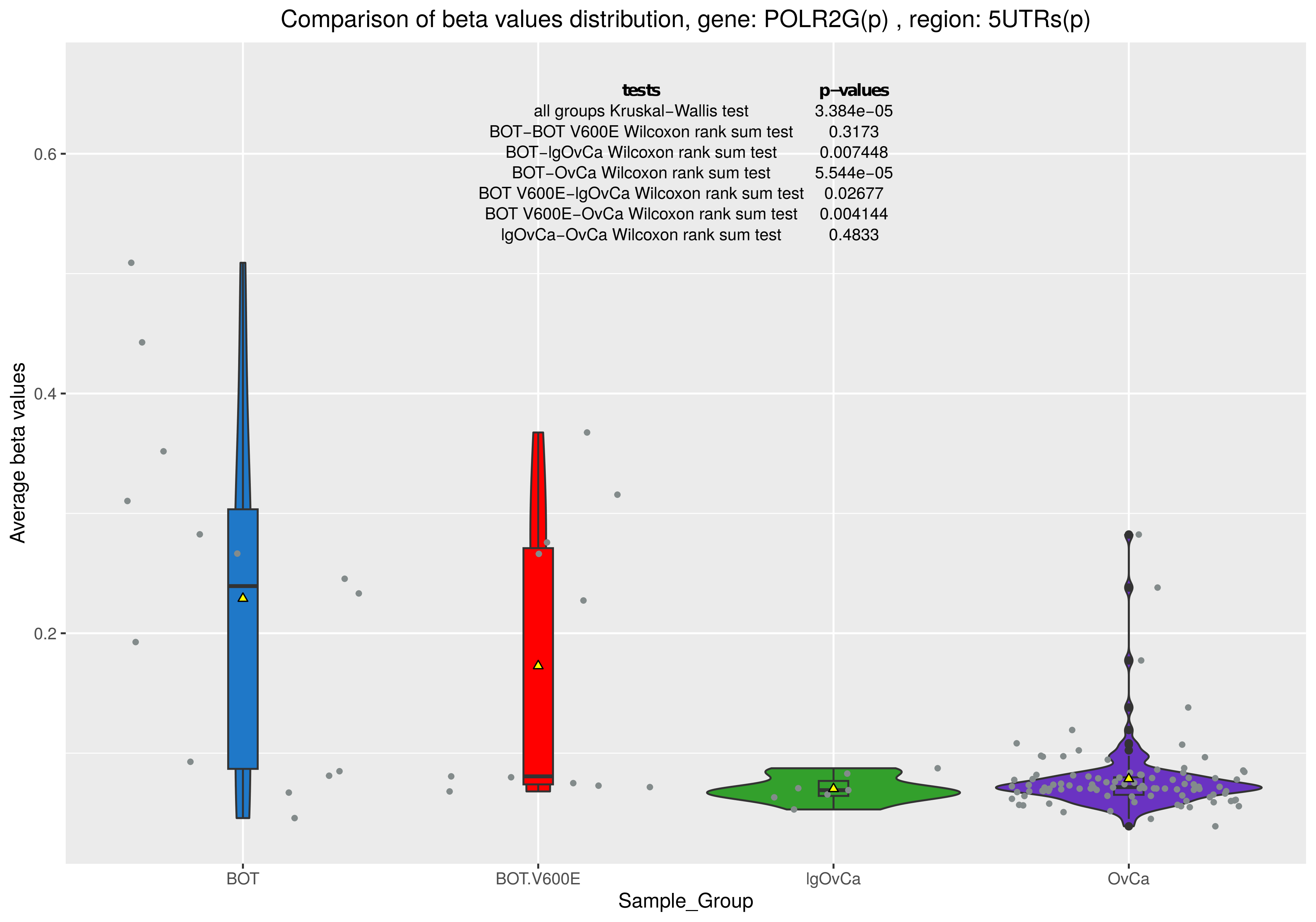

Comparison of beta values distribution, gene: POLR2G(p) , region: 5UTRs(p)
tests
p−values
all groups Kruskal−Wallis test 3.384e−05
BOT−BOT V600E Wilcoxon rank sum test 0.3173
BOT−lgOvCa Wilcoxon rank sum test 0.007448
BOT−OvCa Wilcoxon rank sum test 5.544e−05
BOT V600E−lgOvCa Wilcoxon rank sum test 0.02677
BOT V600E−OvCa Wilcoxon rank sum test 0.004144
lgOvCa−OvCa Wilcoxon rank sum test 0.4833
Average beta values
0.6
0.4
0.2
BOT
BOT.V600E
lgOvCa
OvCa
Sample_Group

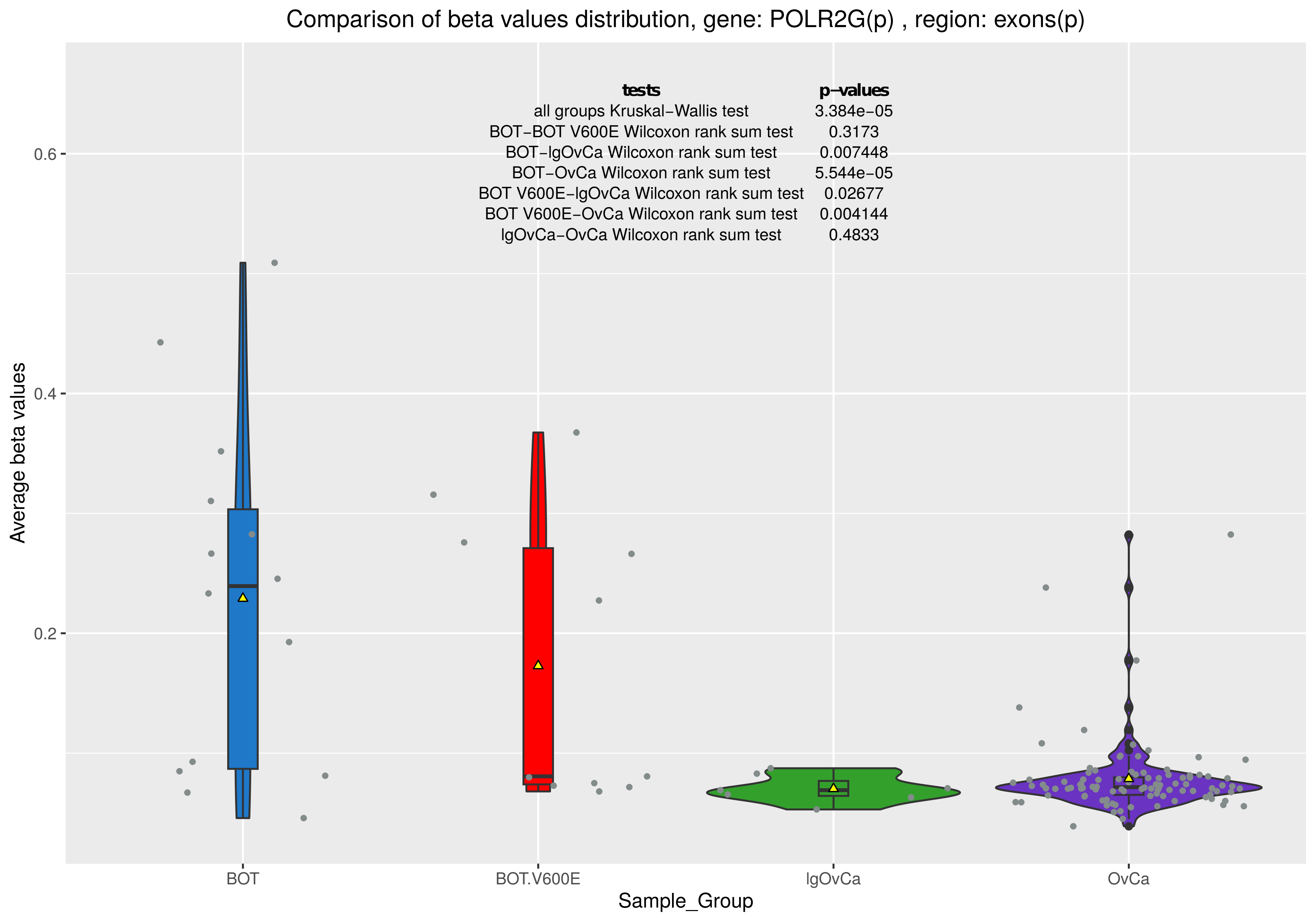

Comparison of beta values distribution, gene: POLR2G(p) , region: exons(p)
tests
p−values
all groups Kruskal−Wallis test
3.384e−05
BOT−BOT V600E Wilcoxon rank sum test
0.3173
BOT−lgOvCa Wilcoxon rank sum test
0.007448
BOT−OvCa Wilcoxon rank sum test
5.544e−05
BOT V600E−lgOvCa Wilcoxon rank sum test
0.02677
BOT V600E−OvCa Wilcoxon rank sum test
0.004144
lgOvCa−OvCa Wilcoxon rank sum test
0.4833
Average beta values
0.6
0.4
0.2
BOT
BOT.V600E
lgOvCa
OvCa
Sample_Group

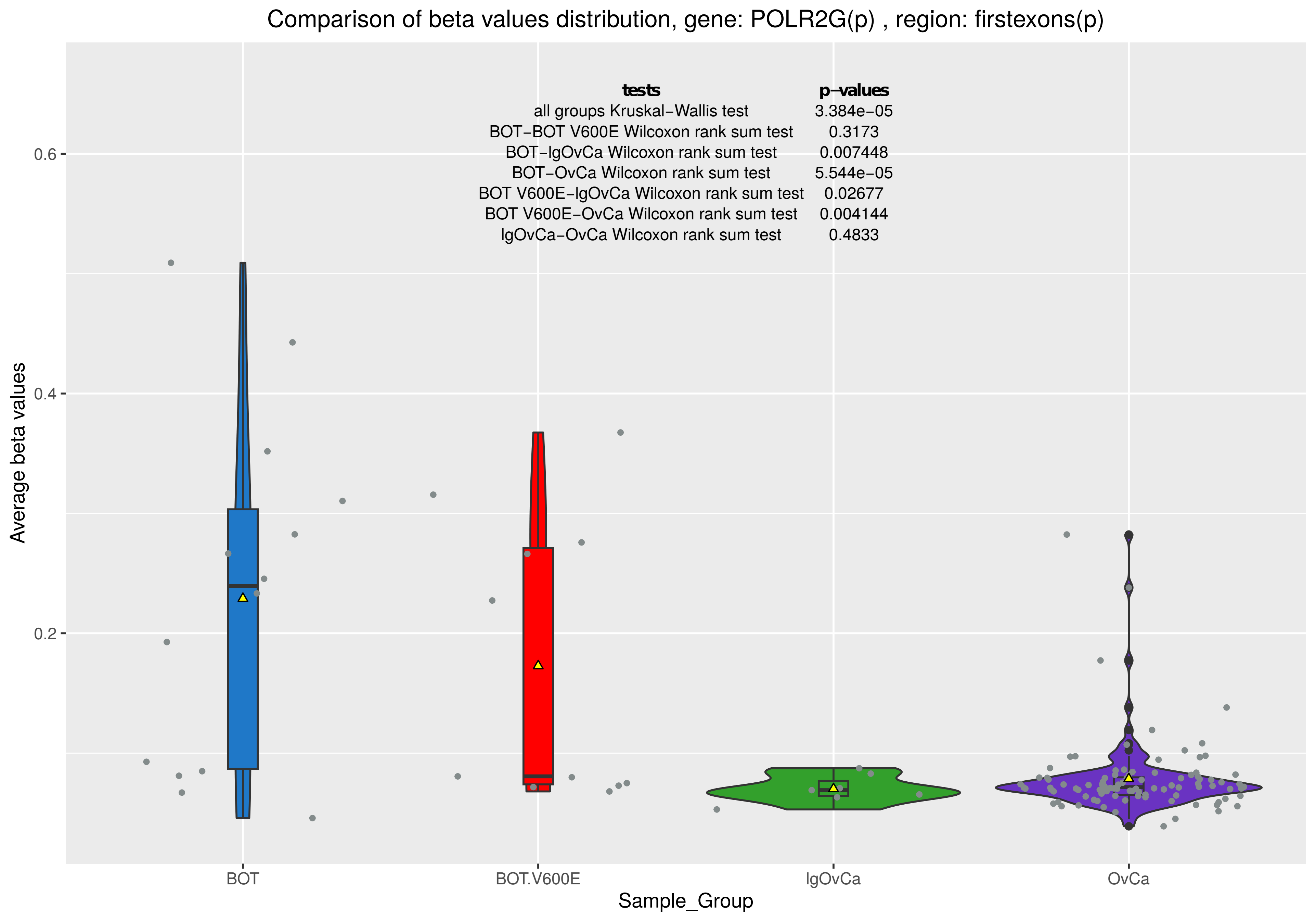
Comparison of beta values distribution, gene: POLR2G(p) , region: firstexons(p)
tests
p−values
all groups Kruskal−Wallis test
3.384e−05
BOT−BOT V600E Wilcoxon rank sum test
0.3173
BOT−lgOvCa Wilcoxon rank sum test
0.007448
BOT−OvCa Wilcoxon rank sum test
5.544e−05
BOT V600E−lgOvCa Wilcoxon rank sum test
0.02677
BOT V600E−OvCa Wilcoxon rank sum test
0.004144
lgOvCa−OvCa Wilcoxon rank sum test
0.4833
Average beta values
0.6
0.4
0.2
BOT
BOT.V600E
lgOvCa
OvCa
Sample_Group

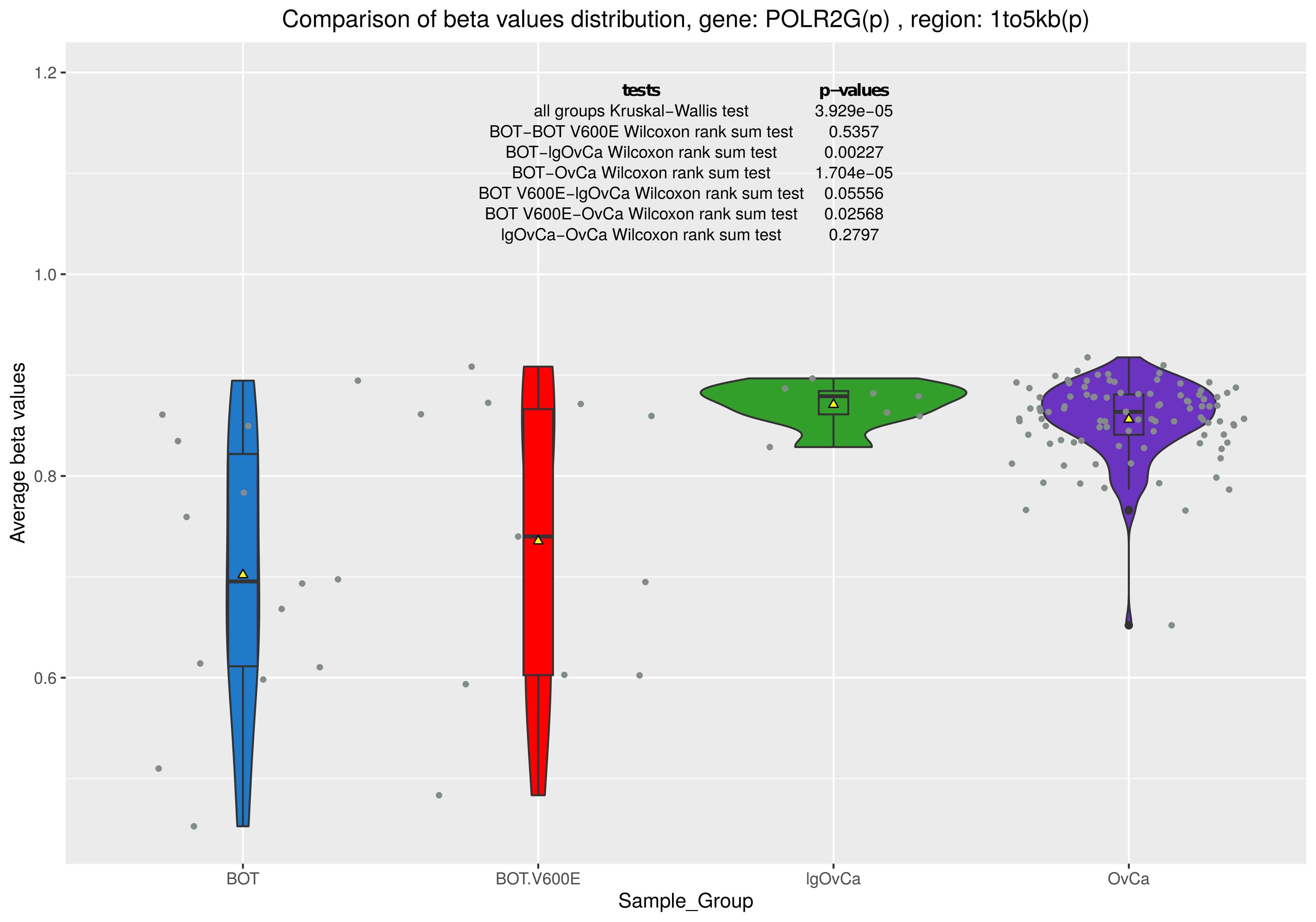

Comparison of beta values distribution, gene: POLR2G(p) , region: 1to5kb(p)
tests
p−values
all groups Kruskal−Wallis test
3.929e−05
BOT−BOT V600E Wilcoxon rank sum test
0.5357
BOT−lgOvCa Wilcoxon rank sum test
0.00227
BOT−OvCa Wilcoxon rank sum test
1.704e−05
BOT V600E−lgOvCa Wilcoxon rank sum test
0.05556
BOT V600E−OvCa Wilcoxon rank sum test
0.02568
lgOvCa−OvCa Wilcoxon rank sum test
0.2797
Average beta values
1.2
1.0
0.8
0.6
BOT
BOT.V600E
lgOvCa
OvCa
Sample_Group

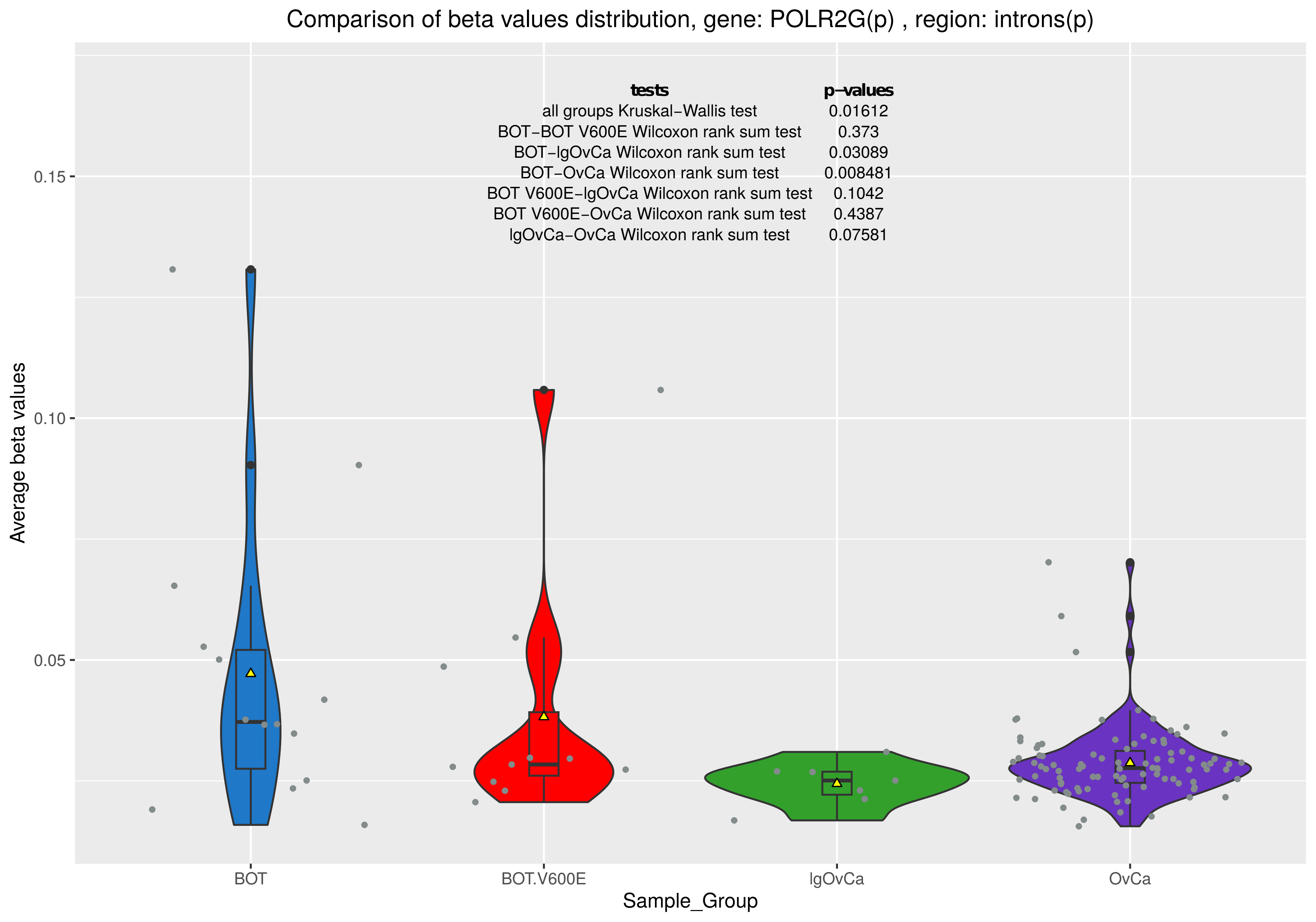

Comparison of beta values distribution, gene: POLR2G(p) , region: introns(p)
tests
p−values
all groups Kruskal−Wallis test
0.01612
BOT−BOT V600E Wilcoxon rank sum test
0.373
BOT−lgOvCa Wilcoxon rank sum test
0.03089
BOT−OvCa Wilcoxon rank sum test
0.008481
BOT V600E−lgOvCa Wilcoxon rank sum test
0.1042
BOT V600E−OvCa Wilcoxon rank sum test
0.4387
lgOvCa−OvCa Wilcoxon rank sum test
0.07581
Average beta values
0.15
0.10
0.05
BOT
BOT.V600E
lgOvCa
OvCa
Sample_Group

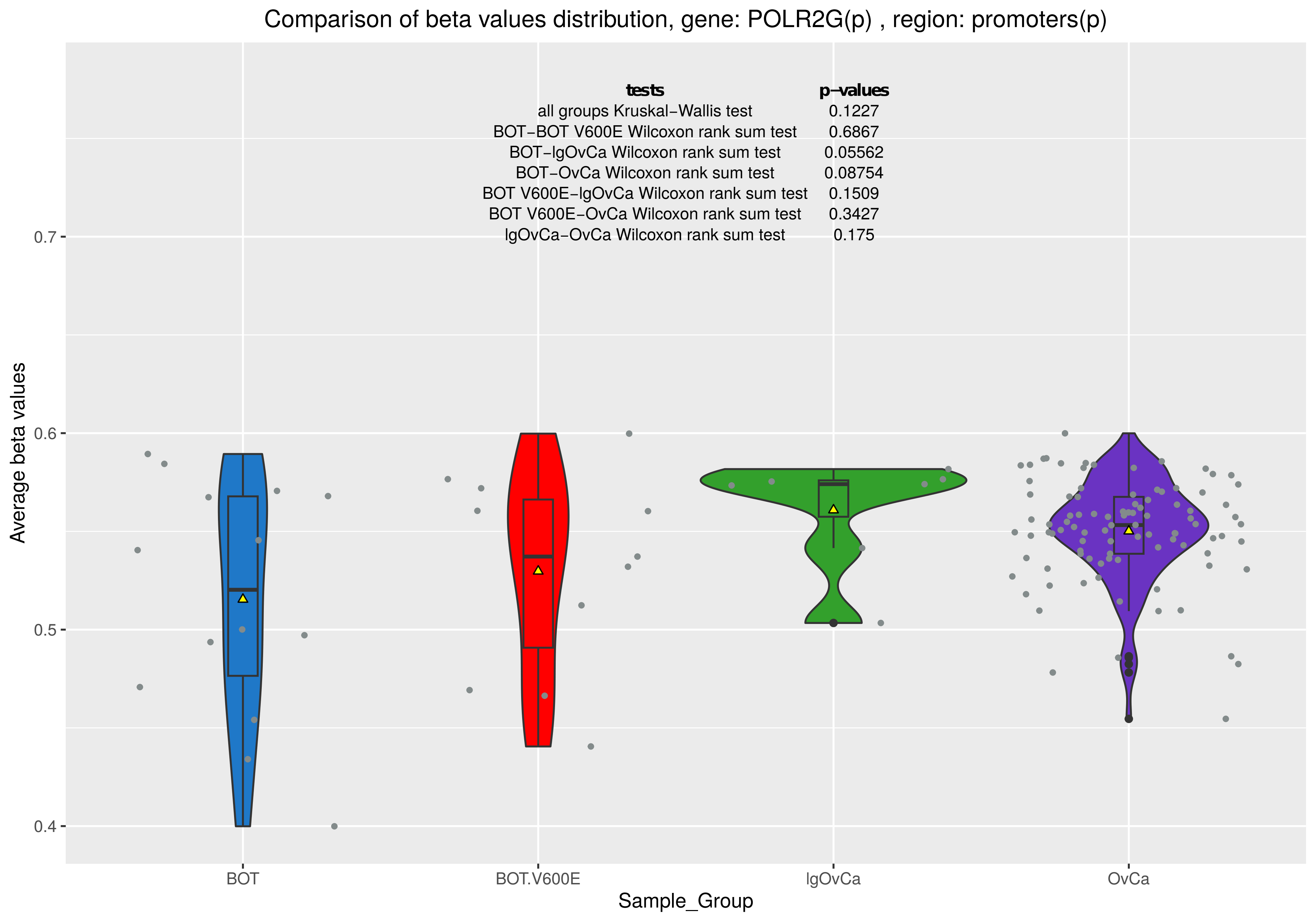

Comparison of beta values distribution, gene: POLR2G(p) , region: promoters(p)
tests
p−values
all groups Kruskal−Wallis test
0.1227
BOT−BOT V600E Wilcoxon rank sum test
0.6867
BOT−lgOvCa Wilcoxon rank sum test
0.05562
BOT−OvCa Wilcoxon rank sum test
0.08754
BOT V600E−lgOvCa Wilcoxon rank sum test
0.1509
BOT V600E−OvCa Wilcoxon rank sum test
0.3427
lgOvCa−OvCa Wilcoxon rank sum test
0.175
Average beta values
0.7
0.6
0.5
0.4
BOT
BOT.V600E
lgOvCa
OvCa
Sample_Group

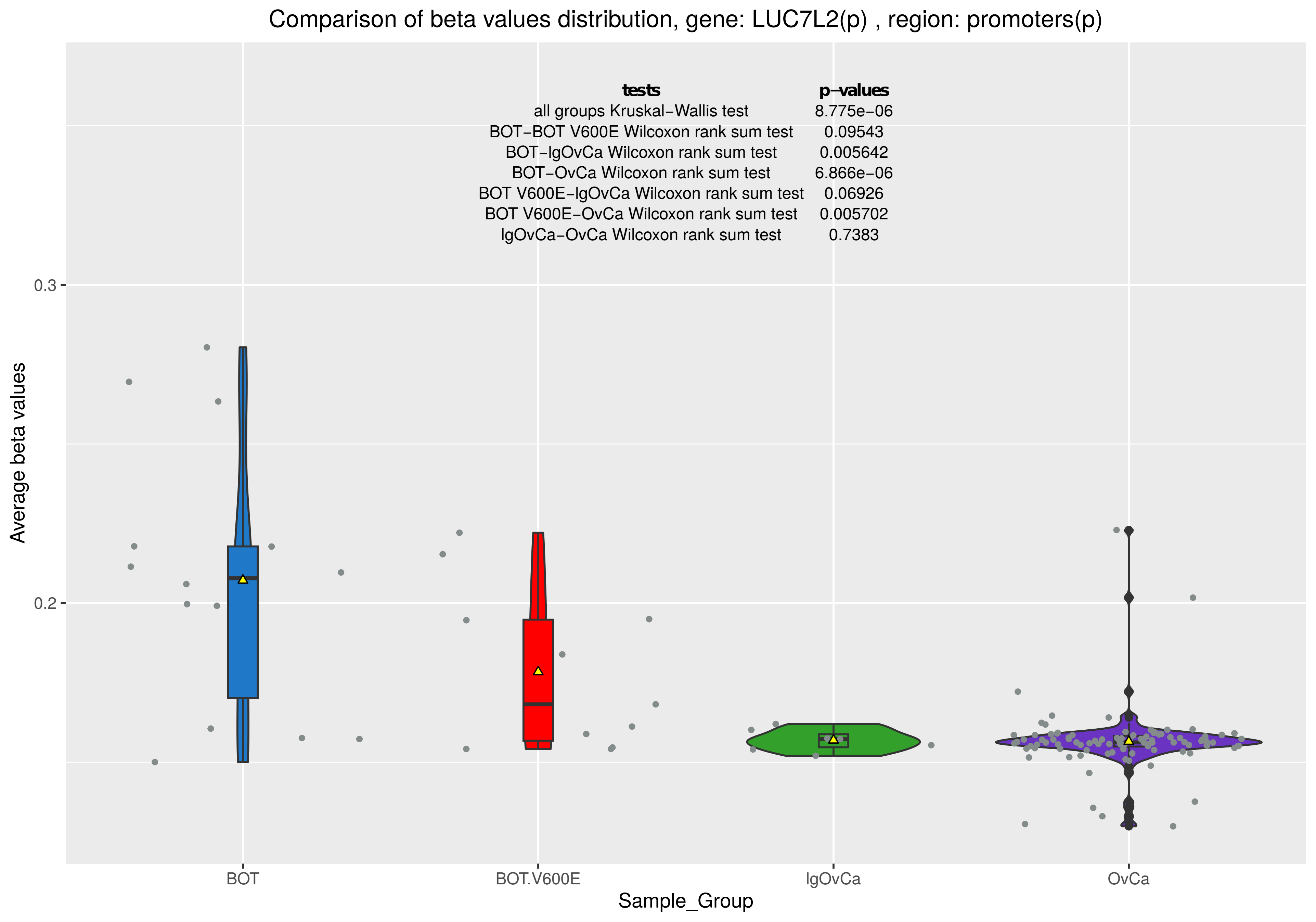
Comparison of beta values distribution, gene: LUC7L2(p) , region: promoters(p)
tests
p−values
all groups Kruskal−Wallis test
8.775e−06
BOT−BOT V600E Wilcoxon rank sum test
0.09543
BOT−lgOvCa Wilcoxon rank sum test
0.005642
BOT−OvCa Wilcoxon rank sum test
6.866e−06
BOT V600E−lgOvCa Wilcoxon rank sum test
0.06926
BOT V600E−OvCa Wilcoxon rank sum test
0.005702
lgOvCa−OvCa Wilcoxon rank sum test
0.7383
Average beta values
0.3
0.2
BOT
BOT.V600E
lgOvCa
OvCa
Sample_Group

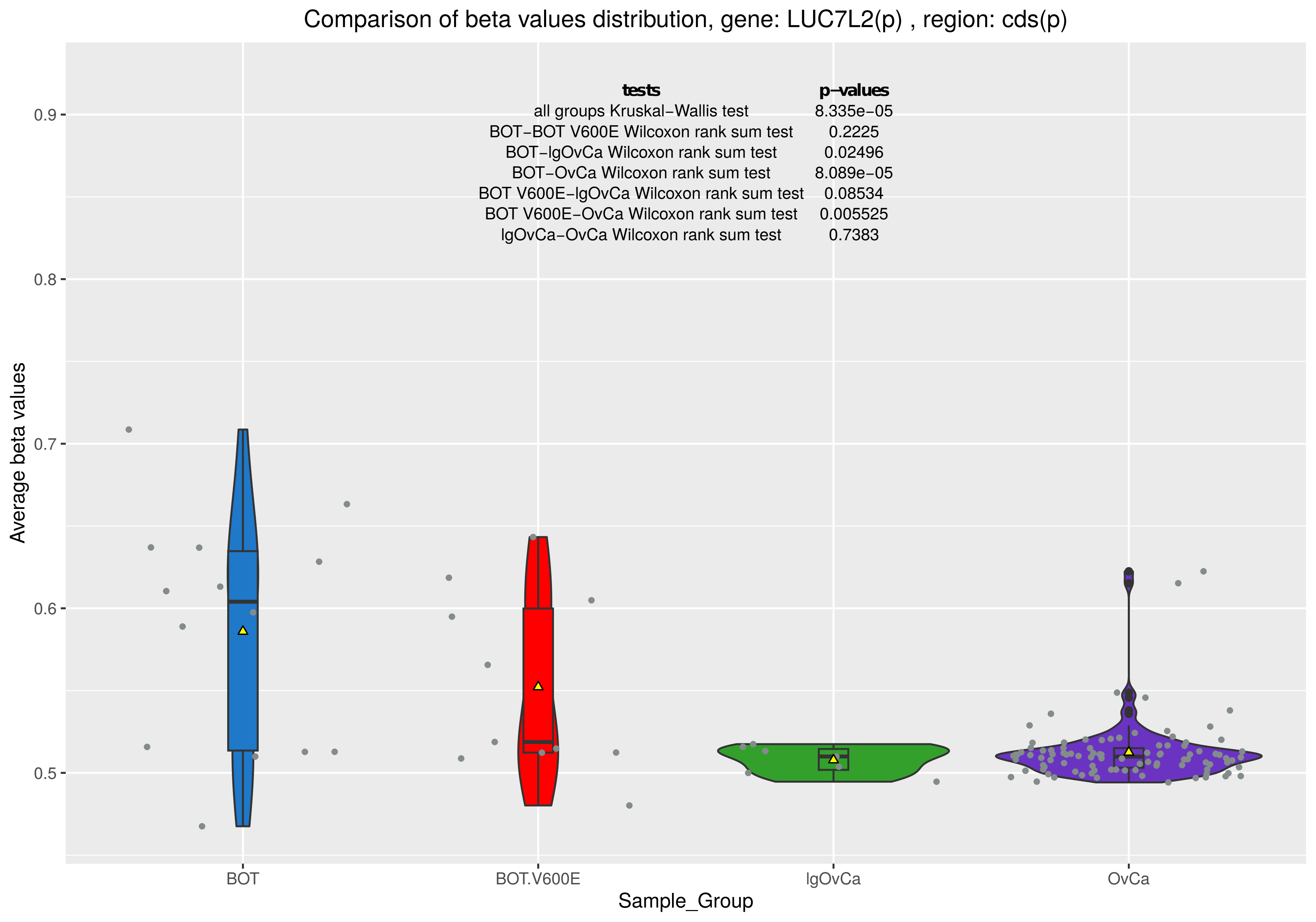

Comparison of beta values distribution, gene: LUC7L2(p) , region: cds(p)
tests
p−values
all groups Kruskal−Wallis test
8.335e−05
BOT−BOT V600E Wilcoxon rank sum test
0.2225
BOT−lgOvCa Wilcoxon rank sum test
0.02496
BOT−OvCa Wilcoxon rank sum test
8.089e−05
BOT V600E−lgOvCa Wilcoxon rank sum test
0.08534
BOT V600E−OvCa Wilcoxon rank sum test
0.005525
lgOvCa−OvCa Wilcoxon rank sum test
0.7383
Average beta values
0.9
0.8
0.7
0.6
0.5
BOT
BOT.V600E
lgOvCa
OvCa
Sample_Group

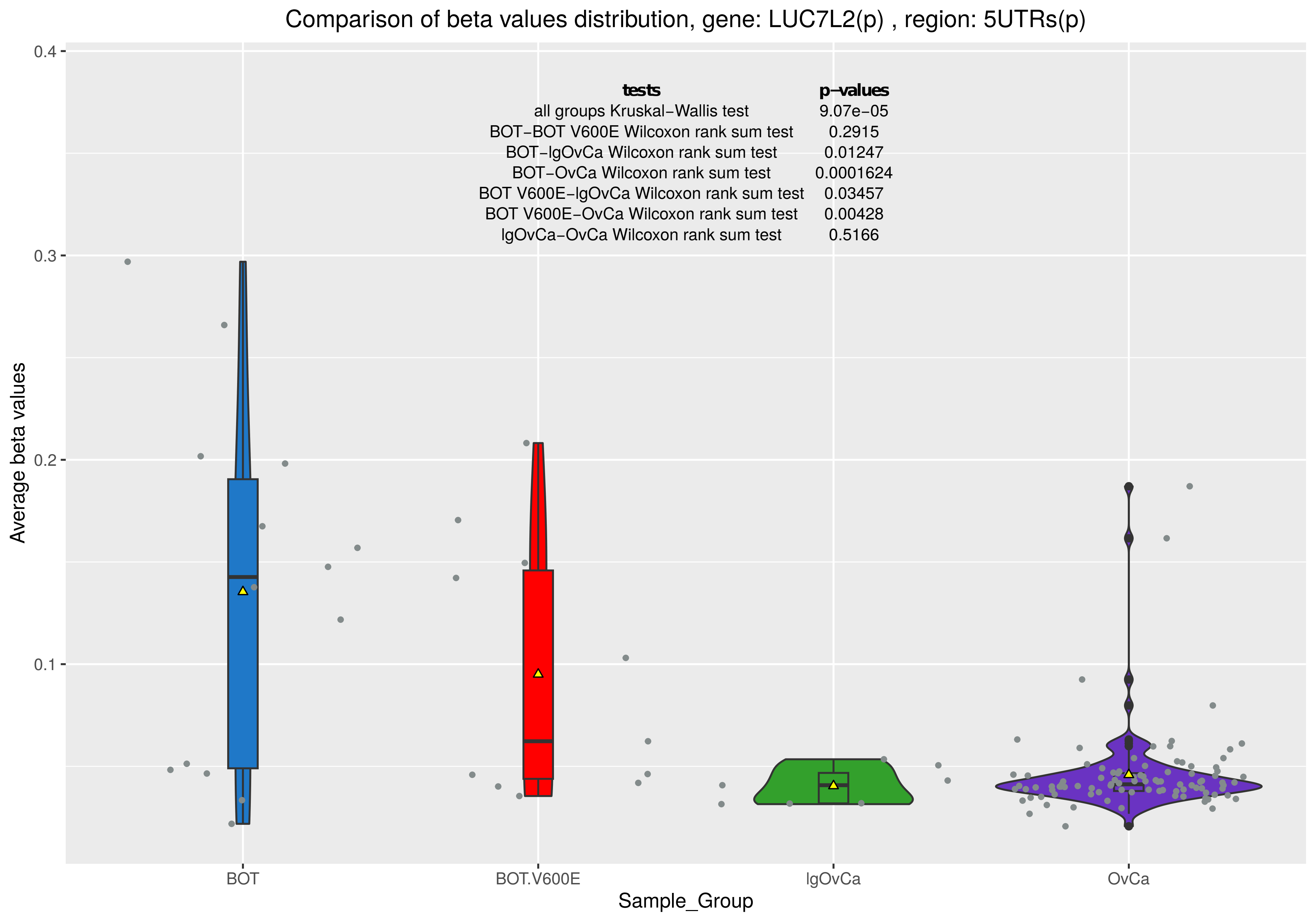
Comparison of beta values distribution, gene: LUC7L2(p) , region: 5UTRs(p)
tests
p−values
all groups Kruskal−Wallis test
9.07e−05
BOT−BOT V600E Wilcoxon rank sum test
0.2915
BOT−lgOvCa Wilcoxon rank sum test
0.01247
BOT−OvCa Wilcoxon rank sum test
0.0001624
BOT V600E−lgOvCa Wilcoxon rank sum test
0.03457
BOT V600E−OvCa Wilcoxon rank sum test
0.00428
lgOvCa−OvCa Wilcoxon rank sum test
0.5166
Average beta values
0.4
0.3
0.2
0.1
BOT
BOT.V600E
lgOvCa
OvCa
Sample_Group

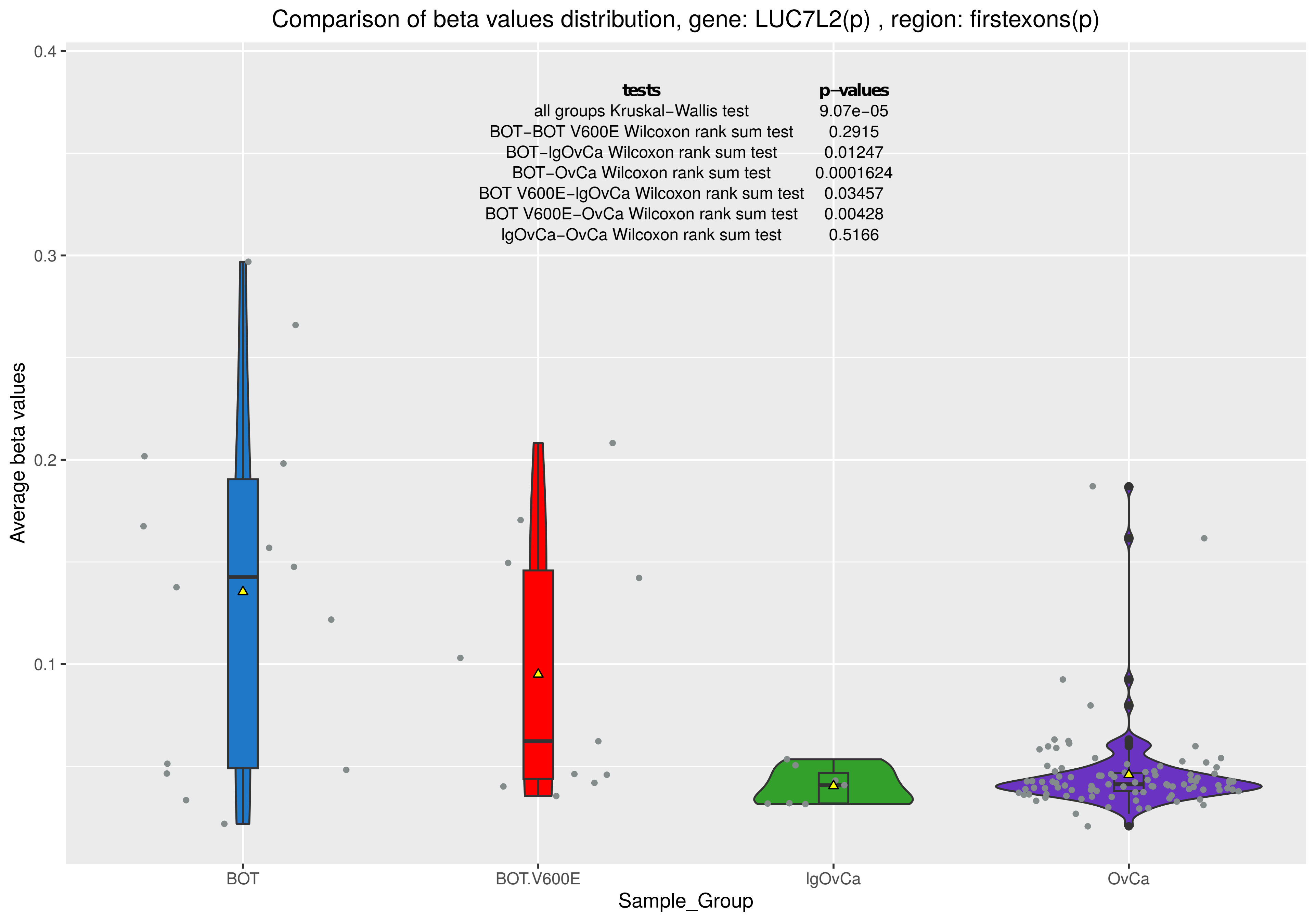
Comparison of beta values distribution, gene: LUC7L2(p) , region: firstexons(p)
tests
p−values
all groups Kruskal−Wallis test
9.07e−05
BOT−BOT V600E Wilcoxon rank sum test
0.2915
BOT−lgOvCa Wilcoxon rank sum test
0.01247
BOT−OvCa Wilcoxon rank sum test
0.0001624
BOT V600E−lgOvCa Wilcoxon rank sum test
0.03457
BOT V600E−OvCa Wilcoxon rank sum test
0.00428
lgOvCa−OvCa Wilcoxon rank sum test
0.5166
Average beta values
0.4
0.3
0.2
0.1
BOT
BOT.V600E
lgOvCa
OvCa
Sample_Group

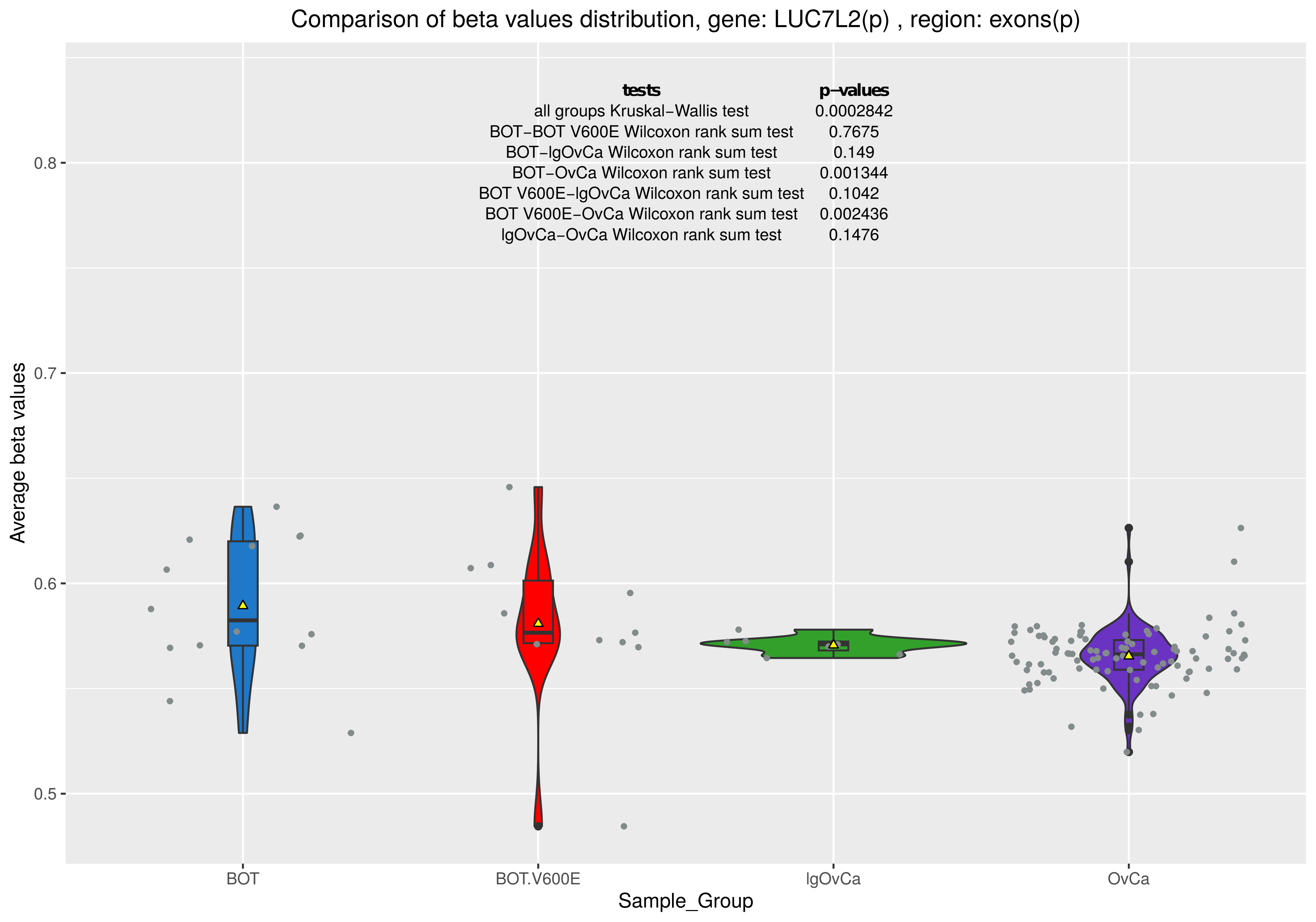
Comparison of beta values distribution, gene: LUC7L2(p) , region: exons(p)
tests
p−values
all groups Kruskal−Wallis test
0.0002842
BOT−BOT V600E Wilcoxon rank sum test
0.7675
BOT−lgOvCa Wilcoxon rank sum test
0.149
BOT−OvCa Wilcoxon rank sum test
0.001344
BOT V600E−lgOvCa Wilcoxon rank sum test
0.1042
BOT V600E−OvCa Wilcoxon rank sum test
0.002436
lgOvCa−OvCa Wilcoxon rank sum test
0.1476
Average beta values
0.8
0.7
0.6
0.5
BOT
BOT.V600E
lgOvCa
OvCa
Sample_Group

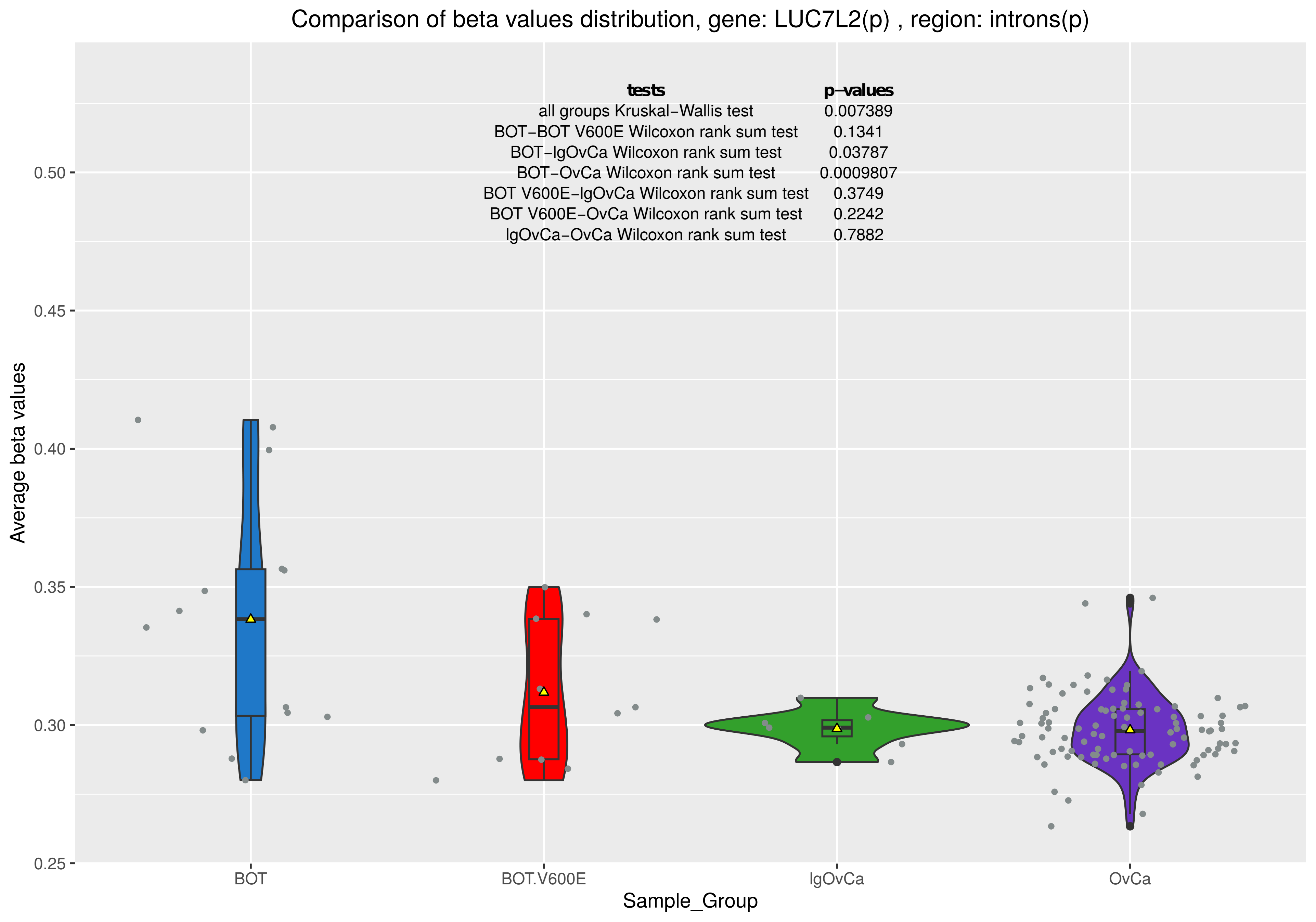

Comparison of beta values distribution, gene: LUC7L2(p) , region: introns(p)
tests
p−values
all groups Kruskal−Wallis test
0.007389
BOT−BOT V600E Wilcoxon rank sum test
0.1341
BOT−IgOvCa Wilcoxon rank sum test
0.03787
BOT−OvCa Wilcoxon rank sum test
0.0009807
BOT V600E−IgOvCa Wilcoxon rank sum test
0.3749
BOT V600E−OvCa Wilcoxon rank sum test
0.2242
IgOvCa−OvCa Wilcoxon rank sum test
0.7882
Average beta values
0.50
0.45
0.40
0.35
0.30
0.25
BOT
BOT.V600E
IgOvCa
OvCa
Sample_Group

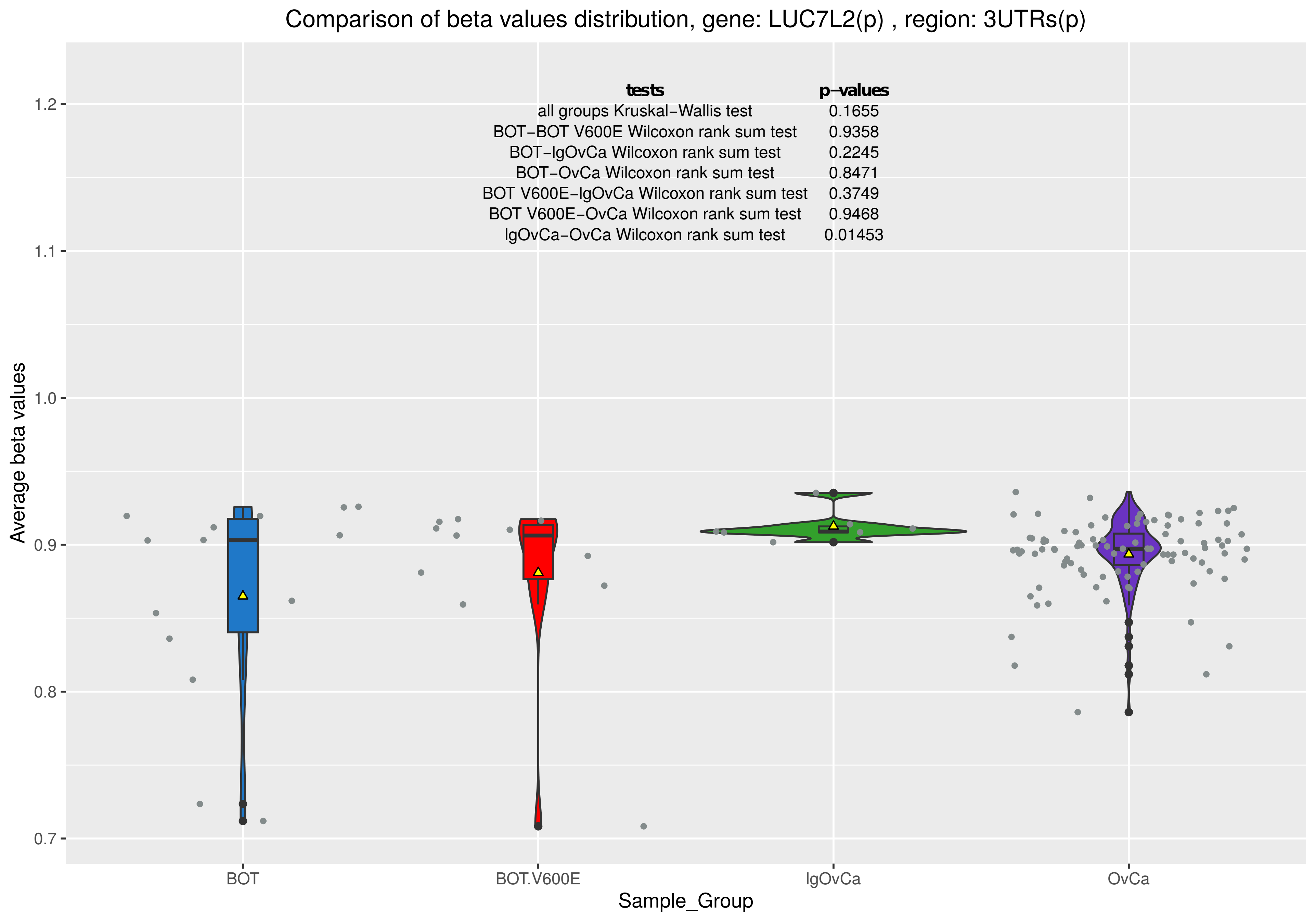
Comparison of beta values distribution, gene: LUC7L2(p) , region: 3UTRs(p)
tests
p−values
all groups Kruskal−Wallis test
0.1655
BOT−BOT V600E Wilcoxon rank sum test
0.9358
BOT−lgOvCa Wilcoxon rank sum test
0.2245
BOT−OvCa Wilcoxon rank sum test
0.8471
BOT V600E−lgOvCa Wilcoxon rank sum test
0.3749
BOT V600E−OvCa Wilcoxon rank sum test
0.9468
lgOvCa−OvCa Wilcoxon rank sum test
0.01453
Average beta values
1.2
1.1
1.0
0.9
0.8
0.7
BOT
BOT.V600E
lgOvCa
OvCa
Sample_Group

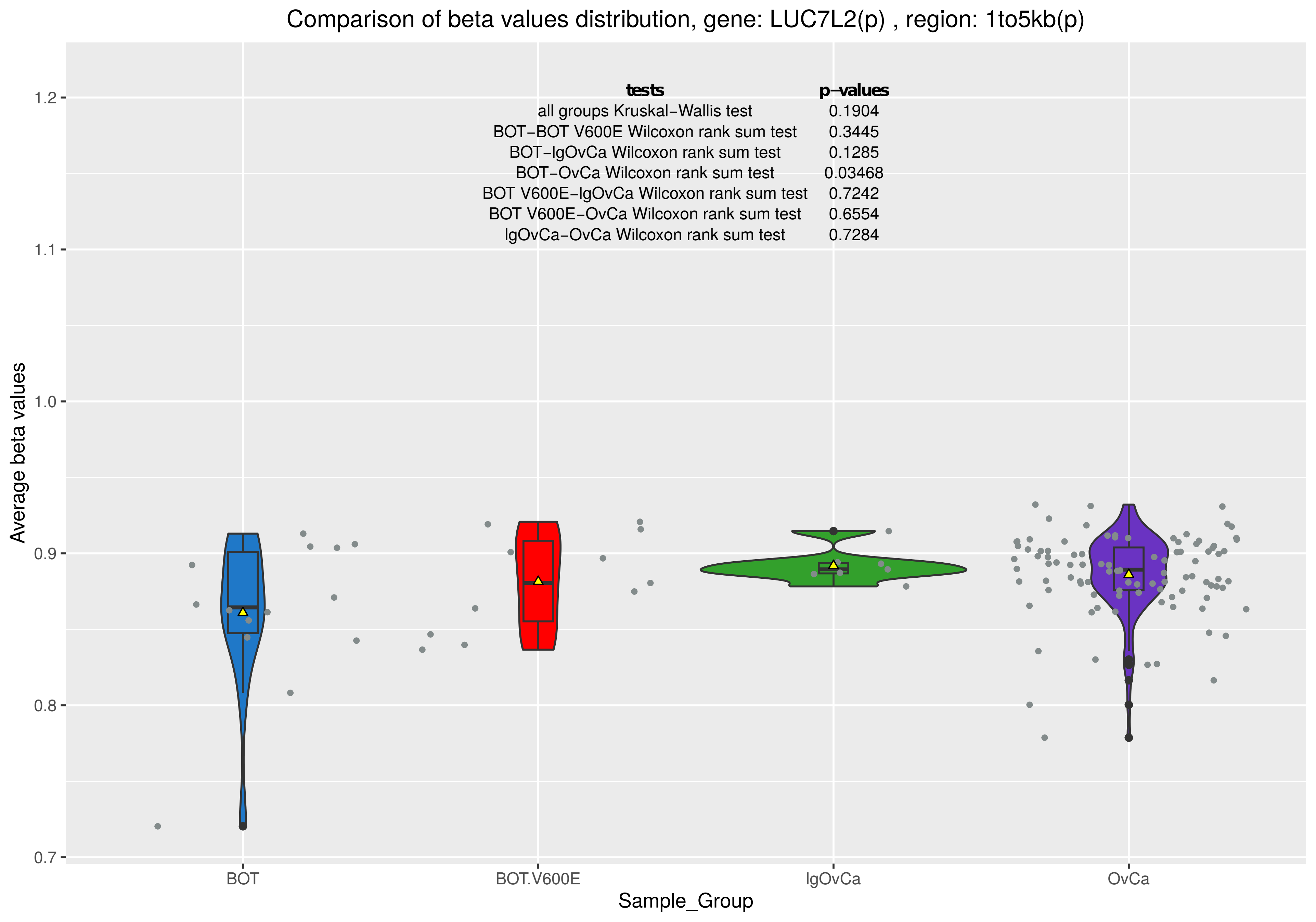
Comparison of beta values distribution, gene: LUC7L2(p) , region: 1to5kb(p)
tests p−values
all groups Kruskal−Wallis test 0.1904
BOT−BOT V600E Wilcoxon rank sum test 0.3445
BOT−lgOvCa Wilcoxon rank sum test 0.1285
BOT−OvCa Wilcoxon rank sum test 0.03468
BOT V600E−lgOvCa Wilcoxon rank sum test 0.7242
BOT V600E−OvCa Wilcoxon rank sum test 0.6554
lgOvCa−OvCa Wilcoxon rank sum test 0.7284
Average beta values
1.2
1.1
1.0
0.9
0.8
0.7
BOT
BOT.V600E
lgOvCa
OvCa
Sample_Group

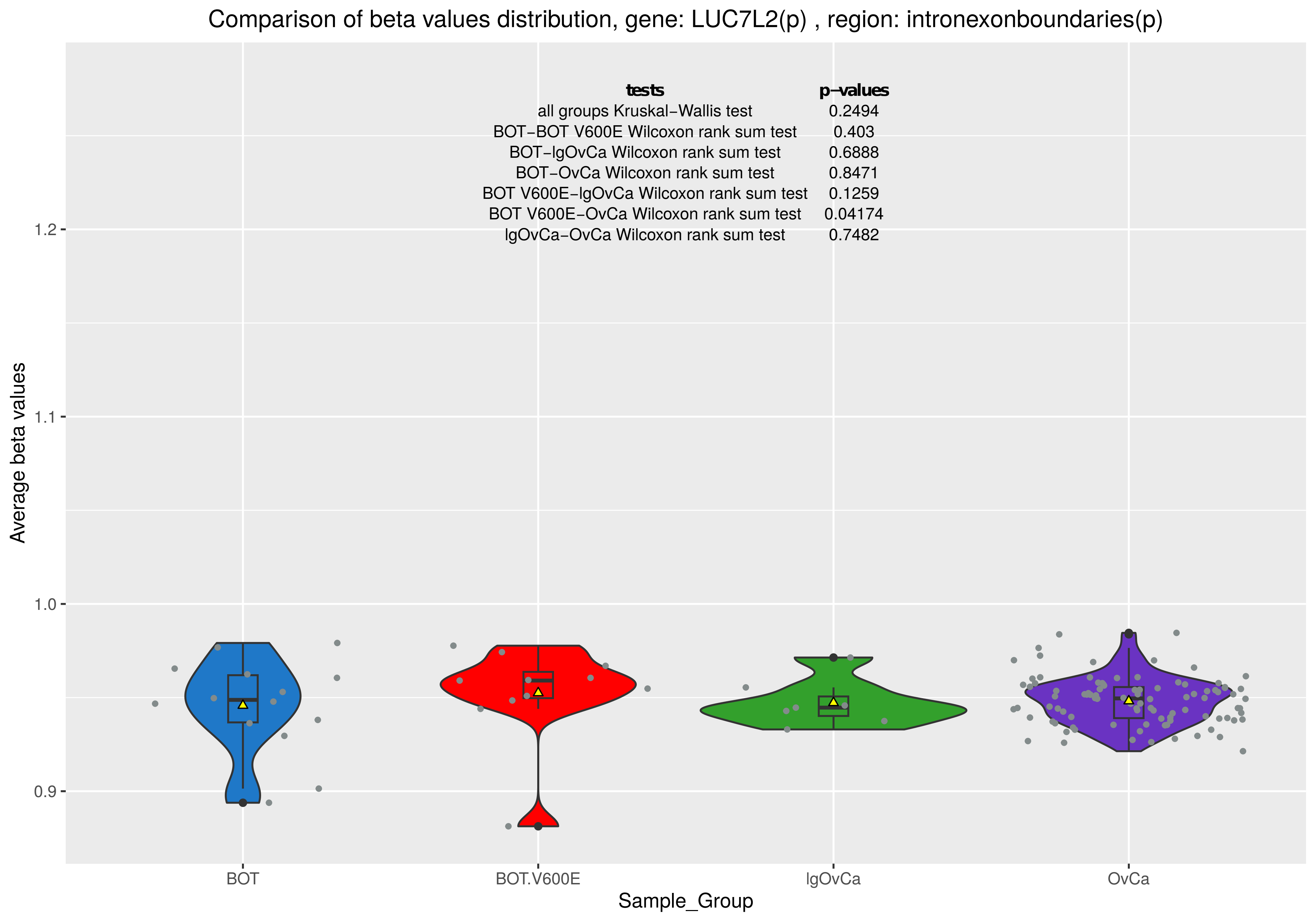

Comparison of beta values distribution, gene: LUC7L2(p) , region: intronexonboundaries(p)
tests
p−values
all groups Kruskal−Wallis test
0.2494
BOT−BOT V600E Wilcoxon rank sum test
0.403
BOT−lgOvCa Wilcoxon rank sum test
0.6888
BOT−OvCa Wilcoxon rank sum test
0.8471
BOT V600E−lgOvCa Wilcoxon rank sum test
0.1259
BOT V600E−OvCa Wilcoxon rank sum test
0.04174
lgOvCa−OvCa Wilcoxon rank sum test
0.7482
Average beta values
1.2
1.1
1.0
0.9
BOT
BOT.V600E
lgOvCa
OvCa
Sample_Group

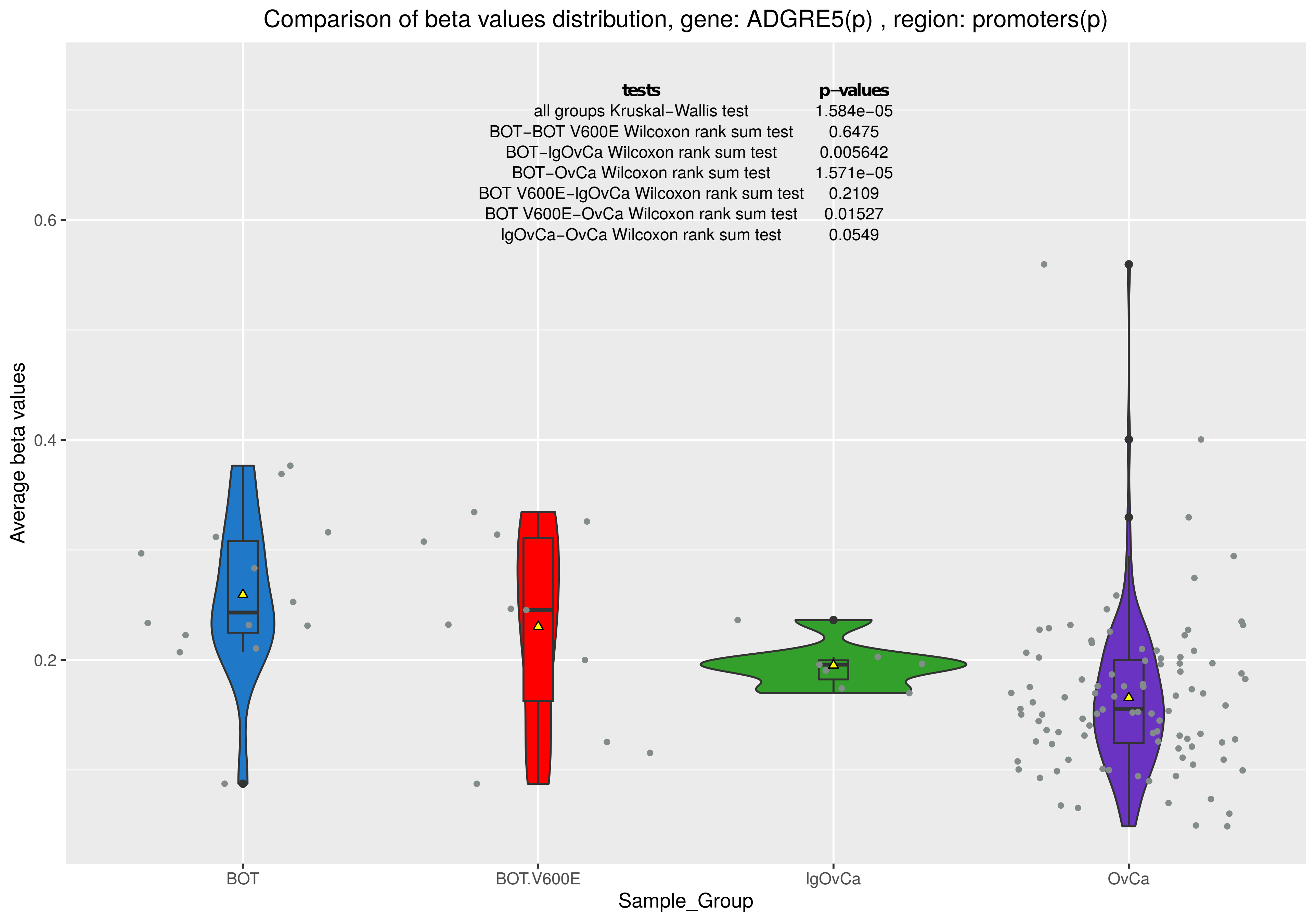

Comparison of beta values distribution, gene: ADGRE5(p) , region: promoters(p)
tests
p−values
all groups Kruskal−Wallis test
1.584e−05
BOT−BOT V600E Wilcoxon rank sum test
0.6475
BOT−lgOvCa Wilcoxon rank sum test
0.005642
BOT−OvCa Wilcoxon rank sum test
1.571e−05
BOT V600E−lgOvCa Wilcoxon rank sum test
0.2109
BOT V600E−OvCa Wilcoxon rank sum test
0.01527
lgOvCa−OvCa Wilcoxon rank sum test
0.0549
Average beta values
0.6
0.4
0.2
BOT
BOT.V600E
lgOvCa
OvCa
Sample_Group

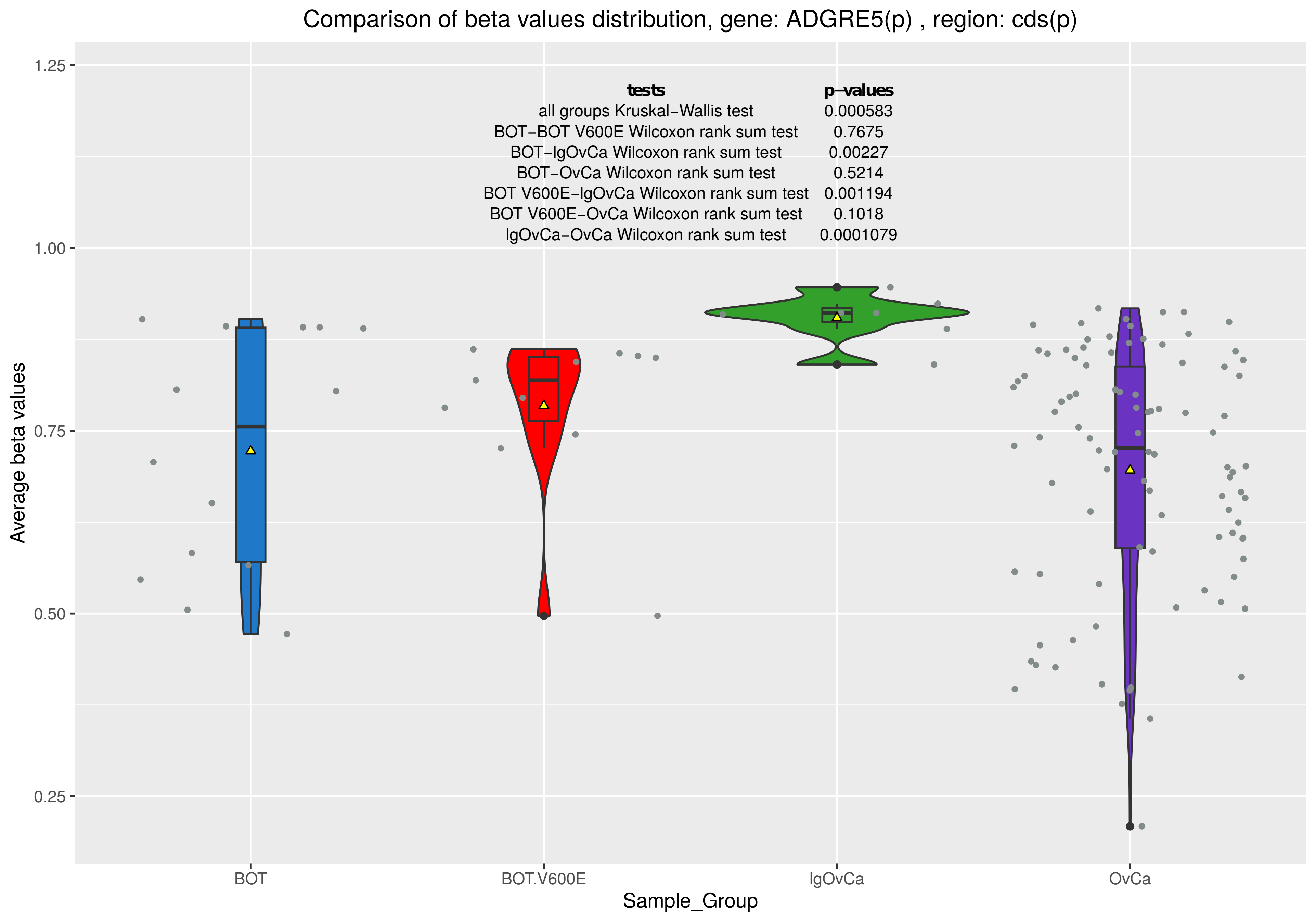
Comparison of beta values distribution, gene: ADGRE5(p) , region: cds(p)
tests
p−values
all groups Kruskal−Wallis test 0.000583
BOT−BOT V600E Wilcoxon rank sum test 0.7675
BOT−IgOvCa Wilcoxon rank sum test 0.00227
BOT−OvCa Wilcoxon rank sum test 0.5214
BOT V600E−IgOvCa Wilcoxon rank sum test 0.001194
BOT V600E−OvCa Wilcoxon rank sum test 0.1018
IgOvCa−OvCa Wilcoxon rank sum test 0.0001079
1.25
1.00
0.75
0.50
0.25
Average beta values
BOT
BOT.V600E
IgOvCa
OvCa
Sample_Group

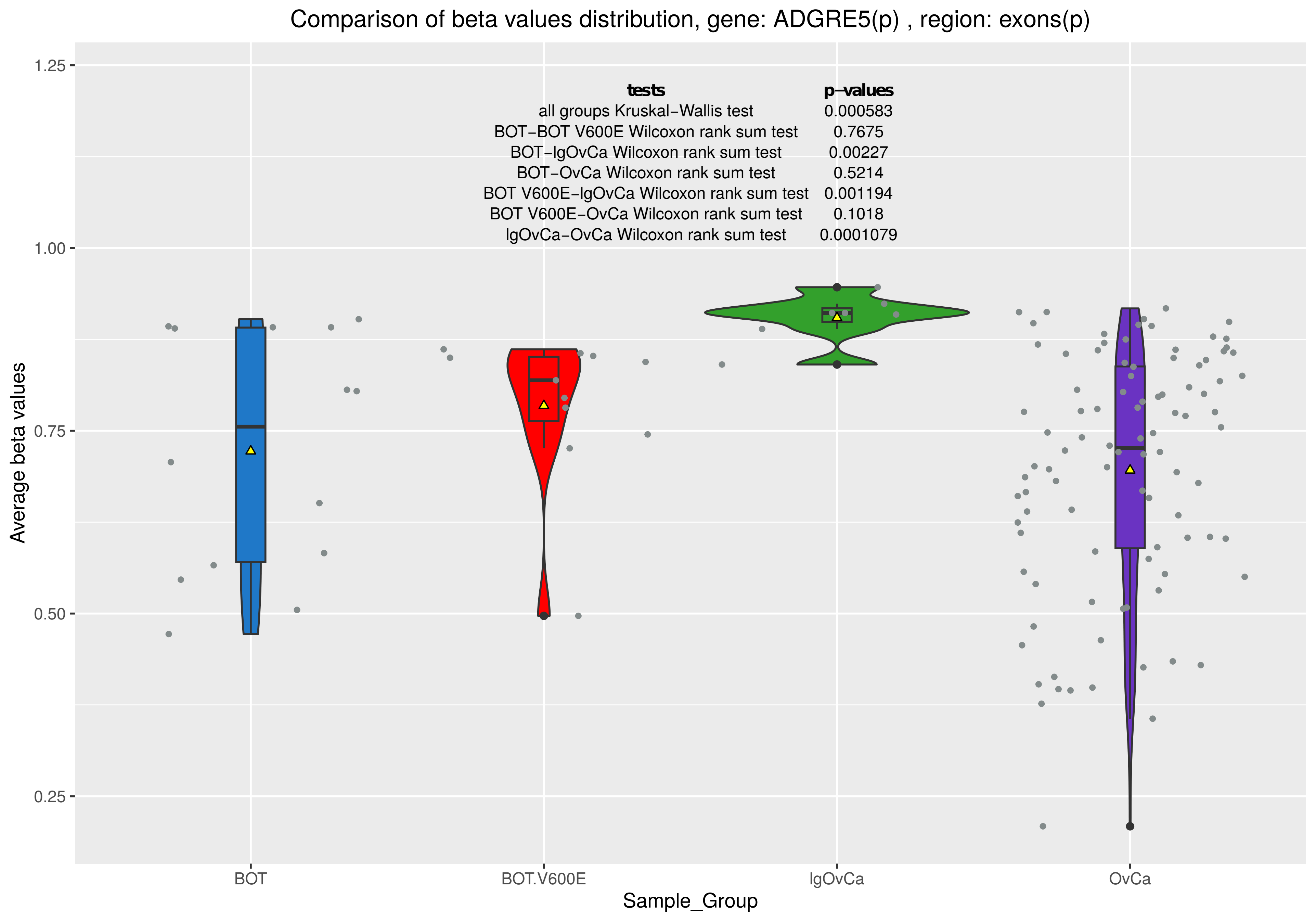
Comparison of beta values distribution, gene: ADGRE5(p) , region: exons(p)
tests
p−values
all groups Kruskal−Wallis test
0.000583
BOT−BOT V600E Wilcoxon rank sum test
0.7675
BOT−IgOvCa Wilcoxon rank sum test
0.00227
BOT−OvCa Wilcoxon rank sum test
0.5214
BOT V600E−IgOvCa Wilcoxon rank sum test
0.001194
BOT V600E−OvCa Wilcoxon rank sum test
0.1018
IgOvCa−OvCa Wilcoxon rank sum test
0.0001079
Average beta values
1.25
1.00
0.75
0.50
0.25
BOT
BOT.V600E
IgOvCa
OvCa
Sample_Group

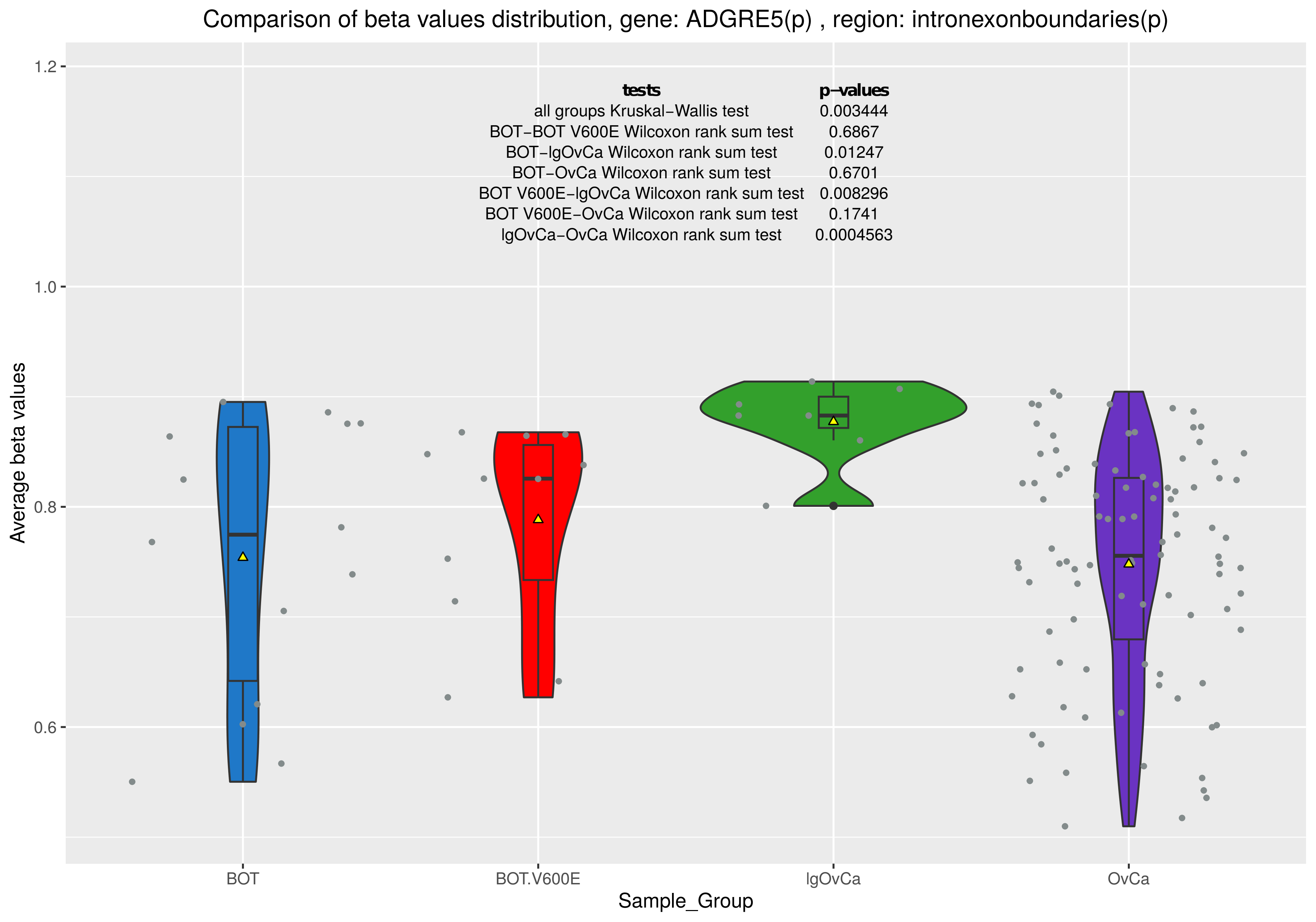

Comparison of beta values distribution, gene: ADGRE5(p) , region: intronexonboundaries(p)
tests
p−values
all groups Kruskal−Wallis test
0.003444
BOT−BOT V600E Wilcoxon rank sum test
0.6867
BOT−lgOvCa Wilcoxon rank sum test
0.01247
BOT−OvCa Wilcoxon rank sum test
0.6701
BOT V600E−lgOvCa Wilcoxon rank sum test
0.008296
BOT V600E−OvCa Wilcoxon rank sum test
0.1741
lgOvCa−OvCa Wilcoxon rank sum test
0.0004563
Average beta values
1.2
1.0
0.8
0.6
BOT
BOT.V600E
lgOvCa
OvCa
Sample_Group

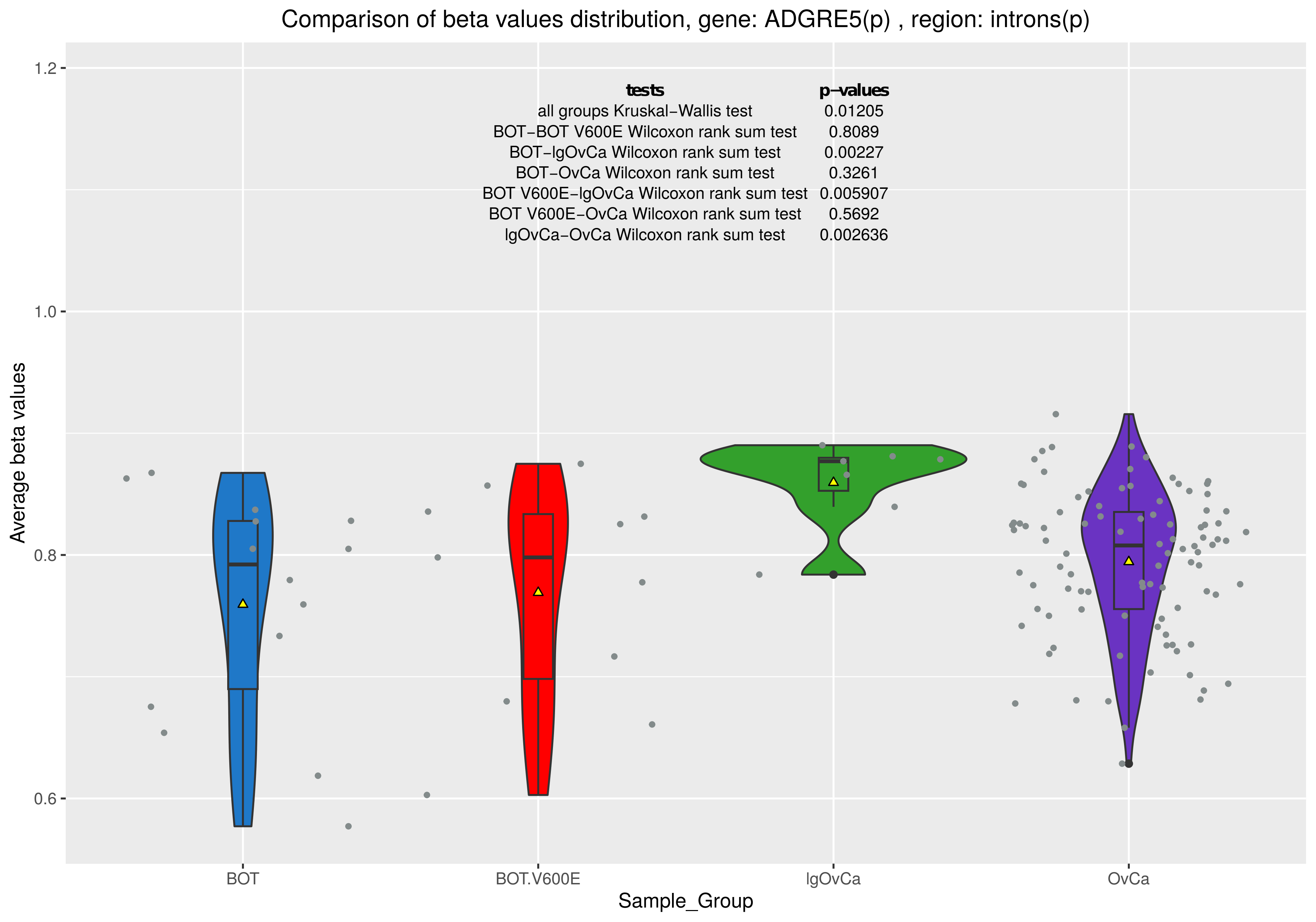
Comparison of beta values distribution, gene: ADGRE5(p) , region: introns(p)
tests
p−values
all groups Kruskal−Wallis test
0.01205
BOT−BOT V600E Wilcoxon rank sum test
0.8089
BOT−lgOvCa Wilcoxon rank sum test
0.00227
BOT−OvCa Wilcoxon rank sum test
0.3261
BOT V600E−lgOvCa Wilcoxon rank sum test
0.005907
BOT V600E−OvCa Wilcoxon rank sum test
0.5692
lgOvCa−OvCa Wilcoxon rank sum test
0.002636
Average beta values
1.2
1.0
0.8
0.6
BOT
BOT.V600E
lgOvCa
OvCa
Sample_Group

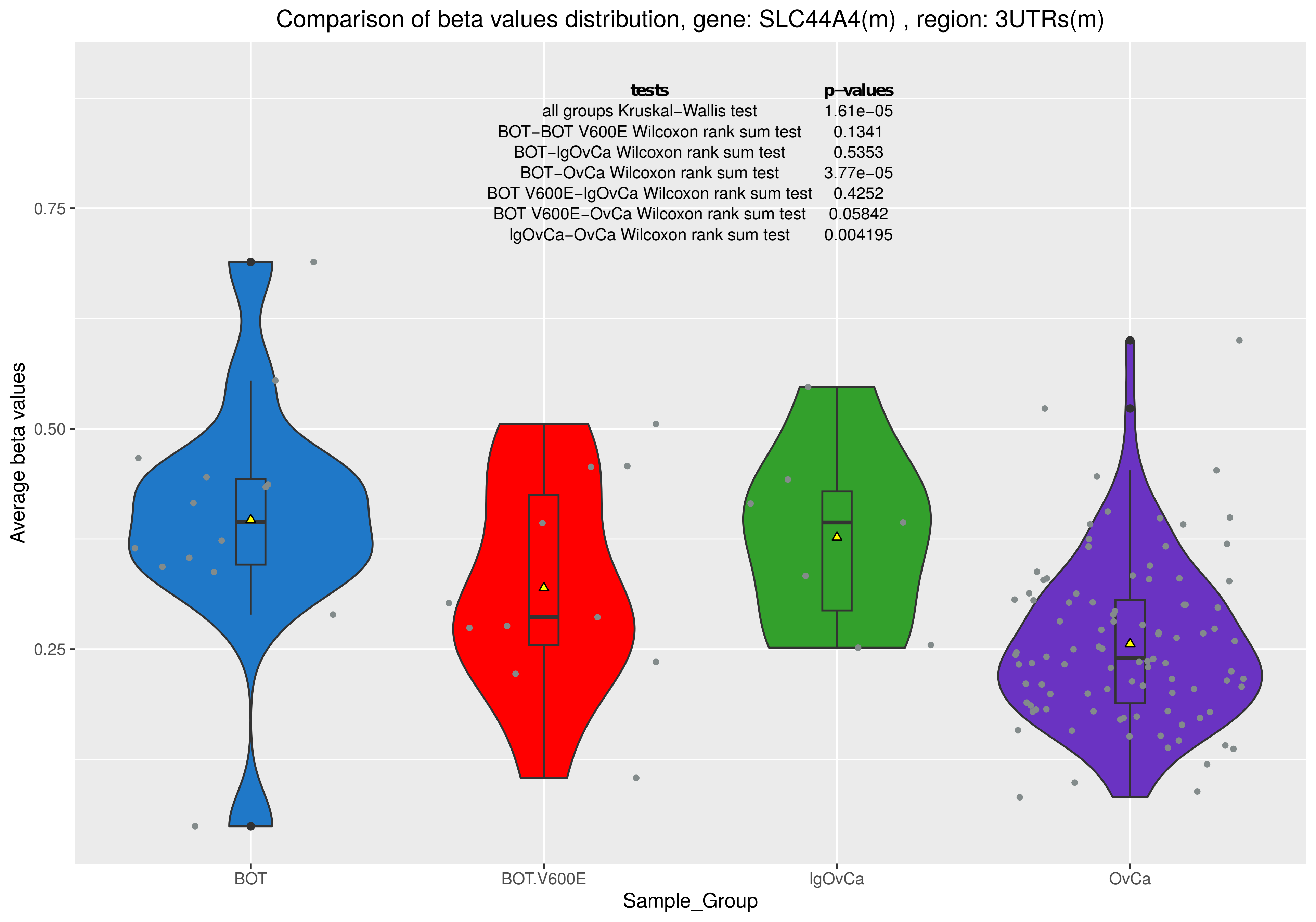

Comparison of beta values distribution, gene: SLC44A4(m) , region: 3UTRs(m)
tests
p−values
all groups Kruskal−Wallis test
1.61e−05
BOT−BOT V600E Wilcoxon rank sum test
0.1341
BOT−lgOvCa Wilcoxon rank sum test
0.5353
BOT−OvCa Wilcoxon rank sum test
3.77e−05
BOT V600E−lgOvCa Wilcoxon rank sum test
0.4252
BOT V600E−OvCa Wilcoxon rank sum test
0.05842
lgOvCa−OvCa Wilcoxon rank sum test
0.004195
0.75
0.50
0.25
Average beta values
BOT
BOT.V600E
lgOvCa
OvCa
Sample_Group

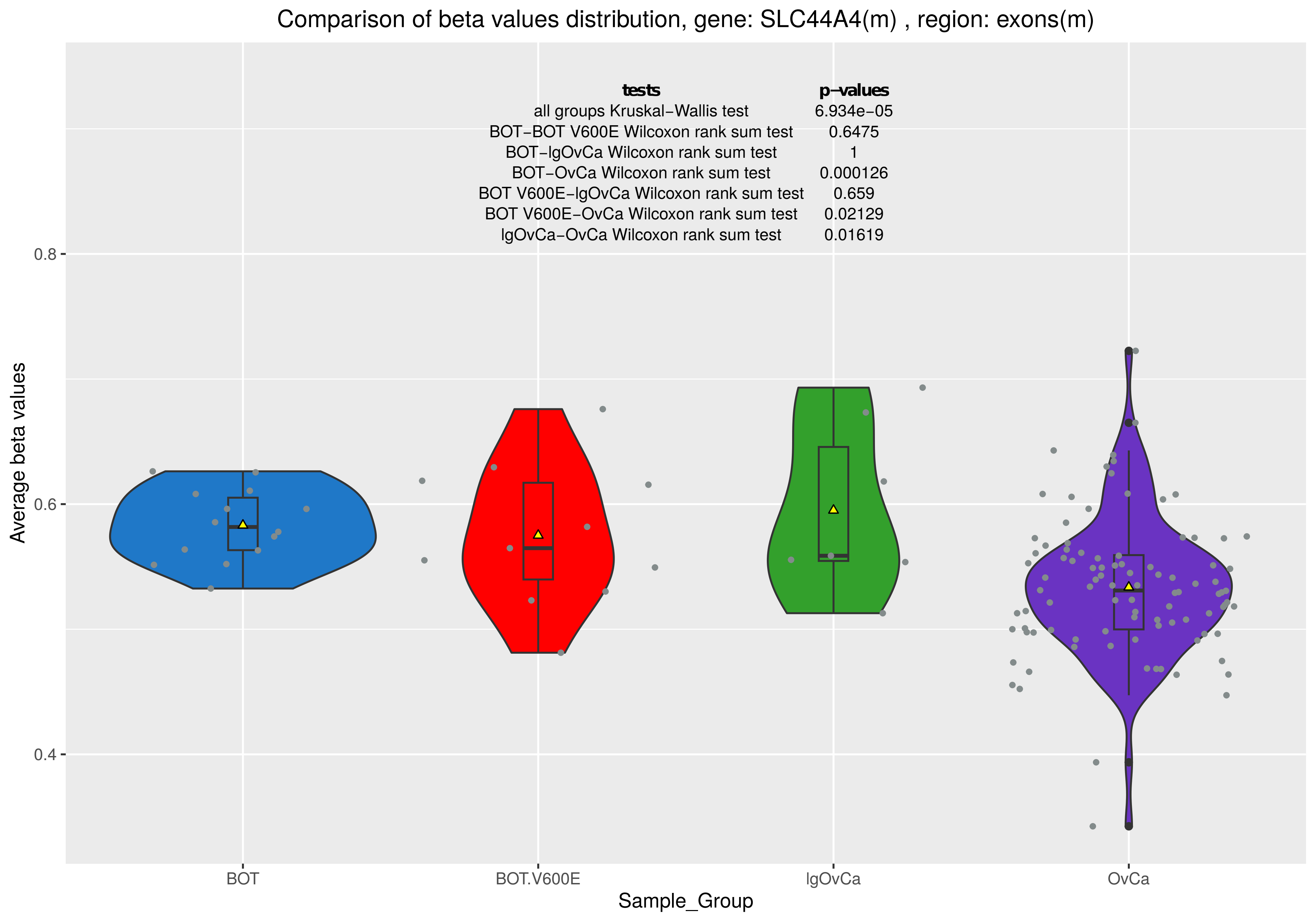

Comparison of beta values distribution, gene: SLC44A4(m) , region: exons(m)
tests
p−values
all groups Kruskal−Wallis test
6.934e−05
BOT−BOT V600E Wilcoxon rank sum test
0.6475
BOT−lgOvCa Wilcoxon rank sum test
1
BOT−OvCa Wilcoxon rank sum test
0.000126
BOT V600E−lgOvCa Wilcoxon rank sum test
0.659
BOT V600E−OvCa Wilcoxon rank sum test
0.02129
lgOvCa−OvCa Wilcoxon rank sum test
0.01619
Average beta values
0.8
0.6
0.4
BOT
BOT.V600E
lgOvCa
OvCa
Sample_Group

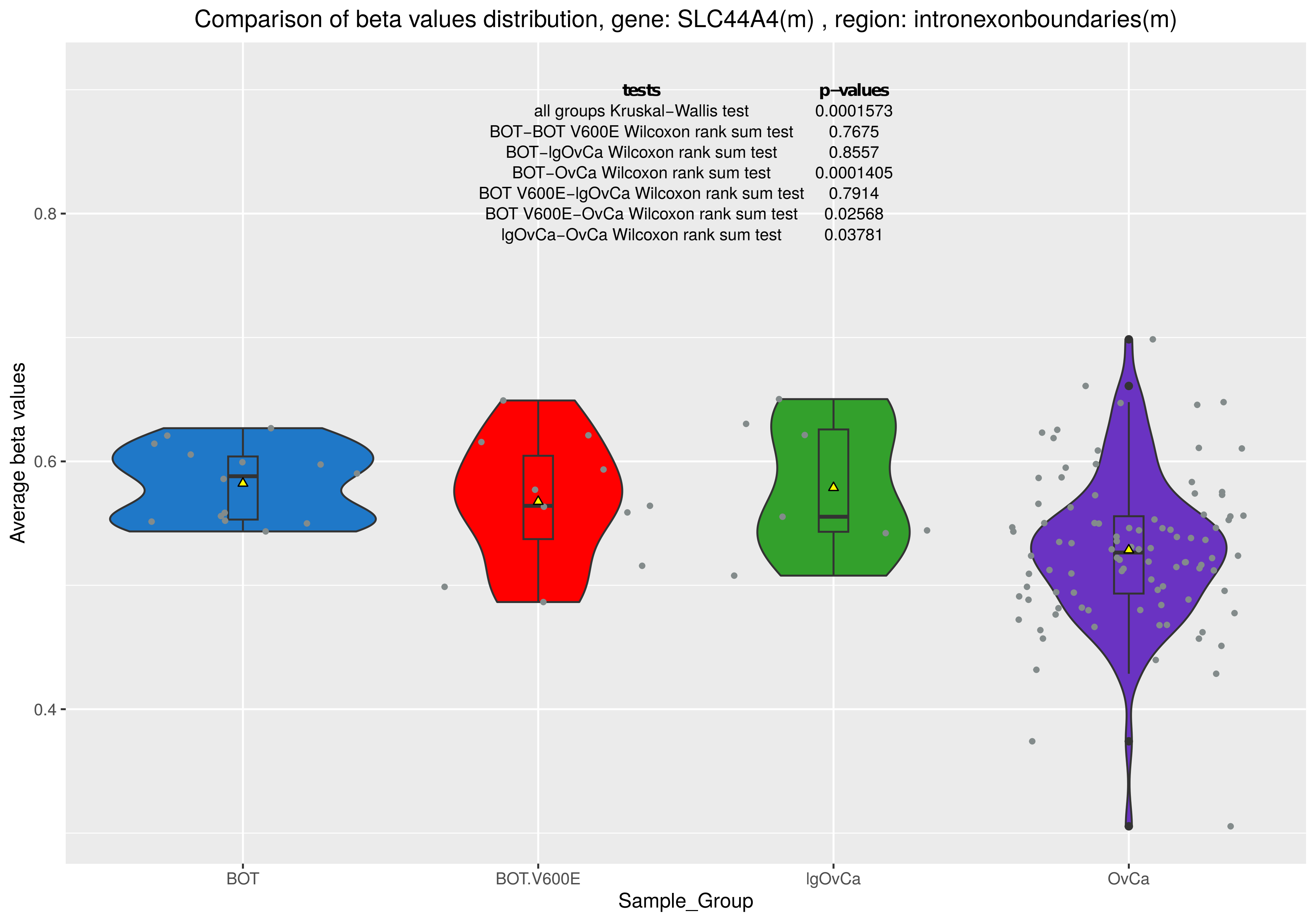

Comparison of beta values distribution, gene: SLC44A4(m) , region: intronexonboundaries(m)
tests
p−values
all groups Kruskal−Wallis test
0.0001573
BOT−BOT V600E Wilcoxon rank sum test
0.7675
BOT−lgOvCa Wilcoxon rank sum test
0.8557
BOT−OvCa Wilcoxon rank sum test
0.0001405
BOT V600E−lgOvCa Wilcoxon rank sum test
0.7914
BOT V600E−OvCa Wilcoxon rank sum test
0.02568
lgOvCa−OvCa Wilcoxon rank sum test
0.03781
Average beta values
0.8
0.6
0.4
BOT
BOT.V600E
lgOvCa
OvCa
Sample_Group

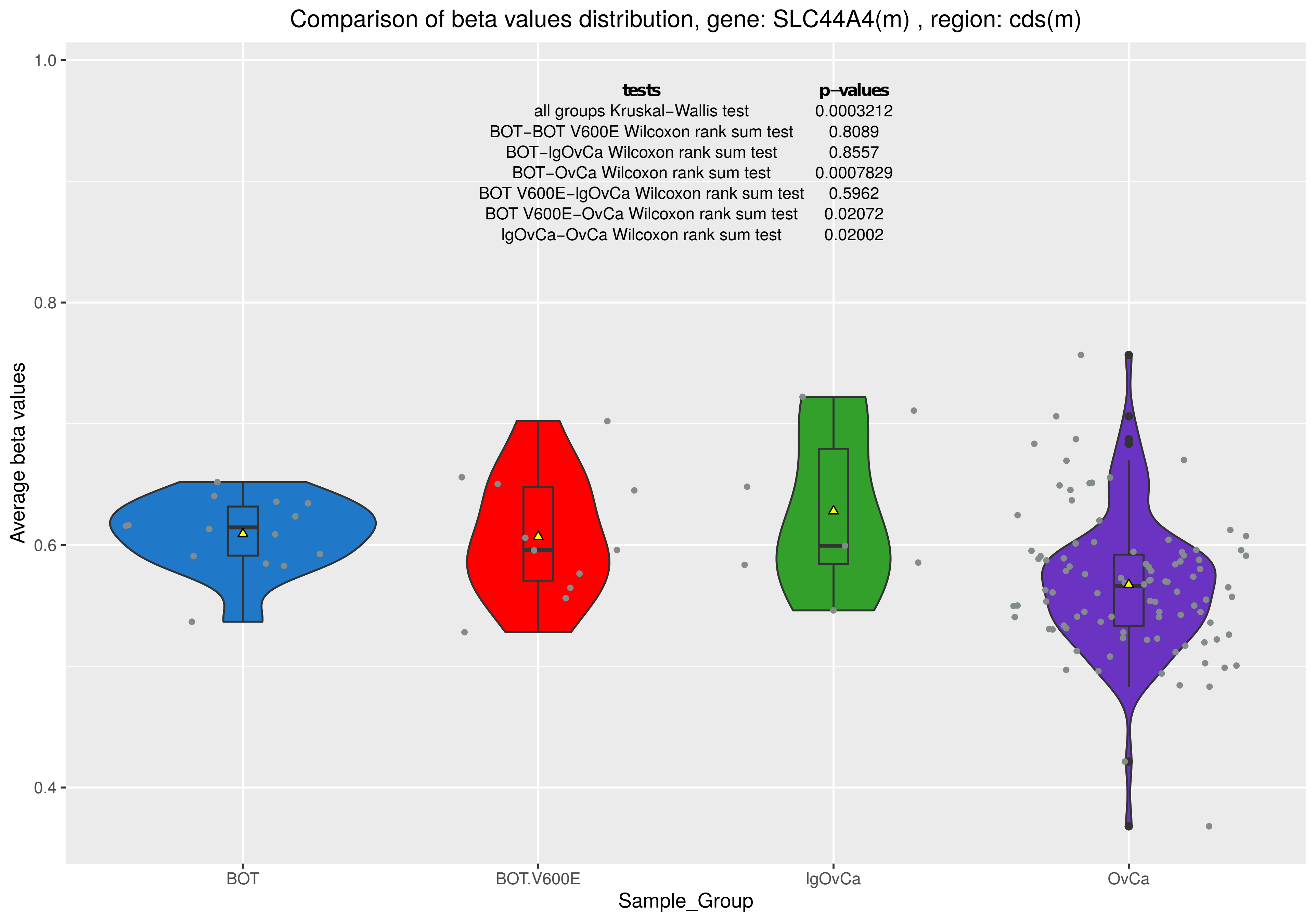

Comparison of beta values distribution, gene: SLC44A4(m) , region: cds(m)
tests
p−values
all groups Kruskal−Wallis test
0.0003212
BOT−BOT V600E Wilcoxon rank sum test
0.8089
BOT−lgOvCa Wilcoxon rank sum test
0.8557
BOT−OvCa Wilcoxon rank sum test
0.0007829
BOT V600E−lgOvCa Wilcoxon rank sum test
0.5962
BOT V600E−OvCa Wilcoxon rank sum test
0.02072
lgOvCa−OvCa Wilcoxon rank sum test
0.02002
Average beta values
1.0
0.8
0.6
0.4
BOT
BOT.V600E
lgOvCa
OvCa
Sample_Group

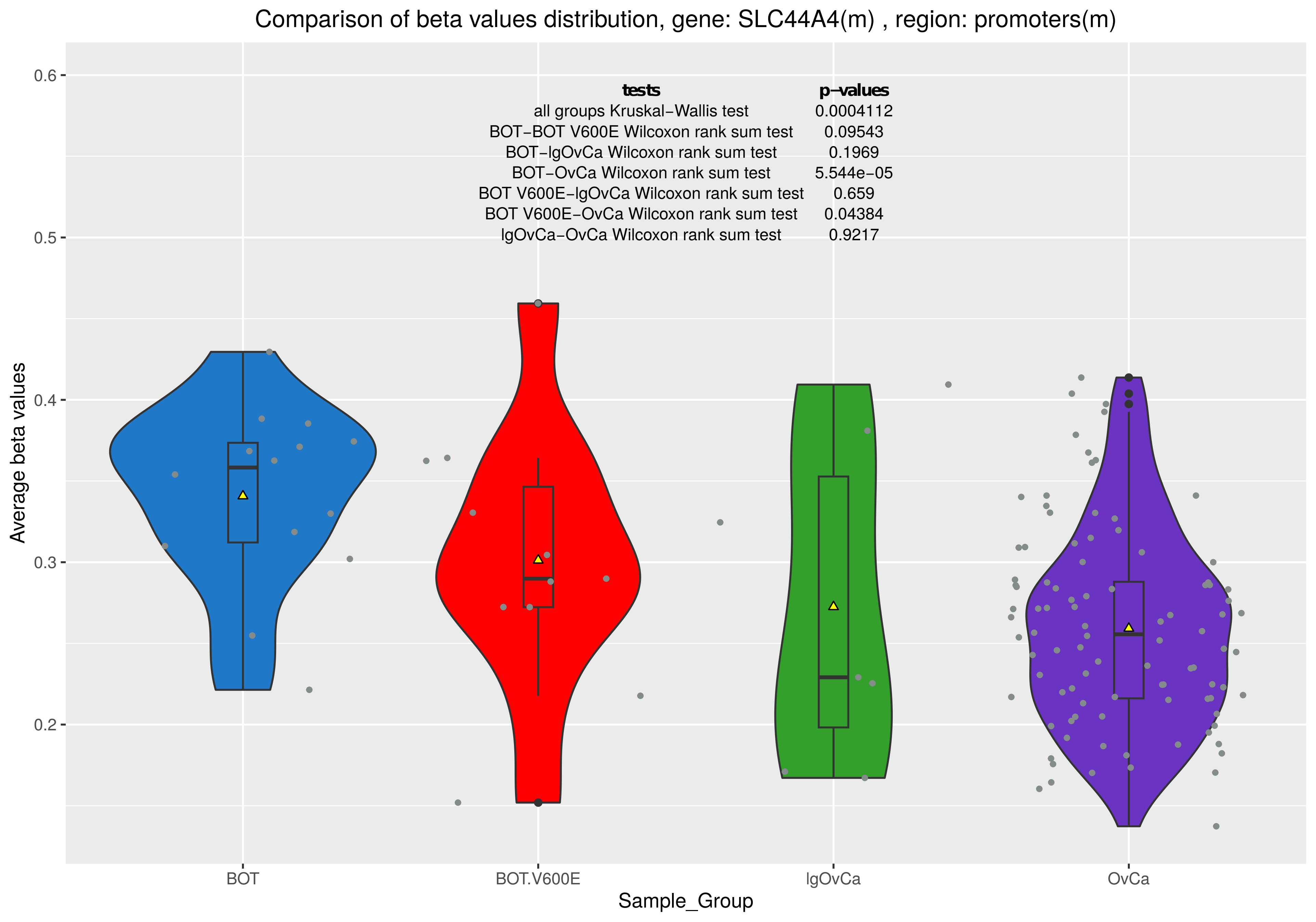
Comparison of beta values distribution, gene: SLC44A4(m) , region: promoters(m)
tests
p−values
all groups Kruskal−Wallis test
0.0004112
BOT−BOT V600E Wilcoxon rank sum test
0.09543
BOT−lgOvCa Wilcoxon rank sum test
0.1969
BOT−OvCa Wilcoxon rank sum test
5.544e−05
BOT V600E−lgOvCa Wilcoxon rank sum test
0.659
BOT V600E−OvCa Wilcoxon rank sum test
0.04384
lgOvCa−OvCa Wilcoxon rank sum test
0.9217
Average beta values
0.6
0.5
0.4
0.3
0.2
BOT
BOT.V600E
lgOvCa
OvCa
Sample_Group

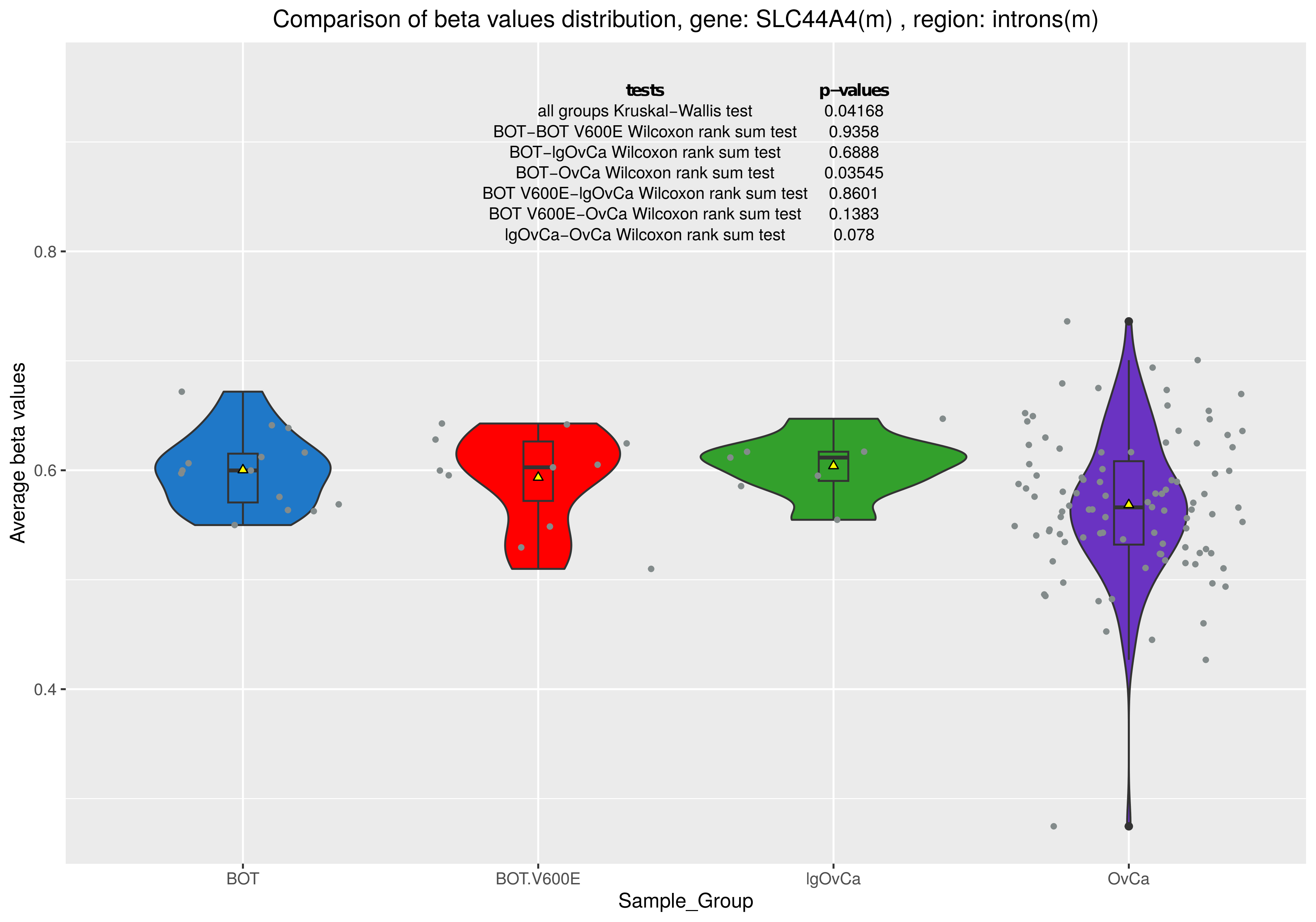

Comparison of beta values distribution, gene: SLC44A4(m) , region: introns(m)
tests
p−values
all groups Kruskal−Wallis test
0.04168
BOT−BOT V600E Wilcoxon rank sum test
0.9358
BOT−lgOvCa Wilcoxon rank sum test
0.6888
BOT−OvCa Wilcoxon rank sum test
0.03545
BOT V600E−lgOvCa Wilcoxon rank sum test
0.8601
BOT V600E−OvCa Wilcoxon rank sum test
0.1383
lgOvCa−OvCa Wilcoxon rank sum test
0.078
Average beta values
0.8
0.6
0.4
BOT
BOT.V600E
lgOvCa
OvCa
Sample_Group

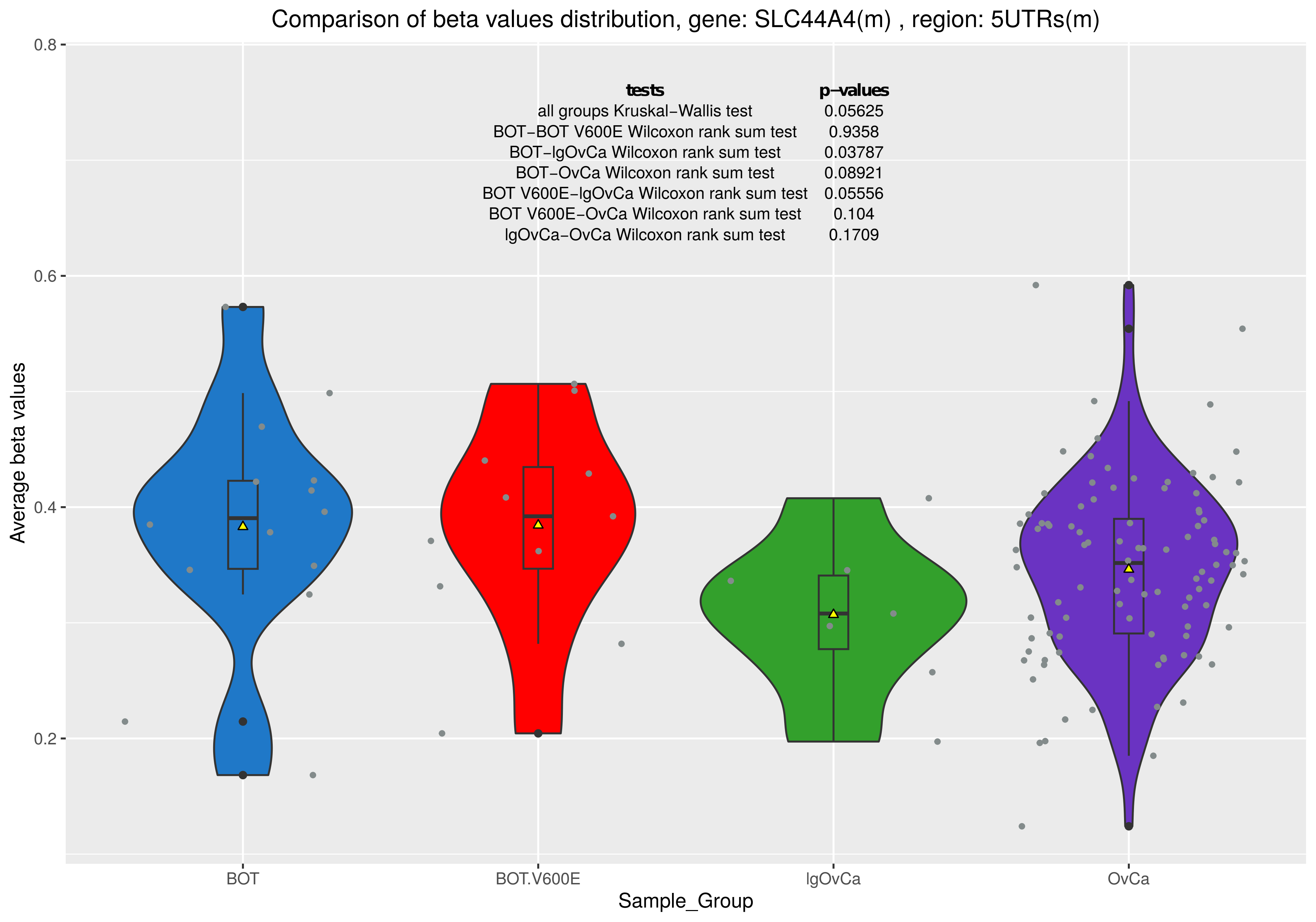
Comparison of beta values distribution, gene: SLC44A4(m) , region: 5UTRs(m)
tests
p−values
all groups Kruskal−Wallis test
0.05625
BOT−BOT V600E Wilcoxon rank sum test
0.9358
BOT−lgOvCa Wilcoxon rank sum test
0.03787
BOT−OvCa Wilcoxon rank sum test
0.08921
BOT V600E−lgOvCa Wilcoxon rank sum test
0.05556
BOT V600E−OvCa Wilcoxon rank sum test
0.104
lgOvCa−OvCa Wilcoxon rank sum test
0.1709
Average beta values
0.8
0.6
0.4
0.2
BOT
BOT.V600E
lgOvCa
OvCa
Sample_Group

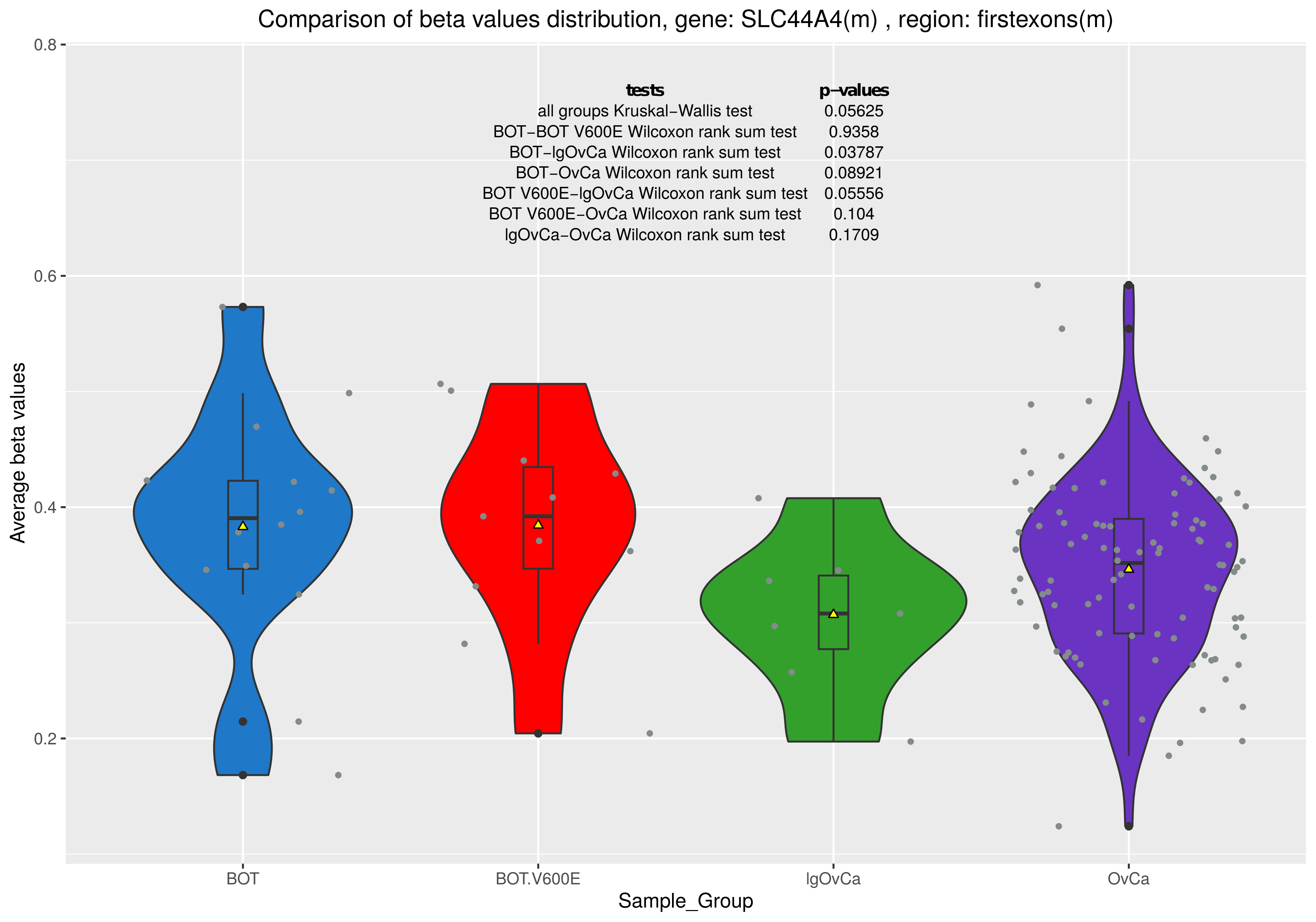

Comparison of beta values distribution, gene: SLC44A4(m) , region: firstexons(m)
tests
p−values
all groups Kruskal−Wallis test
0.05625
BOT−BOT V600E Wilcoxon rank sum test
0.9358
BOT−lgOvCa Wilcoxon rank sum test
0.03787
BOT−OvCa Wilcoxon rank sum test
0.08921
BOT V600E−lgOvCa Wilcoxon rank sum test
0.05556
BOT V600E−OvCa Wilcoxon rank sum test
0.104
lgOvCa−OvCa Wilcoxon rank sum test
0.1709
Average beta values
0.8
0.6
0.4
0.2
BOT
BOT.V600E
lgOvCa
OvCa
Sample_Group

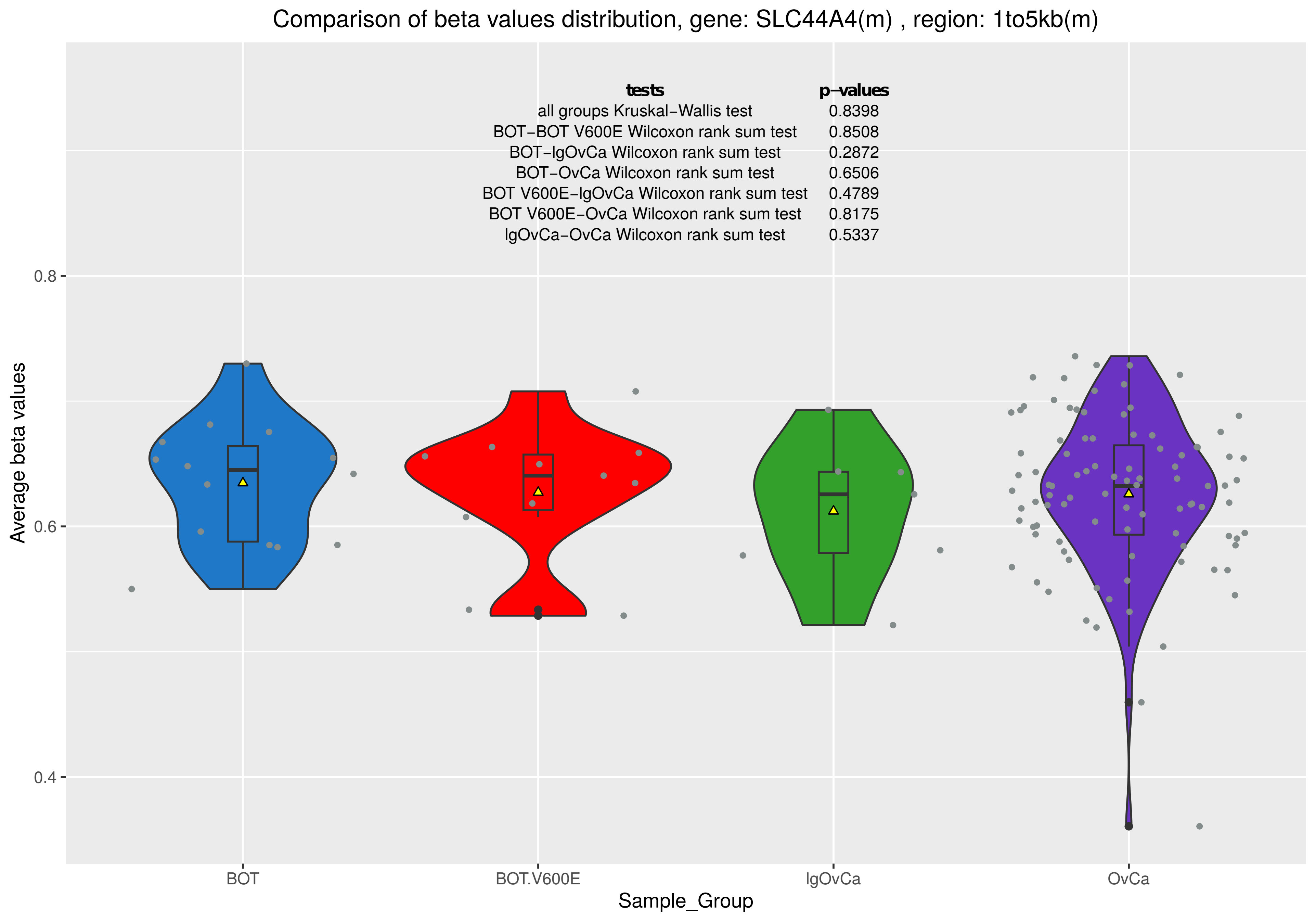

Comparison of beta values distribution, gene: SLC44A4(m) , region: 1to5kb(m)
tests
p−values
all groups Kruskal−Wallis test
0.8398
BOT−BOT V600E Wilcoxon rank sum test
0.8508
BOT−lgOvCa Wilcoxon rank sum test
0.2872
BOT−OvCa Wilcoxon rank sum test
0.6506
BOT V600E−lgOvCa Wilcoxon rank sum test
0.4789
BOT V600E−OvCa Wilcoxon rank sum test
0.8175
lgOvCa−OvCa Wilcoxon rank sum test
0.5337
Average beta values
0.8
0.6
0.4
BOT
BOT.V600E
lgOvCa
OvCa
Sample_Group

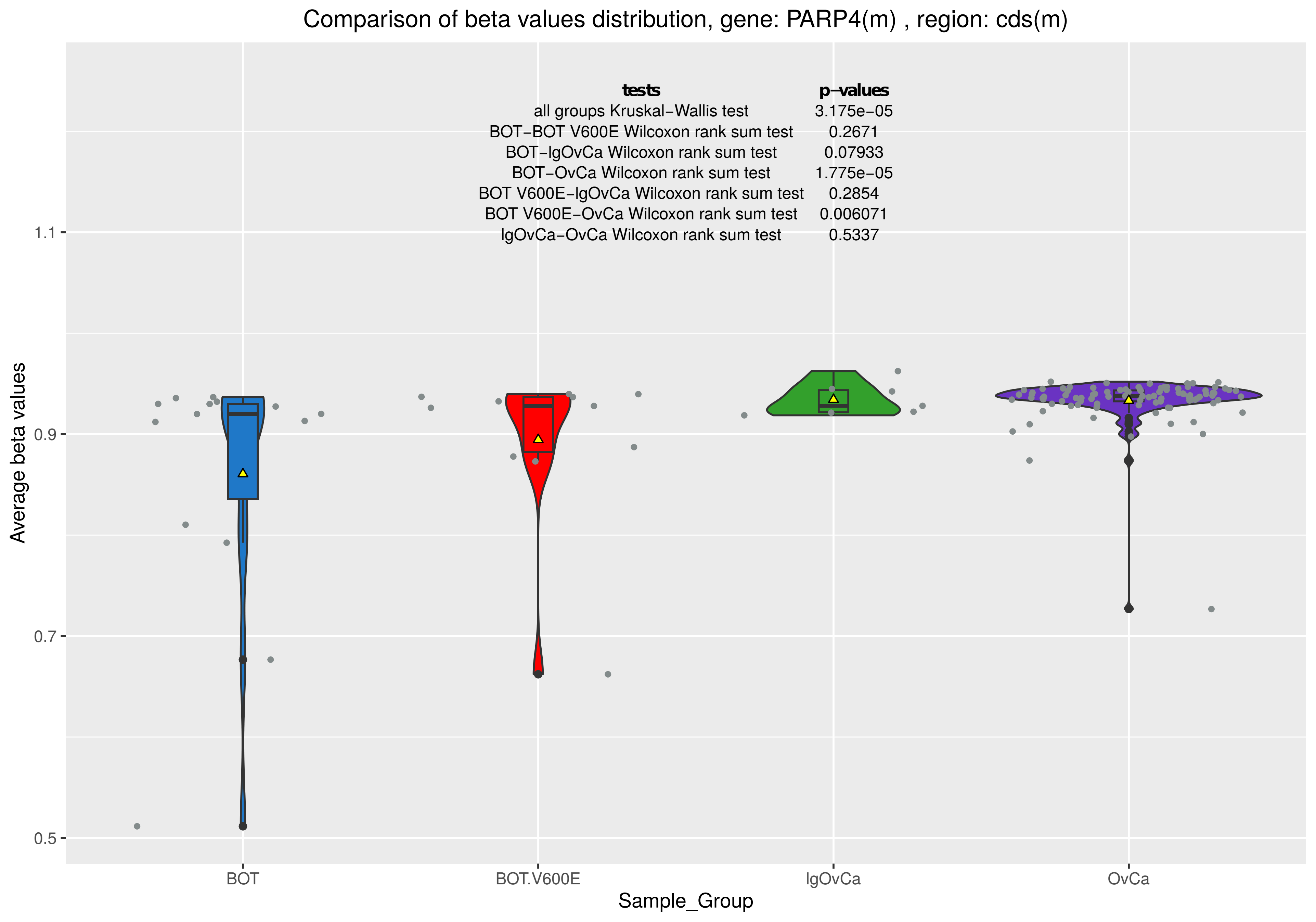

Comparison of beta values distribution, gene: PARP4(m) , region: cds(m)
tests
p−values
all groups Kruskal−Wallis test
3.175e−05
BOT−BOT V600E Wilcoxon rank sum test
0.2671
BOT−lgOvCa Wilcoxon rank sum test
0.07933
BOT−OvCa Wilcoxon rank sum test
1.775e−05
BOT V600E−lgOvCa Wilcoxon rank sum test
0.2854
BOT V600E−OvCa Wilcoxon rank sum test
0.006071
lgOvCa−OvCa Wilcoxon rank sum test
0.5337
Average beta values
1.1
0.9
0.7
0.5
BOT
BOT.V600E
lgOvCa
OvCa
Sample_Group

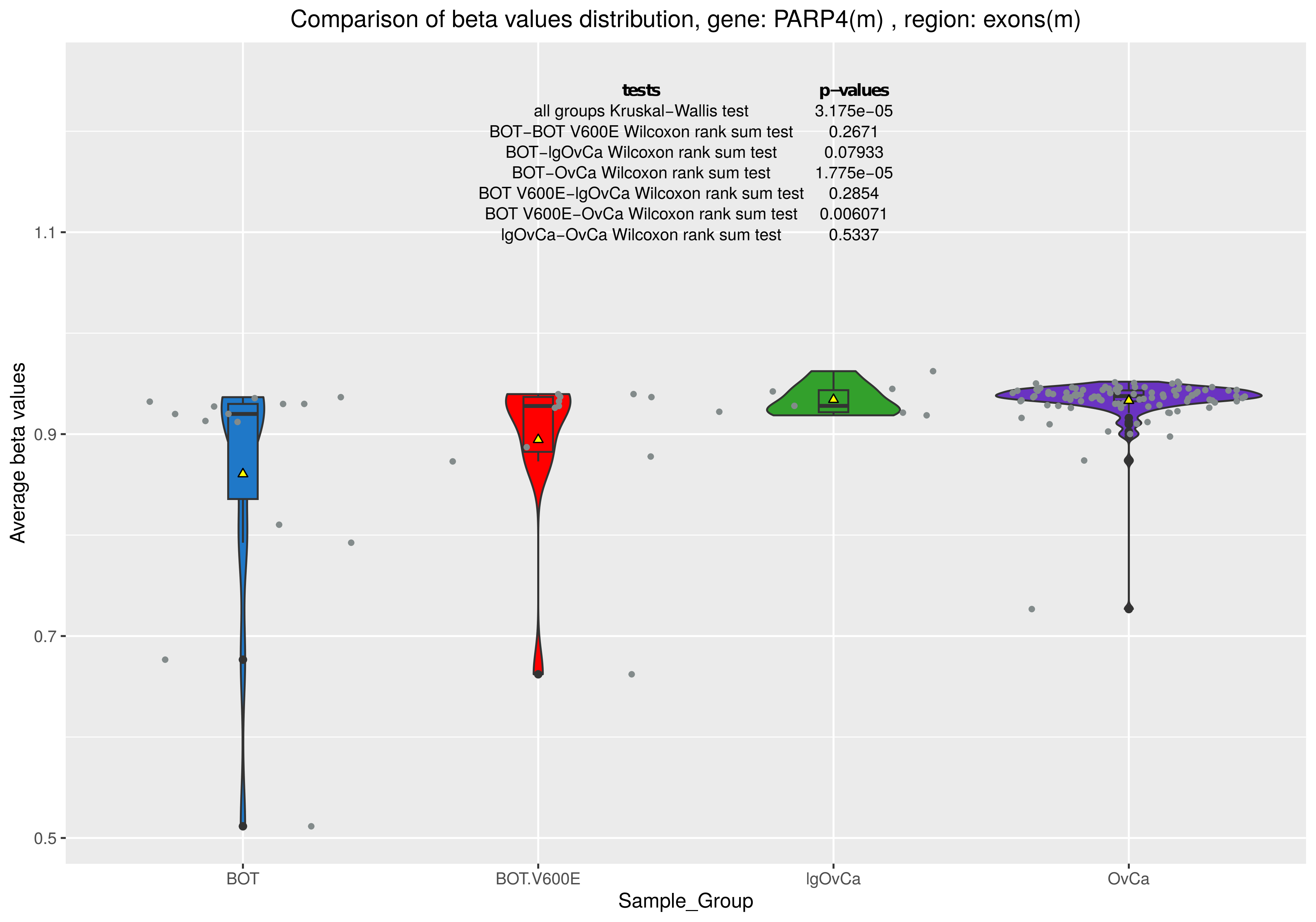
Comparison of beta values distribution, gene: PARP4(m) , region: exons(m)
tests
p−values
all groups Kruskal−Wallis test
3.175e−05
BOT−BOT V600E Wilcoxon rank sum test
0.2671
BOT−lgOvCa Wilcoxon rank sum test
0.07933
BOT−OvCa Wilcoxon rank sum test
1.775e−05
BOT V600E−lgOvCa Wilcoxon rank sum test
0.2854
BOT V600E−OvCa Wilcoxon rank sum test
0.006071
lgOvCa−OvCa Wilcoxon rank sum test
0.5337
Average beta values
1.1
0.9
0.7
0.5
BOT
BOT.V600E
lgOvCa
OvCa
Sample_Group

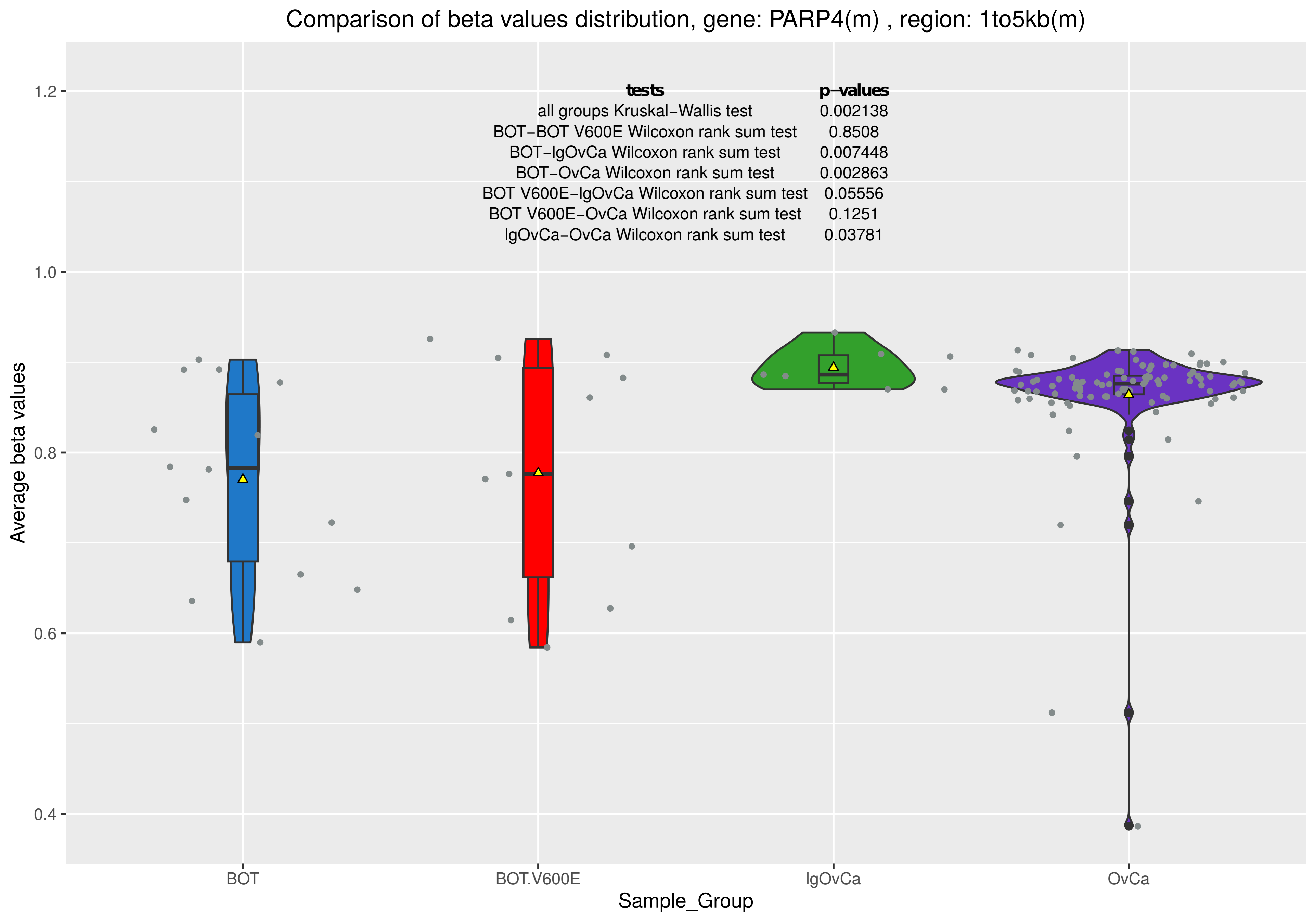

Comparison of beta values distribution, gene: PARP4(m) , region: 1to5kb(m)
tests
p−values
all groups Kruskal−Wallis test 0.002138
BOT−BOT V600E Wilcoxon rank sum test 0.8508
BOT−lgOvCa Wilcoxon rank sum test 0.007448
BOT−OvCa Wilcoxon rank sum test 0.002863
BOT V600E−lgOvCa Wilcoxon rank sum test 0.05556
BOT V600E−OvCa Wilcoxon rank sum test 0.1251
lgOvCa−OvCa Wilcoxon rank sum test 0.03781
Average beta values
1.2
1.0
0.8
0.6
0.4
BOT
BOT.V600E
lgOvCa
OvCa
Sample_Group

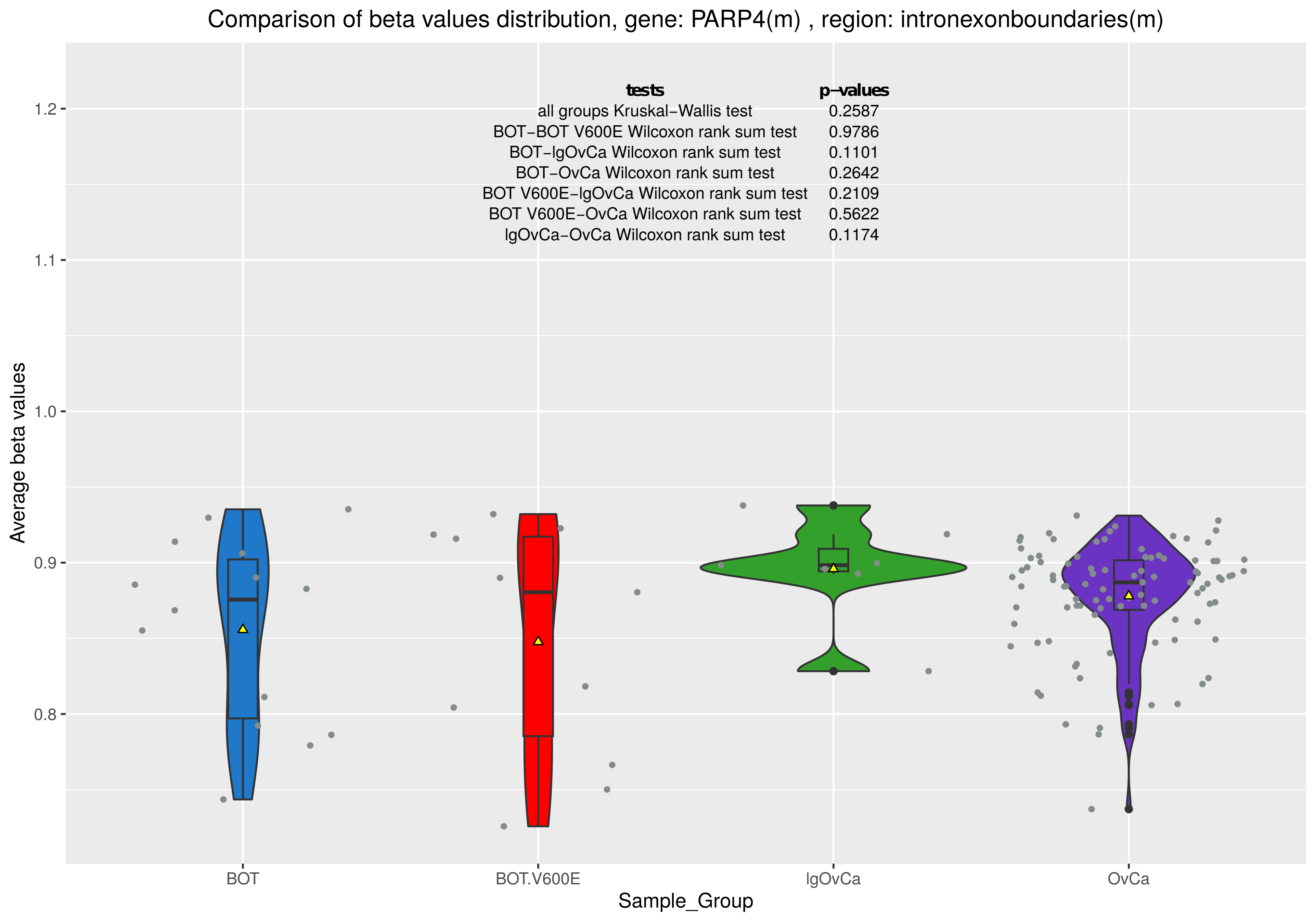
Comparison of beta values distribution, gene: PARP4(m) , region: intronexonboundaries(m)
tests
p−values
all groups Kruskal−Wallis test
0.2587
BOT−BOT V600E Wilcoxon rank sum test
0.9786
BOT−lgOvCa Wilcoxon rank sum test
0.1101
BOT−OvCa Wilcoxon rank sum test
0.2642
BOT V600E−lgOvCa Wilcoxon rank sum test
0.2109
BOT V600E−OvCa Wilcoxon rank sum test
0.5622
lgOvCa−OvCa Wilcoxon rank sum test
0.1174
1.2
1.1
1.0
0.9
0.8
Average beta values
BOT
BOT.V600E
lgOvCa
OvCa
Sample_Group

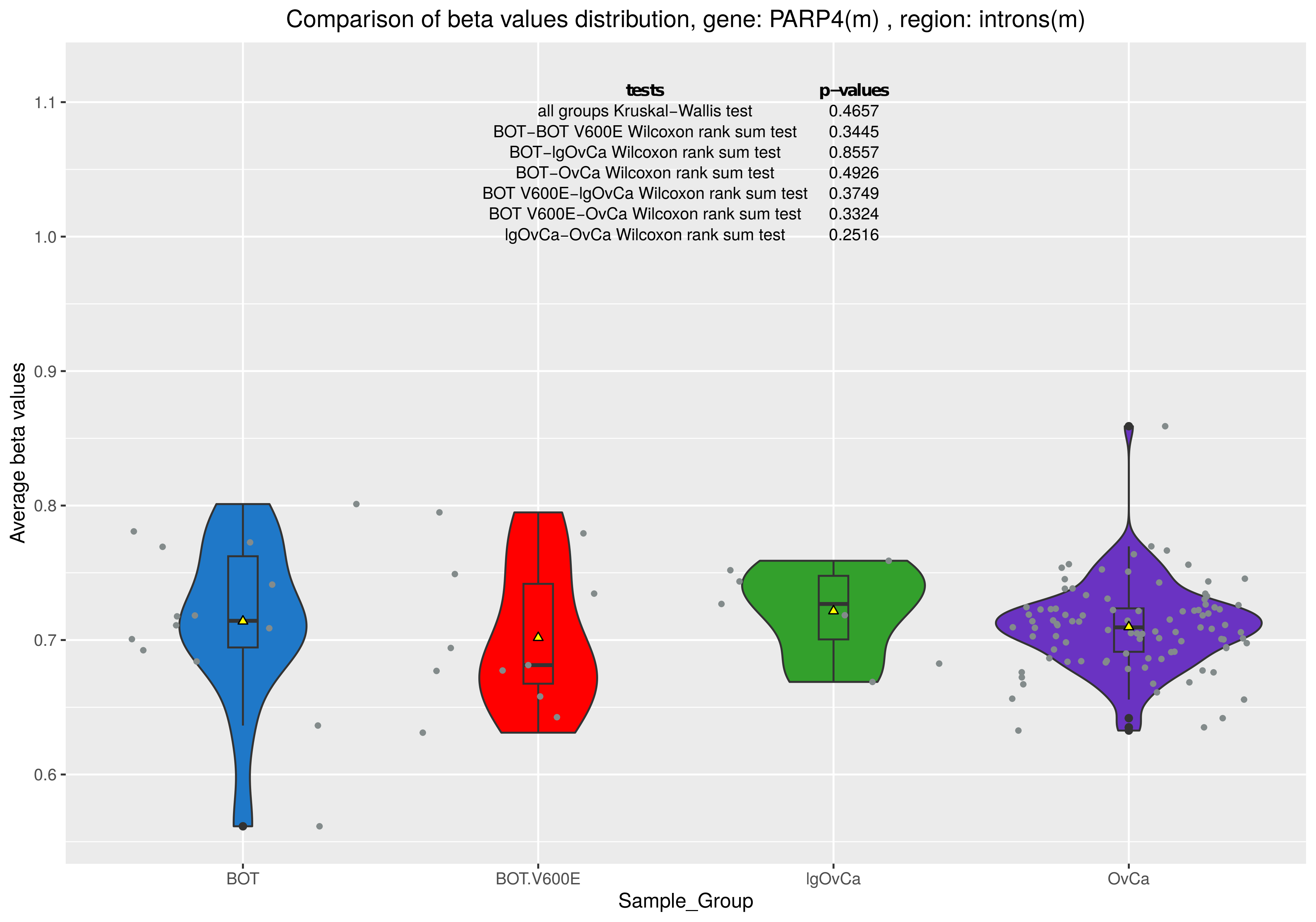

Comparison of beta values distribution, gene: PARP4(m) , region: introns(m)
tests
p−values
all groups Kruskal−Wallis test
0.4657
BOT−BOT V600E Wilcoxon rank sum test
0.3445
BOT−IgOvCa Wilcoxon rank sum test
0.8557
BOT−OvCa Wilcoxon rank sum test
0.4926
BOT V600E−IgOvCa Wilcoxon rank sum test
0.3749
BOT V600E−OvCa Wilcoxon rank sum test
0.3324
IgOvCa−OvCa Wilcoxon rank sum test
0.2516
Average beta values
1.1
1.0
0.9
0.8
0.7
0.6
BOT
BOT.V600E
IgOvCa
OvCa
Sample_Group

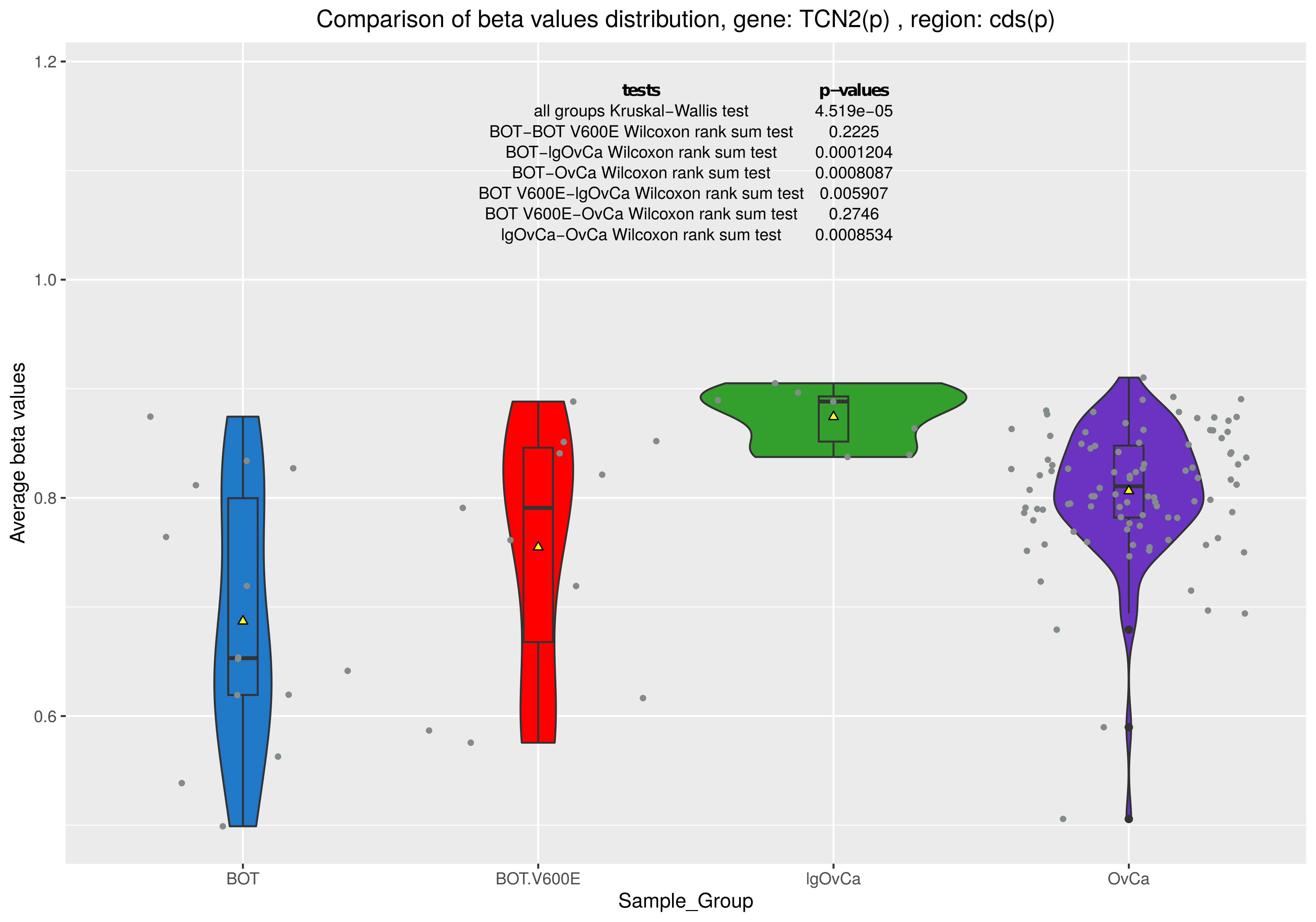
Comparison of beta values distribution, gene: TCN2(p) , region: cds(p)
tests
p−values
all groups Kruskal−Wallis test
4.519e−05
BOT−BOT V600E Wilcoxon rank sum test
0.2225
BOT−lgOvCa Wilcoxon rank sum test
0.0001204
BOT−OvCa Wilcoxon rank sum test
0.0008087
BOT V600E−lgOvCa Wilcoxon rank sum test
0.005907
BOT V600E−OvCa Wilcoxon rank sum test
0.2746
lgOvCa−OvCa Wilcoxon rank sum test
0.0008534
Average beta values
1.2
1.0
0.8
0.6
BOT
BOT.V600E
lgOvCa
OvCa
Sample_Group

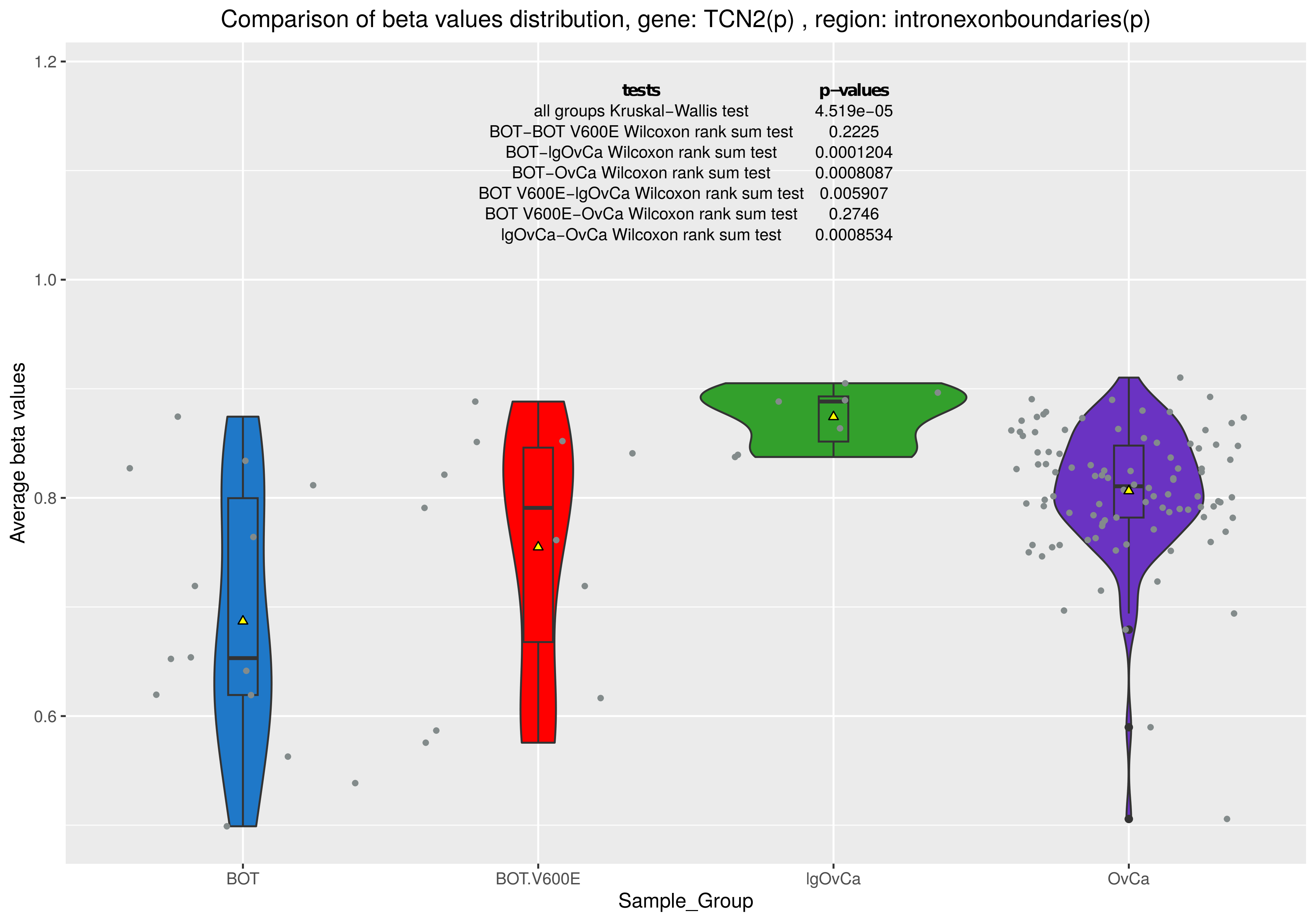

Comparison of beta values distribution, gene: TCN2(p) , region: intronexonboundaries(p)
tests
p−values
all groups Kruskal−Wallis test
4.519e−05
BOT−BOT V600E Wilcoxon rank sum test
0.2225
BOT−lgOvCa Wilcoxon rank sum test
0.0001204
BOT−OvCa Wilcoxon rank sum test
0.0008087
BOT V600E−lgOvCa Wilcoxon rank sum test
0.005907
BOT V600E−OvCa Wilcoxon rank sum test
0.2746
lgOvCa−OvCa Wilcoxon rank sum test
0.0008534
Average beta values
1.2
1.0
0.8
0.6
BOT
BOT.V600E
lgOvCa
OvCa
Sample_Group

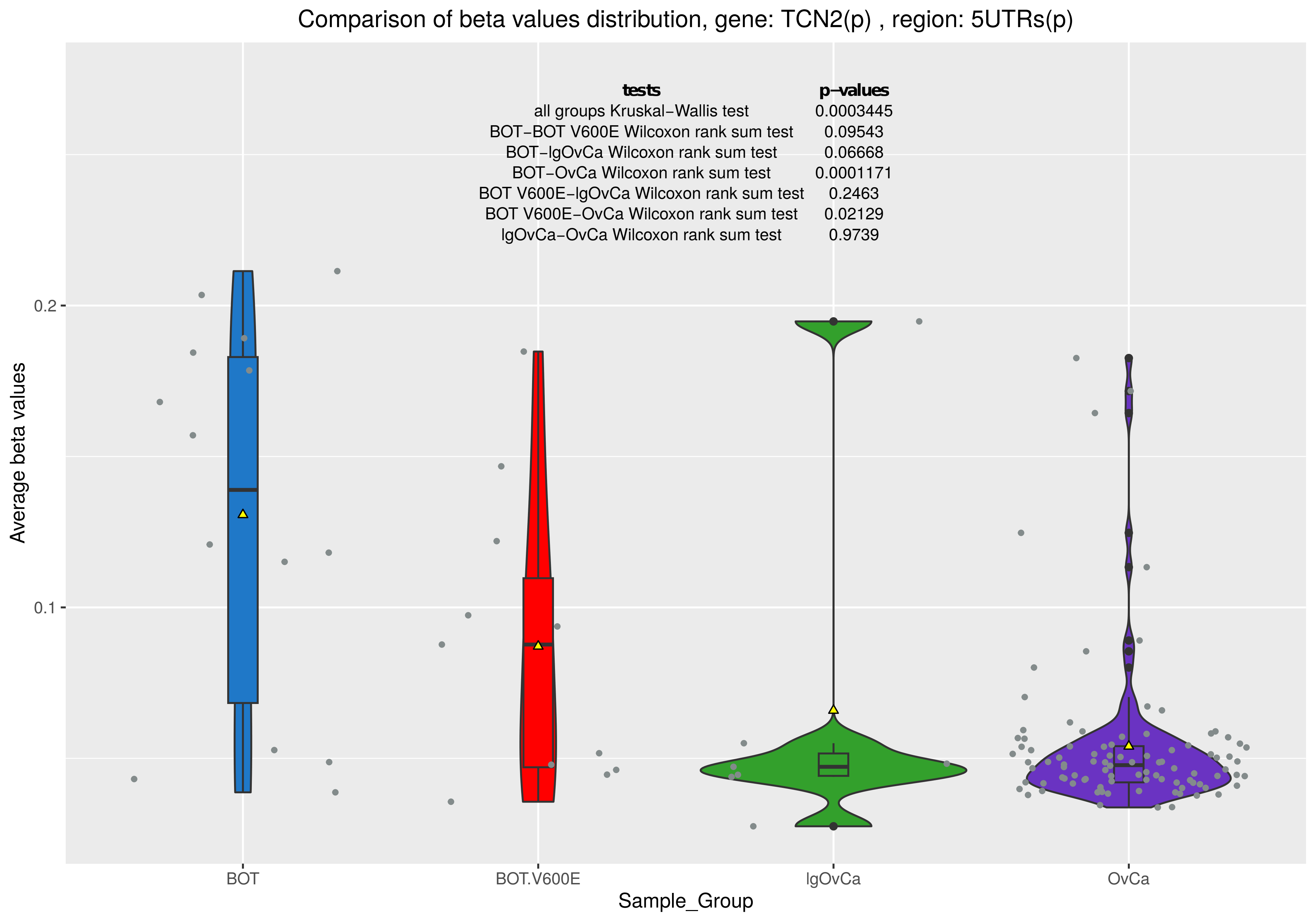
Comparison of beta values distribution, gene: TCN2(p) , region: 5UTRs(p)
tests
p−values
all groups Kruskal−Wallis test
0.0003445
BOT−BOT V600E Wilcoxon rank sum test
0.09543
BOT−lgOvCa Wilcoxon rank sum test
0.06668
BOT−OvCa Wilcoxon rank sum test
0.0001171
BOT V600E−lgOvCa Wilcoxon rank sum test
0.2463
BOT V600E−OvCa Wilcoxon rank sum test
0.02129
lgOvCa−OvCa Wilcoxon rank sum test
0.9739
Average beta values
0.2
0.1
BOT
BOT.V600E
lgOvCa
OvCa
Sample_Group

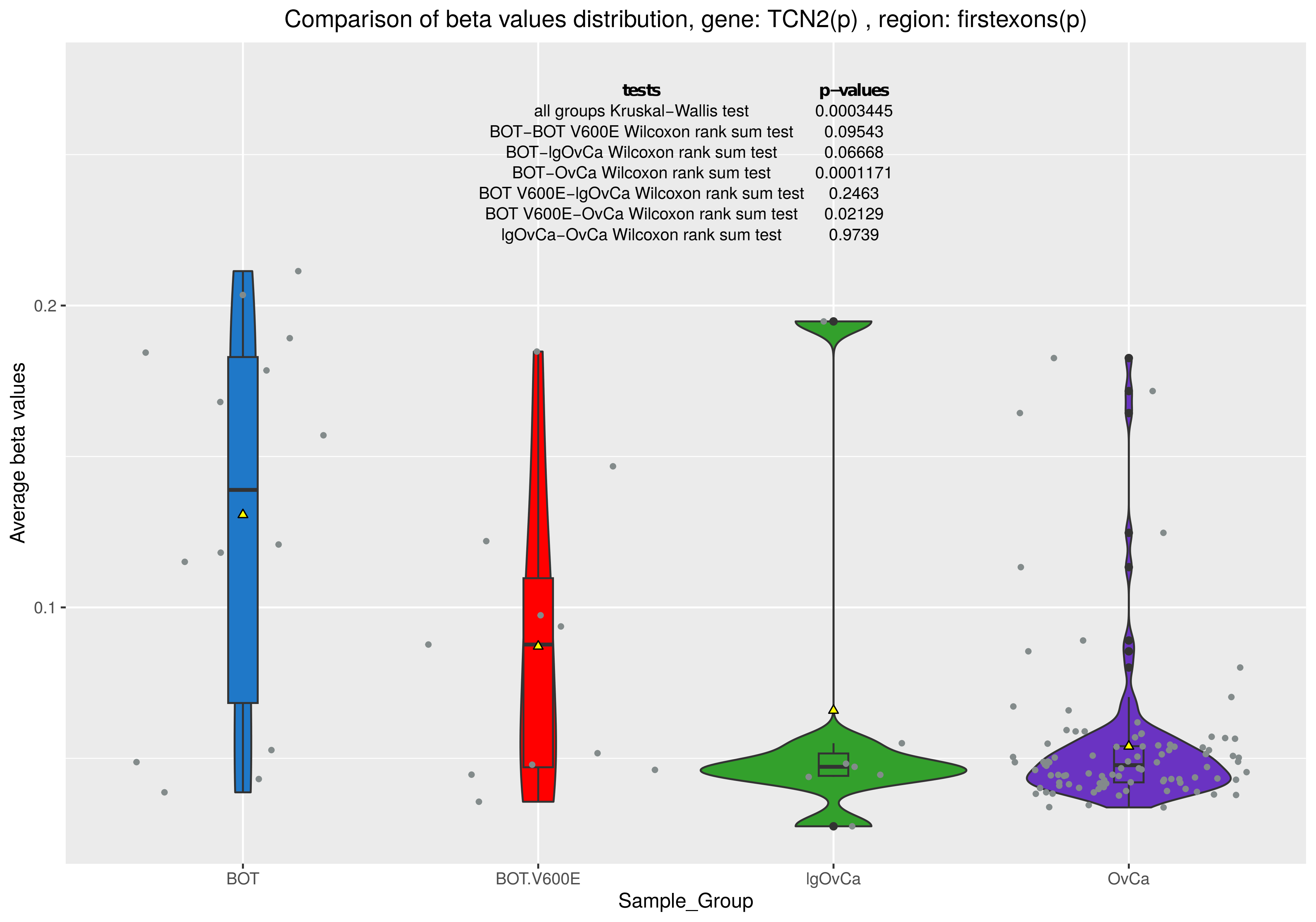

Comparison of beta values distribution, gene: TCN2(p) , region: firstexons(p)
tests
p−values
all groups Kruskal−Wallis test
0.0003445
BOT−BOT V600E Wilcoxon rank sum test
0.09543
BOT−lgOvCa Wilcoxon rank sum test
0.06668
BOT−OvCa Wilcoxon rank sum test
0.0001171
BOT V600E−lgOvCa Wilcoxon rank sum test
0.2463
BOT V600E−OvCa Wilcoxon rank sum test
0.02129
lgOvCa−OvCa Wilcoxon rank sum test
0.9739
Average beta values
0.2
0.1
BOT
BOT.V600E
lgOvCa
OvCa
Sample_Group

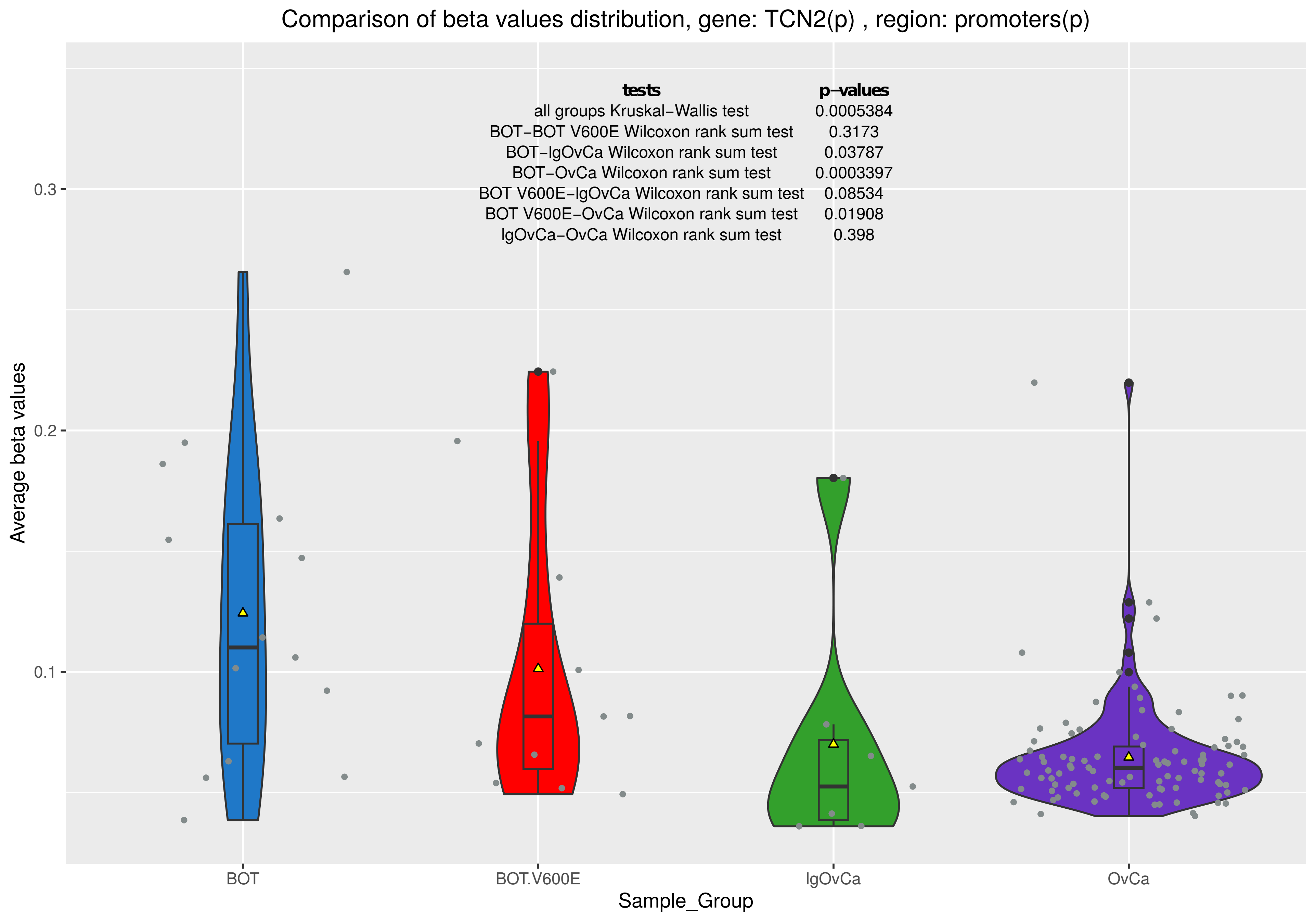

Comparison of beta values distribution, gene: TCN2(p) , region: promoters(p)
tests
p−values
all groups Kruskal−Wallis test
0.0005384
BOT−BOT V600E Wilcoxon rank sum test
0.3173
BOT−lgOvCa Wilcoxon rank sum test
0.03787
BOT−OvCa Wilcoxon rank sum test
0.0003397
BOT V600E−lgOvCa Wilcoxon rank sum test
0.08534
BOT V600E−OvCa Wilcoxon rank sum test
0.01908
lgOvCa−OvCa Wilcoxon rank sum test
0.398
Average beta values
0.3
0.2
0.1
BOT
BOT.V600E
lgOvCa
OvCa
Sample_Group

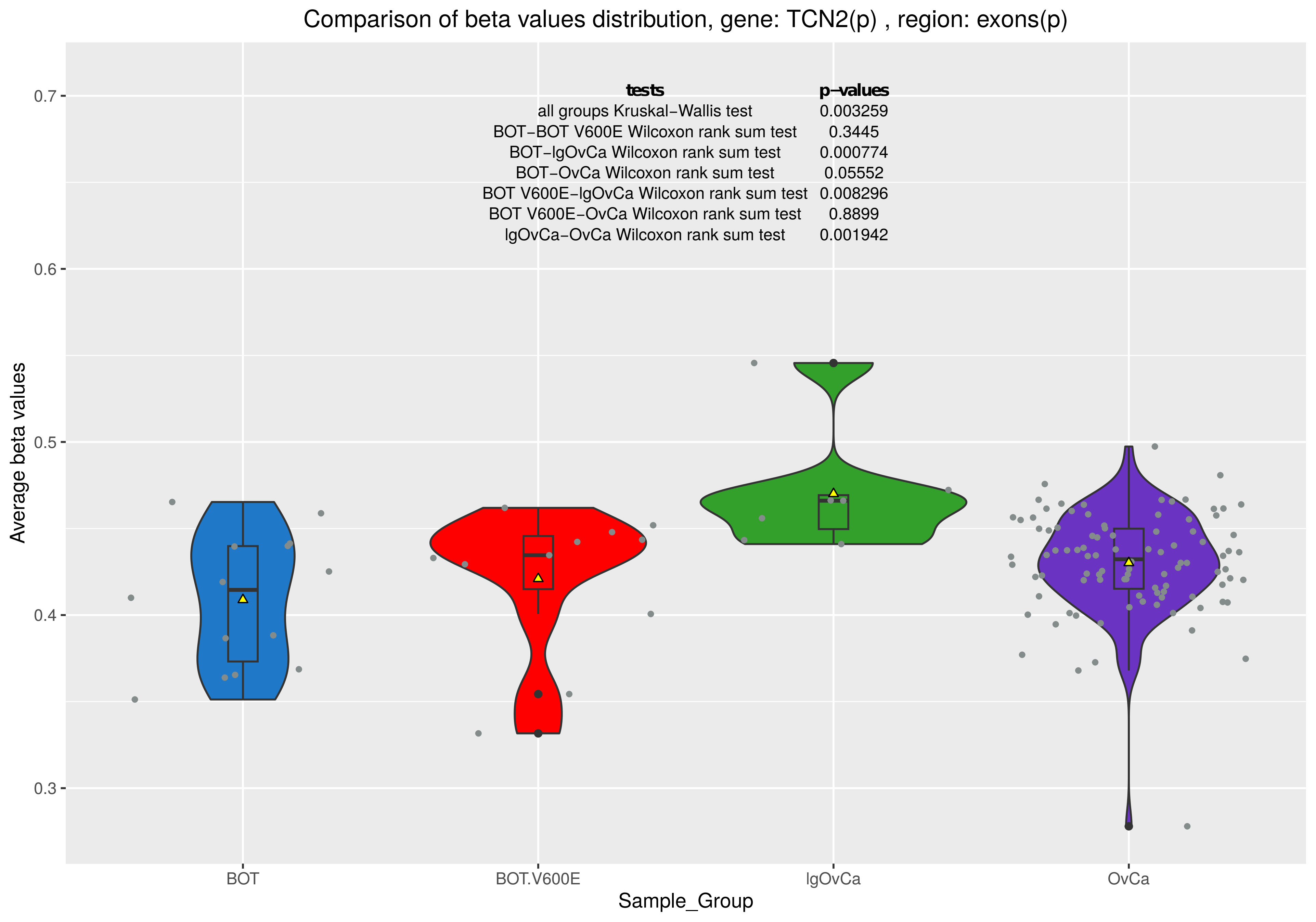
Comparison of beta values distribution, gene: TCN2(p) , region: exons(p)
tests
p−values
all groups Kruskal−Wallis test 0.003259
BOT−BOT V600E Wilcoxon rank sum test 0.3445
BOT−lgOvCa Wilcoxon rank sum test 0.000774
BOT−OvCa Wilcoxon rank sum test 0.05552
BOT V600E−lgOvCa Wilcoxon rank sum test 0.008296
BOT V600E−OvCa Wilcoxon rank sum test 0.8899
lgOvCa−OvCa Wilcoxon rank sum test 0.001942
Average beta values
0.7
0.6
0.5
0.4
0.3
BOT
BOT.V600E
lgOvCa
OvCa
Sample_Group

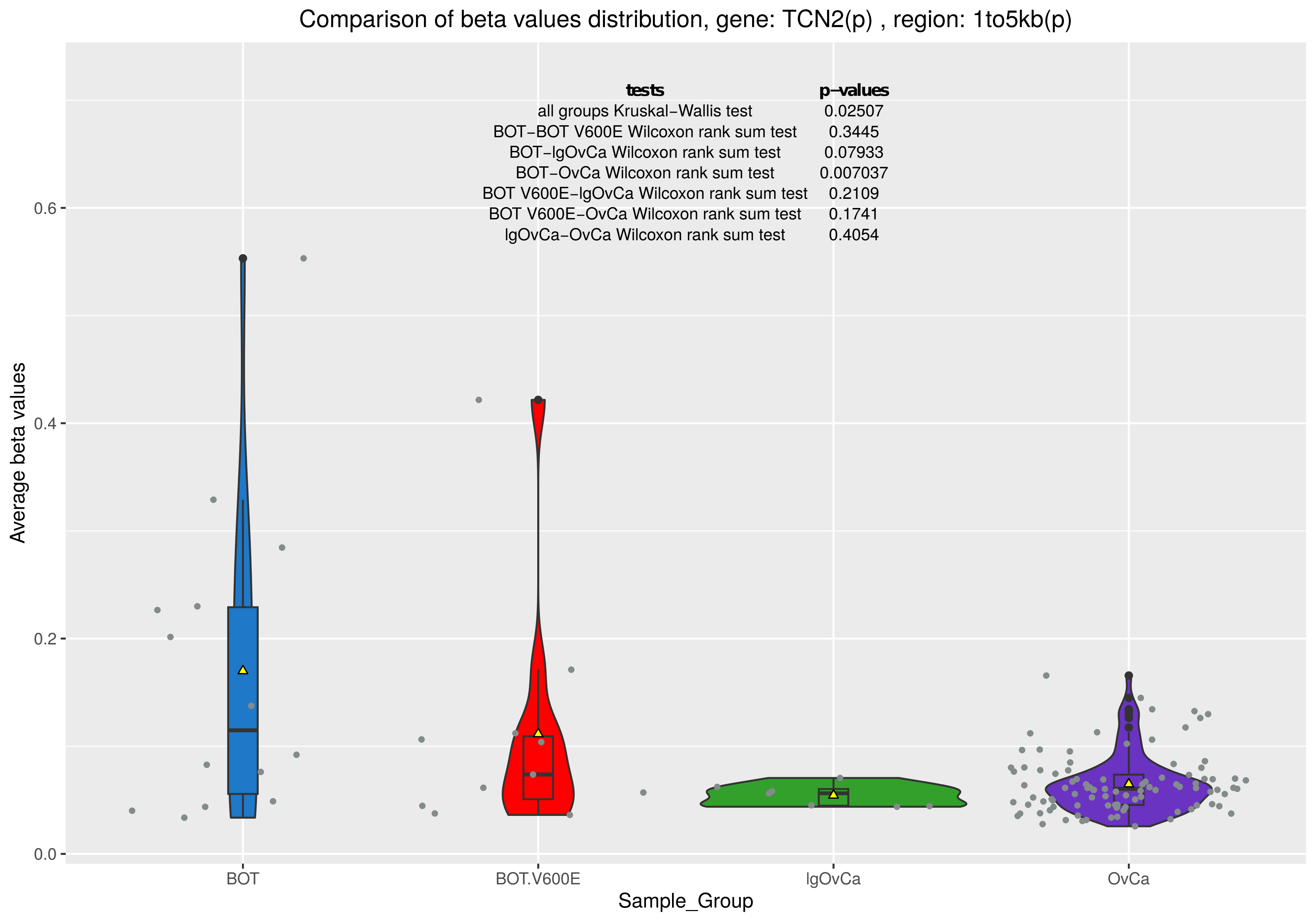

Comparison of beta values distribution, gene: TCN2(p) , region: 1to5kb(p)
tests
p−values
all groups Kruskal−Wallis test
0.02507
BOT−BOT V600E Wilcoxon rank sum test
0.3445
BOT−lgOvCa Wilcoxon rank sum test
0.07933
BOT−OvCa Wilcoxon rank sum test
0.007037
BOT V600E−lgOvCa Wilcoxon rank sum test
0.2109
BOT V600E−OvCa Wilcoxon rank sum test
0.1741
lgOvCa−OvCa Wilcoxon rank sum test
0.4054
Average beta values
0.6
0.4
0.2
0.0
BOT
BOT.V600E
lgOvCa
OvCa
Sample_Group

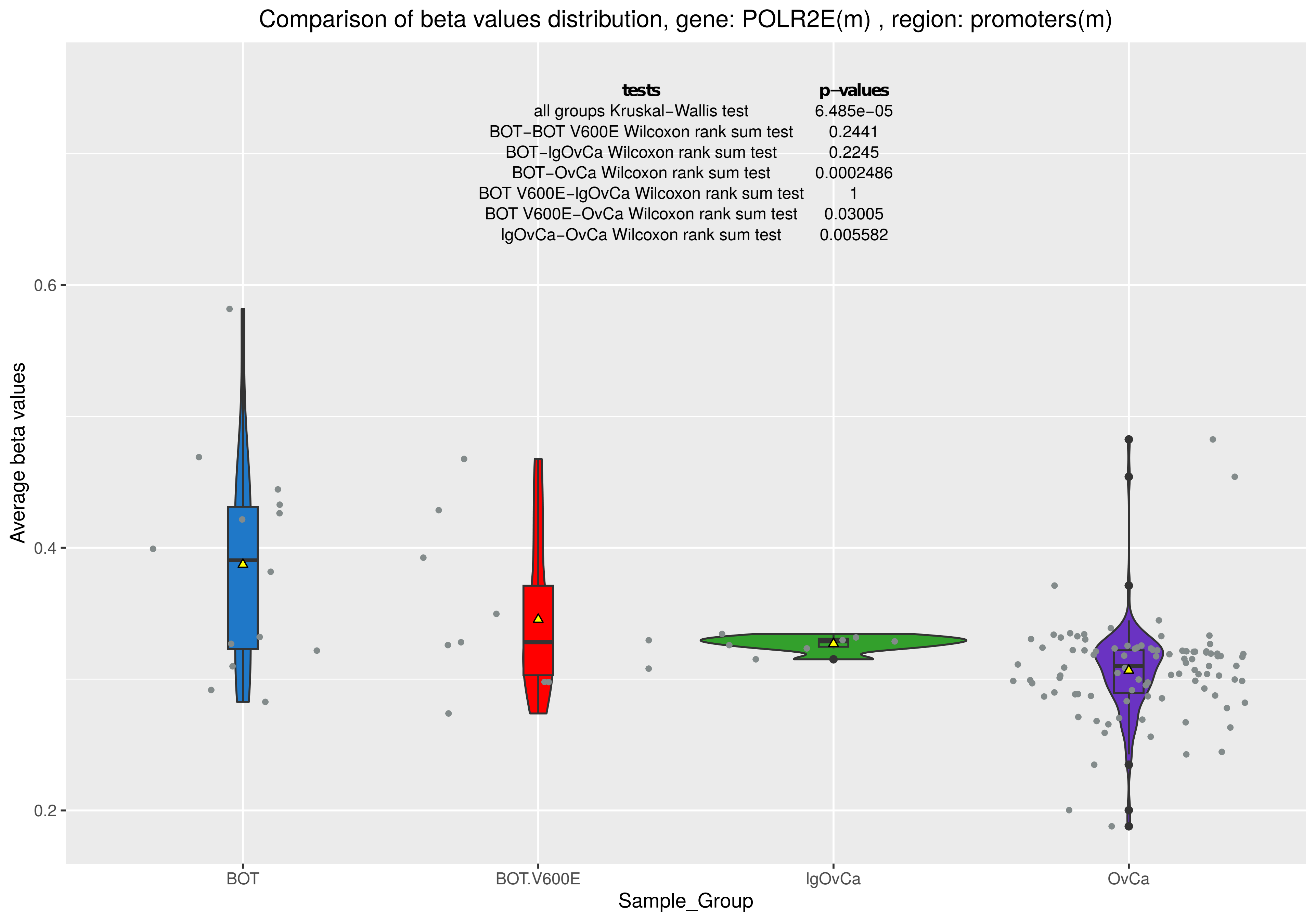

Comparison of beta values distribution, gene: POLR2E(m) , region: promoters(m)
tests
p−values
all groups Kruskal−Wallis test
6.485e−05
BOT−BOT V600E Wilcoxon rank sum test
0.2441
BOT−lgOvCa Wilcoxon rank sum test
0.2245
BOT−OvCa Wilcoxon rank sum test
0.0002486
BOT V600E−lgOvCa Wilcoxon rank sum test
1
BOT V600E−OvCa Wilcoxon rank sum test
0.03005
lgOvCa−OvCa Wilcoxon rank sum test
0.005582
Average beta values
0.6
0.4
0.2
BOT
BOT.V600E
lgOvCa
OvCa
Sample_Group

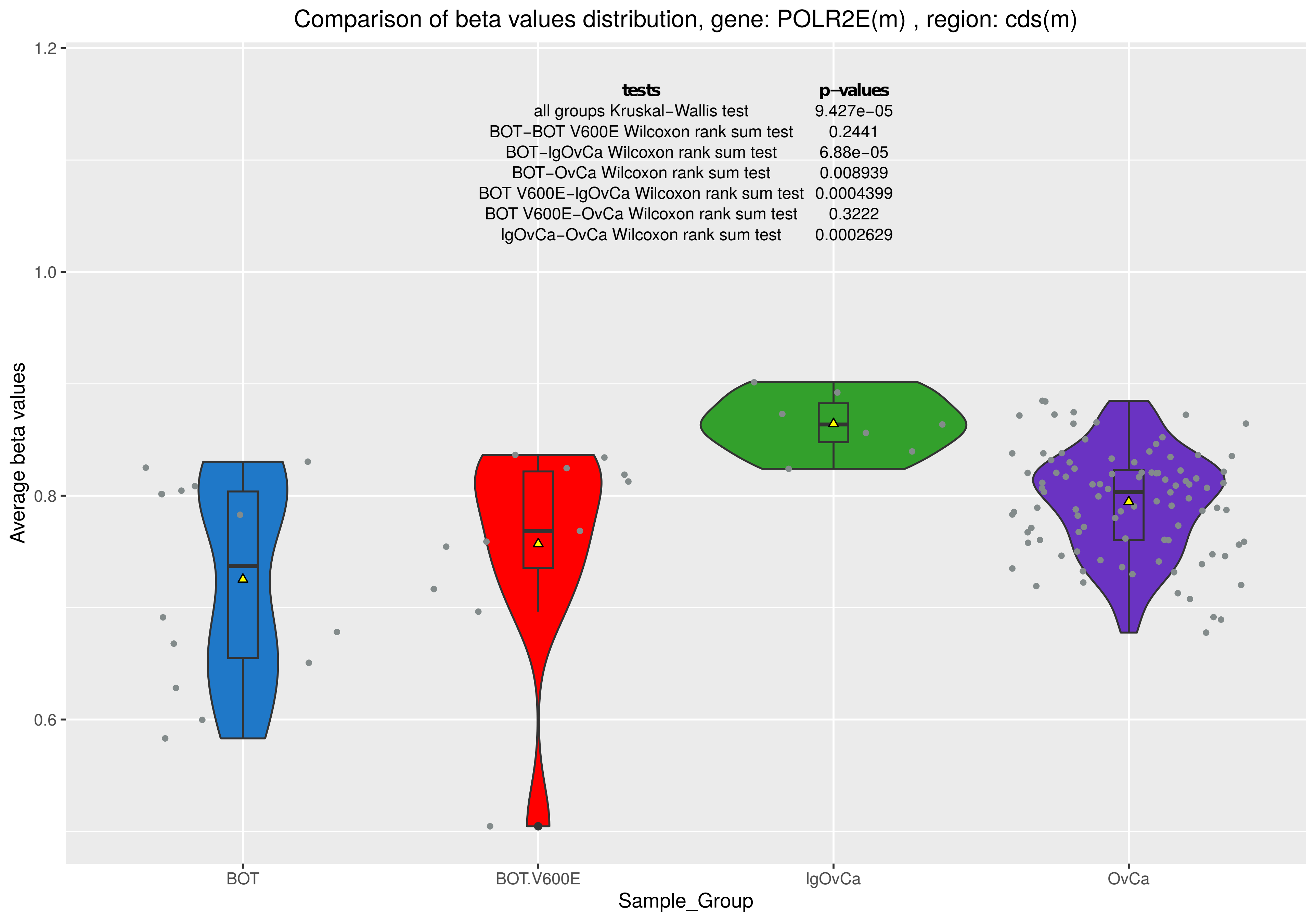

Comparison of beta values distribution, gene: POLR2E(m) , region: cds(m)
tests
p−values
all groups Kruskal−Wallis test
9.427e−05
BOT−BOT V600E Wilcoxon rank sum test
0.2441
BOT−lgOvCa Wilcoxon rank sum test
6.88e−05
BOT−OvCa Wilcoxon rank sum test
0.008939
BOT V600E−lgOvCa Wilcoxon rank sum test
0.0004399
BOT V600E−OvCa Wilcoxon rank sum test
0.3222
lgOvCa−OvCa Wilcoxon rank sum test
0.0002629
Average beta values
1.2
1.0
0.8
0.6
BOT
BOT.V600E
lgOvCa
OvCa
Sample_Group

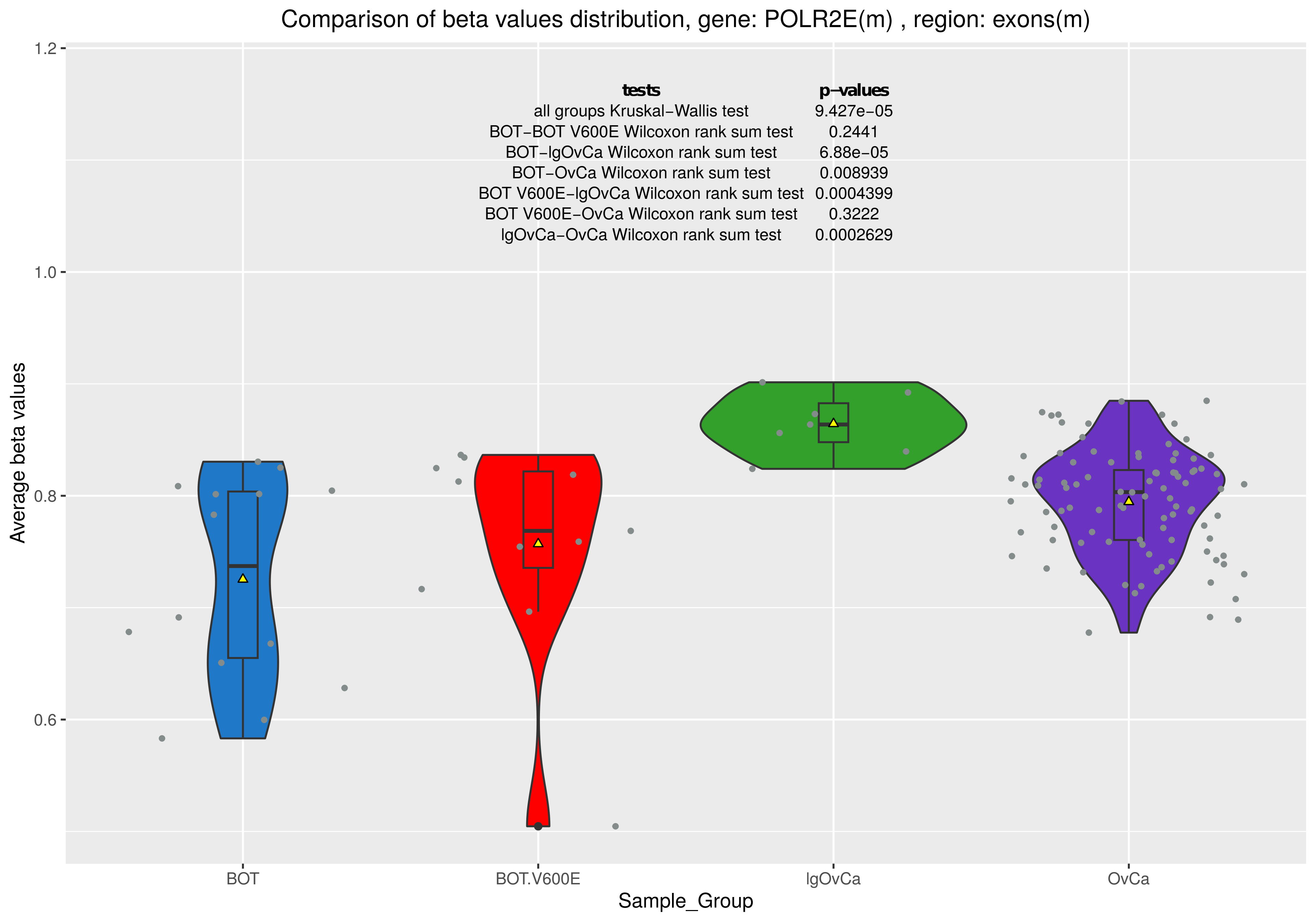
Comparison of beta values distribution, gene: POLR2E(m) , region: exons(m)
tests
p−values
all groups Kruskal−Wallis test
9.427e−05
BOT−BOT V600E Wilcoxon rank sum test
0.2441
BOT−lgOvCa Wilcoxon rank sum test
6.88e−05
BOT−OvCa Wilcoxon rank sum test
0.008939
BOT V600E−lgOvCa Wilcoxon rank sum test
0.0004399
BOT V600E−OvCa Wilcoxon rank sum test
0.3222
lgOvCa−OvCa Wilcoxon rank sum test
0.0002629
Average beta values
1.2
1.0
0.8
0.6
BOT
BOT.V600E
lgOvCa
OvCa
Sample_Group

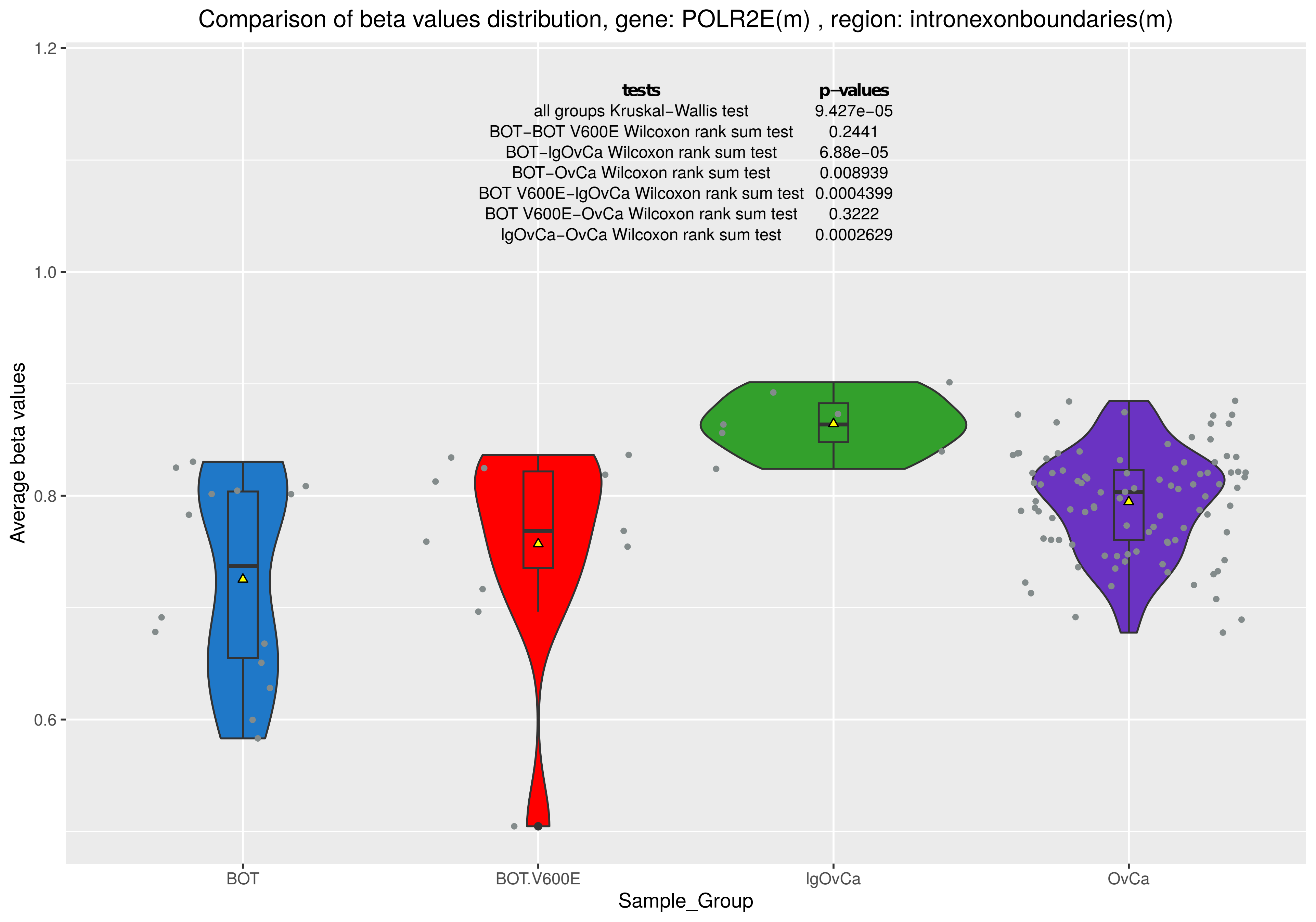
Comparison of beta values distribution, gene: POLR2E(m) , region: intronexonboundaries(m)
tests
p−values
all groups Kruskal−Wallis test
9.427e−05
BOT−BOT V600E Wilcoxon rank sum test
0.2441
BOT−lgOvCa Wilcoxon rank sum test
6.88e−05
BOT−OvCa Wilcoxon rank sum test
0.008939
BOT V600E−lgOvCa Wilcoxon rank sum test
0.0004399
BOT V600E−OvCa Wilcoxon rank sum test
0.3222
lgOvCa−OvCa Wilcoxon rank sum test
0.0002629
Average beta values
1.2
1.0
0.8
0.6
BOT
BOT.V600E
lgOvCa
OvCa
Sample_Group

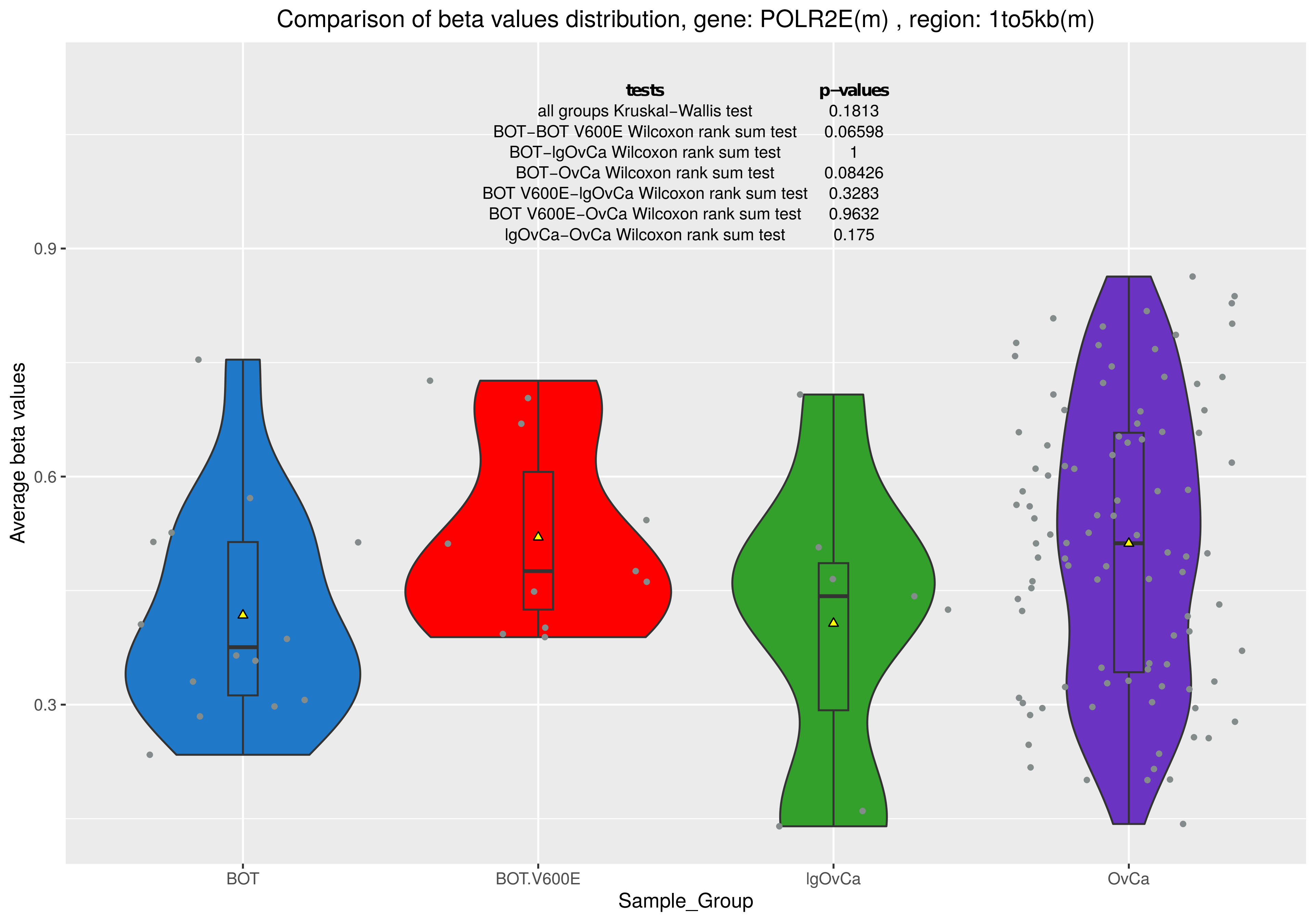

Comparison of beta values distribution, gene: POLR2E(m) , region: 1to5kb(m)
tests
p−values
all groups Kruskal−Wallis test
0.1813
BOT−BOT V600E Wilcoxon rank sum test
0.06598
BOT−lgOvCa Wilcoxon rank sum test
1
BOT−OvCa Wilcoxon rank sum test
0.08426
BOT V600E−lgOvCa Wilcoxon rank sum test
0.3283
BOT V600E−OvCa Wilcoxon rank sum test
0.9632
lgOvCa−OvCa Wilcoxon rank sum test
0.175
Average beta values
0.9
0.6
0.3
BOT
BOT.V600E
lgOvCa
OvCa
Sample_Group

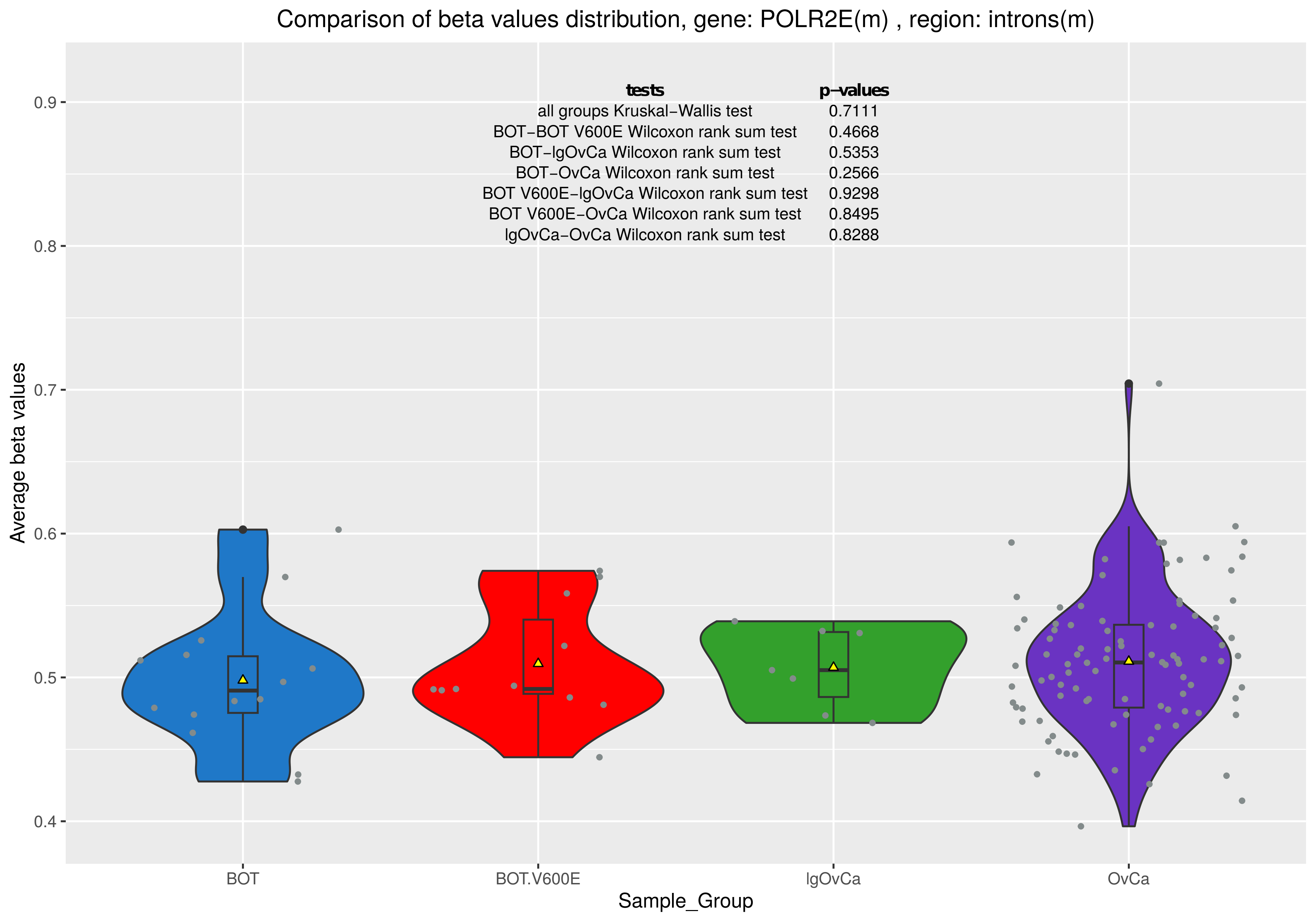

Comparison of beta values distribution, gene: POLR2E(m) , region: introns(m)
tests
p−values
all groups Kruskal−Wallis test
0.7111
BOT−BOT V600E Wilcoxon rank sum test
0.4668
BOT−IgOvCa Wilcoxon rank sum test
0.5353
BOT−OvCa Wilcoxon rank sum test
0.2566
BOT V600E−IgOvCa Wilcoxon rank sum test
0.9298
BOT V600E−OvCa Wilcoxon rank sum test
0.8495
IgOvCa−OvCa Wilcoxon rank sum test
0.8288
Average beta values
0.4
0.5
0.6
0.7
0.8
0.9
BOT
BOT.V600E
IgOvCa
OvCa
Sample_Group

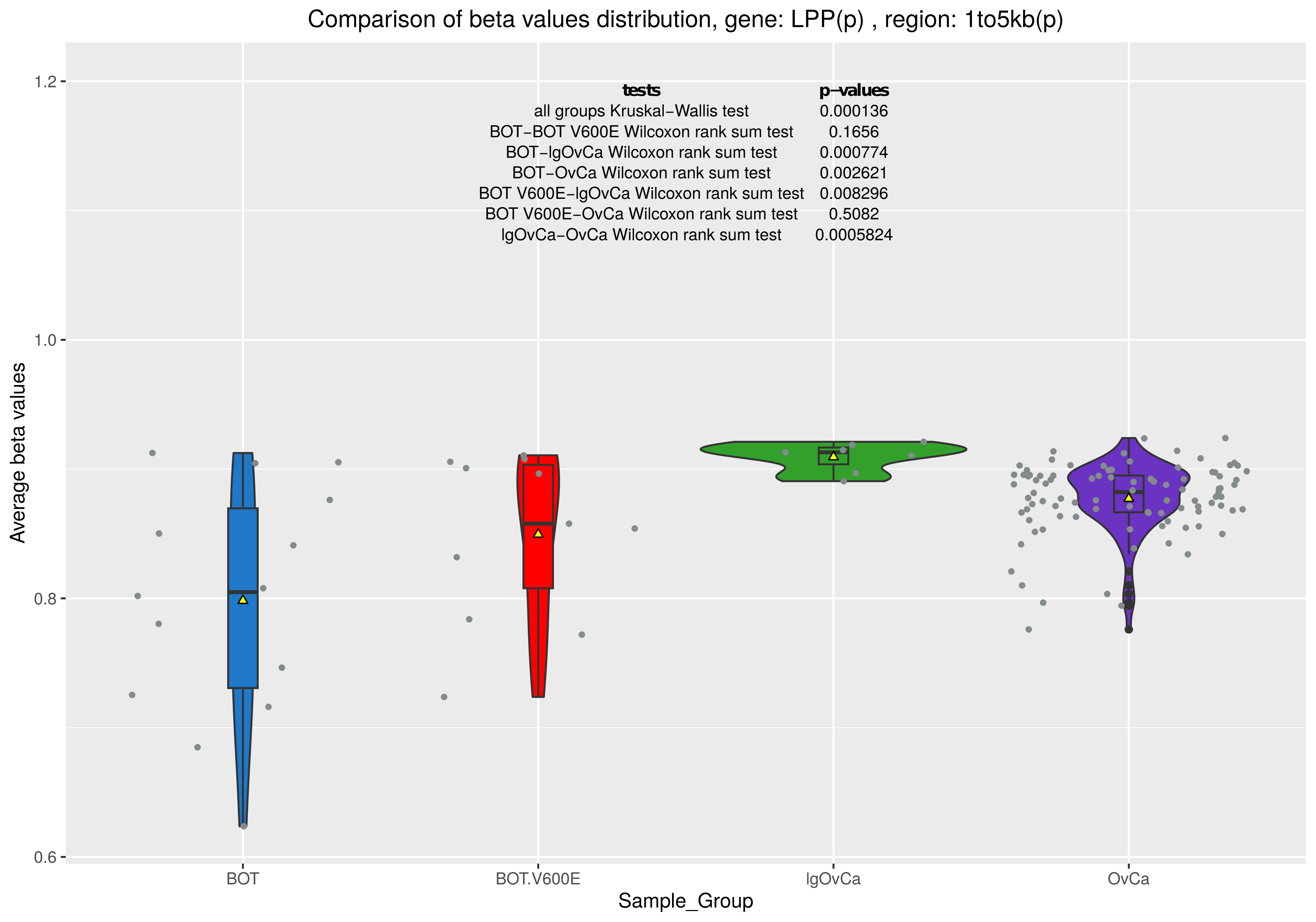

Comparison of beta values distribution, gene: LPP(p) , region: 1to5kb(p)
tests
p−values
all groups Kruskal−Wallis test
0.000136
BOT−BOT V600E Wilcoxon rank sum test
0.1656
BOT−IgOvCa Wilcoxon rank sum test
0.000774
BOT−OvCa Wilcoxon rank sum test
0.002621
BOT V600E−IgOvCa Wilcoxon rank sum test
0.008296
BOT V600E−OvCa Wilcoxon rank sum test
0.5082
IgOvCa−OvCa Wilcoxon rank sum test
0.0005824
Average beta values
1.2
1.0
0.8
0.6
BOT
BOT.V600E
IgOvCa
OvCa
Sample_Group

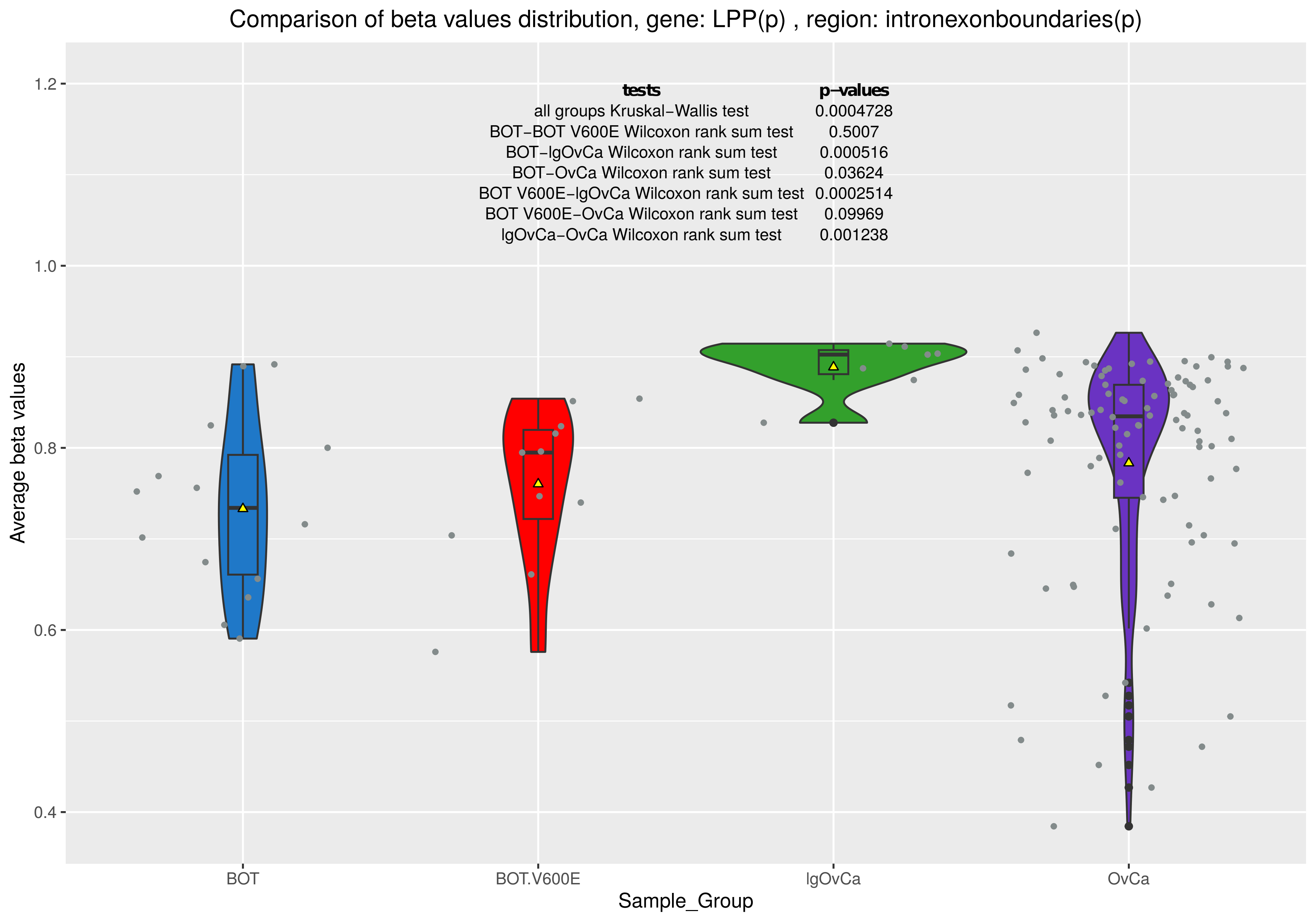

Comparison of beta values distribution, gene: LPP(p) , region: intronexonboundaries(p)
tests
p−values
all groups Kruskal−Wallis test
0.0004728
BOT−BOT V600E Wilcoxon rank sum test
0.5007
BOT−lgOvCa Wilcoxon rank sum test
0.000516
BOT−OvCa Wilcoxon rank sum test
0.03624
BOT V600E−lgOvCa Wilcoxon rank sum test
0.0002514
BOT V600E−OvCa Wilcoxon rank sum test
0.09969
lgOvCa−OvCa Wilcoxon rank sum test
0.001238
Average beta values
1.2
1.0
0.8
0.6
0.4
BOT
BOT.V600E
lgOvCa
OvCa
Sample_Group

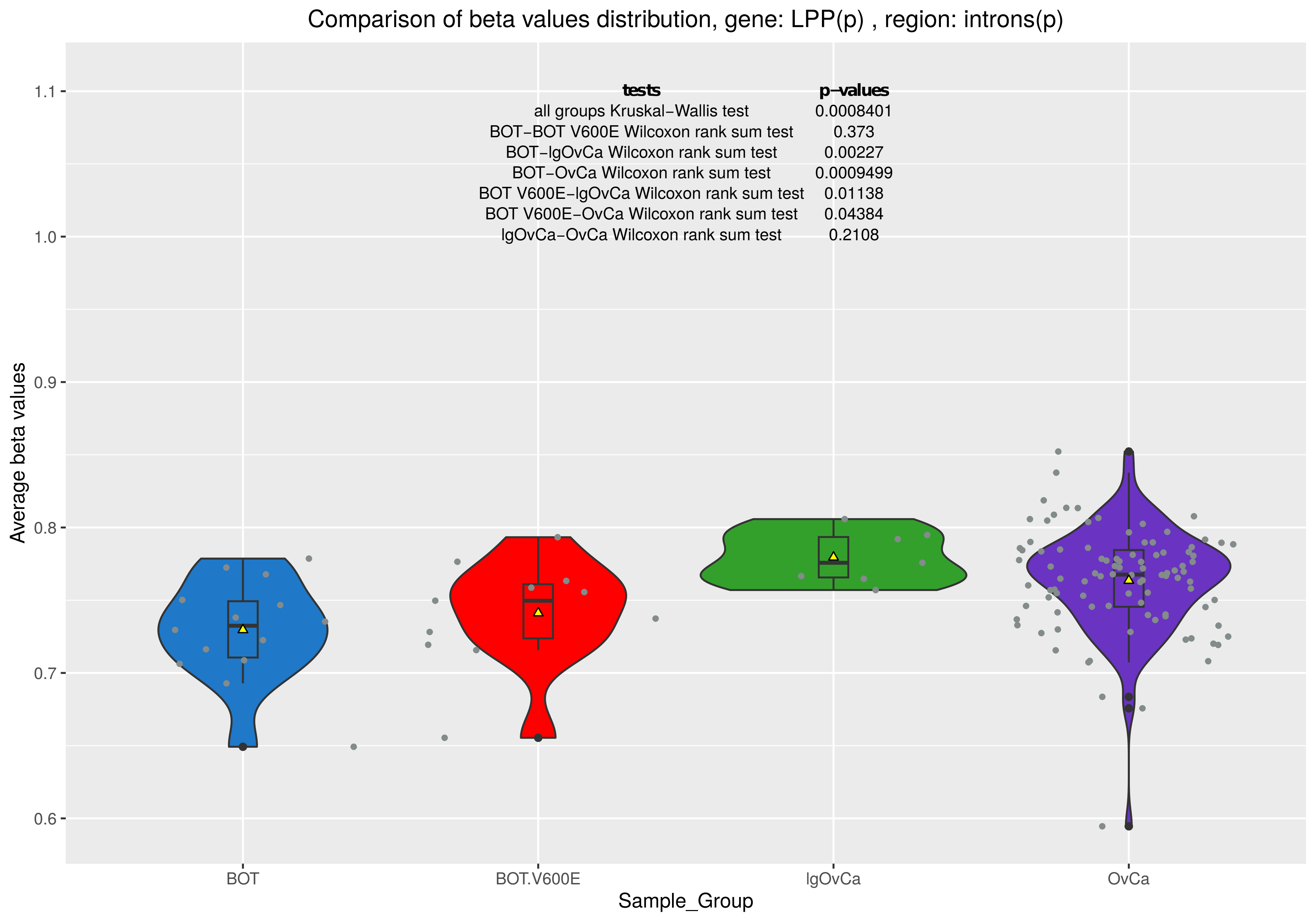

Comparison of beta values distribution, gene: LPP(p) , region: introns(p)
tests
p−values
all groups Kruskal−Wallis test
0.0008401
BOT−BOT V600E Wilcoxon rank sum test
0.373
BOT−IgOvCa Wilcoxon rank sum test
0.00227
BOT−OvCa Wilcoxon rank sum test
0.0009499
BOT V600E−IgOvCa Wilcoxon rank sum test
0.01138
BOT V600E−OvCa Wilcoxon rank sum test
0.04384
IgOvCa−OvCa Wilcoxon rank sum test
0.2108
Average beta values
1.1
1.0
0.9
0.8
0.7
0.6
BOT
BOT.V600E
IgOvCa
OvCa
Sample_Group

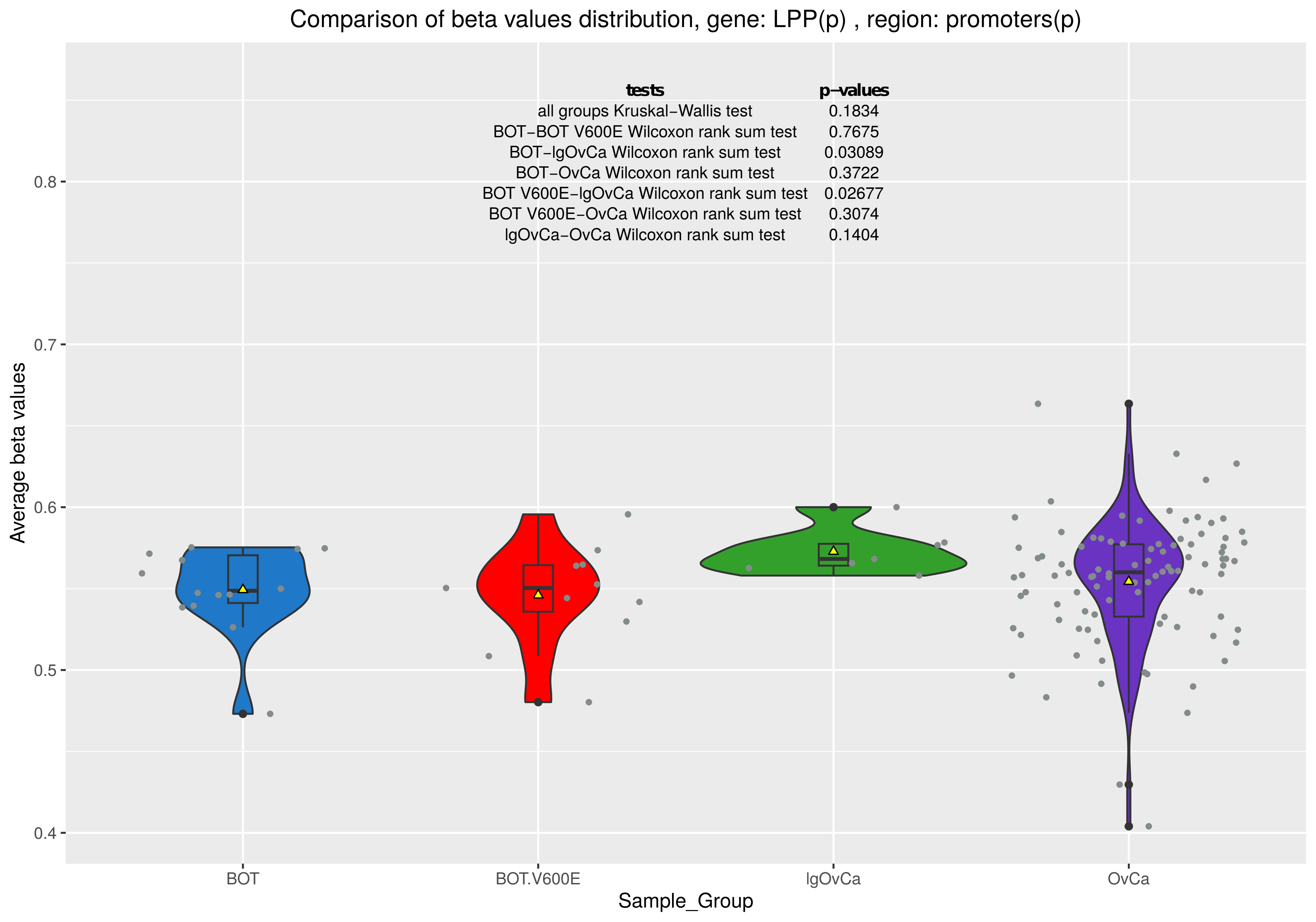

Comparison of beta values distribution, gene: LPP(p) , region: promoters(p)
tests
p−values
all groups Kruskal−Wallis test
0.1834
BOT−BOT V600E Wilcoxon rank sum test
0.7675
BOT−lgOvCa Wilcoxon rank sum test
0.03089
BOT−OvCa Wilcoxon rank sum test
0.3722
BOT V600E−lgOvCa Wilcoxon rank sum test
0.02677
BOT V600E−OvCa Wilcoxon rank sum test
0.3074
lgOvCa−OvCa Wilcoxon rank sum test
0.1404
0.8
0.7
0.6
0.5
0.4
Average beta values
BOT
BOT.V600E
lgOvCa
OvCa
Sample_Group

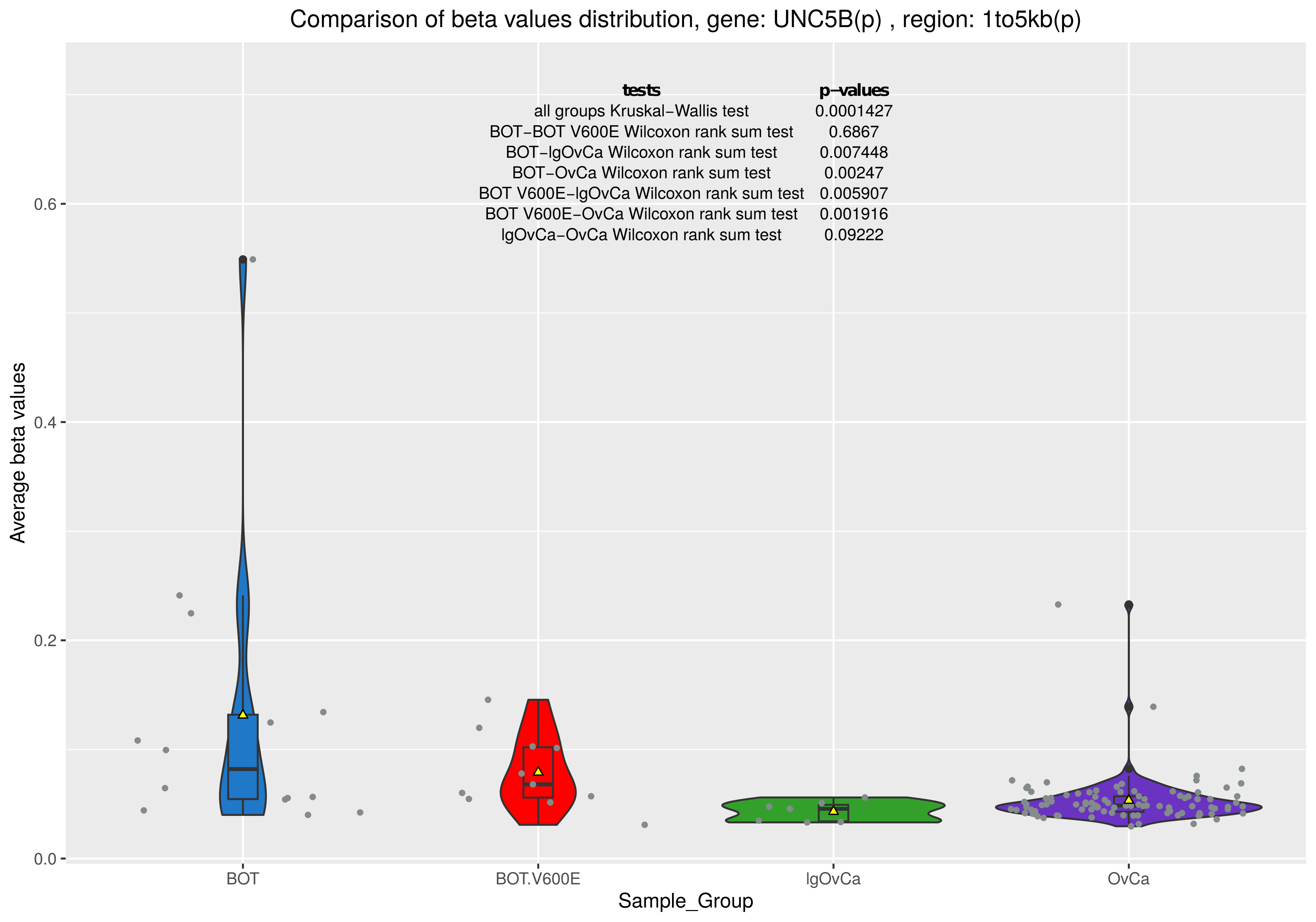

Comparison of beta values distribution, gene: UNC5B(p) , region: 1to5kb(p)
tests
p−values
all groups Kruskal−Wallis test
0.0001427
BOT−BOT V600E Wilcoxon rank sum test
0.6867
BOT−lgOvCa Wilcoxon rank sum test
0.007448
BOT−OvCa Wilcoxon rank sum test
0.00247
BOT V600E−lgOvCa Wilcoxon rank sum test
0.005907
BOT V600E−OvCa Wilcoxon rank sum test
0.001916
lgOvCa−OvCa Wilcoxon rank sum test
0.09222
Average beta values
0.6
0.4
0.2
0.0
BOT
BOT.V600E
lgOvCa
OvCa
Sample_Group

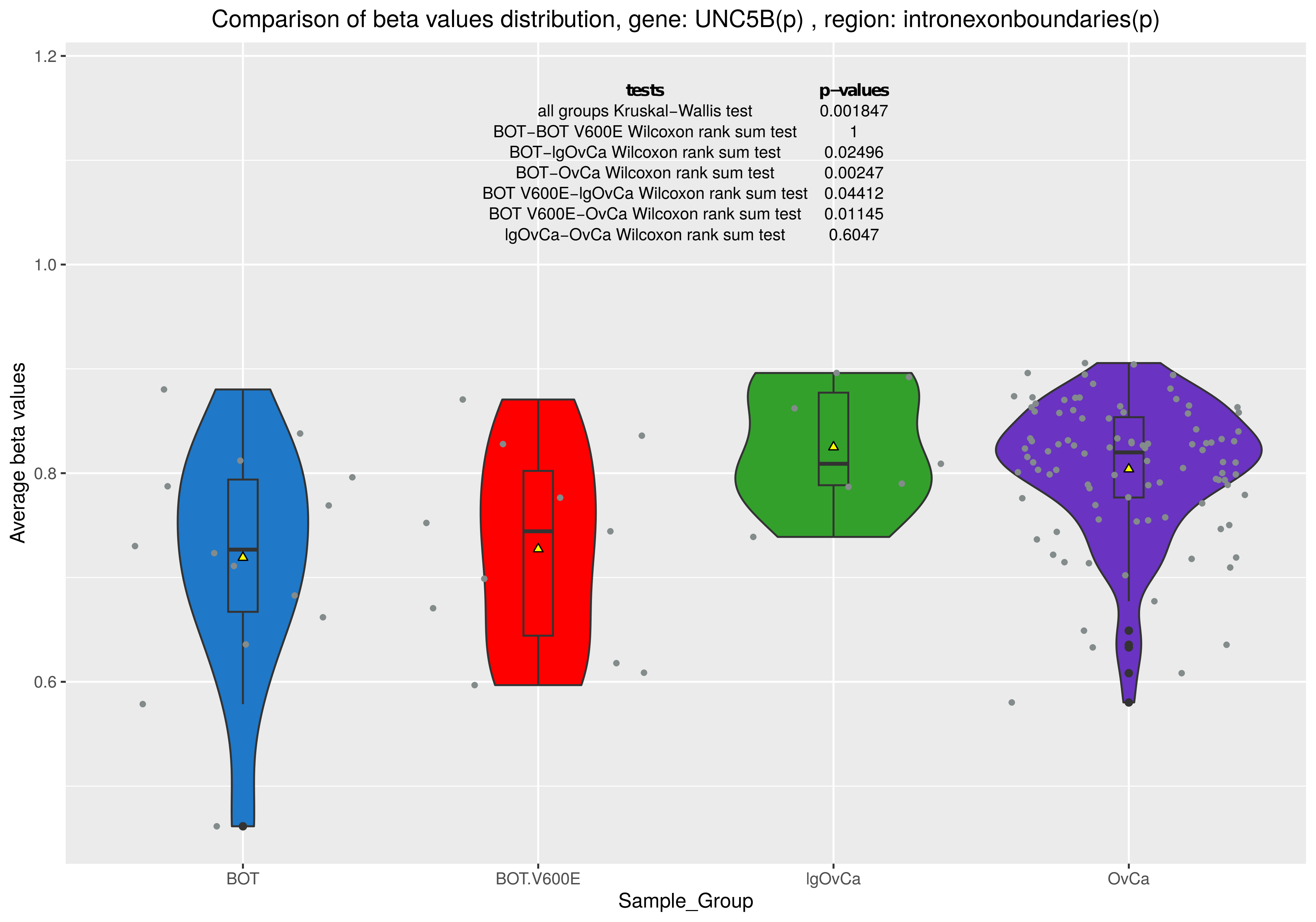
Comparison of beta values distribution, gene: UNC5B(p) , region: intronexonboundaries(p)
tests
p−values
all groups Kruskal−Wallis test
0.001847
BOT−BOT V600E Wilcoxon rank sum test
1
BOT−lgOvCa Wilcoxon rank sum test
0.02496
BOT−OvCa Wilcoxon rank sum test
0.00247
BOT V600E−lgOvCa Wilcoxon rank sum test
0.04412
BOT V600E−OvCa Wilcoxon rank sum test
0.01145
lgOvCa−OvCa Wilcoxon rank sum test
0.6047
Average beta values
1.2
1.0
0.8
0.6
BOT
BOT.V600E
lgOvCa
OvCa
Sample_Group

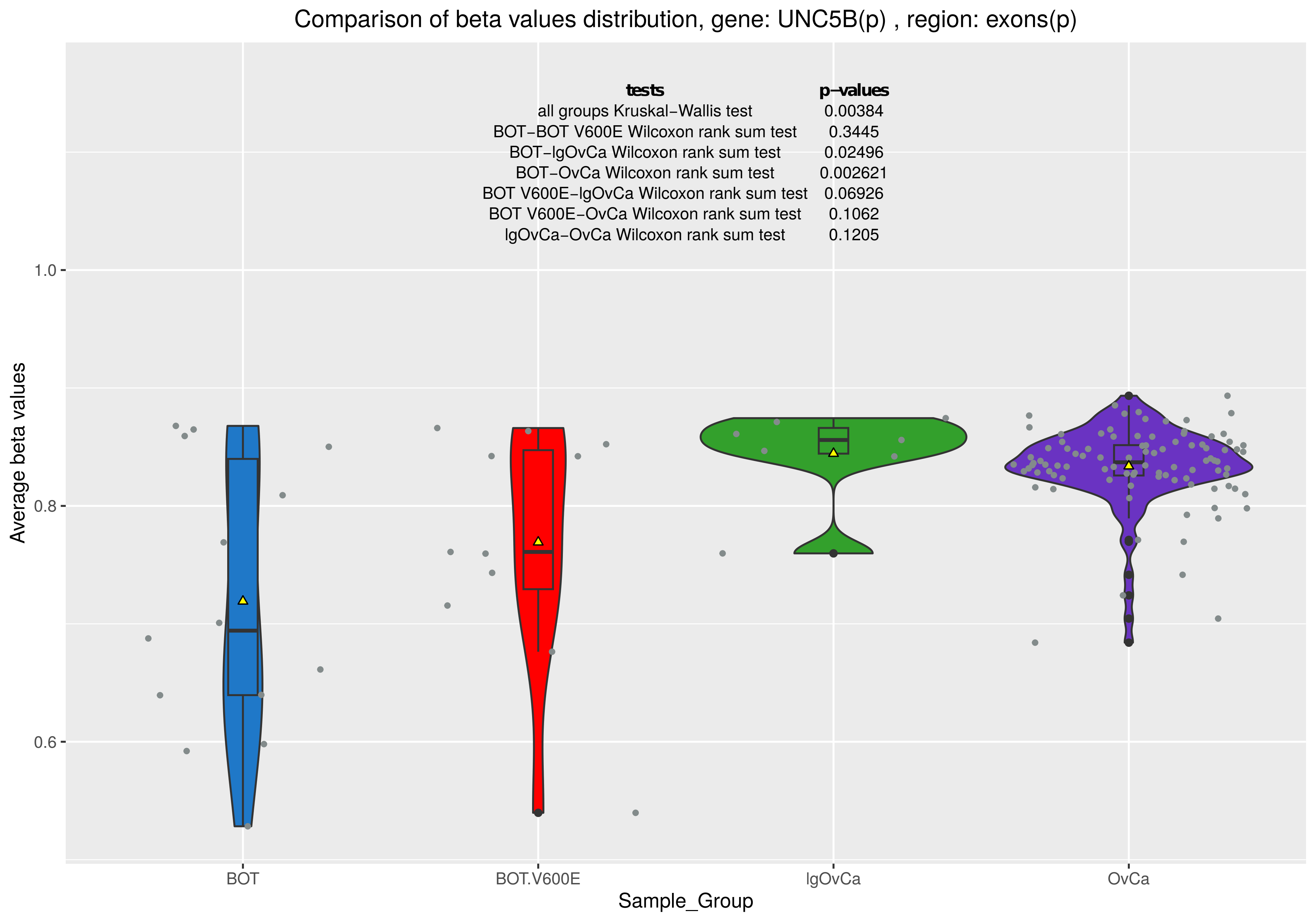
Comparison of beta values distribution, gene: UNC5B(p) , region: exons(p)
tests
p−values
all groups Kruskal−Wallis test
0.00384
BOT−BOT V600E Wilcoxon rank sum test
0.3445
BOT−IgOvCa Wilcoxon rank sum test
0.02496
BOT−OvCa Wilcoxon rank sum test
0.002621
BOT V600E−IgOvCa Wilcoxon rank sum test
0.06926
BOT V600E−OvCa Wilcoxon rank sum test
0.1062
IgOvCa−OvCa Wilcoxon rank sum test
0.1205
Average beta values
1.0
0.8
0.6
BOT
BOT.V600E
IgOvCa
OvCa
Sample_Group

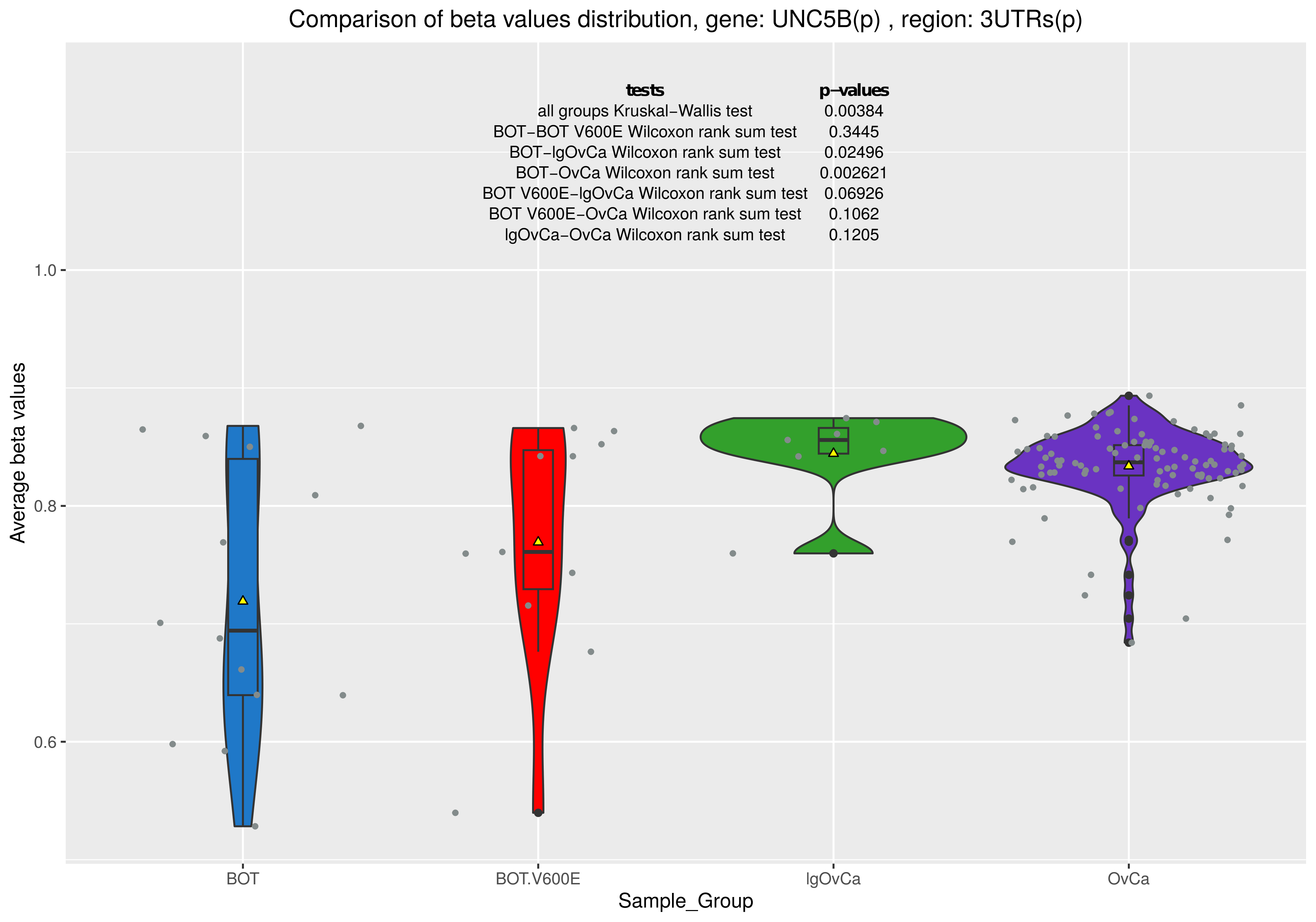
Comparison of beta values distribution, gene: UNC5B(p) , region: 3UTRs(p)
tests
p−values
all groups Kruskal−Wallis test
0.00384
BOT−BOT V600E Wilcoxon rank sum test
0.3445
BOT−lgOvCa Wilcoxon rank sum test
0.02496
BOT−OvCa Wilcoxon rank sum test
0.002621
BOT V600E−lgOvCa Wilcoxon rank sum test
0.06926
BOT V600E−OvCa Wilcoxon rank sum test
0.1062
lgOvCa−OvCa Wilcoxon rank sum test
0.1205
Average beta values
1.0
0.8
0.6
BOT
BOT.V600E
lgOvCa
OvCa
Sample_Group

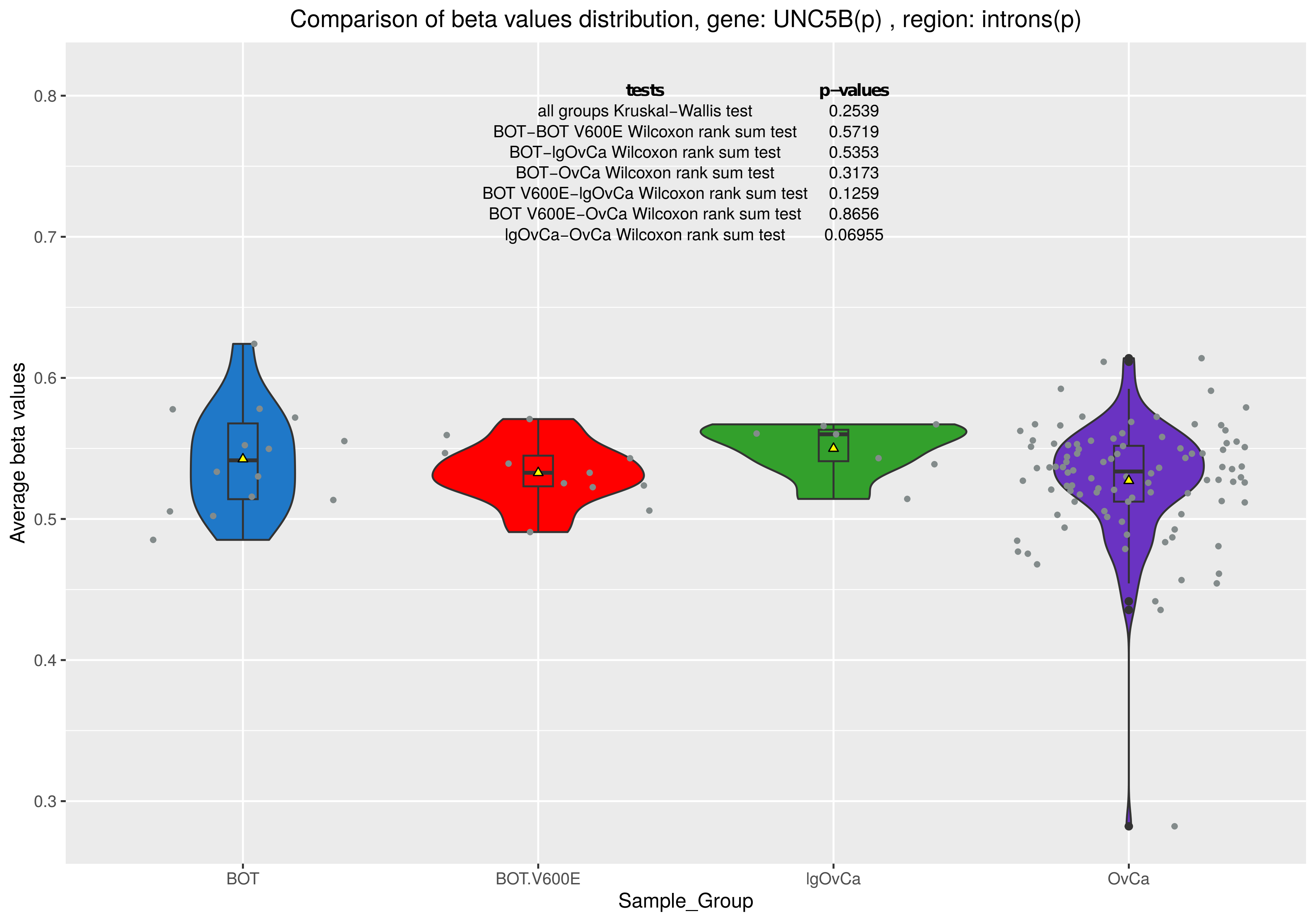

Comparison of beta values distribution, gene: UNC5B(p) , region: introns(p)
tests
p−values
all groups Kruskal−Wallis test
0.2539
BOT−BOT V600E Wilcoxon rank sum test
0.5719
BOT−lgOvCa Wilcoxon rank sum test
0.5353
BOT−OvCa Wilcoxon rank sum test
0.3173
BOT V600E−lgOvCa Wilcoxon rank sum test
0.1259
BOT V600E−OvCa Wilcoxon rank sum test
0.8656
lgOvCa−OvCa Wilcoxon rank sum test
0.06955
Average beta values
0.8
0.7
0.6
0.5
0.4
0.3
BOT
BOT.V600E
lgOvCa
OvCa
Sample_Group

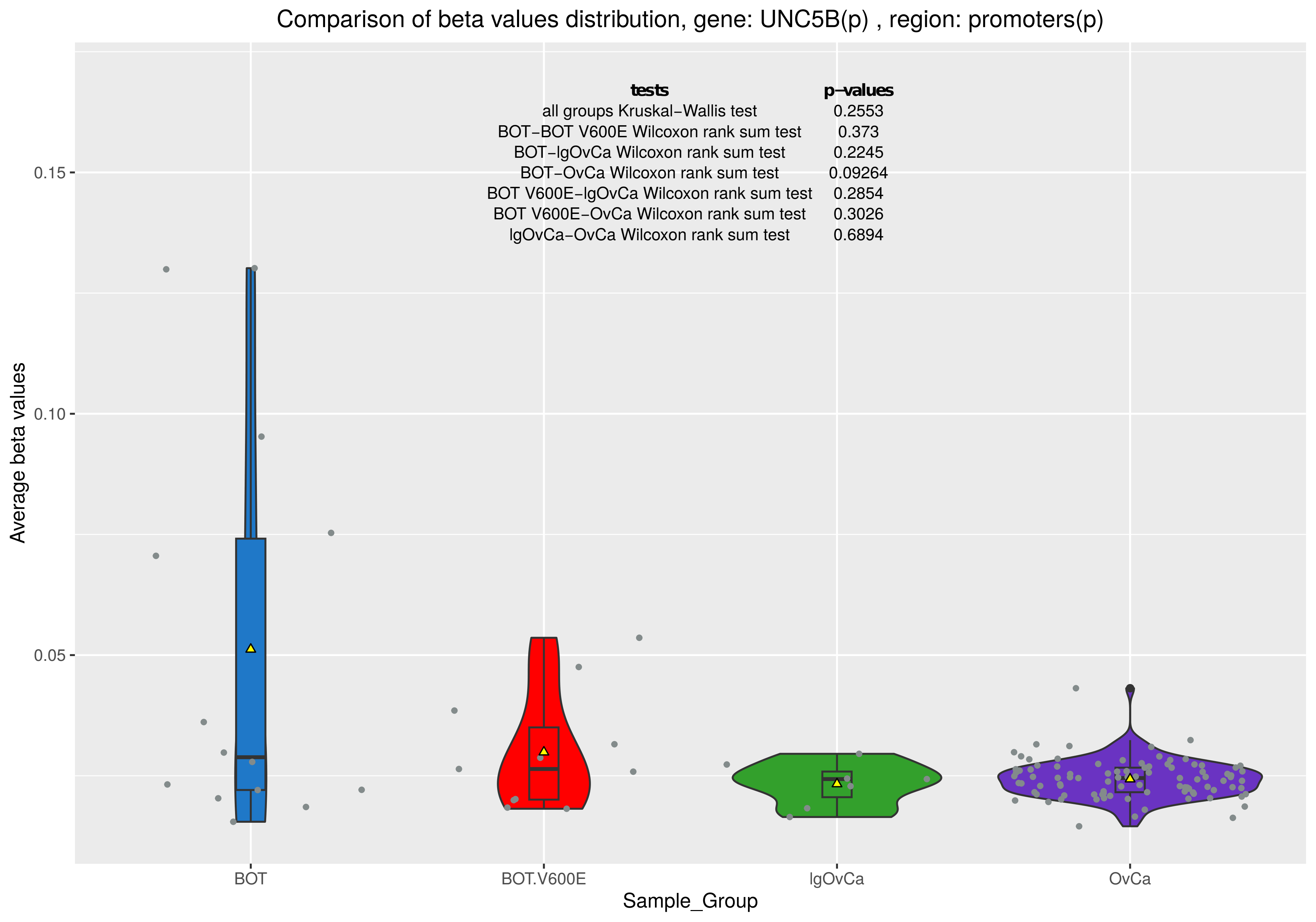

Comparison of beta values distribution, gene: UNC5B(p) , region: promoters(p)
tests
p−values
all groups Kruskal−Wallis test
0.2553
BOT−BOT V600E Wilcoxon rank sum test
0.373
BOT−IgOvCa Wilcoxon rank sum test
0.2245
BOT−OvCa Wilcoxon rank sum test
0.09264
BOT V600E−IgOvCa Wilcoxon rank sum test
0.2854
BOT V600E−OvCa Wilcoxon rank sum test
0.3026
IgOvCa−OvCa Wilcoxon rank sum test
0.6894
Average beta values
0.15
0.10
0.05
BOT
BOT.V600E
IgOvCa
OvCa
Sample_Group

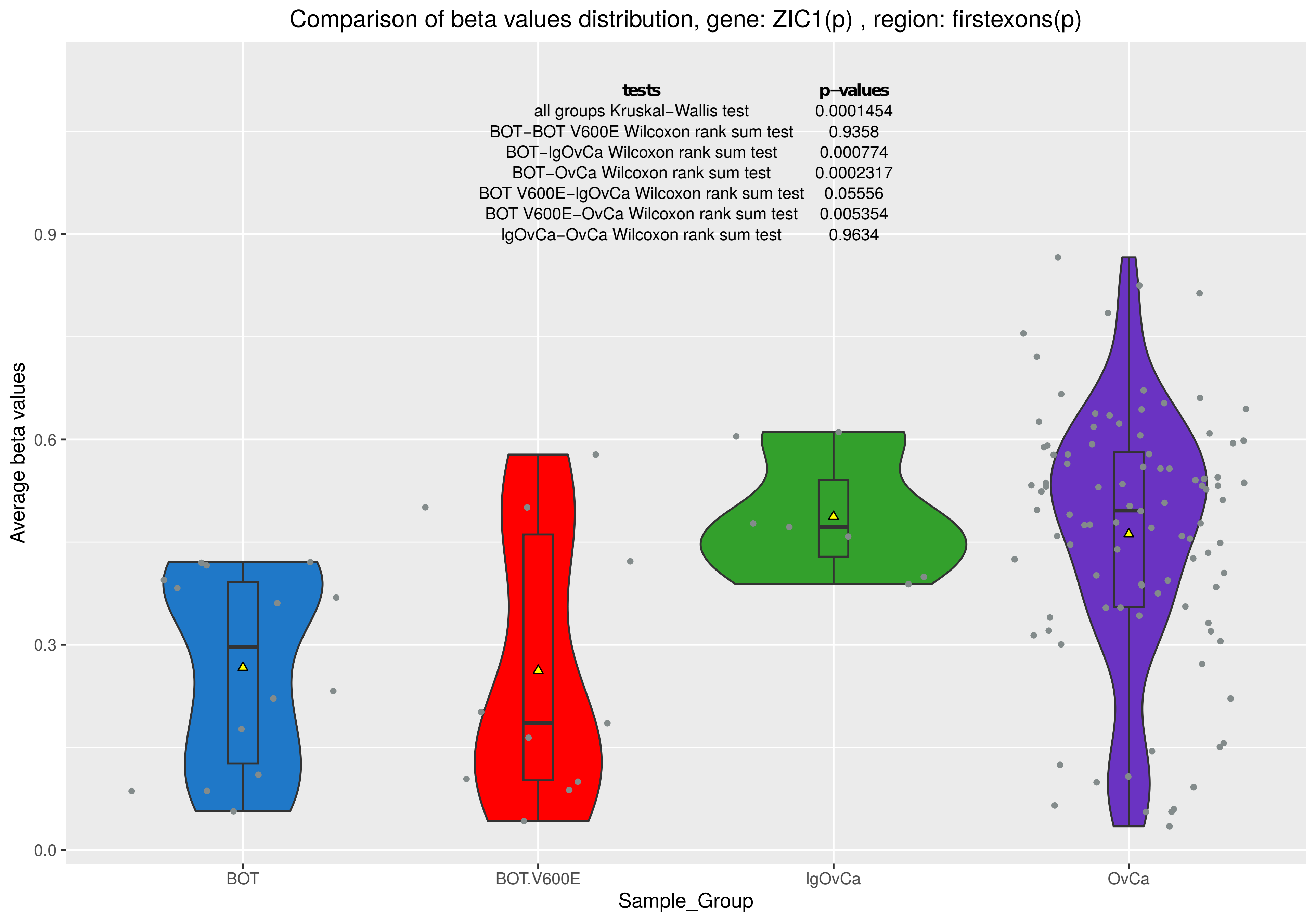
Comparison of beta values distribution, gene: ZIC1(p) , region: firstexons(p)
tests
p−values
all groups Kruskal−Wallis test
0.0001454
BOT−BOT V600E Wilcoxon rank sum test
0.9358
BOT−lgOvCa Wilcoxon rank sum test
0.000774
BOT−OvCa Wilcoxon rank sum test
0.0002317
BOT V600E−lgOvCa Wilcoxon rank sum test
0.05556
BOT V600E−OvCa Wilcoxon rank sum test
0.005354
lgOvCa−OvCa Wilcoxon rank sum test
0.9634
Average beta values
0.9
0.6
0.3
0.0
BOT
BOT.V600E
lgOvCa
OvCa
Sample_Group

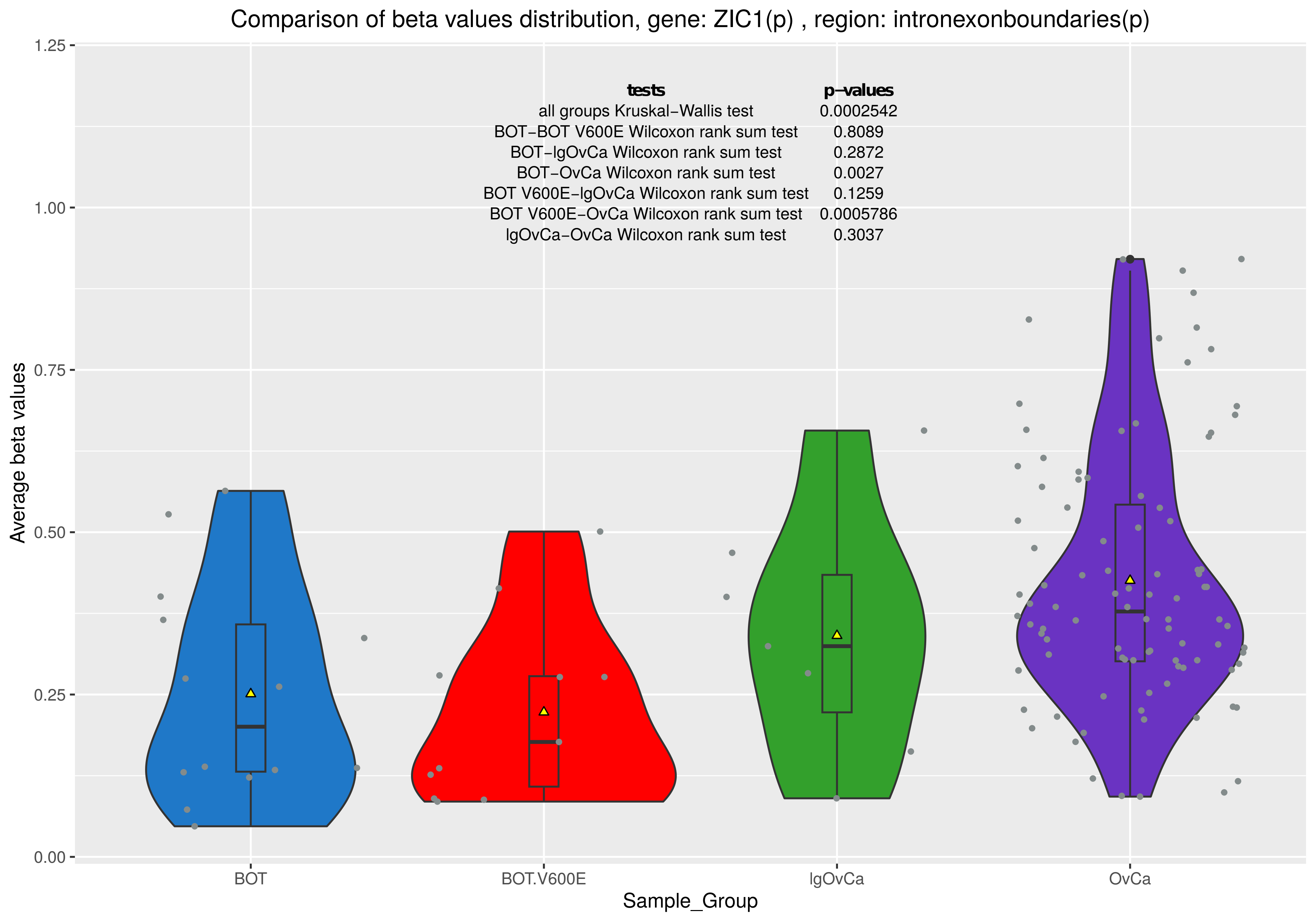

Comparison of beta values distribution, gene: ZIC1(p) , region: intronexonboundaries(p)
tests
p−values
all groups Kruskal−Wallis test
0.0002542
BOT−BOT V600E Wilcoxon rank sum test
0.8089
BOT−lgOvCa Wilcoxon rank sum test
0.2872
BOT−OvCa Wilcoxon rank sum test
0.0027
BOT V600E−lgOvCa Wilcoxon rank sum test
0.1259
BOT V600E−OvCa Wilcoxon rank sum test
0.0005786
lgOvCa−OvCa Wilcoxon rank sum test
0.3037
Average beta values
1.25
1.00
0.75
0.50
0.25
0.00
BOT
BOT.V600E
lgOvCa
OvCa
Sample_Group

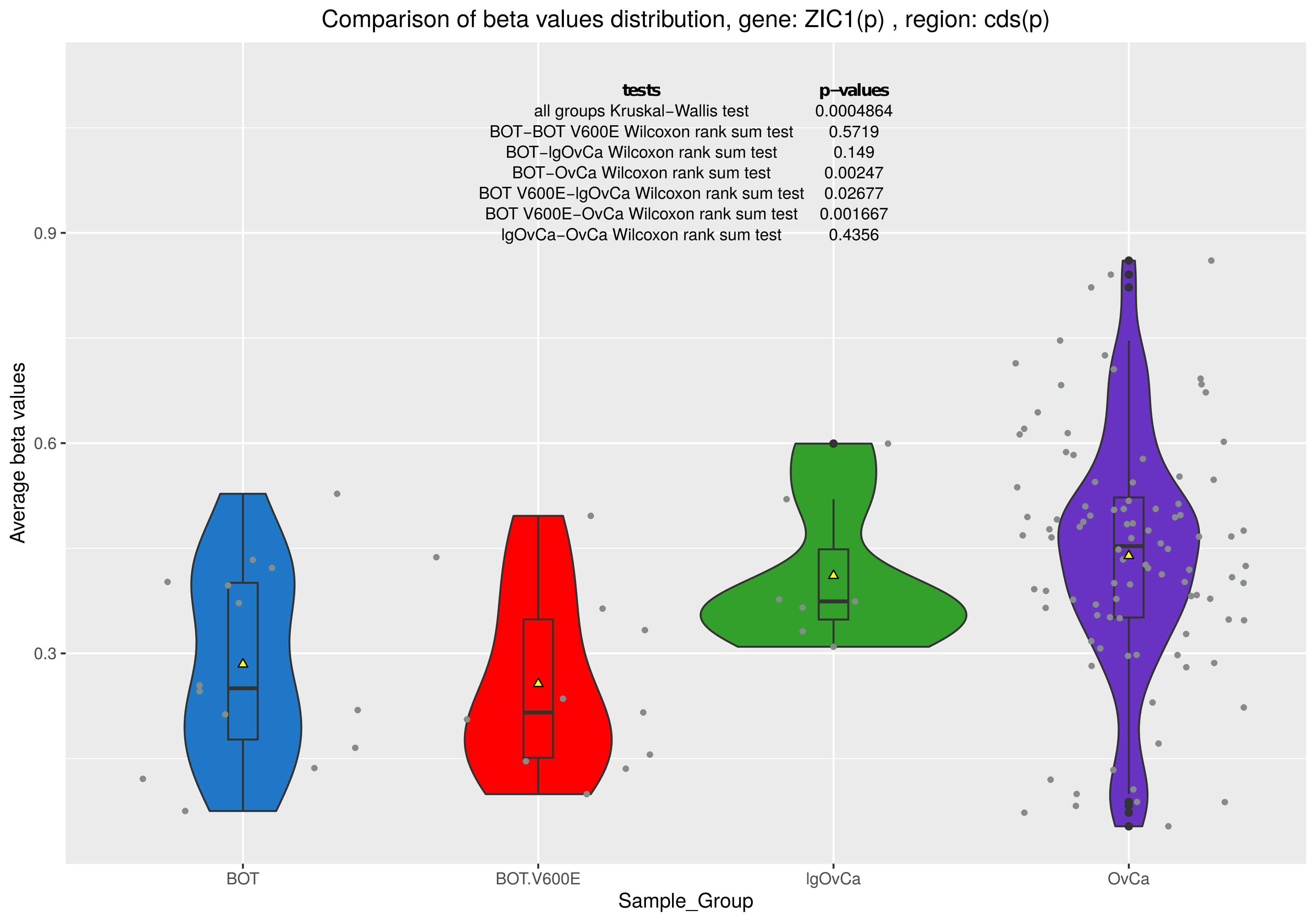
Comparison of beta values distribution, gene: ZIC1(p) , region: cds(p)
tests
p−values
all groups Kruskal−Wallis test
0.0004864
BOT−BOT V600E Wilcoxon rank sum test
0.5719
BOT−lgOvCa Wilcoxon rank sum test
0.149
BOT−OvCa Wilcoxon rank sum test
0.00247
BOT V600E−lgOvCa Wilcoxon rank sum test
0.02677
BOT V600E−OvCa Wilcoxon rank sum test
0.001667
lgOvCa−OvCa Wilcoxon rank sum test
0.4356
Average beta values
0.9
0.6
0.3
BOT
BOT.V600E
lgOvCa
OvCa
Sample_Group

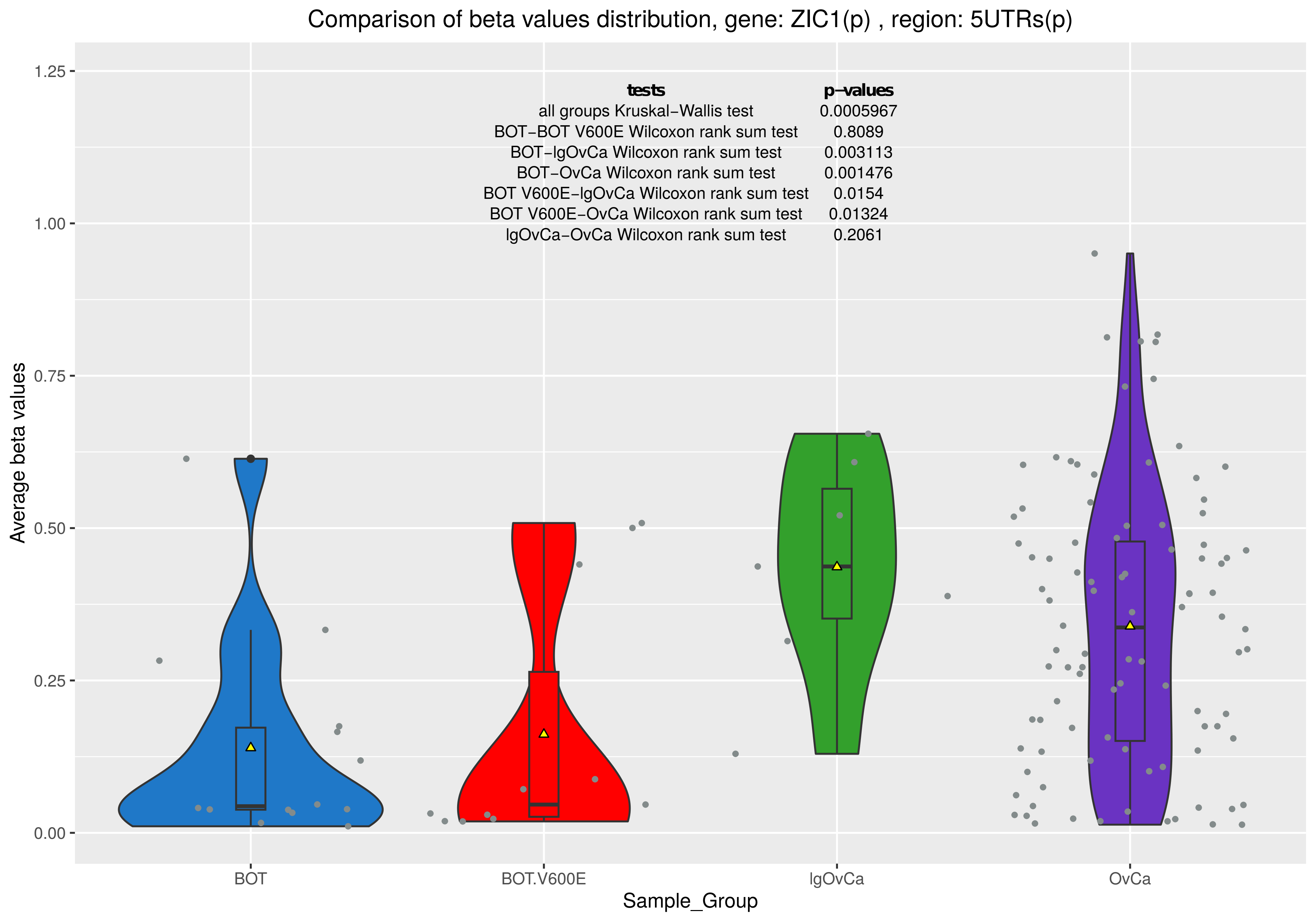

Comparison of beta values distribution, gene: ZIC1(p) , region: 5UTRs(p)
tests
p−values
all groups Kruskal−Wallis test
0.0005967
BOT−BOT V600E Wilcoxon rank sum test
0.8089
BOT−lgOvCa Wilcoxon rank sum test
0.003113
BOT−OvCa Wilcoxon rank sum test
0.001476
BOT V600E−lgOvCa Wilcoxon rank sum test
0.0154
BOT V600E−OvCa Wilcoxon rank sum test
0.01324
lgOvCa−OvCa Wilcoxon rank sum test
0.2061
Average beta values
1.25
1.00
0.75
0.50
0.25
0.00
BOT
BOT.V600E
lgOvCa
OvCa
Sample_Group

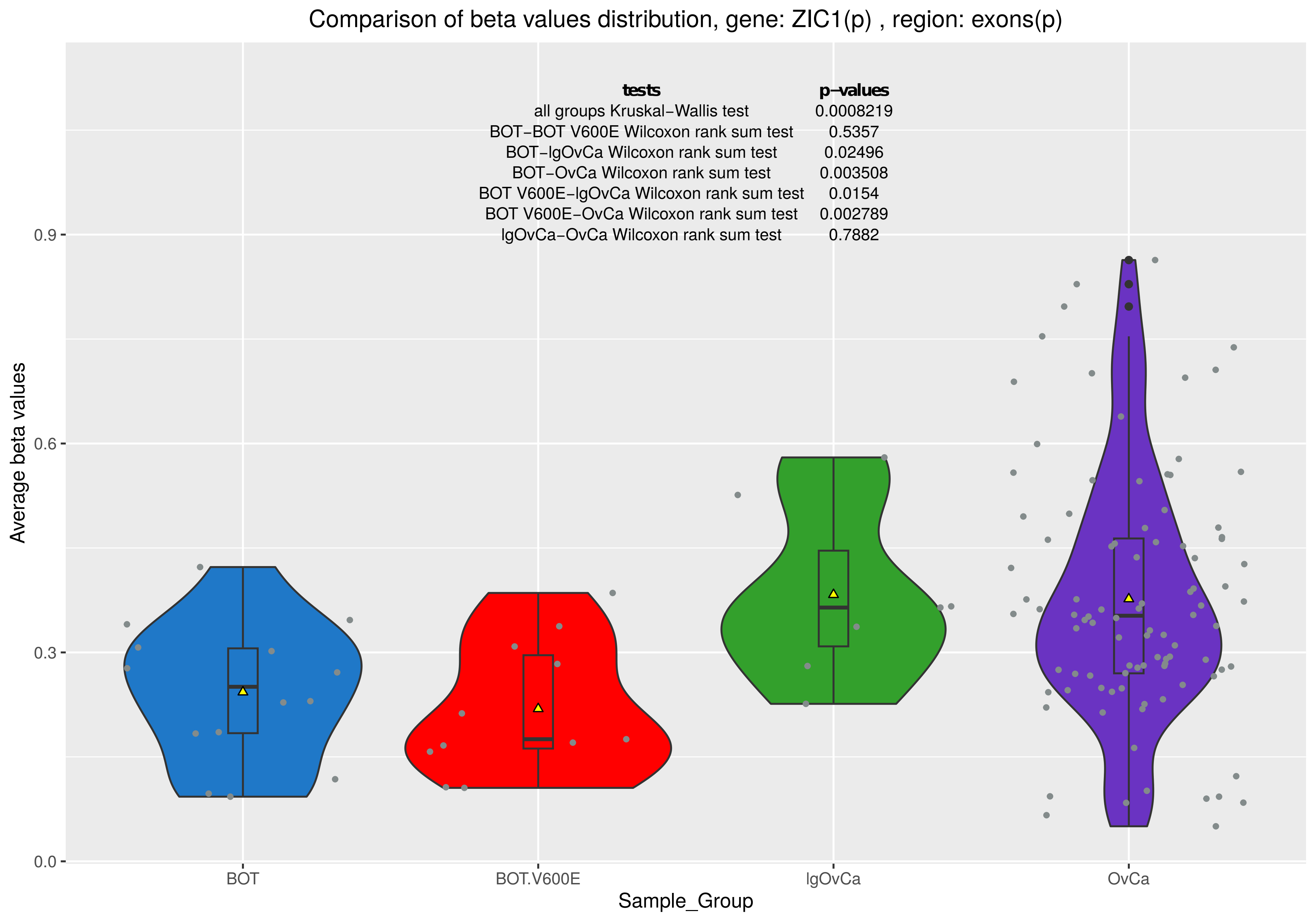

Comparison of beta values distribution, gene: ZIC1(p) , region: exons(p)
tests
p−values
all groups Kruskal−Wallis test 0.0008219
BOT−BOT V600E Wilcoxon rank sum test 0.5357
BOT−lgOvCa Wilcoxon rank sum test 0.02496
BOT−OvCa Wilcoxon rank sum test 0.003508
BOT V600E−lgOvCa Wilcoxon rank sum test 0.0154
BOT V600E−OvCa Wilcoxon rank sum test 0.002789
lgOvCa−OvCa Wilcoxon rank sum test 0.7882
Average beta values
0.9
0.6
0.3
0.0
BOT
BOT.V600E
lgOvCa
OvCa
Sample_Group

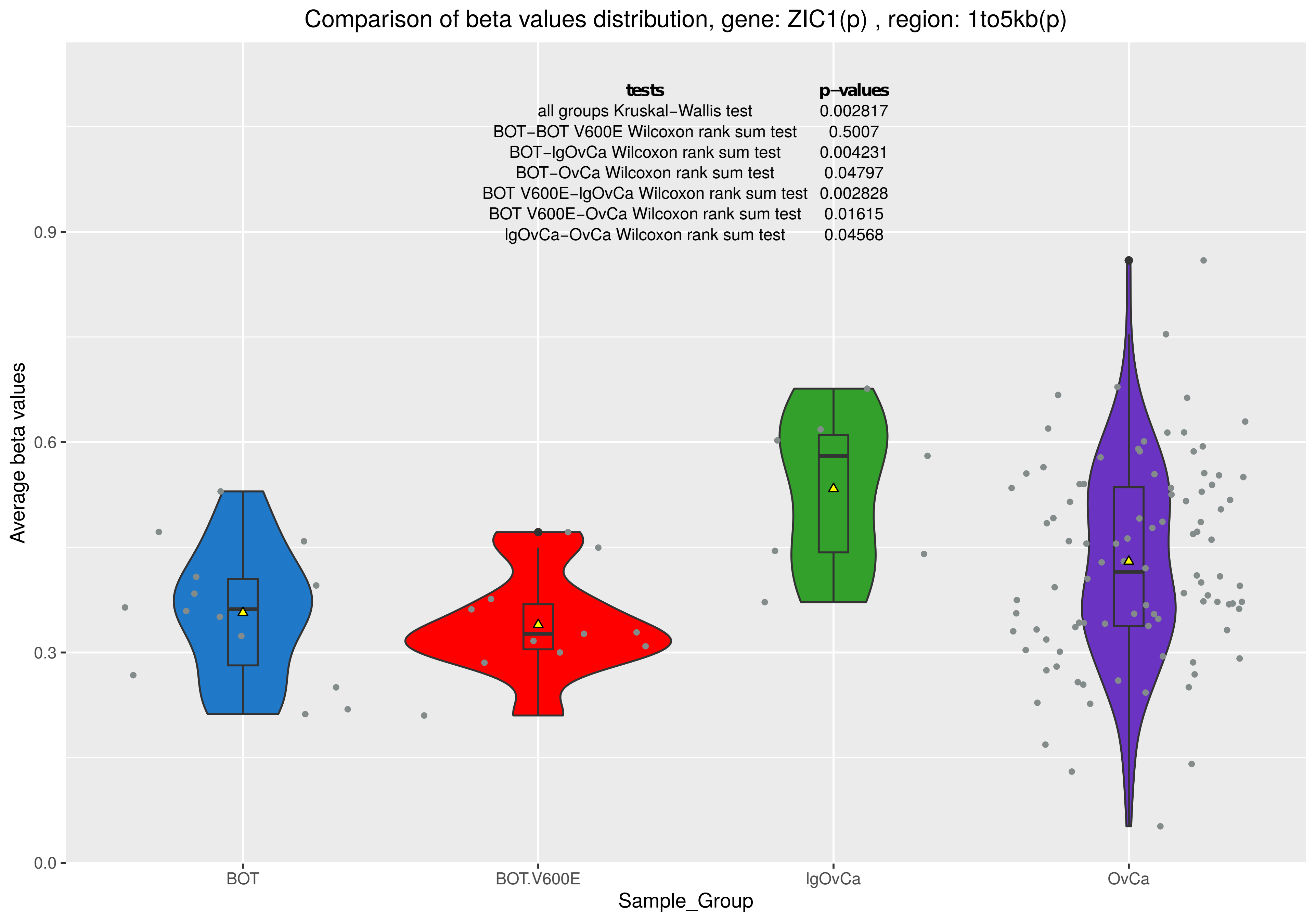

Comparison of beta values distribution, gene: ZIC1(p) , region: 1to5kb(p)
tests
p−values
all groups Kruskal−Wallis test
0.002817
BOT−BOT V600E Wilcoxon rank sum test
0.5007
BOT−lgOvCa Wilcoxon rank sum test
0.004231
BOT−OvCa Wilcoxon rank sum test
0.04797
BOT V600E−lgOvCa Wilcoxon rank sum test
0.002828
BOT V600E−OvCa Wilcoxon rank sum test
0.01615
lgOvCa−OvCa Wilcoxon rank sum test
0.04568
Average beta values
0.0
0.3
0.6
0.9
BOT
BOT.V600E
lgOvCa
OvCa
Sample_Group

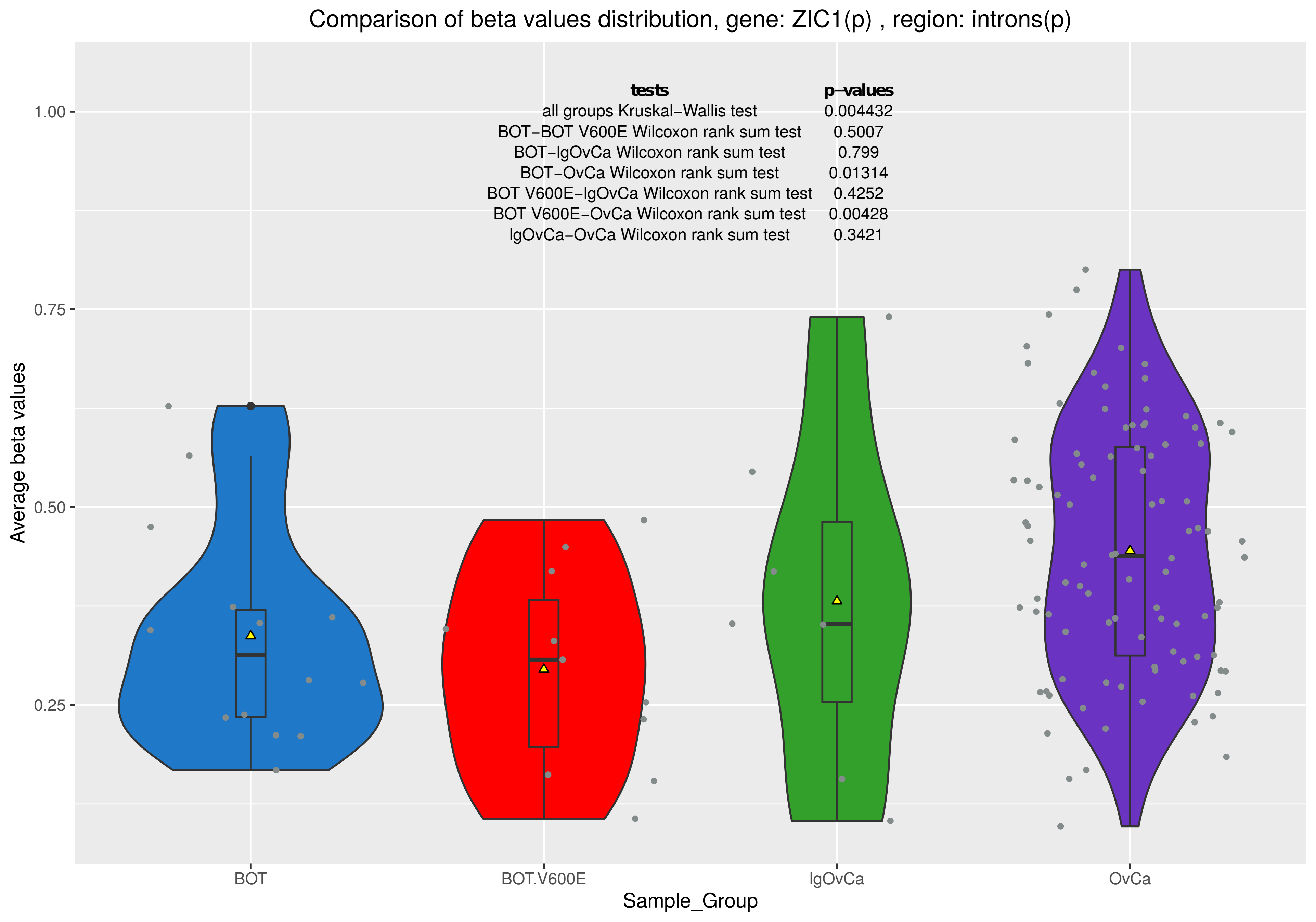

Comparison of beta values distribution, gene: ZIC1(p) , region: introns(p)
tests
p−values
all groups Kruskal−Wallis test
0.004432
BOT−BOT V600E Wilcoxon rank sum test
0.5007
BOT−lgOvCa Wilcoxon rank sum test
0.799
BOT−OvCa Wilcoxon rank sum test
0.01314
BOT V600E−lgOvCa Wilcoxon rank sum test
0.4252
BOT V600E−OvCa Wilcoxon rank sum test
0.00428
lgOvCa−OvCa Wilcoxon rank sum test
0.3421
Average beta values
1.00
0.75
0.50
0.25
BOT
BOT.V600E
lgOvCa
OvCa
Sample_Group

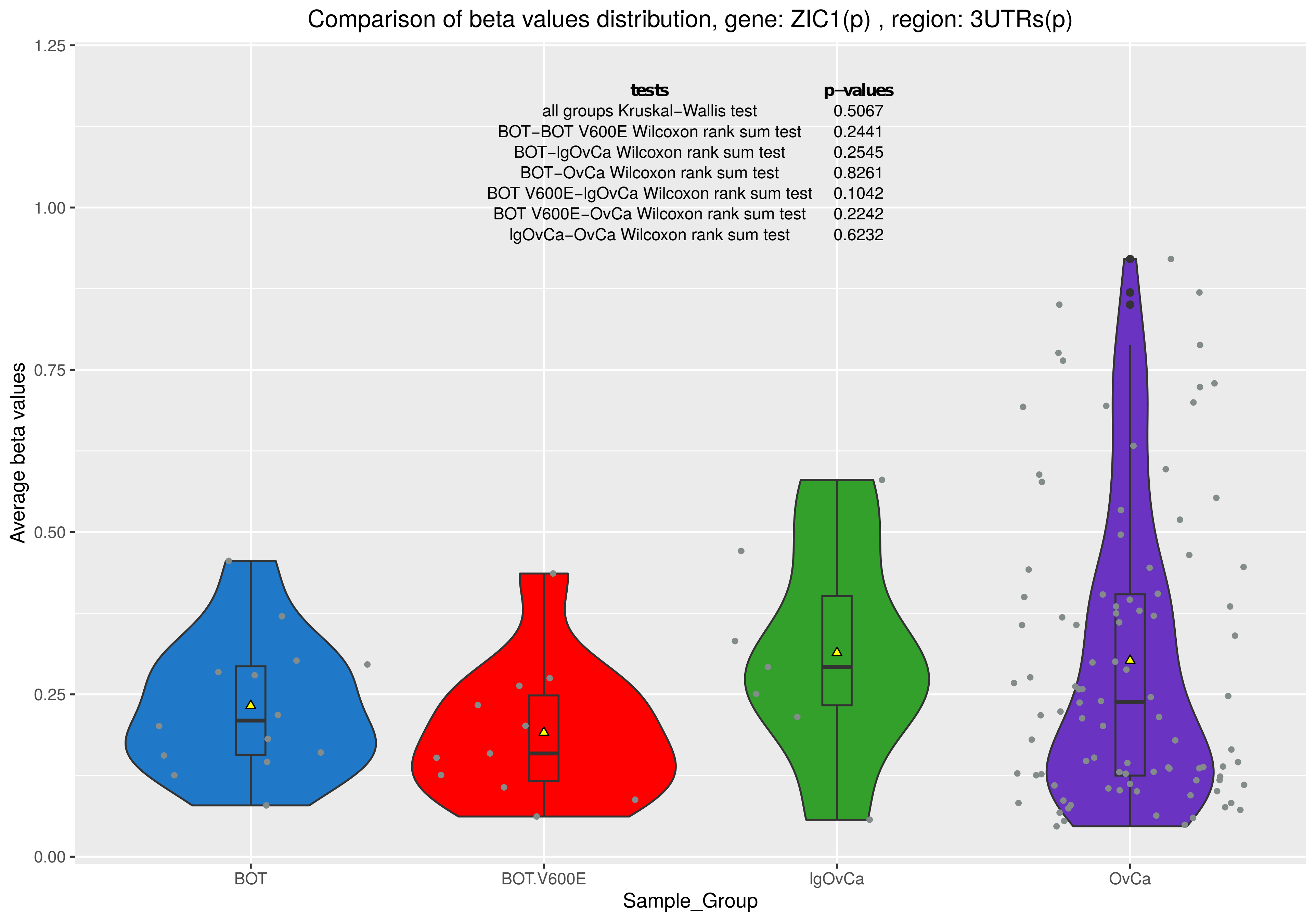
Comparison of beta values distribution, gene: ZIC1(p) , region: 3UTRs(p)
tests
p−values
all groups Kruskal−Wallis test
0.5067
BOT−BOT V600E Wilcoxon rank sum test
0.2441
BOT−lgOvCa Wilcoxon rank sum test
0.2545
BOT−OvCa Wilcoxon rank sum test
0.8261
BOT V600E−lgOvCa Wilcoxon rank sum test
0.1042
BOT V600E−OvCa Wilcoxon rank sum test
0.2242
lgOvCa−OvCa Wilcoxon rank sum test
0.6232
Average beta values
1.25
1.00
0.75
0.50
0.25
0.00
BOT
BOT.V600E
lgOvCa
OvCa
Sample_Group

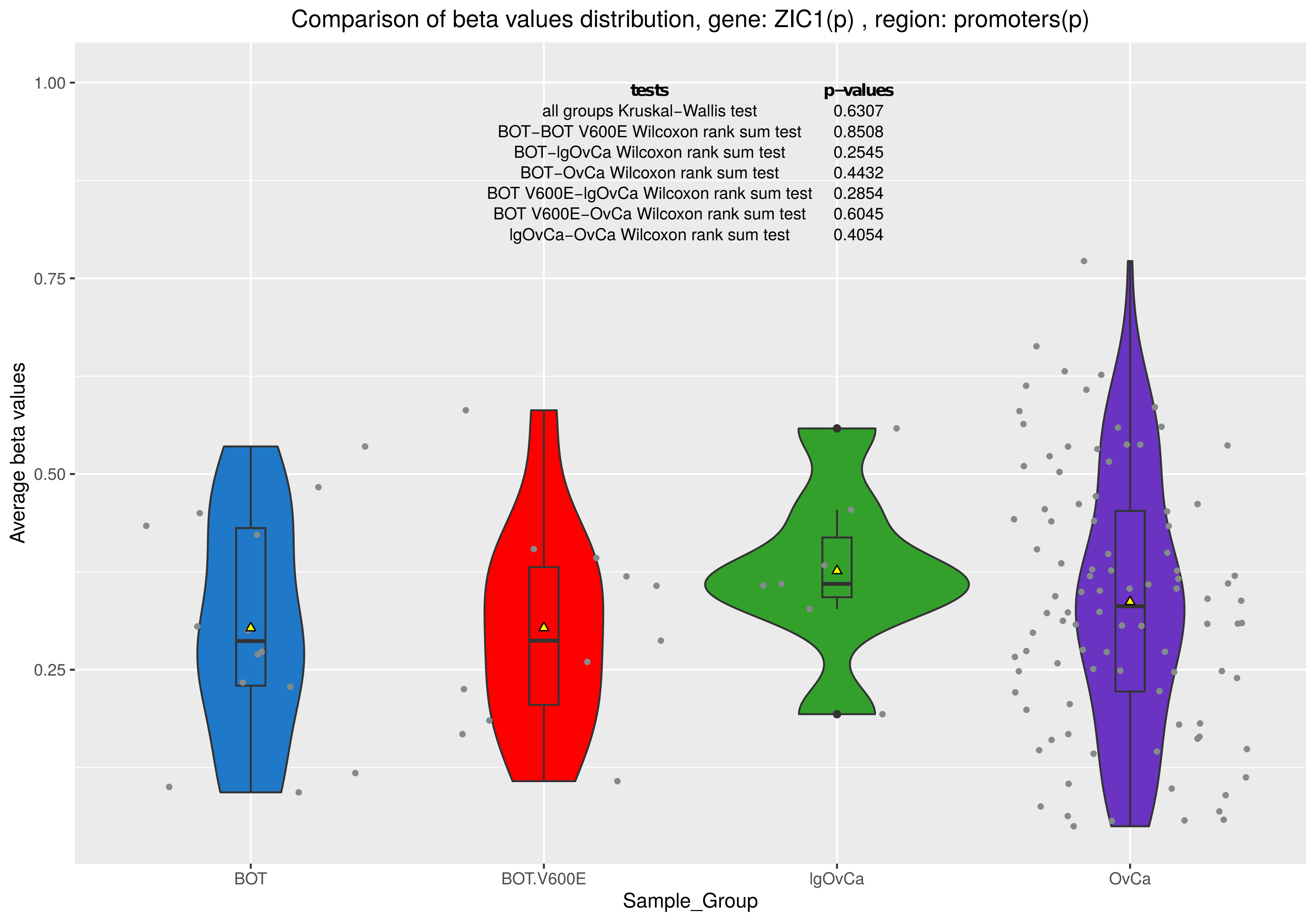
Comparison of beta values distribution, gene: ZIC1(p) , region: promoters(p)
tests
p−values
all groups Kruskal−Wallis test
0.6307
BOT−BOT V600E Wilcoxon rank sum test
0.8508
BOT−lgOvCa Wilcoxon rank sum test
0.2545
BOT−OvCa Wilcoxon rank sum test
0.4432
BOT V600E−lgOvCa Wilcoxon rank sum test
0.2854
BOT V600E−OvCa Wilcoxon rank sum test
0.6045
lgOvCa−OvCa Wilcoxon rank sum test
0.4054
Average beta values
1.00
0.75
0.50
0.25
BOT
BOT.V600E
lgOvCa
OvCa
Sample_Group

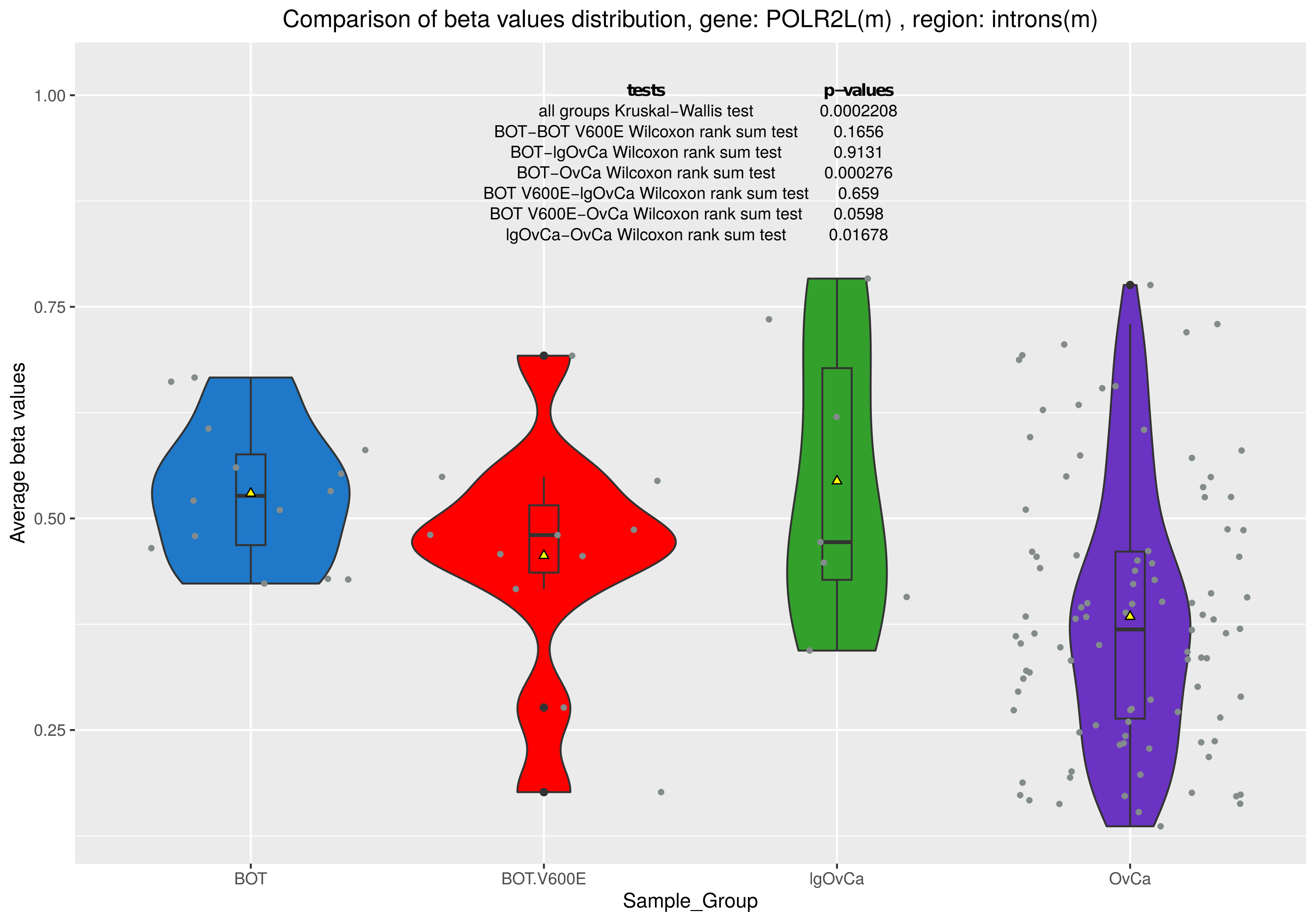
Comparison of beta values distribution, gene: POLR2L(m) , region: introns(m)
tests
p−values
all groups Kruskal−Wallis test
0.0002208
BOT−BOT V600E Wilcoxon rank sum test
0.1656
BOT−IgOvCa Wilcoxon rank sum test
0.9131
BOT−OvCa Wilcoxon rank sum test
0.000276
BOT V600E−IgOvCa Wilcoxon rank sum test
0.659
BOT V600E−OvCa Wilcoxon rank sum test
0.0598
IgOvCa−OvCa Wilcoxon rank sum test
0.01678
Average beta values
1.00
0.75
0.50
0.25
BOT
BOT.V600E
IgOvCa
OvCa
Sample_Group

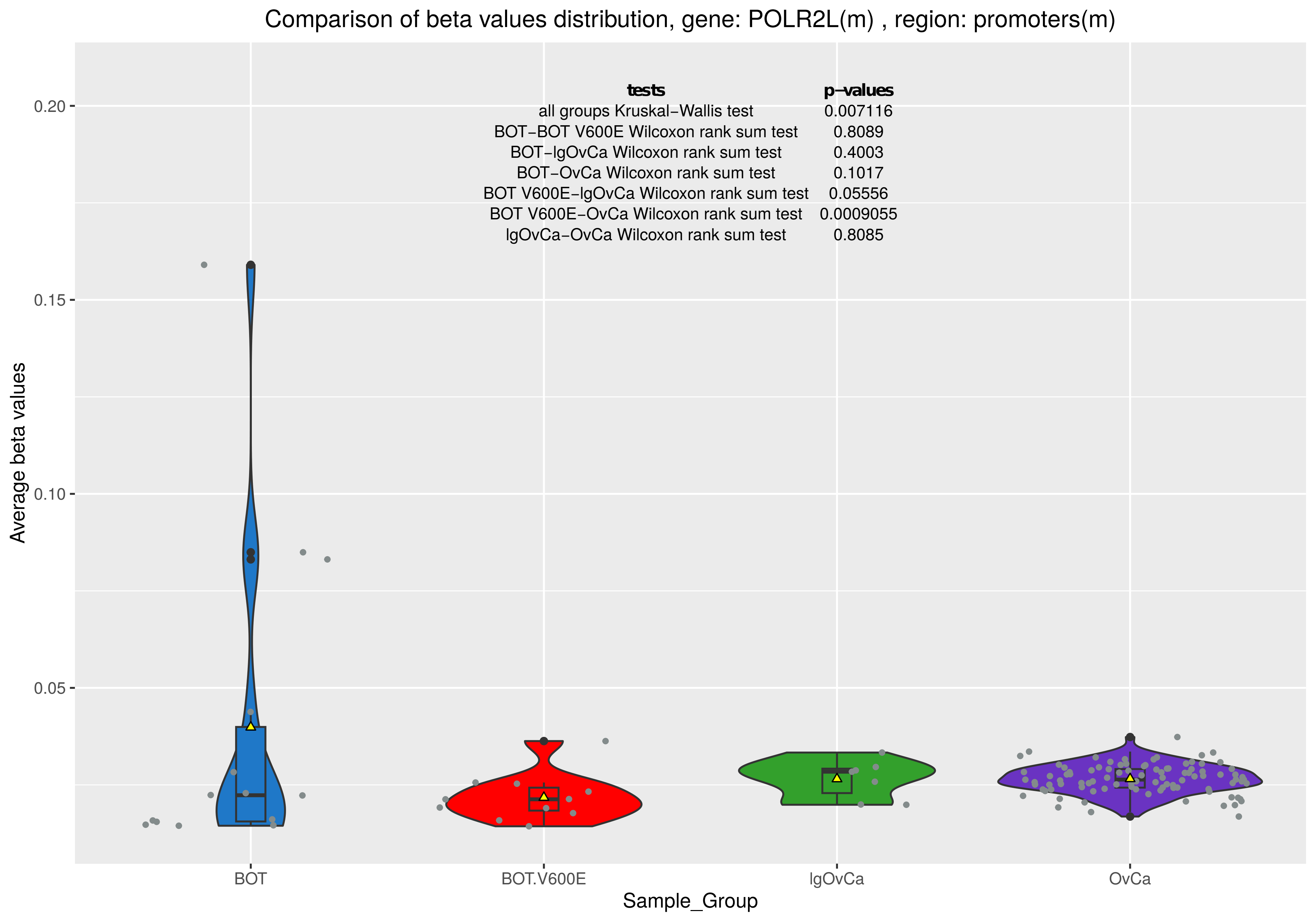

Comparison of beta values distribution, gene: POLR2L(m) , region: promoters(m)
tests
p−values
all groups Kruskal−Wallis test
0.007116
BOT−BOT V600E Wilcoxon rank sum test
0.8089
BOT−lgOvCa Wilcoxon rank sum test
0.4003
BOT−OvCa Wilcoxon rank sum test
0.1017
BOT V600E−lgOvCa Wilcoxon rank sum test
0.05556
BOT V600E−OvCa Wilcoxon rank sum test
0.0009055
lgOvCa−OvCa Wilcoxon rank sum test
0.8085
Average beta values
0.20
0.15
0.10
0.05
BOT
BOT.V600E
lgOvCa
OvCa
Sample_Group

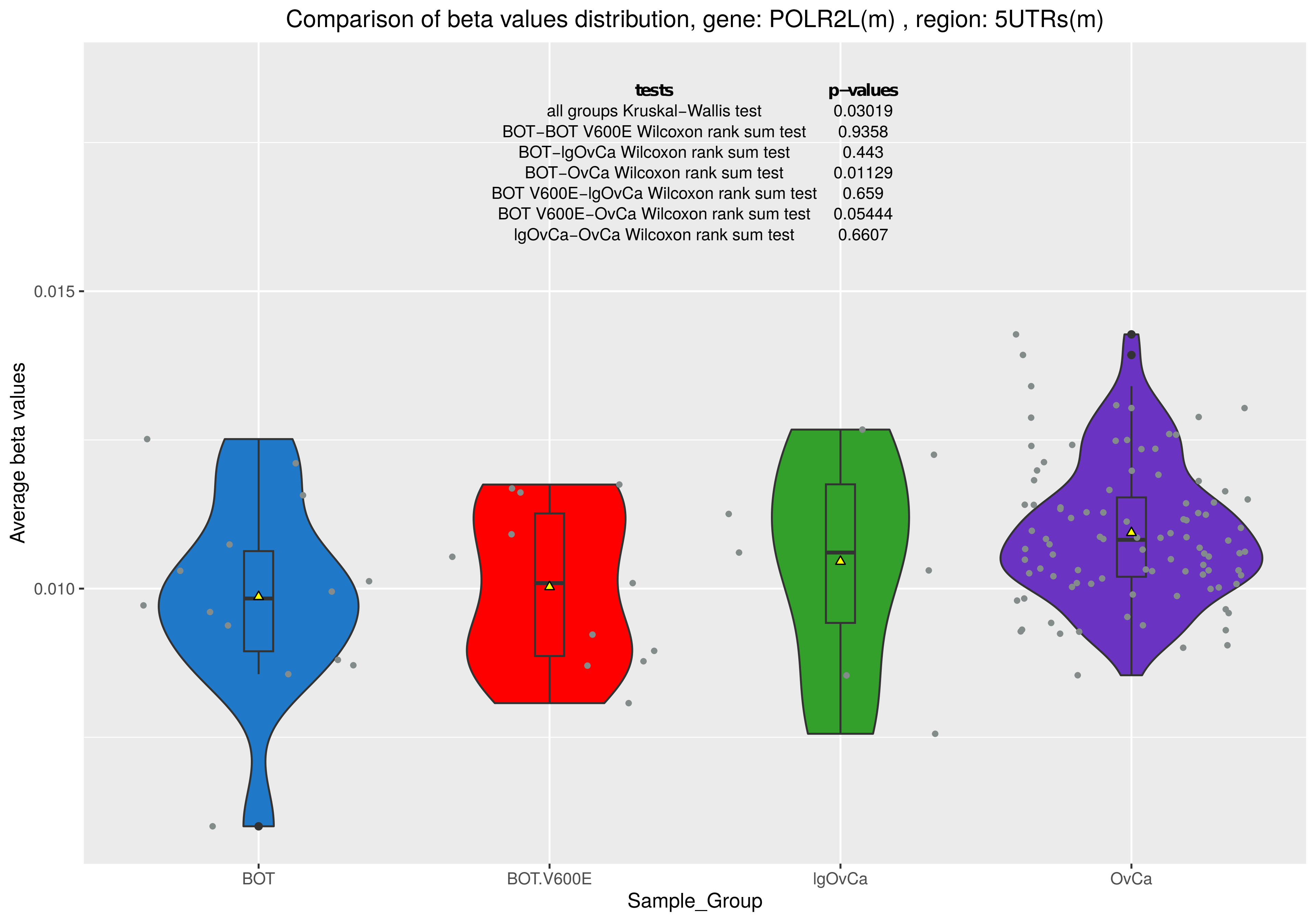

Comparison of beta values distribution, gene: POLR2L(m) , region: 5UTRs(m)
tests
p−values
all groups Kruskal−Wallis test
0.03019
BOT−BOT V600E Wilcoxon rank sum test
0.9358
BOT−lgOvCa Wilcoxon rank sum test
0.443
BOT−OvCa Wilcoxon rank sum test
0.01129
BOT V600E−lgOvCa Wilcoxon rank sum test
0.659
BOT V600E−OvCa Wilcoxon rank sum test
0.05444
lgOvCa−OvCa Wilcoxon rank sum test
0.6607
Average beta values
0.015
0.010
BOT
BOT.V600E
lgOvCa
OvCa
Sample_Group

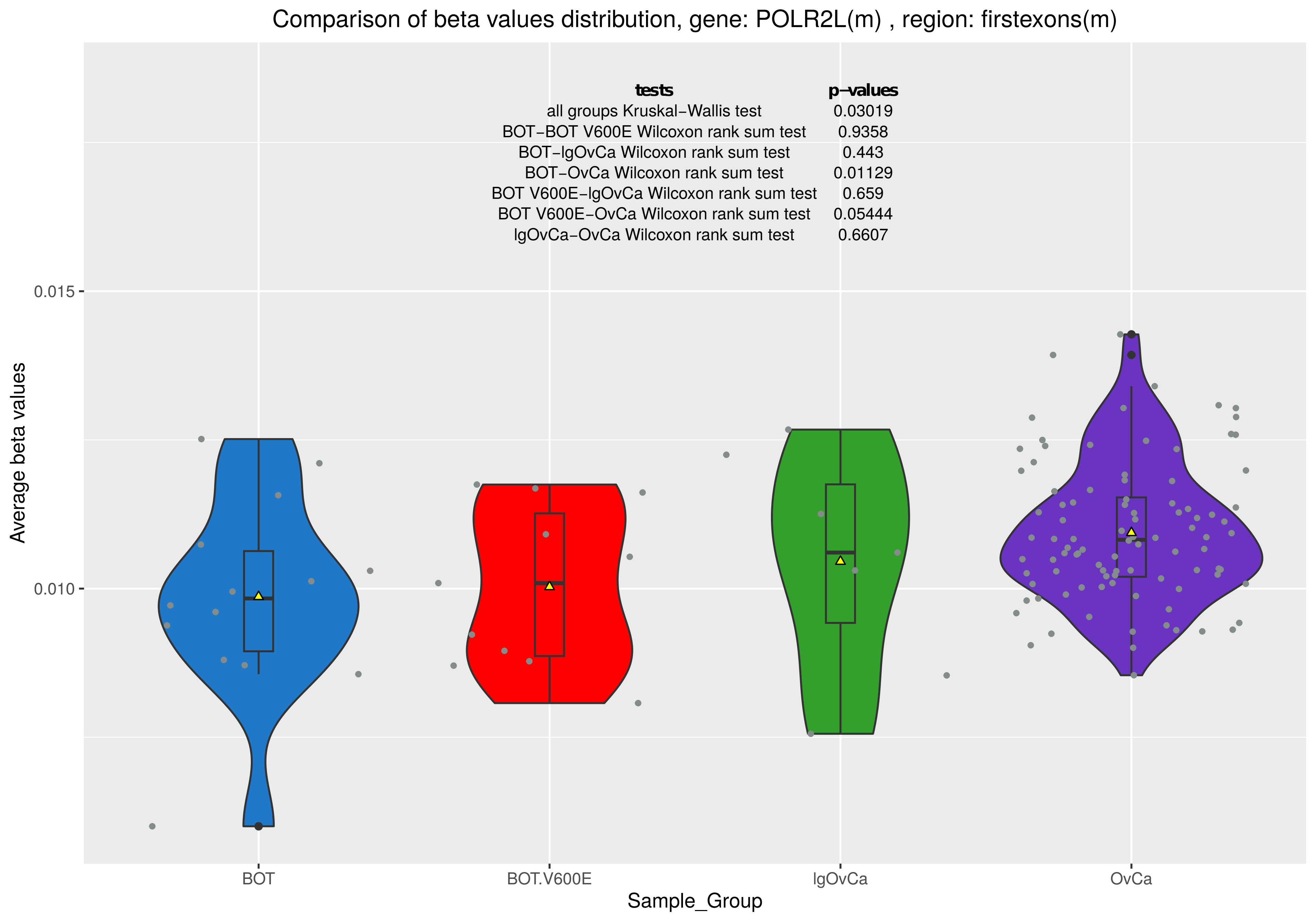
Comparison of beta values distribution, gene: POLR2L(m) , region: firstexons(m)
tests
p−values
all groups Kruskal−Wallis test
0.03019
BOT−BOT V600E Wilcoxon rank sum test
0.9358
BOT−IgOvCa Wilcoxon rank sum test
0.443
BOT−OvCa Wilcoxon rank sum test
0.01129
BOT V600E−IgOvCa Wilcoxon rank sum test
0.659
BOT V600E−OvCa Wilcoxon rank sum test
0.05444
IgOvCa−OvCa Wilcoxon rank sum test
0.6607
0.015
0.010
Average beta values
BOT
BOT.V600E
IgOvCa
OvCa
Sample_Group

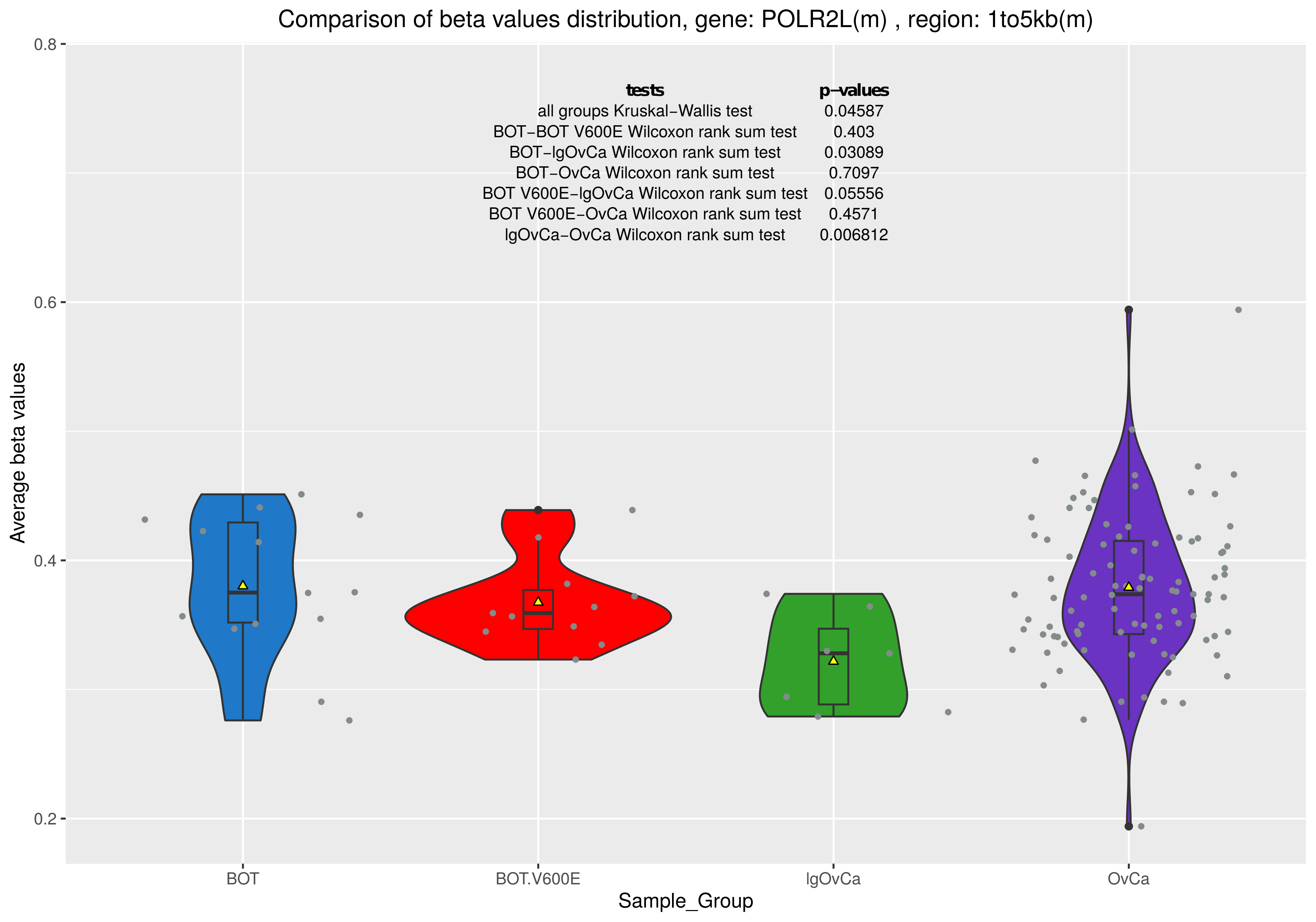

Comparison of beta values distribution, gene: POLR2L(m) , region: 1to5kb(m)
tests
p−values
all groups Kruskal−Wallis test
0.04587
BOT−BOT V600E Wilcoxon rank sum test
0.403
BOT−lgOvCa Wilcoxon rank sum test
0.03089
BOT−OvCa Wilcoxon rank sum test
0.7097
BOT V600E−lgOvCa Wilcoxon rank sum test
0.05556
BOT V600E−OvCa Wilcoxon rank sum test
0.4571
lgOvCa−OvCa Wilcoxon rank sum test
0.006812
0.8
0.6
0.4
0.2
Average beta values
BOT
BOT.V600E
lgOvCa
OvCa
Sample_Group

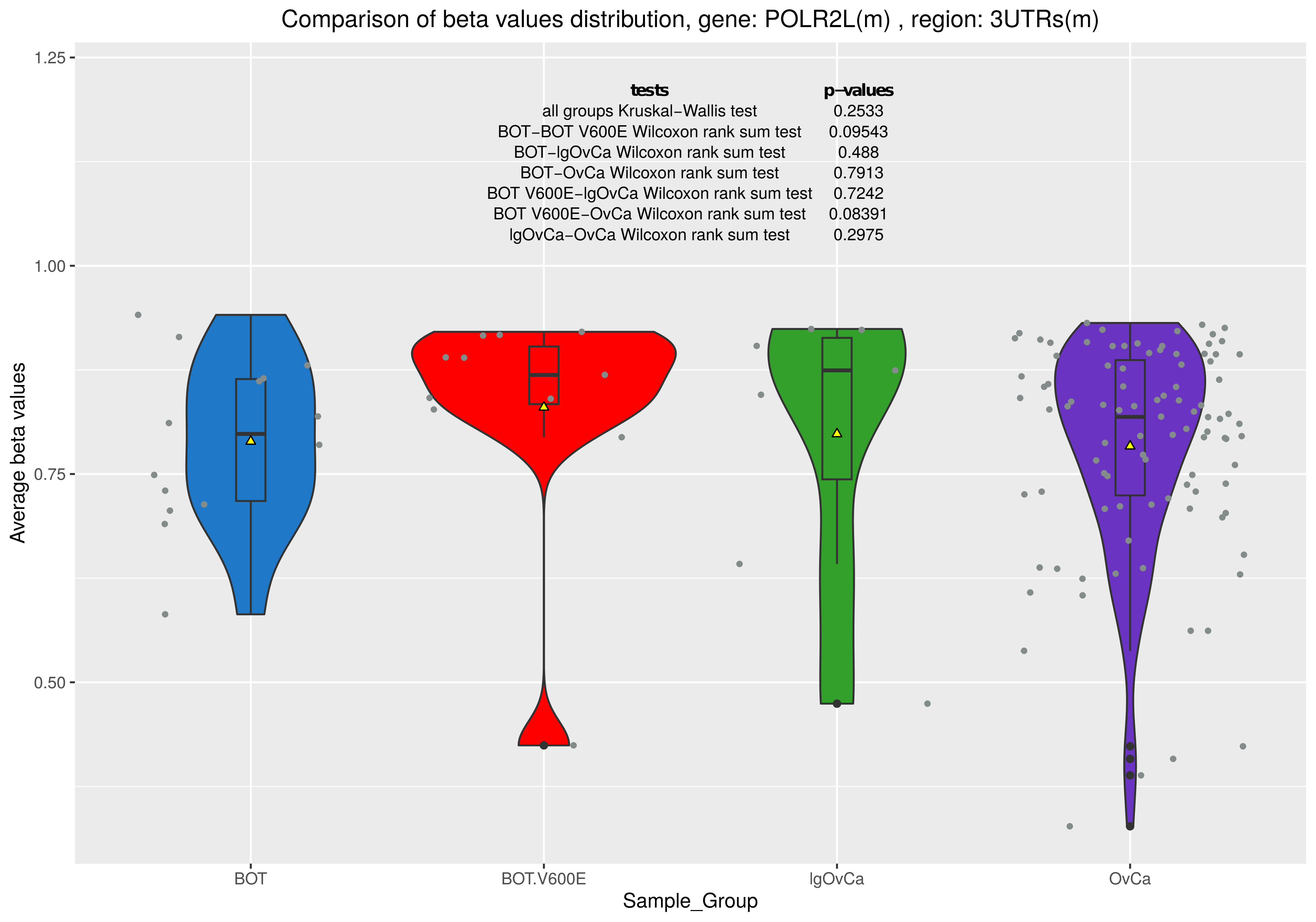

Comparison of beta values distribution, gene: POLR2L(m) , region: 3UTRs(m)
tests
p−values
all groups Kruskal−Wallis test
0.2533
BOT−BOT V600E Wilcoxon rank sum test
0.09543
BOT−lgOvCa Wilcoxon rank sum test
0.488
BOT−OvCa Wilcoxon rank sum test
0.7913
BOT V600E−lgOvCa Wilcoxon rank sum test
0.7242
BOT V600E−OvCa Wilcoxon rank sum test
0.08391
lgOvCa−OvCa Wilcoxon rank sum test
0.2975
Average beta values
1.25
1.00
0.75
0.50
BOT
BOT.V600E
lgOvCa
OvCa
Sample_Group

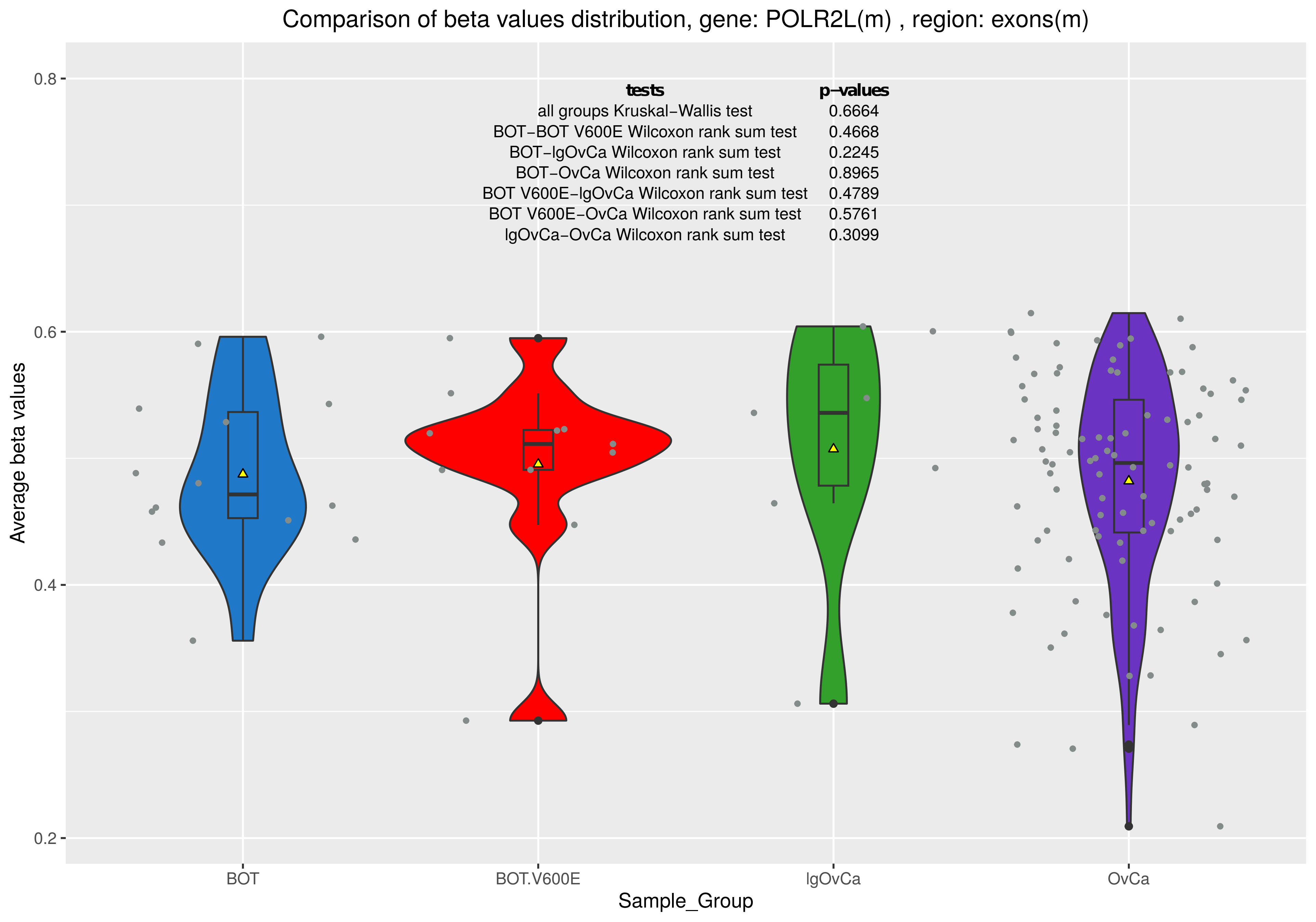
Comparison of beta values distribution, gene: POLR2L(m) , region: exons(m)
tests
p−values
all groups Kruskal−Wallis test 0.6664
BOT−BOT V600E Wilcoxon rank sum test 0.4668
BOT−lgOvCa Wilcoxon rank sum test 0.2245
BOT−OvCa Wilcoxon rank sum test 0.8965
BOT V600E−lgOvCa Wilcoxon rank sum test 0.4789
BOT V600E−OvCa Wilcoxon rank sum test 0.5761
lgOvCa−OvCa Wilcoxon rank sum test 0.3099
Average beta values
0.8
0.6
0.4
0.2
BOT
BOT.V600E
lgOvCa
OvCa
Sample_Group

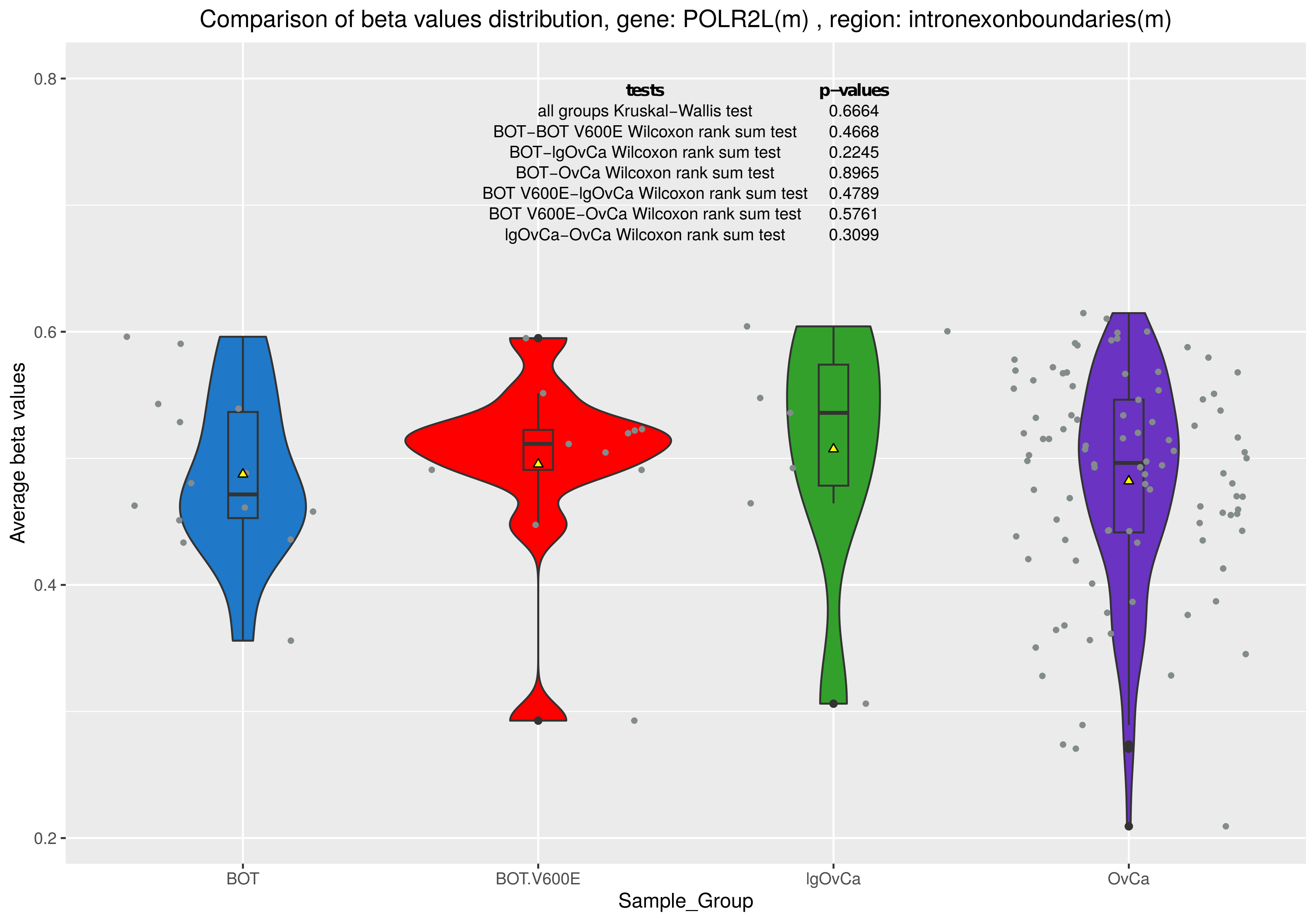
Comparison of beta values distribution, gene: POLR2L(m) , region: intronexonboundaries(m)
tests
p−values
all groups Kruskal−Wallis test
0.6664
BOT−BOT V600E Wilcoxon rank sum test
0.4668
BOT−IgOvCa Wilcoxon rank sum test
0.2245
BOT−OvCa Wilcoxon rank sum test
0.8965
BOT V600E−IgOvCa Wilcoxon rank sum test
0.4789
BOT V600E−OvCa Wilcoxon rank sum test
0.5761
IgOvCa−OvCa Wilcoxon rank sum test
0.3099
Average beta values
0.8
0.6
0.4
0.2
BOT
BOT.V600E
IgOvCa
OvCa
Sample_Group

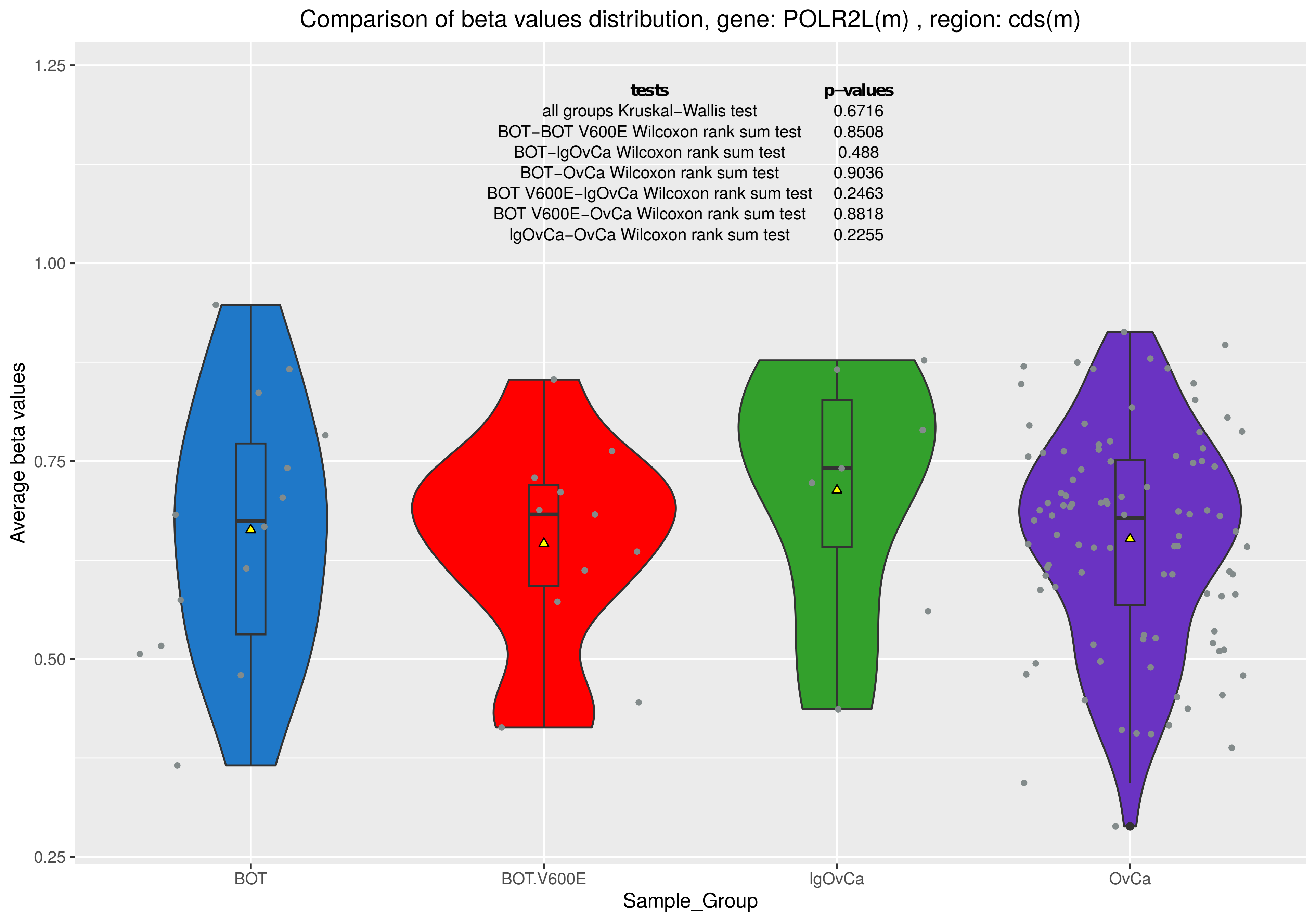
Comparison of beta values distribution, gene: POLR2L(m) , region: cds(m)
tests
p−values
all groups Kruskal−Wallis test
0.6716
BOT−BOT V600E Wilcoxon rank sum test
0.8508
BOT−lgOvCa Wilcoxon rank sum test
0.488
BOT−OvCa Wilcoxon rank sum test
0.9036
BOT V600E−lgOvCa Wilcoxon rank sum test
0.2463
BOT V600E−OvCa Wilcoxon rank sum test
0.8818
lgOvCa−OvCa Wilcoxon rank sum test
0.2255
Average beta values
1.25
1.00
0.75
0.50
0.25
BOT
BOT.V600E
lgOvCa
OvCa
Sample_Group

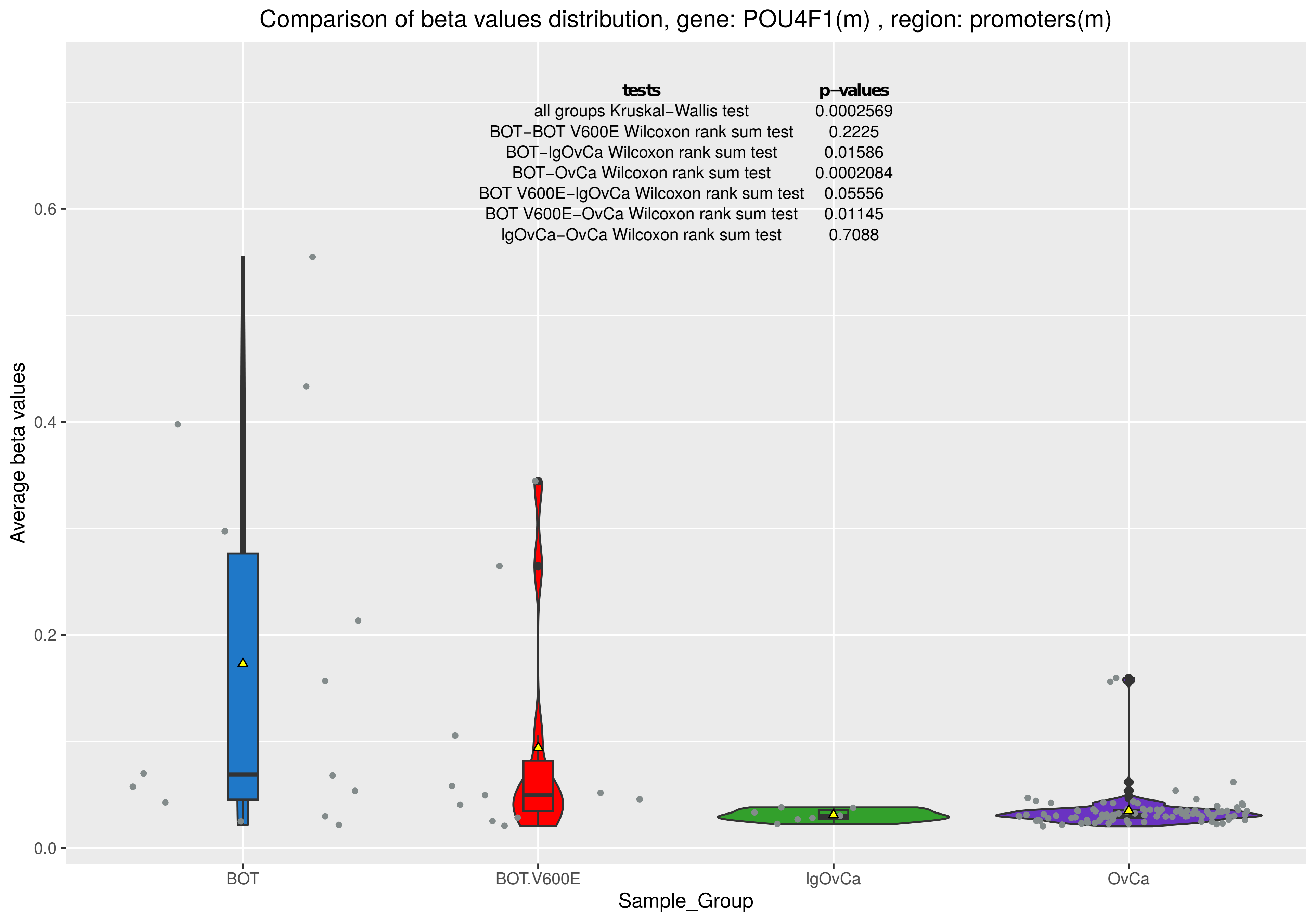

Comparison of beta values distribution, gene: POU4F1(m) , region: promoters(m)
tests
p−values
all groups Kruskal−Wallis test
0.0002569
BOT−BOT V600E Wilcoxon rank sum test
0.2225
BOT−lgOvCa Wilcoxon rank sum test
0.01586
BOT−OvCa Wilcoxon rank sum test
0.0002084
BOT V600E−lgOvCa Wilcoxon rank sum test
0.05556
BOT V600E−OvCa Wilcoxon rank sum test
0.01145
lgOvCa−OvCa Wilcoxon rank sum test
0.7088
Average beta values
0.6
0.4
0.2
0.0
BOT
BOT.V600E
lgOvCa
OvCa
Sample_Group

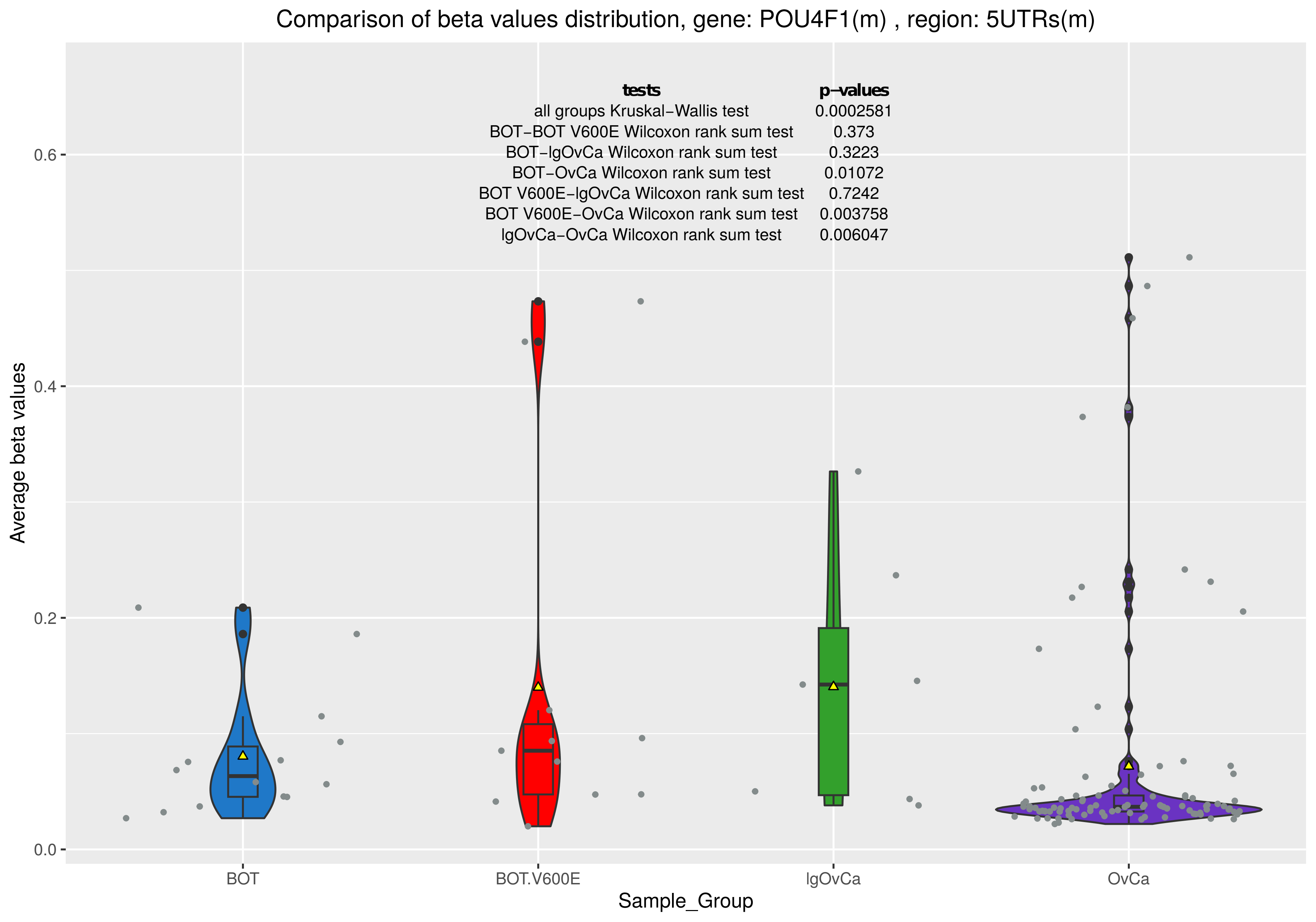
Comparison of beta values distribution, gene: POU4F1(m) , region: 5UTRs(m)
tests
p−values
all groups Kruskal−Wallis test
0.0002581
BOT−BOT V600E Wilcoxon rank sum test
0.373
BOT−lgOvCa Wilcoxon rank sum test
0.3223
BOT−OvCa Wilcoxon rank sum test
0.01072
BOT V600E−lgOvCa Wilcoxon rank sum test
0.7242
BOT V600E−OvCa Wilcoxon rank sum test
0.003758
lgOvCa−OvCa Wilcoxon rank sum test
0.006047
Average beta values
0.0
0.2
0.4
0.6
BOT
BOT.V600E
lgOvCa
OvCa
Sample_Group

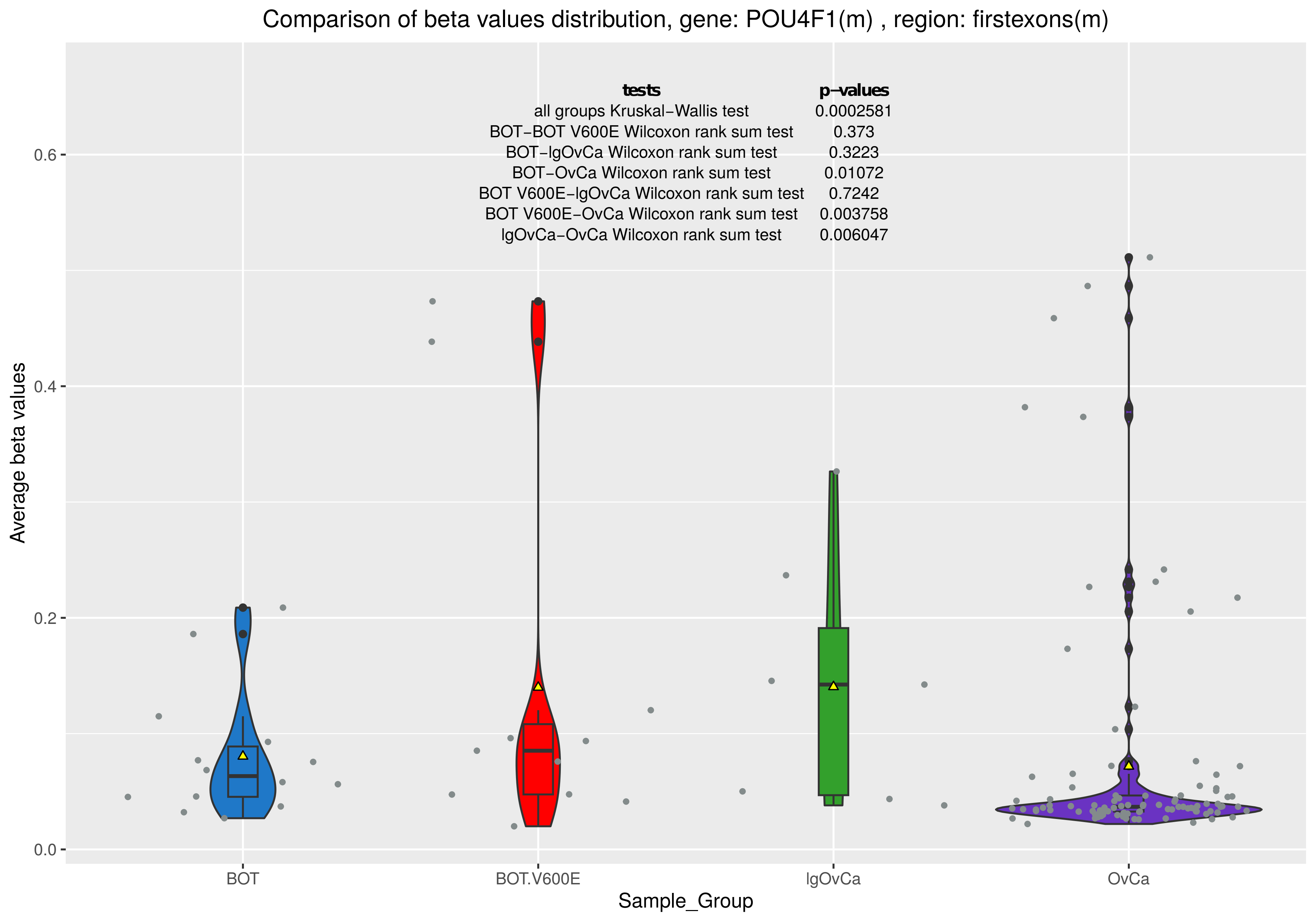
Comparison of beta values distribution, gene: POU4F1(m) , region: firstexons(m)
tests
p−values
all groups Kruskal−Wallis test
0.0002581
BOT−BOT V600E Wilcoxon rank sum test
0.373
BOT−lgOvCa Wilcoxon rank sum test
0.3223
BOT−OvCa Wilcoxon rank sum test
0.01072
BOT V600E−lgOvCa Wilcoxon rank sum test
0.7242
BOT V600E−OvCa Wilcoxon rank sum test
0.003758
lgOvCa−OvCa Wilcoxon rank sum test
0.006047
Average beta values
0.6
0.4
0.2
0.0
BOT
BOT.V600E
lgOvCa
OvCa
Sample_Group

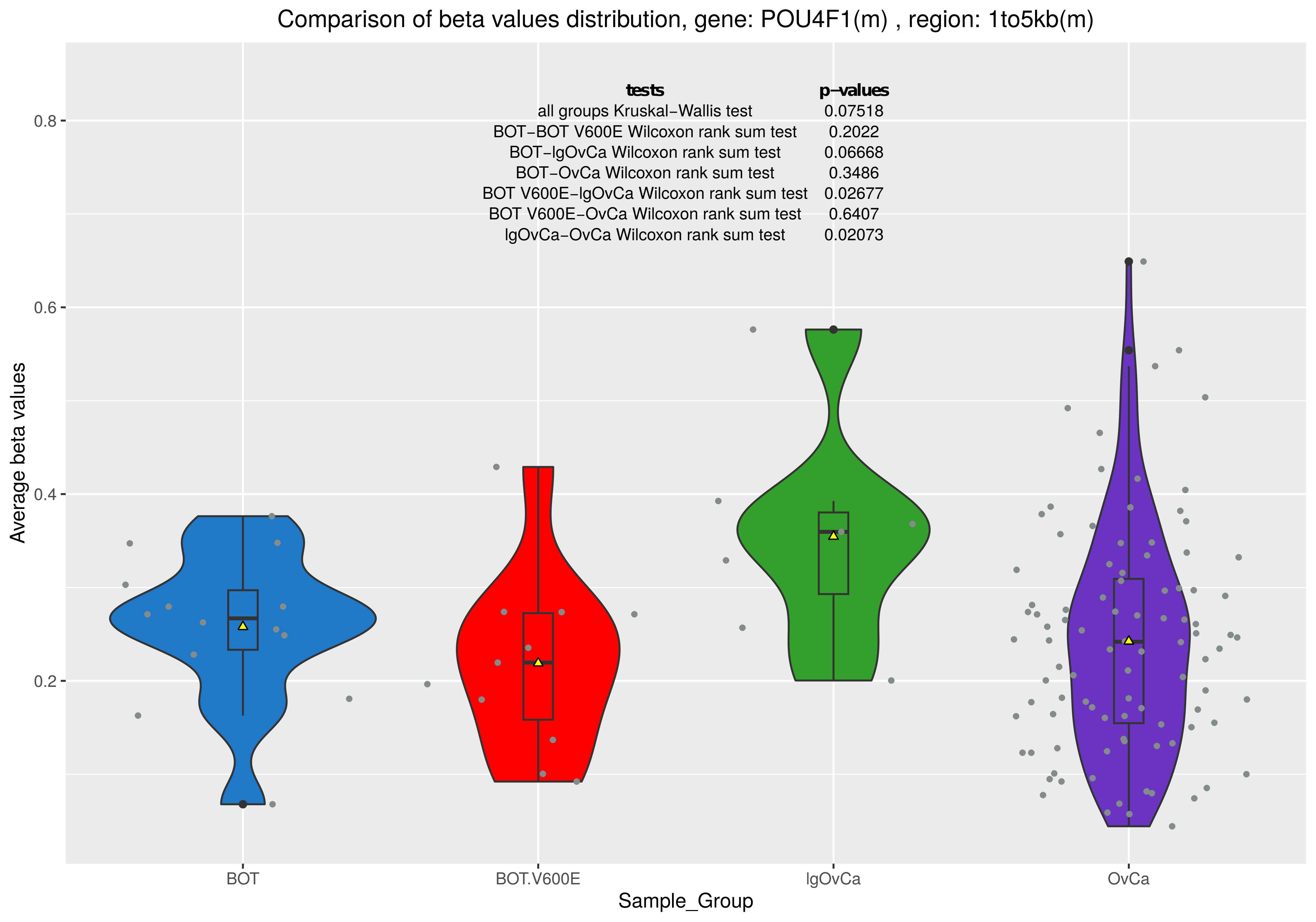

Comparison of beta values distribution, gene: POU4F1(m) , region: 1to5kb(m)
tests
p−values
all groups Kruskal−Wallis test
0.07518
BOT−BOT V600E Wilcoxon rank sum test
0.2022
BOT−lgOvCa Wilcoxon rank sum test
0.06668
BOT−OvCa Wilcoxon rank sum test
0.3486
BOT V600E−lgOvCa Wilcoxon rank sum test
0.02677
BOT V600E−OvCa Wilcoxon rank sum test
0.6407
lgOvCa−OvCa Wilcoxon rank sum test
0.02073
Average beta values
0.8
0.6
0.4
0.2
BOT
BOT.V600E
lgOvCa
OvCa
Sample_Group

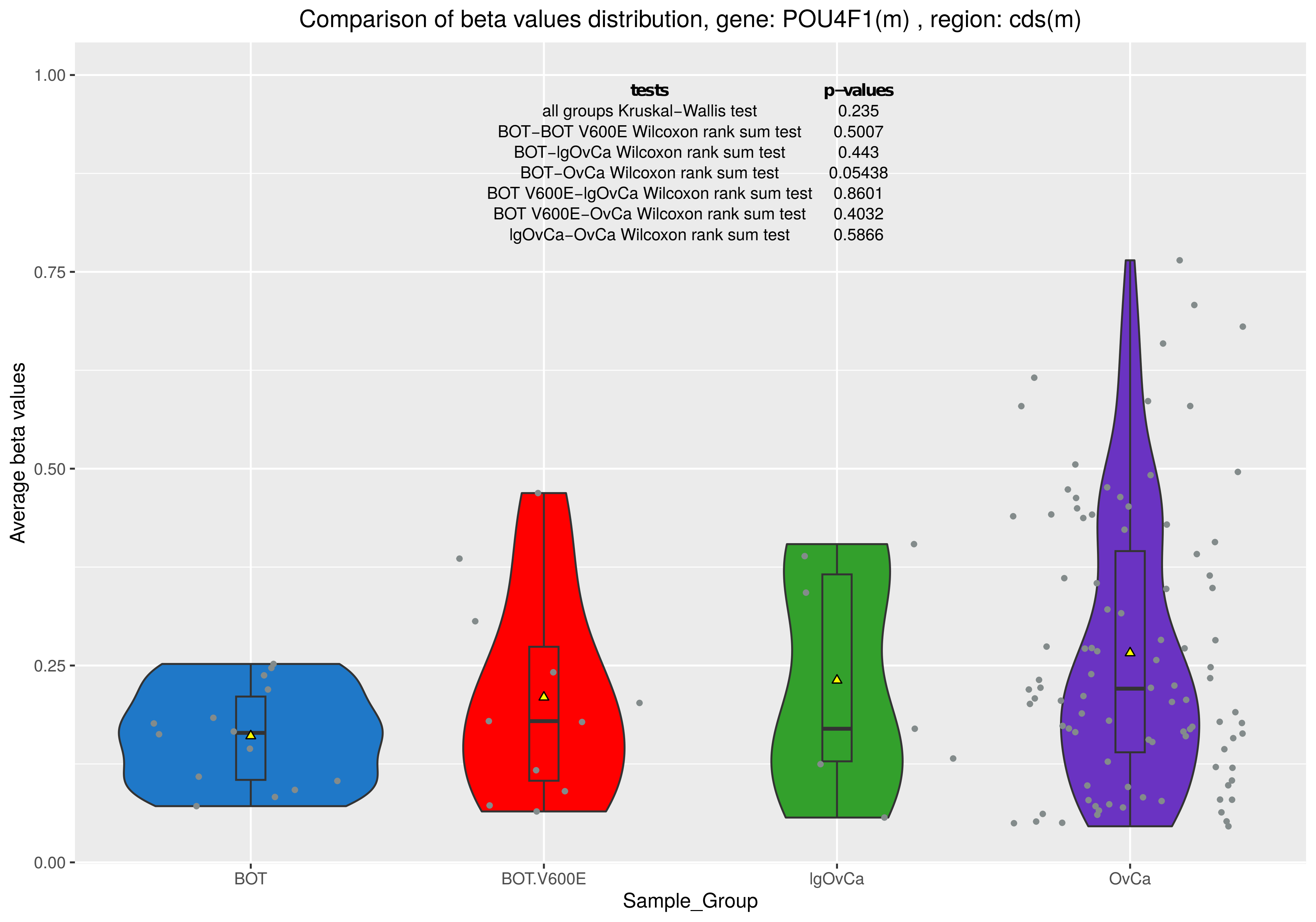
Comparison of beta values distribution, gene: POU4F1(m) , region: cds(m)
tests
p−values
all groups Kruskal−Wallis test 0.235
BOT−BOT V600E Wilcoxon rank sum test 0.5007
BOT−lgOvCa Wilcoxon rank sum test 0.443
BOT−OvCa Wilcoxon rank sum test 0.05438
BOT V600E−lgOvCa Wilcoxon rank sum test 0.8601
BOT V600E−OvCa Wilcoxon rank sum test 0.4032
lgOvCa−OvCa Wilcoxon rank sum test 0.5866
Average beta values
1.00
0.75
0.50
0.25
0.00
BOT
BOT.V600E
lgOvCa
OvCa
Sample_Group

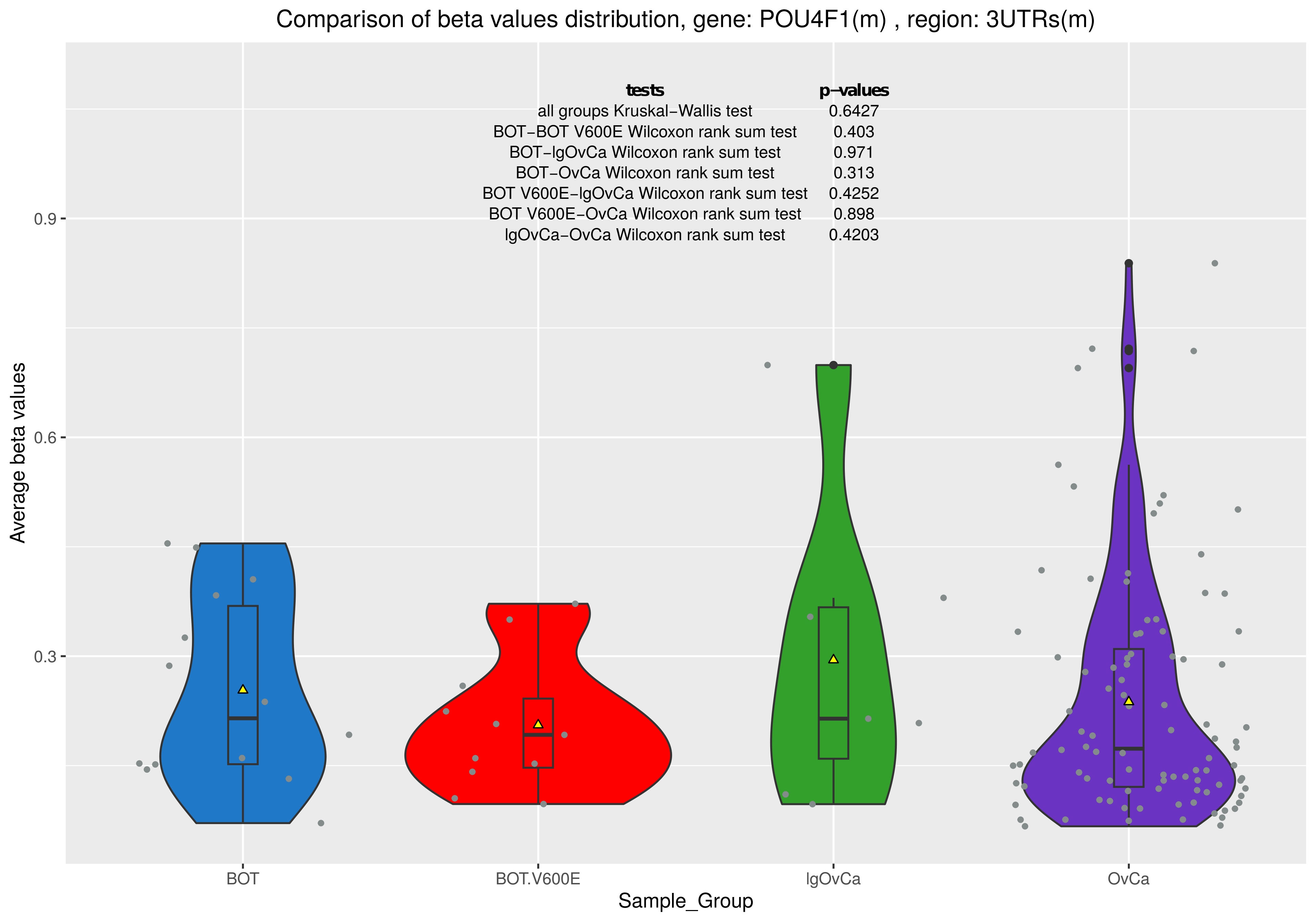

Comparison of beta values distribution, gene: POU4F1(m) , region: 3UTRs(m)
tests
p−values
all groups Kruskal−Wallis test
0.6427
BOT−BOT V600E Wilcoxon rank sum test
0.403
BOT−lgOvCa Wilcoxon rank sum test
0.971
BOT−OvCa Wilcoxon rank sum test
0.313
BOT V600E−lgOvCa Wilcoxon rank sum test
0.4252
BOT V600E−OvCa Wilcoxon rank sum test
0.898
lgOvCa−OvCa Wilcoxon rank sum test
0.4203
Average beta values
0.9
0.6
0.3
BOT
BOT.V600E
lgOvCa
OvCa
Sample_Group

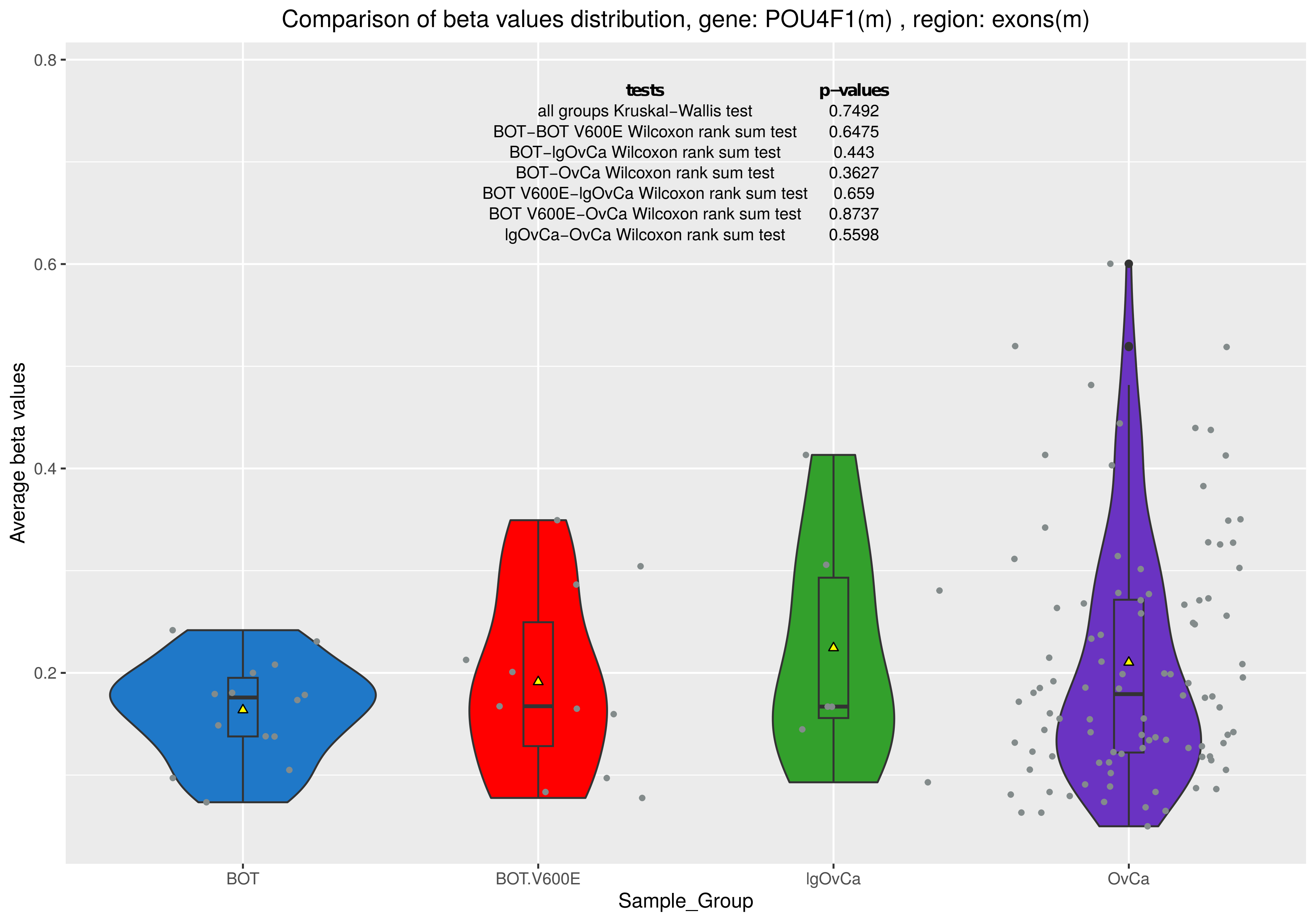

Comparison of beta values distribution, gene: POU4F1(m) , region: exons(m)
tests
p−values
all groups Kruskal−Wallis test
0.7492
BOT−BOT V600E Wilcoxon rank sum test
0.6475
BOT−lgOvCa Wilcoxon rank sum test
0.443
BOT−OvCa Wilcoxon rank sum test
0.3627
BOT V600E−lgOvCa Wilcoxon rank sum test
0.659
BOT V600E−OvCa Wilcoxon rank sum test
0.8737
lgOvCa−OvCa Wilcoxon rank sum test
0.5598
Average beta values
0.8
0.6
0.4
0.2
BOT
BOT.V600E
lgOvCa
OvCa
Sample_Group

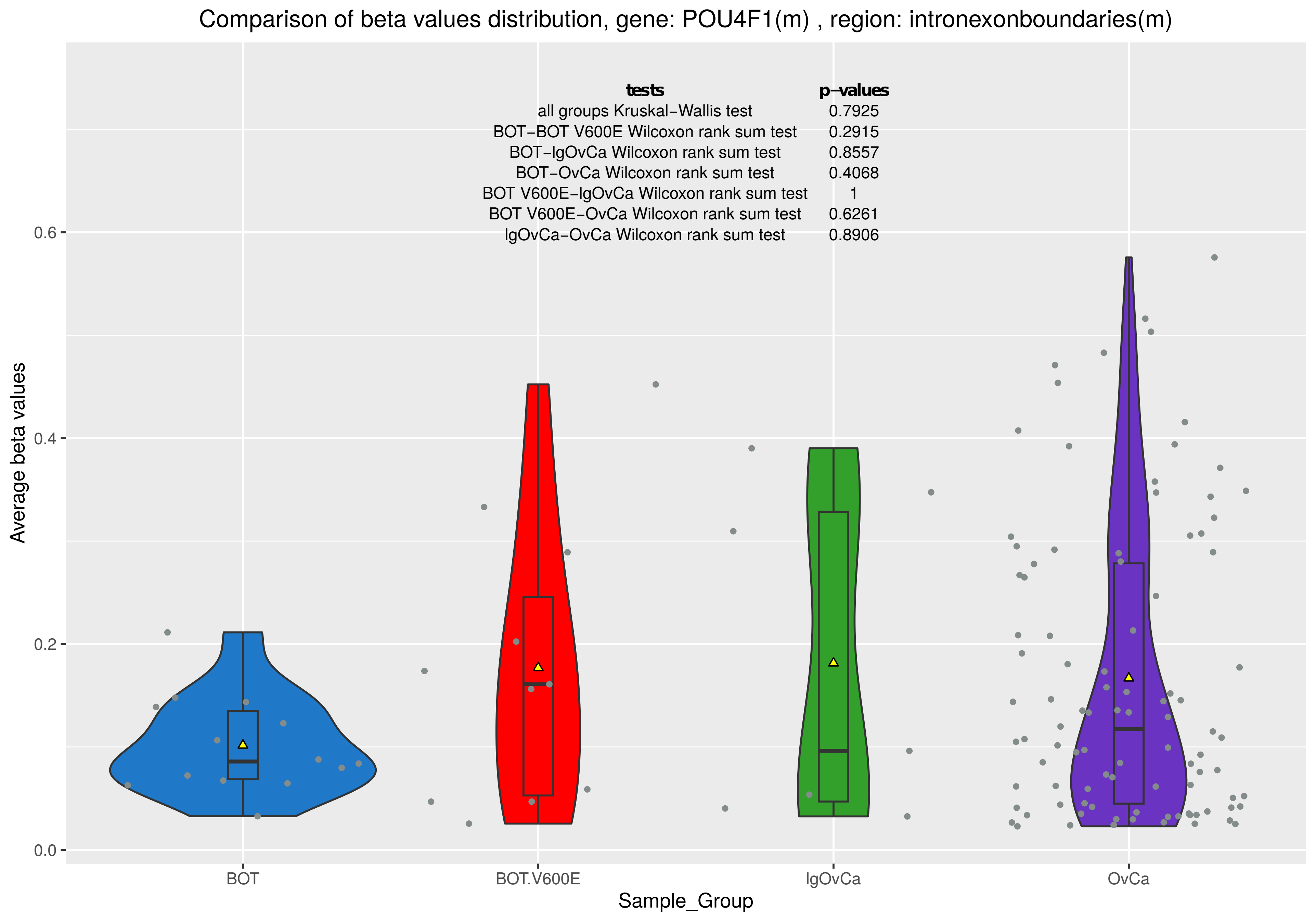
Comparison of beta values distribution, gene: POU4F1(m) , region: intronexonboundaries(m)
tests
p−values
all groups Kruskal−Wallis test
0.7925
BOT−BOT V600E Wilcoxon rank sum test
0.2915
BOT−lgOvCa Wilcoxon rank sum test
0.8557
BOT−OvCa Wilcoxon rank sum test
0.4068
BOT V600E−lgOvCa Wilcoxon rank sum test
1
BOT V600E−OvCa Wilcoxon rank sum test
0.6261
lgOvCa−OvCa Wilcoxon rank sum test
0.8906
Average beta values
0.0
0.2
0.4
0.6
BOT
BOT.V600E
lgOvCa
OvCa
Sample_Group

Comparison of beta values distribution, gene: NTN1(p) , region: introns(p)

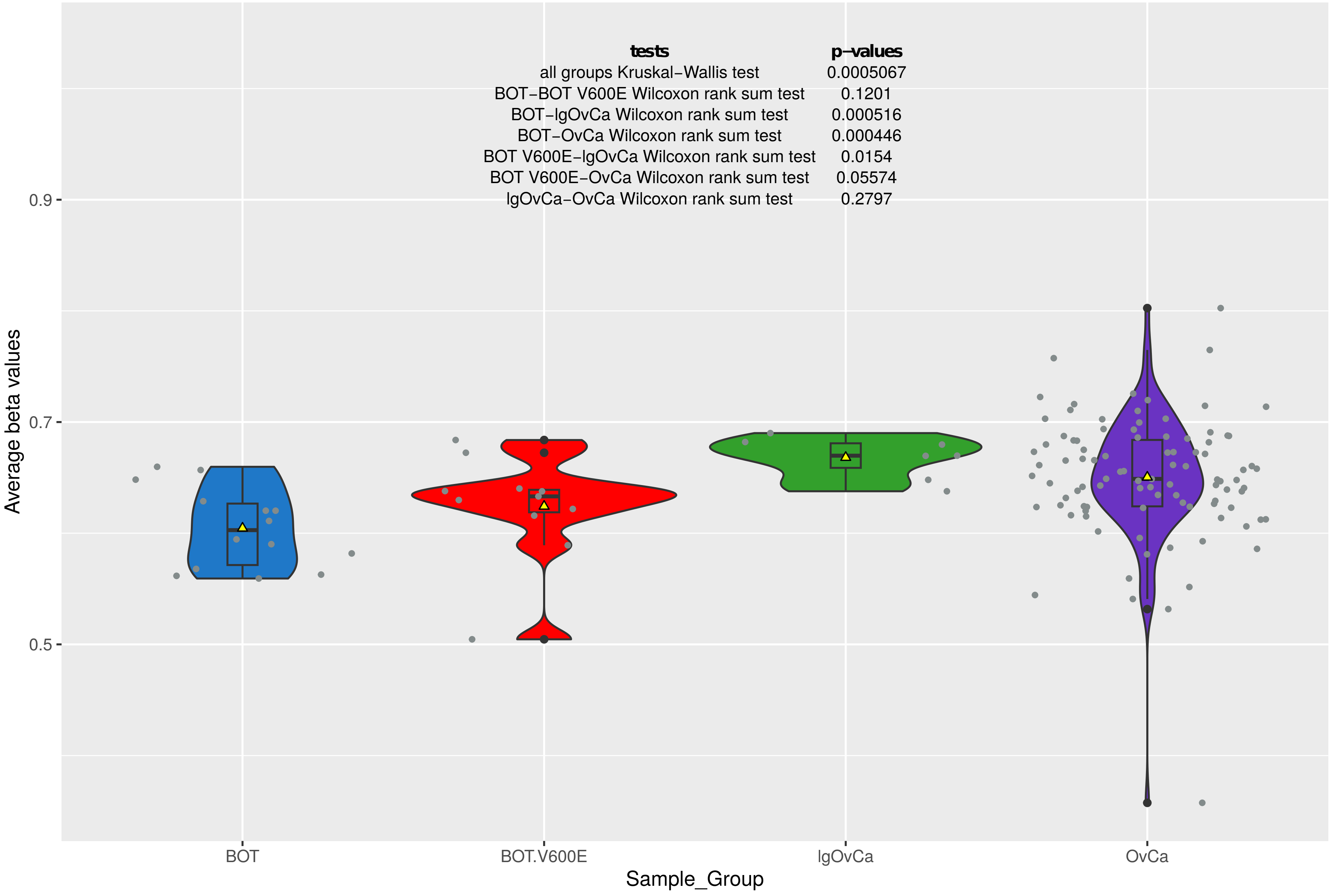

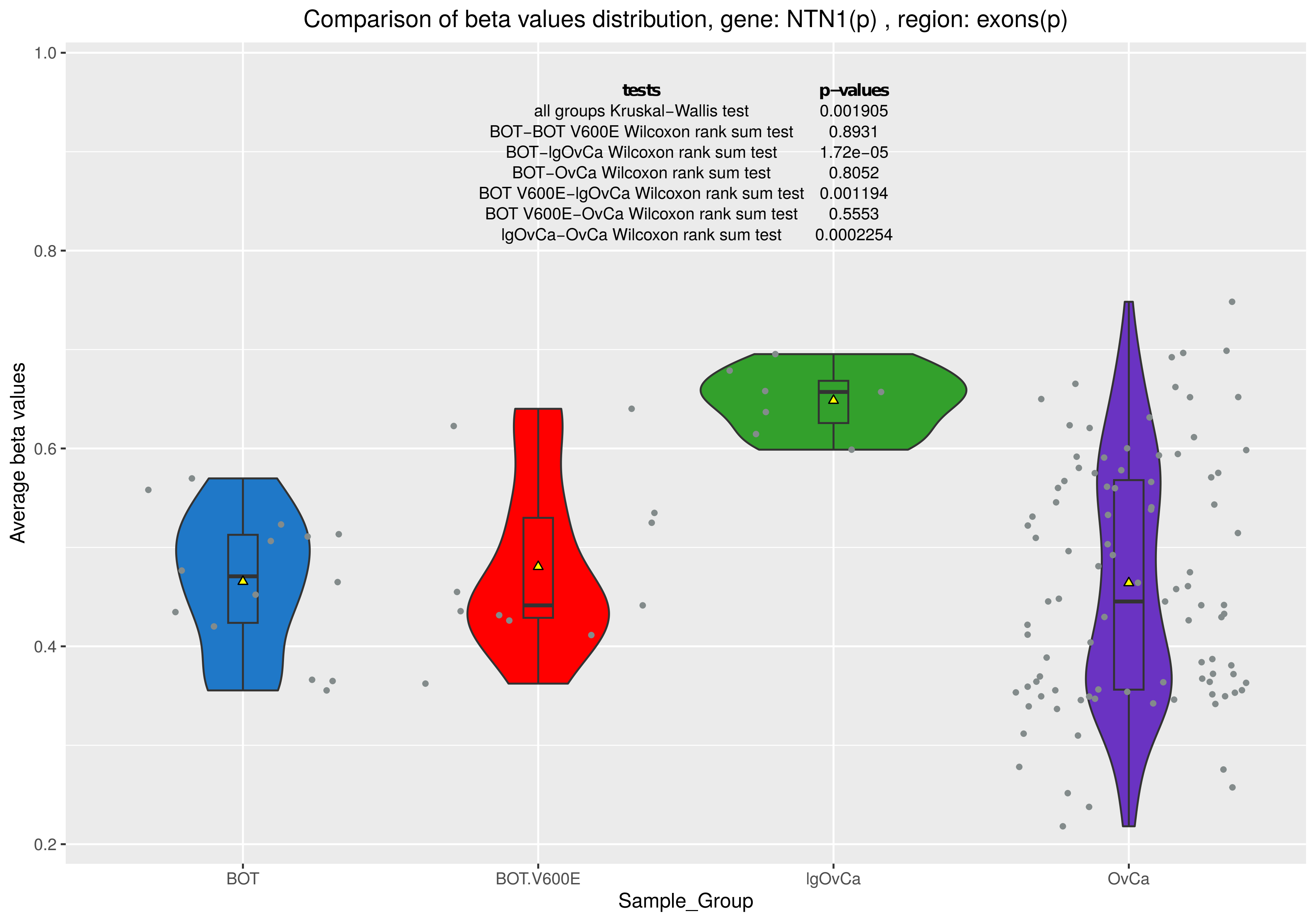

Comparison of beta values distribution, gene: NTN1(p) , region: exons(p)
tests
p−values
all groups Kruskal−Wallis test 0.001905
BOT−BOT V600E Wilcoxon rank sum test 0.8931
BOT−lgOvCa Wilcoxon rank sum test 1.72e−05
BOT−OvCa Wilcoxon rank sum test 0.8052
BOT V600E−lgOvCa Wilcoxon rank sum test 0.001194
BOT V600E−OvCa Wilcoxon rank sum test 0.5553
lgOvCa−OvCa Wilcoxon rank sum test 0.0002254
Average beta values
1.0
0.8
0.6
0.4
0.2
BOT
BOT.V600E
lgOvCa
OvCa
Sample_Group

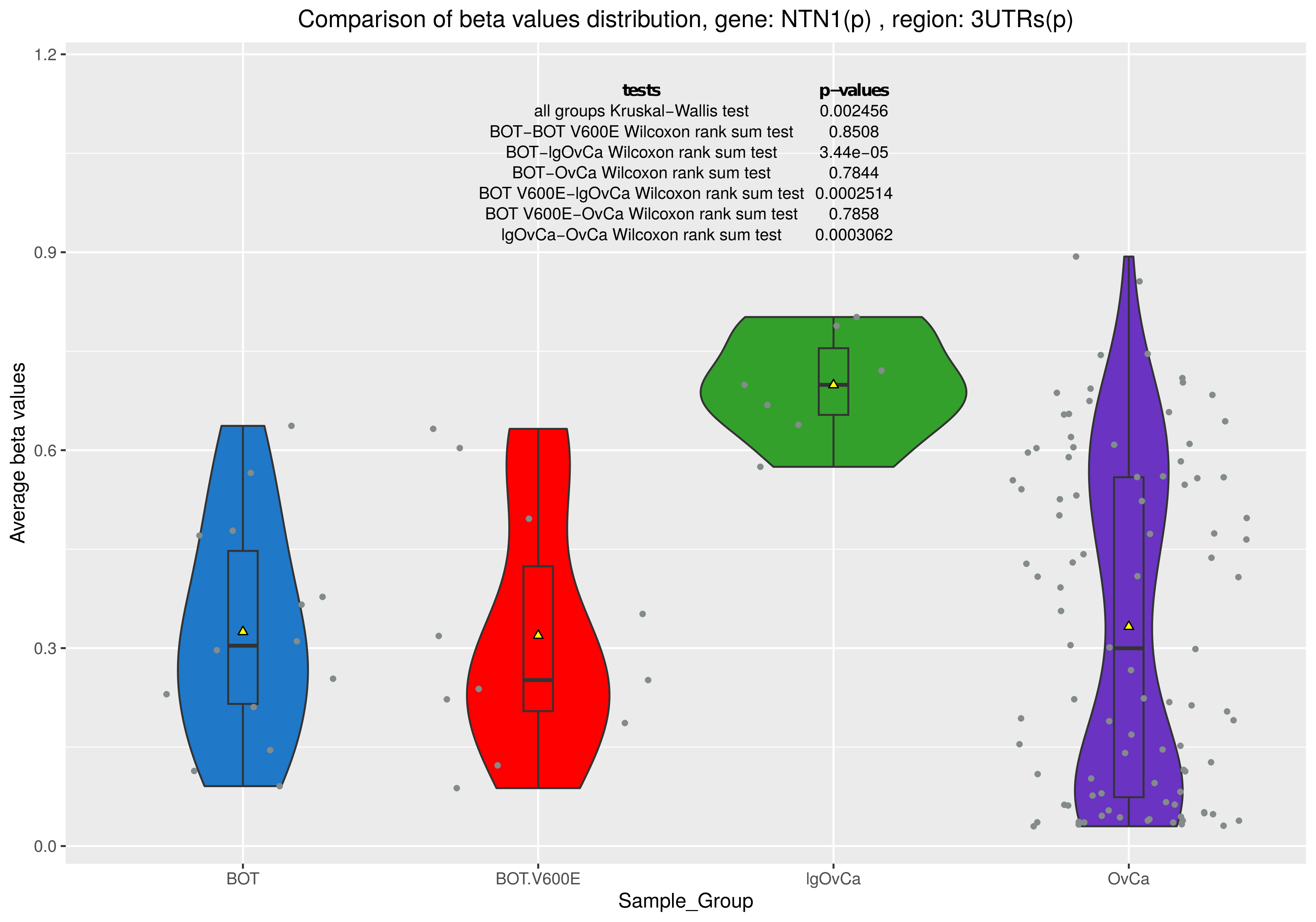

Comparison of beta values distribution, gene: NTN1(p) , region: 3UTRs(p)
tests
p−values
all groups Kruskal−Wallis test
0.002456
BOT−BOT V600E Wilcoxon rank sum test
0.8508
BOT−lgOvCa Wilcoxon rank sum test
3.44e−05
BOT−OvCa Wilcoxon rank sum test
0.7844
BOT V600E−lgOvCa Wilcoxon rank sum test
0.0002514
BOT V600E−OvCa Wilcoxon rank sum test
0.7858
lgOvCa−OvCa Wilcoxon rank sum test
0.0003062
Average beta values
1.2
0.9
0.6
0.3
0.0
BOT
BOT.V600E
lgOvCa
OvCa
Sample_Group

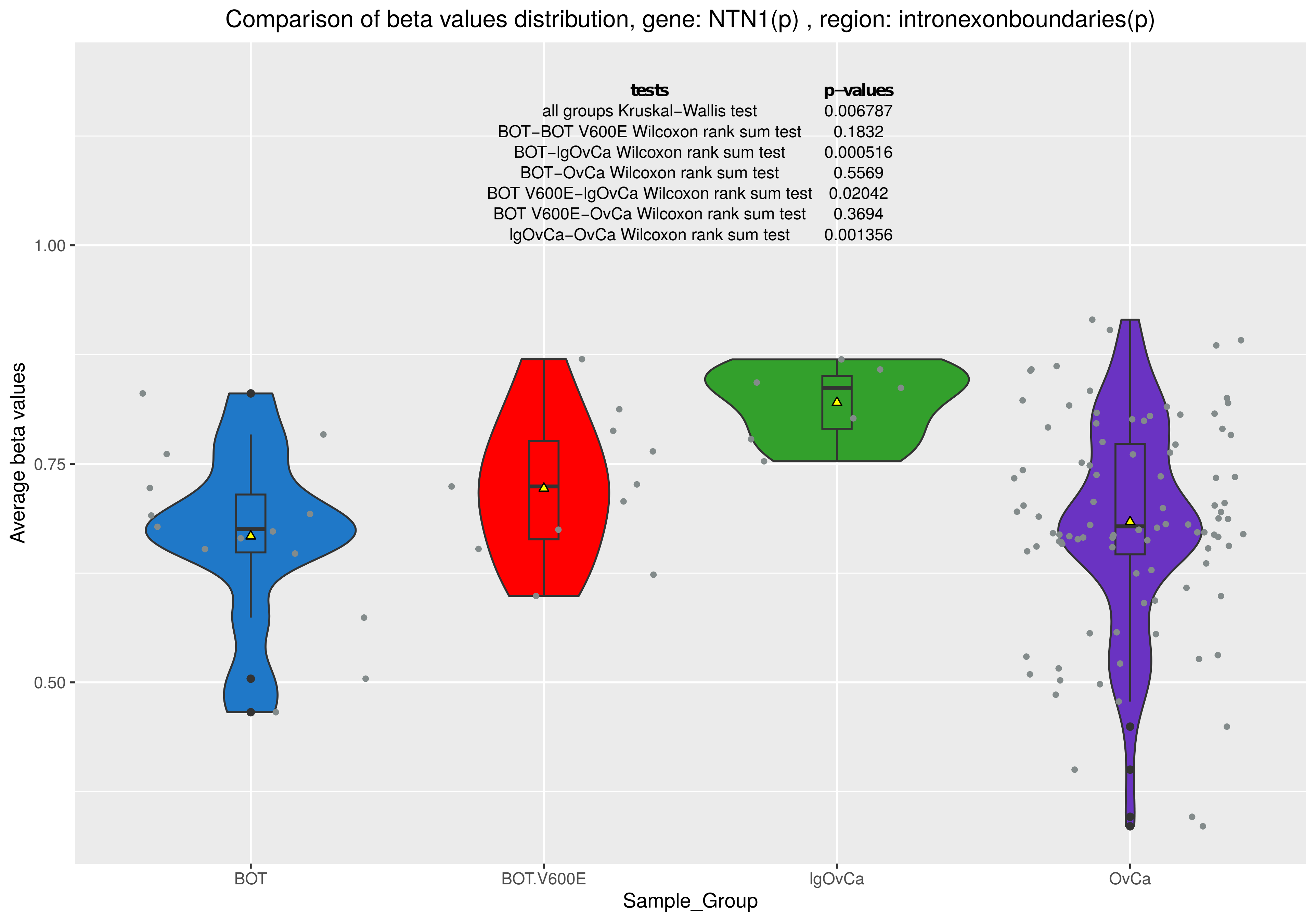

Comparison of beta values distribution, gene: NTN1(p) , region: intronexonboundaries(p)
tests
p−values
all groups Kruskal−Wallis test
0.006787
BOT−BOT V600E Wilcoxon rank sum test
0.1832
BOT−lgOvCa Wilcoxon rank sum test
0.000516
BOT−OvCa Wilcoxon rank sum test
0.5569
BOT V600E−lgOvCa Wilcoxon rank sum test
0.02042
BOT V600E−OvCa Wilcoxon rank sum test
0.3694
lgOvCa−OvCa Wilcoxon rank sum test
0.001356
Average beta values
1.00
0.75
0.50
BOT
BOT.V600E
lgOvCa
OvCa
Sample_Group

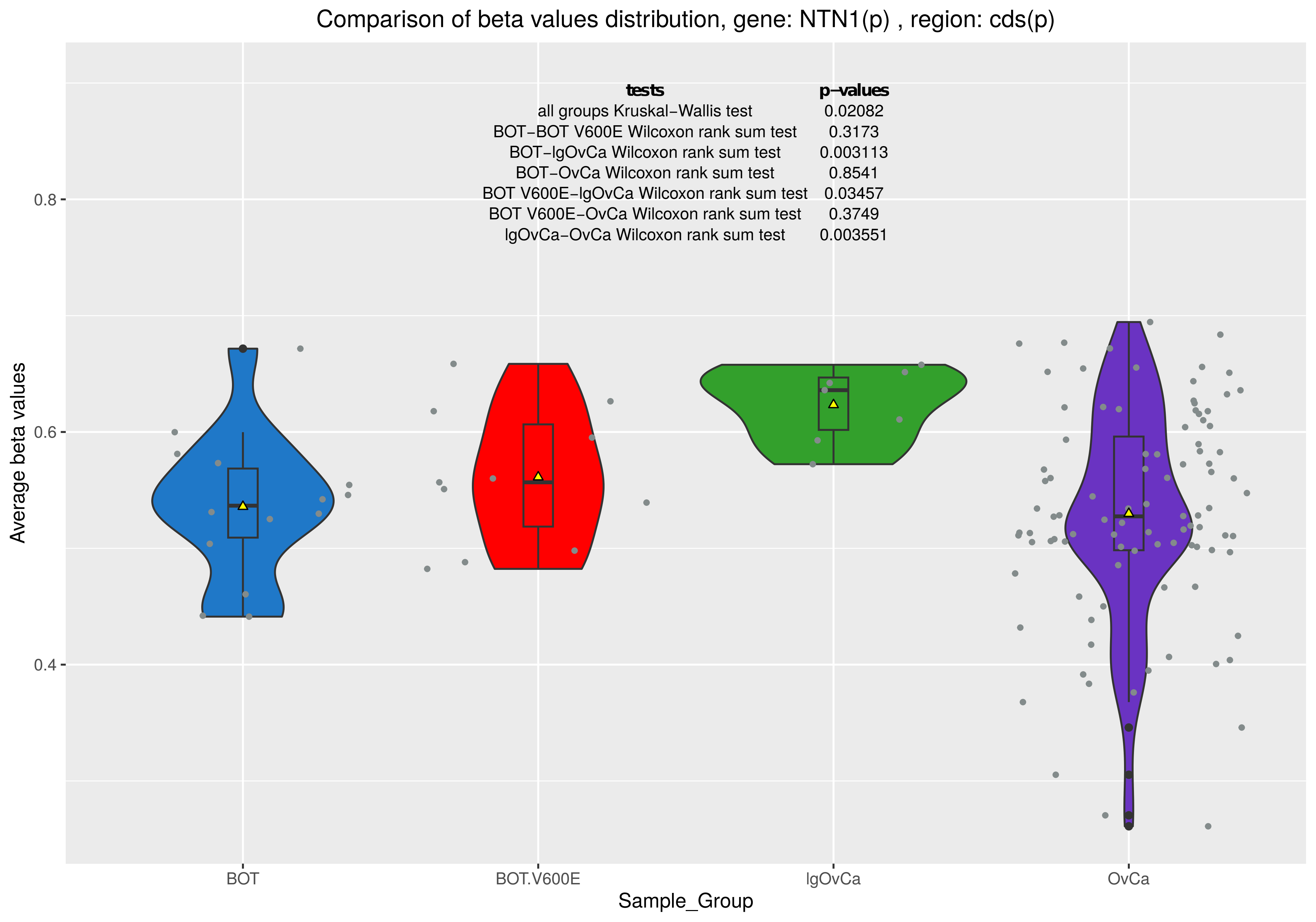
Comparison of beta values distribution, gene: NTN1(p) , region: cds(p)
tests
p−values
all groups Kruskal−Wallis test
0.02082
BOT−BOT V600E Wilcoxon rank sum test
0.3173
BOT−lgOvCa Wilcoxon rank sum test
0.003113
BOT−OvCa Wilcoxon rank sum test
0.8541
BOT V600E−lgOvCa Wilcoxon rank sum test
0.03457
BOT V600E−OvCa Wilcoxon rank sum test
0.3749
lgOvCa−OvCa Wilcoxon rank sum test
0.003551
Average beta values
0.8
0.6
0.4
BOT
BOT.V600E
lgOvCa
OvCa
Sample_Group

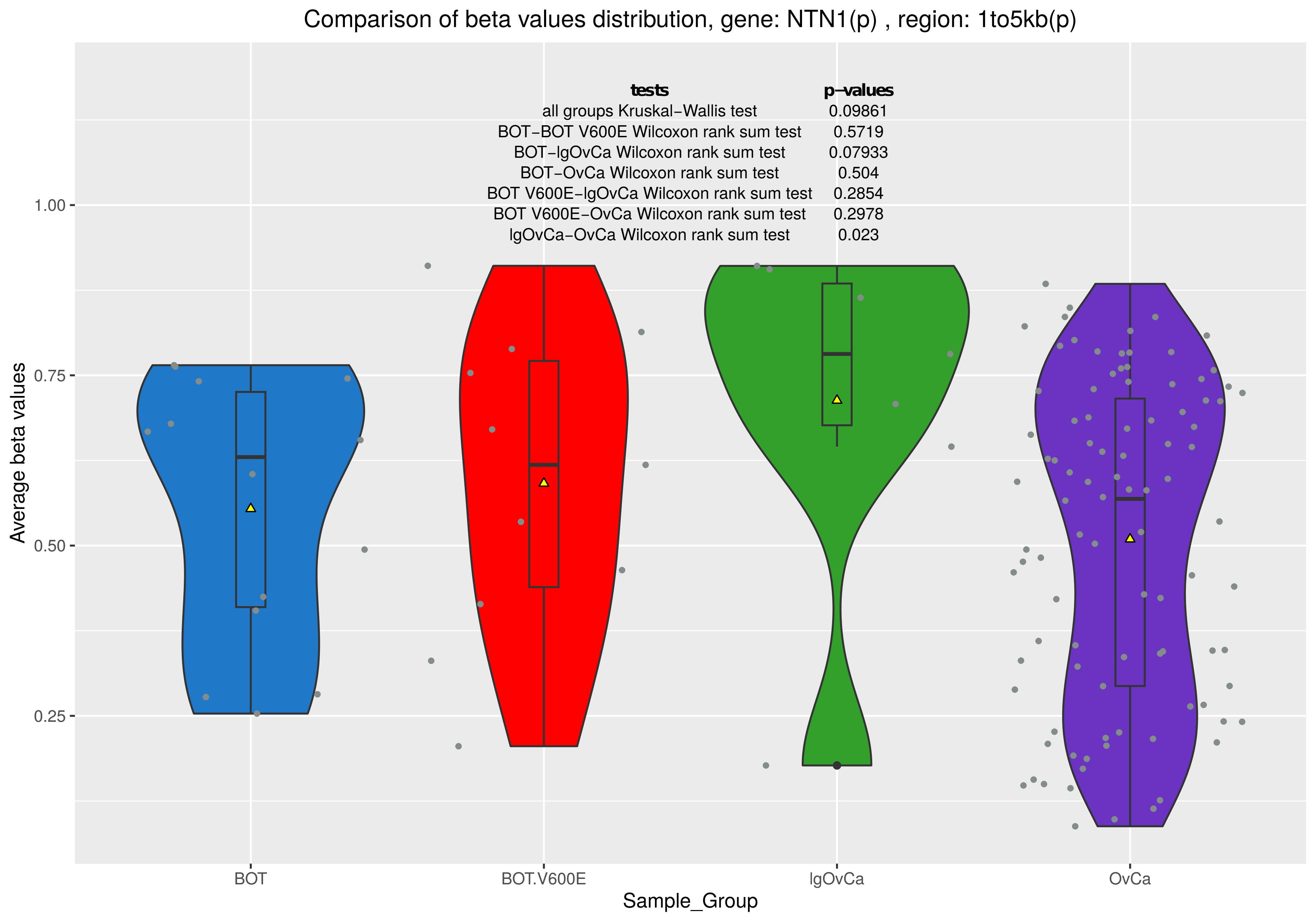

Comparison of beta values distribution, gene: NTN1(p) , region: 1to5kb(p)
tests
p−values
all groups Kruskal−Wallis test
0.09861
BOT−BOT V600E Wilcoxon rank sum test
0.5719
BOT−lgOvCa Wilcoxon rank sum test
0.07933
BOT−OvCa Wilcoxon rank sum test
0.504
BOT V600E−lgOvCa Wilcoxon rank sum test
0.2854
BOT V600E−OvCa Wilcoxon rank sum test
0.2978
lgOvCa−OvCa Wilcoxon rank sum test
0.023
Average beta values
1.00
0.75
0.50
0.25
BOT
BOT.V600E
lgOvCa
OvCa
Sample_Group

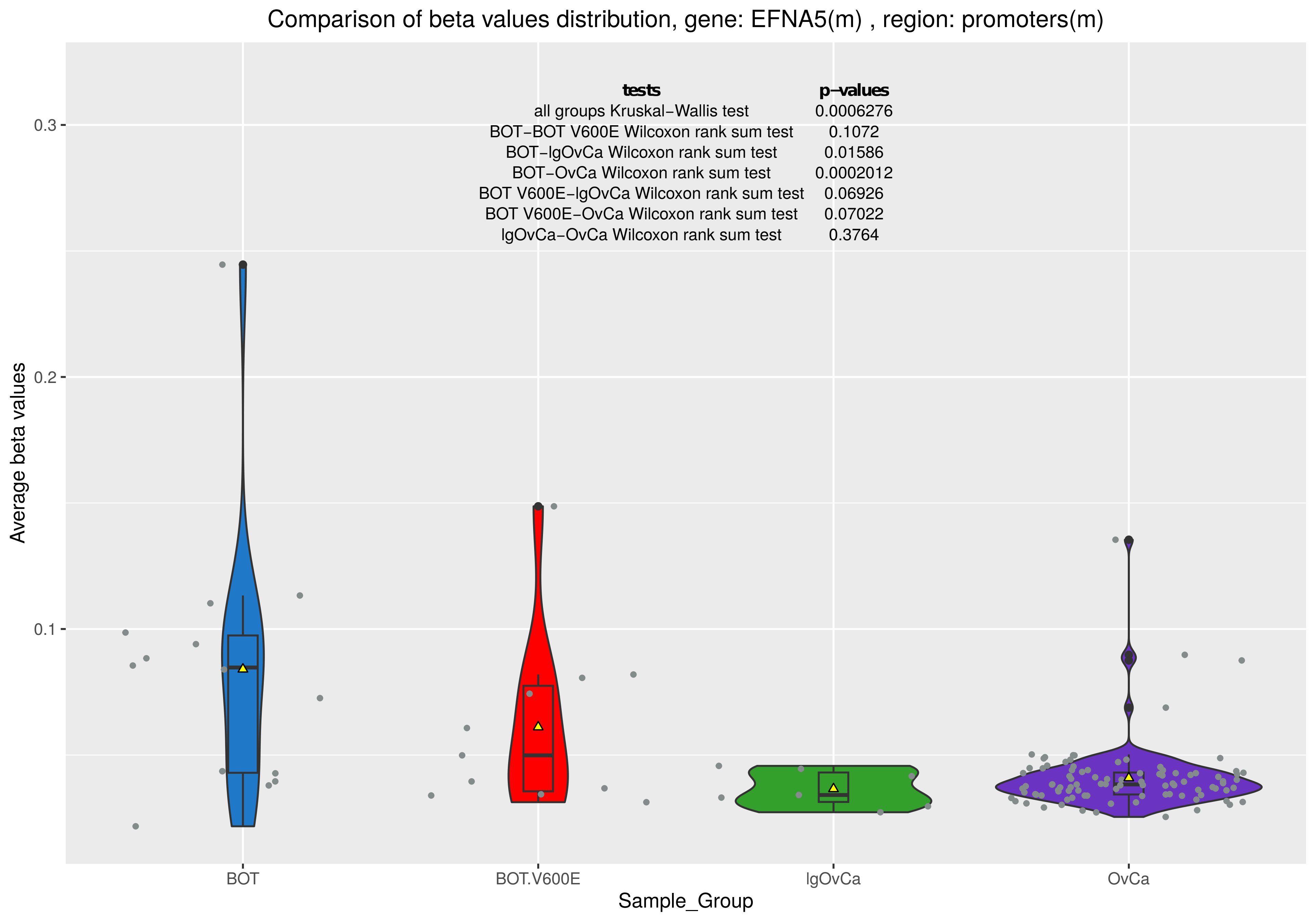

Comparison of beta values distribution, gene: EFNA5(m) , region: promoters(m)
tests
p−values
all groups Kruskal−Wallis test
0.0006276
BOT−BOT V600E Wilcoxon rank sum test
0.1072
BOT−IgOvCa Wilcoxon rank sum test
0.01586
BOT−OvCa Wilcoxon rank sum test
0.0002012
BOT V600E−IgOvCa Wilcoxon rank sum test
0.06926
BOT V600E−OvCa Wilcoxon rank sum test
0.07022
IgOvCa−OvCa Wilcoxon rank sum test
0.3764
Average beta values
0.3
0.2
0.1
BOT
BOT.V600E
lgOvCa
OvCa
Sample_Group

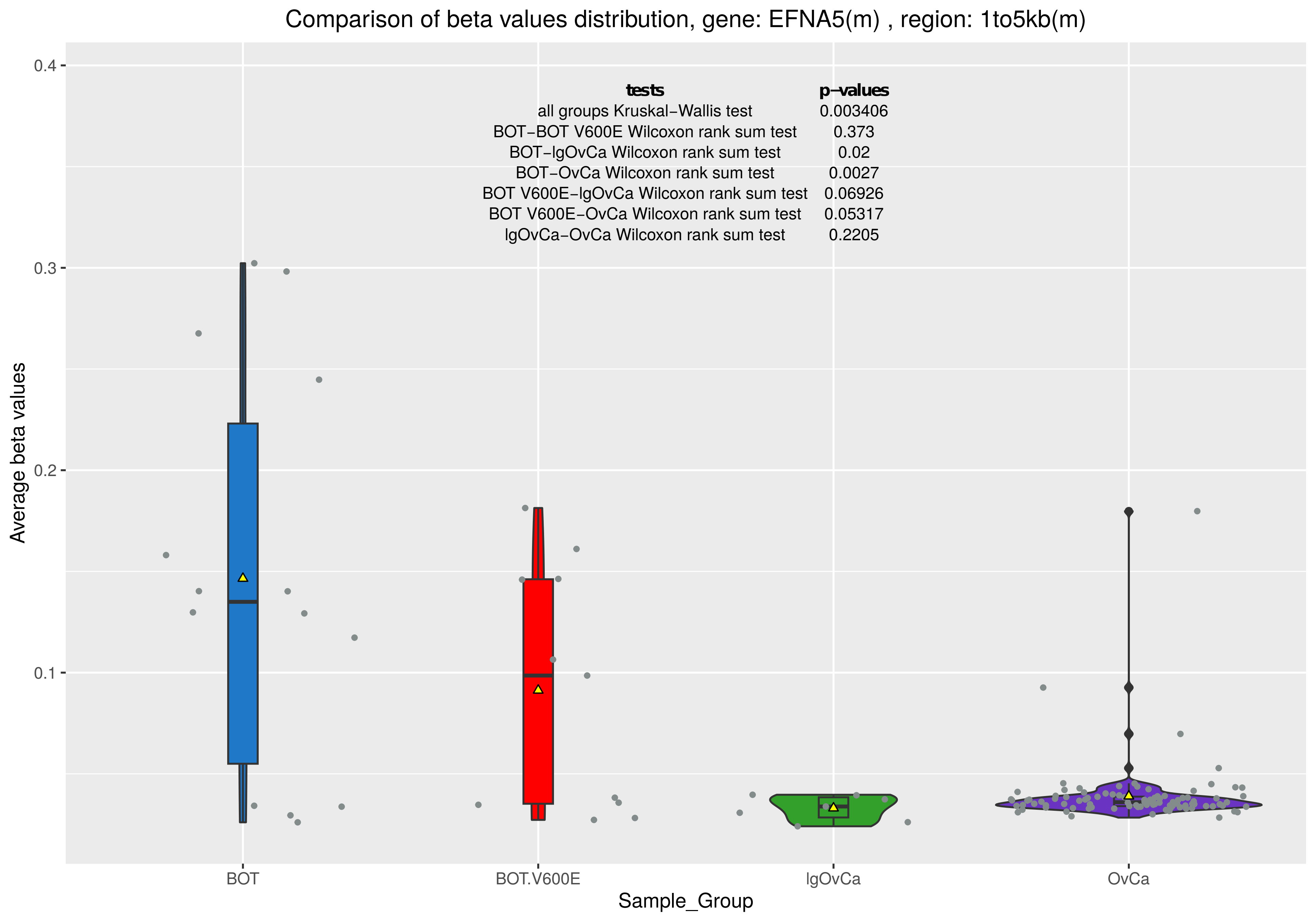
Comparison of beta values distribution, gene: EFNA5(m) , region: 1to5kb(m)
tests
p−values
all groups Kruskal−Wallis test
0.003406
BOT−BOT V600E Wilcoxon rank sum test
0.373
BOT−IgOvCa Wilcoxon rank sum test
0.02
BOT−OvCa Wilcoxon rank sum test
0.0027
BOT V600E−IgOvCa Wilcoxon rank sum test
0.06926
BOT V600E−OvCa Wilcoxon rank sum test
0.05317
IgOvCa−OvCa Wilcoxon rank sum test
0.2205
Average beta values
0.4
0.3
0.2
0.1
BOT
BOT.V600E
IgOvCa
OvCa
Sample_Group

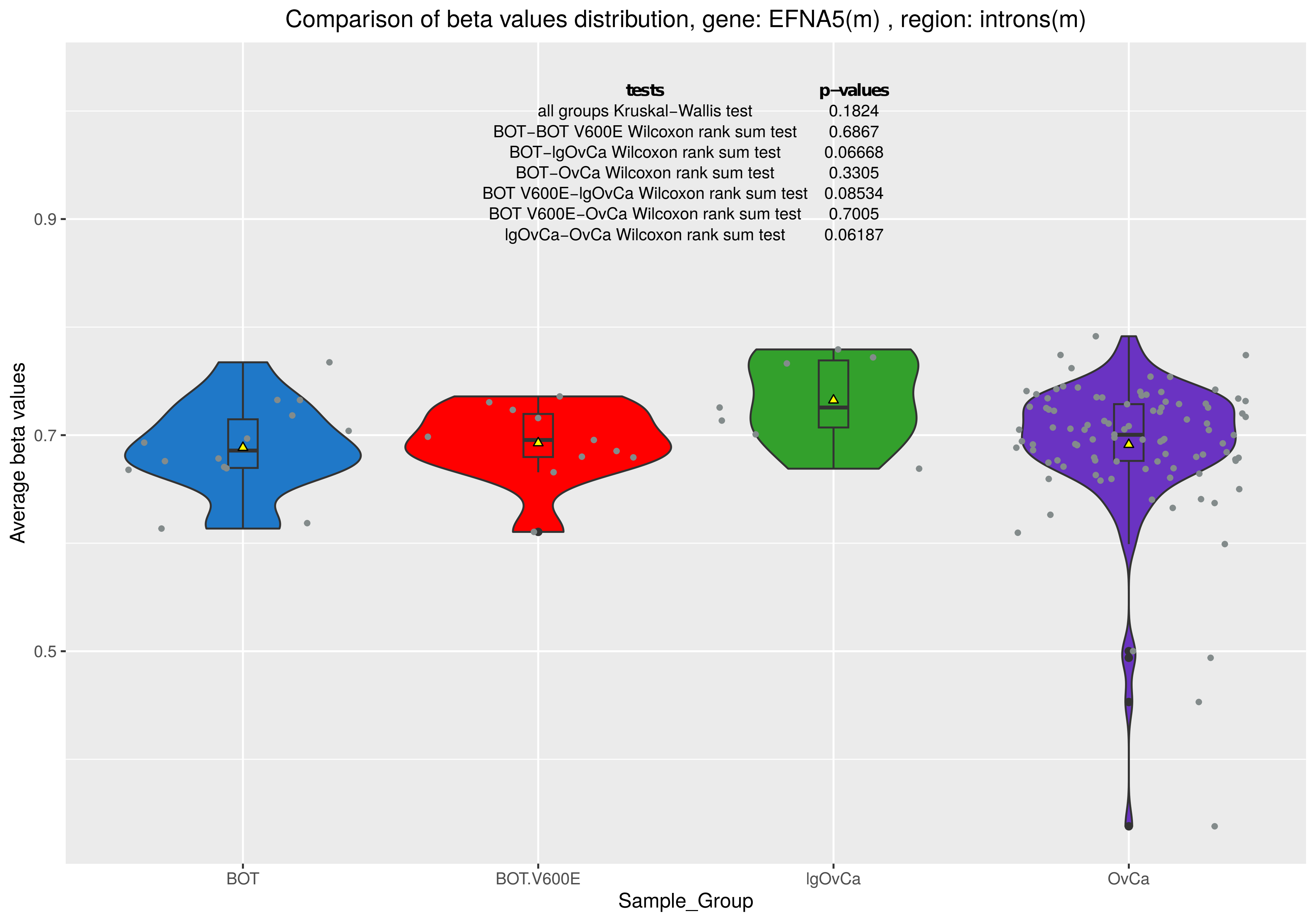

Comparison of beta values distribution, gene: EFNA5(m) , region: introns(m)
tests
p−values
all groups Kruskal−Wallis test
0.1824
BOT−BOT V600E Wilcoxon rank sum test
0.6867
BOT−lgOvCa Wilcoxon rank sum test
0.06668
BOT−OvCa Wilcoxon rank sum test
0.3305
BOT V600E−lgOvCa Wilcoxon rank sum test
0.08534
BOT V600E−OvCa Wilcoxon rank sum test
0.7005
lgOvCa−OvCa Wilcoxon rank sum test
0.06187
0.9
0.7
0.5
Average beta values
BOT
BOT.V600E
lgOvCa
OvCa
Sample_Group

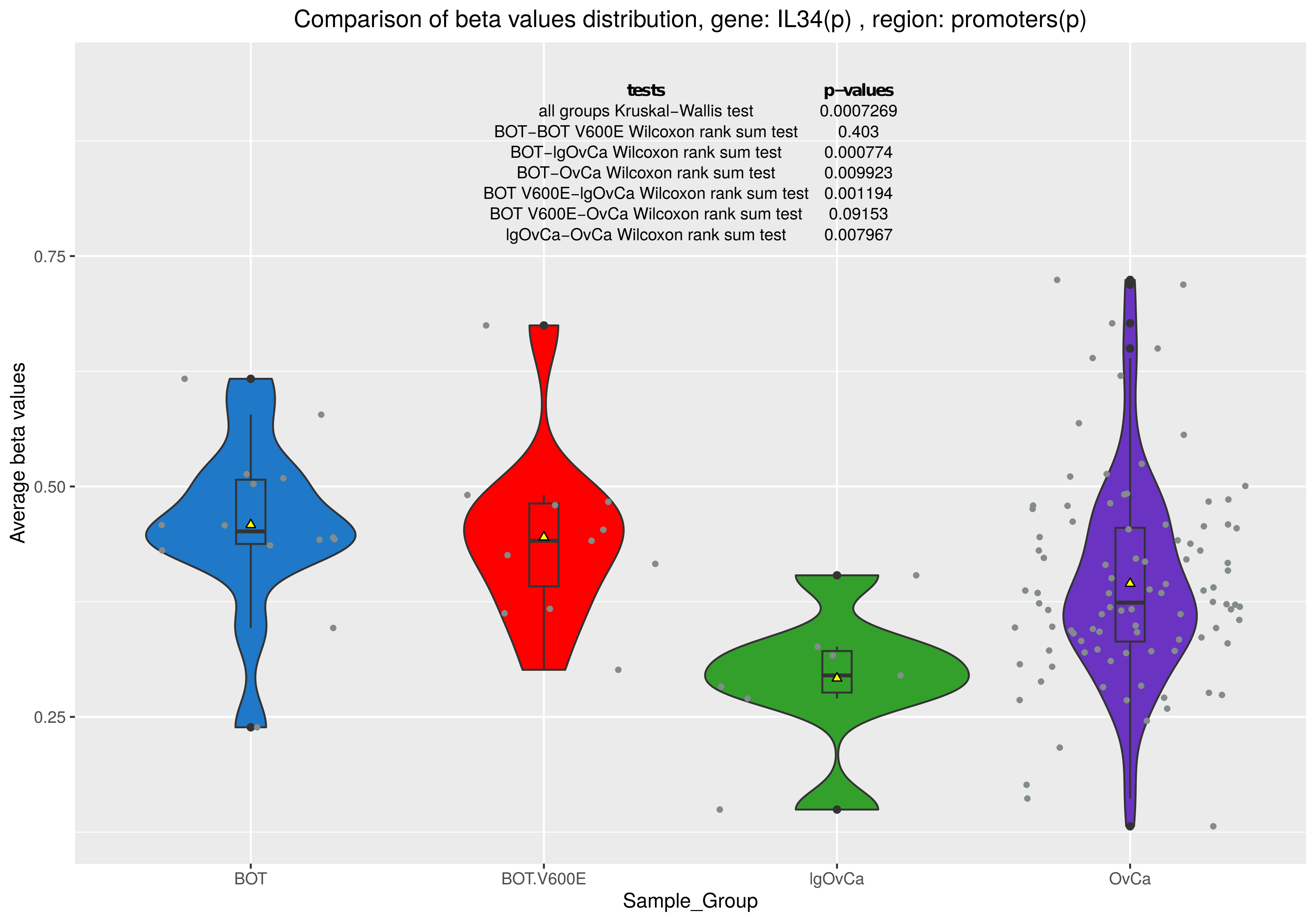

Comparison of beta values distribution, gene: IL34(p) , region: promoters(p)
tests
p−values
all groups Kruskal−Wallis test
0.0007269
BOT−BOT V600E Wilcoxon rank sum test
0.403
BOT−IgOvCa Wilcoxon rank sum test
0.000774
BOT−OvCa Wilcoxon rank sum test
0.009923
BOT V600E−IgOvCa Wilcoxon rank sum test
0.001194
BOT V600E−OvCa Wilcoxon rank sum test
0.09153
IgOvCa−OvCa Wilcoxon rank sum test
0.007967
Average beta values
0.75
0.50
0.25
BOT
BOT.V600E
IgOvCa
OvCa
Sample_Group

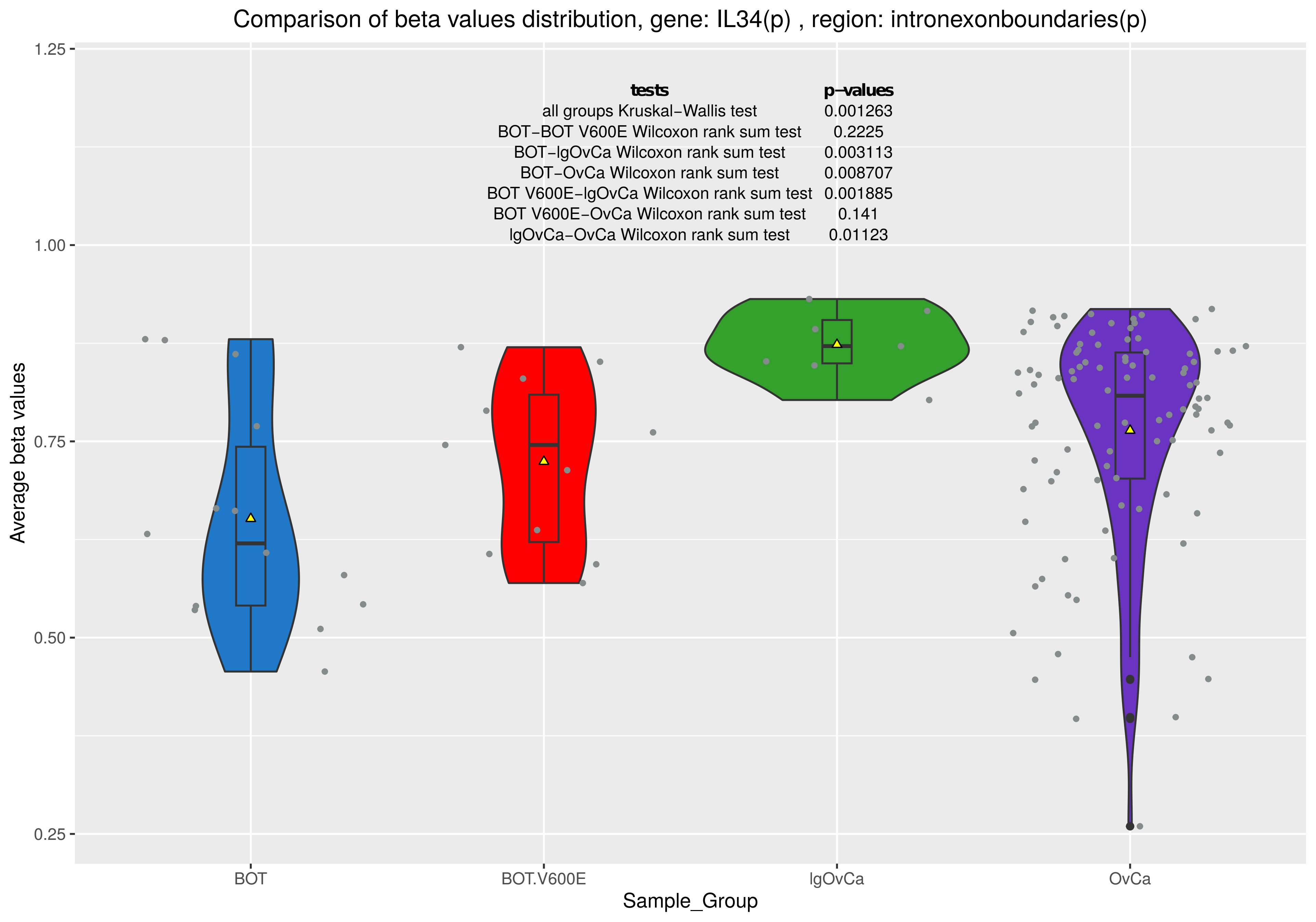

Comparison of beta values distribution, gene: IL34(p) , region: intronexonboundaries(p)
tests
p−values
all groups Kruskal−Wallis test
0.001263
BOT−BOT V600E Wilcoxon rank sum test
0.2225
BOT−lgOvCa Wilcoxon rank sum test
0.003113
BOT−OvCa Wilcoxon rank sum test
0.008707
BOT V600E−lgOvCa Wilcoxon rank sum test
0.001885
BOT V600E−OvCa Wilcoxon rank sum test
0.141
lgOvCa−OvCa Wilcoxon rank sum test
0.01123
Average beta values
1.25
1.00
0.75
0.50
0.25
BOT
BOT.V600E
lgOvCa
OvCa
Sample_Group

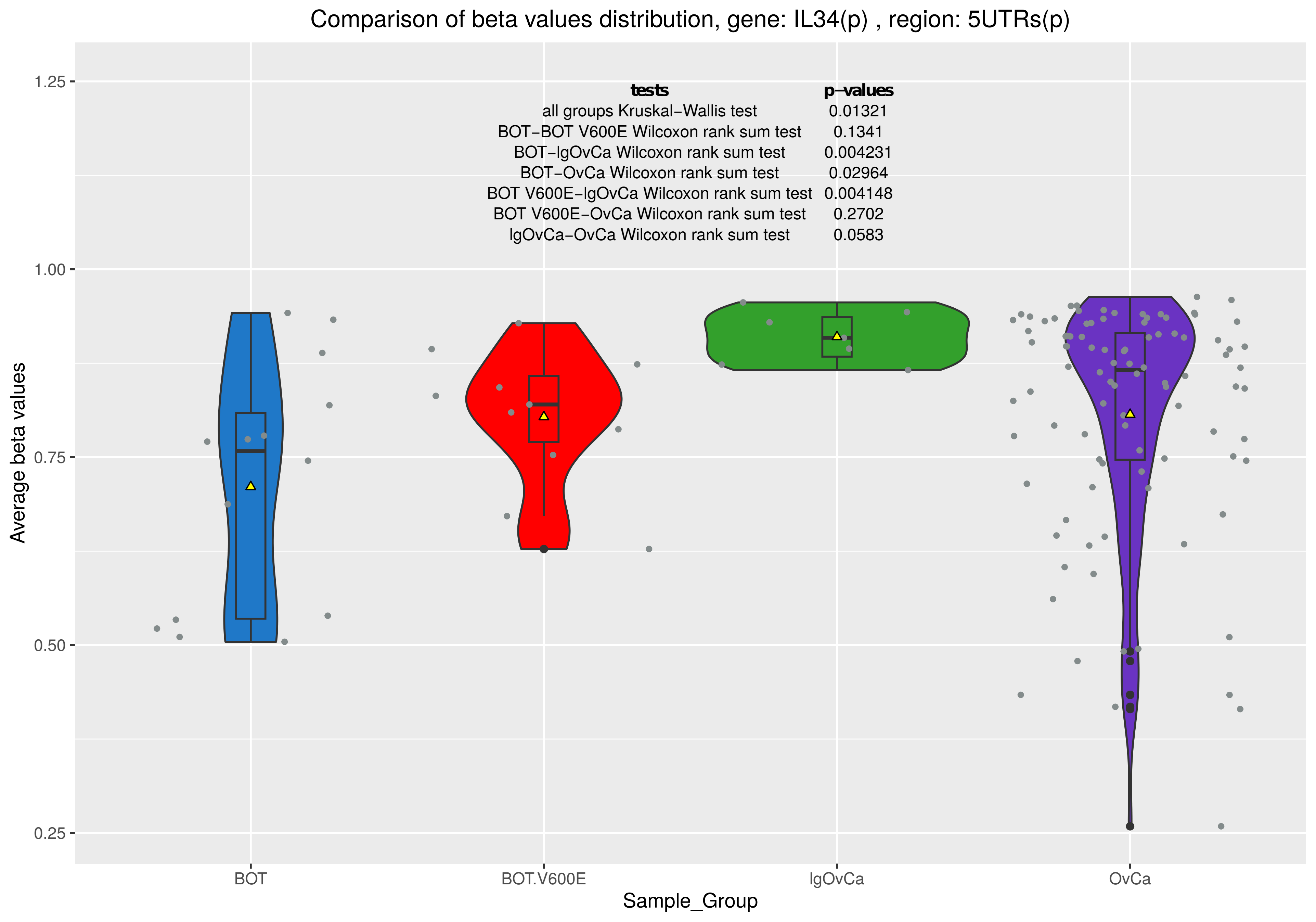
Comparison of beta values distribution, gene: IL34(p) , region: 5UTRs(p)
tests
p−values
all groups Kruskal−Wallis test
0.01321
BOT−BOT V600E Wilcoxon rank sum test
0.1341
BOT−lgOvCa Wilcoxon rank sum test
0.004231
BOT−OvCa Wilcoxon rank sum test
0.02964
BOT V600E−lgOvCa Wilcoxon rank sum test
0.004148
BOT V600E−OvCa Wilcoxon rank sum test
0.2702
lgOvCa−OvCa Wilcoxon rank sum test
0.0583
Average beta values
1.25
1.00
0.75
0.50
0.25
BOT
BOT.V600E
lgOvCa
OvCa
Sample_Group

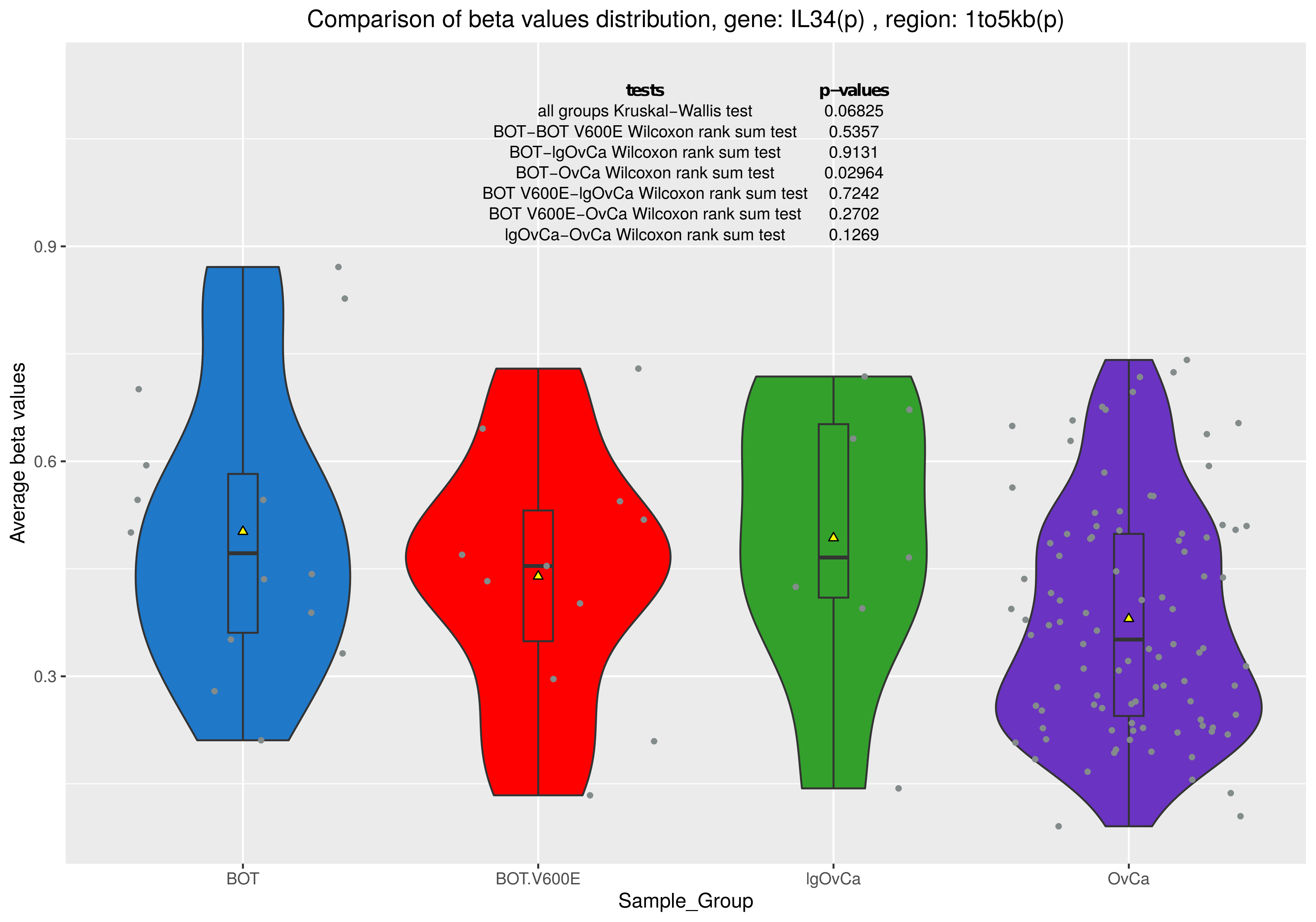
Comparison of beta values distribution, gene: IL34(p) , region: 1to5kb(p)
tests
p−values
all groups Kruskal−Wallis test
0.06825
BOT−BOT V600E Wilcoxon rank sum test
0.5357
BOT−lgOvCa Wilcoxon rank sum test
0.9131
BOT−OvCa Wilcoxon rank sum test
0.02964
BOT V600E−lgOvCa Wilcoxon rank sum test
0.7242
BOT V600E−OvCa Wilcoxon rank sum test
0.2702
lgOvCa−OvCa Wilcoxon rank sum test
0.1269
Average beta values
0.9
0.6
0.3
BOT
BOT.V600E
lgOvCa
OvCa
Sample_Group

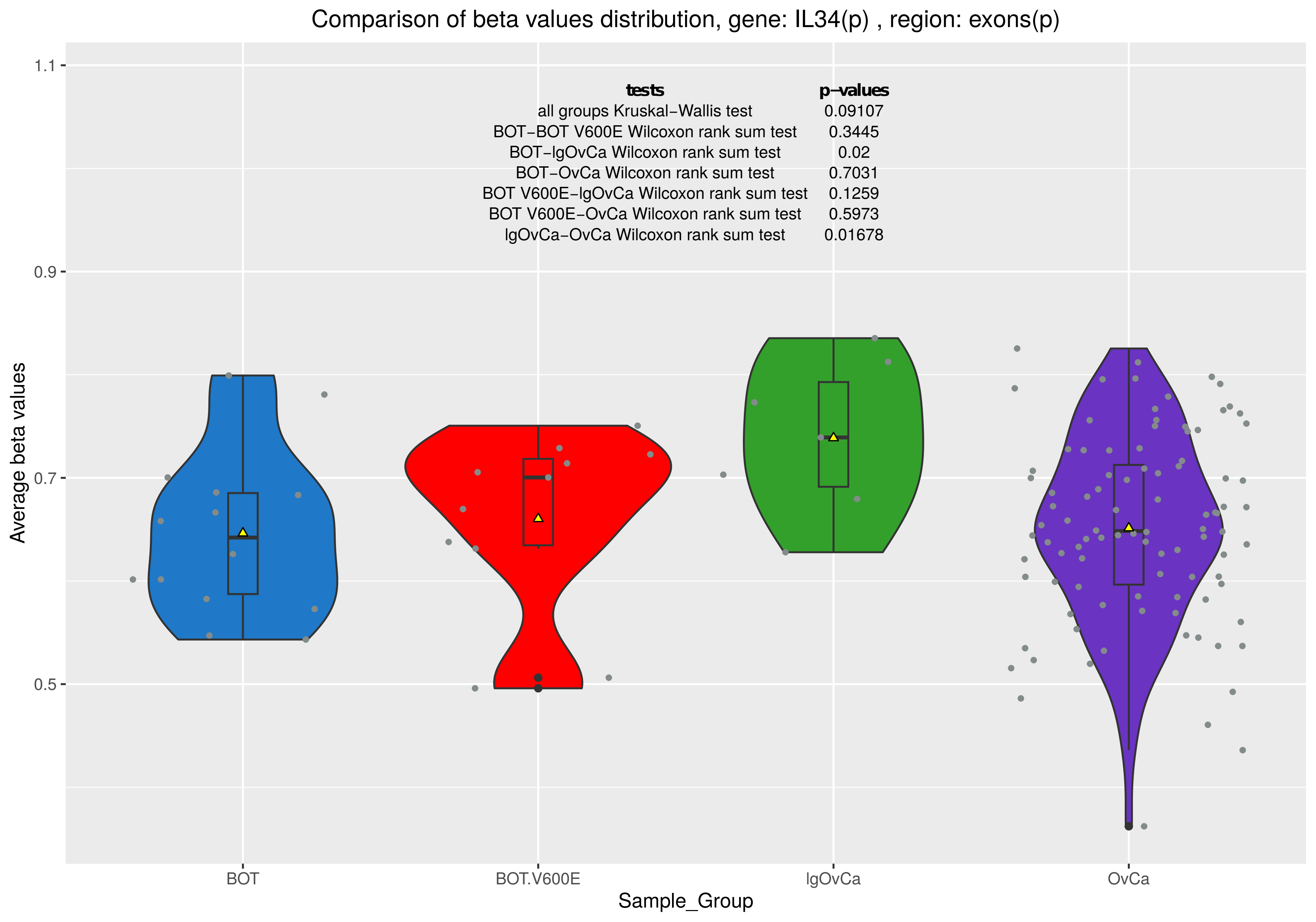

Comparison of beta values distribution, gene: IL34(p) , region: exons(p)
tests
p−values
all groups Kruskal−Wallis test
0.09107
BOT−BOT V600E Wilcoxon rank sum test
0.3445
BOT−IgOvCa Wilcoxon rank sum test
0.02
BOT−OvCa Wilcoxon rank sum test
0.7031
BOT V600E−IgOvCa Wilcoxon rank sum test
0.1259
BOT V600E−OvCa Wilcoxon rank sum test
0.5973
IgOvCa−OvCa Wilcoxon rank sum test
0.01678
Average beta values
1.1
0.9
0.7
0.5
BOT
BOT.V600E
IgOvCa
OvCa
Sample_Group

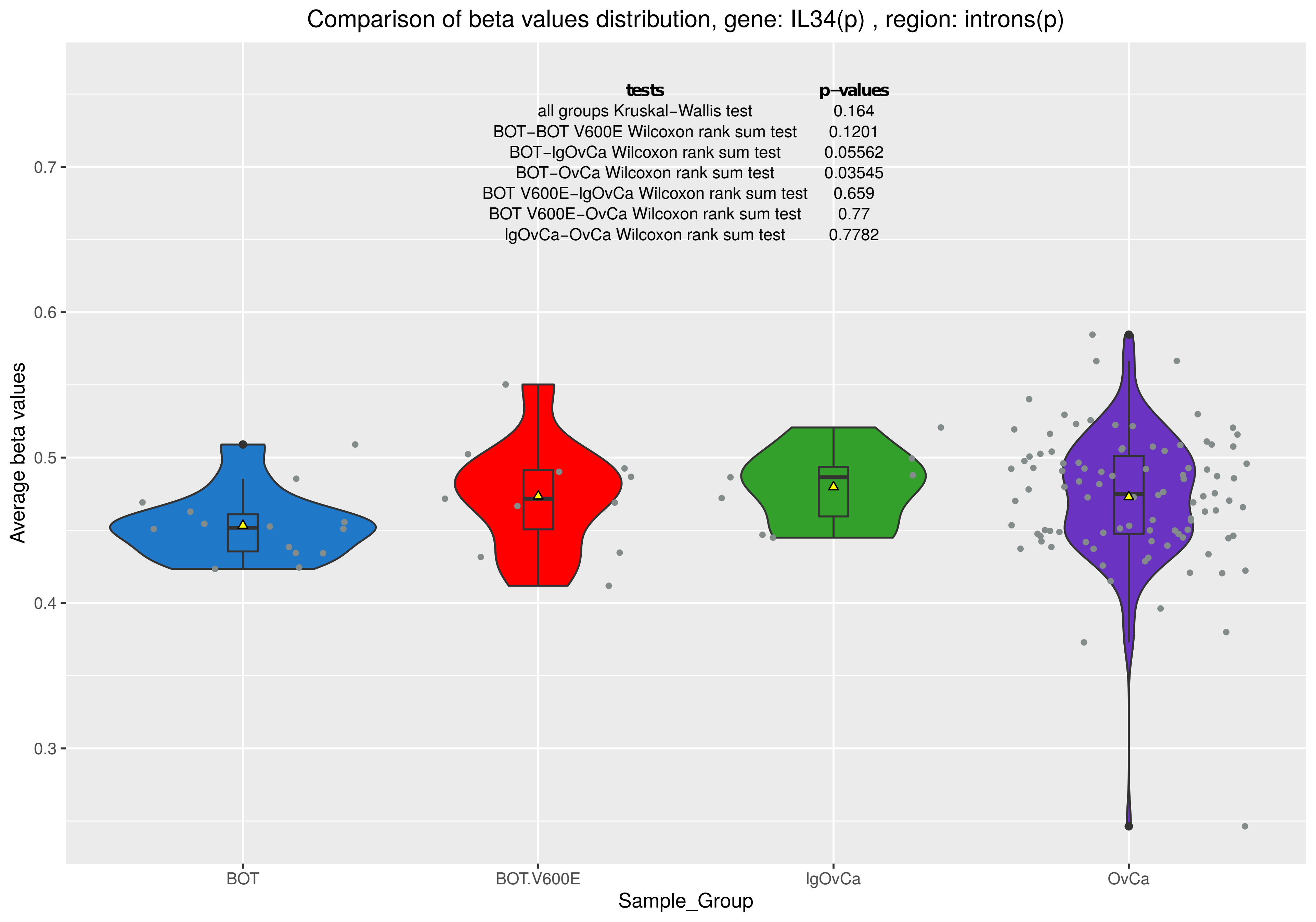

Comparison of beta values distribution, gene: IL34(p) , region: introns(p)
tests
p−values
all groups Kruskal−Wallis test
0.164
BOT−BOT V600E Wilcoxon rank sum test
0.1201
BOT−lgOvCa Wilcoxon rank sum test
0.05562
BOT−OvCa Wilcoxon rank sum test
0.03545
BOT V600E−lgOvCa Wilcoxon rank sum test
0.659
BOT V600E−OvCa Wilcoxon rank sum test
0.77
lgOvCa−OvCa Wilcoxon rank sum test
0.7782
Average beta values
0.7
0.6
0.5
0.4
0.3
BOT
BOT.V600E
lgOvCa
OvCa
Sample_Group

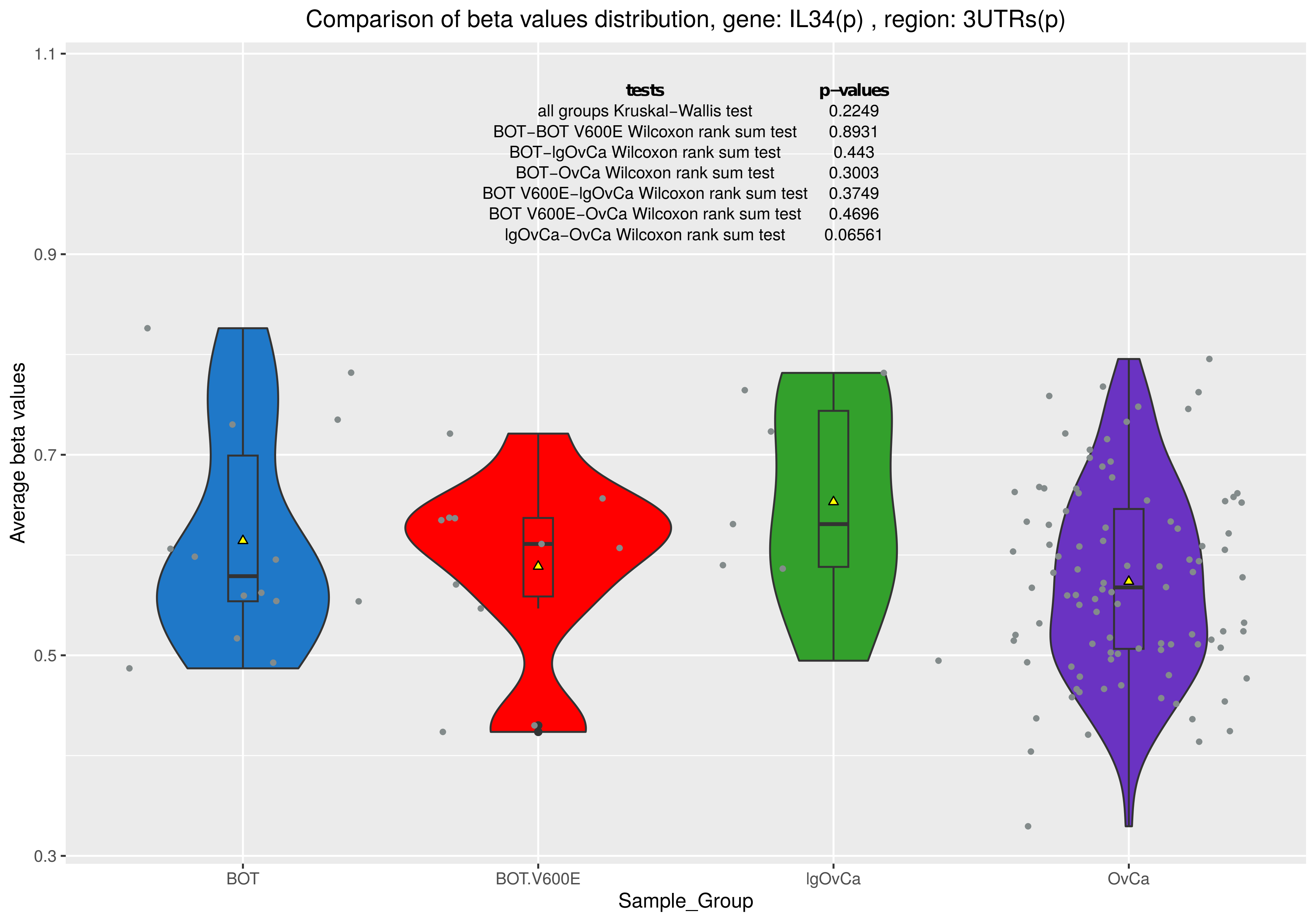

Comparison of beta values distribution, gene: IL34(p) , region: 3UTRs(p)
tests
p−values
all groups Kruskal−Wallis test
0.2249
BOT−BOT V600E Wilcoxon rank sum test
0.8931
BOT−IgOvCa Wilcoxon rank sum test
0.443
BOT−OvCa Wilcoxon rank sum test
0.3003
BOT V600E−IgOvCa Wilcoxon rank sum test
0.3749
BOT V600E−OvCa Wilcoxon rank sum test
0.4696
IgOvCa−OvCa Wilcoxon rank sum test
0.06561
Average beta values
1.1
0.9
0.7
0.5
0.3
BOT
BOT.V600E
IgOvCa
OvCa
Sample_Group

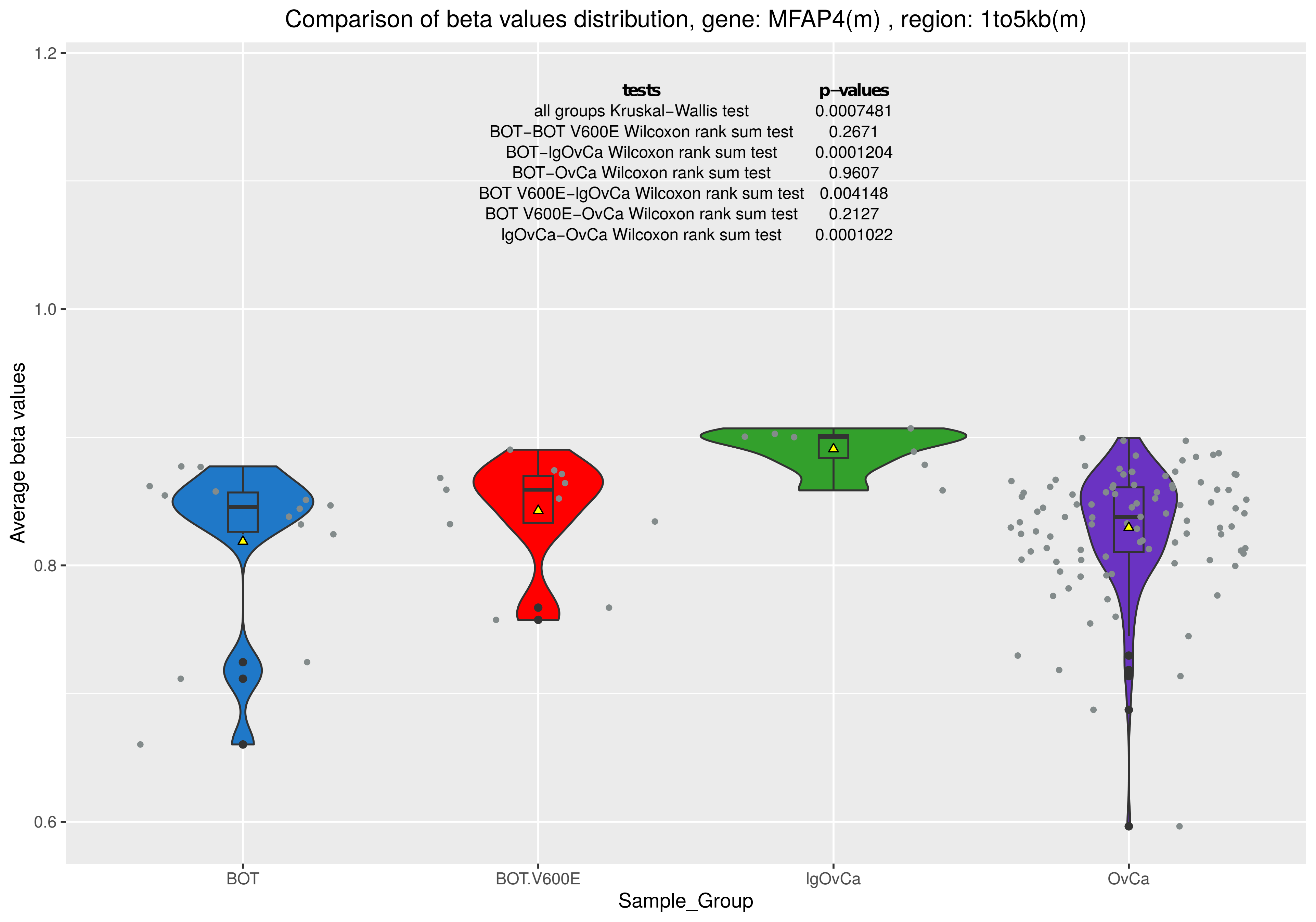

Comparison of beta values distribution, gene: MFAP4(m) , region: 1to5kb(m)
tests
p−values
all groups Kruskal−Wallis test
0.0007481
BOT−BOT V600E Wilcoxon rank sum test
0.2671
BOT−lgOvCa Wilcoxon rank sum test
0.0001204
BOT−OvCa Wilcoxon rank sum test
0.9607
BOT V600E−lgOvCa Wilcoxon rank sum test
0.004148
BOT V600E−OvCa Wilcoxon rank sum test
0.2127
lgOvCa−OvCa Wilcoxon rank sum test
0.0001022
1.2
1.0
0.8
0.6
Average beta values
BOT
BOT.V600E
lgOvCa
OvCa
Sample_Group

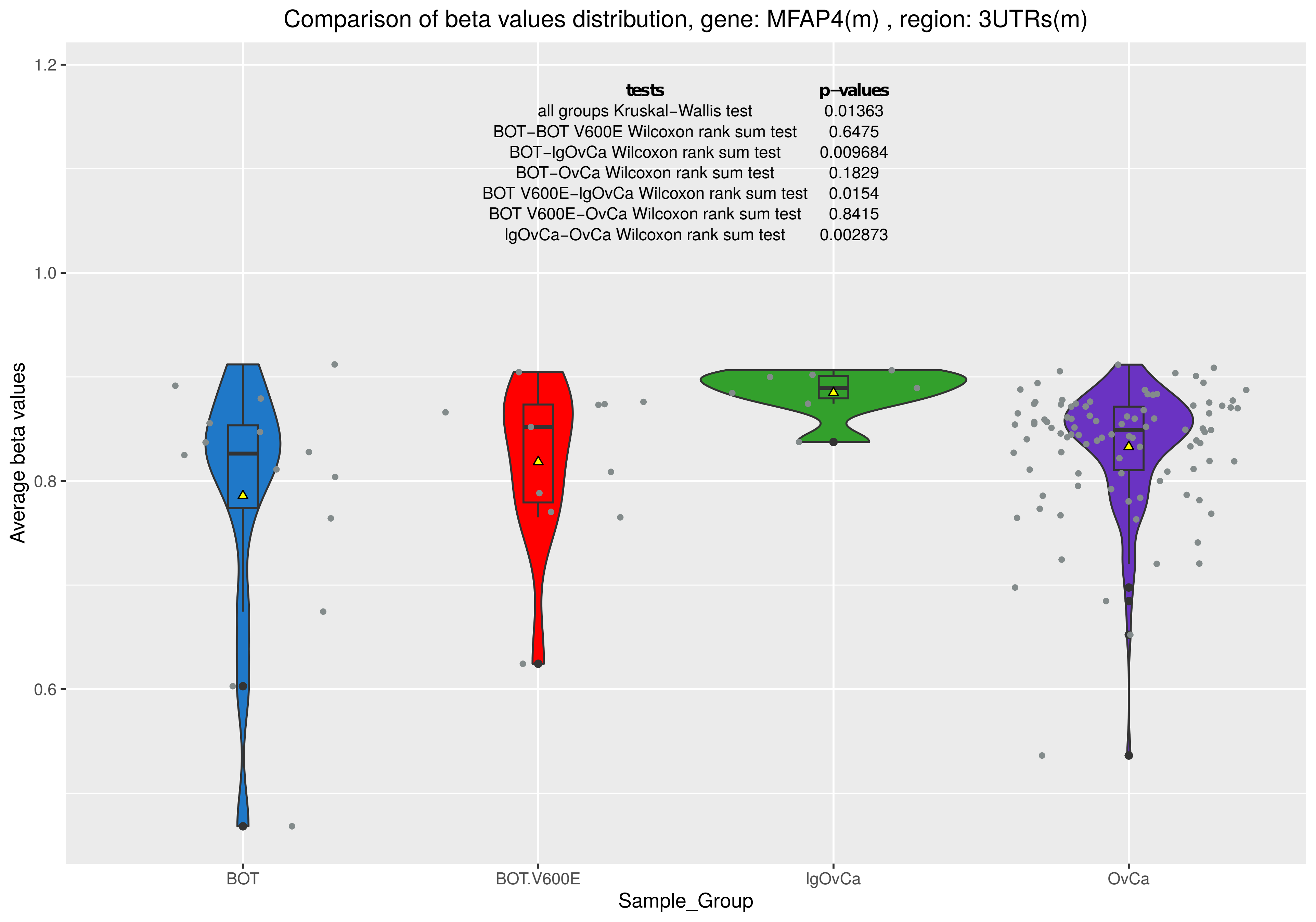

Comparison of beta values distribution, gene: MFAP4(m) , region: 3UTRs(m)
tests
p−values
all groups Kruskal−Wallis test
0.01363
BOT−BOT V600E Wilcoxon rank sum test
0.6475
BOT−lgOvCa Wilcoxon rank sum test
0.009684
BOT−OvCa Wilcoxon rank sum test
0.1829
BOT V600E−lgOvCa Wilcoxon rank sum test
0.0154
BOT V600E−OvCa Wilcoxon rank sum test
0.8415
lgOvCa−OvCa Wilcoxon rank sum test
0.002873
Average beta values
1.2
1.0
0.8
0.6
Sample_Group
BOT
BOT.V600E
lgOvCa
OvCa

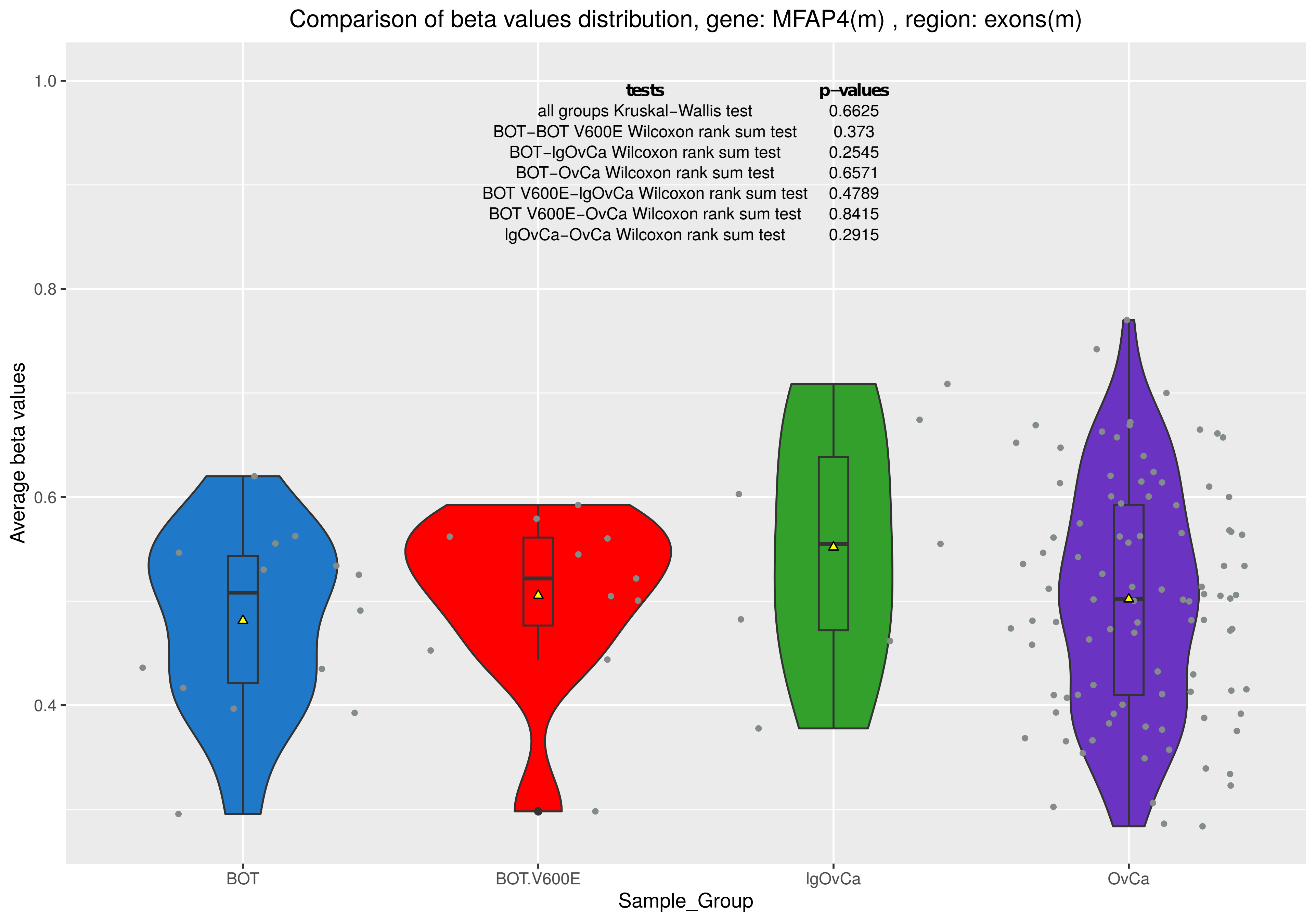
Comparison of beta values distribution, gene: MFAP4(m) , region: exons(m)
tests
p−values
all groups Kruskal−Wallis test
0.6625
BOT−BOT V600E Wilcoxon rank sum test
0.373
BOT−lgOvCa Wilcoxon rank sum test
0.2545
BOT−OvCa Wilcoxon rank sum test
0.6571
BOT V600E−lgOvCa Wilcoxon rank sum test
0.4789
BOT V600E−OvCa Wilcoxon rank sum test
0.8415
lgOvCa−OvCa Wilcoxon rank sum test
0.2915
Average beta values
1.0
0.8
0.6
0.4
BOT
BOT.V600E
lgOvCa
OvCa
Sample_Group

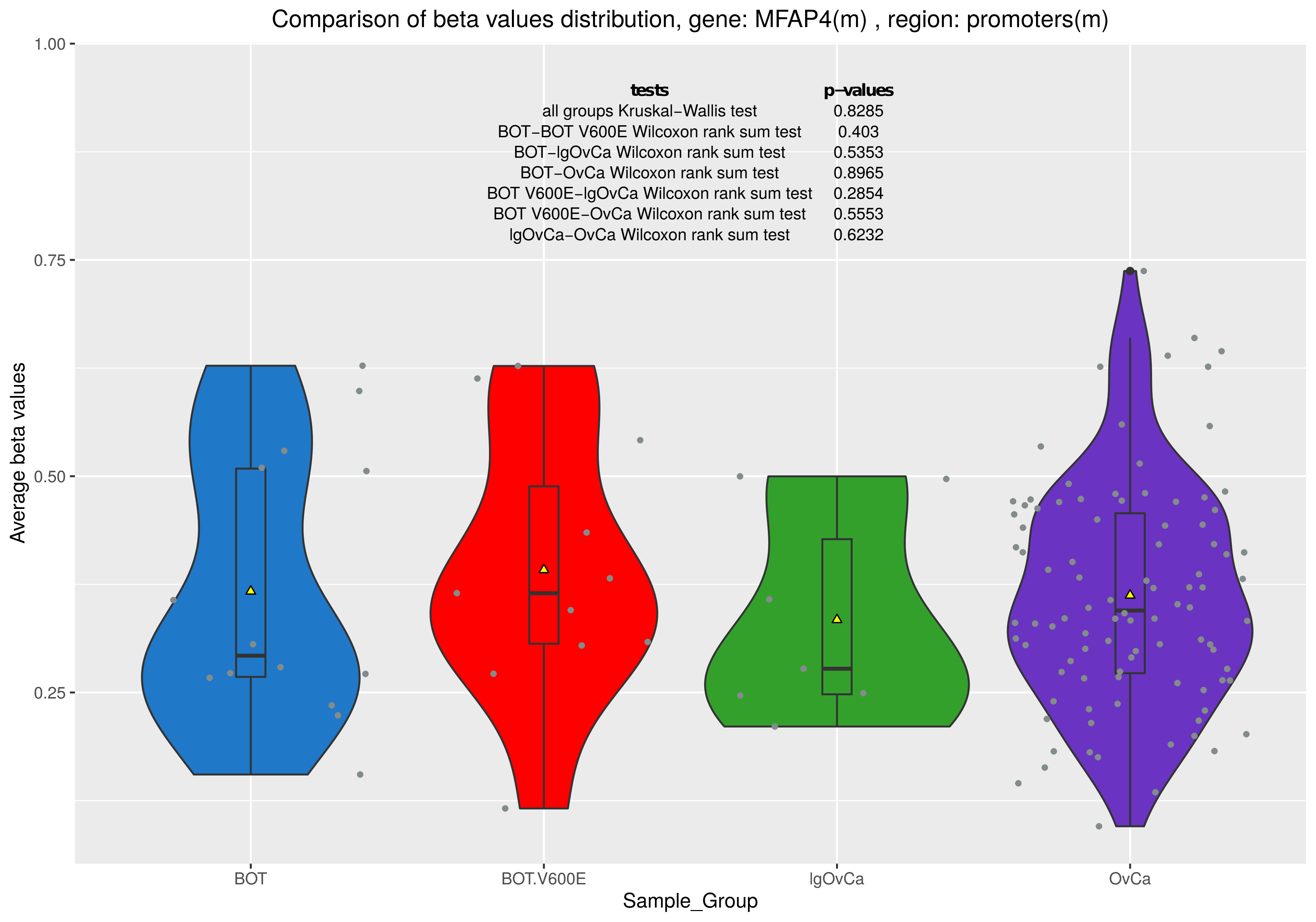

Comparison of beta values distribution, gene: MFAP4(m) , region: promoters(m)
tests
p−values
all groups Kruskal−Wallis test 0.8285
BOT−BOT V600E Wilcoxon rank sum test 0.403
BOT−lgOvCa Wilcoxon rank sum test 0.5353
BOT−OvCa Wilcoxon rank sum test 0.8965
BOT V600E−lgOvCa Wilcoxon rank sum test 0.2854
BOT V600E−OvCa Wilcoxon rank sum test 0.5553
lgOvCa−OvCa Wilcoxon rank sum test 0.6232
Average beta values
1.00
0.75
0.50
0.25
BOT
BOT.V600E
lgOvCa
OvCa
Sample_Group

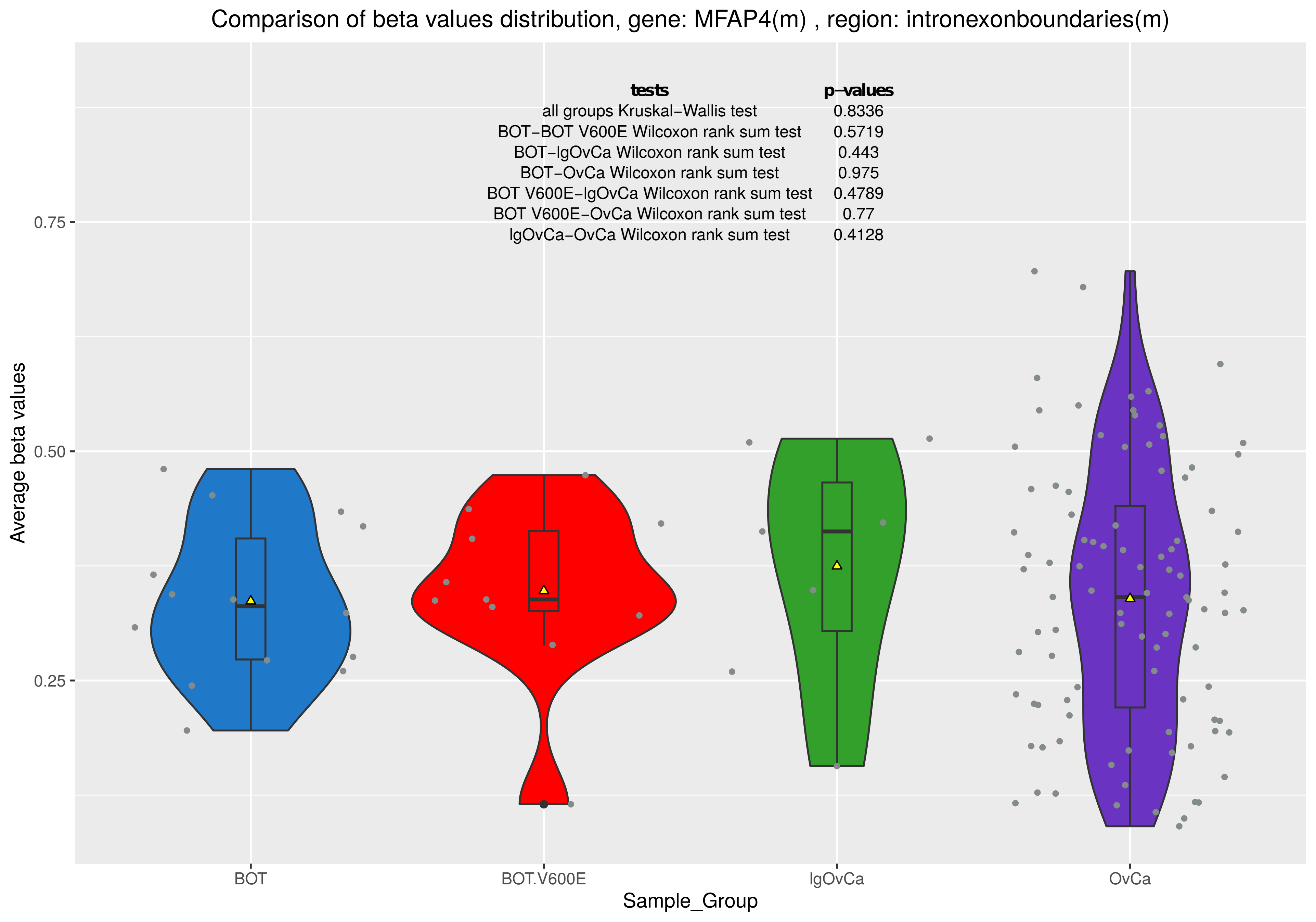
Comparison of beta values distribution, gene: MFAP4(m) , region: intronexonboundaries(m)
tests
p−values
all groups Kruskal−Wallis test 0.8336
BOT−BOT V600E Wilcoxon rank sum test 0.5719
BOT−lgOvCa Wilcoxon rank sum test 0.443
BOT−OvCa Wilcoxon rank sum test 0.975
BOT V600E−lgOvCa Wilcoxon rank sum test 0.4789
BOT V600E−OvCa Wilcoxon rank sum test 0.77
lgOvCa−OvCa Wilcoxon rank sum test 0.4128
0.75
0.50
0.25
Average beta values
BOT
BOT.V600E
lgOvCa
OvCa
Sample_Group

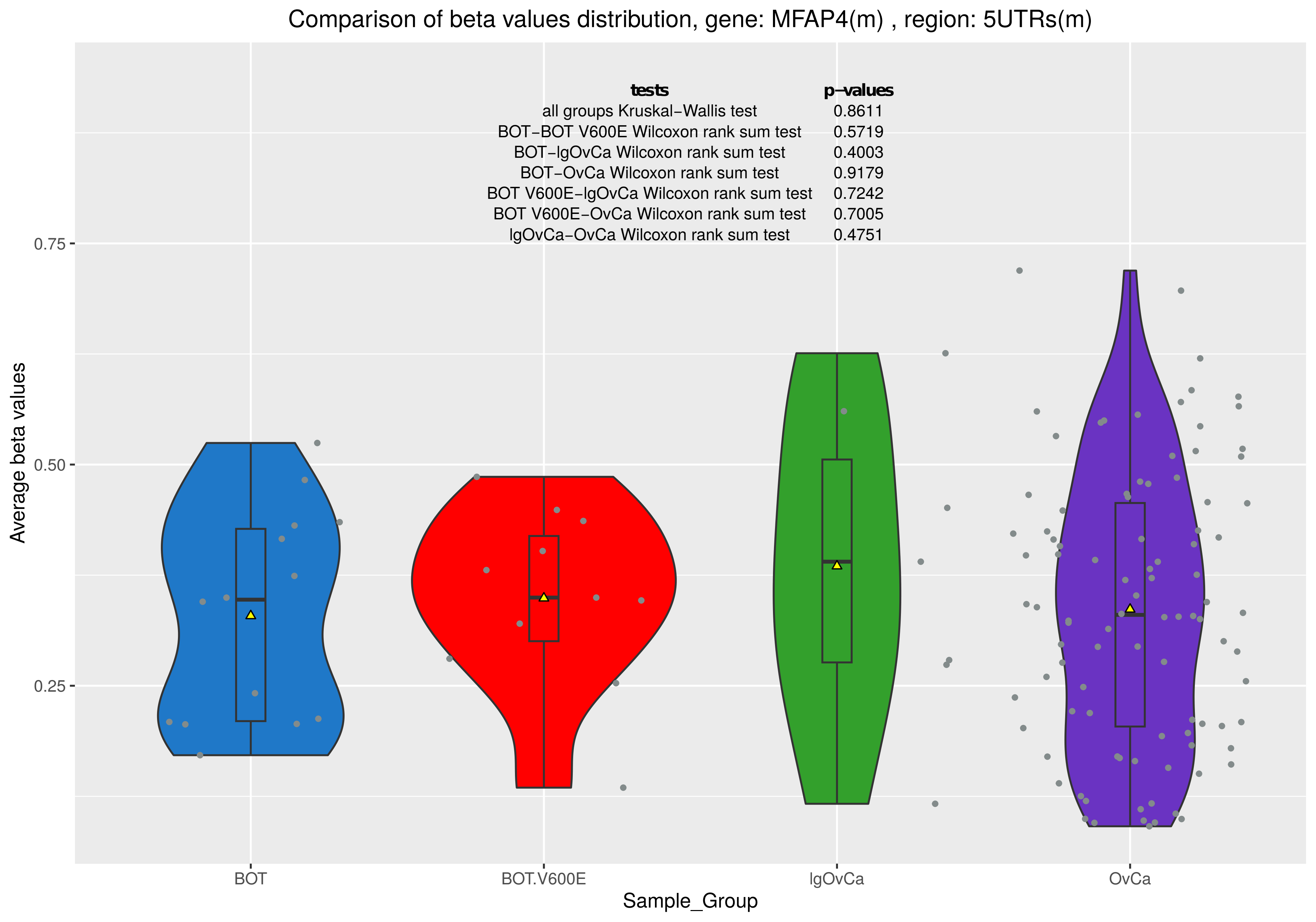

Comparison of beta values distribution, gene: MFAP4(m) , region: 5UTRs(m)
tests
p−values
all groups Kruskal−Wallis test
0.8611
BOT−BOT V600E Wilcoxon rank sum test
0.5719
BOT−lgOvCa Wilcoxon rank sum test
0.4003
BOT−OvCa Wilcoxon rank sum test
0.9179
BOT V600E−lgOvCa Wilcoxon rank sum test
0.7242
BOT V600E−OvCa Wilcoxon rank sum test
0.7005
lgOvCa−OvCa Wilcoxon rank sum test
0.4751
Average beta values
0.75
0.50
0.25
BOT
BOT.V600E
lgOvCa
OvCa
Sample_Group

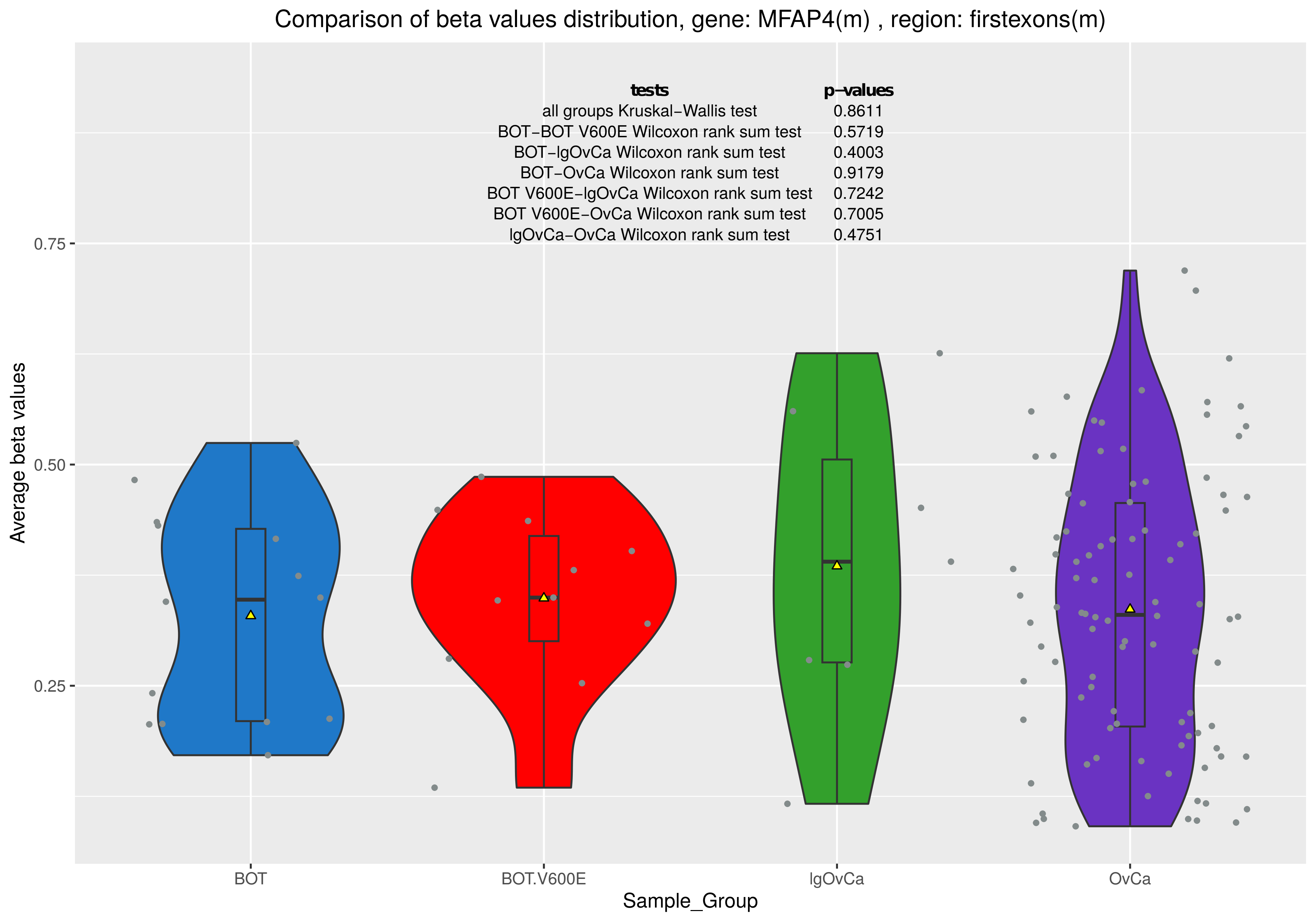
Comparison of beta values distribution, gene: MFAP4(m) , region: firstexons(m)
tests
p−values
all groups Kruskal−Wallis test
0.8611
BOT−BOT V600E Wilcoxon rank sum test
0.5719
BOT−lgOvCa Wilcoxon rank sum test
0.4003
BOT−OvCa Wilcoxon rank sum test
0.9179
BOT V600E−lgOvCa Wilcoxon rank sum test
0.7242
BOT V600E−OvCa Wilcoxon rank sum test
0.7005
lgOvCa−OvCa Wilcoxon rank sum test
0.4751
Average beta values
0.75
0.50
0.25
BOT
BOT.V600E
lgOvCa
OvCa
Sample_Group

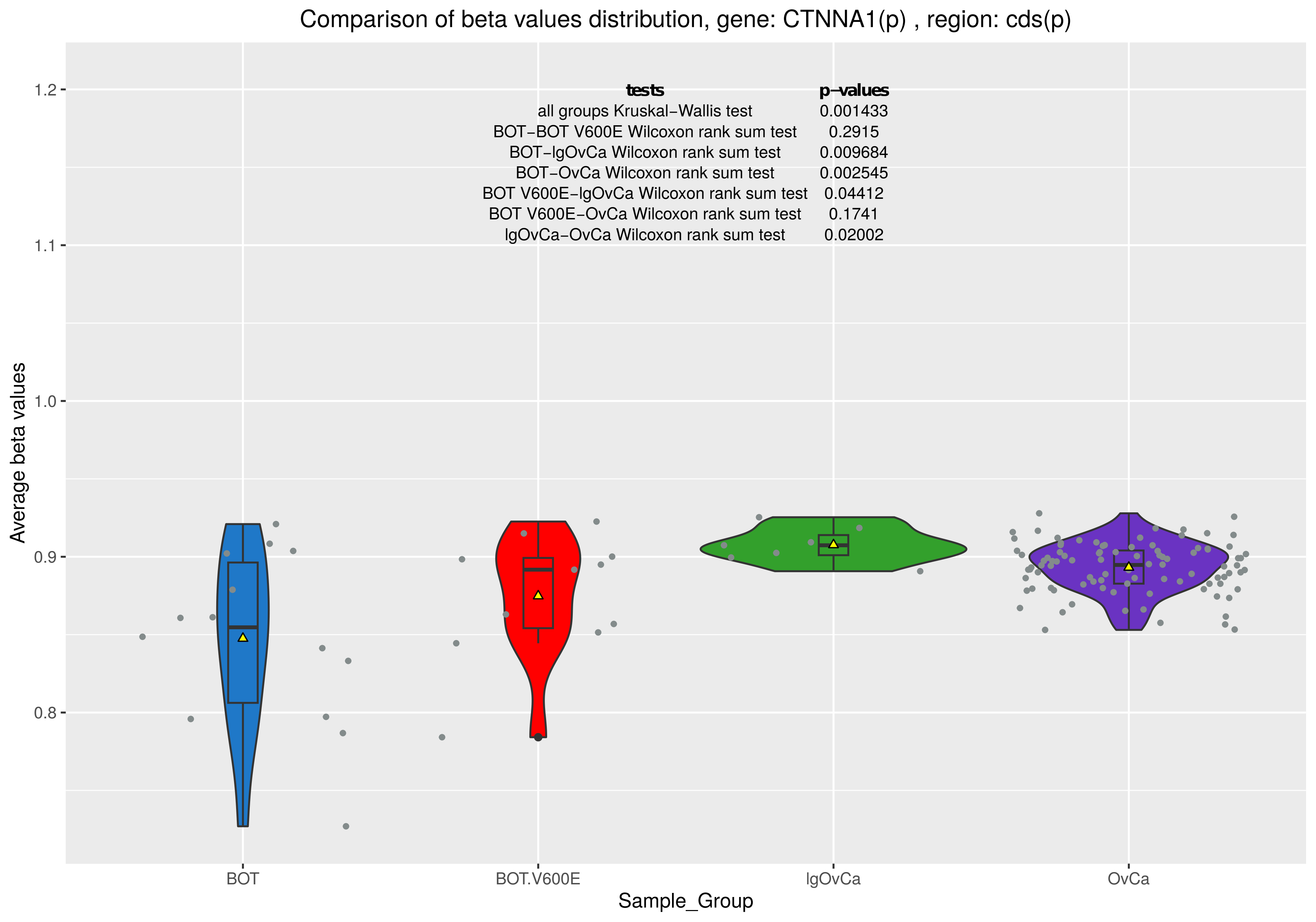

Comparison of beta values distribution, gene: CTNNA1(p) , region: cds(p)
tests
p−values
all groups Kruskal−Wallis test 0.001433
BOT−BOT V600E Wilcoxon rank sum test 0.2915
BOT−lgOvCa Wilcoxon rank sum test 0.009684
BOT−OvCa Wilcoxon rank sum test 0.002545
BOT V600E−lgOvCa Wilcoxon rank sum test 0.04412
BOT V600E−OvCa Wilcoxon rank sum test 0.1741
lgOvCa−OvCa Wilcoxon rank sum test 0.02002
Average beta values
1.2
1.1
1.0
0.9
0.8
BOT
BOT.V600E
lgOvCa
OvCa
Sample_Group

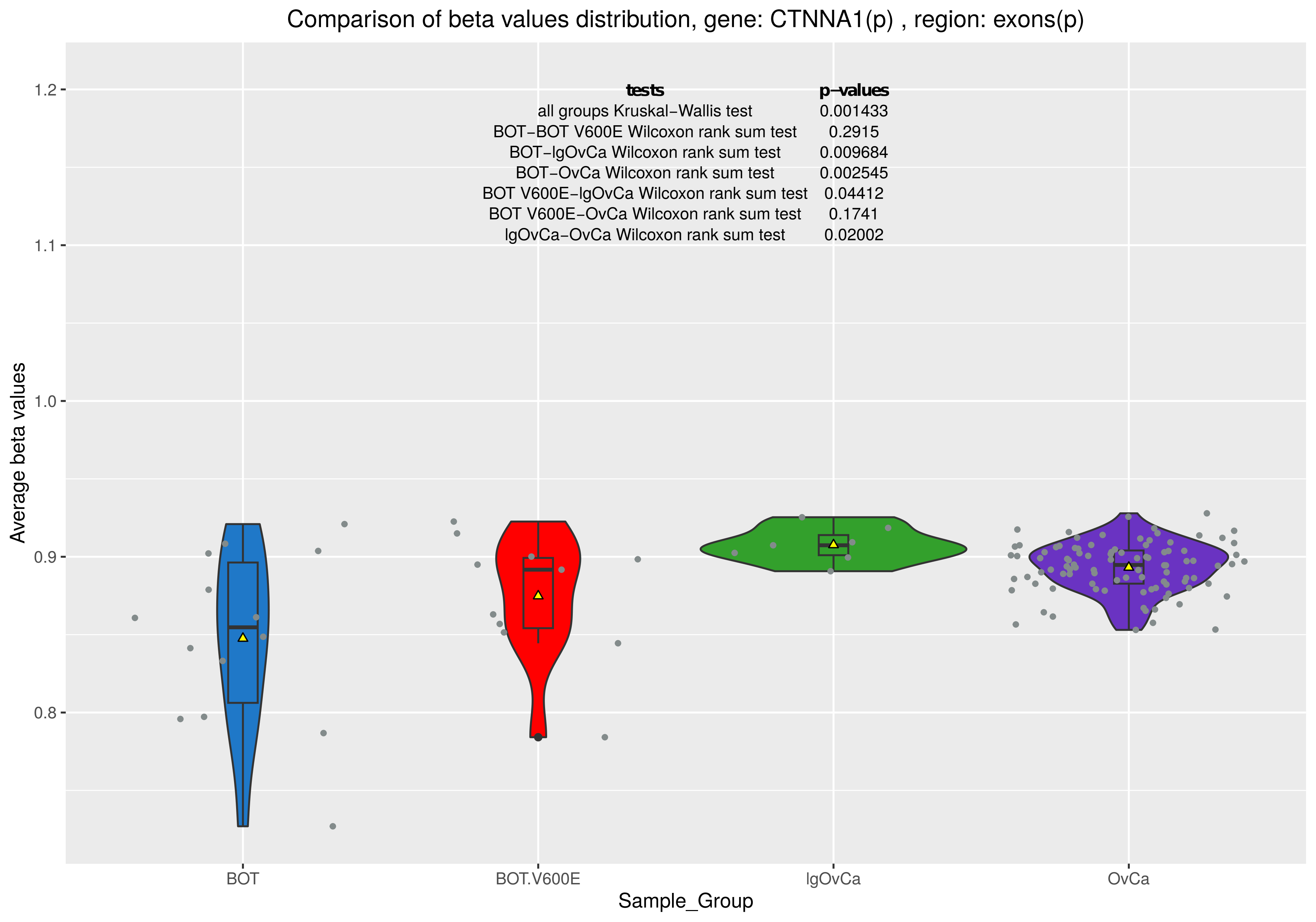
Comparison of beta values distribution, gene: CTNNA1(p) , region: exons(p)
tests
p−values
all groups Kruskal−Wallis test
0.001433
BOT−BOT V600E Wilcoxon rank sum test
0.2915
BOT−lgOvCa Wilcoxon rank sum test
0.009684
BOT−OvCa Wilcoxon rank sum test
0.002545
BOT V600E−lgOvCa Wilcoxon rank sum test
0.04412
BOT V600E−OvCa Wilcoxon rank sum test
0.1741
lgOvCa−OvCa Wilcoxon rank sum test
0.02002
Average beta values
1.2
1.1
1.0
0.9
0.8
BOT
BOT.V600E
lgOvCa
OvCa
Sample_Group

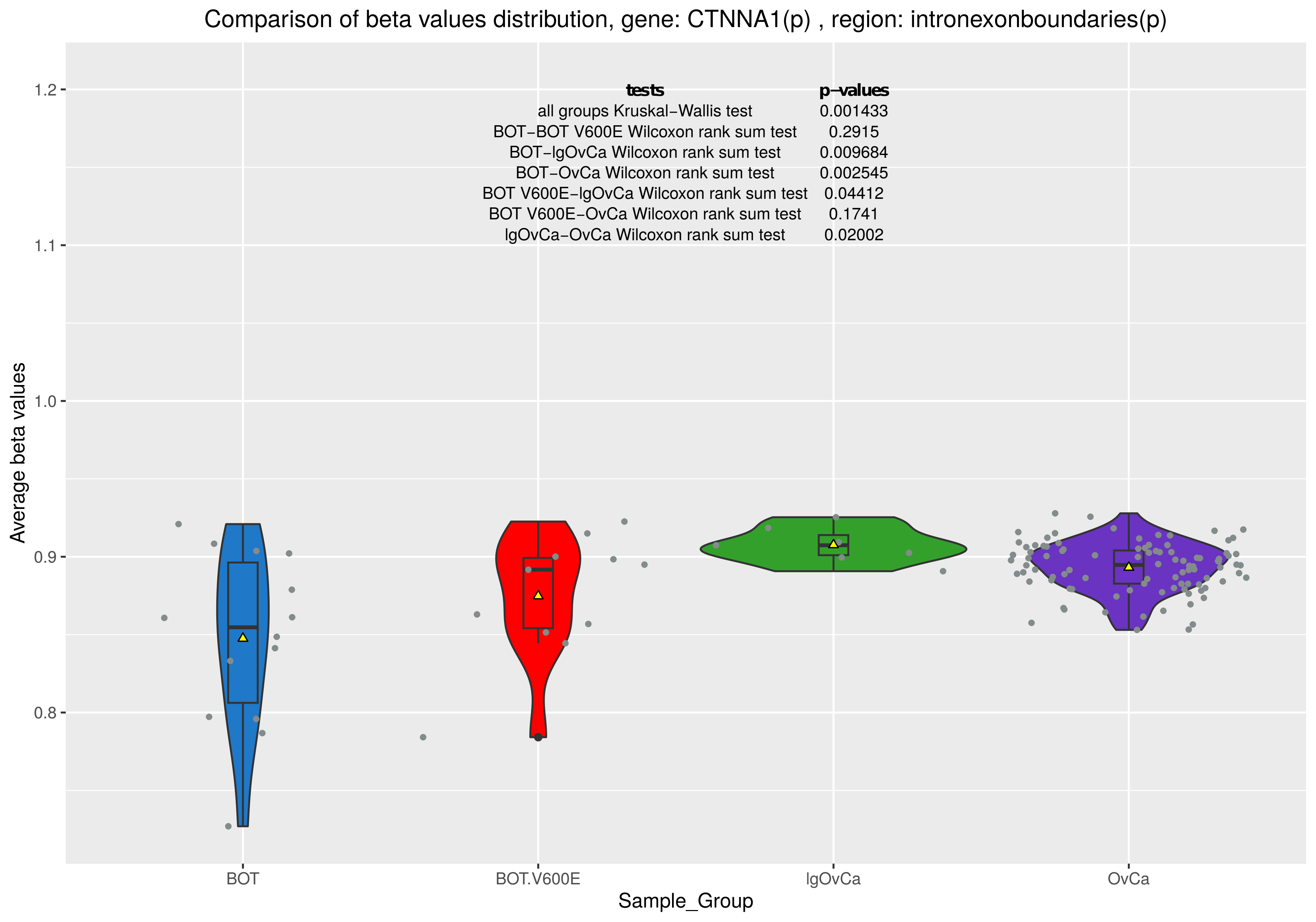

Comparison of beta values distribution, gene: CTNNA1(p) , region: intronexonboundaries(p)
tests
p−values
all groups Kruskal−Wallis test
0.001433
BOT−BOT V600E Wilcoxon rank sum test
0.2915
BOT−lgOvCa Wilcoxon rank sum test
0.009684
BOT−OvCa Wilcoxon rank sum test
0.002545
BOT V600E−lgOvCa Wilcoxon rank sum test
0.04412
BOT V600E−OvCa Wilcoxon rank sum test
0.1741
lgOvCa−OvCa Wilcoxon rank sum test
0.02002
Average beta values
1.2
1.1
1.0
0.9
0.8
BOT
BOT.V600E
lgOvCa
OvCa
Sample_Group

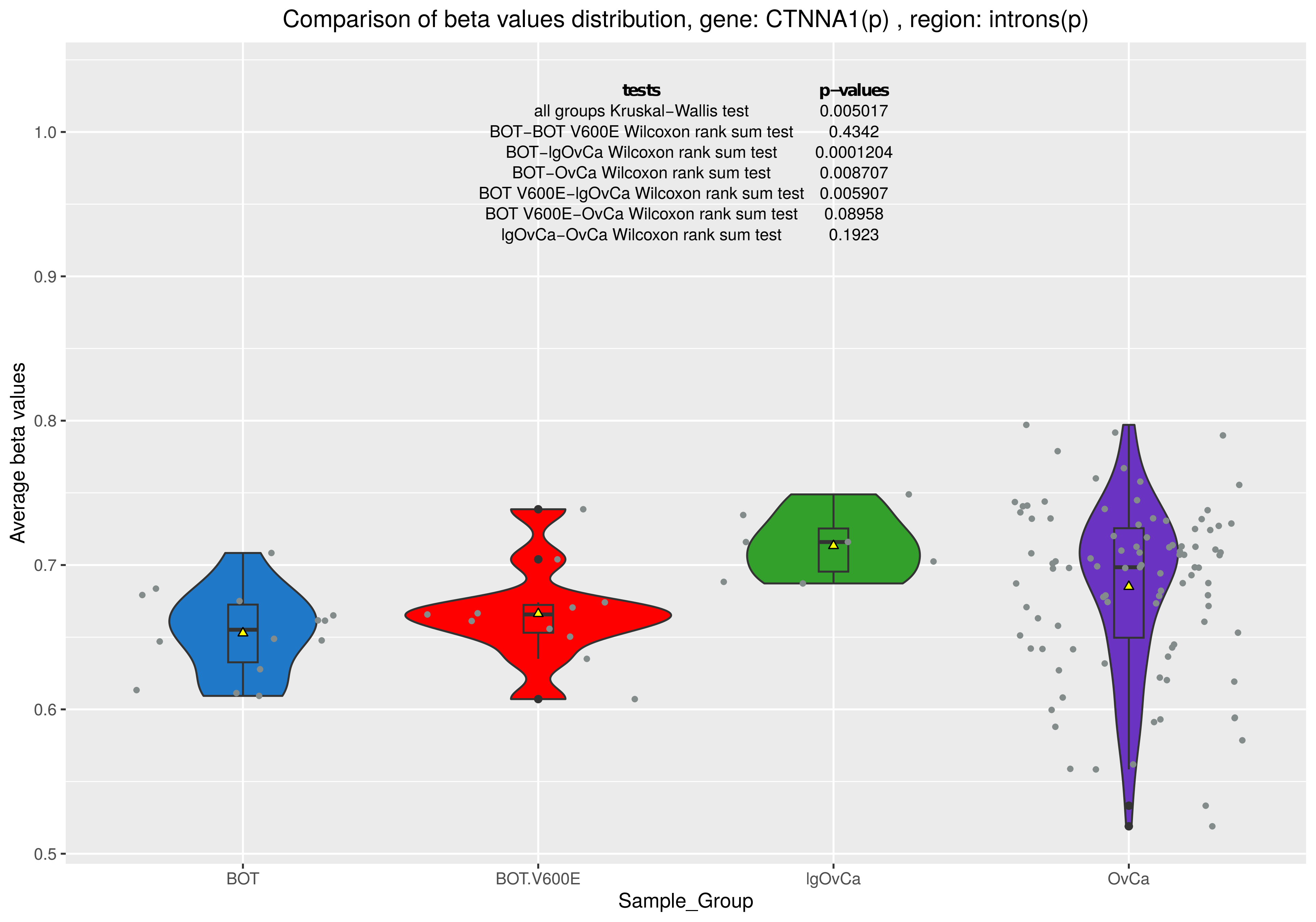

Comparison of beta values distribution, gene: CTNNA1(p) , region: introns(p)
tests
p−values
all groups Kruskal−Wallis test
0.005017
BOT−BOT V600E Wilcoxon rank sum test
0.4342
BOT−lgOvCa Wilcoxon rank sum test
0.0001204
BOT−OvCa Wilcoxon rank sum test
0.008707
BOT V600E−lgOvCa Wilcoxon rank sum test
0.005907
BOT V600E−OvCa Wilcoxon rank sum test
0.08958
lgOvCa−OvCa Wilcoxon rank sum test
0.1923
Average beta values
1.0
0.9
0.8
0.7
0.6
0.5
BOT
BOT.V600E
lgOvCa
OvCa
Sample_Group

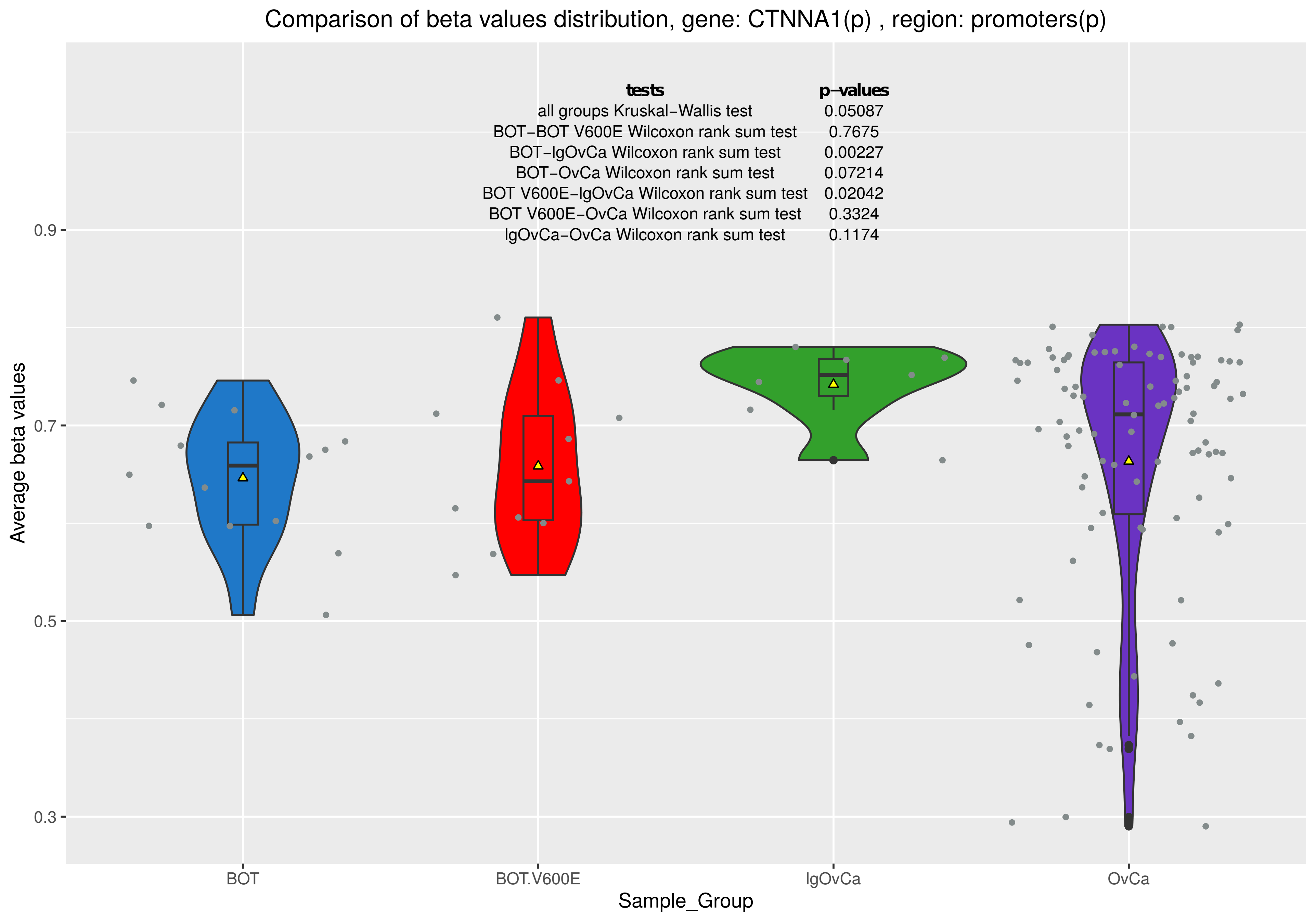
Comparison of beta values distribution, gene: CTNNA1(p) , region: promoters(p)
tests
p−values
all groups Kruskal−Wallis test
0.05087
BOT−BOT V600E Wilcoxon rank sum test
0.7675
BOT−IgOvCa Wilcoxon rank sum test
0.00227
BOT−OvCa Wilcoxon rank sum test
0.07214
BOT V600E−IgOvCa Wilcoxon rank sum test
0.02042
BOT V600E−OvCa Wilcoxon rank sum test
0.3324
IgOvCa−OvCa Wilcoxon rank sum test
0.1174
Average beta values
0.9
0.7
0.5
0.3
BOT
BOT.V600E
IgOvCa
OvCa
Sample_Group

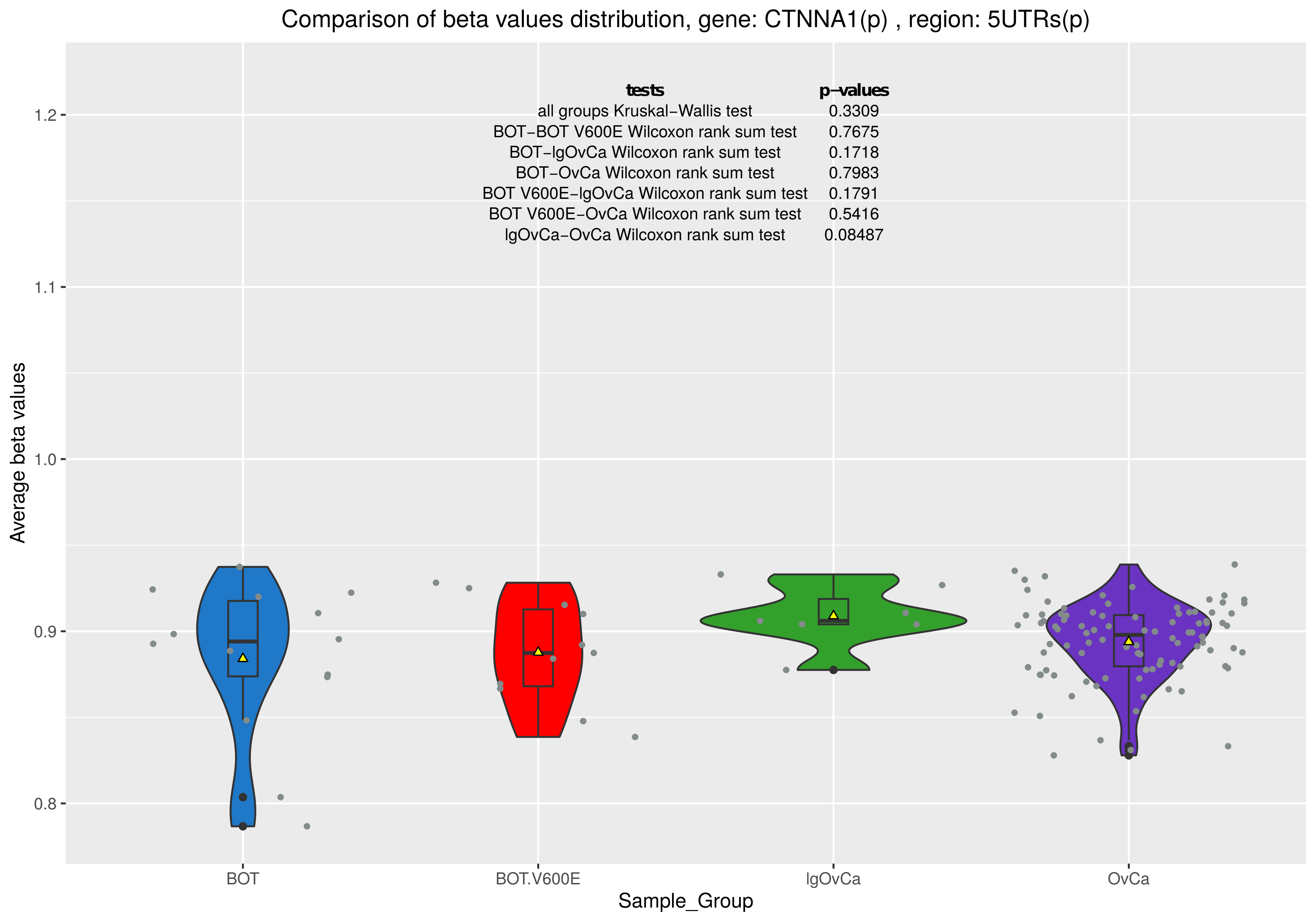
Comparison of beta values distribution, gene: CTNNA1(p) , region: 5UTRs(p)
tests
p−values
all groups Kruskal−Wallis test
0.3309
BOT−BOT V600E Wilcoxon rank sum test
0.7675
BOT−IgOvCa Wilcoxon rank sum test
0.1718
BOT−OvCa Wilcoxon rank sum test
0.7983
BOT V600E−IgOvCa Wilcoxon rank sum test
0.1791
BOT V600E−OvCa Wilcoxon rank sum test
0.5416
IgOvCa−OvCa Wilcoxon rank sum test
0.08487
Average beta values
1.2
1.1
1.0
0.9
0.8
BOT
BOT.V600E
IgOvCa
OvCa
Sample_Group

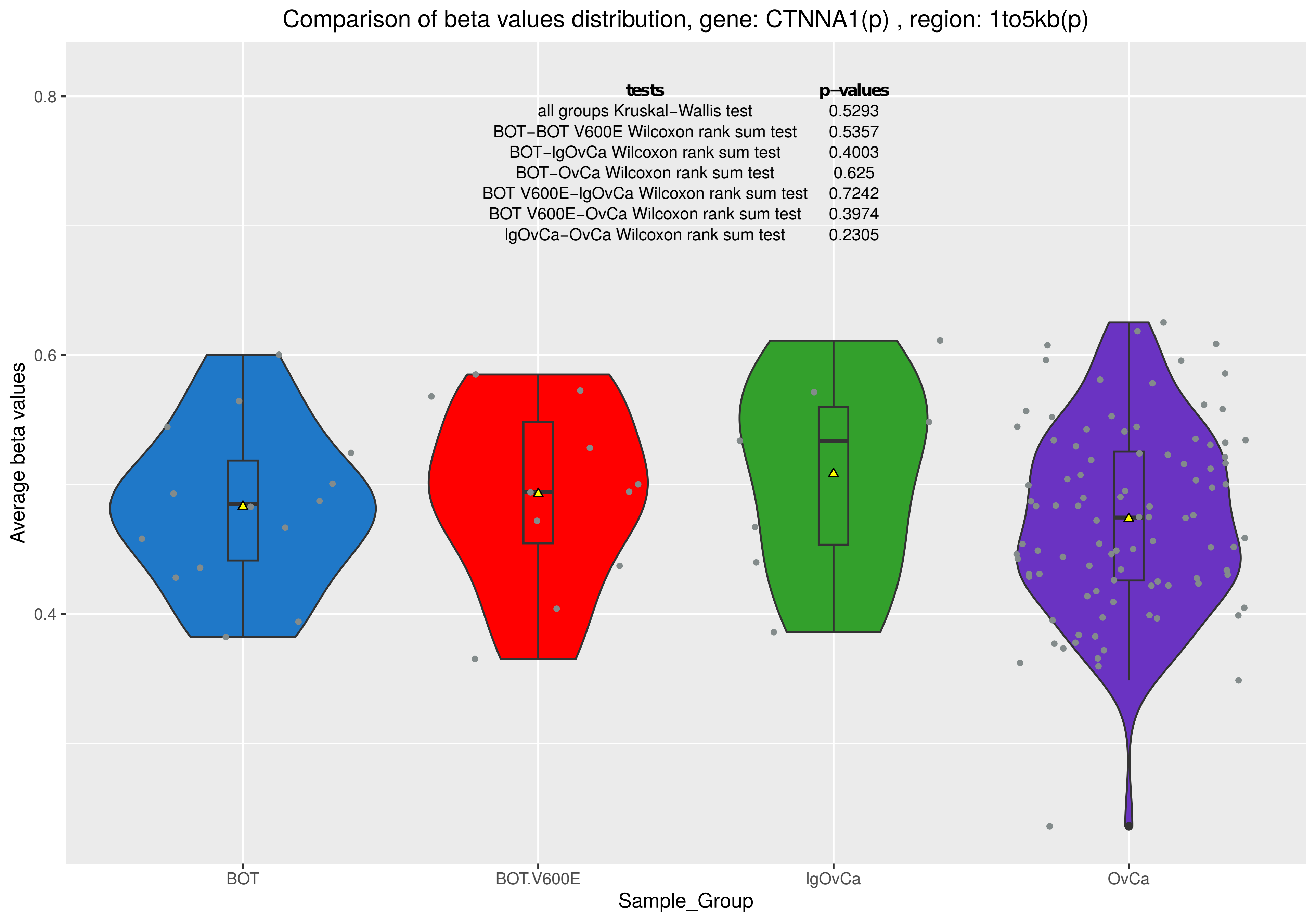

Comparison of beta values distribution, gene: CTNNA1(p) , region: 1to5kb(p)
tests
p−values
all groups Kruskal−Wallis test
0.5293
BOT−BOT V600E Wilcoxon rank sum test
0.5357
BOT−lgOvCa Wilcoxon rank sum test
0.4003
BOT−OvCa Wilcoxon rank sum test
0.625
BOT V600E−lgOvCa Wilcoxon rank sum test
0.7242
BOT V600E−OvCa Wilcoxon rank sum test
0.3974
lgOvCa−OvCa Wilcoxon rank sum test
0.2305
Average beta values
0.8
0.6
0.4
BOT
BOT.V600E
lgOvCa
OvCa
Sample_Group

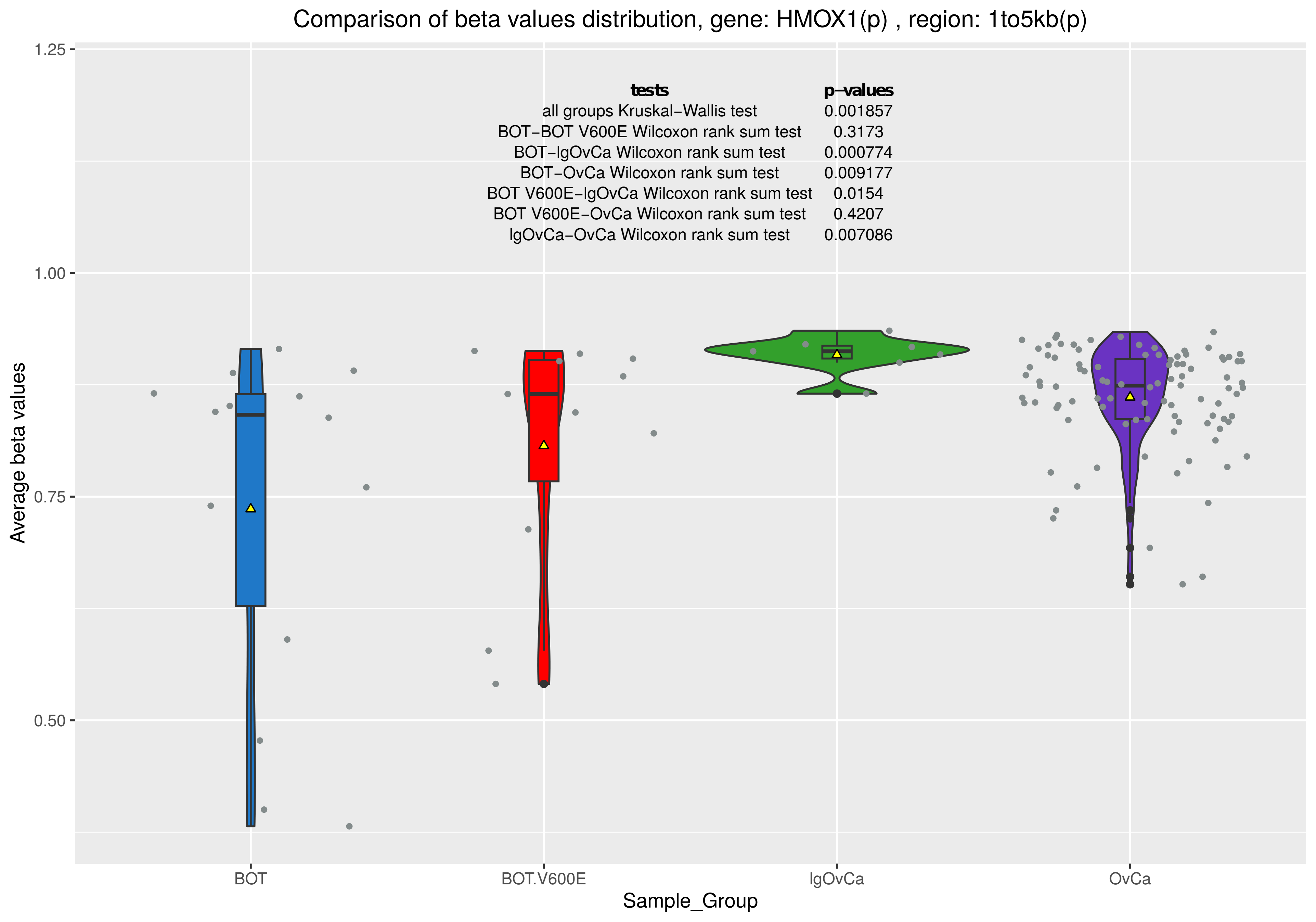

Comparison of beta values distribution, gene: HMOX1(p) , region: 1to5kb(p)
tests
p−values
all groups Kruskal−Wallis test
0.001857
BOT−BOT V600E Wilcoxon rank sum test
0.3173
BOT−IgOvCa Wilcoxon rank sum test
0.000774
BOT−OvCa Wilcoxon rank sum test
0.009177
BOT V600E−IgOvCa Wilcoxon rank sum test
0.0154
BOT V600E−OvCa Wilcoxon rank sum test
0.4207
IgOvCa−OvCa Wilcoxon rank sum test
0.007086
Average beta values
1.25
1.00
0.75
0.50
BOT
BOT.V600E
IgOvCa
OvCa
Sample_Group

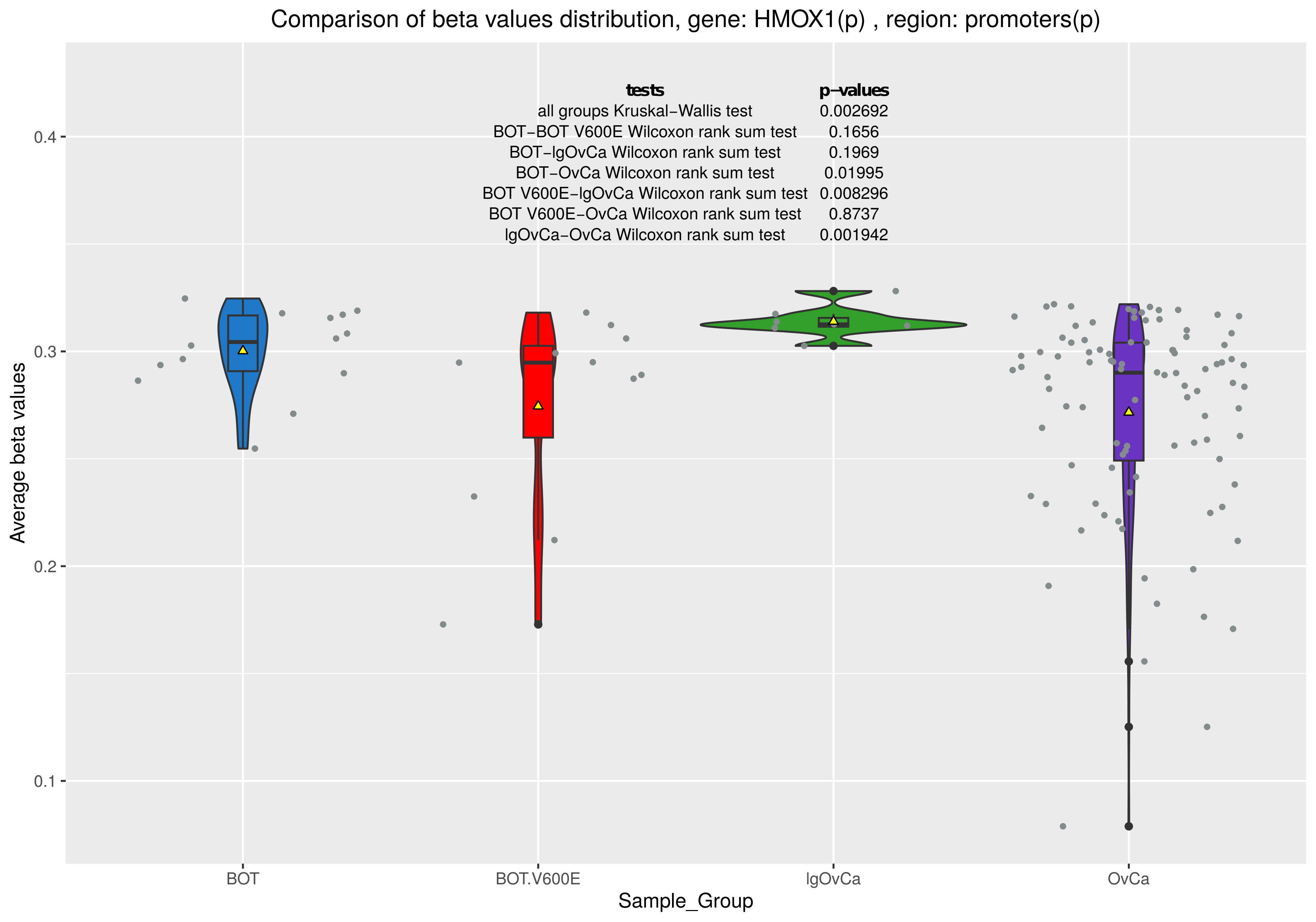

Comparison of beta values distribution, gene: HMOX1(p) , region: promoters(p)
tests
p−values
all groups Kruskal−Wallis test
0.002692
BOT−BOT V600E Wilcoxon rank sum test
0.1656
BOT−lgOvCa Wilcoxon rank sum test
0.1969
BOT−OvCa Wilcoxon rank sum test
0.01995
BOT V600E−lgOvCa Wilcoxon rank sum test
0.008296
BOT V600E−OvCa Wilcoxon rank sum test
0.8737
lgOvCa−OvCa Wilcoxon rank sum test
0.001942
Average beta values
0.4
0.3
0.2
0.1
BOT
BOT.V600E
lgOvCa
OvCa
Sample_Group

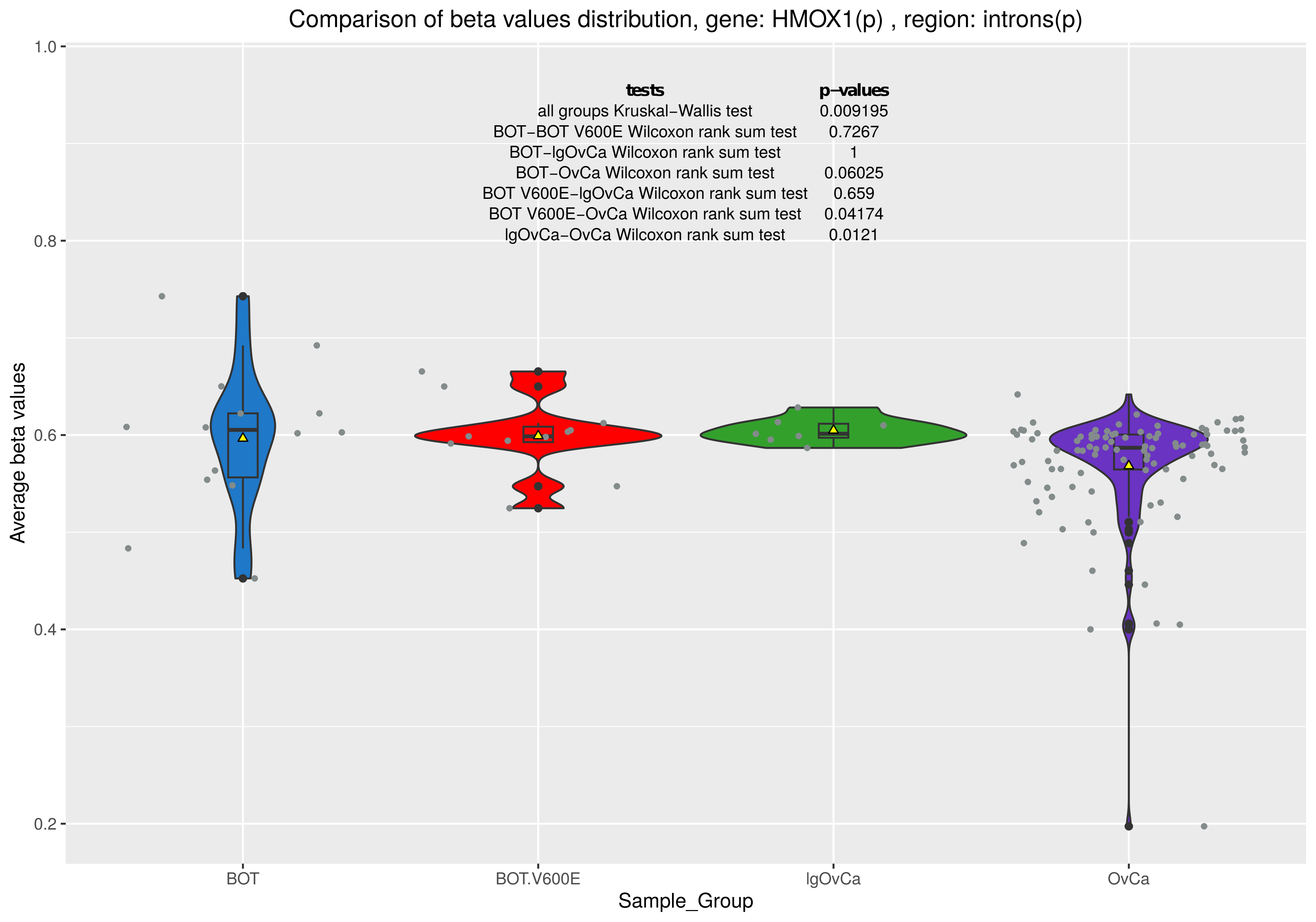
Comparison of beta values distribution, gene: HMOX1(p) , region: introns(p)
tests
p−values
all groups Kruskal−Wallis test
0.009195
BOT−BOT V600E Wilcoxon rank sum test
0.7267
BOT−lgOvCa Wilcoxon rank sum test
1
BOT−OvCa Wilcoxon rank sum test
0.06025
BOT V600E−lgOvCa Wilcoxon rank sum test
0.659
BOT V600E−OvCa Wilcoxon rank sum test
0.04174
lgOvCa−OvCa Wilcoxon rank sum test
0.0121
Average beta values
1.0
0.8
0.6
0.4
0.2
BOT
BOT.V600E
lgOvCa
OvCa
Sample_Group

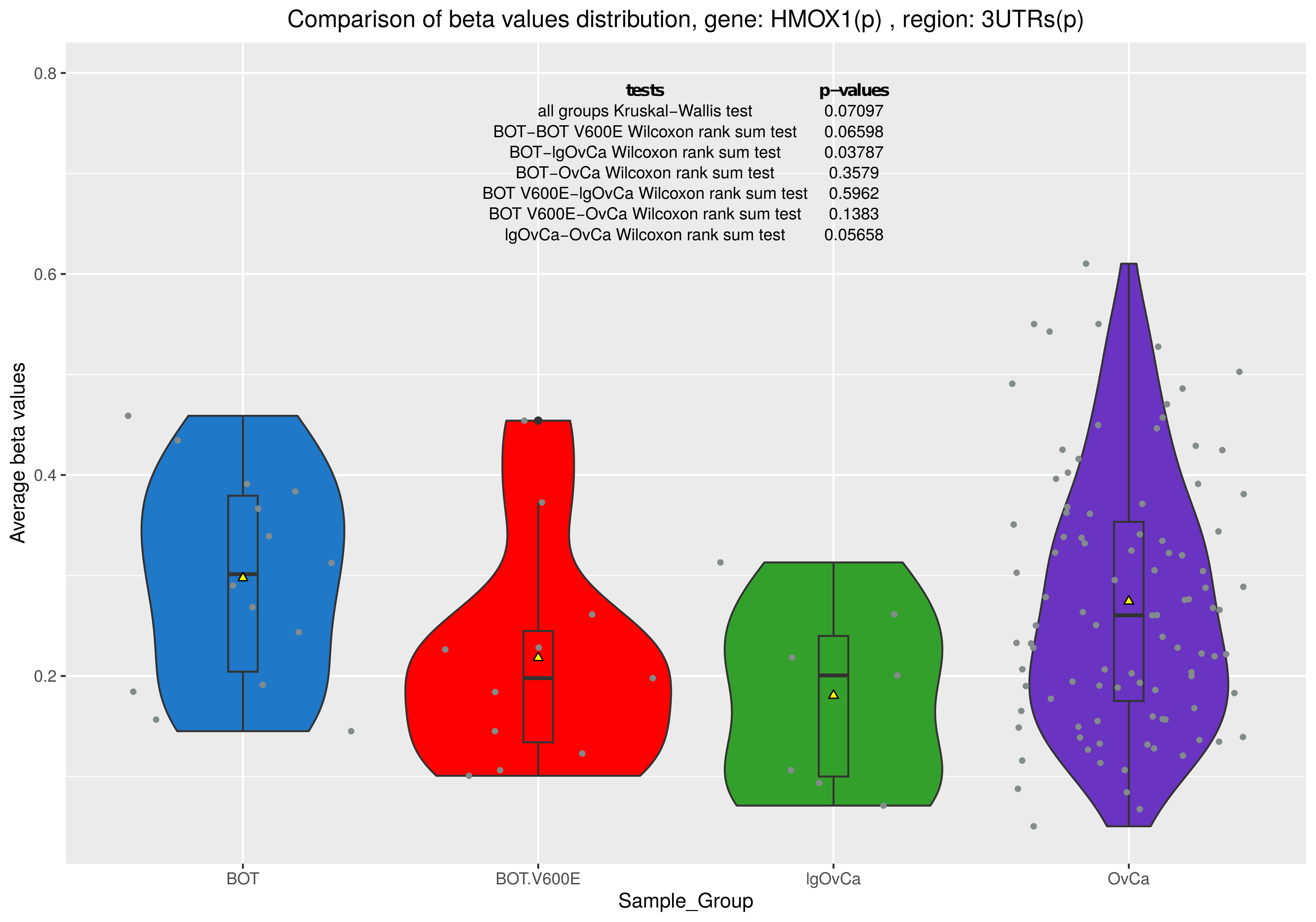

Comparison of beta values distribution, gene: HMOX1(p) , region: 3UTRs(p)
tests
p−values
all groups Kruskal−Wallis test
0.07097
BOT−BOT V600E Wilcoxon rank sum test
0.06598
BOT−IgOvCa Wilcoxon rank sum test
0.03787
BOT−OvCa Wilcoxon rank sum test
0.3579
BOT V600E−IgOvCa Wilcoxon rank sum test
0.5962
BOT V600E−OvCa Wilcoxon rank sum test
0.1383
IgOvCa−OvCa Wilcoxon rank sum test
0.05658
Average beta values
0.8
0.6
0.4
0.2
BOT
BOT.V600E
IgOvCa
OvCa
Sample_Group

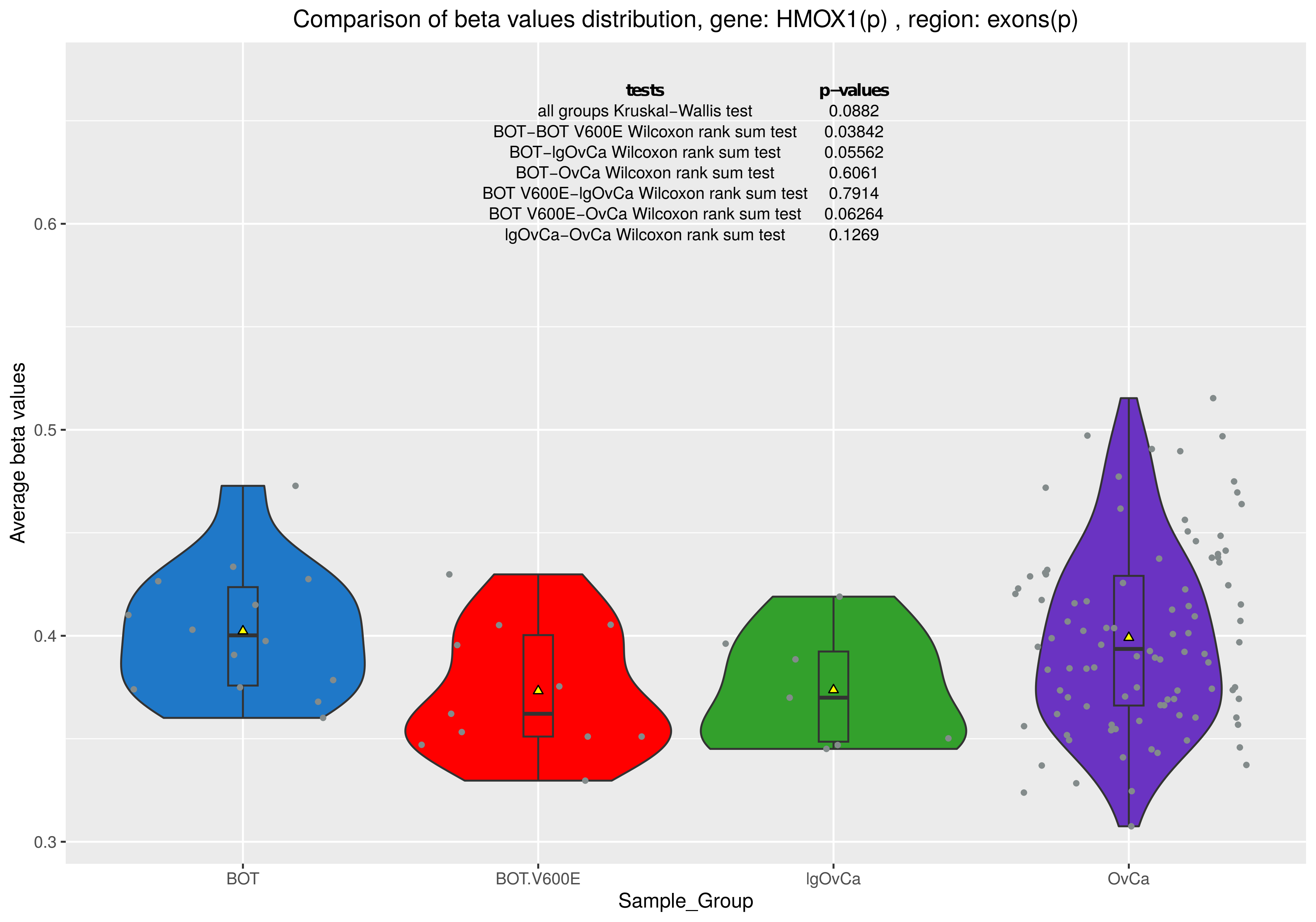
Comparison of beta values distribution, gene: HMOX1(p) , region: exons(p)
tests
p−values
all groups Kruskal−Wallis test
0.0882
BOT−BOT V600E Wilcoxon rank sum test
0.03842
BOT−lgOvCa Wilcoxon rank sum test
0.05562
BOT−OvCa Wilcoxon rank sum test
0.6061
BOT V600E−lgOvCa Wilcoxon rank sum test
0.7914
BOT V600E−OvCa Wilcoxon rank sum test
0.06264
lgOvCa−OvCa Wilcoxon rank sum test
0.1269
Average beta values
0.6
0.5
0.4
0.3
BOT
BOT.V600E
lgOvCa
OvCa
Sample_Group

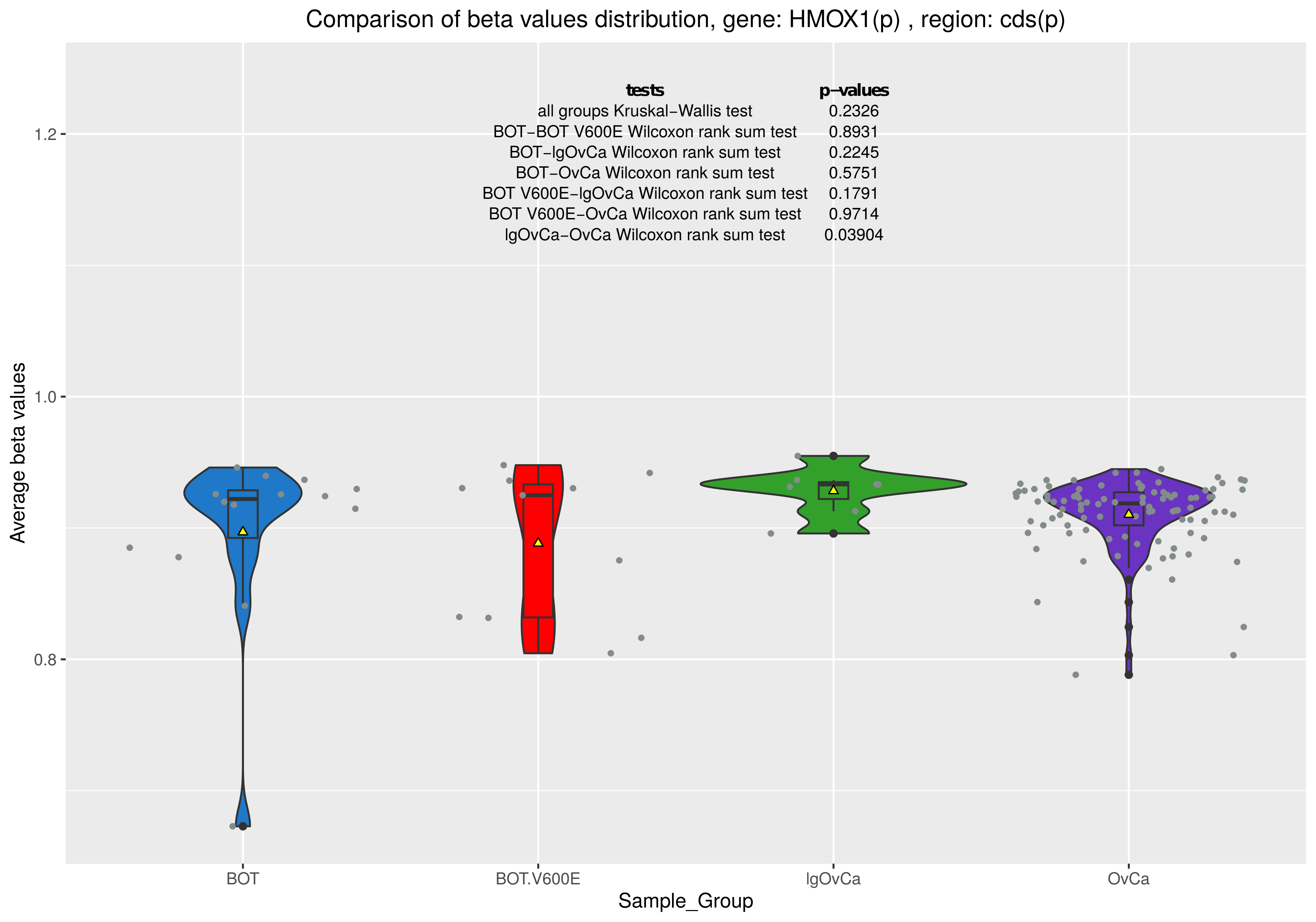
Comparison of beta values distribution, gene: HMOX1(p) , region: cds(p)
tests
p−values
all groups Kruskal−Wallis test
0.2326
BOT−BOT V600E Wilcoxon rank sum test
0.8931
BOT−IgOvCa Wilcoxon rank sum test
0.2245
BOT−OvCa Wilcoxon rank sum test
0.5751
BOT V600E−IgOvCa Wilcoxon rank sum test
0.1791
BOT V600E−OvCa Wilcoxon rank sum test
0.9714
IgOvCa−OvCa Wilcoxon rank sum test
0.03904
Average beta values
1.2
1.0
0.8
BOT
BOT.V600E
IgOvCa
OvCa
Sample_Group

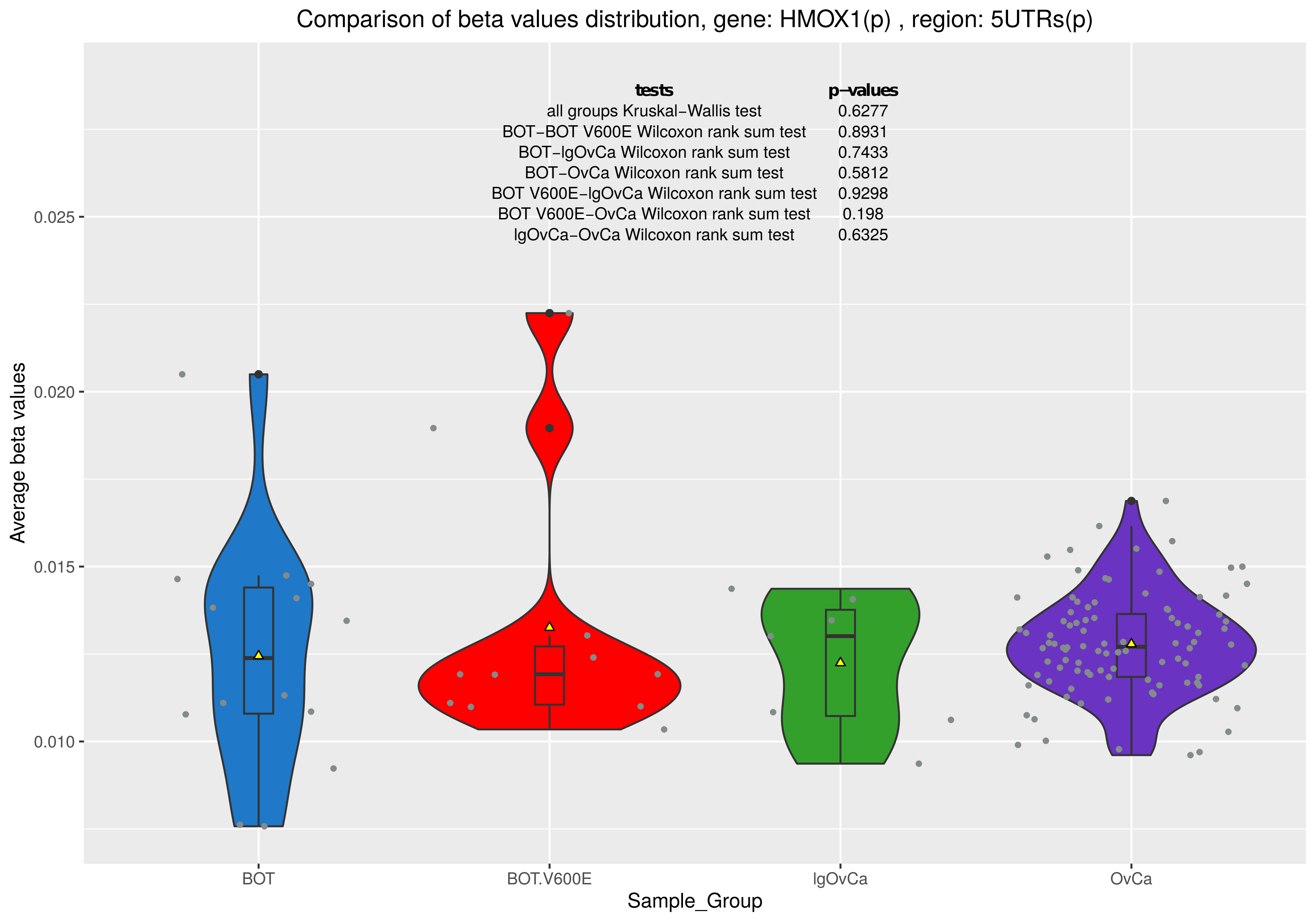

Comparison of beta values distribution, gene: HMOX1(p) , region: 5UTRs(p)
tests
p−values
all groups Kruskal−Wallis test
0.6277
BOT−BOT V600E Wilcoxon rank sum test
0.8931
BOT−lgOvCa Wilcoxon rank sum test
0.7433
BOT−OvCa Wilcoxon rank sum test
0.5812
BOT V600E−lgOvCa Wilcoxon rank sum test
0.9298
BOT V600E−OvCa Wilcoxon rank sum test
0.198
lgOvCa−OvCa Wilcoxon rank sum test
0.6325
Average beta values
0.025
0.020
0.015
0.010
BOT
BOT.V600E
lgOvCa
OvCa
Sample_Group

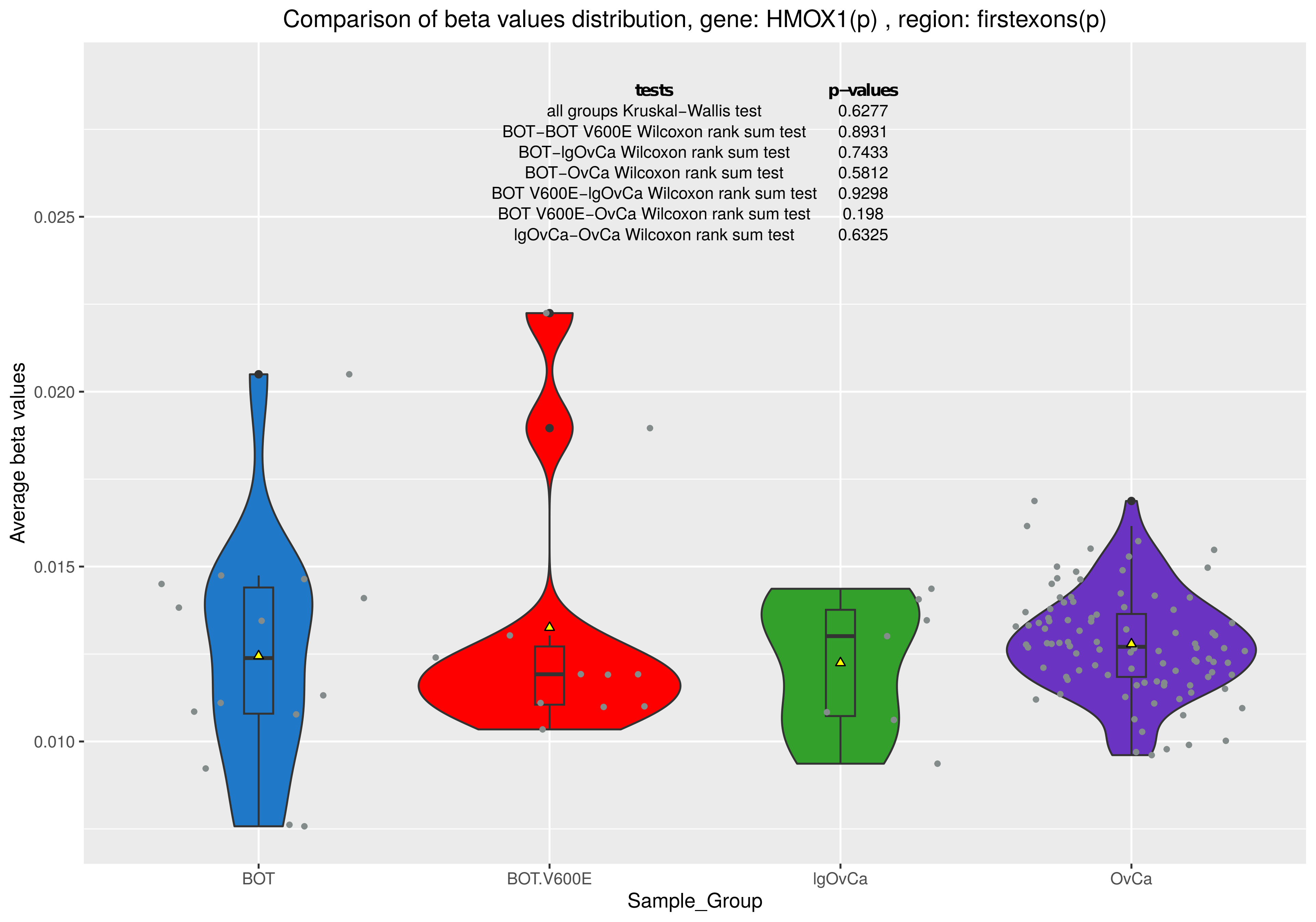
Comparison of beta values distribution, gene: HMOX1(p) , region: firstexons(p)
tests
p−values
all groups Kruskal−Wallis test
0.6277
BOT−BOT V600E Wilcoxon rank sum test
0.8931
BOT−IgOvCa Wilcoxon rank sum test
0.7433
BOT−OvCa Wilcoxon rank sum test
0.5812
BOT V600E−IgOvCa Wilcoxon rank sum test
0.9298
BOT V600E−OvCa Wilcoxon rank sum test
0.198
IgOvCa−OvCa Wilcoxon rank sum test
0.6325
Average beta values
0.025
0.020
0.015
0.010
BOT
BOT.V600E
IgOvCa
OvCa
Sample_Group

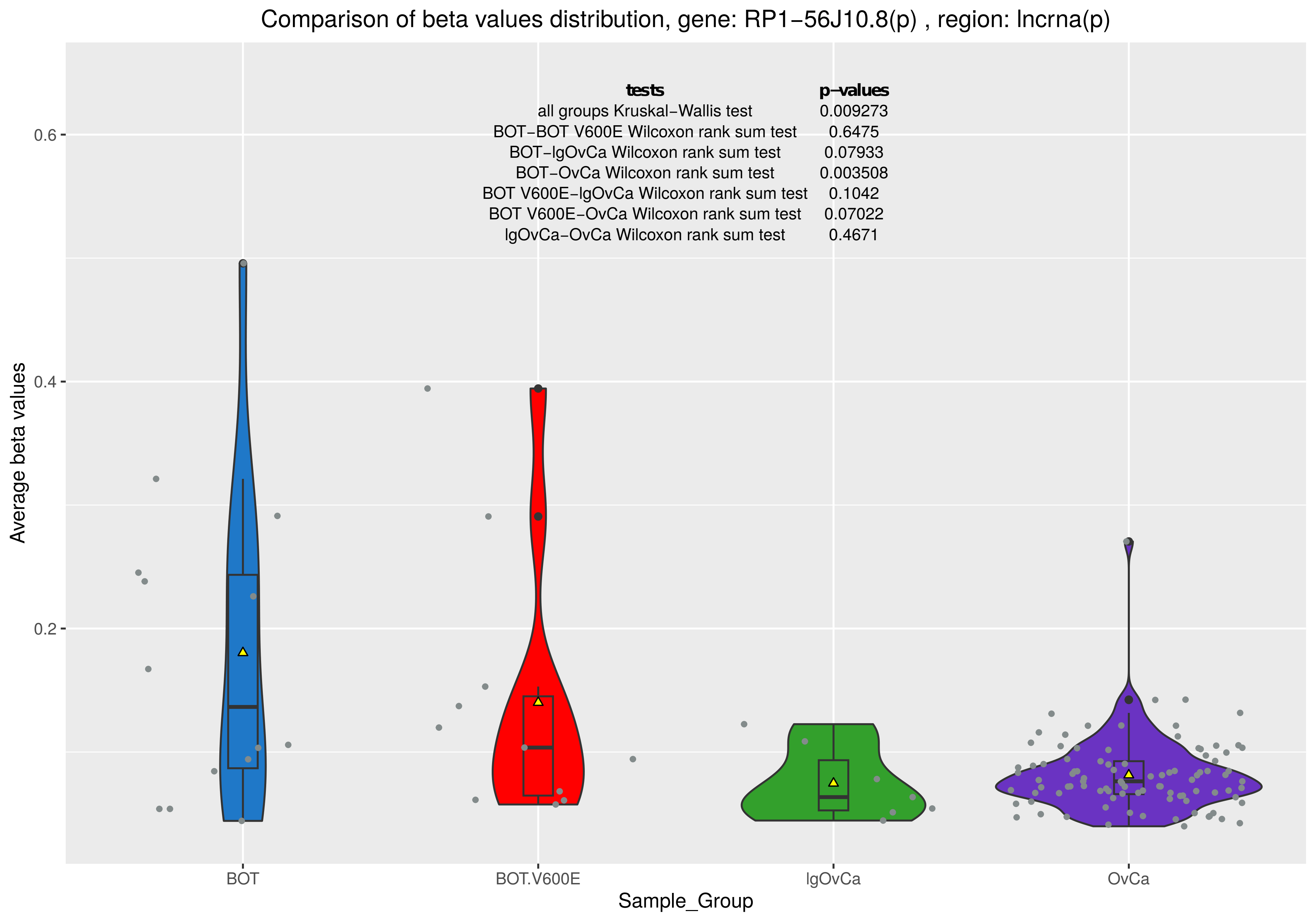

Comparison of beta values distribution, gene: RP1−56J10.8(p) , region: lncrna(p)
tests
p−values
all groups Kruskal−Wallis test
0.009273
BOT−BOT V600E Wilcoxon rank sum test
0.6475
BOT−lgOvCa Wilcoxon rank sum test
0.07933
BOT−OvCa Wilcoxon rank sum test
0.003508
BOT V600E−lgOvCa Wilcoxon rank sum test
0.1042
BOT V600E−OvCa Wilcoxon rank sum test
0.07022
lgOvCa−OvCa Wilcoxon rank sum test
0.4671
Average beta values
0.6
0.4
0.2
BOT
BOT.V600E
lgOvCa
OvCa
Sample_Group

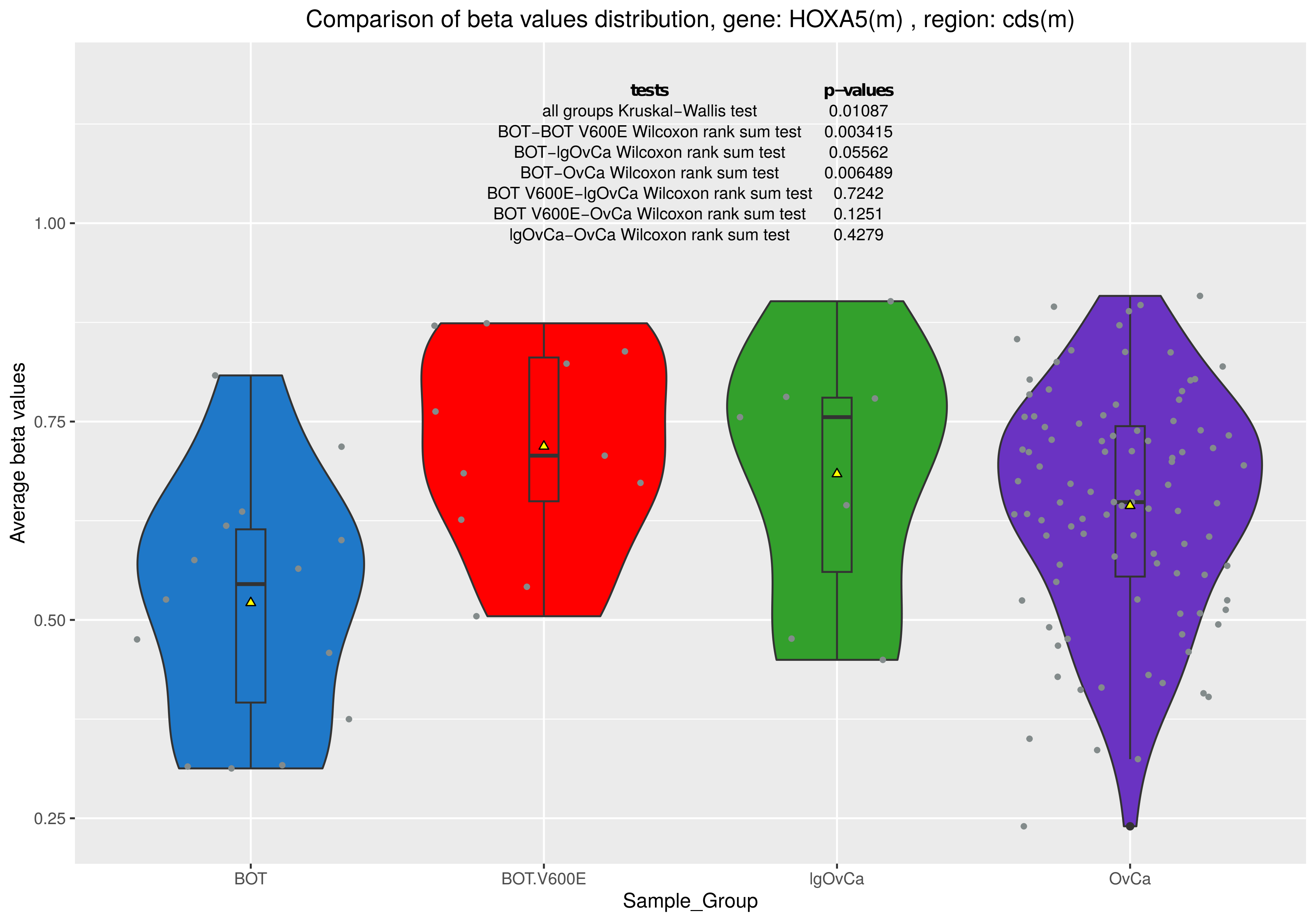
Comparison of beta values distribution, gene: HOXA5(m) , region: cds(m)
tests
p−values
all groups Kruskal−Wallis test
0.01087
BOT−BOT V600E Wilcoxon rank sum test
0.003415
BOT−IgOvCa Wilcoxon rank sum test
0.05562
BOT−OvCa Wilcoxon rank sum test
0.006489
BOT V600E−IgOvCa Wilcoxon rank sum test
0.7242
BOT V600E−OvCa Wilcoxon rank sum test
0.1251
IgOvCa−OvCa Wilcoxon rank sum test
0.4279
Average beta values
1.00
0.75
0.50
0.25
BOT
BOT.V600E
IgOvCa
OvCa
Sample_Group

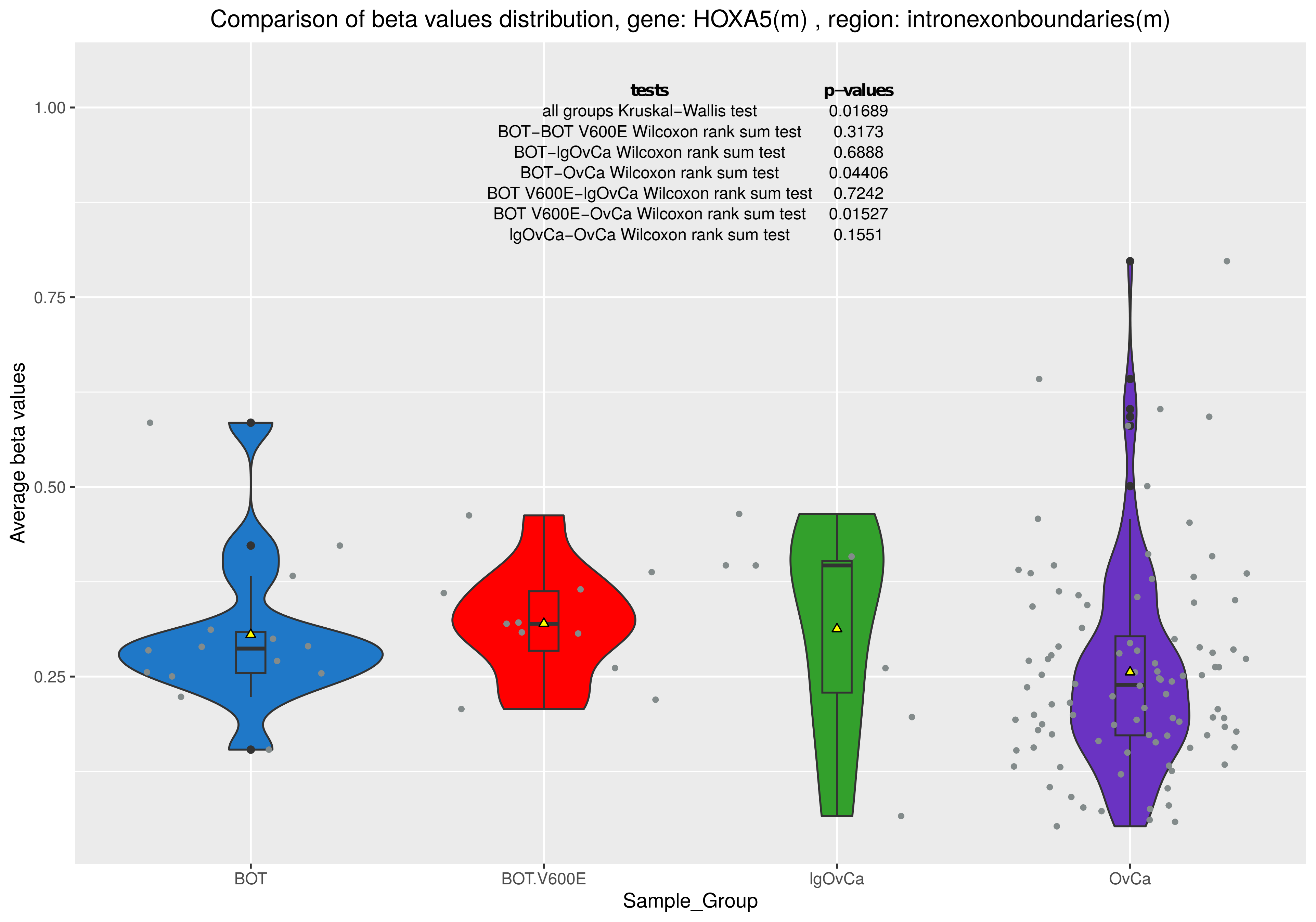
Comparison of beta values distribution, gene: HOXA5(m) , region: intronexonboundaries(m)
tests
p−values
all groups Kruskal−Wallis test
0.01689
BOT−BOT V600E Wilcoxon rank sum test
0.3173
BOT−lgOvCa Wilcoxon rank sum test
0.6888
BOT−OvCa Wilcoxon rank sum test
0.04406
BOT V600E−lgOvCa Wilcoxon rank sum test
0.7242
BOT V600E−OvCa Wilcoxon rank sum test
0.01527
lgOvCa−OvCa Wilcoxon rank sum test
0.1551
Average beta values
1.00
0.75
0.50
0.25
BOT
BOT.V600E
lgOvCa
OvCa
Sample_Group

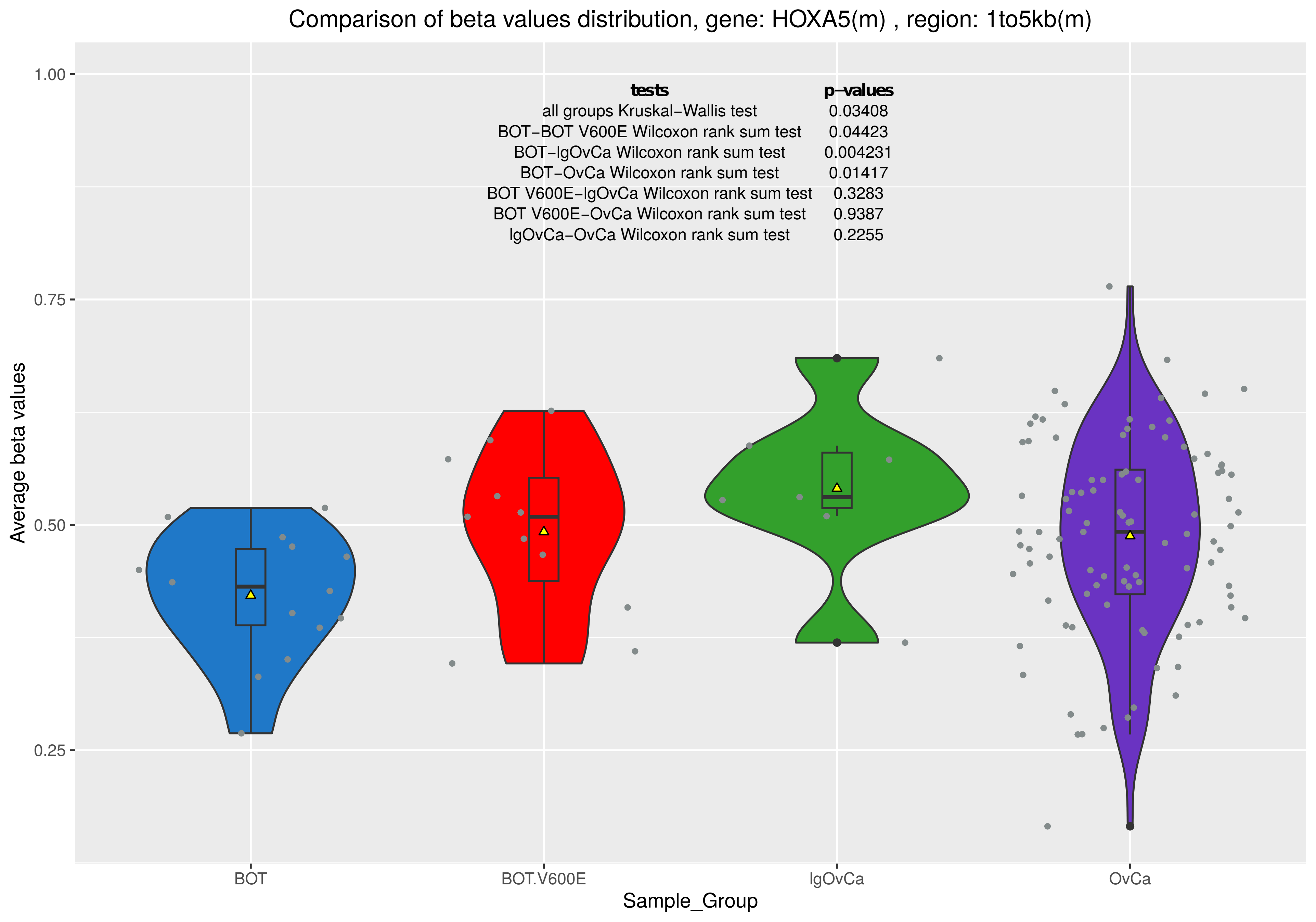

Comparison of beta values distribution, gene: HOXA5(m) , region: 1to5kb(m)
tests
p−values
all groups Kruskal−Wallis test 0.03408
BOT−BOT V600E Wilcoxon rank sum test 0.04423
BOT−lgOvCa Wilcoxon rank sum test 0.004231
BOT−OvCa Wilcoxon rank sum test 0.01417
BOT V600E−lgOvCa Wilcoxon rank sum test 0.3283
BOT V600E−OvCa Wilcoxon rank sum test 0.9387
lgOvCa−OvCa Wilcoxon rank sum test 0.2255
Average beta values
1.00
0.75
0.50
0.25
BOT
BOT.V600E
lgOvCa
OvCa
Sample_Group

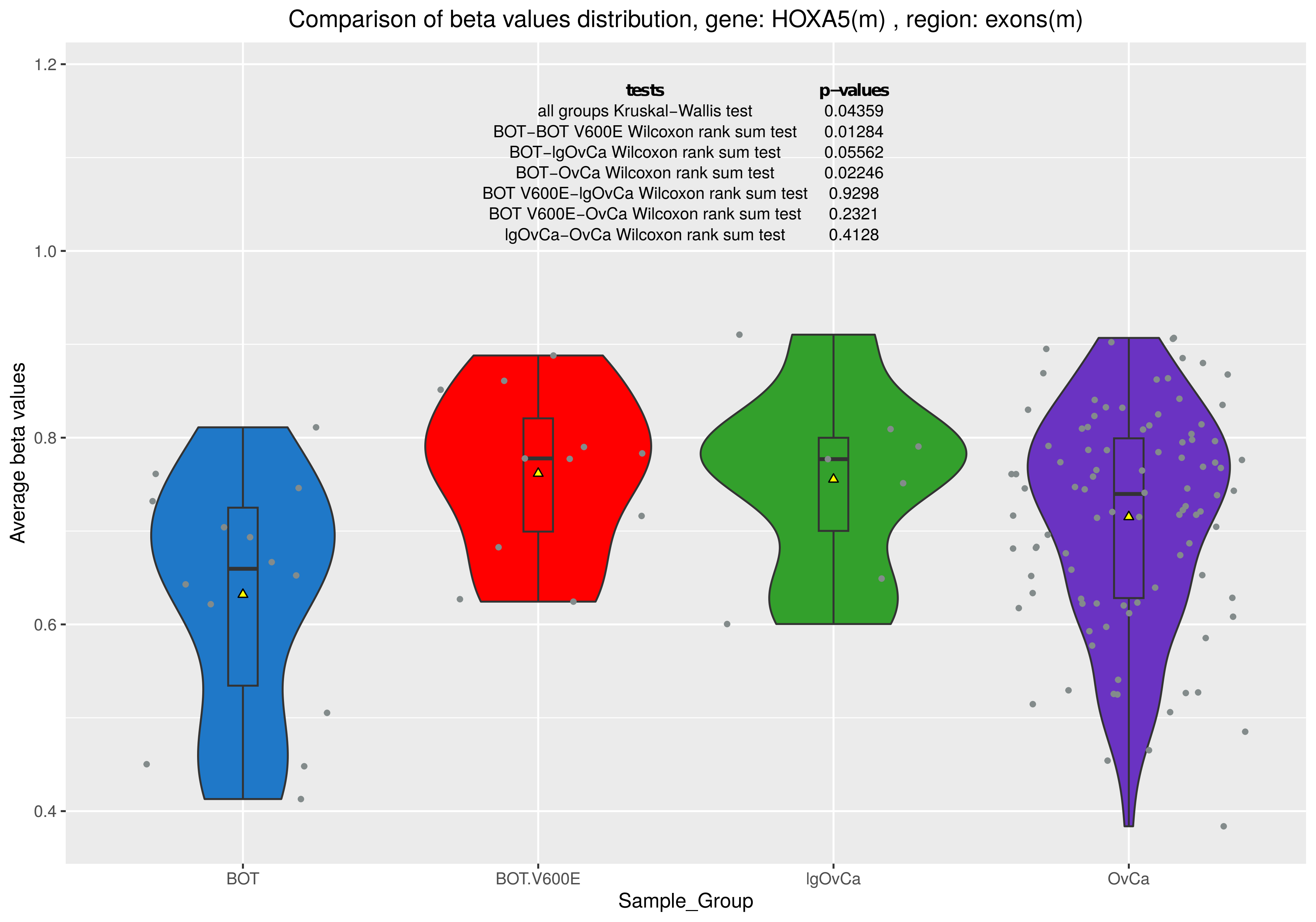
Comparison of beta values distribution, gene: HOXA5(m) , region: exons(m)
tests
p−values
all groups Kruskal−Wallis test 0.04359
BOT−BOT V600E Wilcoxon rank sum test 0.01284
BOT−IgOvCa Wilcoxon rank sum test 0.05562
BOT−OvCa Wilcoxon rank sum test 0.02246
BOT V600E−IgOvCa Wilcoxon rank sum test 0.9298
BOT V600E−OvCa Wilcoxon rank sum test 0.2321
IgOvCa−OvCa Wilcoxon rank sum test 0.4128
Average beta values
1.2
1.0
0.8
0.6
0.4
BOT
BOT.V600E
IgOvCa
OvCa
Sample_Group

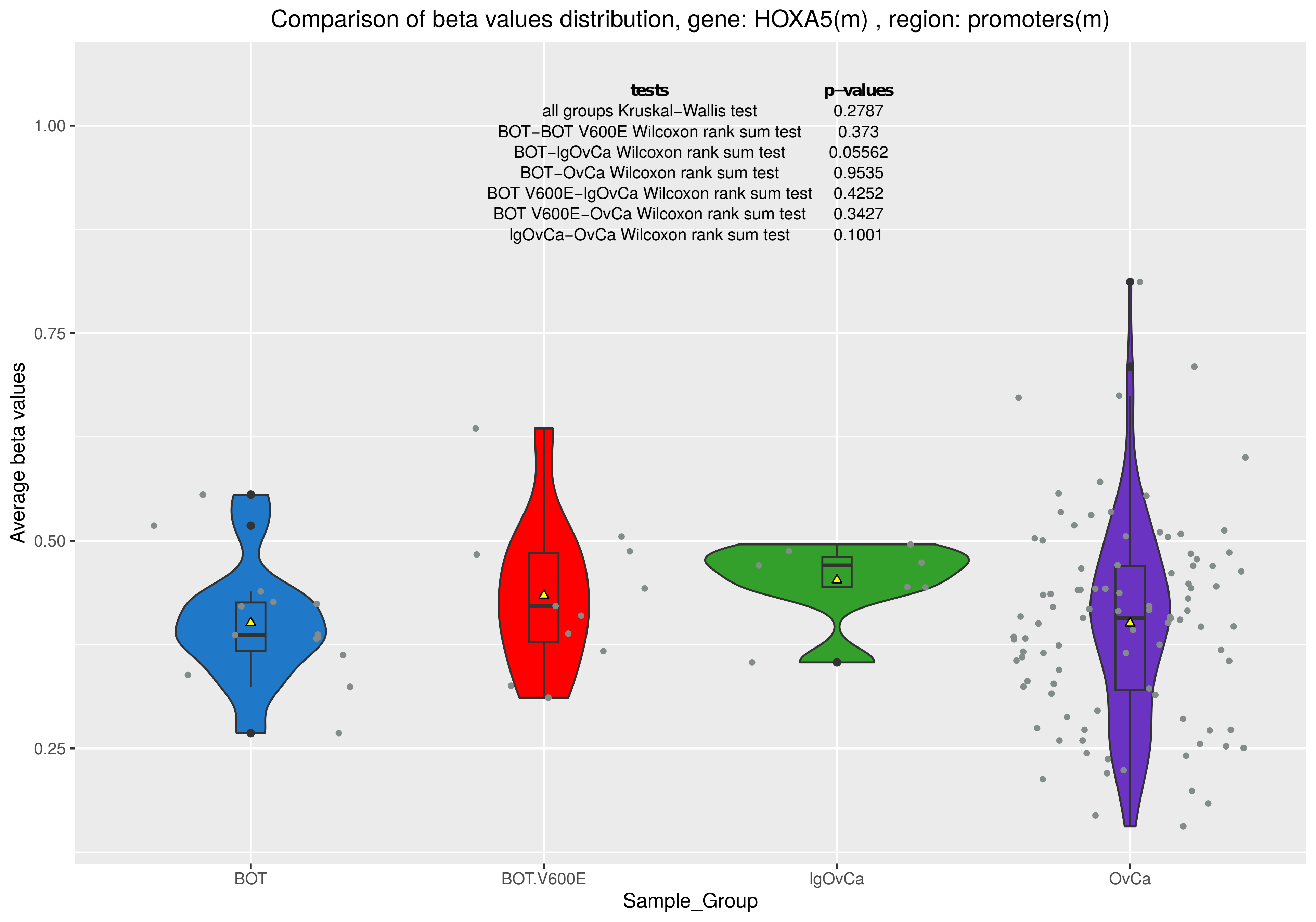
Comparison of beta values distribution, gene: HOXA5(m) , region: promoters(m)
tests
p−values
all groups Kruskal−Wallis test
0.2787
BOT−BOT V600E Wilcoxon rank sum test
0.373
BOT−lgOvCa Wilcoxon rank sum test
0.05562
BOT−OvCa Wilcoxon rank sum test
0.9535
BOT V600E−lgOvCa Wilcoxon rank sum test
0.4252
BOT V600E−OvCa Wilcoxon rank sum test
0.3427
lgOvCa−OvCa Wilcoxon rank sum test
0.1001
Average beta values
1.00
0.75
0.50
0.25
BOT
BOT.V600E
lgOvCa
OvCa
Sample_Group

Comparison of beta values distribution, gene: HOXA5(m) , region: introns(m)

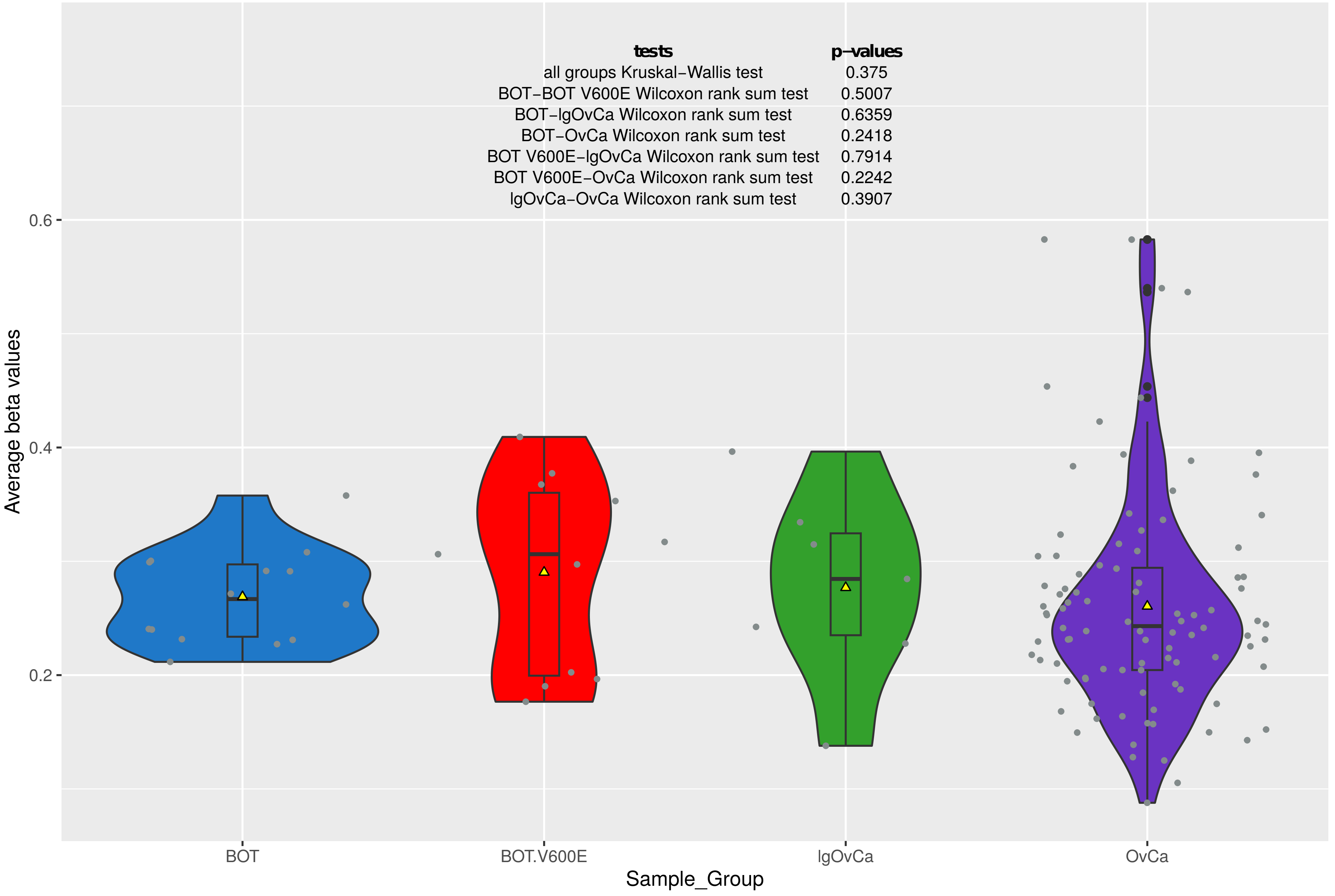

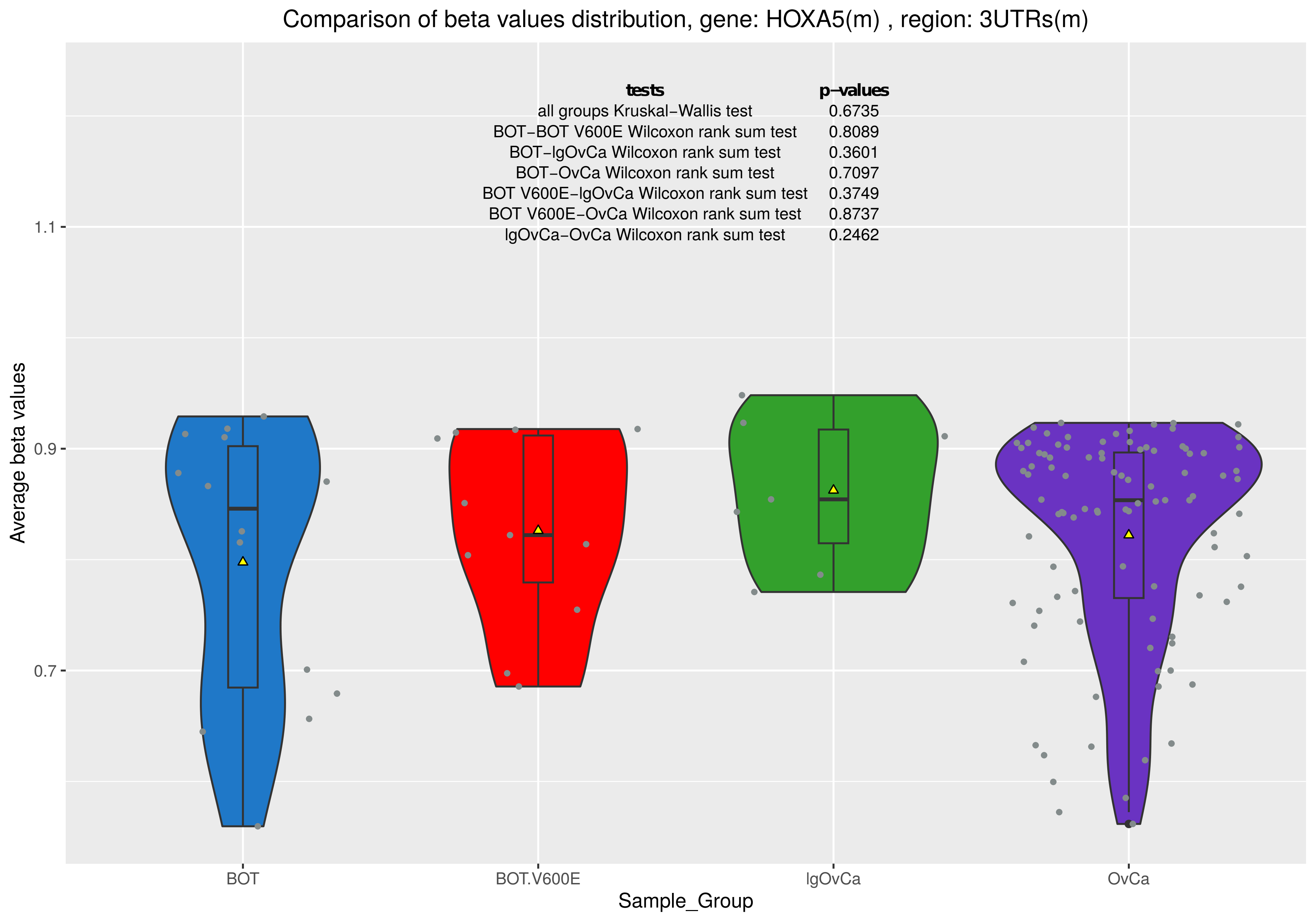
Comparison of beta values distribution, gene: HOXA5(m) , region: 3UTRs(m)
tests
p−values
all groups Kruskal−Wallis test 0.6735
BOT−BOT V600E Wilcoxon rank sum test 0.8089
BOT−lgOvCa Wilcoxon rank sum test 0.3601
BOT−OvCa Wilcoxon rank sum test 0.7097
BOT V600E−lgOvCa Wilcoxon rank sum test 0.3749
BOT V600E−OvCa Wilcoxon rank sum test 0.8737
lgOvCa−OvCa Wilcoxon rank sum test 0.2462
Average beta values
1.1
0.9
0.7
BOT
BOT.V600E
lgOvCa
OvCa
Sample_Group

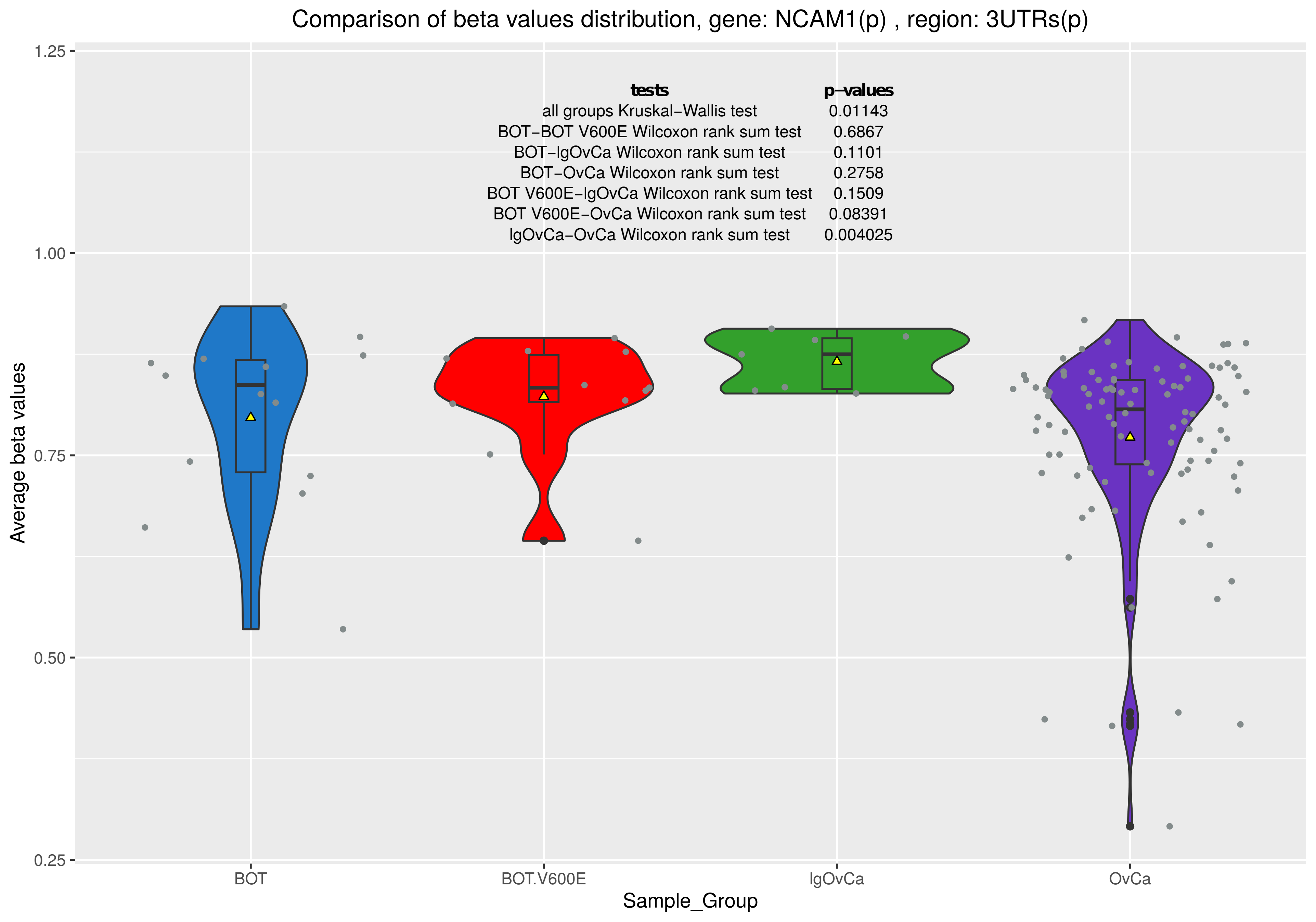

Comparison of beta values distribution, gene: NCAM1(p) , region: 3UTRs(p)
tests
p−values
all groups Kruskal−Wallis test
0.01143
BOT−BOT V600E Wilcoxon rank sum test
0.6867
BOT−IgOvCa Wilcoxon rank sum test
0.1101
BOT−OvCa Wilcoxon rank sum test
0.2758
BOT V600E−IgOvCa Wilcoxon rank sum test
0.1509
BOT V600E−OvCa Wilcoxon rank sum test
0.08391
IgOvCa−OvCa Wilcoxon rank sum test
0.004025
Average beta values
1.25
1.00
0.75
0.50
0.25
BOT
BOT.V600E
IgOvCa
OvCa
Sample_Group

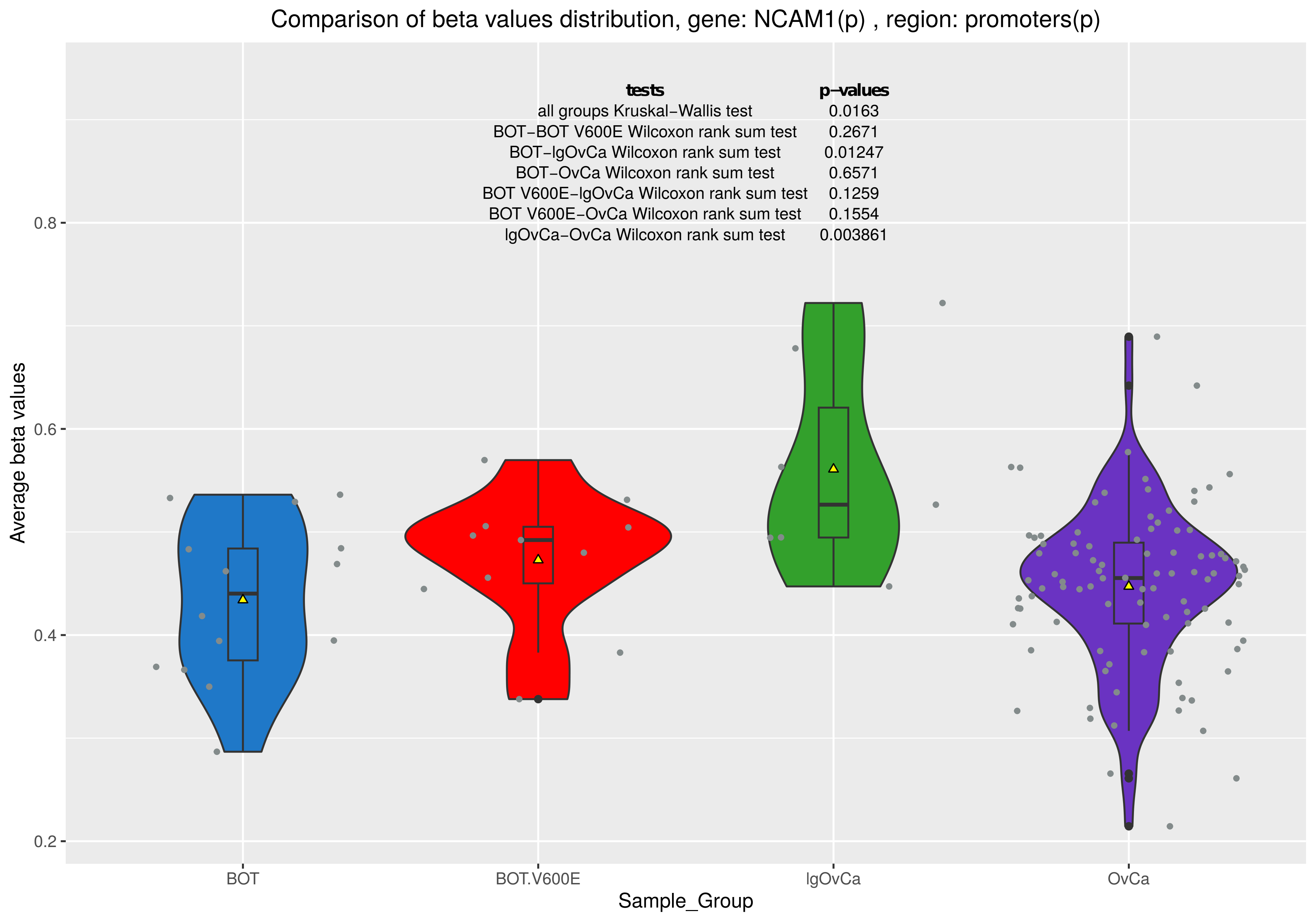
Comparison of beta values distribution, gene: NCAM1(p) , region: promoters(p)
tests
p−values
all groups Kruskal−Wallis test
0.0163
BOT−BOT V600E Wilcoxon rank sum test
0.2671
BOT−lgOvCa Wilcoxon rank sum test
0.01247
BOT−OvCa Wilcoxon rank sum test
0.6571
BOT V600E−lgOvCa Wilcoxon rank sum test
0.1259
BOT V600E−OvCa Wilcoxon rank sum test
0.1554
lgOvCa−OvCa Wilcoxon rank sum test
0.003861
Average beta values
0.8
0.6
0.4
0.2
BOT
BOT.V600E
lgOvCa
OvCa
Sample_Group

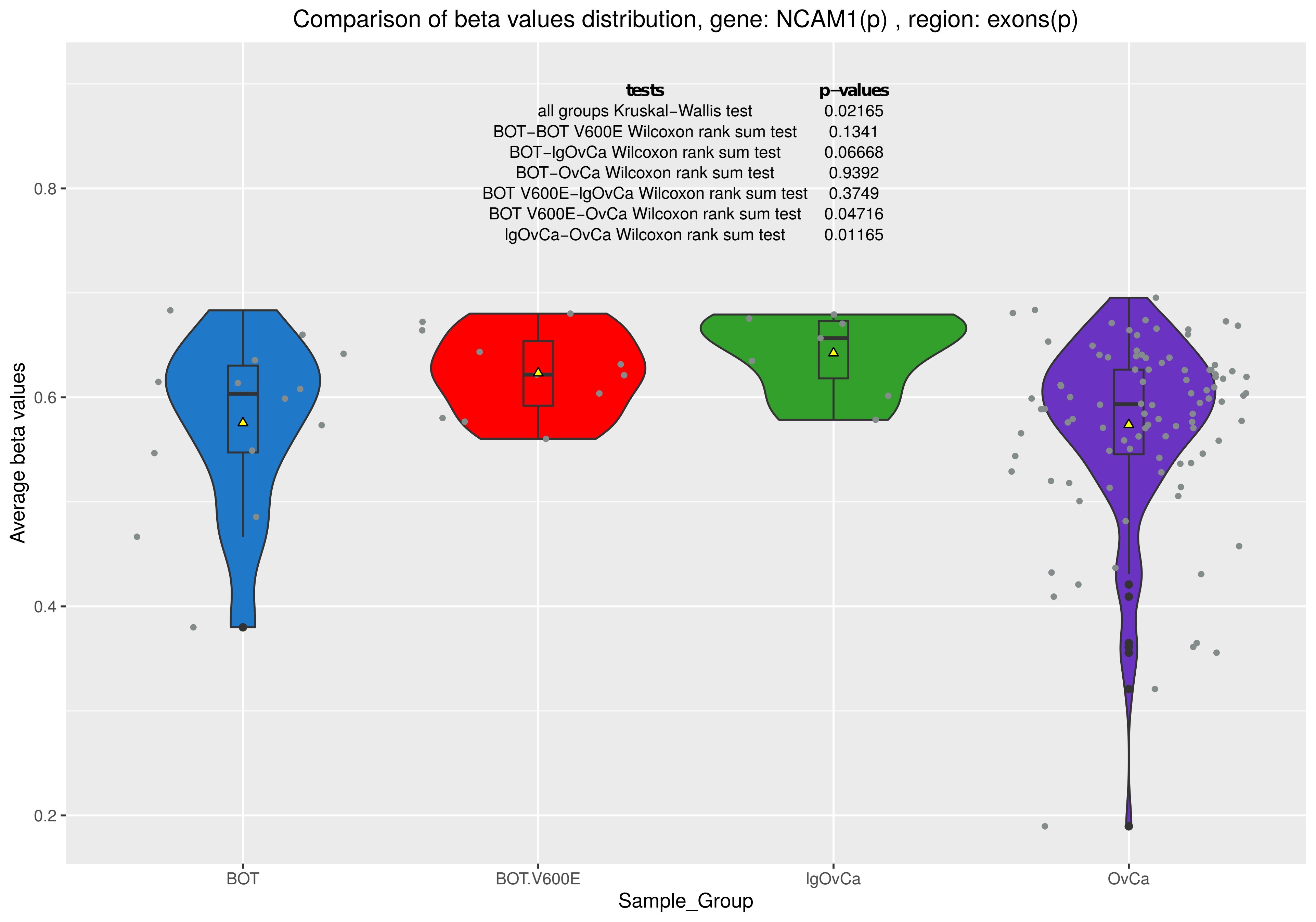
Comparison of beta values distribution, gene: NCAM1(p) , region: exons(p)
tests
p−values
all groups Kruskal−Wallis test
0.02165
BOT−BOT V600E Wilcoxon rank sum test
0.1341
BOT−lgOvCa Wilcoxon rank sum test
0.06668
BOT−OvCa Wilcoxon rank sum test
0.9392
BOT V600E−lgOvCa Wilcoxon rank sum test
0.3749
BOT V600E−OvCa Wilcoxon rank sum test
0.04716
lgOvCa−OvCa Wilcoxon rank sum test
0.01165
Average beta values
0.8
0.6
0.4
0.2
BOT
BOT.V600E
lgOvCa
OvCa
Sample_Group

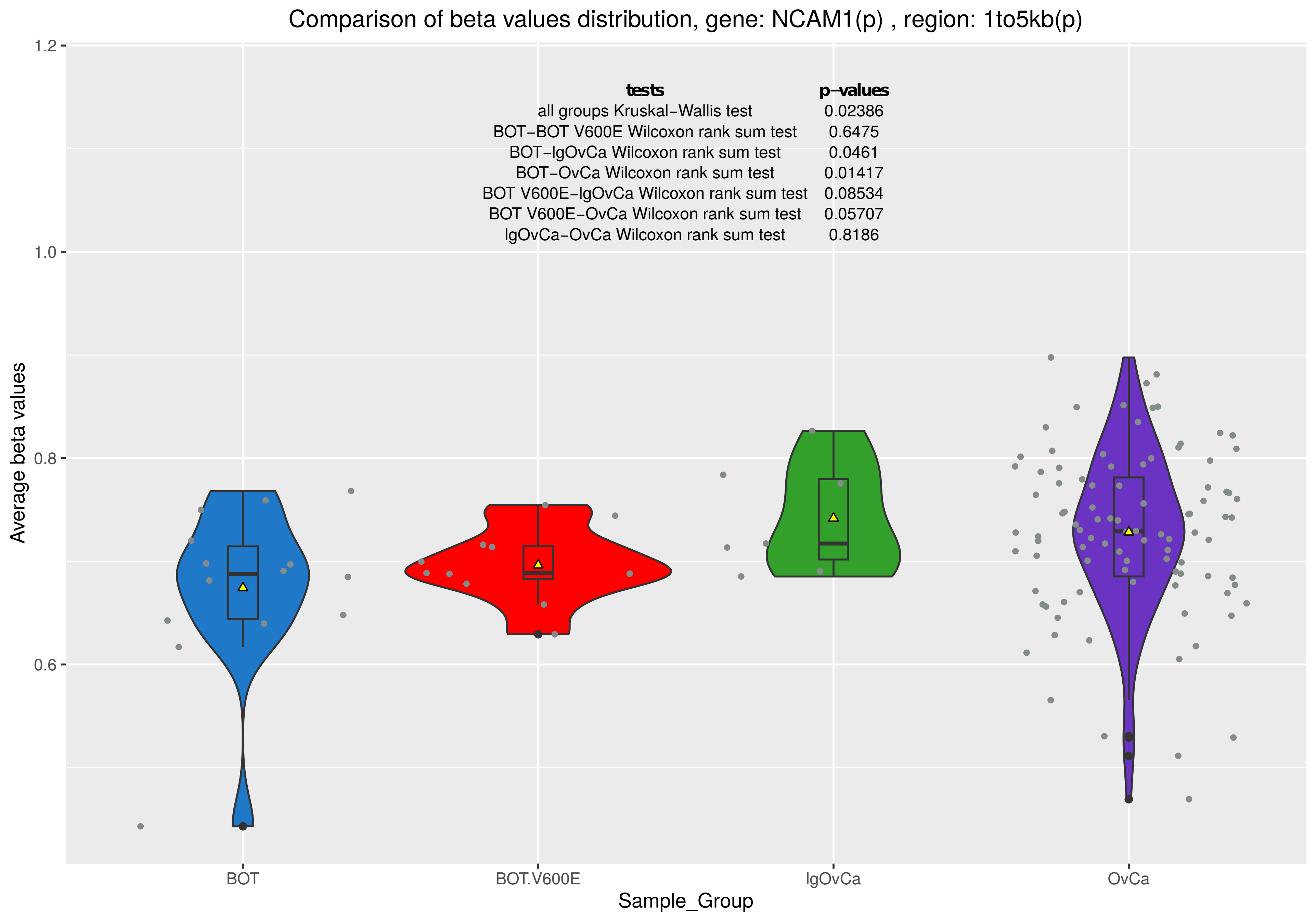

Comparison of beta values distribution, gene: NCAM1(p) , region: 1to5kb(p)
tests
p−values
all groups Kruskal−Wallis test
0.02386
BOT−BOT V600E Wilcoxon rank sum test
0.6475
BOT−IgOvCa Wilcoxon rank sum test
0.0461
BOT−OvCa Wilcoxon rank sum test
0.01417
BOT V600E−IgOvCa Wilcoxon rank sum test
0.08534
BOT V600E−OvCa Wilcoxon rank sum test
0.05707
IgOvCa−OvCa Wilcoxon rank sum test
0.8186
Average beta values
1.2
1.0
0.8
0.6
BOT
BOT.V600E
IgOvCa
OvCa
Sample_Group

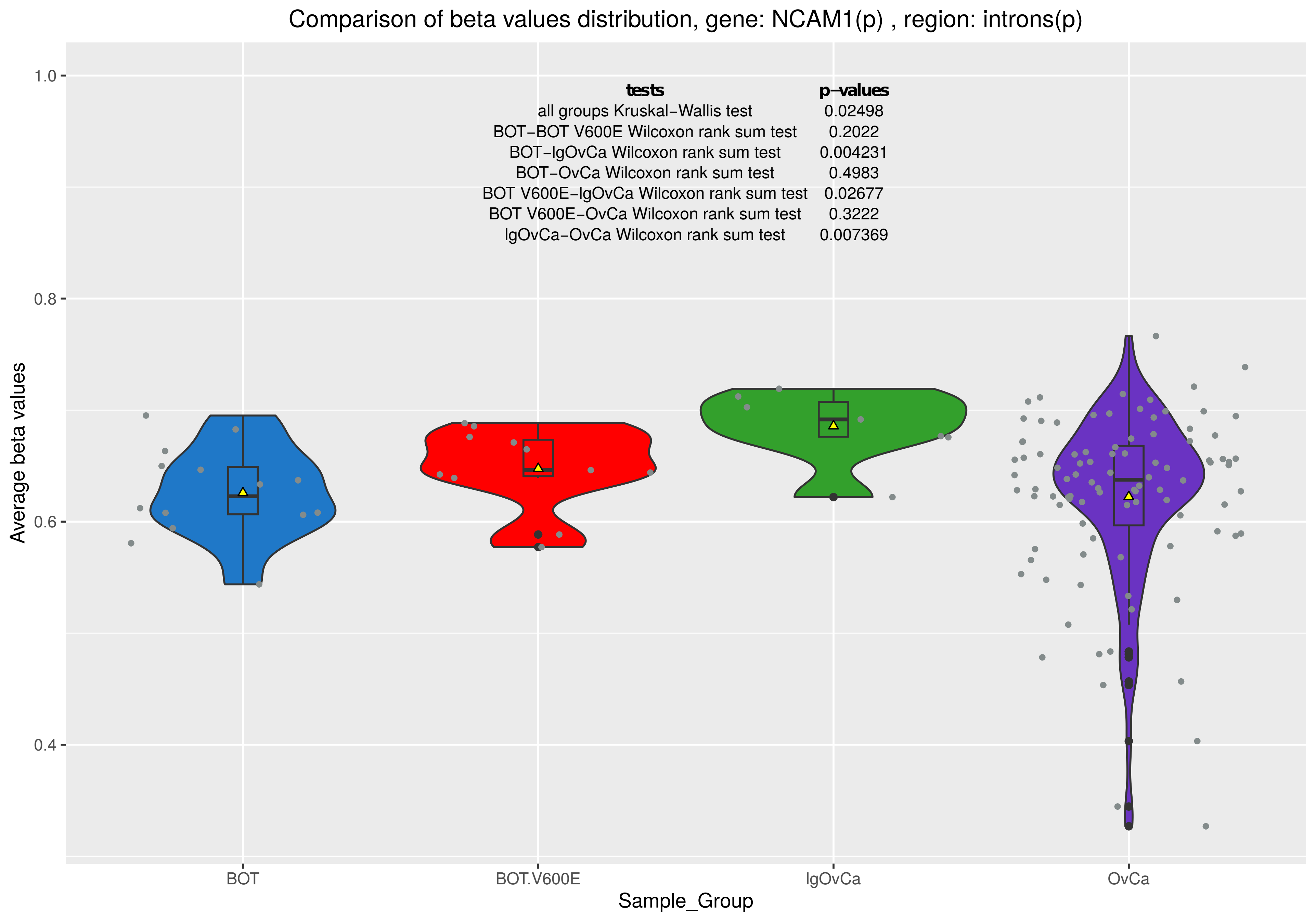

Comparison of beta values distribution, gene: NCAM1(p) , region: introns(p)
tests
p−values
all groups Kruskal−Wallis test 0.02498
BOT−BOT V600E Wilcoxon rank sum test 0.2022
BOT−lgOvCa Wilcoxon rank sum test 0.004231
BOT−OvCa Wilcoxon rank sum test 0.4983
BOT V600E−lgOvCa Wilcoxon rank sum test 0.02677
BOT V600E−OvCa Wilcoxon rank sum test 0.3222
lgOvCa−OvCa Wilcoxon rank sum test 0.007369
Average beta values
1.0
0.8
0.6
0.4
BOT
BOT.V600E
lgOvCa
OvCa
Sample_Group

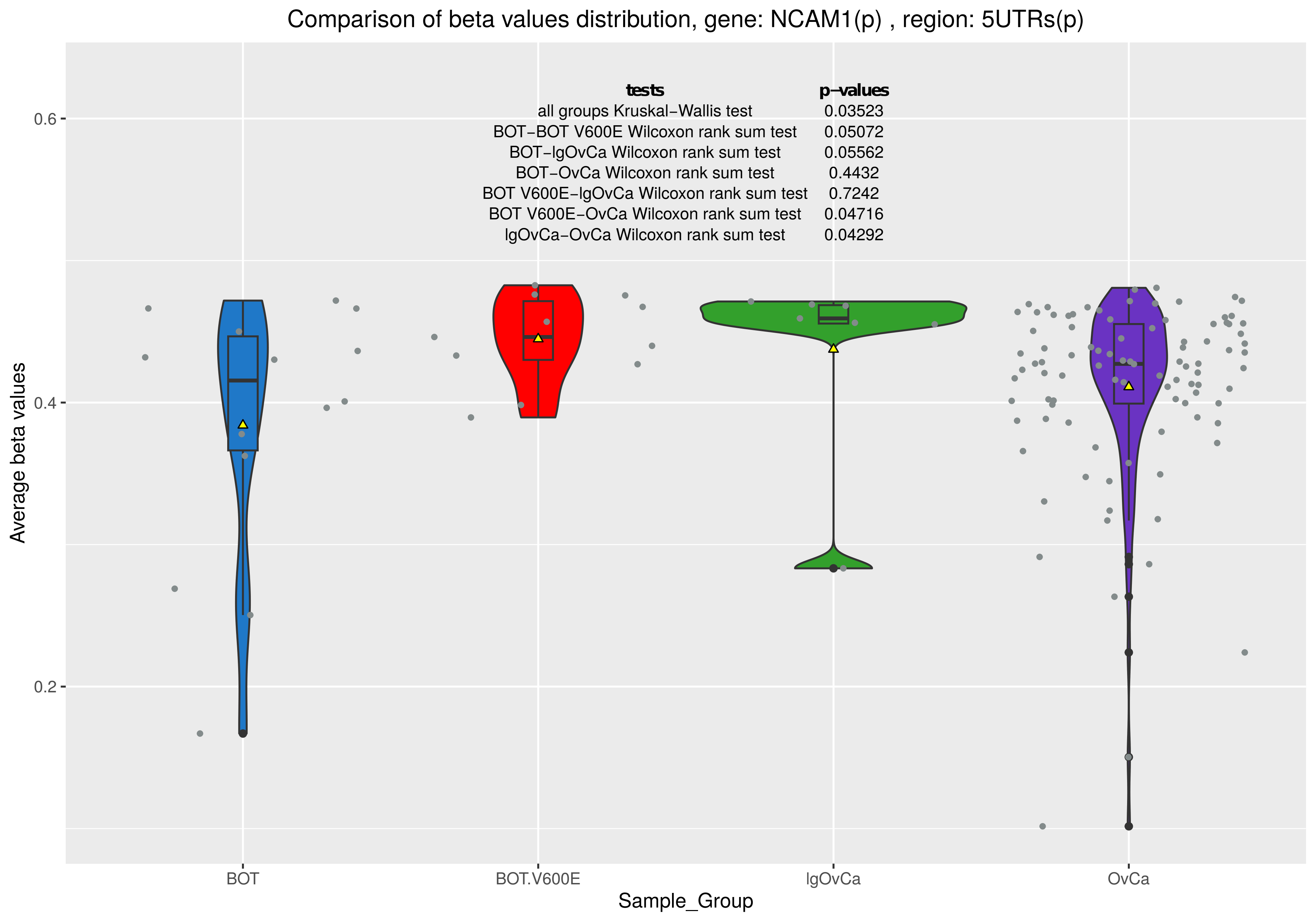
Comparison of beta values distribution, gene: NCAM1(p) , region: 5UTRs(p)
tests
p−values
all groups Kruskal−Wallis test
0.03523
BOT−BOT V600E Wilcoxon rank sum test
0.05072
BOT−lgOvCa Wilcoxon rank sum test
0.05562
BOT−OvCa Wilcoxon rank sum test
0.4432
BOT V600E−lgOvCa Wilcoxon rank sum test
0.7242
BOT V600E−OvCa Wilcoxon rank sum test
0.04716
lgOvCa−OvCa Wilcoxon rank sum test
0.04292
Average beta values
0.6
0.4
0.2
BOT
BOT.V600E
lgOvCa
OvCa
Sample_Group

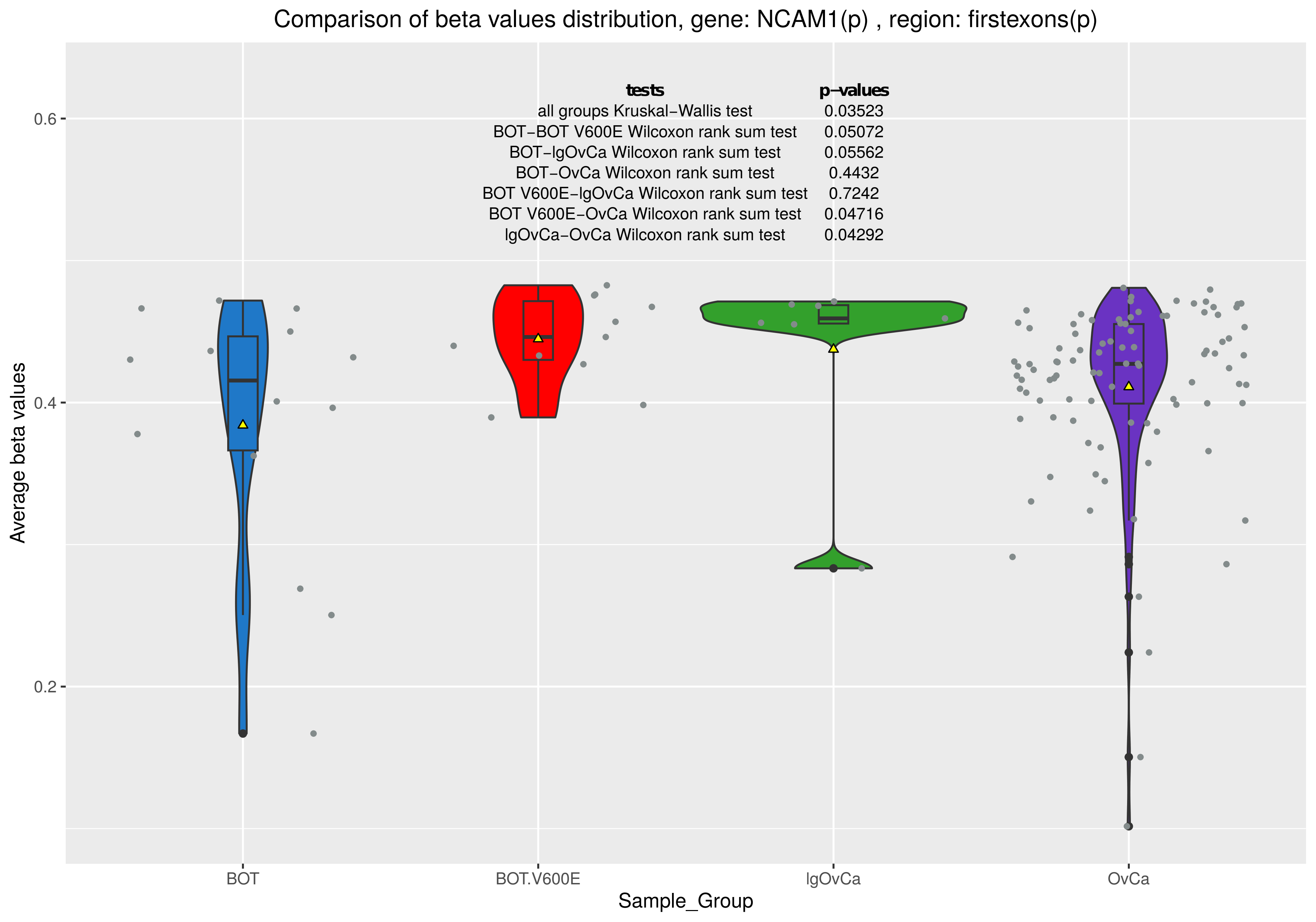

Comparison of beta values distribution, gene: NCAM1(p) , region: firstexons(p)
tests
p−values
all groups Kruskal−Wallis test
0.03523
BOT−BOT V600E Wilcoxon rank sum test
0.05072
BOT−lgOvCa Wilcoxon rank sum test
0.05562
BOT−OvCa Wilcoxon rank sum test
0.4432
BOT V600E−lgOvCa Wilcoxon rank sum test
0.7242
BOT V600E−OvCa Wilcoxon rank sum test
0.04716
lgOvCa−OvCa Wilcoxon rank sum test
0.04292
Average beta values
0.6
0.4
0.2
BOT
BOT.V600E
lgOvCa
OvCa
Sample_Group

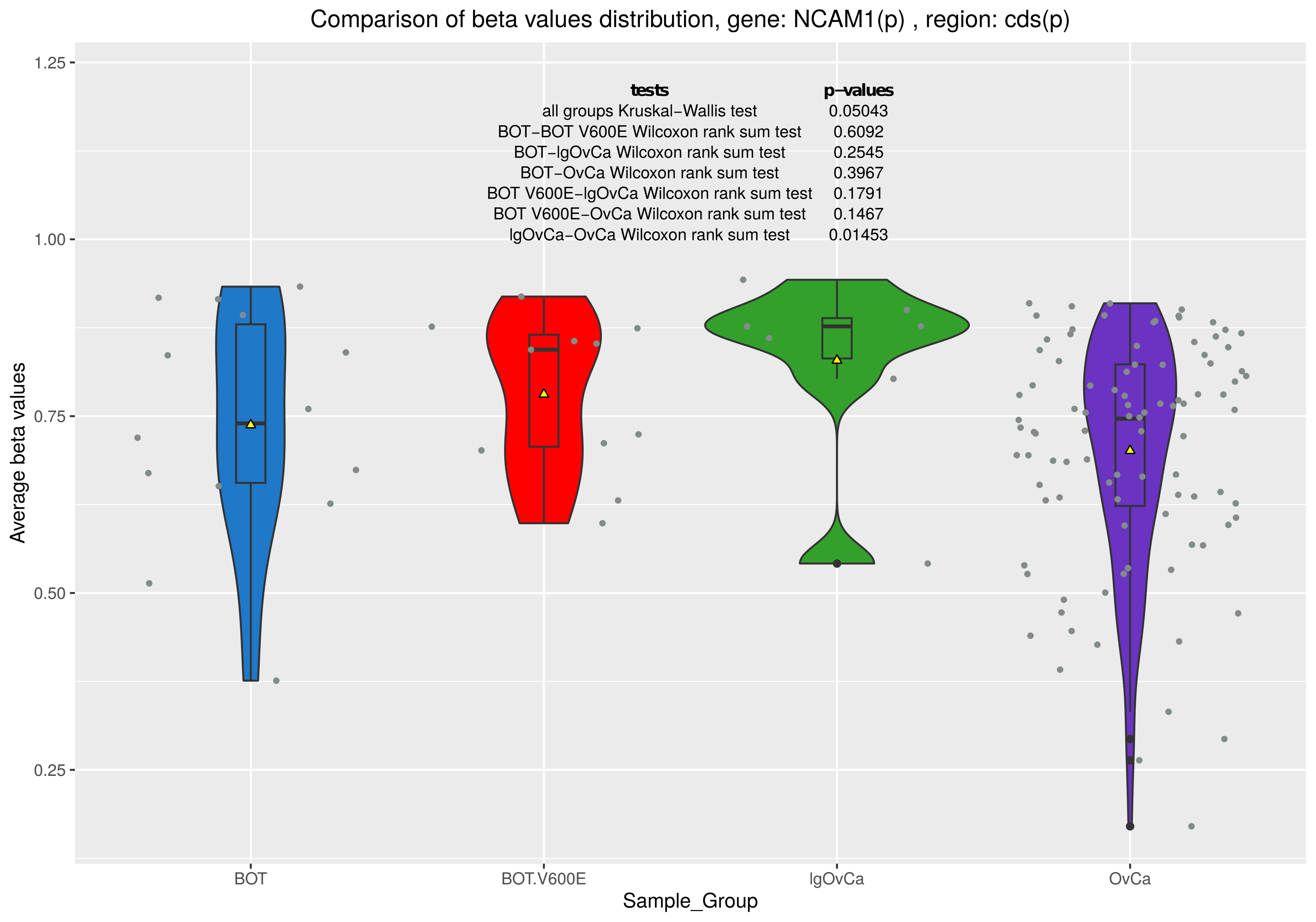

Comparison of beta values distribution, gene: NCAM1(p) , region: cds(p)
tests
p−values
all groups Kruskal−Wallis test
0.05043
BOT−BOT V600E Wilcoxon rank sum test
0.6092
BOT−IgOvCa Wilcoxon rank sum test
0.2545
BOT−OvCa Wilcoxon rank sum test
0.3967
BOT V600E−IgOvCa Wilcoxon rank sum test
0.1791
BOT V600E−OvCa Wilcoxon rank sum test
0.1467
IgOvCa−OvCa Wilcoxon rank sum test
0.01453
Average beta values
1.25
1.00
0.75
0.50
0.25
BOT
BOT.V600E
IgOvCa
OvCa
Sample_Group

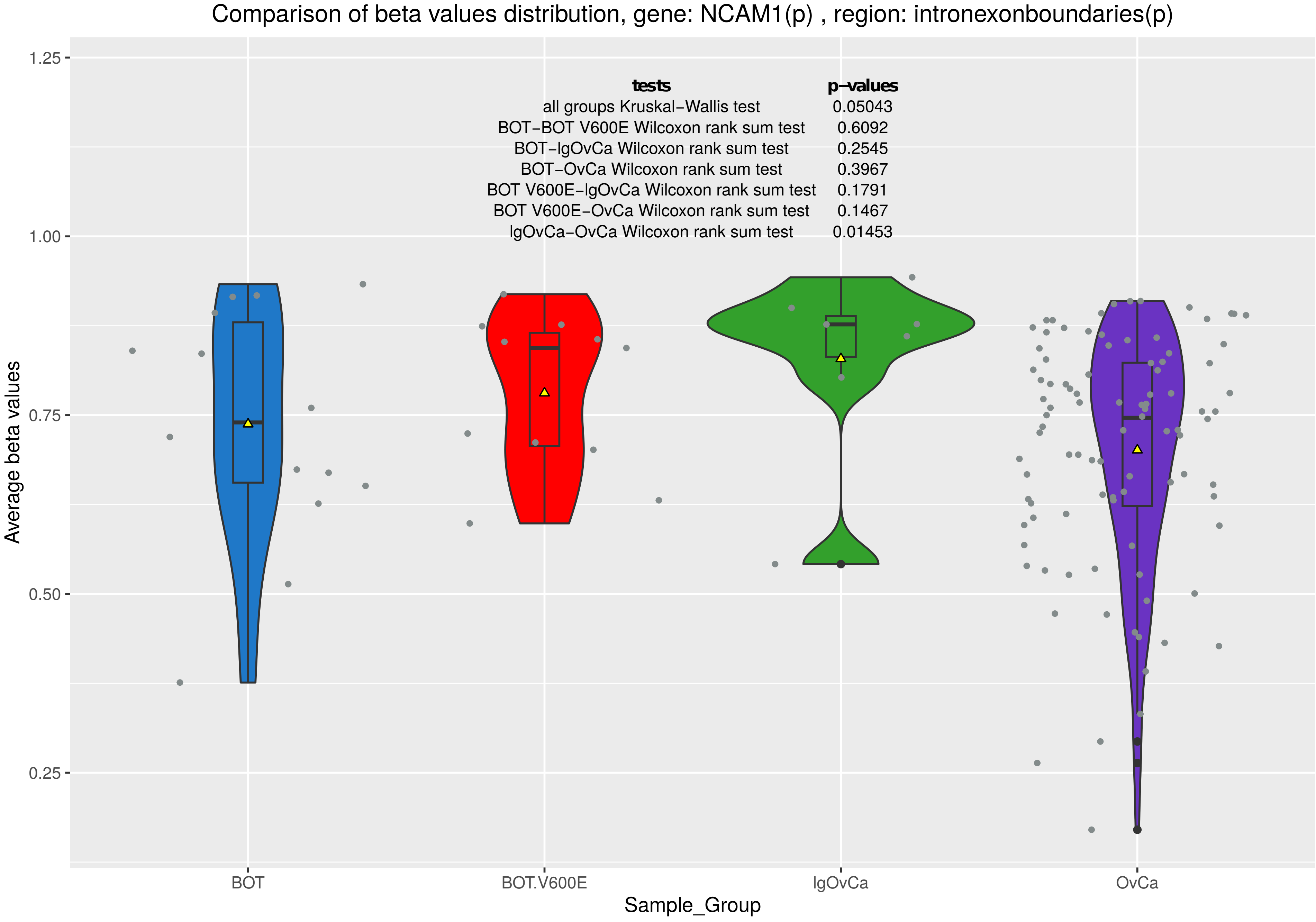

Comparison of beta values distribution, gene: NCAM1(p) , region: intronexonboundaries(p)
tests
p−values
all groups Kruskal−Wallis test
0.05043
BOT−BOT V600E Wilcoxon rank sum test
0.6092
BOT−lgOvCa Wilcoxon rank sum test
0.2545
BOT−OvCa Wilcoxon rank sum test
0.3967
BOT V600E−lgOvCa Wilcoxon rank sum test
0.1791
BOT V600E−OvCa Wilcoxon rank sum test
0.1467
lgOvCa−OvCa Wilcoxon rank sum test
0.01453
Average beta values
1.25
1.00
0.75
0.50
0.25
BOT
BOT.V600E
lgOvCa
OvCa
Sample_Group

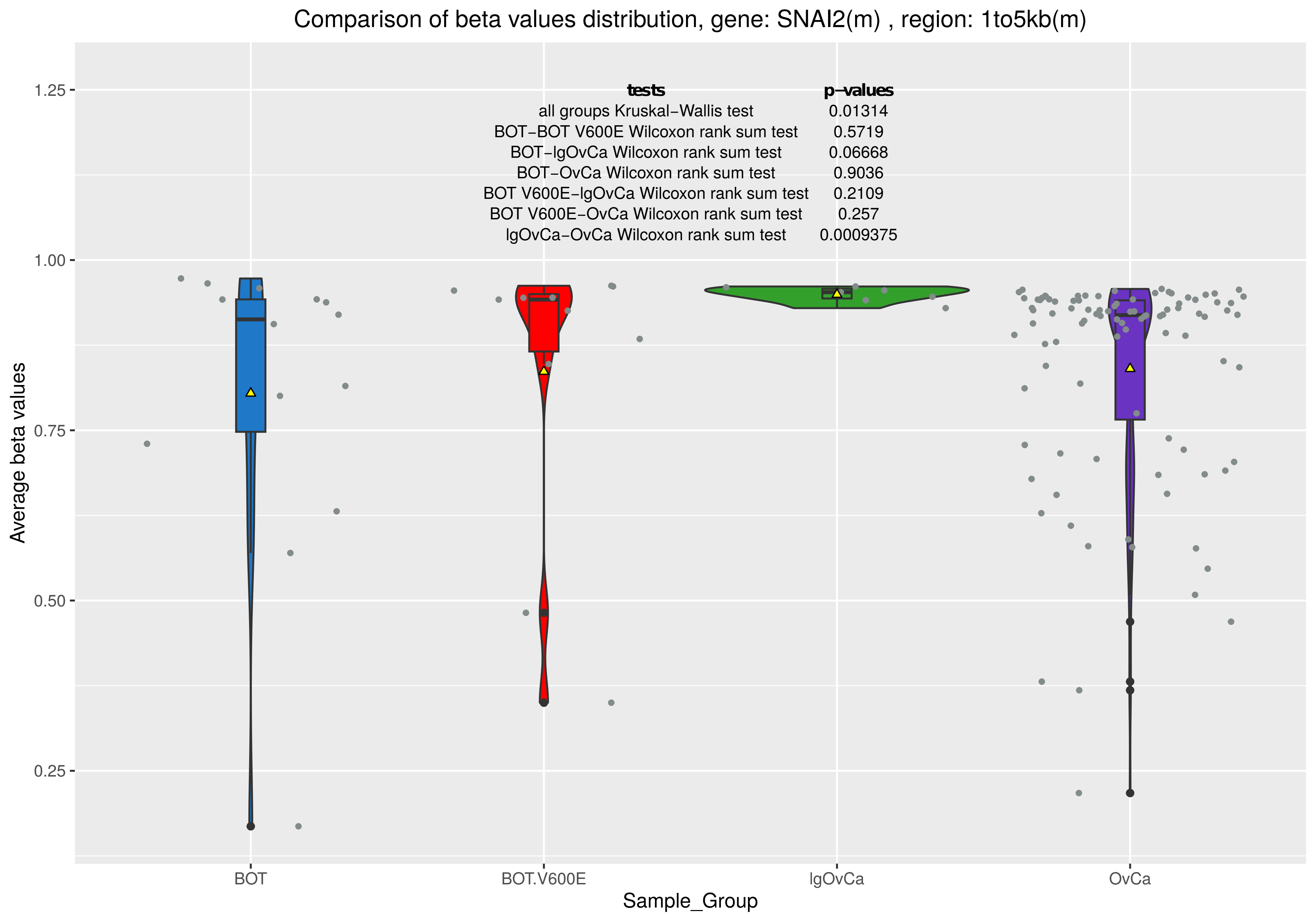
Comparison of beta values distribution, gene: SNAI2(m) , region: 1to5kb(m)
tests
p−values
all groups Kruskal−Wallis test
0.01314
BOT−BOT V600E Wilcoxon rank sum test
0.5719
BOT−lgOvCa Wilcoxon rank sum test
0.06668
BOT−OvCa Wilcoxon rank sum test
0.9036
BOT V600E−lgOvCa Wilcoxon rank sum test
0.2109
BOT V600E−OvCa Wilcoxon rank sum test
0.257
lgOvCa−OvCa Wilcoxon rank sum test
0.0009375
Average beta values
1.25
1.00
0.75
0.50
0.25
BOT
BOT.V600E
lgOvCa
OvCa
Sample_Group

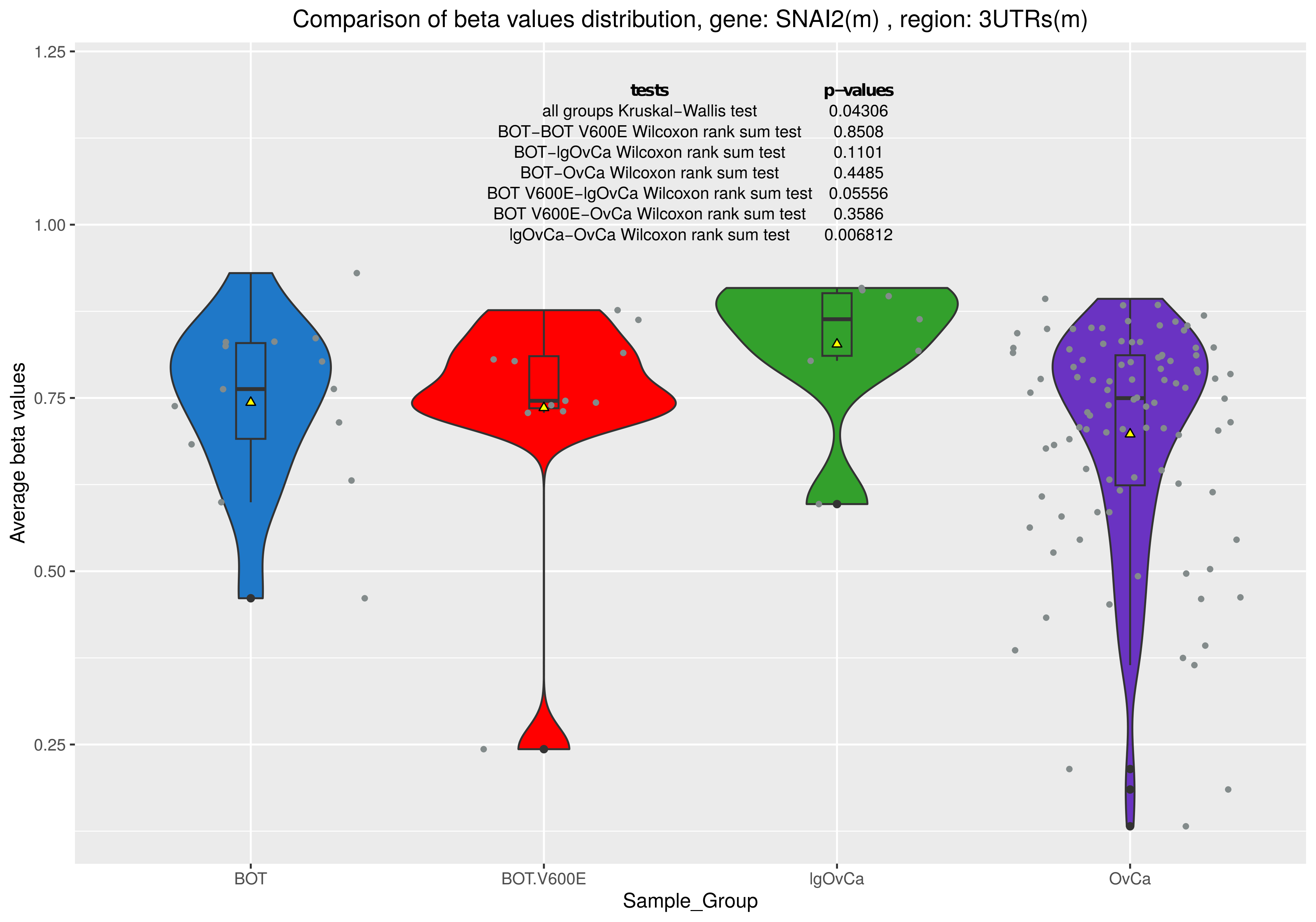
Comparison of beta values distribution, gene: SNAI2(m) , region: 3UTRs(m)
tests
p−values
all groups Kruskal−Wallis test
0.04306
BOT−BOT V600E Wilcoxon rank sum test
0.8508
BOT−lgOvCa Wilcoxon rank sum test
0.1101
BOT−OvCa Wilcoxon rank sum test
0.4485
BOT V600E−lgOvCa Wilcoxon rank sum test
0.05556
BOT V600E−OvCa Wilcoxon rank sum test
0.3586
lgOvCa−OvCa Wilcoxon rank sum test
0.006812
Average beta values
1.25
1.00
0.75
0.50
0.25
BOT
BOT.V600E
lgOvCa
OvCa
Sample_Group

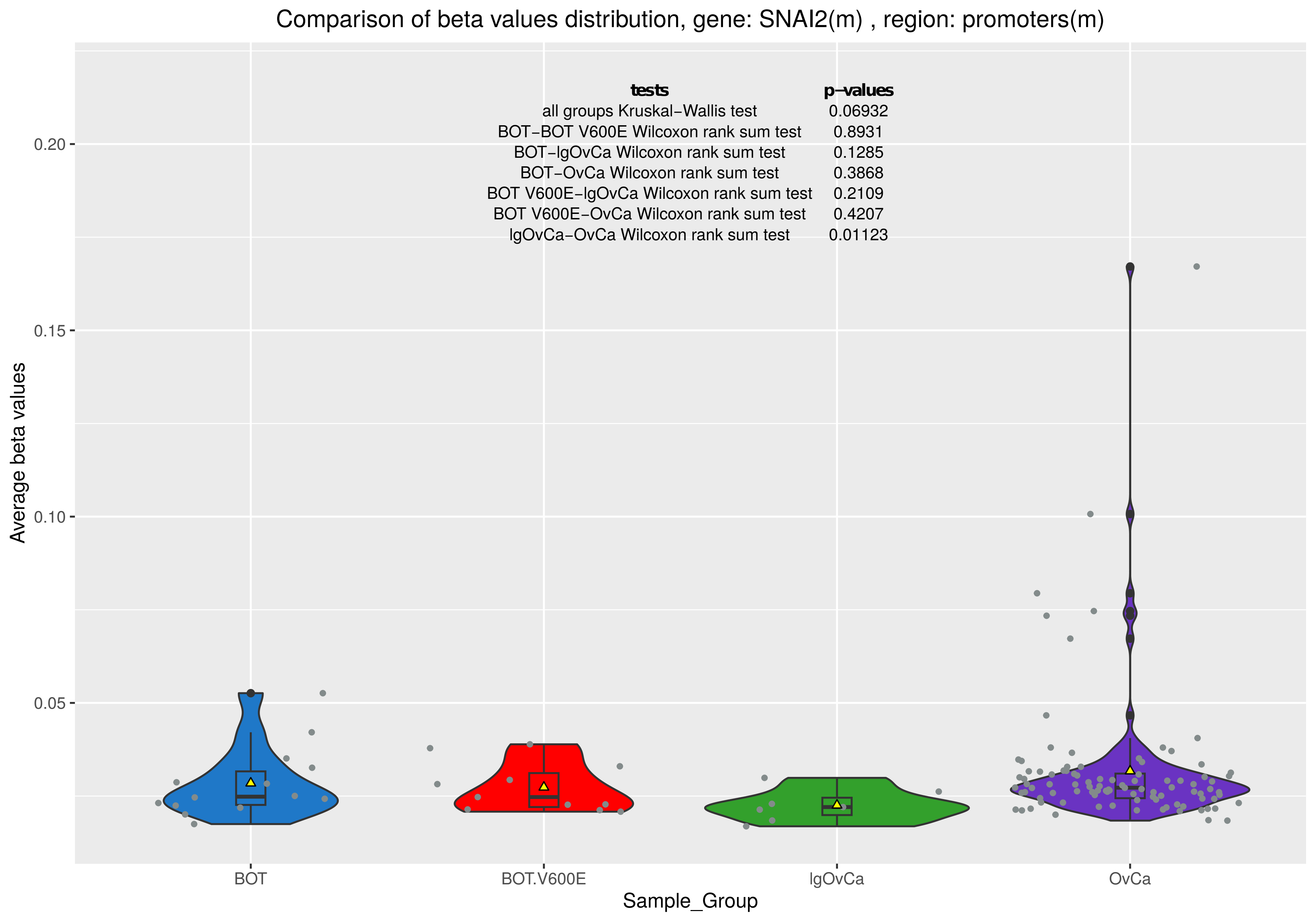

Comparison of beta values distribution, gene: SNAI2(m) , region: promoters(m)
tests
p−values
all groups Kruskal−Wallis test
0.06932
BOT−BOT V600E Wilcoxon rank sum test
0.8931
BOT−lgOvCa Wilcoxon rank sum test
0.1285
BOT−OvCa Wilcoxon rank sum test
0.3868
BOT V600E−lgOvCa Wilcoxon rank sum test
0.2109
BOT V600E−OvCa Wilcoxon rank sum test
0.4207
lgOvCa−OvCa Wilcoxon rank sum test
0.01123
Average beta values
0.20
0.15
0.10
0.05
BOT
BOT.V600E
lgOvCa
OvCa
Sample_Group

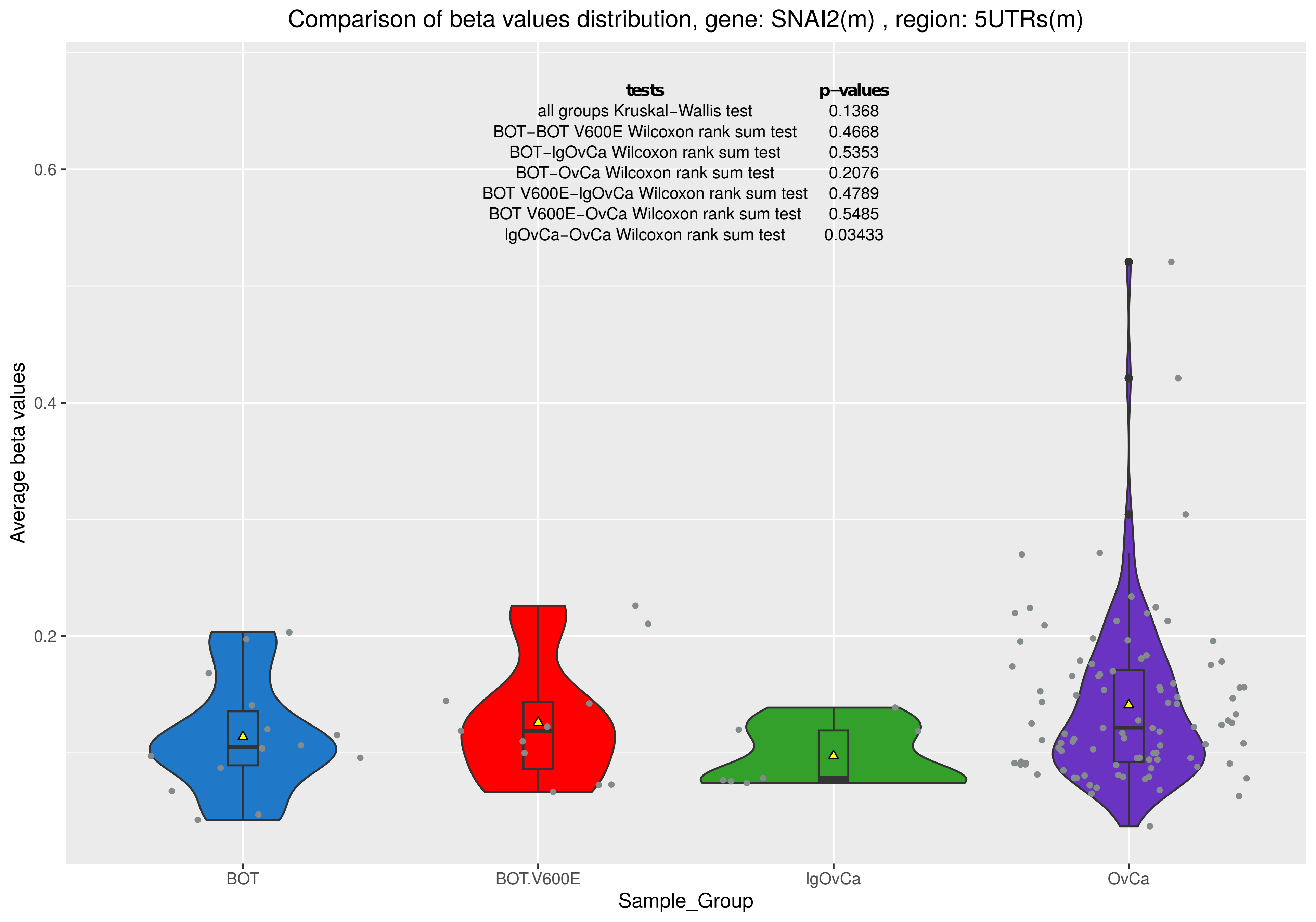
Comparison of beta values distribution, gene: SNAI2(m) , region: 5UTRs(m)
tests
p−values
all groups Kruskal−Wallis test
0.1368
BOT−BOT V600E Wilcoxon rank sum test
0.4668
BOT−lgOvCa Wilcoxon rank sum test
0.5353
BOT−OvCa Wilcoxon rank sum test
0.2076
BOT V600E−lgOvCa Wilcoxon rank sum test
0.4789
BOT V600E−OvCa Wilcoxon rank sum test
0.5485
lgOvCa−OvCa Wilcoxon rank sum test
0.03433
Average beta values
0.6
0.4
0.2
BOT
BOT.V600E
lgOvCa
OvCa
Sample_Group

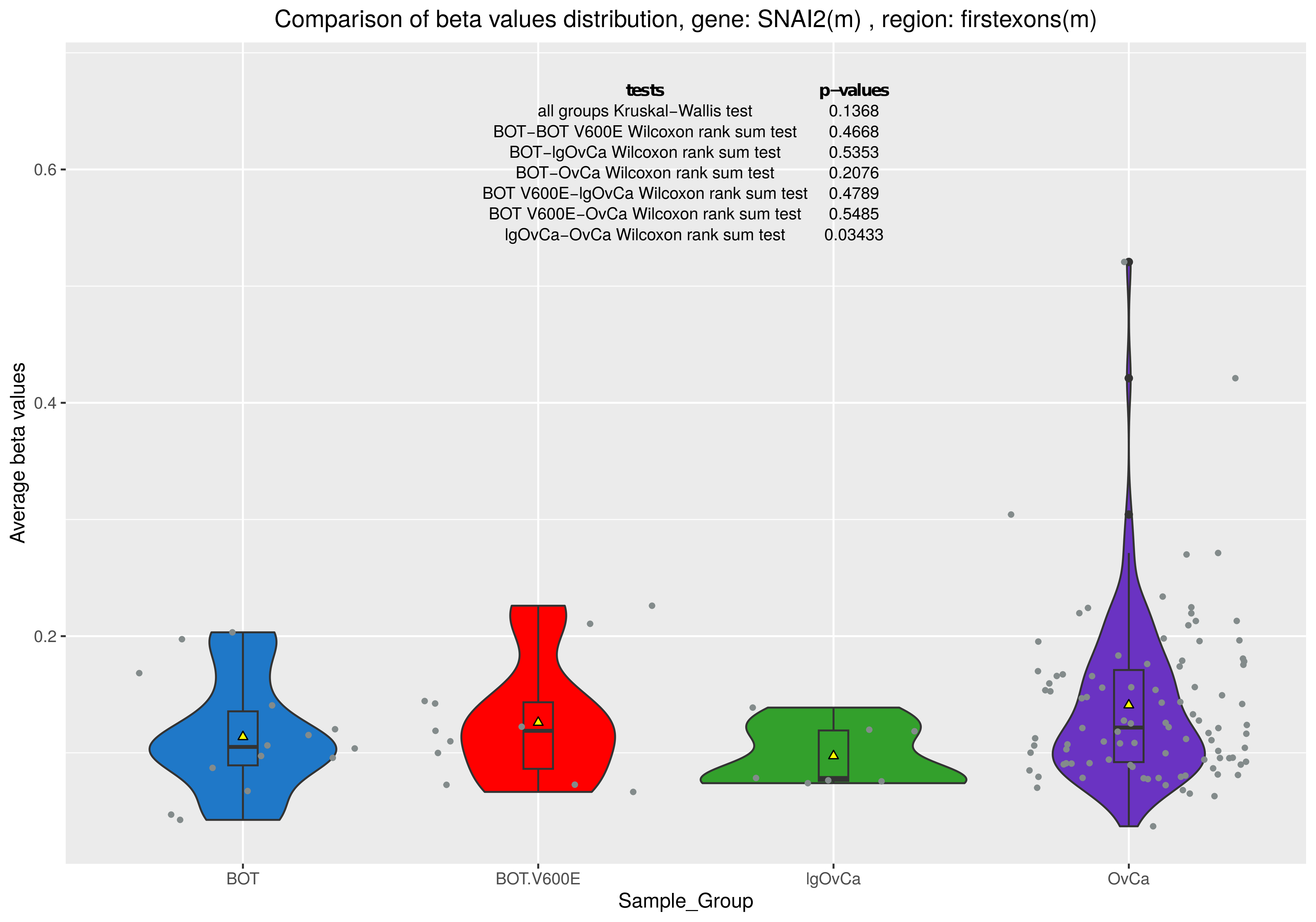

Comparison of beta values distribution, gene: SNAI2(m) , region: firstexons(m)
tests
p−values
all groups Kruskal−Wallis test 0.1368
BOT−BOT V600E Wilcoxon rank sum test 0.4668
BOT−lgOvCa Wilcoxon rank sum test 0.5353
BOT−OvCa Wilcoxon rank sum test 0.2076
BOT V600E−lgOvCa Wilcoxon rank sum test 0.4789
BOT V600E−OvCa Wilcoxon rank sum test 0.5485
lgOvCa−OvCa Wilcoxon rank sum test 0.03433
Average beta values
0.6
0.4
0.2
BOT
BOT.V600E
lgOvCa
OvCa
Sample_Group

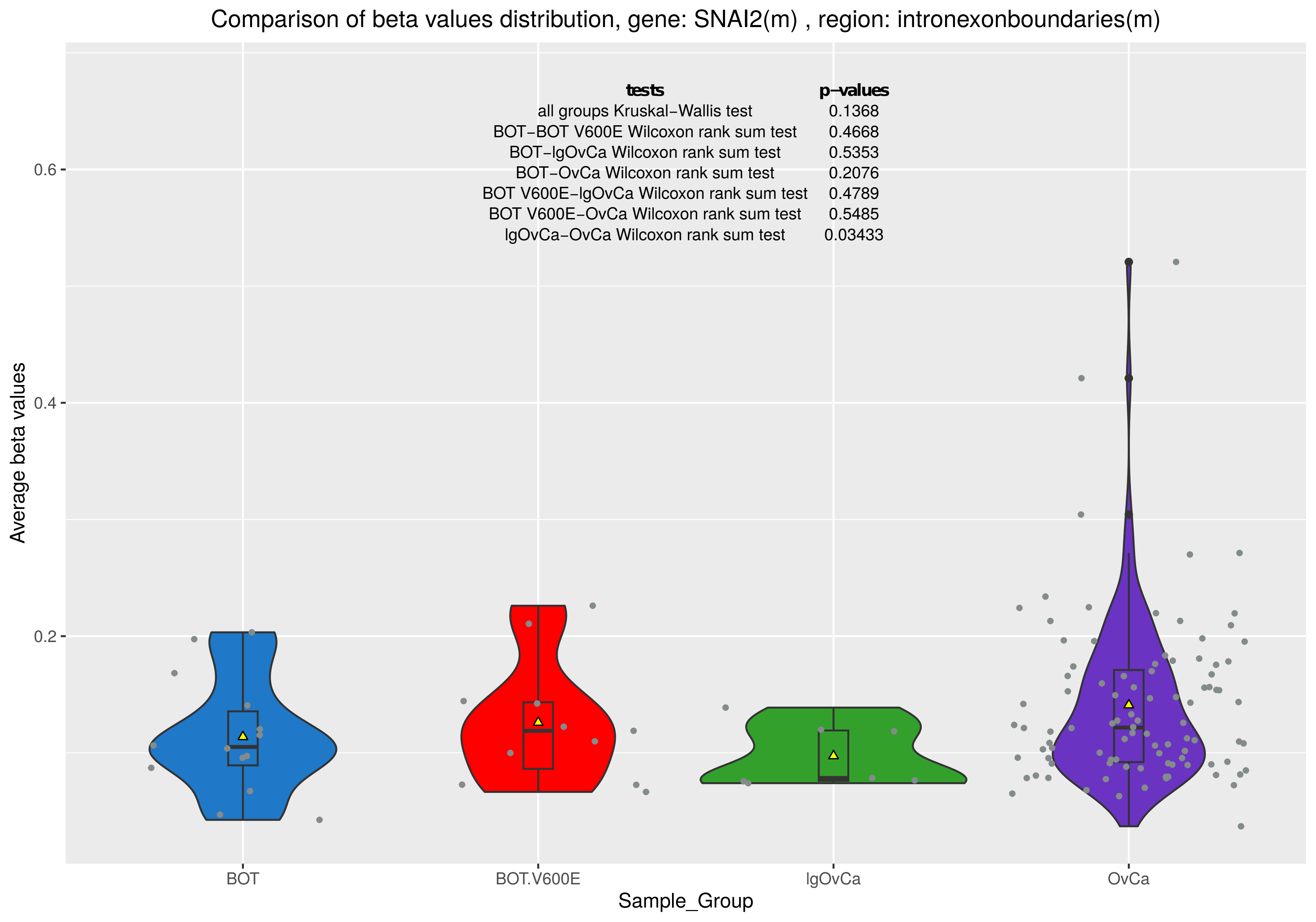

Comparison of beta values distribution, gene: SNAI2(m) , region: intronexonboundaries(m)
tests
p−values
all groups Kruskal−Wallis test
0.1368
BOT−BOT V600E Wilcoxon rank sum test
0.4668
BOT−lgOvCa Wilcoxon rank sum test
0.5353
BOT−OvCa Wilcoxon rank sum test
0.2076
BOT V600E−lgOvCa Wilcoxon rank sum test
0.4789
BOT V600E−OvCa Wilcoxon rank sum test
0.5485
lgOvCa−OvCa Wilcoxon rank sum test
0.03433
Average beta values
0.6
0.4
0.2
BOT
BOT.V600E
lgOvCa
OvCa
Sample_Group

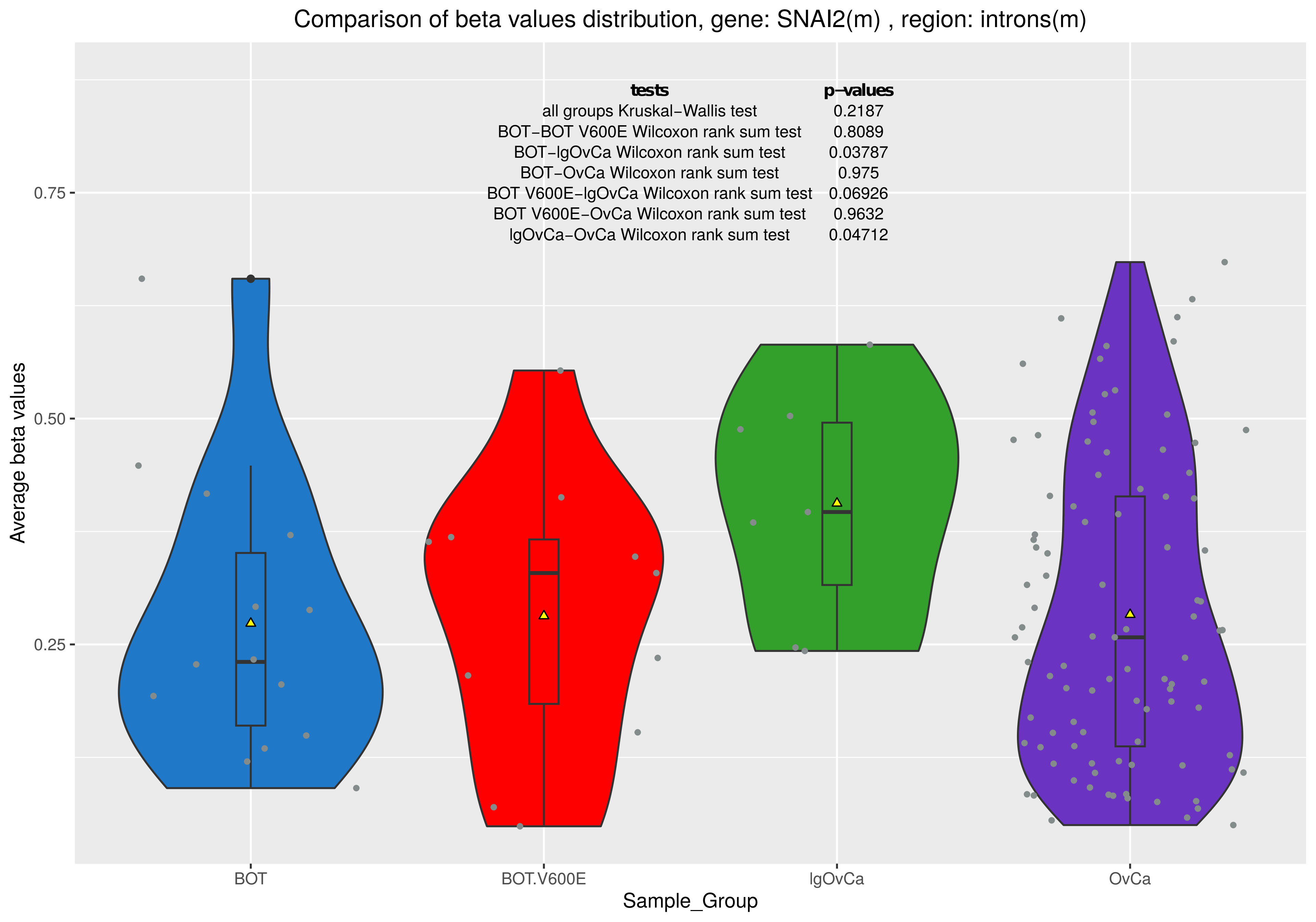
Comparison of beta values distribution, gene: SNAI2(m) , region: introns(m)
tests
p−values
all groups Kruskal−Wallis test
0.2187
BOT−BOT V600E Wilcoxon rank sum test
0.8089
BOT−lgOvCa Wilcoxon rank sum test
0.03787
BOT−OvCa Wilcoxon rank sum test
0.975
BOT V600E−lgOvCa Wilcoxon rank sum test
0.06926
BOT V600E−OvCa Wilcoxon rank sum test
0.9632
lgOvCa−OvCa Wilcoxon rank sum test
0.04712
0.75
0.50
0.25
Average beta values
BOT
BOT.V600E
lgOvCa
OvCa
Sample_Group

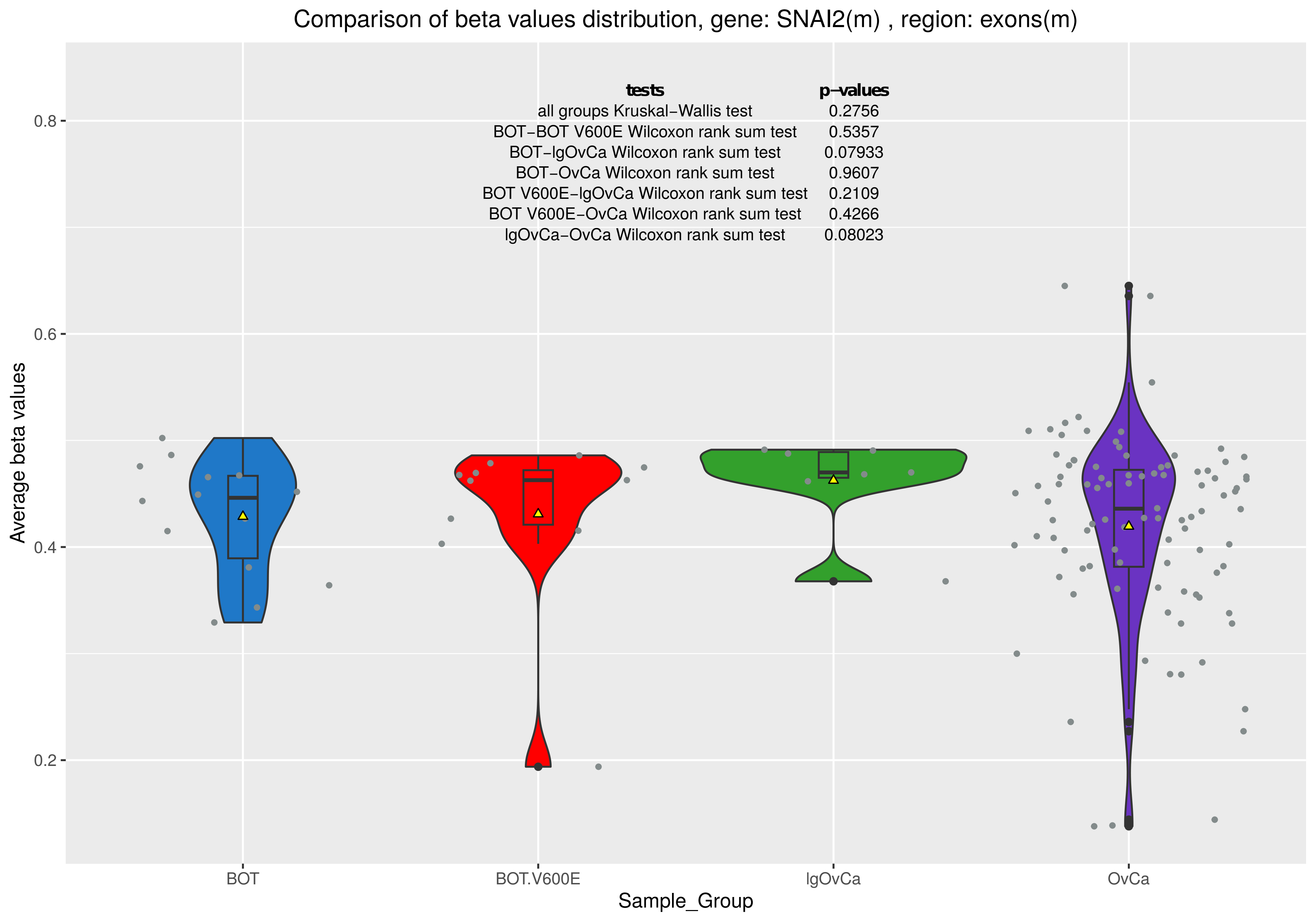
Comparison of beta values distribution, gene: SNAI2(m) , region: exons(m)
tests
p−values
all groups Kruskal−Wallis test
0.2756
BOT−BOT V600E Wilcoxon rank sum test
0.5357
BOT−lgOvCa Wilcoxon rank sum test
0.07933
BOT−OvCa Wilcoxon rank sum test
0.9607
BOT V600E−lgOvCa Wilcoxon rank sum test
0.2109
BOT V600E−OvCa Wilcoxon rank sum test
0.4266
lgOvCa−OvCa Wilcoxon rank sum test
0.08023
Average beta values
0.8
0.6
0.4
0.2
BOT
BOT.V600E
lgOvCa
OvCa
Sample_Group
