# Supplementary material for: The Diversity of Methylation Patterns in Serous Borderline Ovarian Tumors and Serous Ovarian Carcinomas
Source: Cancers (Basel). 2024 Oct 18;16(20):3524. doi: 10.3390/cancers16203524 (PMC11505613; doi:10.3390/cancers16203524)
Supplement: Supplementary file 1 [file cancers-16-03524-s001.zip › Szafron et al.-supplement.pdf]

Table S1. The clinicopathological characteristics of the BOTS series used in the present study.

BRAF.V600E	No	Yes	All_samples
N	14	10	24
Microinvasion_or_ni_implants.No	8	5	13
Microinvasion_or_ni_implants.Yes	6	5	11
Relapse.No	12	8	20
Relapse.Yes	2	2	4
RFS.Min.	1502	293	293
RFS.1st Qu.	4597.75	2755	3629
RFS.Median	5155	4139	4890
RFS.Mean	4960.36	3811.1	4481.5
RFS.3rd Qu.	5411.25	5361.25	5478.75
RFS.Max.	7085	6140	7085
Chemotherapy.No	14	10	24
Primary_tumor.Yes	14	10	24
FIGO.IA-IB	4	3	7
FIGO.IC	4	4	8
FIGO.IIA-IIIC	6	3	9
Type.serous	14	10	24
Age.Min.	18	25	18
Age.1st Qu.	29.25	29	28.75
Age.Median	43.5	33.5	35.5
Age.Mean	44.29	36.4	41
Age.3rd Qu.	58.75	37.5	49.75
Age.Max.	74	76	76
Frozen_samples	4	4	8
FFPE_samples	10	6	16

Microinvasion_or_ni_implants – microinvasion or non-invasive implants; RFS – relapse-free survival; Type – histological type; Qu. – quartile.

Table S2. The clinicopathological characteristics of the OvCa series used in the present study.

Therapy	PC	PC	TP	TP	All_samples
**TP53.acc.**	**0**	**1**	**0**	**1**	
**N**	9	13	31	50	103
**CR.0**	4	5	10	13	32
**CR.1**	5	8	21	37	71
**Relapse.0**	0	1	2	5	8
**Relapse.1**	5	7	19	32	63
**Relapse.NA**	4	5	10	13	32
**Death.0**	0	1	2	9	12
**Death.1**	9	12	29	41	91
**PS.0**	4	9	13	17	43
**PS.1**	5	4	18	33	60
**DFS.Min.**	0	0	0	0	0
**DFS.1st Qu.**	0	0	0	22.5	0
**DFS.Median**	226	127	237	381	229
**DFS.Mean**	270.44	337.62	288.45	516.3	403.69
**DFS.3rd Qu.**	523	222	458.5	727	562.5
**DFS.Max.**	815	2521	1205	2452	2521
**OS.Min.**	56	81	263	296	56
**OS.1st Qu.**	467	443	620	891.25	634.5
**OS.Median**	1138	687	853	1236	1066
**OS.Mean**	1196.33	925.69	1168.39	1554.76	1327.76
**OS.3rd Qu.**	1955	1318	1376.5	1953.25	1765.5
**OS.Max.**	2742	2801	3343	5630	5630
**Type.serous**	9	13	31	50	103
**FIGO ≤ IIC**	0	0	1	1	2
**FIGO.IIIA-IIIB**	1	2	4	4	11
**FIGO.IIIC**	5	10	24	42	81
**FIGO.IV**	3	1	2	3	9
**Grade.lgOvCa**	4	0	3	0	7
**Grade.hgOvCa**	5	13	28	50	96
**RT = 0 cm**	2	1	6	10	19
**RT < 2 cm**	4	3	20	28	55
**RT ≥ 2 cm**	3	9	5	11	28
**RT.NA**	0	0	0	1	1
**Age.Min.**	34	42	29	33	29
**Age.1st Qu.**	43	55	49.5	47	47
**Age.Median**	48	60	54	54	55
**Age.Mean**	52.56	58.38	53.48	54.5	54.51
**Age.3rd Qu.**	64	65	62	62.5	63.5
**Age.Max.**	68	77	74	84	84
**Frozen_samples**	8	13	28	48	97
**FFPE_samples**	1	0	3	2	6

TP53.acc. – TP53 accumulation; CR – complete remission; PS – platinum sensitivity; DFS – disease-free survival; OS – overall survival; Type – histological type; RT – residual tumor size; PC – platinum/cyclophosphamide; TP – taxane/platinum; 0 – “No”; 1 – “Yes”; NA – not applicable/missing data; Qu. – quartile.

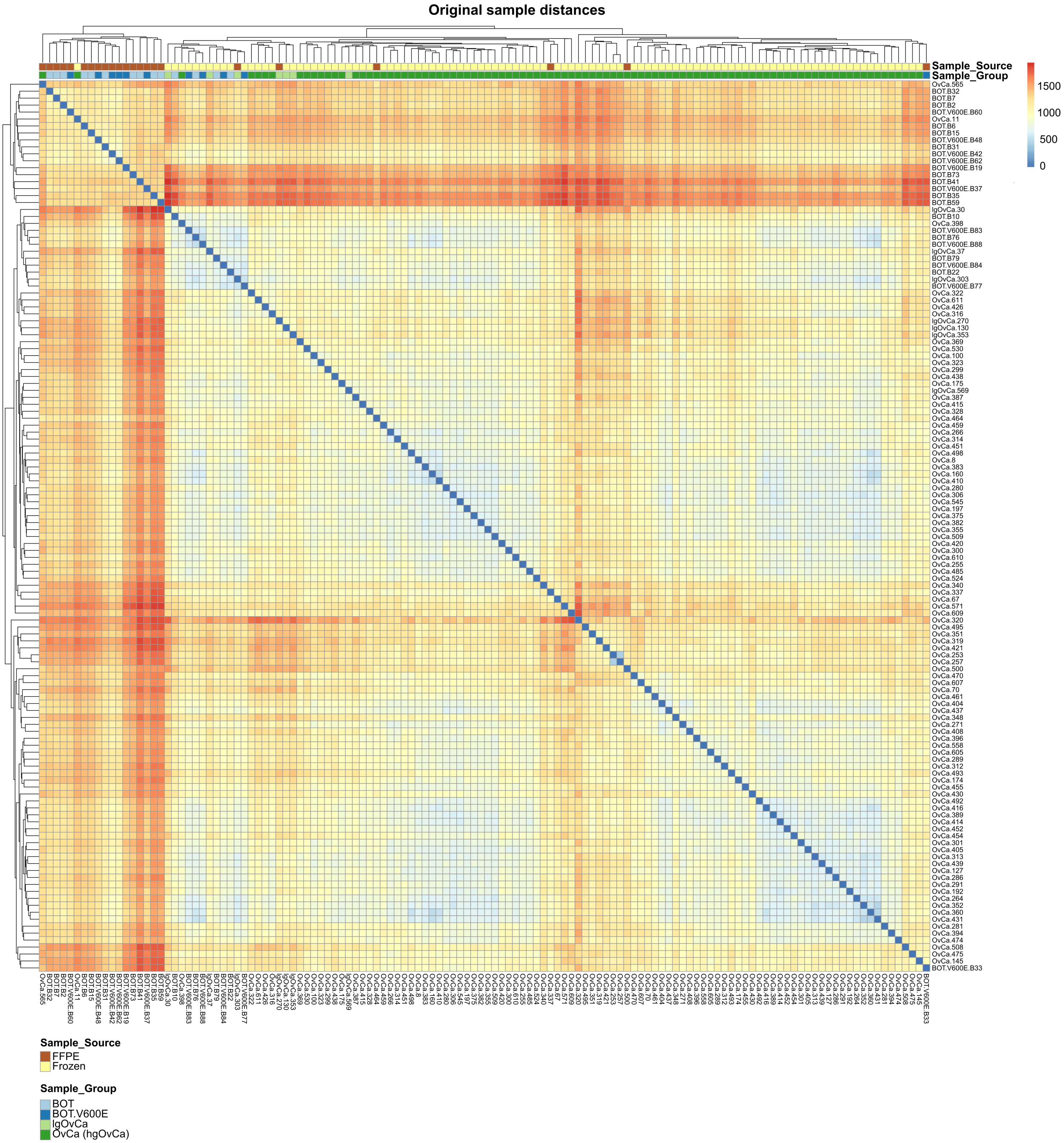

Figure S1. **A heatmap showing distances in overall methylation patterns between the analyzed samples.** For every sample, its group (BOT, BOT.V600E, lgOvCa or hgOvCa (OvCa)) and its source (FFPE blocks (FFPE) or snap-frozen sections (Frozen)) is also displayed on the heatmap.

Table S3. The conditions of methylation-specific PCR and Sanger sequencing for selected genes.

DHDDS/HMGN2						
**Methylation-specific PCR**	95 °C (5 min)	94 °C (30 sec)	50 °C (10 sec)	72 °C (25 sec)	72 °C (5 min)	4 °C (∞)
		40 Cycles				
**Sanger sequencing**	95 °C (2 min)	96 °C (10 sec)	50 °C (5 sec)	60 °C (4 min)	4 °C (∞)	
		35 Cycles				
SKI						
**Methylation-specific PCR**	95 °C (5 min)	94 °C (30 sec)	63 °C (10 sec)	72 °C (25 sec)	72 °C (5 min)	4 °C (∞)
		40 Cycles				
**Sanger sequencing**	95 °C (2 min)	96 °C (10 sec)	63 °C (5 sec)	70 °C (4 min)	4 °C (∞)	
		35 Cycles				
**Sanger sequencing***	95 °C (2 min)	96 °C (10 sec)	50 °C (5 sec)	60 °C (4 min)	4 °C (∞)	
		35 Cycles				

Asterisk (*) indicates alternative approach with addition of 1% DMSO. Primers used in Sanger sequencing, for which good-quality chromatograms were obtained: *DHDDS/HMGN2:* Reverse, *SKI:* Reverse (without DMSO), Forward (with 1% DMSO).

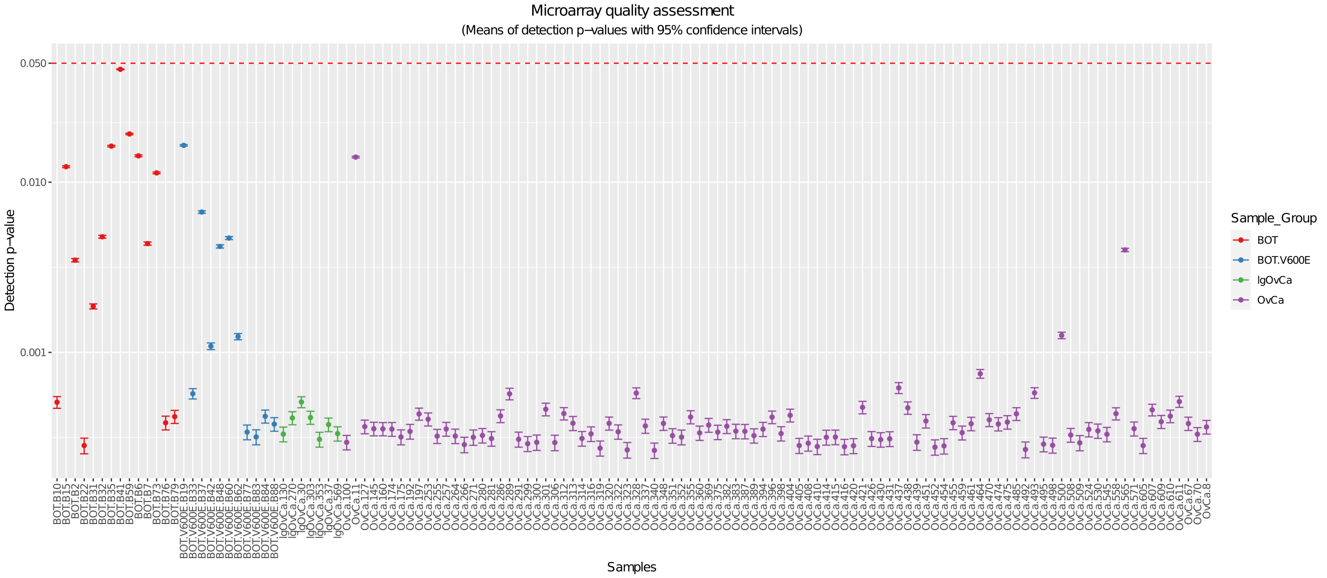

Figure S2. **A hybridization quality assessment for 128 tumor samples.** Red dots – borderline ovarian tumors without the *BRAF* V600E mutation (BOT); blue dots – borderline ovarian tumors with the *BRAF* V600E mutation (BOT.V600E); green dots – low-grade ovarian cancers (lgOvCa), violet dots – high-grade ovarian cancers (OvCa). All the samples passed the quality filter of the detection p-value < 0.05, denoted with a red dashed line.

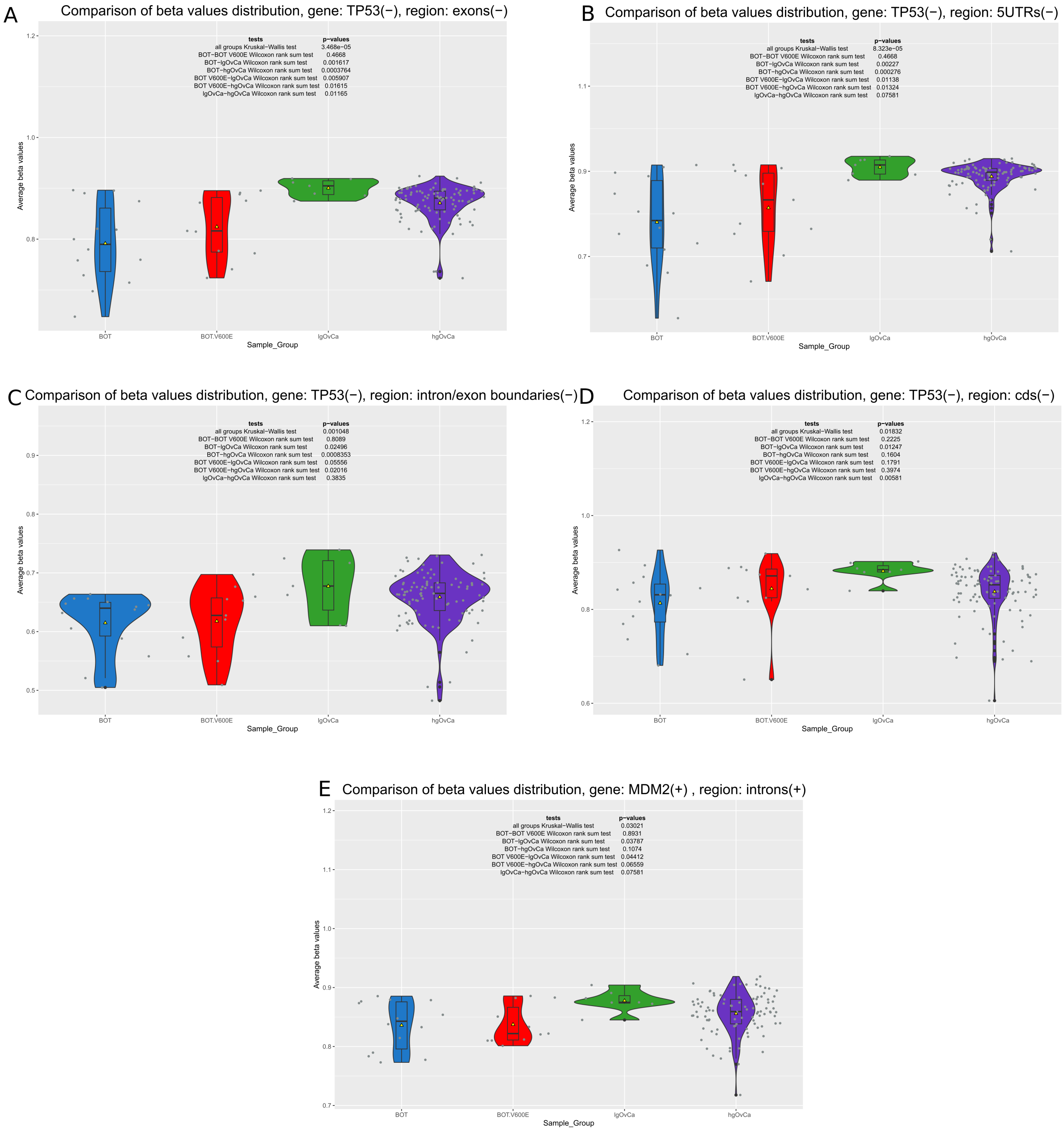

Figure S3. **Violin plots for statistically significant methylation changes (average beta values) in additional regions of the *TP53* and *MDM2* genes (other significant results are presented in Figure 1 in the manuscript).** The values range from 0 to 1 (where 0 means no methylation and 1 denotes 100% methylation of CpGs detected in the region). Each analysis is supplemented with the results of two non-parametric statistical tests: the Kruskal-Wallis test (to determine overall methylation differences between the groups) and the Wilcoxon rank sum test to identify differences between particular groups.

Table S4. **A list of CpGs in all analyzed regions of the *TP53, MDM2* and *CDKN1A* genes.** For a region to be listed here, the methylation pattern of CpGs included in this region had to be significantly different in at least one comparison between tumor groups analyzed herein. For genomic locations of CpG sites listed here, refer to supplementary file: Illumina_Infinium_methyl_EPIC_array_hg19_ext_attributes.xlsx.

Gene name (strand)	Region	Number of CpGs	List of CpGs (cg number)
TP53(−)	Proximal promoter (−)	8	cg10792831 & cg02087342 & cg13468400 & cg06365412 & cg27105645 & cg12373934 & cg21050342 & cg18311066
TP53(−)	1-5kb (−)	7	cg10792831 & cg02087342 & cg13468400 & cg06365412 & cg27105645 & cg10653997 & cg26867494
TP53(−)	1st exon (−)	1	cg10792831
TP53(−)	Exons (−)	3	cg10792831 & cg13468400 & cg06365412
TP53(−)	5'UTRs (−)	2	cg10792831 & cg06365412
TP53(−)	intron/exon boundaries(−)	5	cg10792831 & cg13468400 & cg06365412 & cg27105645 & cg12373934
TP53(−)	cds(−)	1	cg13468400
MDM2(+)	Proximal promoter (+) = 1-5kb (+)	4	cg00614420 & cg11025645 & cg15434599 & cg05085337
MDM2(+)	Introns (+)	4	cg17456064 & cg05111885 & cg16296697 & cg12611405
CDKN1A(+)	Proximal promoter	5	cg05460965 & cg21091547 & cg11920449 & cg03714916 & cg03354771
CDKN1A(+)	1st exon (=5'UTR,=exons)	2	cg11920449 & cg03354771

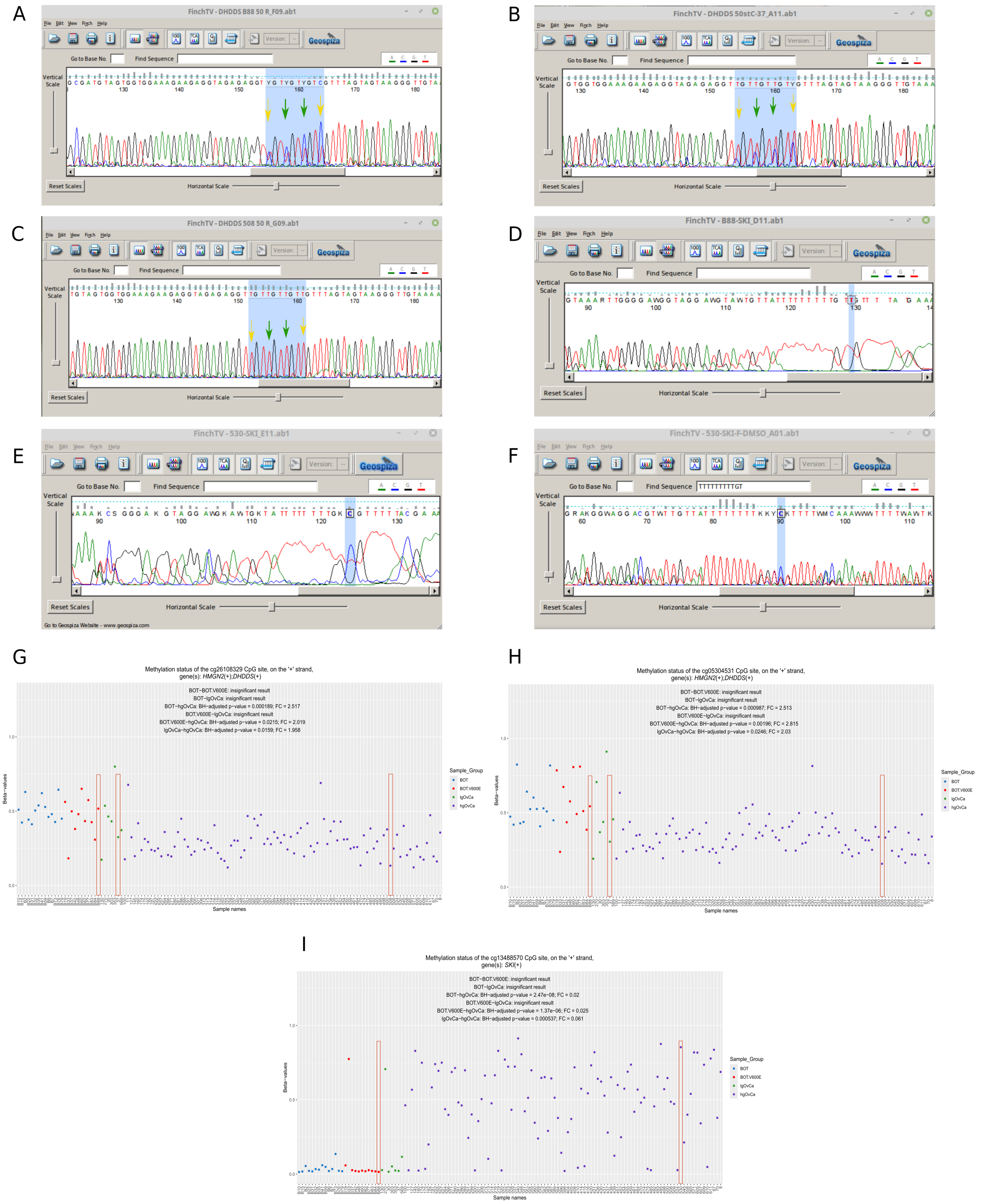

Figure S4. **The validation of methylation microarray data for three CpGs**. The methylation level of two CpGs in the *HMGN2*/*DHDDS* gene, cg26108329; chr1:g.(+)26797585 & cg05304531, chr1:g.(+)26797576, and one CpG in the *SKI*(+) gene, cg13488570; chr1:g.(+)2222253, was assessed by Sanger sequencing (A-F) and methylation microarrays (G-I). In chromatograms (A-F), each relevant CpG site(s) is/are highlighted in blue. Yellow arrows in A-C denote CpGs in *HMGN2*/*DHDDS* that have been analyzed with EPIC microarrays, too, whereas green arrows indicate other CpGs, not detected by these microarrays. Remarkably, methylation patterns of all the four CpG sites in A-C seem to be correlated. Of note, in E and F, the same PCR product is sequenced from either the reverse primer (E) or the forward primer with the addition of DMSO (F). In dot plots (G-I), red rectangles indicate samples for which the results of methylation-specific PCR and Sanger sequencing are presented in A-F. Each dot-plot is additionally supplemented with significant Benjamini-Hochberg (BH)-adjusted p-values of the linear regression analysis and corresponding fold change (FC) values.

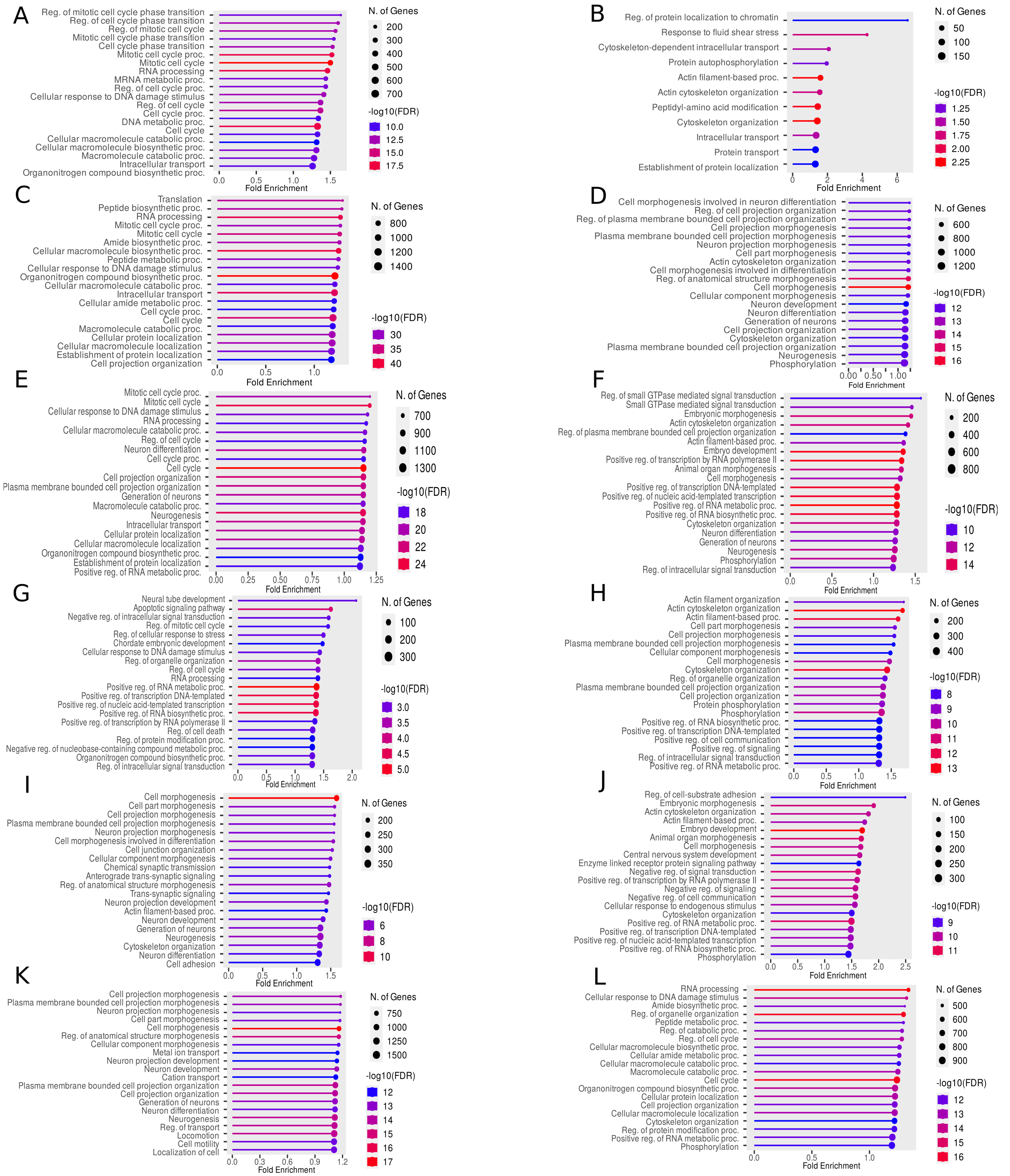

Figure S5. **The GO enrichment analysis for the most differentiative biological processes (BP) in all inter-tumor-group comparisons.** A,B: BOT vs. BOT.V600E, C,D: BOT vs. lgOvCa, E,F: BOT vs. hgOvCa, G,H: BOT.V600E vs. lgOvCa, I,J: BOT.V600E vs. hgOvCa, and K,L: lgOvCa vs. hgOVCa. On the y-axis, ontological terms are listed. The terms were sorted by the decreasing fold enrichment, shown on the x-axis. In each row, the terms for up-methylated genes (in the first of the compared groups) are presented on the left, while those for down-methylated genes (also in the first group) are on the right side of the figure. The FDR cutoff point was set to 0.1.

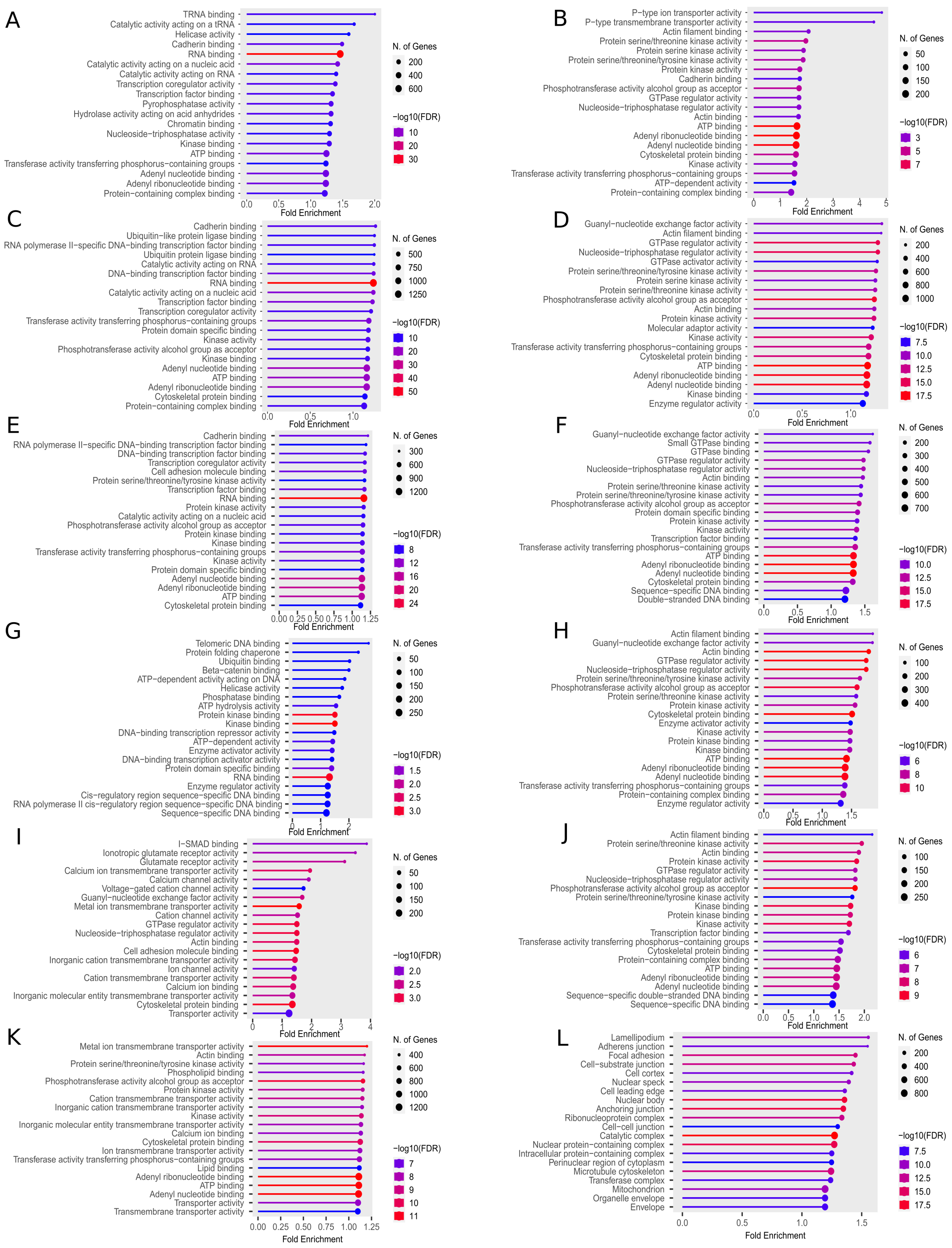

Figure S6. **The GO enrichment analysis for the most differentiative molecular functions (MF) in all inter-tumor-group comparisons.** A,B: BOT vs. BOT.V600E, C,D: BOT vs. lgOvCa, E,F: BOT vs. hgOvCa, G,H: BOT.V600E vs. lgOvCa, I,J: BOT.V600E vs. hgOvCa, and K,L: lgOvCa vs. hgOVCa. On the y-axis, ontological terms are listed. The terms were sorted by the decreasing fold enrichment, shown on the x-axis. In each row, the terms for up-methylated genes (in the first of the compared groups) are presented on the left, while those for down-methylated genes (also in the first group) are on the right side of the figure. The FDR cutoff point was set to 0.1.

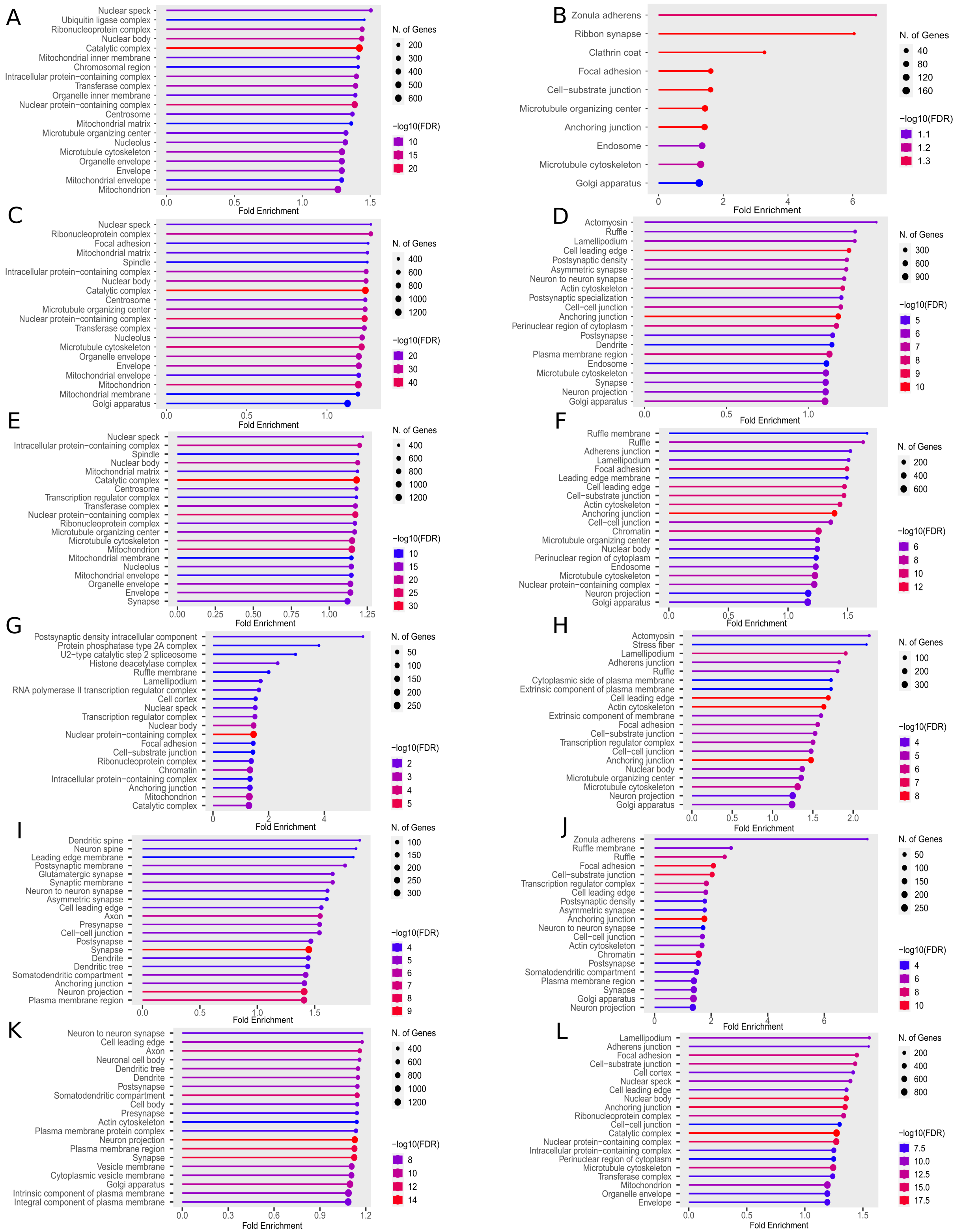

Figure S7. **The GO enrichment analysis for the most differentiative cellular components (CC) in all inter-tumor-group comparisons.** A,B: BOT vs. BOT.V600E, C,D: BOT vs. lgOvCa, E,F: BOT vs. hgOvCa, G,H: BOT.V600E vs. lgOvCa, I,J: BOT.V600E vs. hgOvCa, and K,L: lgOvCa vs. hgOVCa. On the y-axis, ontological terms are listed. The terms were sorted by the decreasing fold enrichment, shown on the x-axis. In each row, the terms for up-methylated genes (in the first of the compared groups) are presented on the left, while those for down-methylated genes (also in the first group) are on the right side of the figure. The FDR cutoff point was set to 0.1.

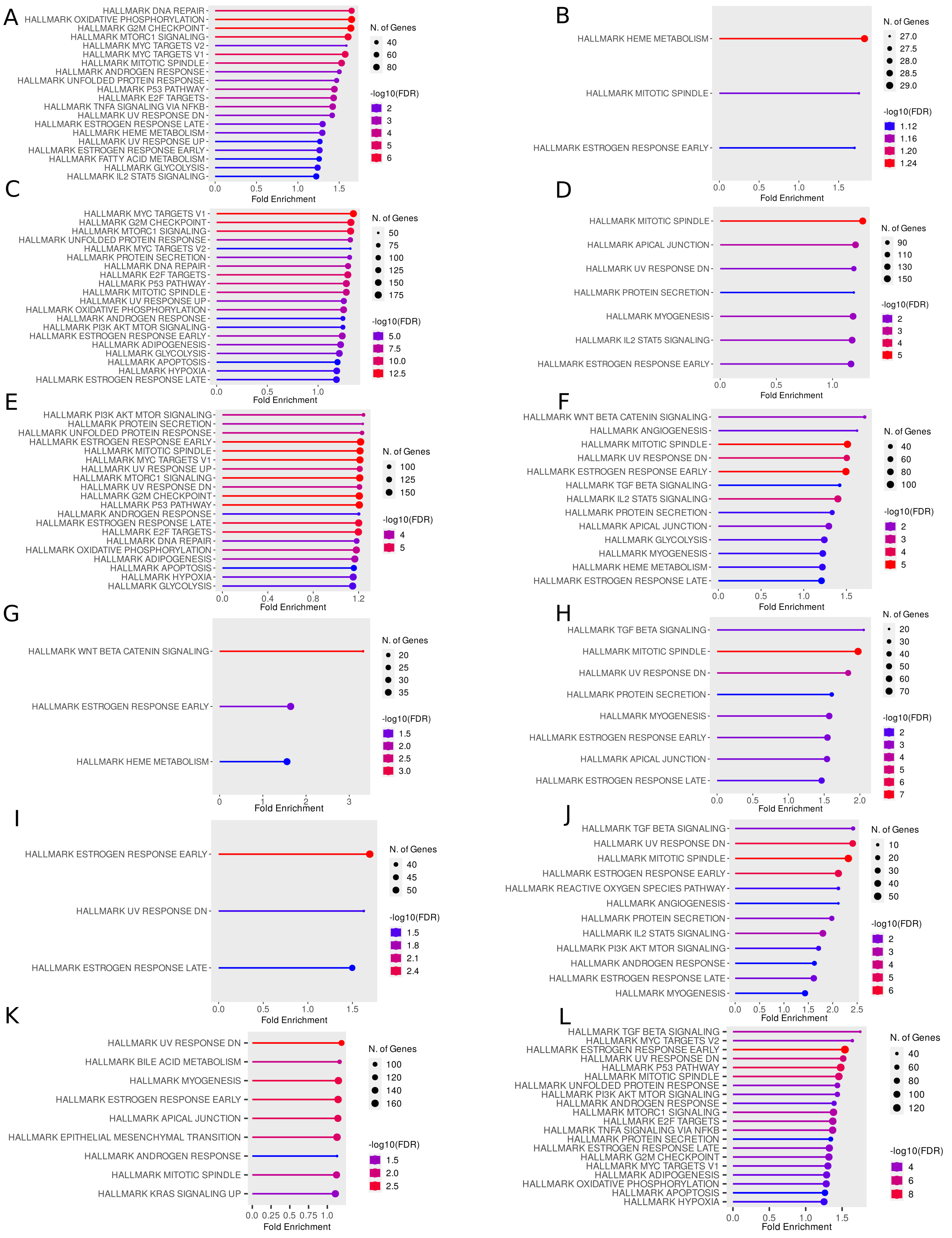

Figure S8. **The Molecular Signature Database (MSigDB) analysis (Hallmark gene sets) in all inter-tumor-group comparisons.** A,B: BOT vs. BOT.V600E, C,D: BOT vs. lgOvCa, E,F: BOT vs. hgOvCa, G,H: BOT.V600E vs. lgOvCa, I,J: BOT.V600E vs. hgOvCa, and K,L: lgOvCa vs. hgOVCa. On the y-axis, ontological terms are listed. The terms were sorted by the decreasing fold enrichment, shown on the x-axis. In each row, the terms for up-methylated genes (in the first of the compared groups) are presented on the left, while those for down-methylated genes (also in the first group) are on the right side of the figure. The FDR cutoff point was set to 0.1.

Table S5. The ShinyGO results for genes/DMRs matching between uni- and multivariable regression analyses in hgOvCa.

Cox regression analyses		
**BP – biological process**		
**N**	**High level GO category**	**Genes**
24	GO:0051234 establishment of localization	*DNAJC5, PRELID3B, ZPR1, PACSIN1, HIKESHI, FAM3B, TCN2, SYN3, SLC24A5, MYO6, TST, DLG4, BMP8A, CD5L, HMOX1, PKDREJ, SPTBN4, CLDN1, PLA2G4F, SERPINA5, INHBA, RIPOR1, TBC1D1, CAMK2G*
20	GO:0033036 macromolecule localization	*PRELID3B, ZPR1, DLG4, HIKESHI, SPTBN4, MAPRE2, GPC6, FAM3B, TST, AXIN2, BMP8A, MYL12A, PACSIN1, PLA2G4F, SERPINA5, MYO6, INHBA, RIPOR1, TBC1D1, CAMK2G*
20	GO:0065008 regulation of biological quality	*MYL12A, HMOX1, DLG4, CNOT8, CDC42EP3, GPC6, FAM3B, SYN3, SLC24A5, INHBA, BMP8A, ACADVL, HSD17B12, SPTBN4, CLDN1, NDST2, TBC1D1, AXIN2, CAMK2G, DNAJC5*
19	GO:0048583 regulation of response to stimulus	*HMOX1, INHBA, MCF2L, NREP, ARHGEF3, DUSP7, BMP8A, RBBP7, AXIN2, CD5L, SGCA, DLG4, MAPRE2, GPC6, RIPOR1, TBC1D1, TIMP3, CLDN1, CAMK2G*
17	GO:0042221 response to chemical	*TIMP3, NREP, NTN3, CXCL3, BMP8A, RBBP7, HMOX1, ZPR1, INHBA, DLG4, CLDN1, AXIN2, PLA2G4F, TP53I13, RIPOR1, TBC1D1, MPST*
16	GO:0023051 regulation of signaling	*HMOX1, INHBA, MCF2L, NREP, ARHGEF3, DUSP7, BMP8A, RBBP7, AXIN2, DLG4, MAPRE2, GPC6, RIPOR1, TBC1D1, TIMP3, SYN3*
16	GO:0032879 regulation of localization	*DNAJC5, ZPR1, PACSIN1, GPC6, RBBP7, BMP8A, HMOX1, DLG4, SPTBN4, MAPRE2, INHBA, RIPOR1, TBC1D1, CLDN1, MYO6, CAMK2G*
15	GO:0065009 regulation of molecular function	*TBC1D1, TIMP3, SERPINA5, DUSP7, ANXA4, HMOX1, PAPLN, MCF2L, DLG4, NRBF2, ARHGEF3, MAPRE2, AXIN2, RIPOR1, PLAAT4*
14	GO:0006950 response to stress	*MYL12A, HMOX1, NREP, CXCL3, RBBP7, HIKESHI, MYO6, ACADVL, CD5L, NRBF2, CLDN1, RIPOR1, AXIN2, INHBA*
14	GO:0050793 regulation of developmental process	*PACSIN1, NREP, CDC42EP3, MTA3, RBBP7, HMOX1, INHBA, MYL12A, ZPR1, DLG4, SPTBN4, AXIN2, GPC6, PLAAT4*
14	GO:0051641 cellular localization	*DNAJC5, ZPR1, DLG4, HIKESHI, SPTBN4, MAPRE2, GPC6, SYN3, MYO6, HMOX1, MYL12A, PACSIN1, RIPOR1, TBC1D1*
13	GO:0009056 catabolic process	*TIMP3, HMOX1, PLBD1, AMDHD1, NRBF2, CNOT8, PLA2G4F, AXIN2, ACADVL, PLAAT4, ZPR1, MPST, TST*
13	GO:0009653 anatomical structure morphogenesis	*PACSIN1, CDH24, SPTBN4, NTN3, CDC42EP3, MYO6, HMOX1, INHBA, MYL12A, DLG4, ACTC1, AXIN2, GPC6*
13	GO:0051239 regulation of multicellular organismal process	*RBBP7, HMOX1, INHBA, MAST2, SGCA, ZPR1, DLG4, SPTBN4, MAPRE2, AXIN2, ANXA4, PLAAT4, CAMK2G*
11	GO:0009893 positive regulation of metabolic process	*ZPR1, INHBA, CNOT8, BMP8A, MTA3, RBBP7, HMOX1, DLG4, AXIN2, MYO6, ACTC1*
10	GO:0003008 system process	*ACTC1, TIMP3, HMOX1, SGCA, DLG4, SPTBN4, NDST2, MYO6, INHBA, CAMK2G*
10	GO:0044085 cellular component biogenesis	*PES1, CDH24, CDC42EP3, CLDN1, MAPRE2, SYNPO, GPC6, DLG4, ACTC1, SPTBN4*

**MF – molecular function**		
14	GO:0016787 hydrolase activity	*MTMR11, CD5L, PAPLN PLBD1, PLAAT4, KIF21A, AMDHD1, CNOT8, DUSP7, NDST2 ABHD15, PLA2G4F, HMOX1, CAMK2G*
11	GO:0098772 molecular function regulator activity	*TBC1D1, TIMP3, INHBA, MCF2L, CXCL3, BMP8A, SERPINA5, PAPLN, ARHGEF3, FAM3B, ANXA4*
10	GO:0016740 transferase activity	*MAST2, CMAS, MPST, TST, PLAAT4, CAMK2G, FDPS, NDST2, GLT8D2, PACSIN1*
**CC – cellular component**		
25	GO:0005576 extracellular region	*MTMR11, RIPOR1, CD5L, TTC38, MYL12A, MCF2L, MPST, TST, ACTC1, SPTBN4, FAM3B, SERPINA5, MYO6, ANXA4, TIMP3, PLBD1, INHBA, CXCL3, BMP8A, TCN2, PAPLN, NTN3, ABHD15, GPC6, HMOX1*
22	GO:0005615 extracellular space	*MTMR11, RIPOR1, CD5L, TTC38, MYL12A, MCF2L, MPST, TST, ACTC1, SPTBN4, FAM3B, SERPINA5, MYO6, ANXA4, TIMP3, INHBA, CXCL3, BMP8A, TCN2, PLBD1, GPC6, HMOX1*
14	GO:0031090 organelle membrane	*DNAJC5, SYN3, ACADVL, HMOX1, HSD17B12, SERPINA5, MYO6, ANXA4, PACSIN1, CAMK2G, NDST2, RIPOR1, DLG4, SLC24A5*
13	GO:0030054 cell junction	*ACTC1, DNAJC5, DLG4, CDH24, SPTBN4, CLDN1, SYNPO, GPC6, SYN3, MAPRE2, SGCA, PACSIN1, MPST*
11	GO:0042995 cell projection	*ZPR1, DLG4, CAMK2G, SPTBN4, PLA2G4F, SYNPO, MYO6, PACSIN1, MPST, KIF21A, ACTC1*
11	GO:0043230 extracellular organelle	*MTMR11, RIPOR1, TTC38, MYL12A, MPST, ACTC1, SPTBN4, FAM3B, SERPINA5, MYO6, ANXA4*
11	GO:1903561 extracellular vesicle	*MTMR11, RIPOR1, TTC38, MYL12A, MPST, ACTC1, SPTBN4, FAM3B, SERPINA5, MYO6, ANXA4*
**Logistic regression analyses**		
**BP – biological process**		
**N**	**High level GO category**	**Genes**
33	GO:0009893 positive regulation of metabolic process	*BRAT1, RMND1, SLAMF6, RFXANK, LDLRAP1, PAX6, CARD10, GLI3, CCND3, ONECUT2, EGR2, WNT1, TAF8, E4F1, HDAC3, IST1, RPS6KA2, JADE1, IRAK3, BABAM1, HAMP, EZH1, KDM3A, TAOK2, ZNF496, LMX1A, TSHR, GATM, IL20RB, RASSF5, CREM, TCP1, MALT1*
30	GO:0051234 establishment of localization	*MPC1, GDAP1, ARRDC2, HAMP, DDX25, LMAN2L, KIF17, VPS13C, NUF2, MCM3AP, SHISA8, GSTO1, MTX1, TOMM7, BORCS8, MRPL18, LDLRAP1, ANKLE1, HDAC3, MALT1, PAX6, GLI3, TCP1, EGR2, FRMD4A, PROCA1, UQCRFS1, IST1, SFT2D1, TAOK2*
28	GO:0033036 macromolecule localization	*TCP1, GDAP1, ARRDC2, DDX25, VPS13C, WDR45B, MCM3AP, IST1, MTX1, TOMM7, MRPL18, TAF8, ANKLE1, HDAC3, PAX6, NRCAM, GLI3, KIF17, EGR2, FRMD4A, PROCA1, SFT2D1, RASSF5, CARD10, CEP128, LMAN2L, LDLRAP1, TAOK2*
28	GO:0048583 regulation of response to stimulus	*MALT1, C18orf32, IRAK3, MFNG, ARMT1, TAOK2, RELL2, SHISA8, BABAM1, JADE1, GLI3, WNT1, ANKLE1, SLAMF6, RASGEF1A, HAMP, RASAL1, CCND3, ONECUT2, WDR83, RGS5, IL20RB, VPS13C, HDAC3, NPTXR, NCAM1, KIF7, ARHGAP39*
27	GO:0065008 regulation of biological	*HAMP, GNA14, BOLA2, SHISA8, GSTO1, BORCS8, TAOK2,*

	quality	*HDAC3, RPS6KA2, NRCAM, MAG, KDM3A, EGR2, ARPC1B, WDTC1, LDLRAP1, TSHR, GATM, MTX1, IL20RB, IST1, RDH14, ARPC1A, CHST8, PAX6, TCP1, TOMM7*
26	GO:0051641 cellular localization	*TCP1, GDAP1, DDX25, LMAN2L, KIF17, VPS13C, WDR45B, NUF2, MCM3AP, GSTO1, MTX1, TOMM7, BORCS8, TAF8, ANKLE1, HDAC3, MALT1, PAX6, HAMP, GLI3, EGR2, RASSF5, CARD10, BRAT1, LDLRAP1, TAOK2*
25	GO:0042221 response to chemical	*IRAK3, NRCAM, GNA14, TSHR, IL20RB, KDM3A, GSTO1, PAX6, RPS6KA2, GDAP1, HAMP, GLI3, RASAL1, CCND3, ONECUT2, EGR2, WDR83, WNT1, VPS13C, ARPC1B, WDTC1, LMX1A, MALT1, LDLRAP1, NCAM1*
22	GO:0023051 regulation of signaling	*MALT1, C18orf32, IRAK3, MFNG, TAOK2, RELL2, SHISA8, JADE1, GLI3, WNT1, RASGEF1A, RASAL1, CCND3, ONECUT2, EGR2, WDR83, RGS5, HDAC3, NPTXR, NCAM1, KIF7, ARHGAP39*
21	GO:0006950 response to stress	*BABAM1, HAMP, BRAT1, ARMT1, RELL2, MALT1, NT5C3A, WNT1, WDR45B, TAOK2, ANKLE1, SLAMF6, HDAC3, IRAK3, MAG, GLI3, WDR83, IL20RB, PAX6, VPS13C, TOMM7*
20	GO:0009653 anatomical structure morphogenesis	*NRCAM, ISM1, SOBP, TAOK2, RASAL1, PAX6, MFNG, MAG, GLI3, ONECUT2, EGR2, WNT1, LMX1A, TSHR, THBS3, IST1, CARD10, IQCE, NCAM1, EZH1*
20	GO:0051239 regulation of multicellular organismal process	*IRAK3, ISM1, SLAMF6, GSTO1, WNT1, PAX6, MAG, HAMP, GLI3, KDM3A, EGR2, TSHR, GATM, MALT1, IL20RB, IST1, CARD10, LDLRAP1, NPTXR, HDAC3*
20	GO:0065009 regulation of molecular function	*IRAK3, CCND3, TSHR, MALT1, RASGEF1A, SHISA8, PAX6, CARD10, GLI3, GSTO1, MFNG, RASAL1, WNT1, WDTC1, RGS5, ARHGAP39, TAOK2, TCP1, LDLRAP1, NPTXR*
19	GO:0044085 cellular component biogenesis	*WDR45B, TMEM70, TAOK2, BOLA2, MAG, ONECUT2, ARPC1B, YBEY, ARPC1A, QSOX1 WNT1, UQCRFS1, HDAC3, TAF8, EZH1, CARD10, TCP1, IST1, NRCAM*
17	GO:0050793 regulation of developmental process	*ISM1, RASAL1, WNT1, TAF8, TAOK2, PAX6, MAG, HAMP, GLI3, KDM3A, EGR2, ANKLE1, LMX1A, TSHR, MALT1, IST1, NRCAM*
17	GO:0051240 positive regulation of multicellular organismal process	*SLAMF6, GSTO1, WNT1, PAX6, IRAK3, MAG, HAMP, GLI3, KDM3A, EGR2, TSHR, GATM, MALT1, IL20RB, IST1, LDLRAP1, HDAC3*
16	GO:0009605 response to external stimulus	*NRCAM, HAMP, MALT1, IRAK3, NT5C3A, WDR45B, SLAMF6, PAX6, GDAP1, MAG, GLI3, EGR2, WDR83, LMX1A, IL20RB, NCAM1*
15	GO:0002376 immune system process	*SLAMF6, MALT1, CDH26, MCM3AP, IRAK3, MFNG, BABAM1, HAMP, GLI3, CCND3, WNT1, ANKLE1, TSHR, IL20RB, RASSF5*
15	GO:0040007 growth	*JADE1, RASAL1, MAG, HAMP, GLI3, TAF8, WDTC1, LMX1A, TSHR, E4F1, THBS3, IST1, BRAT1, NRCAM, TAOK2*
13	GO:0007155 cell adhesion	*NRCAM, MAG, CDH26, FIBCD1, RELL2, WNT1, TAOK2, GLI3, ONECUT2, NCAM1, THBS3, MALT1, IL20RB*
13	GO:0008283 cell population proliferation	*RPS6KA2, WNT1, PAX6, BABAM1, BRAT1, GLI3, CCND3, TAF8, MALT1, IL20RB, RASSF5, LDLRAP1, TSHR*
13	GO:0032879 regulation of localization	*TCP1, HAMP, SHISA8, GSTO1, HDAC3, PAX6, GLI3, ONECUT2, FRMD4A, RASSF5, CARD10, LDLRAP1, TOMM7*
12	GO:0040011 locomotion	*NRCAM, RASGEF1A, PAX6, GLI3, ONECUT2, EGR2, LMX1A, TSHR, CARD10, BRAT1, NCAM1, TAOK2*

12	GO:0003008 system process	*GSTO1, RPS6KA2, NRCAM, MAN2B1, MAG, HAMP, SOBP, EGR2, LMX1A, GATM, NPTXR, PAX6*
12	GO:0009056 catabolic process	*WDR45B, ELOC, FBXL18, GSTO1, MALT1, IRAK3, HAMP, NT5C3A, WNT1, QSOX1, VPS13C, TOMM7*
11	GO:0009719 response to endogenous stimulus	*GNA14, TSHR, KDM3A, GLI3, CCND3, ONECUT2, EGR2, WNT1, VPS13C, ARPC1B, WDTC1*
11	GO:0022402 cell cycle process	*BABAM1, CCND3, NUF2, JADE1, E4F1, PAX6, RPS6KA2, TAOK2, TRIM36, HDAC3, IST1*
11	GO:0040008 regulation of growth	*JADE1, RASAL1, MAG, HAMP, LMX1A, TSHR, E4F1, IST1, BRAT1, NRCAM, TAOK2*
11	GO:0045321 leukocyte activation	*SLAMF6, MALT1, CDH26, MFNG, HAMP, GLI3, CCND3, WNT1, TSHR, IL20RB, RASSF5*
11	GO:0051094 positive regulation of developmental process	*RASAL1, WNT1, PAX6, MAG, HAMP, GLI3, EGR2, TSHR, MALT1, IST1, NRCAM*
10	GO:0002520 immune system development	*SLAMF6, MCM3AP, IRAK3, MFNG, BABAM1, GLI3, WNT1, ANKLE1, TSHR, MALT1*
10	GO:0048589 developmental growth	*RASAL1, MAG, HAMP, GLI3, TAF8, WDTC1, TSHR, THBS3, IST1, NRCAM*
10	GO:0048646 anatomical structure formation involved in morphogenesis	*ISM1, PAX6, MFNG, MAG, GLI3, EGR2, WNT1, THBS3, NRCAM, CARD10*
	**MF – molecular function**	
19	GO:0016740 transferase activity	*RPS6KA2, IRAK3, MFNG, CHST8, GSTO1, TAOK2, GATM, UAP1L1, UBE2QL1, ARMT1, MCM3AP, MALT1, EZH1, CCND3, NT5C3A, EGR2, WDR83, TRIM36, E4F1*
15	GO:0016787 hydrolase activity	*MAN2B1, KIF17, NT5C3A, ARMT1, GNA14, KIF7, HDAC3, ANKLE1, PPM1M, MALT1, YBEY, DDX25, TCP1, PROCA1, RGS5*
12	GO:0036094 small molecule binding	*LMAN2L, IRAK3, QSOX1, RPS6KA2, DDX25, KIF17, TCP1, NT5C3A, TAOK2, GNA14, KIF7, UBE2QL1*
12	GO:0044877 protein-containing complex binding	*NUF2, GNA14, ARPC1A, GLI3, CDH26, ARPC1B, WDR45B, LDLRAP1, TSHR, TMEM70, IST1, RELL2*
12	GO:0097367 carbohydrate derivative binding	*MAG, IRAK3, FIBCD1, THBS3, RPS6KA2, DDX25, KIF17, TCP1, TAOK2, GNA14, KIF7, UBE2QL1*
10	GO:0098772 molecular function regulator activity	*CCND3, WNT1, ARHGAP39, RASGEF1A, HAMP, RASAL1, WDTC1, MALT1, RGS5, TAOK2*
10	GO:0003700 DNA-binding transcription factor activity	*PAX6, CREM, GLI3, ONECUT2, EGR2, ZNF496, LMX1A, E4F1, ZNF627, RFXANK*
	**CC – cellular component**	
23	GO:0031090 organelle membrane	*MTX1, RDH14, MPC1, GDAP1, LMAN2L, QSOX1, UQCRFS1, TMEM70, MRPL18, TOMM7, BORCS8, VPS13C, WTAP, MCM3AP, MFNG, IQCE, CHST8, TAOK2, GATM, WDR83, WNT1, NCAM1, LDLRAP1*
21	GO:0005576 extracellular region	*MAN2B1, MRPL18, QSOX1, TCP1, VPS13C, ARPC1B, GSTO1, GNA14, SLAMF6, GATM, IST1, ARPC1A, HAMP, WNT1, FIBCD1, RFXANK, NRCAM, ISM1, NCAM1, THBS3, MFNG*
16	GO:0005615 extracellular space	*MAN2B1, MRPL18, QSOX1, TCP1, VPS13C, ARPC1B, GSTO1, GNA14, SLAMF6, GATM, IST1, ARPC1A, HAMP, WNT1, FIBCD1,*

		MFNG
14	GO:0042995 cell projection	*NRCAM, KIF17, NCAM1, SHISA8, JADE1, CEP128, GLI3, KIF7, MAG, IQCE, RSPH3, TAOK2, LDLRAP1, BAALC*
12	GO:0043230 extracellular organelle	*MAN2B1, QSOX1, TCP1, VPS13C, ARPC1B, GSTO1, GNA14, SLAMF6, GATM, IST1, ARPC1A, WNT1*
12	GO:1903561 extracellular vesicle	*MAN2B1, QSOX1, TCP1, VPS13C, ARPC1B, GSTO1, GNA14, SLAMF6, GATM, IST1, ARPC1A, WNT1*

The FDR cutoff point was set to 0.1. N: the number of genes enriching each GO term.

Table S6. The ShinyGO results for genes/DMRs matching between uni- and multivariable regression analyses in BOTS.

Logistic regression analyses		
**BP – biological process**		
**N**	**High level GO category**	**Genes**
24	GO:0042221 response to chemical	*F7, STAT2, LMO3, RASGRP2, CDH13, TRPM2, MSN, IL34, HDAC4, TP73, HOXA2, FGFBP1, SLC26A5, CHST11, LPL, UMODL1, IGF2R, TXNIP, WNT10A, BAIAP3, TBC1D1, SH3BP4, FLNA, FEZ2*
23	GO:0048583 regulation of response to stimulus	*FLNA, F7, SH3BP1, MGRN1, BPIFB1, LAMB1, LPL, LMO3, FBLN1, MAP3K20, F10, CDH13, IL34, HDAC4, TP73, SH3BP4, FGFBP1, RGS3, TRPM2, CHST11, TBC1D1, STAT2, EMC10*
22	GO:0023051 regulation of signaling	*FLNA, SH3BP1, MGRN1, BPIFB1, LAMB1, LMO3, F7, FBLN1, MAP3K20, F10, CDH13, IL34, TP73, SH3BP4, FGFBP1, RGS3, TRPM2, CHST11, BAIAP3, TBC1D1, STAT2, EMC10*
21	GO:0009653 anatomical structure morphogenesis	*LAMB1, CDH13, MSN, DCHS1, HOXC4, FBLN1, FGFBP1, FLNA, MAP3K20, HOXA2, WNT10A, KDM6A, EMC10, PHOX2A, SLC26A5, CHST11, SH3BP1, TMIGD2, STAT2, RIPK4, FEZ2*
21	GO:0009893 positive regulation of metabolic process	*CNOT6, STAT2, HDAC4, FBLN1, TP73, EAPP, CAMK4, IL34, PHOX2A, TMIGD2, TPCN1, HOXC4, HOXA2, WNT10A, KDM6A, LPL, PRG2, MSN, SH3BP4, CDH13, GARIN5A*
21	GO:0032879 regulation of localization	*F7, TRPM2, MSN, FLNA, BAIAP3, FBLN1, LAMB1, FGFBP1, CDH13, IL34, MCOLN3, HDAC4, STK10, CLIC3, SLC26A5, TPCN1, TBC1D1, SH3BP1, LPL, EMC10, F10*
21	GO:0051234 establishment of localization	*MGRN1, TRPM2, MSN, SLC16A12, CLIC3, TXNIP, FLNA, BAIAP3, GGA2, SLC43A3, EMC10, MCOLN3, SH3BP1, SH3BP4, SLC26A5, APOF, TPCN1, IGF2R, TBC1D1, CDH13, CYB5R1*
19	GO:0040011 locomotion	*F7, LAMB1, UMODL1, FBLN1, FGFBP1, CDH13, MSN, IL34, FLNA, HDAC4, STK10, SH3BP1, HOXA2, TRPM2, DCHS1, SLC26A5, EMC10, F10, FEZ2*
19	GO:0006950 response to stress	*FLNA, F7, BPIFB1, F10, STAT2, LPL, FBLN1, TP73, MAP3K20, TRPM2, CAMK4, IL34, RNF166, SLC26A5, PRG2, TXNIP, GARIN5A, EMC10, HDAC4*
18	GO:0050793 regulation of developmental process	*HEMGN, MSN, IL34, LAMB1, LPL, FBLN1, HDAC4, TP73, HOXA2, WNT10A, SLC26A5, UMODL1, FLNA, LMO3, EMC10, TMIGD2, STAT2, CAMK4*
18	GO:0065008 regulation of biological quality	*FLNA, F7, CNOT6, F10, TRPM2, MSN, BOLA2, LPL, FBLN1, MCOLN3, GPR6, SLC26A5, TPCN1, BAIAP3, TBC1D1, SH3BP1, ANKRD9, RP1L1*
17	GO:0008283 cell population proliferation	*EAPP, IL34, STAT2, FBLN1, FGFBP1, CDH13, MSN, EMC10, TMIGD2, HDAC4, DCHS1, CHST11, TXNIP, TP73, CNOT6, SH3BP4, LAMB1*
17	GO:0044085 cellular component biogenesis	*LAMB1, MSN, WDR90, DCHS1, RP1L1, CDH13, TRPM2, BOLA2, FLNA, HDAC4, TP73, SH3BP1, TSPAN33, SLX9, CNOT6, FEZ2, LIM2*
17	GO:0048870 cell motility	*F7, LAMB1, UMODL1, FBLN1, FGFBP1, CDH13, MSN, IL34, FLNA, HDAC4, STK10, SH3BP1, TRPM2, DCHS1, SLC26A5, EMC10, F10*
17	GO:0051674 localization of cell	*F7, LAMB1, UMODL1, FBLN1, FGFBP1, CDH13, MSN, IL34, FLNA, HDAC4, STK10, SH3BP1, TRPM2, DCHS1, SLC26A5,*

		EMC10, F10
16	GO:0009605 response to external stimulus	*F7, BPIFB1, STAT2, LPL, CDH13, IL34, HDAC4, HOXA2, TRPM2, RNF166, SLC26A5, PRG2, FLNA, TXNIP, GARIN5A, FEZ2*
16	GO:0033036 macromolecule localization	*MSN, TSPAN33, TXNIP, GGA2, TNS4, EMC10, FLNA, SLC43A3, TRPM2, DCHS1, CHST11, APOF, BAIAP3, TBC1D1, SH3BP4, LPL*
16	GO:0051641 cellular localization	*MGRN1, TRPM2, MSN, TSPAN33, FLNA, BAIAP3, GGA2, EMC10, MCOLN3, DCHS1, TPCN1, IGF2R, TXNIP, TBC1D1, SH3BP4, CDH13*
15	GO:0002376 immune system process	*F7, BPIFB1, IL34, TMIGD2, STAT2, UMODL1, FLNA, MSN, STK10, TRPM2, CAMK4, RNF166, PRG2, GARIN5A, HDAC4*
15	GO:0009719 response to endogenous stimulus	*STAT2, LMO3, CDH13, F7, HDAC4, FGFBP1, TRPM2, SLC26A5, CHST11, LPL, UMODL1, TXNIP, WNT10A, SH3BP4, FLNA*
15	GO:0051239 regulation of multicellular organismal process	*IL34, FLNA, FBLN1, FGFBP1, TMIGD2, F7, HDAC4, TP73, PHOX2A, LPL, PRG2, SH3BP1, EMC10, GARIN5A, CAMK4*
14	GO:0040012 regulation of locomotion	*F7, FBLN1, LAMB1, FGFBP1, CDH13, IL34, FLNA, HDAC4, STK10, SLC26A5, SH3BP1, MSN, EMC10, F10*
13	GO:0009056 catabolic process	*INPP5A, PYGM, CNOT6, RNF166, LPL, TPCN1, HDAC4, CUL9, MSN, SH3BP4, ANKRD9, FEZ2, FLNA*
13	GO:0065009 regulation of molecular function	*TBC1D1, RASGRP2, SH3BP1, RIPK4, SH3BP4, IL34, FLNA, FBLN1, PPP1R1A, RGS3, UMODL1, TXNIP, HDAC4*
10	GO:0023057 negative regulation of signaling	*MGRN1, BPIFB1, LMO3, FBLN1, SH3BP4, RGS3, CHST11, TBC1D1, SH3BP1, STAT2*
10	GO:0044419 biological process involved in interspecies interaction between organisms	*BPIFB1, STAT2, IL34, RNF166, LPL, PRG2, TPCN1, FBLN1, IGF2R, GARIN5A*
	**MF – molecular function**	
12	GO:0098772 molecular function regulator activity	*TBC1D1, RASGRP2, SH3BP1, WNT10A, IL34, FLNA, FBLN1, PPP1R1A, UMODL1, TXNIP, SH3BP4, RGS3*
12	GO:0016740 transferase activity	*HDAC4, PYGM, MGRN1, CAMK4, RNF166, CHST11, CTU2, STK10, MAP3K20, CUL9, RIPK4, IGF2R*
11	GO:0036094 small molecule binding	*PYGM, CYB5R1, MAP3K20, STK10, CUL9, HAO2, CAMK4, DNHD1, RIPK4, IGF2R, APOF*
11	GO:0097367 carbohydrate derivative binding	*MAP3K20, TRPM2, LPL, STK10, CUL9, HAO2, CAMK4, DNHD1, RIPK4, PRG2, FGFBP1*
10	GO:0008289 lipid binding	*BAIAP3, F10, MCOLN3, GGA2, BPIFB1, LPL, TPCN1, IGF2R, RASGRP2, APOF*
	**CC – cellular component**	
27	GO:0005576 extracellular region	*PYGM, STK10, FBLN1, LAMB1, MGRN1, BPIFB1, SH3BP4, CDH13, MSN, CAMK4, CYB5R1, CLIC3, LPL, DNHD1, PRG2, FLNA, IGF2R, F7, F10, WNT10A, IL34, TMIGD2, APOF, UMODL1, EMC10, FGFBP1, CDCP2*
25	GO:0005615 extracellular space	*PYGM, STK10, FBLN1, LAMB1, MGRN1, BPIFB1, SH3BP4, CDH13, MSN, CAMK4, CYB5R1, CLIC3, LPL, DNHD1, PRG2, FLNA, IGF2R, F7, F10, WNT10A, IL34, TMIGD2, APOF, UMODL1, FGFBP1*
16	GO:0043230 extracellular organelle	*PYGM, STK10, FBLN1, LAMB1, MGRN1, BPIFB1, SH3BP4, CDH13, MSN, CAMK4, CYB5R1, CLIC3, DNHD1, PRG2, FLNA,*

		IGF2R
16	GO:1903561 extracellular vesicle	*PYGM, STK10, FBLN1, LAMB1, MGRN1, BPIFB1, SH3BP4, CDH13, MSN, CAMK4, CYB5R1, CLIC3, DNHD1, PRG2, FLNA, IGF2R*
15	GO:0030054 cell junction	*CDH13, MSN, FLNA, IGF2R, TNS4, DCHS1, TP73, SH3BP1, HOXC5, BAIAP3, HDAC4, RASGRP2, CAMK4, TSPAN33, LIM2*
10	GO:0031090 organelle membrane	*MCOLN3, EMC10, TPCN1, BAIAP3, IGF2R, GGA2, TRPM2, CHST11, STK10, CYB5R1*
10	GO:0042995 cell projection	*MSN, FEZ2, DNHD1, RP1L1, SH3BP1, CDH13, INPP5A, RASGRP2, TRPM2, FLNA*

The FDR cutoff point was set to 0.1. N: the number of genes enriching each GO term.

Table S7. The list of genomic locations for all DMRs identified as potential biomarkers in BOTS and hgOvCa, described in Table 4 in the manuscript.

Gene(s)/DMR	List of significant CpGs (genomic locations)
HMOX1(+)/ NA(−): chr22:g.(−)35776686-35777032	chr22:g.35776686; chr22:g.35776719; chr22:g.35777032
HMOX1(+)/NA(−):chr22:g.(−)35775959-35777032	chr22:g.35775959; chr22:g.35776269; chr22:g.35776686; chr22:g.35776719; chr22:g.35777032
TCN2(+)/PES1(−)/RP1-56J10.8(+): chr22:g.(−)31002067-31003655	chr22:g.31002067; chr22:g.31002795; chr22:g.31002892; chr22:g.31003010; chr22:g.31003127; chr22:g.31003227; chr22:g.31003283; chr22:g.31003571; chr22:g.31003587; chr22:g.31003655
TCN2(+)/PES1(−)/RP1-56J10.8(+): chr22:g.both 31002067-31003655	chr22:g.31002067; chr22:g.31002362; chr22:g.31002795; chr22:g.31002892; chr22:g.31002942; chr22:g.31002946; chr22:g.31002991; chr22:g.31003010; chr22:g.31003127; chr22:g.31003147; chr22:g.31003172; chr22:g.31003227; chr22:g.31003283; chr22:g.31003571; chr22:g.31003587; chr22:g.31003655
TCN2(+)/PES1(−)/RP1-56J10.8(+): chr22:g.both 31002362-31004367	chr22:g.31002362; chr22:g.31002795; chr22:g.31002892; chr22:g.31002942; chr22:g.31002946; chr22:g.31002991; chr22:g.31003010; chr22:g.31003127; chr22:g.31003147; chr22:g.31003172; chr22:g.31003227; chr22:g.31003283; chr22:g.31003571; chr22:g.31003587; chr22:g.31003655; chr22:g.31004367
NA(−)/NA(+): chr16:g.(−)880831-880831	chr16:g.880831
ABR(−)/NA(+): chr17:g.(−)1131424-1131781	chr17:g.1131424; chr17:g.1131781
NCAM1(+)/RP11-629G13.1(−): chr11:g.(−)112831728-112832249	chr11:g.112831728; chr11:g.112832045; chr11:g.112832152; chr11:g.112832159; chr11:g.112832249
AC006372.4 (+)/NA(−): chr7:g.(−)157258854-157259343	chr7:g.157258854; chr7:g.157259343
NPTXR(−)/NA(+): chr.22:g.(+)39240094-39240424	chr22:g.39240094; chr22:g.39240100; chr22:g.39240109; chr22:g.39240424
BAIAP3(+)/NA(−): chr.16:g.(−)1389301-1389301	chr16:g.1389301
IL34(+)/NA(−): chr16:g.both 70613332-70613944	chr16:g.70613332; chr16:g.70613522; chr16:g.70613641; chr16:g.70613716; chr16:g.70613725; chr16:g.70613738; chr16:g.70613789; chr16:g.70613944
IL34(+)/NA(−): chr16:g.(−)70613332-70613944	chr16:g.70613332; chr16:g.70613522; chr16:g.70613716; chr16:g.70613725; chr16:g.70613789; chr16:g.70613944
WNT10A(+)/NA(−): chr2:g.(+)219748780-219748780	chr2:g.219748780
NEU1(−)/SLC44A4(−)/NA(+): chr.6:g.(+)31827414-31834178	chr6:g.31827414; chr6:g.31827858; chr6:g.31827926; chr6:g.31828314; chr6:g.31828335; chr6:g.31828340; chr6:g.31828373; chr6:g.31828747; chr6:g.31829026; chr6:g.31829093; chr6:g.31829147; chr6:g.31829177; chr6:g.31829205; chr6:g.31829511; chr6:g.31829888; chr6:g.31829895; chr6:g.31829960; chr6:g.31830364; chr6:g.31830527; chr6:g.31830561; chr6:g.31830570; chr6:g.31830572; chr6:g.31830600; chr6:g.31830616; chr6:g.31830656; chr6:g.31830721; chr6:g.31830729; chr6:g.31830812;

	chr6:g.31830937; chr6:g.31830947; chr6:g.31831439; chr6:g.31831599; chr6:g.31832173; chr6:g.31832428; chr6:g.31832445; chr6:g.31832796; chr6:g.31832962; chr6:g.31833072; chr6:g.31833104; chr6:g.31833291; chr6:g.31833747; chr6:g.31833941; chr6:g.31834032; chr6:g.31834049; chr6:g.31834178

All genomic locations presented in this table correspond to the GRCh37 (hg19) assembly of the human genome.
